# Lignin-degrading-inspired dehydrogenative photocatalysis via reductive quenching of donor-acceptor covalent organic framework

**DOI:** 10.1126/sciadv.aef6109

**Published:** 2026-08-26

**Authors:** Xiangfeng Lin, Abdullahi Bello Umar, Jiaxian Zheng, Hankun Zheng, Wenjing Sang, Zhanhua Huang, Jie Wang, Yunqing Kang, Dong Jiang, Yusuke Yamauchi, Chaoqun Zhang, Guofeng Guan, Zhanhui Yuan

**Affiliations:** ^1^ College of Materials Engineering, Fujian Agriculture and Forestry University, Fuzhou 350002, P. R. China.; ^2^ Key Laboratory of Bio-based Material Science & Technology, Material Science and Engineering College, Northeast Forestry University, Harbin 150040, P. R. China.; ^3^ Australian Institute for Bioengineering and Nanotechnology (AIBN), The University of Queensland, Brisbane, QLD 4072, Australia.; ^4^ Department of Materials Process Engineering, Graduate School of Engineering, Nagoya University, Nagoya 464-8603, Japan.; ^5^ College of Material Science and Art Design, Inner Mongolia Agricultural University, Hohhot 010018, P. R. China.; ^6^ Key Laboratory for Biobased Materials and Energy of Ministry of Education, College of Materials and Chemical Engineering, South China Agricultural University, Guangzhou 510642, P. R. China.; ^7^ State Key Laboratory of Materials-Oriented Chemical Engineering, College of Chemical Engineering, Nanjing Tech University, Nanjing, 211816, China.; ^8^ College of Materials and Chemical Engineering, Minjiang University, Fuzhou, 350108, China.

## Abstract

Achieving a photocatalytic reductive quenching cycle that enables direct oxidation of the photocatalyst radical anion by H(I) species, without requiring stoichiometric oxidants or cocatalysts, remains a long-standing challenge in artificial photoredox catalysis. Here we report a bioinspired approach based on the lignin-degrading-mediated proton-coupled electron transfer (PCET) process, implemented through a perylene-based covalent organic framework (NPy-Per-COF) with precisely engineered donor-acceptor characteristics. This heterogeneous photocatalyst sustains a reductive quenching cycle in which controlled radical generation, catalyst regeneration and hydrogen evolution are intrinsically coupled. The system enables dehydrogenative radical cascade reactions between lignin-inspired precursors and structurally diverse unsaturated partners, providing access to a broad range of functionalized aromatic architectures. Further synthetic modifications and in silico analyses confirm the biological relevance and medicinal potential of the synthesized scaffolds. Beyond model substrates, the photocatalytic system can be extended to lignin-derived motifs, highlighting its potential relevance to complex biomass-related chemical space. Mechanistic investigations support the view that donor-acceptor polarization within the COF promotes efficient charge separation, stabilizes the photocatalyst radical anion and facilitates a PCET-initiated reductive quenching cycle. By abstracting and translating key elements of enzymatic redox control into a fully artificial, heterogeneous photocatalytic framework, this work establishes mechanistically informed design guideline for photocatalytic reductive quenching cycle and advances the development of bioinspired systems for controlled dehydrogenative transformations.

## INTRODUCTION

**Fig. 1.**
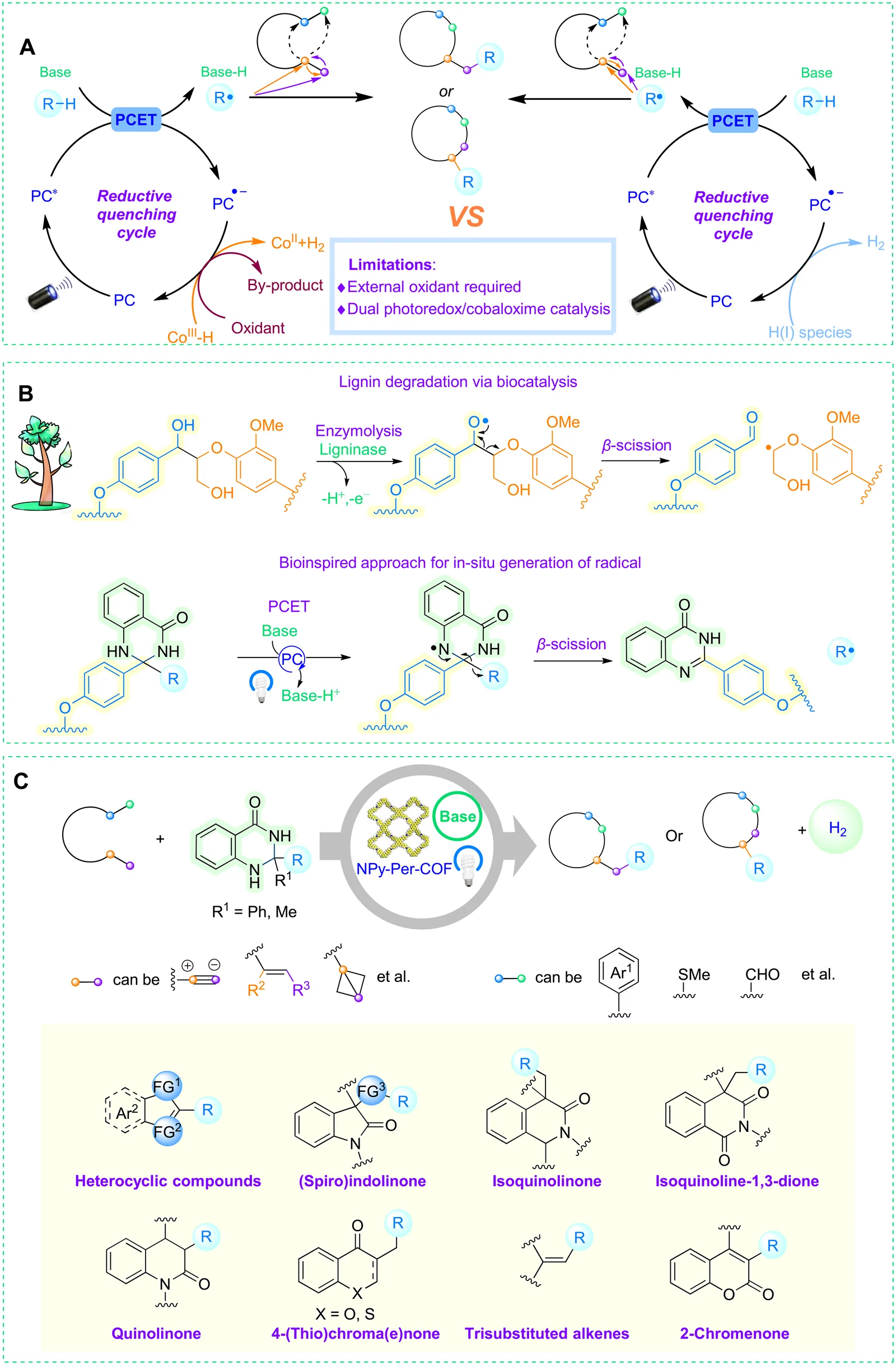
Lignin-degrading-inspired dehydrogenative photocatalysis via reductive quenching enabled by a D-A COF. (**A**) Traditional reductive quenching cycle and dehydrogenative reductive quenching cycle. (**B**) Biocatalytic lignin degradation mediated by lignin-degrading enzymes and bioinspired approach for in situ generation of C-radical. (**C**) Dehydrogenative photocatalysis via reductive quenching of D-A COF.

Controlling proton-coupled electron transfer (PCET) and radical reactivity is a central challenge in modern photocatalysis. Although visible-light photocatalysis has enabled a wide range of single-electron transformations under mild conditions, (*1*–*5*) most synthetic systems rely on oxidative quenching cycle to sustain catalytic turnover (*6*–*13*). In contrast, photocatalytic reductive quenching cycle, initiated by single electron transfer (SET) reduction of H(I) species, remains challenging. A key limitation arises from inefficient catalyst regeneration, as the fate of the photocatalyst radical anion is often poorly controlled, leading to catalyst deactivation or the need for external oxidants (Fig. 1A) (*14*–*19*). While the use of stoichiometric oxidants or dual photoredox/metal hydride systems can restore turnover, these strategies frequently introduce additional reaction complexity, generate stoichiometric by-products, or display strong sensitivity to reaction conditions (*12*, *20*–*35*). Consequently, a reductive quenching cycle that enables direct oxidation of the photocatalyst radical anion by H(I) species without relying on external oxidants or cocatalysts, remains a long-standing and unresolved challenge in photocatalytic system design.

Nature offers a compelling blueprint for addressing this challenge. In a lignin-degrading biosynthetic pathway, such as lignin peroxidase employed by white-rot fungi, chemically demanding bond-breaking processes proceed through a tightly regulated single-electron pathway (Fig. 1B) (*36*–*39*). A defining feature of these enzymatic systems is their reliance on substrates initiated via PCET-mediated oxidation, producing transient aryl or benzylic radical species that undergo selective bond cleavage, rearrangement or downstream radical transformations (*40*–*43*). Crucially, electron and proton transfer (ET and PT) are intrinsically coupled within the enzymatic redox cycle, enabling efficient turnover without the accumulation of high-energy intermediates or the need for stoichiometric reagents. Despite the conceptual appeal of this system, translating a controlled PCET process into artificial photocatalytic systems, particularly heterogeneous platforms, has remained elusive. Inspired by this biodegradation pathway, we envisioned a bioinspired approach wherein lignin-inspired dihydroquinazolinones serve as latent radical precursors (*44*–*54*). Owing to their low oxidation potential, these compounds are poised to undergo a facile PCET or SET process under photocatalytic conditions, enabling in-situ generation of alkyl radicals and mimicking the PCET pathway in lignin biodegradation.

From the perspective of artificial photocatalysis, emulating biosynthetic redox control requires both efficient PCET or SET initiation under a reductive quenching cycle to generate defined C-radicals and a controlled pathway for regeneration of the photocatalyst radical anion via H(I) species reduction, thereby closing the catalytic cycle. Covalent organic framework (COF) presents an attractive platform to meet these requirements, owing to their modular architectures, extended π-conjugation, and tunable electronic structures. However, most reported COF-based photocatalysts operate predominantly through oxidative quenching or energy transfer pathways, and examples that sustain a true reductive quenching cycle remain rare. Our previous work demonstrated that a pyrene-based covalent organic framework (Py-COF) can mediate visible-light-driven cascade transformations between tryptamine-derived isocyanides and phosphine oxides, wherein SET occurs between the COF radical anion and an H(I)-base adduct, leading to hydrogen evolution (*55*). While this finding highlights the feasibility of coupling reductive quenching to H(I) species reduction in a heterogeneous framework, the strongly electron-donating nature of the pyrene unit limits its ability to efficiently initiate PCET or SET oxidation of a radical precursor (*56*–*63*). In contrast, perylene units possess a lower-lying Lowest Unoccupied Molecular Orbital (LUMO) and enhanced oxidative capability, making them more suitable for these oxidations (*64*). These considerations motivate the development of donor-acceptor (D-A) COF that spatially and electronically decouple radical generation from catalyst regeneration, thereby enabling a more efficient reductive quenching cycle (*65*–*70*).

Inspired specifically by the PCET logic invovled in the lignin degradation, herein we report a perylene-pyrene-based D-A covalent organic framework, NPy-Per-COF. By integrating electron-accepting perylene units with electron-donating pyrene motifs within a crystalline framework, the COF achieves efficient charge separation, stabilizes the photocatalyst radical anion and enables a PCET-initiated reductive quenching cycle accompanied by hydrogen evolution. This bioinspired photocatalytic system enables dehydrogenative radical cascade transformations under visible light across various unsaturated compounds, enabling access to a broad range of functionalized aromatic scaffolds, including heterocycles, (spiro)indolinones, quinolinones, isoquinolinones, isoquinoline-1,3-diones, trisubstituted alkenes and (thio)chroma(e)nones, while maintaining a closed redox cycle without external oxidants or cocatalysts. The biological relevance and medicinal potential of these scaffolds are collectively confirmed through further synthetic modifications and in silico analyses. Moreover, the utility of the photocatalytic system extends beyond model substrates to include lignin-derived motifs, thereby broadening its relevance to complex, biomass-related chemical space. Mechanistic investigations further identify D-A polarization as a critical factor governing radical reactivity and catalyst regeneration, establishing mechanistically informed design guidelines for reductive quenching cycles inspired by enzymatic redox logic.

## RESULTS

### Synthesis and characterizations of the COFs

**Fig. 2.**
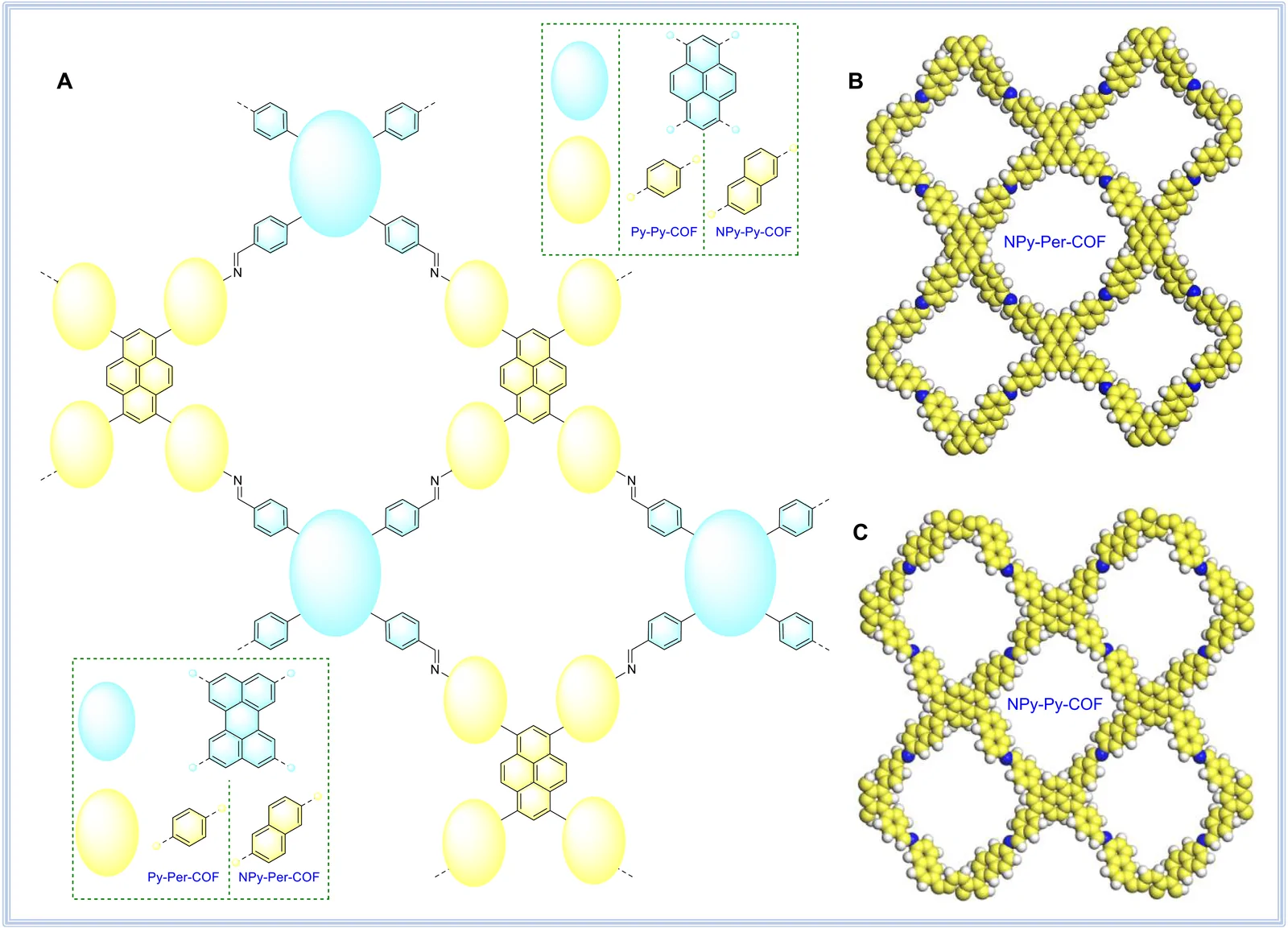
Structures of perylene and pyrene-based COF. (**A**) Chemical structures of perylene and pyrene-based COF. (**B** and **C**) Graphical view of one layer of perylene and pyrene-based COF simulated by Materials Studio. (yellow, C; blue, N; white, H).

Initially, we synthesized NPy-Per-COF through the condensation reaction between 4,4′,4″,4‴-(perylene-2,5,8,11-tetrayl)tetrabenzaldehyde (Per-CHO) and 1,3,6,8-tetrakis(2-aminonaphthyl)pyrene (NPy-NH_2_) (Fig. 2 and fig. S1). In contrast to the previously reported Py-Per-COF, NPy-Per-COF exhibits enhanced electron-withdrawing characteristics and a more extended π-conjugated framework, endowing it with a higher propensity for electron abstraction during the PCET process. Notably, the D-A configuration in NPy-Per-COF is further optimized relative to NPy-Py-COF, owing to the lower-lying LUMO localized on the perylene unit rather than the pyrene moiety. Powder X-ray diffraction (PXRD) analysis reveals three prominent diffraction peaks for NPy-Per-COF, with the most intense reflection at 4.36° and additional peaks at 6.60° and 8.84°, which are indexed to the (110), (200) and (220) planes, respectively (fig. S2A). The experimental PXRD pattern shows excellent agreement with the simulated eclipsed AA-stacked model (P1 space group) generated using Materials Studio. Pawley refinement affords unit-cell parameters of *a* = 28.1 Å, *b* = 30.7 Å, *c* = 3.8 Å, α = 97.9°, β = 74.8° and γ = 89.5°, with good agreement factors (*R_wp_* = 4.77% and *R_p_* = 3.09%) (table S1). For comparison, we also synthesized the NPy-Py-COF via the condensation reaction between 4,4′,4″,4‴-(pyrene-1,3,6,8-tetrayl)tetrabenzaldehyde (Py-CHO) and 1,3,6,8-tetrakis(2-aminonaphthyl)pyrene (NPy-NH_2_). Pawley refinement and simulation of NPy-Py-COF are shown in fig. S2B and table S2. The robust framework integrity of NPy-Per-COF was evidenced by negligible changes in the PXRD patterns after exposure to harsh chemical environments, including 14 M NaOH, triethylamine, concentrated HCl, trifluoroacetic acid and boiling water (fig. S3), highlighting its exceptional chemical stability. Fourier-transform infrared (FT-IR) spectroscopy confirms the successful formation of imine linkages, as indicated by the disappearance of the C=O stretching bands of Per-CHO (1697 cm^−1^) and the N-H vibrations of NPy-NH_2_ at 3370 cm^−1^, accompanied by the emergence of characteristic C=N stretching bands at 1590 cm^−1^ (fig. S4A). Similar spectral features were observed for NPy-Py-COF (fig. S4B). Solid-state ^13^C CP/TOSS NMR spectroscopy further corroborates imine formation, with resonances at 159.7 and 154.8 ppm assigned to the C=N carbons of NPy-Per-COF and NPy-Py-COF, respectively (fig. S5). Thermogravimetric analysis (TGA) demonstrates that NPy-Per-COF remains thermally stable up to 350°C under a nitrogen atmosphere, highlighting its excellent thermal stability (fig. S6). Scanning electron microscopy (SEM) images demonstrate that NPy-Per-COF exhibits a rod-like morphology (fig. S7), while dynamic light scattering (DLS) measurements give a Z-average particle diameter of 186.9 nm (fig. S8). High-resolution transmission electron microscopy (HR-TEM) further confirms the high crystallinity of NPy-Per-COF, revealing well-defined lattice fringes and a periodic pore structure with a spacing of approximately 1.8 nm (fig. S9). Consistent with these observations, N_2_ sorption measurements show a mesoporous architecture with a Brunauer-Emmett-Teller (BET) surface area of 1175 m^2^ g^−1^, a pore volume of 0.65 cm^3^ g^−1^ and a pore size centered at 1.43 nm (fig. S10), in good agreement with both the simulated structure and HR-TEM analysis.

### Reaction Optimizations

**Fig. 3.**
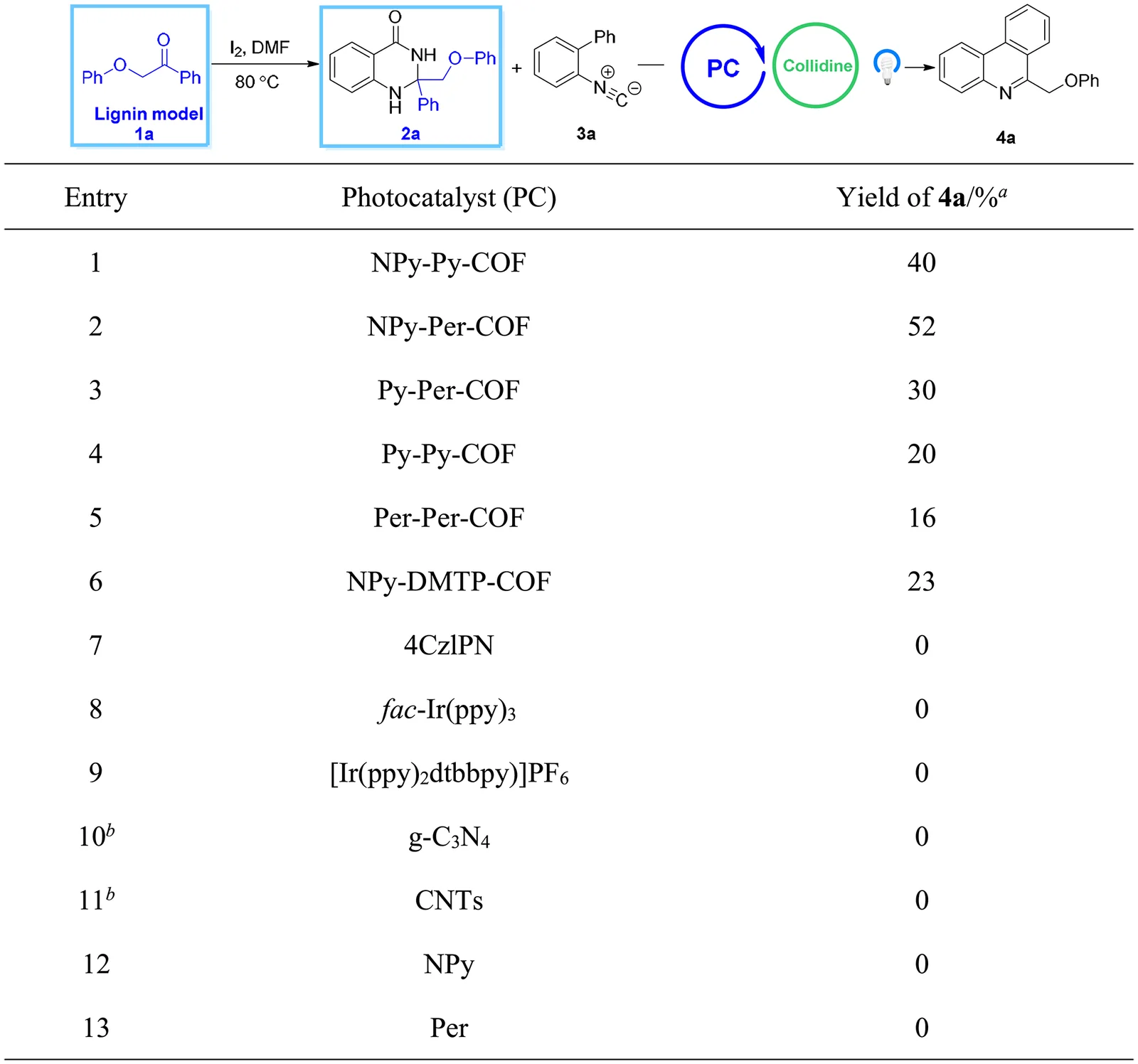
Optimizations of the reaction. All reactions were carried out on a 0.1 mmol scale with 1.0 eq. isocyanide **3a**, 2.0 eq. dihydroquinazolinone **2a**, 5 mol% catalyst and 2.0 eq. collidine in 1.0 mL CH_3_CN under irradiation of blue LEDs with N_2_ protection at room temperature. 4CzlPN = 2,4,5,6-tetrakis(carbazol-9-yl)-1,3-dicyanobenzene. *^a^*Isolated yield. *^b^*The reaction was performed with 5 mg photocatalyst.

The photocatalytic activities of NPy-Py-COF and NPy-Per-COF were first evaluated using a representative dehydrogenative radical cascade reaction under visible-light irradiation (420 nm, blue LED) at ambient temperature (Fig. 3). Both COFs catalyzed the formation of product **4a** as the major product, affording yields of 40% and 52% for NPy-Py-COF and NPy-Per-COF in CH_3_CN, respectively at ambient temperature (Fig. 3, entries 1 and 2). In contrast, structurally related COF lacking the naphthyl substituent, Py-Per-COF, prepared via Huang’s method (*66*) from Py-NH_2_ and Per-CHO, gave **4a** in only 30% yield (Fig. 3, entry 3). These results underscore the critical role of the naphthyl group in modulating the excited-state redox properties of the framework. Furthermore, an electron-donating Py-Py-COF synthesized according to Auras’s method (*65*) via condensation of Py-NH_2_ and Py-CHO or a more electron-withdrawing Per-Per-COF prepared via Huang’s method (*71*) from Per-NH_2_ and Per-CHO, gave lower yields of **4a** under the same conditions (Fig. 3, entries 4 and 5). These results indicate that photocatalytic performance is highly sensitive to donor-acceptor organization within the COF scaffold. A recently reported NPy-DMTP-COF by our group likewise exhibited limited activity under identical conditions (Fig. 3, entry 6) and a range of representative homogeneous and heterogeneous photocatalysts proved ineffective (Fig. 3, entries 7 to 11).

To elucidate the structure-property relationships, we performed comparative DFT and TD-DFT calculations on the D-A pairs derived from NPy-Per-COF and three structurally related analogues (Fig. 3, entries 2 to 5). These were conducted at the B3LYP/6-311G(d,p) and CAM-B3LYP/6-311G(d,p) levels of theory, respectively (*72*–*75*). As shown in fig. S11, the frontier orbitals of the four D-A pairs reveal clear differences in donor-acceptor character, with the Highest Occupied Molecular Orbital (HOMO) mainly localized on the donor-rich pyrene segment and the LUMO preferentially distributed over the acceptor-rich perylene segment. This spatial separation is most pronounced for NPy-Per-COF, which also exhibits the smallest HOMO-LUMO gap (2.890 eV), whereas Py-Per-COF, Py-Py-COF, and Per-Per-COF show larger gaps of 3.147, 3.143 and 3.306 eV, respectively, indicating that NPy-Per-COF has the most favorable frontier-orbital alignment for photoinduced charge separation. The electrostatic potential maps in fig. S12 show the same overall trend, with NPy-Per-COF possessing the largest dipole moment (2.448 D), compared with Py-Per-COF (1.838 D), Py-Py-COF (1.652 D), and Per-Per-COF (1.475 D), supporting its stronger intrinsic donor-acceptor polarization.

More direct quantitative evidence is provided by the excited-state descriptors (*76*). As summarized in table S3, NPy-Per-COF shows the largest hole-electron separation distance *D* (13.522 Å), together with the lowest overlap index *S_r_* (0.505) and the smallest *S*/*D* value (0.0373 Å^−1^), while Py-Per-COF, Py-Py-COF, and Per-Per-COF display progressively less favorable combinations of these parameters. This trend is further visualized in fig. S13A, which presents the computed charge density difference (Δρ) between the ground and excited states, where blue and red indicate an increase and a decrease in electron density, respectively. To probe the underlying electronic structure and charge-transfer dynamics, the distance of charge transfer (*D_CT_*) based on the electron density variation and the amount of transferred electron (*qCT*) during excitation have been calculated (fig. S13B). A larger *D_CT_* and *qCT* denote stronger D-A interactions. Among these candidates, NPy-Per-COF features the largest *D_CT_* value (17.004 Å) and the highest transferred-electron amount (0.951 e). The integrated *D_CT_* and q*CT* values, acting as descriptors for the D-A interaction, decrease in the order: NPy-Per-COF, followed by Py-Per-COF, then Per-Per-COF, and finally Py-Py-COF, which is similar to the trend of the *S*/*D* values (table S3) and charge centroids (fig. S13B). Therefore, NPy-Per-COF possesses the strongest D-A interaction, inferring the superior photocatalytic performance of the resulting framework (*77*, *78*). The calculated vertical and adiabatic electron affinities in fig. S14 show that NPy-Per-COF possesses the highest *E_ea_* values in the series, indicating the most favorable stabilization of the one-electron-reduced state (*79*). These results establish a clear structure-property relationship within this COF series, showing that stronger D-A polarization is associated with more efficient excited-state charge separation and more favorable electron uptake, in agreement with the superior photocatalytic performance of NPy-Per-COF in Fig. 3. Consistent with the computational results, the lack of activity in the molecular precursors (NPy and Per) further supports the importance of the COF in enabling the present photocatalytic transformation (Fig. 3, entries 12 and 13).

To elucidate the origin of these pronounced activity differences, the redox properties of the photocatalysts and substrates were examined by cyclic voltammetry (CV). NPy-Per-COF displays a ground-state reduction potential of −0.93 V, corresponding to the PC/PC^•-^ redox couple (fig. S15D). On the basis of an optical excitation energy (E^0–0^ = 2.38 eV) derived from UV-vis diffuse reflectance and emission spectra (fig. S15, B and F), the excited-state oxidation potential of NPy-Per-COF^*^ is estimated to be 1.45 V *vs* SCE. This experimentally derived excitation energy is corroborated by TD-DFT calculations, which predicts a low-energy S_0_ → S_1_ transition at 539 nm. This value is in excellent agreement with the experimental absorption maximum of 551 nm (fig. S16), thereby validating the robustness of the electronic-structure model.

By comparison, the oxidation potential of substrate **2a** is 1.16 V, but it decreases to 0.69 V in the presence of DABCO (fig. S15E). This energetic alignment enables a thermodynamically favourable PCET process, involving electron transfer from **2a** to the excited NPy-Per-COF^*^ coupled with proton transfer to the base, thereby generating an N-radical and the NPy-Per-COF^•-^. In contrast, the poor perfomance of NPy-Py-COF, *fac-*Ir(ppy)_3_, [Ir(ppy)_2_(dtbbpy)]PF_6_ or 4CzlPN is likely associated with their insufficient excited states oxidative power relative to NPy-Per-COF. The excited-state reduction potentials are E_1/2_(PC^*^/PC^•-^) = 1.39 V *vs* SCE for NPy-Py-COF (fig. S15, A and C), E_1/2_(PC^*^/PC^•-^) = 0.31 V *vs* SCE for *fac-*Ir(ppy)_3_, E_1/2_(PC^*^/PC^•-^) = 0.66 V *vs* SCE for [Ir(ppy)_2_(dtbbpy)]PF_6_ and E_1/2_(PC^*^/PC^•-^) = 1.35 V *vs* SCE for 4CzlPN (*80*, *81*). These results collectively underscore the importance of precise excited-state redox matching in enabling PCET-initiated oxidation of dihydroquinazolinones via D-A COF.

The energy band structures of organic semiconductors play a decisive role in governing their photoredox reactivity. To further elucidate the origin of the distinct photocatalytic performances, the electronic band structures of NPy-Per-COF and NPy-Py-COF were investigated using UV-vis diffuse reflectance spectroscopy and valence-band X-ray photoelectron spectroscopy (VB-XPS). Tauc plot analysis revealed optical band gaps (*E_g_*) of 2.38 eV for NPy-Per-COF and 2.23 eV for NPy-Py-COF (fig. S17, A to C). VB-XPS measurements afforded valence band potentials *E*_VB,XPS_ of 1.6 V and 2.2 V for NPy-Per-COF and NPy-Py-COF (fig. S17, D and E). These values were converted to the normal hydrogen electrode (NHE) scale using the equation *E*_VB,NHE_ = φ + *E*_VB,XPS_ - 4.44, where φ is the instrument work function (4.20 eV). The valence-band potentials were determined to be 1.36 V for NPy-Per-COF and 1.96 V for NPy-Py-COF. Based on these values, the corresponding conduction-band potentials were estimated to be −1.02 V for NPy-Per-COF and − 0.27 V for NPy-Py-COF (fig. S17F). This substantial difference indicates that the radical anion of NPy-Per-COF possesses a markedly stronger reducing power than that of NPy-Py-COF. Consequently, NPy-Per-COF^•-^ is better suited to reduce H(I) species to hydrogen, thereby enabling efficient regeneration of the photocatalyst radical anion and sustaining a closed reductive quenching cycle. In contrast, the shallower conduction band of NPy-Py-COF accounts for its diminished ability to drive H(I) species reduction, consistent with its inferior catalytic performance.

Condition screening further supports the proposed PCET-initiated mechanism. Among the bases examined, DABCO proved most effective in promoting the reaction, consistent with its role in facilitating proton transfer during N-radical generation (table S4, entry 1–5). The reaction also displays a pronounced solvent dependence, with chlorinated solvents generally providing superior performance and CHCl_3_ affording the highest yield. (table S4, entry 6–10). No product formation was observed in the absence of light or under an air atmosphere, confirming the photochemical nature of the process and the sensitivity of the catalytic cycle to oxygen (table S4, entries 11 and 12). In addition, the presence of base and sufficient catalyst loading were both essential to sustain the cascade transformation (table S4, entries 13 and 14). Collectively, these results establish the well-optimized reaction conditions using NPy-Per-COF as the catalyst and DABCO as the base in CHCl_3_ at room temperature (table S4, entry 10).

### Substrate scope

**Fig. 4.**
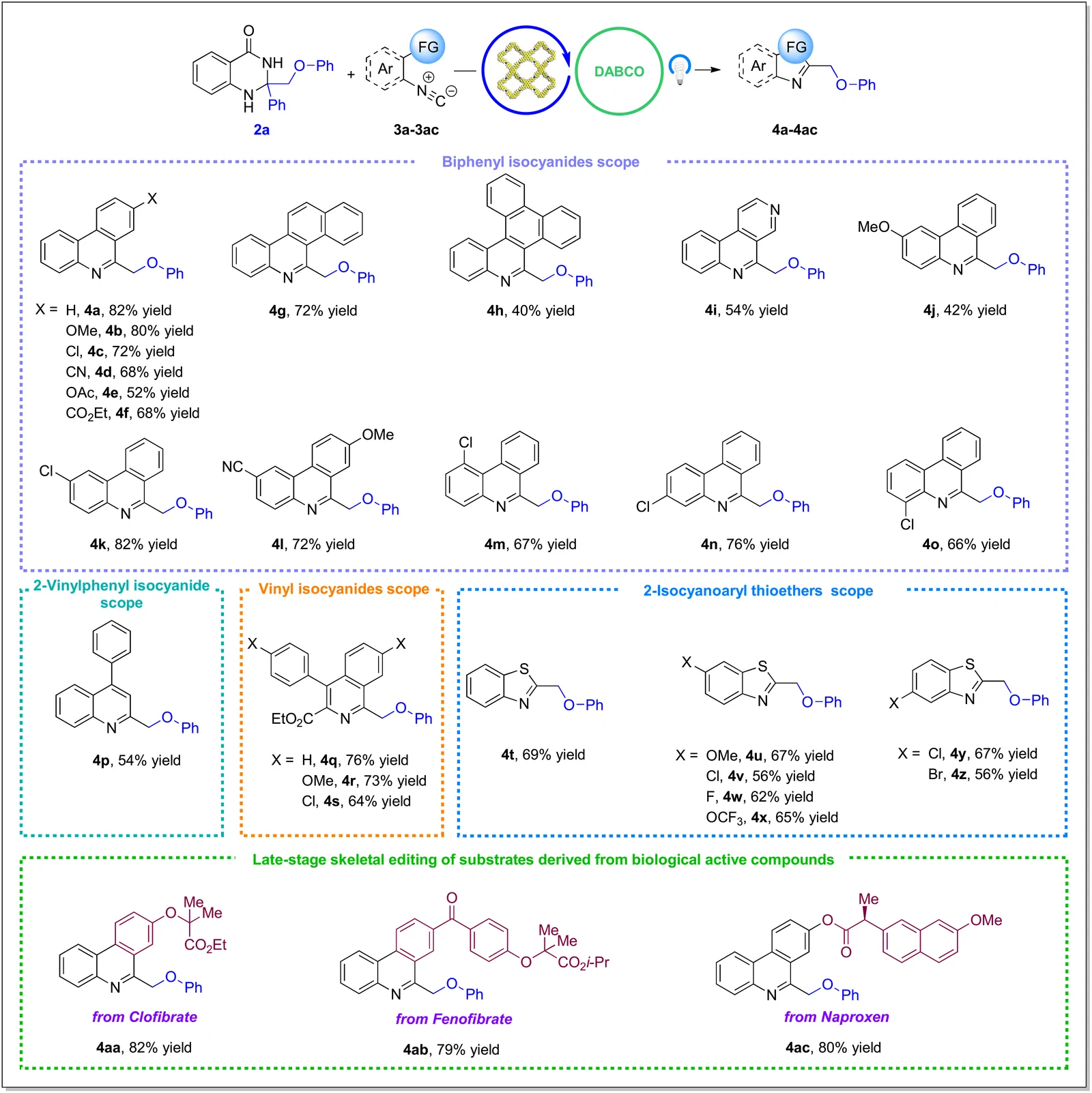
Substrate scope of isocyanides in the enzyme-inspired photocatalytic cascade reactions. All reactions were carried out on a 0.2 mmol scale with 1.0 eq. isocyanide **3a-3ac**, 2.0 eq. dihydroquinazolinone **2a**, 5 mol% NPy-Per-COF and 2.0 eq. DABCO in 4.0 mL CHCl_3_ under irradiation of blue LEDs with N_2_ protection at room temperature. Isolated yield.

Under the optimized reaction conditions, the generality of the photocatalytic cascade was evaluated using a diverse set of isocyanides (Fig. 4). The reaction proved applicable to a range of biphenyl isocyanides bearing diverse substituents, including alkoxy, halogen, cyano, acyloxy and alkoxycarbonyl groups, affording the corresponding 9-phenanthridines **4a-4f** in 52–82% yield. Both electron-donating and electron-withdrawing substituents were well tolerated at either the *para*- or *meta*-position of the benzene ring. Substrates featuring 2-naphthyl, 9-phenanthryl, and 4-pyridyl substituents were also readily accommodated, delivering 9-phenanthridines **4g-4i** up to 72% yield. Furthermore, biphenyl isocyanides **3j-3o** bearing electron-donating or electron-withdrawing substituents at any position on the Ar moiety also underwent efficient transformation. Vinyl-substituted isocyanides, including 2-vinylphenyl isocyanide and related derivatives, participated in analogous cascade processes to furnish quinoline and isoquinoline scaffolds **4p-4s** in moderate to good yields. Sulfur-containing substrates were also compatible, with 2-isocyanoaryl thioethers delivering benzothiazoles **4t-4z** under the standard conditions. The mild reaction conditions further enabled late-stage modification of complex bioactive motifs, as demonstrated by isocyanides derived from Clofibrate, Fenofibrate and Naproxen, which were transformed into the corresponding 9-phenanthridines **4aa-4ac** in high yields of 79–82%.

**Fig. 5.**
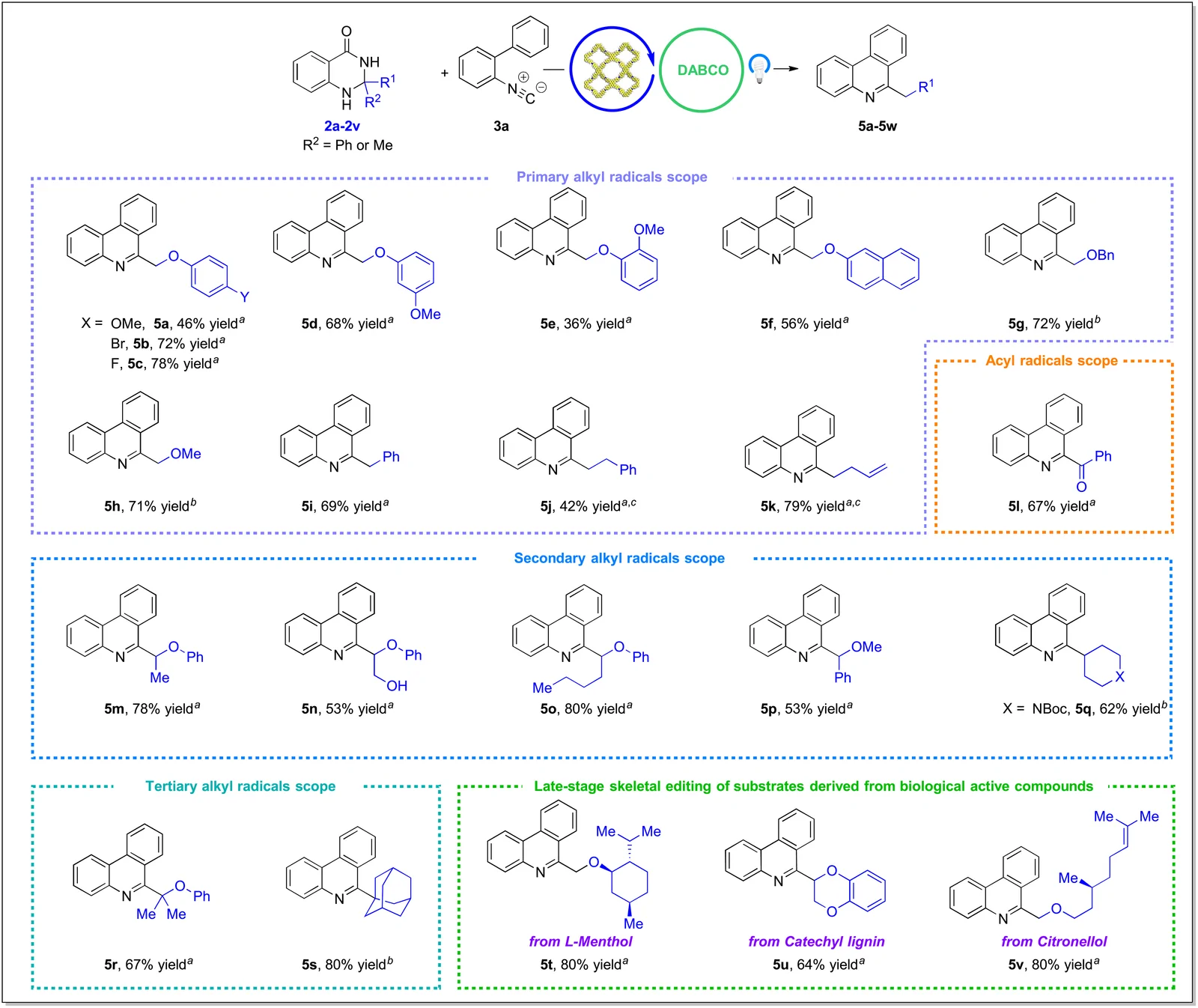
Substrate scope of dihydroquinazolinones in the enzyme-inspired photocatalytic cascade reactions. All reactions were carried out on a 0.2 mmol scale with 1.0 eq. isocyanide **3a**, 2.0 eq. dihydroquinazolinones **2a-2v**, 5 mol% NPy-Per-COF and 2.0 eq. DABCO in 4.0 mL CHCl_3_ under irradiation of blue LEDs with N_2_ protection at room temperature. Isolated yield. *^a^*R^2^ is phenyl. *^b^*R^2^ is methyl. *^c^*The reaction was carried out on a 0.2 mmol scale with 1.0 eq. isocyanide **3a**, 2.0 eq. dihydroquinazolinones **2j-2 k**, 5 mol% NPy-Per-COF, 2.0 eq. DBU and 2.0 eq. LiBr in 4.0 mL CHCl_3_ under irradiation of blue LEDs with N_2_ protection at room temperature.

The generality of the photocatalytic cascade was next examined with respect to the dihydroquinazolinone substrates, using isocyanide **3a** as fa representative coupling partner (Fig. 5). A broad range of primary alkyl-substituted dihydroquinazolinones **2a-2h** bearing diverse functional groups underwent efficient transformation to furnish the corresponding 9-phenanthridines **5a-5h**. Benzylic substrates were likewise competent, delivering product **5i** through a C(*sp^3^*)-centred radical cascade. Notably, our method could be implemented with less-activated radical precursors resulting in the photosynthesis of **5j-5k**. Acyl-substituted dihydroquinazolinones **2l** and a series of secondary alkyl radical precursors **2m-2q** were also compatible under the standard conditions. In particular, substrates derived from γ-substituted lignin motif **2n** underwent smooth conversion to give product **5n** in moderate to high yields, highlighting the relevance of the reaction manifold to lignin-inspired structures. Even tertiary reaction sites could trigger the targeted C(*sp^3^*)-C bond-forming reaction to afford the 9-phenanthridines **5r-5s**. Furthermore, dihydroquinazolinones derived from complex natural products, including L-menthol, Catechyl lignin and Citronellol, were efficiently transformed into the corresponding 9-phenanthridines **5t**-**5v** in yields of 64–80%, demonstrating the robustness of the system toward structurally complex and biologically relevant motifs.

**Fig. 6.**
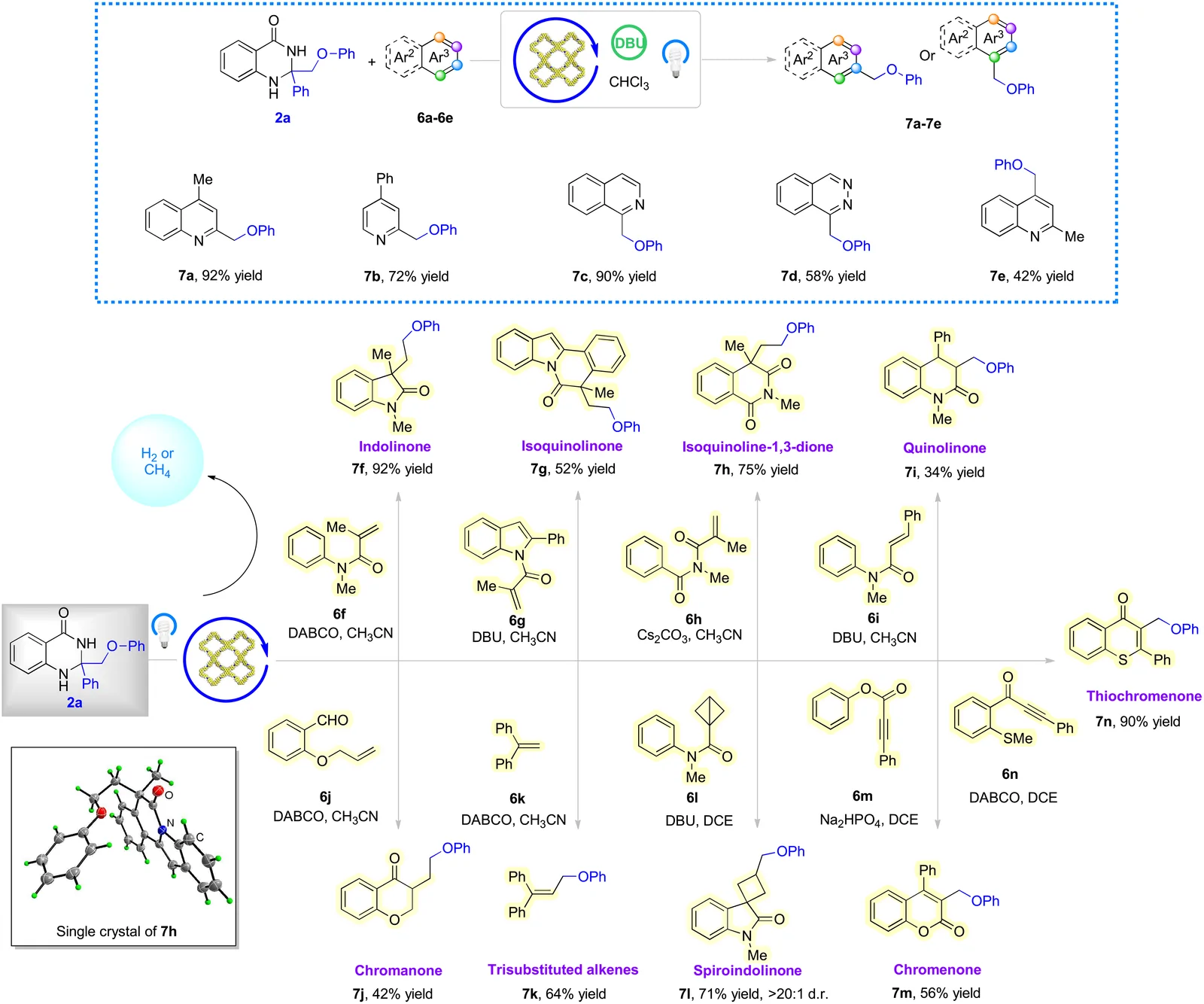
Substrate scope of other unsaturated compounds in the enzyme-inspired reductive-quenching photocatalytic cascade reactions. All reactions were carried out on a 0.2 mmol scale with 1.0 eq. unsaturated compounds **6a-6m**, 2.0 eq. dihydroquinazolinone **2a**, 5 mol% NPy-Per-COF and 2.0 eq. base in 4.0 mL solvent under irradiation of blue LEDs with N_2_ protection at room temperature. Isolated yield.

To further probe the versatility of the photocatalytic system, its applicability was extended to heterogeneous photocatalytic dehydrogenation of heteroaromatic compounds (Fig. 6). A range of *N*-heterocycles proved competent under the standard conditions. For instance, 4-substituted quinoline **6a** and pyridine **6b** underwent efficient dehydrogenative coupling with **2a** to afford the corresponding 2,4-disubstituted quinoline **7a** and pyridine **7b** in high yields. Other heteroaromatic frameworks, including isoquinoline **6c** and phthalazine **6d**, were likewise compatible, furnishing the corresponding 1-substituted products **7c**-**7d** in moderate to high yields. In addition, a 2-substituted quinolone **6e** participated effectively in the transformation to give the 2,4-disubstituted quinoline **7e**. Collectively, these results demonstrate that the bioinspired reductive quenching cycle is not limited to a single cascade manifold but can be extended to distinct heterocycle dehydrogenation processes.

We next evaluated the generality of the photocatalytic platform toward dehydrogenation-cyclization cascade reactions involving structurally diverse unsaturated compounds (Fig. 6). Activated alkenes readily participated in the transformation. *N*-arylacrylamide substrates **6f** underwent efficient β-addition followed by cyclization to furnish indolinone **7f** in high yield, while 2-aryl-*N*-acryloyl indoles **6 g** and *N*-acryloyl benzamides **6h** afforded isoquinolinone and isoquinoline-1,3-dione derivatives **7g** and **7h** through related radical cascade pathways. Beyond terminal alkenes, internal alkenes were also compatible with the catalytic system. For example, the substrate **6i** underwent α-addition followed by cyclization and dehydrogenation to give dihydroquinolin-2-one **7i**. Electron-rich alkenes likewise participated in analogous 6-exo-trig cyclization/dehydrogenation sequences to deliver product **7j**, and notably, even an olefin **6k** proved to be a viable substrate, affording trisubstituted alkene **7k** under the heterogeneous photocatalytic conditions. The scope of unsaturated partners could be further extended to strained and cumulatively activated motifs. Bicyclo[1.1.0]butane (BCB) **6l** underwent efficient ring-opening cascade reactions to deliver spiroindolinone **7l** in high yield, highlighting the ability of the system to engage high-strain carbon frameworks. Unsaturated alkynes were also competent coupling partners. Chromenone **7m** and thiochromenone **7n** were obtained in moderate to excellent yields, with the latter transformation accompanied by methane release. The structure of isoquinoline-1,3-dione **7h** was unambiguously confirmed by single-crystal X-ray diffraction (fig. S30).

### Practicality of the reactions

**Fig. 7.**
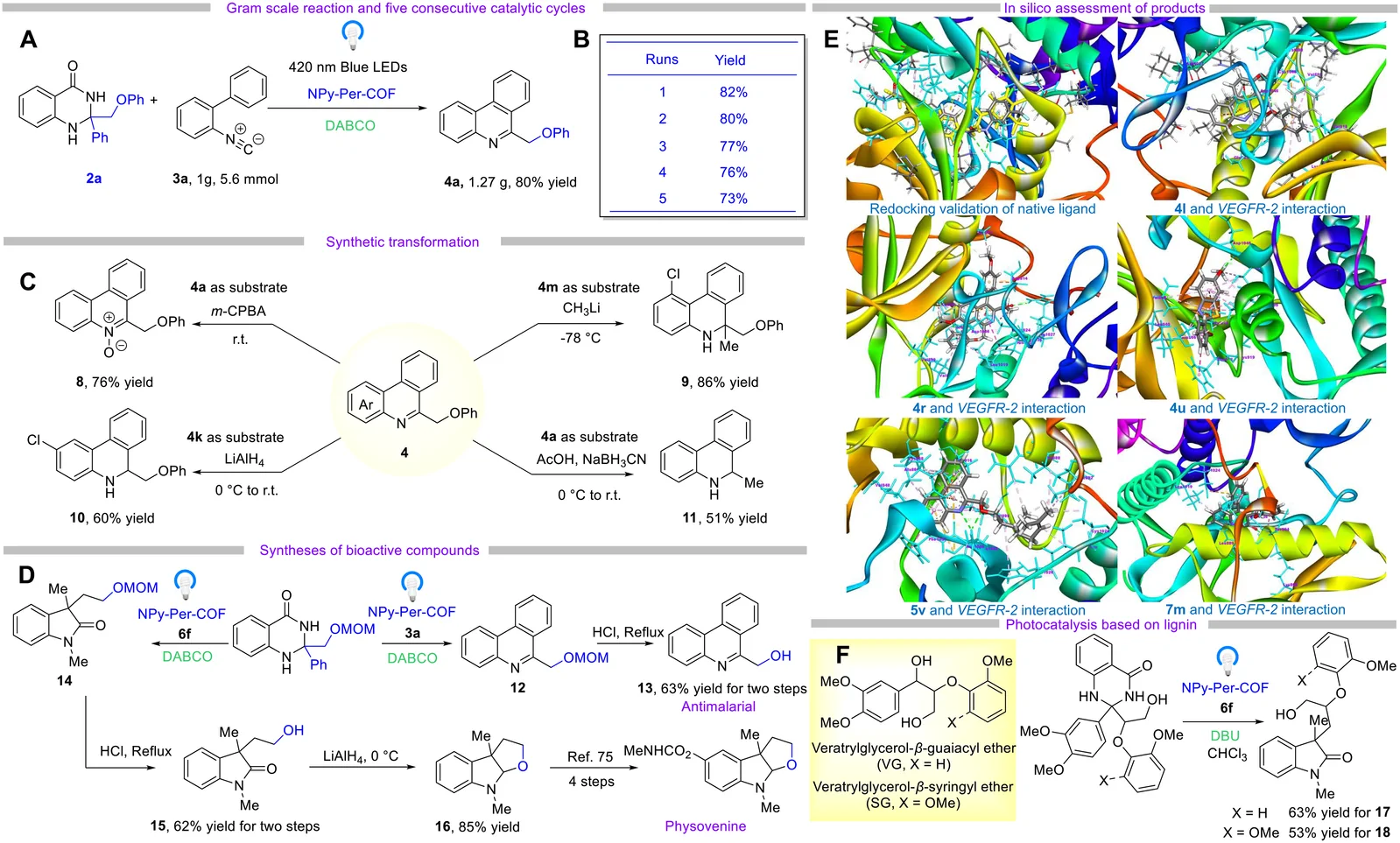
Practicality and transformations of the photocatalytic system. (**A**) Gram scale reaction. (**B**) Yields of **4a** with recovered NPy-Per-COF in five consecutive runs. (**C**) Representative synthetic transformations of the photocatalytic products. (**D**) Derivatization toward structurally complex and biologically relevant scaffolds. (**E**) Representative in silico poses illustrating potential molecular recognition features. Superimposition of the co-crystallized (sorafenib) ligand pose and the redocked pose within the active site of the target protein. The close alignment (low RMSD) confirms the reliability and reproducibility of the docking protocol used in this study. (**F**) Photocatalytic transformations based on key structural motifs of native lignin.

To demonstrate the practicality of this photocatalytic system, the cascade reaction between **2a** and **3a** was performed on gram scale. The target product **4a** was obtained in 80% isolated yield, demonstrating that the transformation is readily scalable without erosion of efficiency (Fig. 7A). Owing to the heterogeneous nature of the COF, the photocatalyst could be conveniently recovered by centrifugation and reused. NPy-Per-COF retains its catalytic activity over at least five consecutive cycles with negligible loss in performance (Fig. 7B). Post-reaction characterization by PXRD and nitrogen sorption analysis reveals only minor changes in crystallinity and essentially unchanged porosity, confirming the structural robustness of the framework under photocatalytic conditions (figs. S18 and S19 and table S5). To assess the synthetic utility of this methodology, 9-phenanthridine **4a** was subjected to oxidation with *m*-CPBA, yielding 9-phenanthridine oxide **8** in 76% yield. Additionally, 9-dihydrophenanthridine **9** was synthesized *via* a nucleophilic addition of 9-phenanthridine **4m** using CH_3_Li, achieving a yield of 86%. Chemoselective reductions could also be achieved in a controlled manner. In the presence of the strong reductant LiAlH_4_, 9-phenanthridine **4k** was readily reduced to 9-dihydrophenanthridine **10** with a yield of 60%. When NaBH_3_CN was used as the reductant, 9-phenanthridine **4a** was efficiently transformed into 9-phenanthridine **11** (Fig. 7C). The broader synthetic relevance of the photocatalytic platform was further illustrated through the preparation of biologically relevant molecules (Fig. 7D). For example, MOM-protected 9-phenanthridine **12**, obtained via the photocatalytic reaction from **3a**, was successfully hydrolyzed to give 9-phenanthridinyl alcohol **13**, a scaffold with reported antimalarial activity (*82*). Similarly, hydrolysis of the MOM-protected indolinone **14**, derived from substrate **6f**, yielded indolinonyl alcohol **15**. Subsequent reduction of **15** with LiAlH_4_ yielded the aminal **16** in high yield, a known precursor to Physovenine (*83*). Collectively, these studies underscore the scalability, robustness and synthetic utility of the enzyme-inspired reductive quenching cycle.

In order to explore the anticancer potential of the products, molecular docking was initially performed using Molegro Virtual Docker to assess the binding affinities of the synthesized derivatives against *VEGFR-2* (PDB ID: 4ASD), a key anticancer target. The docking protocol was validated by redocking the co-crystallized ligand sorafenib, which reproduced the experimental binding pose with a root-mean-square deviation of 1.232 Å (Fig. 7E), indicating reliable pose prediction (*84*–*86*). Several representative products, including **4l**, **4r**, **4u**, **5v** and **7m**, exhibit favourable docking scores relative to sorafenib (tables S6 and S7). Analysis of the predicted binding modes reveals well-defined orientations within the *VEGFR-2* active site, characterized by hydrogen-bonding interactions with the hinge-region residue Asp1046, together with complementary electrostatic and hydrophobic contacts involving residues such as Lys868, Arg1027, Val848, Ile892, Leu889, Phe918 and Phe1047 (Fig. 7E and fig. S20). These interaction patterns closely resemble those reported for established *VEGFR-2* ligands, suggesting that the heteroaromatic frameworks generated by the present photocatalytic strategy can engage biologically relevant recognition motifs (*87*).

To further rationalize the observed docking trends, DFT calculations were performed to analyse the electronic properties of selected compounds. The computed descriptors (table S8 and fig. S21) indicate that compound **7m** possesses a relatively small HOMO-LUMO gap (3.706 eV) and enhanced electrophilicity, consistent with increased polarizability and charge-transfer capability. Compounds **4l**, **4r** and **5v** display intermediate electronic reactivity, whereas **4u**, with a larger HOMO-LUMO gap (4.98 eV), appears less electronically activated, suggesting that its binding is dominated by steric and hydrophobic complementarity rather than orbital-driven interactions (*88*). Molecular electrostatic potential (MEP) maps further support this interpretation by highlighting complementary donor–acceptor regions that align with key interactions in the predicted *VEGFR-2* binding pocket (fig. S22). In addition, calculated physicochemical and pharmacokinetic parameters indicate that several representative products exhibited acceptable physicochemical properties, with no apparent pan-assay interference (PAINS) alerts and moderate synthetic accessibility (tables S9 and S10 and fig. S23) (*85*, *89*–*92*). Although these assessments are based on in silico predictions, they collectively suggest that the photocatalytic system enables rapid access to structurally diverse heteroaromatic scaffolds that combine synthetic accessibility with potential biological recognition features.

Finally, to further demonstrate the relevance of the photocatalytic system to lignin valorization, lignin-derived substrates were examined. Dihydroquinazolinones derived from veratrylglycerol-β-guaiacol (VG) and veratrylglycerol-β-syringyl ether (SG), which incorporate key structural motifs of native lignin (*93*, *94*), react efficiently with substrate **6f** to afford the corresponding indolinones in moderate to high yields (Fig. 7F). These results highlight the potential of the enzyme-inspired reductive quenching cycle strategy to convert lignin-derived fragments into value-added heteroaromatic architectures with prospective biological relevance.

### Mechanistic studies

**Fig. 8.**
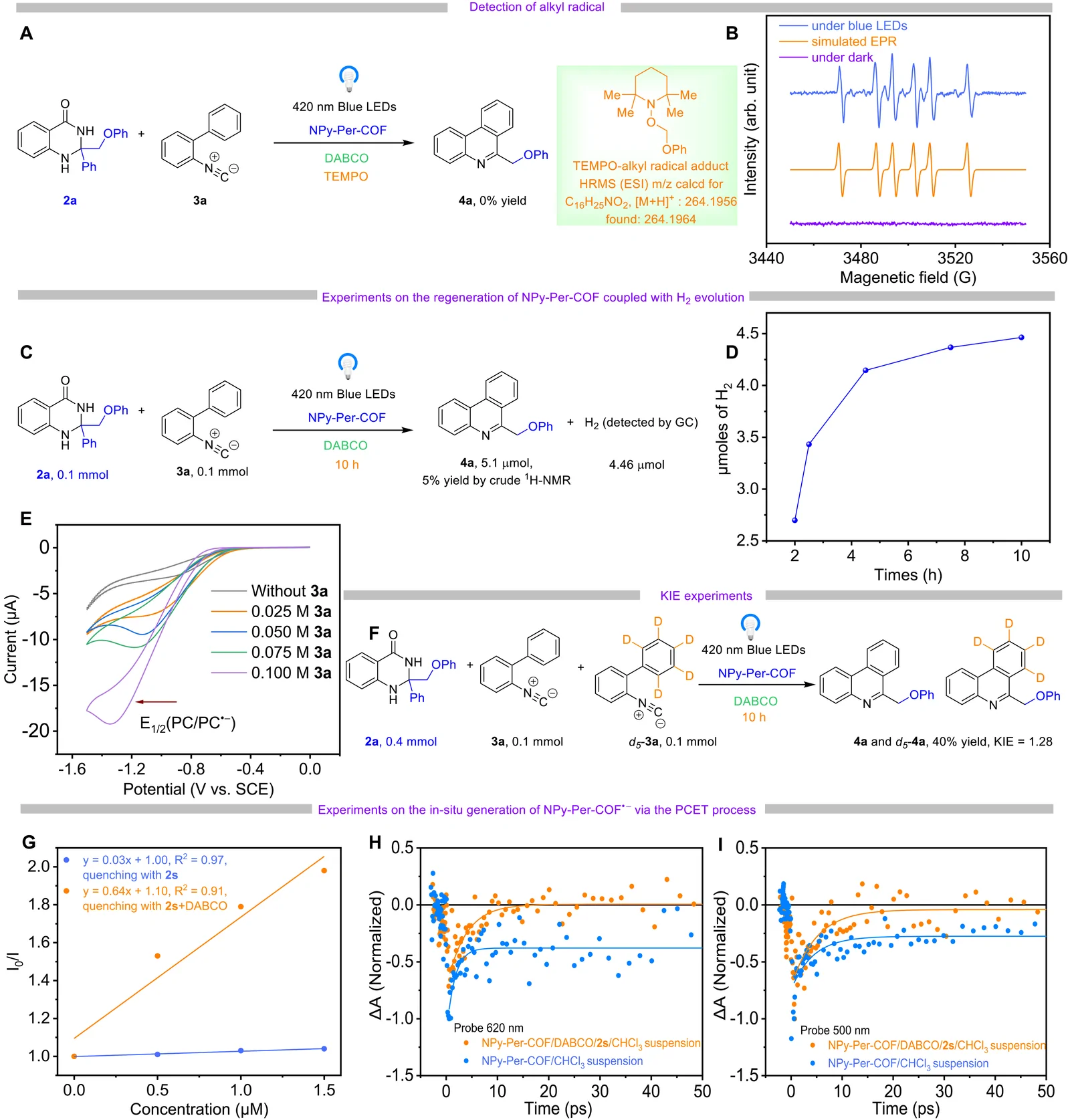
Mechanistic studies. (**A**) Radical prevention experiment. (**B**) EPR analysis. (**C** and **D**) Isolation of intermediate **4a** and quantitative analysis of H_2_. (**E**) CV of NPy-Per-COF in CHCl_3_ solution containing 0.5 M DABCO with the addition of **2a**. (**F**) KIE experiments. (**G**) Stern-Volmer quenching studies. (**H**) Kinetic traces of NPy-Per-COF and NPy-Per-COF with **2s** and DABCO at 620 nm. (**I**) Kinetic traces of NPy-Per-COF and NPy-Per-COF with **2s** and DABCO at 500 nm.

To elucidate the mechanism of the photocatalytic cascade reaction, a series of control and spectroscopic experiments were conducted (Fig. 8). Initially, the addition of 2,2,6,6-tetramethylpiperidin-1-yl)oxyl (TEMPO) completely suppressed the reaction between **2a** and **3a** (Fig. 8A). High-resolution mass spectrometry detects a TEMPO-alkyl radical adduct, supporting the generation of a C-radical generated under the photocatalytic conditions (fig. S24). Direct evidence for radical intermediates was obtained by electron paramagnetic resonance (EPR) spectroscopy using 5,5-dimethyl-1-pyrroline *N*-oxide (DMPO) as a spin-trapping agent. Upon visible-light irradiation of a mixture containing **2a**, DABCO and NPy-Per-COF, a characteristic EPR signal assignable to the DMPO-alkyl radical adduct was observed (g = 2.00168, A_N_ = 15.2 G, A_H_ = 22.3 G), in good agreement with the simulated spectrum (Fig. 8B and fig. S25). When the reaction was conducted under standard conditions for 10 h, 9-phenanthridine **4a** was formed in 5% yield. Notably, gas chromatography quantitatively detected hydrogen in an approximately equimolar amount relative to **4a** (Fig. 8, C and D), providing direct evidence for H(I) species reduction coupled to the photocatalytic cycle. These observations are consistent with a reductive quenching cycle in which the COF radical anion transfers an electron to a H(I) species, regenerating the ground-state photocatalyst and promoting H_2_ evolution. We performed CV of NPy-Per-COF in DABCO/CHCl_3_ solution to investigate the feasibility of its regeneration coupled with H_2_ evolution. Upon addition of **2a** to the electrolyte, the E_1/2_ shifts negatively, indicating that NPy-Per-COF^•-^ is more prone to oxidation back to NPy-Per-COF. Concurrently, the increase in current density may suggest the reduction of H(I) species to H_2_ (Fig. 8E). Moreover, kinetic isotope effect (KIE) experiments using an equimolar mixture of **3a** and deuterium-labelled *d*_5_-**3a** afford a *k*_H_/*k*_D_ value of 1.28 (Fig. 8F and fig. S26), indicating that C-H bond cleavage is not involved in the rate-determining step. Collectively, these results support a PCET-initiated reductive quenching cycle featuring controlled radical generation and H(I) species reduction with hydrogen evolution.

To further probe the interaction between NPy-Per-COF^*^ and dihydroquinazolinone, fluorescence quenching experiments were performed. Stern-Volmer analysis reveals that the emission of NPy-Per-COF was efficiently quenched by a mixture of substrate **2s** and DABCO, but not by **2s** alone (Fig. 8G and fig. S27), indicating a PCET process involving the base-activated substrate. Upon irradiation of a NPy-Per-COF/CHCl_3_ suspension containing DABCO and **2s** under N_2_ atmosphere and 420 nm LED irradiation, an increase in absorption between 400–675 nm and a decrease between 676–800 nm were observed. These spectral changes may be attributed to the formation of NPy-Per-COF^•-^ in PCET process (fig. S28). Finally, ultrafast transient absorption spectroscopy (fs-TA) spectroscopic experiments were conducted for NPy-Per-COF dispersed in CHCl_3_ (fig. S29). The evolving negative signals in the regions of 420–500 nm and 550–750 nm correspond to the ground-state bleach (GSB) arising from intramolecular charge transfer. The TA spectra of NPy-Per-COF in the presence of **2s** and DABCO exhibits a broader and shifted GSB signal. A comparison of the two spectra at early times (0.6 ps) suggests two distinct excited-state conditions for NPy-Per-COF when **2s** and DABCO are present (fig. S29, yellow line). The new bands at 520 nm and 658 nm in fig. S29B indicate that the photocatalyst forms a new ionic excited state, resulting from electron-hole exchange and stabilized by the polar solvent. Comparison of the transient kinetic traces under the two conditions (Fig. 8, H and I) shows that NPy-Per-COF alone features a non-decaying offset that does not relax to baseline, attributable to the accumulation of photogenerated charge carriers. This offset is eliminated upon substrate introduction, implying the potential generation of the NPy-Per-COF^•-^.

The photocatalytic cascade mechanism was subsequently explored through systematic DFT calculations at the B3LYP/6-311G(d,p) level using a SMD solvation model for chloroform (*72*, *73*). All ground-state intermediates and transition states along the catalytic cycle were fully optimized and verified by vibrational frequency analyses, confirming true minima (zero imaginary frequencies) and first-order saddle points (one imaginary frequency). The connectivity of each transition state was further established by intrinsic reaction coordinate (IRC) calculations. Refined energetics were obtained from single-point energy calculations at the M06-2X-D3/def2-TZVP level, with thermal corrections applied at 298.15 K and 1 atm to derive Gibbs free energies. Excited-state energetics were evaluated using TD-DFT within the adiabatic approximation. PCET process was analysed within a vibronically nonadiabatic framework, whereas SET process was assessed using Marcus theory (*95*–*98*).

**Fig. 9.**
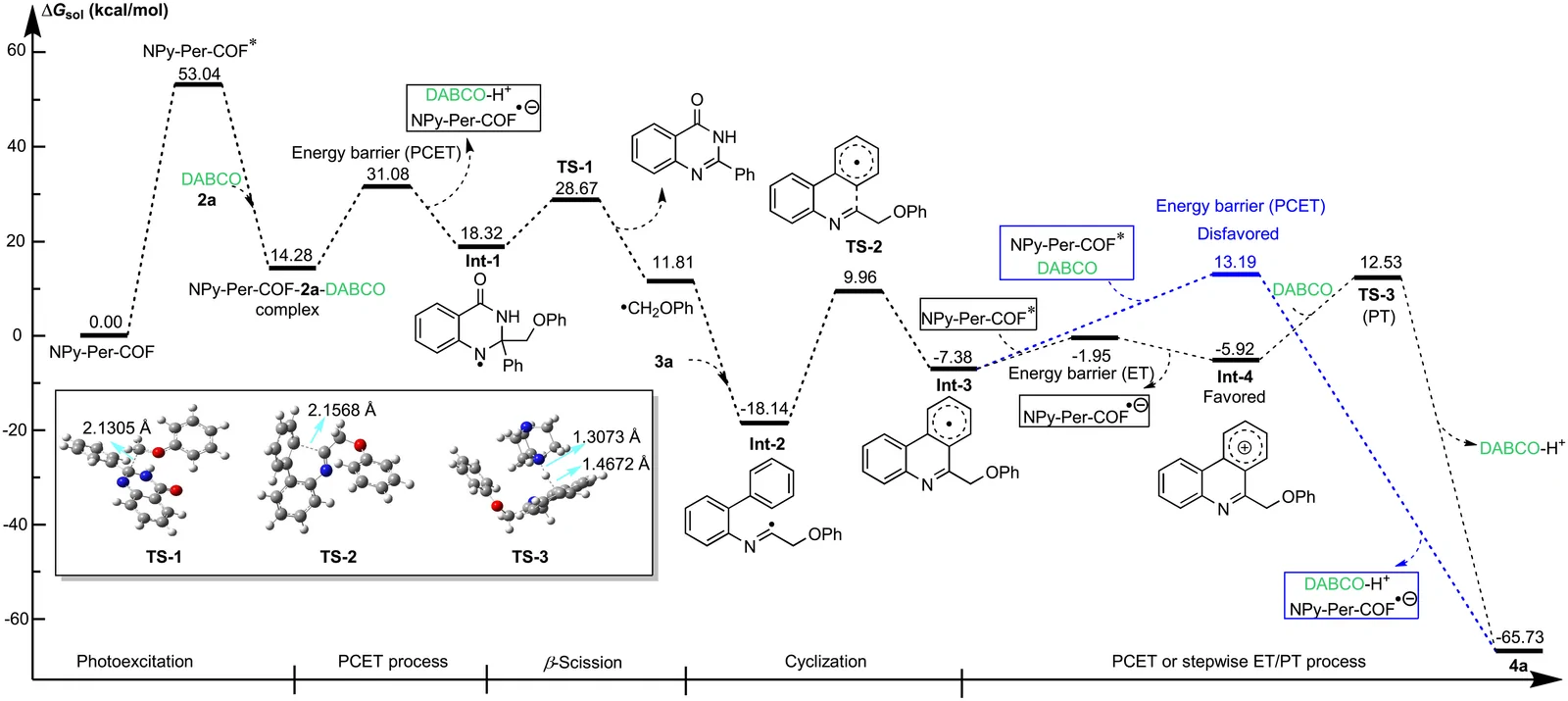
The DFT studies of photocatalytic cascade reaction between 2a and 3a.

As depicted in Fig. 9, the catalytic cycle is initiated upon photoexcitation of NPy-Per-COF, corresponding to an excitation energy of 53.04 kcal/mol. Following the formation of a pre-reactive complex with substrate **2a** and DABCO, C-H bond activation of **2a** proceeds via a nonadiabatic PCET event with an energy barrier of 16.80 kcal/mol, generating an N-radical intermediate **Int-1** together with the COF radical anion and a protonated base DABCO-H^+^. **Int-1** subsequently undergoes β-scission to afford a phenoxymethyl radical and 2-phenylquinazolinone with an energy barrier of 10.35 kcal/mol. Radical addition of the phenoxymethyl species to the unsaturated coupling partner occurs without an appreciable barrier, yielding intermediate **Int-2**. The cyclization of **Int-2** represents the rate-determining step, proceeding through **TS-2** with an activation barrier of 28.10 kcal/mol to afford the cyclic intermediate **Int-3**. Two distinct aromatization pathways from **Int-3** were evaluated. One path involves a concerted PCET to remove a hydrogen atom, which requires a high barrier of 20.57 kcal/mol and is thus disfavored. In contrast, the stepwise ET/PT process is more accessible. First, an ET process from **Int-3** to NPy-Per-COF^*^ generates the cationic intermediate **Int-4** and NPy-Per-COF^•-^. This step is slightly endergonic (ΔG = 1.76 kcal/mol). Marcus theory analysis estimates a low ET activation barrier of 5.43 kcal/mol, with a reaction driving force (ΔG°) of −1.95 kcal/mol, stabilizing **Int-4**. Final elimination is achieved via a PT process from **Int-4** to **4a** through **TS-3**, with an energy barrier of 18.15 kcal/mol relative to **Int-4**.

## DISCUSSION

In summary, we have developed a donor-acceptor covalent organic framework, NPy-Per-COF, with precisely engineered porosity and electronic structure that enables efficient visible-light excitation and controlled charge separation. This framework supports a reductive quenching cycle initiated by PCET process, in which the COF radical anion directly mediates H(I) species reduction to evolve hydrogen, thereby eliminating the need for external oxidants or cocatalysts. Leveraging this enzyme-inspired redox logic, NPy-Per-COF enables a broad range of dehydrogenative radical cascade reactions between lignin-derived dihydroquinazolinones and diverse unsaturated partners, providing streamlined access to structurally diverse heteroaromatic scaffolds. The heterogeneous nature of the COF allows straightforward catalyst recovery and reuse with minimal loss of activity, highlighting its practical advantages over conventional homogeneous photocatalysts. Further synthetic modifications and computational analyses confirm the biological relevance and therapeutic potential of the synthesized scaffolds. Beyond model substrates, the photocatalytic system is also applicable to lignin-derived motifs, demonstrating its applicability in biomass-related chemical space. Combined experimental and computational studies reveal that donor-acceptor polarization within the COF is essential for stabilizing charge-separated states, promoting PCET-driven radical generation and closing the catalytic cycle through H(I) species reduction. Beyond expanding the accessible chemical space of functionalized aromatics, this work establishes mechanistically informed design guidelines for heterogeneous photocatalytic reductive quenching cycle inspired by enzymatic redox control, offering a sustainable strategy for cascade synthesis and value-added transformation of lignin-related feedstocks.

## MATERIALS AND METHODS

### General experimental procedure of photocatalyzed cascade reactions

Under a nitrogen atmosphere, NPy-Per-COF (14.1 mg, 0.01 mmol based on the repeating unit), **2** (0.4 mmol), **3** (0.2 mmol) and DABCO (44.8 mg, 0.4 mmol) were mixed in CHCl_3_ (4.0 mL) in a Schlenk tube. The resulting mixture was stirred under blue LED irradiation (420 nm) at room temperature for 48–96 hours. Upon completion, the residual was filtered through celite pad and filtrate was concentrated. The mixture was purified by silica gel flash chromatography (petroleum ether: ethyl acetate, 10: 1) to afford the desired product **4** or **5**. The desired products **7** were synthesized through the reactions of **2a** with the corresponding substrates using this method. The structure of isoquinoline-1,3-dione **7 h** was unambiguously confirmed by single-crystal X-ray diffraction (fig. S30 and tables S11 to S16).

We only use ChatGPT and Grammarly for refinement, correction, editing, or formatting to improve the clarity of language.

## References

[1] M. Fagnoni, D. Dondi, D. Ravelli, A. Albini, Photocatalysis for the formation of the C-C bond. Chem. Rev. 107, 2725–2756 (2007). 10.1021/cr068352x 17530909

[2] K. L. Skubi, T. R. Blum, T. P. Yoon, Dual catalysis strategies in photochemical synthesis. Chem. Rev. 116, 10035–10074 (2016). 10.1021/acs.chemrev.6b00018 PMC5083252 27109441

[3] N. A. Romero, D. A. Nicewicz, Organic photoredox catalysis. Chem. Rev. 116, 10075–10166 (2016). 10.1021/acs.chemrev.6b00057 27285582

[4] X.-Y. Yu, J.-R. Chen, W.-J. Xiao, Visible light-driven radical-mediated C-C bond cleavage/functionalization in organic synthesis. Chem. Rev. 121, 506–561 (2021). 10.1021/acs.chemrev.0c00030 32469528

[5] L. Marzo, S. K. Pagire, O. Reiser, B. König, Visible-light photocatalysis: does it make a difference in organic synthesis? Angew. Chem. Int. Ed. Engl. 57, 10034–10072 (2018). 10.1002/anie.201709766 29457971

[6] W. Zhou, I. A. Dmitriev, P. Melchiorre, Reductive cross-coupling of olefins via a radical pathway. J. Am. Chem. Soc. 145, 25098–25102 (2023). 10.1021/jacs.3c11285 PMC10682986 37947488

[7] R. Giri, E. Zhilin, M. Kissling, S. Patra, A. J. Fernandes, D. Katayev, Visible-light-mediated vicinal dihalogenation of unsaturated C-C bonds using dual-functional group transfer reagents. J. Am. Chem. Soc. 146, 31547–31559 (2024). 10.1021/jacs.4c09039 PMC11583368 39498866

[8] S. Shibutani, K. Nagao, H. Ohmiya, A dual cobalt and photoredox catalysis for hydrohalogenation of alkenes. J. Am. Chem. Soc. 146, 4375–4379 (2024). 10.1021/jacs.3c10133 38300804

[9] S. Xu, H. Zhang, J. Xu, W. Suo, C.-S. Lu, D. Tu, X. Guo, J. Poater, M. Solà, H. Yan, Photoinduced selective B-H activation of *nido*-carboranes. J. Am. Chem. Soc. 146, 7791–7802 (2024). 10.1021/jacs.4c00550 38461434

[10] P. Wang, L. Lin, Y. Huang, H. Zhang, S. Liao, Radical fluorosulfonamidation: a facile access to sulfamoyl fluorides. Angew. Chem. Int. Ed. Engl. 63, e202405944 (2024). 10.1002/anie.202405944 38837324

[11] F. Zhang, S. Dutta, A. Petti, D. Rana, C. G. Daniliuc, F. Glorius, Solvent–dependent divergent cyclization of bicyclo[1.1.0]butanes. Angew. Chem. Int. Ed. Engl. 64, e202418239 (2024). 10.1002/anie.202418239 39688002

[12] H. Miao, L. Zhu, D. Zhang, Q. Meng, T. Yin, G. Zhang, T. Xiong, Q. Zhang, A photo- and cobalt-catalyzed enantioselective hydroamination of unactivated alkenes with amines. Angew. Chem. Int. Ed. Engl. 64, e13891 (2025). 10.1002/anie.202513891 41186024

[13] K. Pal, R. Kancherla, S. Dutta, S. Zhumagazy, B. Maity, L. Cavallo, M. Rueping, Bypassing β-hydride elimination: excited-state Pd-catalyzed C(sp^3^)-C(sp^2^) coupling at room temperature. Angew. Chem. Int. Ed. Engl. 64, e22979 (2025). 10.1002/anie.202522979 41208650

[14] Y. Liu, X.-L. Chen, X.-Y. Li, S.-S. Zhu, S.-J. Li, Y. Song, L.-B. Qu, B. Yu, 4CzIPN–*^t^*Bu–catalyzed proton-coupled electron transfer for photosynthesis of phosphorylated *N*-heteroaromatics. J. Am. Chem. Soc. 143, 964–972 (2021). 10.1021/jacs.0c11138 33373207

[15] F.-L. Zeng, Z.-Y. Zhang, P.-C. Yin, F.-K. Cheng, X.-L. Chen, L.-B. Qu, Z.-Y. Cao, B. Yu, Visible-light-induced cascade cyclization of 3-(2-(ethynyl)phenyl)quinazolinones to phosphorylated quinolino[2,1-*b*]quinazolinones. Org. Lett. 24, 7912–7917 (2022). 10.1021/acs.orglett.2c02930 36269864

[16] K.-C. Yu, H. Li, Y.-H. Tu, H. Zhao, X.-G. Hu, Metallaphotoredox-enabled construction of the P(O)–N bond from aromatic amines and P(O)–H compounds. Org. Lett. 24, 9130–9134 (2022). 10.1021/acs.orglett.2c03860 36472909

[17] T. Yu, H. Ni, S. Fan, J. Jiang, S. You, C. Deng, Highly regioselective modular assembly of 3-phosphonyl polysubstituted pyridines through radical cascade cyclization of 1,5-enynes with phosphine oxide by photoinitiation. Org. Lett. 26, 10729–10734 (2024). 10.1021/acs.orglett.4c03559 39641439

[18] H. Zhou, C. Wu, Y. Han, B. Huang, C. Wang, S. Mei, J. Yang, Photocatalyzed aerobic cross-dehydrogenative coupling of diarylphosphine oxides with alcohols and phenols. Org. Lett. 26, 2435–2439 (2024). 10.1021/acs.orglett.4c00580 38501966

[19] S. K. Banjare, L. Lezius, E. S. Horst, D. Leifert, C. G. Daniliuc, F. A. Alasmary, A. Studer, Thermal and photoinduced radical cascade annulation using aryl isonitriles: an approach to quinoline-derived benzophosphole oxides. Angew. Chem. Int. Ed. Engl. 63, e20240427 (2024). 10.1002/anie.202404275 38687058

[20] W.-Q. Liu, T. Lei, S. Zhou, X.-L. Yang, J. Li, B. Chen, J. Sivaguru, C.-H. Tung, L.-Z. Wu, Cobaloxime catalysis: selective synthesis of alkenylphosphine oxides under visible light. J. Am. Chem. Soc. 141, 13941–13947 (2019). 10.1021/jacs.9b06920 31401832

[21] W.-L. Yu, Y.-C. Luo, L. Yan, D. Liu, Z.-Y. Wang, P.-F. Xu, Dehydrogenative silylation of alkenes for the synthesis of substituted allylsilanes by photoredox, hydrogen-atom transfer, and cobalt catalysis. Angew. Chem. Int. Ed. Engl. 58, 10941–10945 (2019). 10.1002/anie.201904707 31166076

[22] X.-Y. Zhang, X.-Y. Wu, B. Zhang, Y. Wei, M. Shi, Silyl radical-mediated carbocyclization of acrylamide−/vinyl sulfonamide-attached alkylidenecyclopropanes via photoredox catalysis with a catalytic amount of silane reagent. ACS Catal. 11, 4372–4380 (2021).

[23] J. Li, C.-Y. Huang, J.-T. Han, C.-J. Li, Development of a quinolinium/cobaloxime dual photocatalytic system for oxidative C-C cross-couplings via H_2_ release. ACS Catal. 11, 14148–14158 (2021).

[24] C.-Y. Huang, J. Li, C.-J. Li, A cross-dehydrogenative C(sp^3^)-H heteroarylation via photoinduced catalytic chlorine radical generation. Nat. Commun. 12, 4010 (2021). 10.1038/s41467-021-24280-9 PMC8241867 34188034

[25] J.-X. Yu, Y.-Y. Cheng, B. Chen, C.-H. Tung, L.-Z. Wu, Cobaloxime photocatalysis for the synthesis of phosphorylated heteroaromatics. Angew. Chem. Int. Ed. Engl. 61, e202209293 (2022). 10.1002/anie.202209293 35912895

[26] J.-D. Guo, Y.-J. Chen, C.-H. Wang, Q. He, X.-L. Yang, T.-Y. Ding, K. Zhang, R.-N. Ci, B. Chen, C.-H. Tung, L.-Z. Wu, Direct excitation of aldehyde to activate the C(sp^2^)-H bond by cobaloxime catalysis toward fluorenones synthesis with hydrogen evolution. Angew. Chem. Int. Ed. Engl. 62, e202214944 (2023). 10.1002/anie.202214944 36510781

[27] H.-W. Jiang, W.-L. Yu, D. Wang, P.-F. Xu, Photocatalyzed H_2_-acceptorless dehydrogenative borylation by using amine borane. ACS Catal. 14, 8666–8675 (2024).

[28] C.-H. Wang, J.-D. Guo, J.-X. Yu, J. Qiao, B. Chen, C.-H. Tung, L.-Z. Wu, Photocatalytic cross-coupling of aldehydes and alkenes for aryl vinyl ketones by a single catalyst. Org. Lett. 26, 6927–6932 (2024). 10.1021/acs.orglett.4c02570 39106055

[29] J.-X. Yu, Y.-Y. Cheng, X.-Y. Zeng, B. Chen, C.-H. Tung, L.-Z. Wu, 1,3-Difunctionalization of alkenes by cobaloxime photocatalysis. Org. Lett. 26, 6809–6813 (2024). 10.1021/acs.orglett.4c02027 39102516

[30] H. Huang, X. Luan, Z. Zuo, Cooperative photoredox and cobalt-catalyzed acceptorless dehydrogenative functionalization of cyclopropylamides towards allylic N,O-acyl-acetal derivatives. Angew. Chem. Int. Ed. Engl. 63, e202401579 (2024). 10.1002/anie.202401579 38609328

[31] C. Wang, Z. Chen, J. Sun, L. Tong, W. Wang, S. Song, J. Li, Sulfonamide-directed site-selective functionalization of unactivated C(sp^3^)-H bonds enabled by photocatalytic sequential electron/proton transfer. Nat. Commun. 15, 5087 (2024). 10.1038/s41467-024-49337-3 PMC11178871 38876986

[32] J. Talvitie, A. Mollar-Cuni, J. Nyman, J. Koivula, L. Hendrickx, P. Muñoz Rodríguez, J. Helaja, Photoredox catalytic synthesis of indoles via direct N-H activation to generate putative aminyl radicals. Org. Lett. 27, 10537–10541 (2025). 10.1021/acs.orglett.5c03410 PMC12455651 40920969

[33] C. Huang, S. Tang, Y. Wang, C.-L. Wang, M.-Z. Du, J. Qiao, Z. Wei, H. Cai, Solvent-dependent photocatalytic acceptorless dehydrogenation/dehydrogenation-coupling-aromatization of *N*-heterocycles. Org. Lett. 27, 8370–8375 (2025). 10.1021/acs.orglett.5c02735 40695749

[34] B. Sun, S. Zhou, J. Wang, X. Xu, X. Zhuang, W. Su, C. Jin, Hydrogen-bonding-driven site-selective activation of *α*-C(sp^3^)-H in alcohols: a straightforward dual-catalysis strategy for efficient acceptorless dehydrogenation. Angew. Chem. Int. Ed. Engl. 64, e202506022 (2025). 10.1002/anie.202506022 40353802

[35] A. E. B. S. Stone, A. Fortunato, X. Wang, E. Saggioro, R. Q. Snurr, J. T. Hupp, F. Arcudi, L. Ðorđević, Photocatalytic semi-hydrogenation of acetylene to polymer-grade ethylene with molecular and metal-organic framework cobaloximes. Adv. Mater. 37, e2408658 (2025). 10.1002/adma.202408658 PMC11707558 39439160

[36] P. Ander, K.-E. Eriksson, Selective degradation of wood components by white-rot fungi. Physiol. Plant. 41, 239–248 (1977).

[37] P. J. Harvey, H. E. Schoemaker, R. M. Bowen, J. M. Palmer, Single-electron transfer processes and the reaction mechanism of enzymic degradation of lignin. FEBS Lett. 183, 13–16 (1985).

[38] A. Paterson, K. Lundquist, Polymer biochemistry: Radical breakdown of lignin. Nature 316, 575–576 (1985).

[39] C. Zhang, X. Shen, Y. Jin, J. Cheng, C. Cai, F. Wang, Catalytic strategies and mechanism analysis orbiting the center of critical intermediates in lignin depolymerization. Chem. Rev. 123, 4510–4601 (2023). 10.1021/acs.chemrev.2c00664 37022360

[40] L. J. Karas, C.-H. Wu, J. I. Wu, Barrier-lowering effects of baird antiaromaticity in photoinduced proton-coupled electron transfer (PCET) reactions. J. Am. Chem. Soc. 143, 17970–17974 (2021). 10.1021/jacs.1c09324 PMC8993029 34672631

[41] M. C. Kessinger, J. Xu, K. Cui, Q. Loague, A. V. Soudackov, S. Hammes-Schiffer, G. J. Meyer, Direct evidence for a sequential electron transfer-proton transfer mechanism in the PCET reduction of a metal hydroxide catalyst. J. Am. Chem. Soc. 146, 1742–1747 (2024). 10.1021/jacs.3c10742 38193695

[42] D. A. Granados, Y. E. Du, S. J. Andersson, A. Cirincione-Lynch, K. Cui, A. Reinhold, P. D. Jeffrey, G. D. Scholes, S. Hammes-Schiffer, R. R. Knowles, Iridium polypyridyl carboxylates as excited-state PCET catalysts for the functionalization of unactivated C-H bonds. J. Am. Chem. Soc. 147, 20703–20715 (2025). 10.1021/jacs.5c04000 PMC12237436 40492823

[43] T. Inoue, D. Tomiya, M. Fuki, Y. Kobori, M. Higashi, K. Uesaka, A. Yamakata, S. A. Kawashima, K. Yamatsugu, H. Mitsunuma, M. Kanai, Azaanthraquinone PCET catalysis enables chemoselective decarboxylative functionalization of diverse carboxylic acids. J. Am. Chem. Soc. 147, 40272–40281 (2025). 10.1021/jacs.5c10807 41129730

[44] X.-Y. Lv, R. Abrams, R. Martin, Dihydroquinazolinones as adaptative C(sp^3^) handles in arylations and alkylations via dual catalytic C-C bond-functionalization. Nat. Commun. 13, 2394 (2022). 10.1038/s41467-022-29984-0 PMC9064991 35504911

[45] F. Cong, R. S. Mega, J. Chen, C. S. Day, R. Martin, Trifluoromethylation of carbonyl and unactivated olefin derivatives by C(sp^3^)-C bond cleavage. Angew. Chem. Int. Ed. Engl. 62, e202214633 (2023). 10.1002/anie.202214633 36416716

[46] H. Wu, S. Chen, D. Xiao, F. Li, K. Zhou, X. Yin, C. Liu, X. He, Y. Shang, Visible-light-mediated deacylated alkynylation of unstrained ketones. Org. Lett. 25, 1166–1171 (2023). 10.1021/acs.orglett.3c00145 36786500

[47] Q.-Z. Li, M.-H. He, R. Zeng, Y.-Y. Lei, Z.-Y. Yu, M. Jiang, X. Zhang, J.-L. Li, Molecular editing of ketones through *N*-heterocyclic carbene and photo dual catalysis. J. Am. Chem. Soc. 146, 22829–22839 (2024). 10.1021/jacs.4c08163 39086019

[48] K.-H. He, N. Jin, J.-C. Chen, Y.-F. Zheng, F. Pan, Ketone skeletal modification via a metallaphotoredox-catalyzed deacylation and acylation strategy. Org. Lett. 26, 9503–9507 (2024). 10.1021/acs.orglett.4c03456 39465911

[49] T. Wang, Z. Zhang, F. Gao, X. Yan, Homologation of ketones: direct transformation of alkyl ketones to aryl ketones via a photoredox-catalyzed deacylation-aroylation sequence. Org. Lett. 26, 6915–6920 (2024). 10.1021/acs.orglett.4c02576 39115264

[50] W.-P. Yang, H.-J. Miao, L. Liu, X.-H. Duan, L.-N. Guo, Visible light-promoted aromatization-driven deconstructive fluorination of spiro carbocycles. Org. Lett. 26, 7442–7446 (2024). 10.1021/acs.orglett.4c02793 39186378

[51] J. Li, D. Zhang, L. Tan, C.-J. Li, Direct excitation strategy for deacylative couplings of ketones. Angew. Chem. Int. Ed. Engl. 63, e202410363 (2024). 10.1002/anie.202410363 39043558

[52] H. Wu, S. Chen, C. Liu, Q. Zhao, Z. Wang, Q. Jin, S. Sun, J. Guo, X. He, P. J. Walsh, Y. Shang, Construction of C-S and C-Se bonds from unstrained ketone precursors under photoredox catalysis. Angew. Chem. Int. Ed. Engl. 63, e202314790 (2024). 10.1002/anie.202314790 38185472

[53] Z. Zhang, G. Dong, Carbonyl-to-sulfur swap enabled by sequential double carbon-carbon bond activation. Science 388, 1436–1440 (2025). 10.1126/science.adx2723 PMC13518082 40504950

[54] C.-L. Chan, Y.-T. Tsao, A. S. Paculba, P.-S. Lin, Z.-N. Tsai, H.-H. Chiu, R. Kunitake, M.-J. Chiu, C.-C. Yeh, C.-C. Chiu, H.-H. Liao, Irradiation of bifunctional masked ketone pro-aromatics unveils autoinductive autocatalysis via electron donor-acceptor complexes. Org. Lett. 27, 9593–9598 (2025). 10.1021/acs.orglett.5c02448 PMC12418491 40854869

[55] X. Lin, J. Li, J. Zheng, X. Cai, L. Wang, R. Sa, C. Si, D. Jiang, Y. Kang, J. Wang, Y. Yamauchi, Z. Yuan, Straightforward construction of functionalized *γ*-lactams via conjugated-engineered covalent organic framework photocatalyzed cascade reactions. Nat. Commun. 16, 10818 (2025). 10.1038/s41467-025-66469-2 PMC12669634 41326399

[56] M. Guo, S. Jayakumar, M. Luo, X. Kong, C. Li, H. Li, J. Chen, Q. Yang, The promotion effect of π-π interactions in Pd NPs catalysed selective hydrogenation. Nat. Commun. 13, 1770 (2022). 10.1038/s41467-022-29299-0 PMC8975908 35365621

[57] L. Gutiérrez, V. Martin-Diaconescu, C. Casadevall, F. Oropeza, V. A. de la Peña O’Shea, J. Meng, M. A. Ortuño, J. Lloret-Fillol, Low oxidation state cobalt center stabilized by a covalent organic framework to promote hydroboration of olefins. ACS Catal. 13, 3044–3054 (2023).

[58] Y. Fang, Y. Liu, H. Huang, J. Sun, J. Hong, F. Zhang, X. Wei, W. Gao, M. Shao, Y. Guo, Q. Tang, Y. Liu, Design and synthesis of broadband absorption covalent organic framework for efficient artificial photocatalytic amine coupling. Nat. Commun. 15, 4856 (2024). 10.1038/s41467-024-49036-z PMC11161580 38849337

[59] Y. Zhu, D. Huang, W. Wang, G. Liu, C. Ding, Y. Xiang, Sequential oxidation/cyclization of readily available imine linkages to access benzoxazole-linked covalent organic frameworks. Angew. Chem. Int. Ed. Engl. 63, e202319909 (2024). 10.1002/anie.202319909 38243685

[60] C. Cheng, Y. Liu, G. Sheng, X. Jiang, X. Kang, C. Jiang, Y. Liu, Y. Zhu, Y. Cui, Construction of benzoxazine-linked one-dimensional covalent organic frameworks using the Mannich reaction. Angew. Chem. Int. Ed. Engl. 63, e202403473 (2024). 10.1002/anie.202403473 38829678

[61] M.-Y. Yang, S.-B. Zhang, M. Zhang, Z.-H. Li, Y.-F. Liu, X. Liao, M. Lu, S.-L. Li, Y.-Q. Lan, Three-motif molecular junction type covalent organic frameworks for efficient photocatalytic aerobic oxidation. J. Am. Chem. Soc. 146, 3396–3404 (2024). 10.1021/jacs.3c12724 38266485

[62] J. Cheng, Y. Wu, W. Zhang, J. Zhang, L. Wang, M. Zhou, F. Fan, X. Wu, H. Xu, Fully conjugated 2D sp^2^ carbon-linked covalent organic frameworks for photocatalytic overall water splitting. Adv. Mater. 36, e2305313 (2024). 10.1002/adma.202305313 37818737

[63] R. Gao, R. Shen, C. Huang, K. Huang, G. Liang, P. Zhang, X. Li, 2D/2D hydrogen-bonded organic frameworks/covalent organic frameworks S-scheme heterojunctions for photocatalytic hydrogen evolution. Angew. Chem. Int. Ed. Engl. 64, e202414229 (2025). 10.1002/anie.202414229 39528399

[64] Y. Shan, X. Xu, X. Jin, X. Ding, S. H. Chen, Review of pyrene- and perylene-based photocatalysts: synthesis, development, and applications. Energy Fuel 39, 18376–18405 (2025).

[65] L. Ascherl, E. W. Evans, J. Gorman, S. Orsborne, D. Bessinger, T. Bein, R. H. Friend, F. Auras, Perylene-based covalent organic frameworks for acid vapor sensing. J. Am. Chem. Soc. 141, 15693–15699 (2019). 10.1021/jacs.9b08079 31550149

[66] X. Xu, H. Chen, N. Huang, Perylene-based 2D covalent organic frameworks for selective adsorption and extraction of C_60_. Macromolecules 57, 9457–9465 (2024).

[67] Y.-J. Chen, J.-Z. Zhang, Z.-X. Wu, Y.-X. Qiao, L. Zheng, F. W. Dagnaw, Q.-X. Tong, J.-X. Jian, Molecular engineering of perylene diimide polymers with a robust built-in electric field for enhanced solar-driven water splitting. Angew. Chem. Int. Ed. Engl. 63, e202318224 (2024). 10.1002/anie.202318224 38095880

[68] Z. Li, J. Jiao, W. Fu, K. Gao, X. Peng, Z. Wang, H. Zhuo, C. Yang, M. Yang, G. Chang, L. Yang, X. Zheng, Y. Yan, F. Chen, M. Zhang, Z. Meng, X. Shang, Integration of perylene diimide into a covalent organic framework for photocatalytic oxidation. Angew. Chem. Int. Ed. Engl. 63, e202412977 (2024). 10.1002/anie.202412977 39079914

[69] D. Cao, C. Gong, Y. Han, C. Zhu, Y. Ma, Q. Xia, Y. Peng, G. Yuan, Engineering donor-acceptor arrangement in perylene diimide-based covalent organic frameworks for enhanced singlet oxygen photocatalysis. Angew. Chem. Int. Ed. Engl. 64, e202516908 (2025). 10.1002/anie.202516908 40905454

[70] C. Zhu, C. Gong, D. Cao, L.-L. Ma, D. Liu, L. Zhang, Y. Li, Y. Peng, G. Yuan, Cobalt-metalated 1D perylene diimide carbon-organic framework for enhanced photocatalytic *α*-C(sp^3^)-H Activation and CO_2_ Reduction. Angew. Chem. Int. Ed. Engl. 64, e202504348 (2025). 10.1002/anie.202504348 40192448

[71] X. Xu, Y. Yue, G. Xin, N. Huang, Rational construction of electrically conductive covalent organic frameworks through encapsulating fullerene via donor-acceptor interaction. Macromol. Rapid Commun. 44, e2200715 (2023). 10.1002/marc.202200715 36333909

[72] M. J. Frisch, G. W. Trucks, H. B. Schlegel, G. E. Scuseria, M. A. Robb, J. R. Cheeseman, G. Scalmani, V. Barone, G. A. Petersson, H. Nakatsuji, X. Li, M. Caricato, A. Marenich, J. Bloino, B. G. Janesko, R. Gomperts, B. Mennucci, H. P. Hratchian, J. V. Ortiz, A. F. Izmaylov, J. L. Sonnenberg, D. Williams-Young, F. Ding, F. Lipparini, F. Egidi, J. Goings, B. Peng, A. Petrone, T. Henderson, D. Ranasinghe, V. G. Zakrzewski, J. Gao, N. Rega, G. Zheng, W. Liang, M. Hada, M. Ehara, K. Toyota, R. Fukuda, J. Hasegawa, M. Ishida, T. Nakajima, Y. Honda, O. Kitao, H. Nakai, T. Vreven, K. Throssell, J. A. Montgomery, Jr., J. E. Peralta, F. Ogliaro, M. Bearpark, J. J. Heyd, E. Brothers, K. N. Kudin, V. N. Staroverov, T. Keith, R. Kobayashi, J. Normand, K. Raghavachari, A. Rendell, J. C. Burant, S. S. Iyengar, J. Tomasi, M. Cossi, J. M. Millam, M. Klene, C. Adamo, R. Cammi, J. W. Ochterski, R. L. Martin, K. Morokuma, O. Farkas, J. B. Foresman, D. J. Fox, Gaussian 16 Rev. C.01, Wallingford, CT, 2016.

[73] M. Wang, P. Li, X. Jia, W. Liu, Y. Shao, W. Hu, J. Zheng, B. R. Brooks, Y. Mei, Efficient strategy for the calculation of solvation free energies in water and chloroform at the quantum mechanical/molecular mechanical level. J. Chem. Inf. Model. 57, 2476–2489 (2017). 10.1021/acs.jcim.7b00001 28933850

[74] S. Abu Alrub, A. I. Ali, R. K. Hussein, S. K. Alghamdi, S. A. Eladly, DFT and TD-DFT investigations for the limitations of lengthening the polyene bridge between N,N-dimethylanilino donor and dicyanovinyl acceptor molecules as a D-π-A dye-sensitized solar cell. Int. J. Mol. Sci. 25, 5586 (2024). 10.3390/ijms25115586 PMC11172313 38891775

[75] R. Sarkar, M.-C. Heitz, G. Royal, M. Boggio-Pasqua, Electronic excited states and UV-Vis absorption spectra of the dihydropyrene/cyclophanediene photochromic couple: A theoretical investigation. J. Phys. Chem. A 124, 1567–1579 (2020). 10.1021/acs.jpca.9b11262 32017559

[76] Z. Mi, T. Zhou, W. Weng, J. Unruangsri, K. Hu, W. Yang, C. Wang, K. A. Zhang, J. Guo, Covalent organic frameworks enabling site isolation of viologen-derived electron-transfer mediators for stable photocatalytic hydrogen evolution. Angew. Chem. Int. Ed. Engl. 60, 9642–9649 (2021). 10.1002/anie.202016618 33484039

[77] T. Le Bahers, C. Adamo, I. Ciofini, A qualitative index of spatial extent in charge-transfer excitations. J. Chem. Theory Comput. 7, 2498–2506 (2011). 10.1021/ct200308m 26606624

[78] Y. Qian, Y. Han, X. Zhang, G. Yang, G. Zhang, H.-L. Jiang, Computation-based regulation of excitonic effects in donor-acceptor covalent organic frameworks for enhanced photocatalysis. Nat. Commun. 14, 3083 (2023). 10.1038/s41467-023-38884-w PMC10227069 37248231

[79] J. P. Jeon, Y. J. Kim, S. H. Joo, H. J. Noh, S. K. Kwak, J. B. Baek, Benzotrithiophene-based covalent organic framework photocatalysts with controlled conjugation of building blocks for charge stabilization. Angew. Chem. Int. Ed. Engl. 62, e202217416 (2023). 10.1002/anie.202217416 36545845

[80] C. K. Prier, D. A. Rankic, D. W. C. MacMillan, Visible light photoredox catalysis with transition metal complexes: applications in organic synthesis. Chem. Rev. 113, 5322–5363 (2013). 10.1021/cr300503r PMC4028850 23509883

[81] J. Luo, J. Zhang, Donor-acceptor fluorophores for visible-light-promoted organic synthesis: photoredox/Ni dual catalytic C(sp^3^)-C(sp^2^) cross-coupling. ACS Catal. 6, 873–877 (2016).

[82] C. W. Muth, B. Bhattacharya, R. L. Mahaffey, H. L. Minigh, Synthesis of 6-(.alpha.-hydroxy-.beta.-N,N-dialkylaminoethyl)phenanthridines as potential antimalarials. J. Med. Chem. 16, 303–305 (1973). 10.1021/jm00261a036 4581951

[83] M. G. Kulkarni, A. P. Dhondge, A. S. Borhade, D. D. Gaikwad, S. W. Chavhan, Y. B. Shaikh, V. B. Nigdale, M. P. Desai, D. R. Birhade, M. P. Shinde, Total synthesis of (±)-Physovenine. Eur. J. Org. Chem. 2009, 3875–3877 (2009).

[84] M. A. Fouad, A. A. Osman, N. M. Abdelhamid, M. W. Rashad, A. Y. Nabawy, A. M. El Kerdawy, Discovery of dual kinase inhibitors targeting VEGFR2 and FAK: Structure-based pharmacophore modeling, virtual screening, and molecular docking studies. BMC Chem. 18, 29 (2024). 10.1186/s13065-024-01130-5 PMC10863211 38347617

[85] Z. Amiri, M. Bayat, D. Gheidari, Synthesis of thiazoloquinolinone derivatives: molecular docking, MD simulation, and pharmacological evaluation as VEGFR-2 inhibitors. BMC Chem. 19, 90 (2025). 10.1186/s13065-025-01459-5 PMC11972512 40188066

[86] J. Dong, X. Hao, Pharmacophore screening, molecular docking, and MD simulations for identification of VEGFR-2 and c-Met potential dual inhibitors. Front. Pharmacol. 16, 1534707 (2025). 10.3389/fphar.2025.1534707 PMC11926154 40124780

[87] D. Gheidari, M. Mehrdad, F. Hoseini, Virtual screening, molecular docking, MD simulation studies, DFT calculations, ADMET, and drug likeness of diaza-adamantane as potential MAPK/ERK inhibitors. Front. Pharmacol. 15, 1360226 (2024). 10.3389/fphar.2024.1360226 PMC11253198 39021828

[88] R. G. Parr, Density functional theory of atoms and molecules. *Horizons of Quantum Chemistry*; Fukui, K., Pullman, B., (Eds.) D. Reidel Publishing Company, Springer, 5–15 (1980).

[89] C. A. Lipinski, F. Lombardo, B. W. Dominy, P. J. Feeney, Experimental and computational approaches to estimate solubility and permeability in drug discovery and development settings. Adv. Drug Deliv. Rev. 64, 4–17 (2012). 10.1016/s0169-409x(00)00129-0 11259830

[90] I. Muegge, Selection criteria for drug-like compounds. Med. Res. Rev. 23, 302–321 (2003). 10.1002/med.10041 12647312

[91] Y. Qian, L. Yu, X.-H. Zhang, Z.-Q. Yuan, P. Zhao, L.-N. Sun, Y.-Q. Wang, Genetic polymorphism on the pharmacokinetics and pharmacodynamics of platelet-derived growth factor receptor (PDGFR) kinase inhibitors. Curr. Drug Metab. 19, 1168–1181 (2018). 10.2174/1389200219666180629112943 29956623

[92] S. Baammi, A. El Allali, R. Daoud, Unleashing nature’s potential: A computational approach to discovering novel VEGFR-2 inhibitors from African natural compounds using virtual screening, ADMET analysis, molecular dynamics, and MMPBSA calculations. Front. Mol. Biosci. 10, 1227643 (2023). 10.3389/fmolb.2023.1227643 PMC10548200 37800126

[93] W. Lan, J. B. de Bueren, J. S. Luterbacher, Highly selective oxidation and depolymerization of α,γ-diol-protected lignin. Angew. Chem. Int. Ed. Engl. 58, 2649–2654 (2019). 10.1002/anie.201811630 30600891

[94] X. Zeng, Y. Xu, Q. Dai, J. Li, Q. Lin, J. Ye, C. Liu, W. Lan, Low-temperature degradation of lignin in aprotic solvent system for preparation of monophenolic platform chemicals. Chem. Eng. J. 476, 146466 (2023).

[95] A. Soudackov, S. Hammes-Schiffer, Derivation of rate expressions for nonadiabatic proton-coupled electron transfer reactions in solution. J. Chem. Phys. 113, 2385–2396 (2000).

[96] A. Soudackov, E. Hatcher, S. Hammes-Schiffer, Quantum and dynamical effects of proton donor-acceptor vibrational motion in nonadiabatic proton-coupled electron transfer reactions. J. Chem. Phys. 122, 014505 (2005). 10.1063/1.1814635 15638672

[97] E. R. Sayfutyarova, Z. K. Goldsmith, S. Hammes-Schiffer, Theoretical study of C-H bond cleavage via concerted proton-coupled electron transfer in fluorenyl-benzoates. J. Am. Chem. Soc. 140, 15641–15645 (2018). 10.1021/jacs.8b10461 PMC6402776 30383371

[98] B. Barrios, D. Minakata, Aqueous-phase single-electron transfer calculations for carbonate radicals using the validated Marcus theory. Environ. Sci. Technol. Lett. 10, 204–209 (2023).

