# Supplementary material for: Lignin-degrading-inspired dehydrogenative photocatalysis via reductive quenching of donor-acceptor covalent organic framework
Source: Sci Adv. 2026 Aug 26;12(35):eaef6109. doi: 10.1126/sciadv.aef6109 (PMC13510726; doi:10.1126/sciadv.aef6109)
Supplement: Supplementary file 1 [file sciadv.aef6109_sm.pdf]

Science Advances
AAAS

Supplementary Materials for

Lignin-degrading-inspired dehydrogenative photocatalysis via reductive quenching of donor-acceptor covalent organic framework

Xiangfeng Lin *et al.*

Corresponding author: Zhanhua Huang, huangzh1975@163.com; Dong Jiang, dongjiang@toki.waseda.jp; Chaoqun Zhang, zhangcq@imau.edu.cn; Zhanhui Yuan, zhanhuiyuan@fafu.edu.cn

Sci. Adv. **12**, eaef6109 (2026)
DOI: 10.1126/sciadv.aef6109

The PDF file includes:

Supplementary Text
Figs. S1 to S31
Tables S1 to S16
Legend for data S1

Other Supplementary Material for this manuscript includes the following:

Data S1

1. General information

Characterization of photocatalytic reactions and products: Reactions were monitored by thin layer chromatography (TLC), and column chromatography purifications were carried out using silica gel. Column chromatography was performed on silica gel (300-400 mesh). ^{1}H, ^{13}C, ^{19}F and ^{31}P nuclear magnetic resonance (NMR) spectra were collected on a Bruker AV 400 MHz NMR spectrometer using residual solvent peaks as an internal standard (^{1}H NMR: $CDCl_3$ at 7.26 ppm, DMSO-d_6 at 2.50 ppm; ^{13}C NMR: $CDCl_3$ at 77.00 ppm, DMSO-d_6 at 30.92 ppm). Data for ^{1}H and ^{13}C NMR were recorded as follows: chemical shift (δ, ppm), multiplicity (s = singlet; d = doublet; t = triplet; q = quartet; m = multiplet; br = broad), coupling constant (Hz), integration. High resolution mass spectra for all the new compounds were done by an LTQ-Orbitrap instrument (ESI) (Thermo Fisher Scientific, USA).

Powder X-ray powder diffraction (PXRD): PXRD patterns were measured on a Rigaku RINT smart lab powder diffraction system, equipped with Cu Kα radiation (λ = 1.54 Å).

Fourier-transform infrared spectrometer (FT-IR): FT-IR data were collected using a Bruker VERTEX 70 spectrophotometer with KBr disks.

Solid-state ^{13}C-cross-polarization/total suppression of spinning sidebands nuclear magnetic resonance spectra: Solid-state ^{13}C-CP/TOSS NMR spectra were performed on a Bruker 500 MHz spectrometer.

Scanning electron microscopy (SEM): SEM was performed on a Thermo Fisher Verios G4 UC microscope operating at an accelerating voltage of 5.0 kV.

High-resolution transmission electron microscopy (HR-TEM): HR-TEM was conducted using a Talos F200X instrument (Thermo Fisher) operated at 200 kV, with samples deposited on copper grids coated with an ultrathin carbon film.

Dynamic light scattering (DLS): DLS was performed on Zeta-potential & Particle size analyzer (Malvern ZS90). COF was dispersed in EtOH (0.05 mg/mL), sonicated for 60 min, and left to stand for 10 min prior to DLS measurements.

Thermal gravimetric analyses (TGA): TGA was performed on a Mettler Toledo's TGA/DSC 3+ Synchronous Thermal Analyzer.

Nitrogen adsorption-desorption analysis: Nitrogen sorption isotherms were performed at 77 K to evaluate the porosity property of as-synthesized COFs using an Anton Paar Quantachrome

surface area and pore size analyzer. Each sample (20 mg) was degassed at 120 °C under the flow of nitrogen for 10 hours prior to measurement. The specific surface area of the samples was estimated using the Brunauer-Emmett-Teller (BET) method. The total pore volume was calculated at P/P_0 of 0.99 using a single-point adsorption value. The pore diameter was determined from the adsorption branch by using the Quenched Solid Density Functional Theory (QSDFT) method.

Ultraviolet-visible diffuse reflectance spectra (UV-vis DRS): UV-vis DRS were recorded on a Shimadzu UV-2600 spectrophotometer using $BaSO_4$ as a reflectance standard.

Photoluminescence (PL) spectra: PL excitation spectra and PL emission spectra were recorded on an FLS1000 spectrometer (Edinburgh) equipped with both continuous xenon (450 W) and pulsed flash lamps.

VB-X-ray photoelectron spectroscopy (VB-XPS): VB-XPS measurements were conducted on a ThermoFisher EscaLab Q XI spectrometer using a monochromatic Al Kα X-ray source.

Materials Studio software: The process of simulating COF structure was performed by the Materials Studio software. The triclinic lattice with P1 symmetry group was set as the initial eclipsed COF structure. After the smallest asymmetric fragment was filled into the blank cell, the Forcite tools package was employed to optimize the cell geometry including energy minimization. The cell optimized from the Universal force fields was subsequently refined using the Pawley refinement method in Reflex tools.

Ultrafast transient absorption spectroscopy (fs-TAS): Sub-picosecond transient absorption spectroscopy was made possible using a pump-probe technique with the fundamental 800 nm light as generated by a Coherent Astrella Ti:sapphire Amplifier (120 fs, 1 kHz) and splitting it into two beams. Sufficient power was dumped for both beams so as to not induce damage to any optics. The first beam was directed through an optical parametric amplifier as supplied by Coherent where the wavelength was tuned to 400 nm for pumping. Subsequently, both beams were guided into a HELIOS Femtosecond Transient Absorption Spectrometer (Ultrafast Systems, LLC) where the 400 nm pump passed through a chopper, halving its repetition rate. A neutral density filter was used to adjust the average power to 20 uW.

2. Experimental procedures

2.1 The syntheses of NPy-Per-COF and NPy-Py-COF.

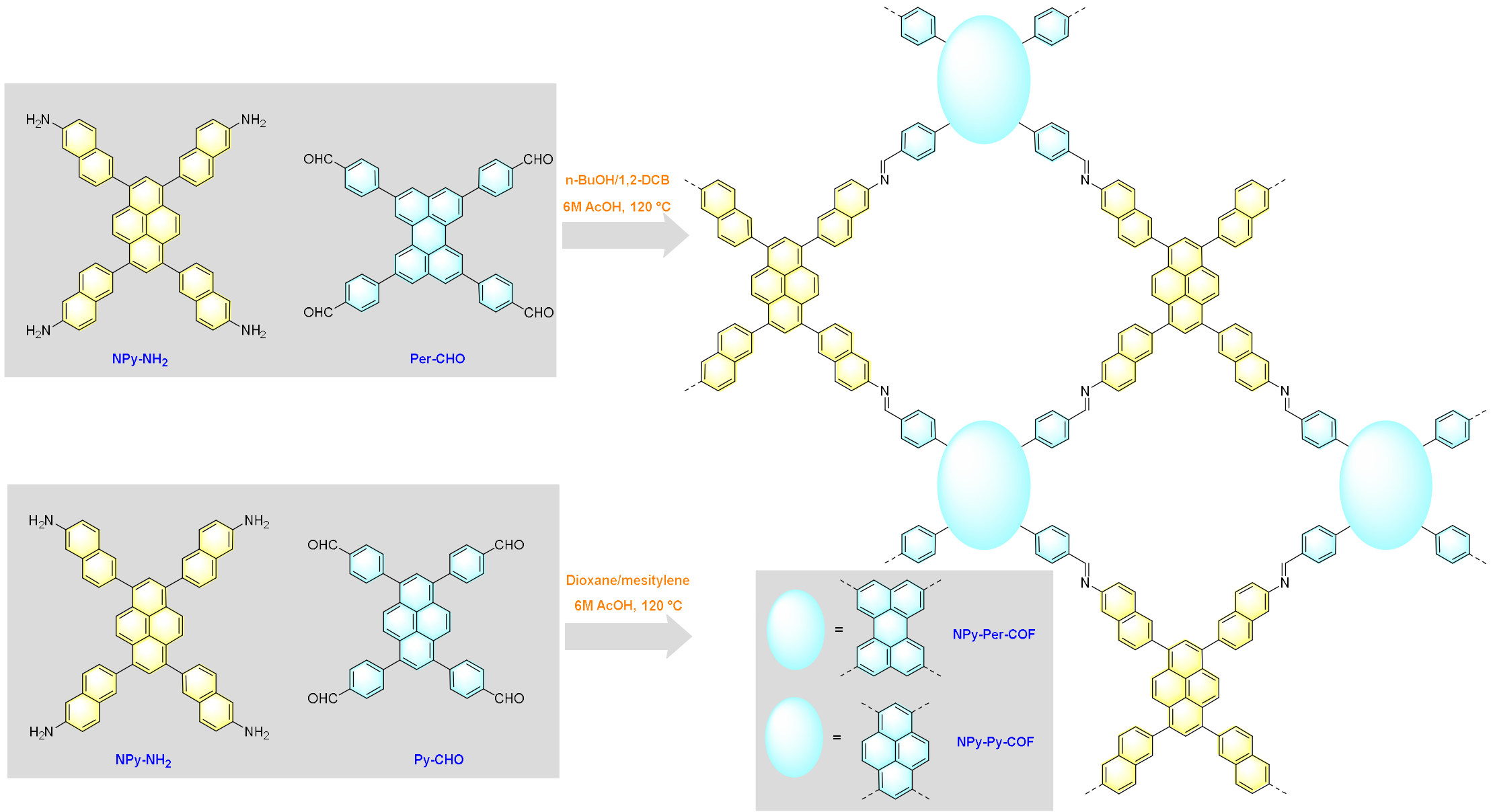

Supplementary Fig. 1. The syntheses of NPy-Per-COF and NPy-Py-COF.

Synthesis of NPy-Per-COF

6,6',6'',6'''-(pyrene-1,3,6,8-tetrayl)tetrakis(naphthalen-2-amine) (NPy-NH_2) (18.4 mg, 0.024 mmol) and 4,4',4'',4'''-(perylene-2,5,8,11-tetrayl)tetrabenzaldehyde (Per-CHO) (16.0 mg, 0.024 mmol) and CH_3COOH (0.1 mL, 6 M) were mixed in a mixture of solvents containing 0.50 mL of *o*-dichlorobenzene and 0.50 mL of 1-butanol in a seal tube. The mixture was ultrasonicated for 10 min and then flash frozen at 77 K (liquid N_2 bath) and degassed through three freeze-pump-thaw cycles. The mixture was then heated at 120 °C for 3 days under an N_2 atmosphere. The resulting solid product was recovered by filtration, washed with methanol and DCM, and subjected to Soxhlet extraction using DCM for 24 hours. After drying under vacuum for 6 hours, an orange solid was obtained with a yield of 86%. This material was designated as NPy-Per-COF.

Synthesis of NPy-Py-COF

6,6',6'',6'''-(pyrene-1,3,6,8-tetrayl)tetrakis(naphthalen-2-amine) (NPy-NH_2) (38.3 mg, 0.05 mmol) and 4,4',4'',4'''-(pyrene-1,3,6,8-tetrayl)tetrabenzaldehyde (Py-CHO) (31.0 mg, 0.05 mmol) and CH_3COOH (0.2 mL, 6 M) were mixed in a mixture of solvents containing 0.50 mL of dioxane

and 1.50 mL of mesitylene in a seal tube. The mixture was ultrasonicated for 10 min and then flash frozen at 77 K (liquid N_2 bath) and degassed through three freeze-pump-thaw cycles. The mixture was then heated at 120 °C for 7 days under an N_2 atmosphere. The resulting solid product was recovered by filtration, washed with methanol and DCM, and subjected to Soxhlet extraction using DCM for 24 hours. After drying under vacuum for 6 hours, a yellow solid was obtained with a yield of 87%. This material was designated as NPy-Py-COF.

2.2 General experimental procedure of photocatalyzed cascade reactions

Syntheses of compounds 4 or 5

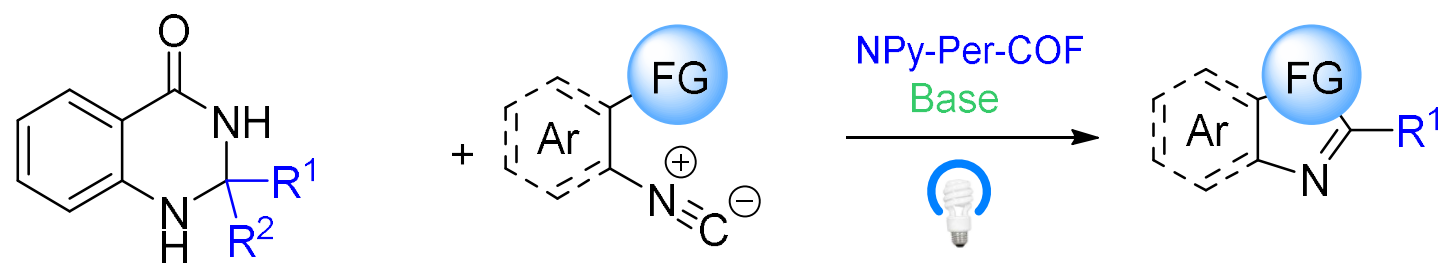

2 **3** **4** or **5**

Under a nitrogen atmosphere, NPy-Per-COF (14.1 mg, 0.01 mmol based on the repeating unit), **2** (0.4 mmol), **3** (0.2 mmol) and DABCO (44.8 mg, 0.4 mmol) were mixed in $CHCl_3$ (4.0 mL) in a Schlenk tube. The resulting mixture was stirred under blue LED irradiation (420 nm) at room temperature for 48-96 hours. Upon completion, the residual was filtered through a Celite pad, and the filtrate was concentrated. The mixture was purified by silica gel flash chromatography (petroleum ether: ethyl acetate, 10 : 1) to afford the desired product **4** or **5**. The synthesis of compounds **5j-k** was conducted on a 0.2 mmol scale using **3a** (35.8 mg, 0.2 mmol) and **2j-k** (0.4 mmol). The reaction was performed in the presence of NPy-Per-COF (14.1 mg, 0.01 mmol based on the repeating unit), DBU (60.8 mg, 0.4 mmol) and LiBr (34.4 mg, 0.4 mmol) in $CHCl_3$ (4.0 mL).

Syntheses of compounds 7

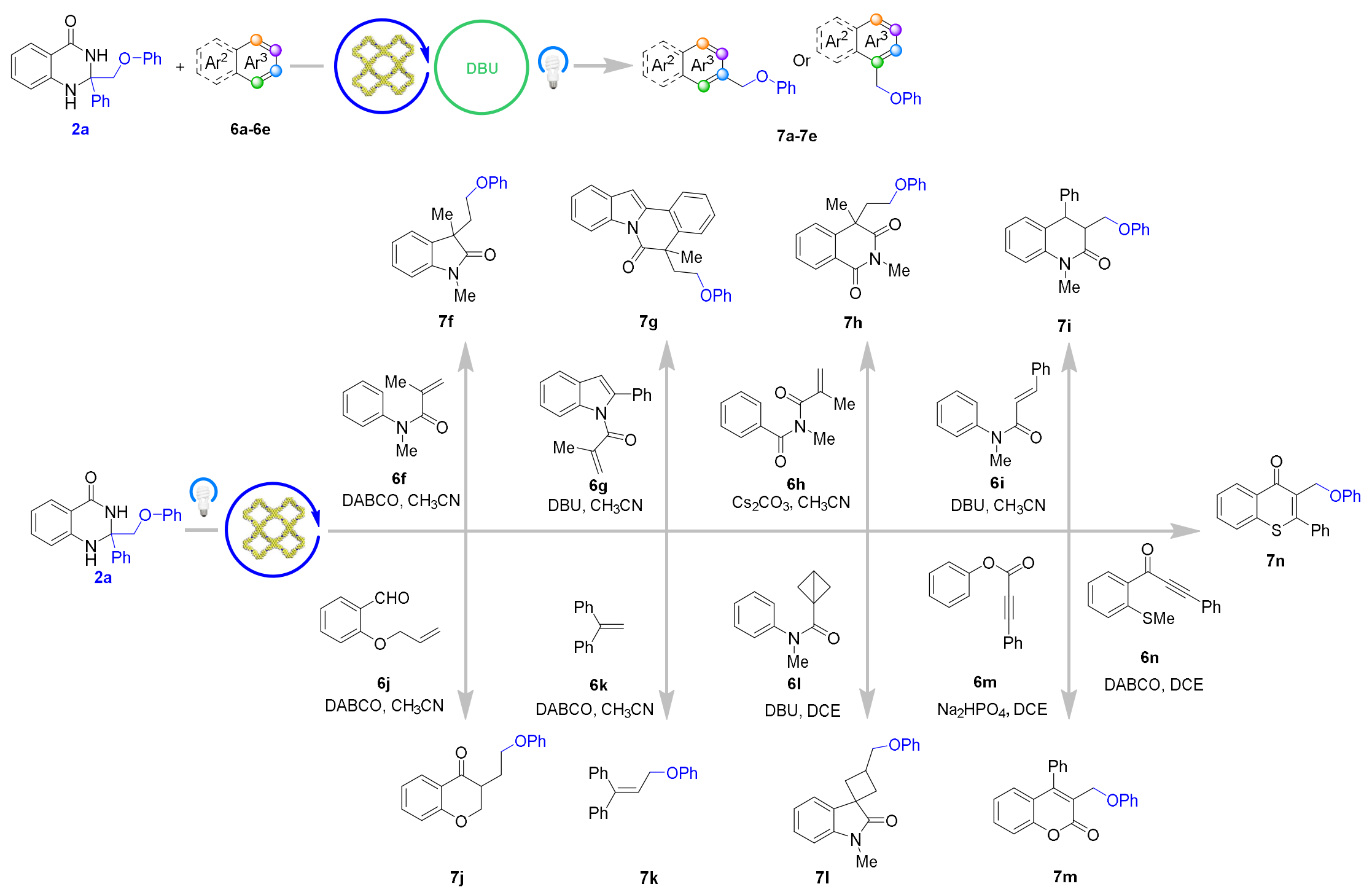

Under a nitrogen atmosphere, NPy-Per-COF (14.1 mg, 0.01 mmol based on the repeating unit), **2a** (132.0 mg, 0.4 mmol), **6** (0.2 mmol) and base (0.4 mmol) were mixed in solvent (4.0 mL) in a Schlenk tube. The resulting mixture was stirred under blue LED irradiation (420 nm) at room temperature for 48-96 hours. Upon completion, the residual was filtered through celite pad and filtrate was concentrated. The mixture was purified by silica gel flash chromatography to afford the desired product **7**.

2.3 Gram scale reaction

Under a nitrogen atmosphere, NPy-Per-COF (790.7 mg, 0.56 mmol based on the repeating unit), **2a** (3.70 g, 11.2 mmol), **3a** (1.0 g, 5.6 mmol) and DABCO (912.2 mg, 8.14 mmol) were mixed in $CHCl_3$ (40.0 mL) in a Schlenk tube. The resulting mixture was stirred at 1200 rpm under blue LED irradiation (420 nm) at room temperature for 120 hours. Upon completion, the residual was filtered through celite pad and filtrate was concentrated. The mixture was purified by silica gel flash chromatography (petroleum ether : ethyl acetate, 10 : 1) to afford the desired product **4a** with a yield of 80%.

2.4 Syntheses of compounds 8 to 18

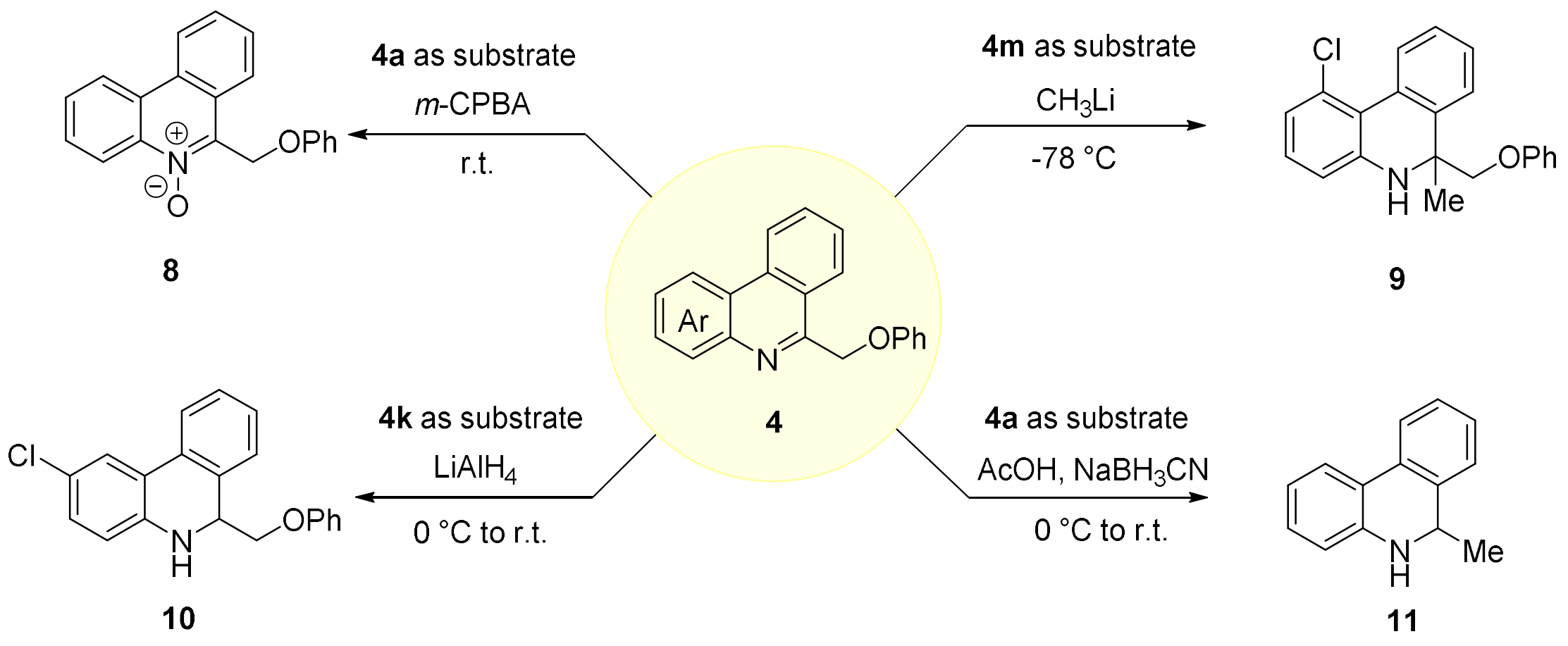

4a (57.0 mg, 0.20 mmol) and *m*-CPBA (103.8 mg, 0.6 mmol) were dissolved in 5.0 mL DCM. The mixture was stirred at room temperature for 12 h. Upon completion, the solvent was removed under reduced pressure, and the resulting mixture was purified by column chromatography on silica gel (petroleum ether : ethyl acetate, 5 : 1) to afford the desired product **8** with a yield of 76%.

Under a nitrogen atmosphere, **4m** (63.8 mg, 0.20 mmol) was dissolved in anhydrous tetrahydrofuran (3.0 mL). Subsequently, CH_3Li in THF at a concentration of 1.0 M (0.6 mL, 3.0 eq.) was added at -78 °C. After 12 hours, the reaction was terminated by the addition of 1.0 mL saturated NaCl solution and extracted with DCM three times. The combined organic layer was dried over Na_2SO_4, filtered, and concentrated under reduced pressure to give a residue. The mixture was purified by silica gel flash chromatography (petroleum ether : ethyl acetate, 20 : 1) to afford the desired product **9** with a yield of 86%.

Under a nitrogen atmosphere, **4k** (63.8 mg, 0.20 mmol) was dissolved in anhydrous tetrahydrofuran (3.0 mL). Subsequently, $LiAlH_4$ in THF at a concentration of 1.0 M (0.6 mL, 3.0 eq.) was added at 0 °C and the mixture was then allowed to warm to room temperature. After 12 hours, the reaction was terminated by the addition of 1.0 mL of a 10% aqueous NaOH solution. The mixture was then desiccated using $MgSO_4$. The residual was filtered through celite pad and filtrate was concentrated. The mixture was purified by silica gel flash chromatography (ethyl acetate : methanol, 10 : 1) to afford the desired product **10** with a yield of 60%.

4a (57.0 mg, 0.20 mmol) was dissolved in AcOH (3.0 mL). Subsequently, $NaBH_3CN$ (31.5 mg, 0.50 mmol) was added at 0 °C and the mixture was then allowed to warm to room

temperature. After 12 hours, the reaction was terminated by the addition of 5.0 mL of a 10% aqueous NaOH solution and extracted with DCM three times. The combined organic layer was dried over Na_2SO_4, filtered, and concentrated under reduced pressure to give a residue. The mixture was purified by silica gel flash chromatography (petroleum ether : ethyl acetate, 20 : 1) to afford the desired product **11** with a yield of 51%.

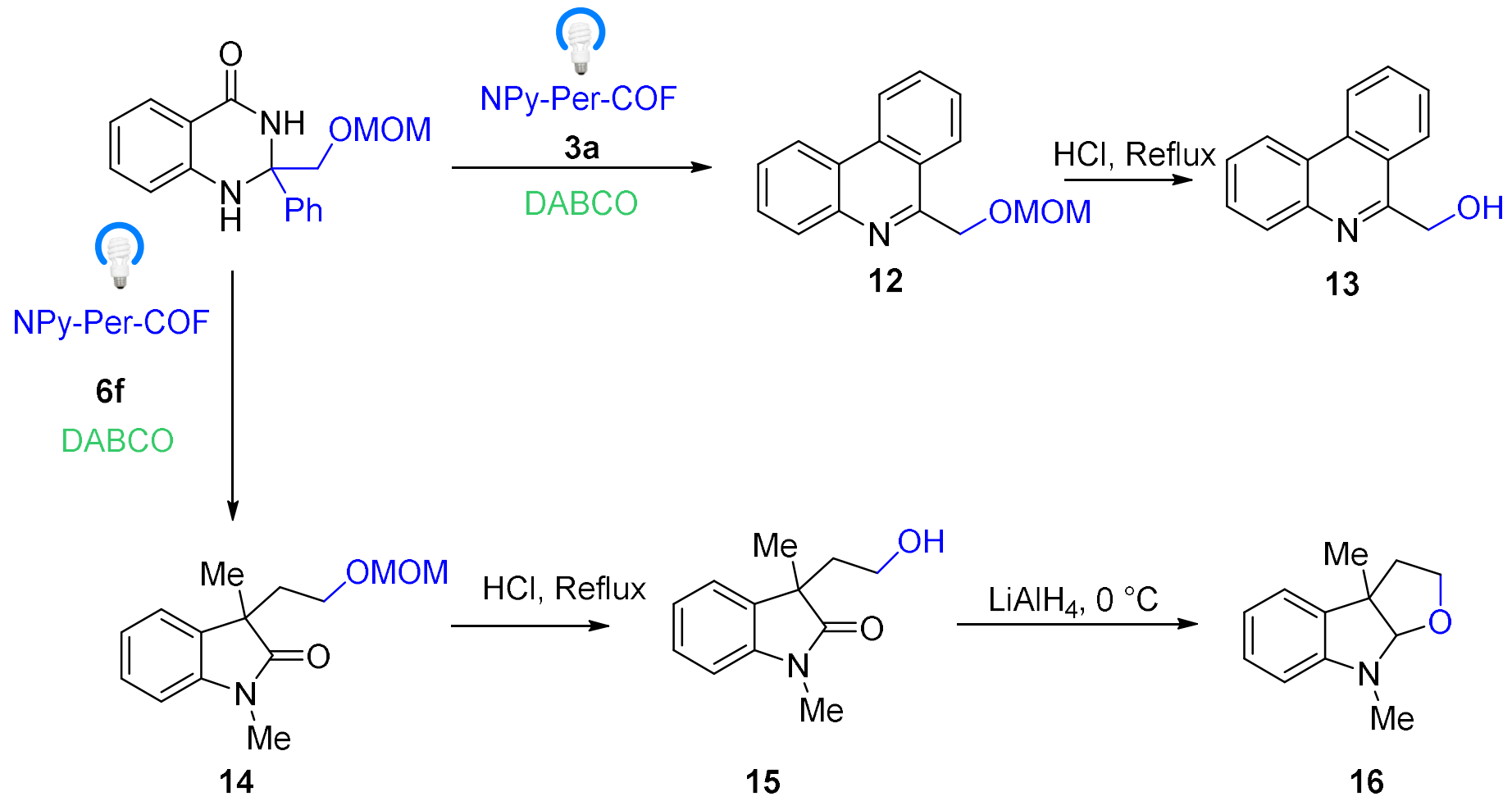

12 was synthesized by the general experimental procedure of photocatalyzed cascade reactions. It exhibits a polarity identical to that of the by-product 2-phenylquinazolin-4(3H)-one, making them difficult to separate via column chromatography.

The mixture of **12** and 2-phenylquinazolin-4(3H)-one was dissolved in 5.0 mL THF, followed by the addition of 0.5 mL conc. HCl. The mixture was stirred at reflux for 12 h. Upon completion, the solvent was removed under reduced pressure, and the resulting mixture was purified by column chromatography on silica gel (petroleum ether : ethyl acetate, 5 : 1) to afford the desired product **13** with a total yield of 63%.

14 was synthesized by the general experimental procedure of photocatalyzed cascade reactions. It exhibits a polarity identical to that of the by-product 2-phenylquinazolin-4(3H)-one, making them difficult to separate via column chromatography.

The mixture of **14** and 2-phenylquinazolin-4(3H)-one was dissolved in 5.0 mL THF, followed by the addition of 0.5 mL conc. HCl. The mixture was stirred at reflux for 12 h. Upon completion, the solvent was removed under reduced pressure, and the resulting mixture was purified by column chromatography on silica gel (petroleum ether : ethyl acetate, 2 : 1) to afford the desired product **15** with a total yield of 62%.

Under a nitrogen atmosphere, **15** (38.1 mg, 0.19 mmol) was dissolved in anhydrous

tetrahydrofuran (3.0 mL). Subsequently, $LiAlH_4$ in THF at a concentration of 1.0 M (0.57 mL, 3.0 eq.) was added at 0 °C and the mixture was then allowed to warm to room temperature. After 12 hours, the reaction was terminated by the addition of 1.0 mL of a 10% aqueous NaOH solution. The mixture was then desiccated using $MgSO_4$. The residual was filtered through celite pad and filtrate was concentrated. The mixture was purified by silica gel flash chromatography (petroleum ether : ethyl acetate, 20 : 1) to afford the desired product **16** with a yield of 85%.

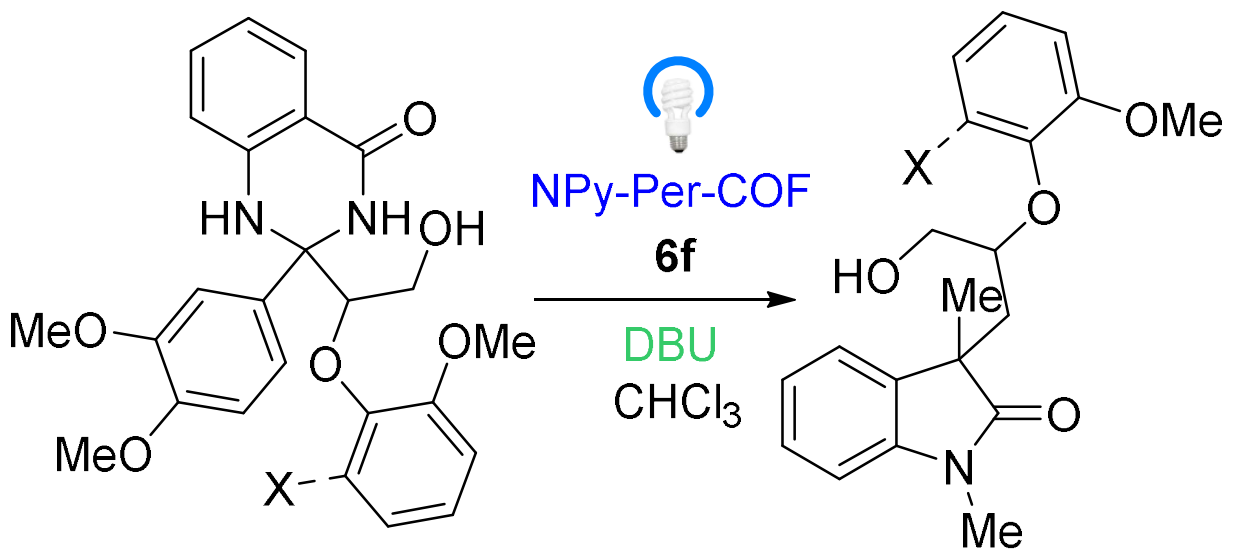

X = H **17**
X = OMe **18**

Under a nitrogen atmosphere, NPy-Per-COF (14.1 mg, 0.01 mmol based on the repeating unit), 2,3-dihydroquinazolin-4(1H)-one (0.4 mmol), **6f** (35.0 mg, 0.2 mmol) and DBU (60.8 mg, 0.4 mmol) were mixed in solvent (4.0 mL) in a Schlenk tube. The resulting mixture was stirred under blue LED irradiation (420 nm) at room temperature for 48 hours. Upon completion, the residual was filtered through celite pad and filtrate was concentrated. The mixture was purified by silica gel flash chromatography to afford the desired product **17** or **18**.

3. Characterizations

Characterizations of NPy-Per-COF and NPy-Py-COF

Supplementary Table 1. Fractional atomic coordinated for unit cell of NPy-Per-COF calculated using the Materials Studio modeling program after Pawley Refinement.

Space group	P1		
Calculated cell parameters	a = 28.1 Å, b = 30.7 Å, c = 3.8 Å, α = 97.9°, β = 74.8°, γ = 89.5°, R_{wp} = 4.77% and R_p = 3.09%		
Atoms	x	y	z
C1	0.50435	0.54448	-0.1073
C2	0.50509	0.58906	-0.18928
C3	0.50353	0.45493	0.03479
C4	0.50373	0.40931	0.08923
C5	0.46131	0.52107	-0.05556
C6	0.4639	0.60993	-0.21896
C7	0.42085	0.58802	-0.15696
C8	0.41997	0.54401	-0.07539
C9	0.37644	0.61136	-0.17071
C10	0.34031	0.58736	-0.28871
C11	0.29856	0.6092	-0.2962
C12	0.29191	0.6555	-0.18746
C13	0.32771	0.67969	-0.0689
C14	0.36933	0.65784	-0.05879
C15	0.24367	0.31157	0.3712
N16	0.24144	0.721	-0.11185
C17	0.20037	0.74752	-0.10499
C18	0.19801	0.79246	0.05236
C19	0.15822	0.81982	0.08032
C20	0.12078	0.80251	-0.06222
C21	0.12324	0.75761	-0.22316

C22	0.16255	0.73018	-0.24265
C23	0.08195	0.8306	-0.04774
C24	0.0782	0.87545	0.12758
C25	0.11398	0.8902	0.29768
C26	0.15447	0.86371	0.26014
C27	0.03647	0.90525	0.13073
C28	0.03853	0.95206	0.09897
C29	0.924	0.97473	-0.02454
C30	0.54589	0.47912	0.00256
C31	0.54564	0.38779	0.09772
C32	0.58745	0.41141	0.07962
C33	0.58631	0.45734	0.05061
C34	0.63291	0.38785	0.0728
C35	0.67702	0.40405	-0.10465
C36	0.71935	0.38099	-0.1306
C37	0.71894	0.34055	0.0112
C38	0.67537	0.32412	0.19016
C39	0.63294	0.3477	0.22391
C40	0.76265	0.68977	-0.37328
N41	0.76546	0.27705	0.06945
C42	0.80708	0.2493	0.02766
C43	0.80136	0.20906	0.17745
C44	0.84047	0.18009	0.13753
C45	0.88581	0.19115	-0.06144
C46	0.89164	0.23135	-0.21025
C47	0.85278	0.26028	-0.16561
C48	0.92473	0.16193	-0.10899
C49	0.91999	0.12241	0.05448
C50	0.87482	0.11297	0.26685

C51	0.83526	0.14067	0.29788
C52	0.96325	0.09436	0.0348
C53	0.96366	0.04693	0.0169
C54	0.08058	0.02348	0.08396
C55	0.54667	0.5239	-0.07861
C56	0.54703	0.61273	-0.2412
C57	0.58893	0.5928	-0.21854
C58	0.58877	0.54868	-0.14273
C59	0.63308	0.61842	-0.26823
C60	0.67612	0.59897	-0.49163
C61	0.71799	0.62267	-0.52793
C62	0.71765	0.66609	-0.34029
C63	0.67449	0.68579	-0.11999
C64	0.63258	0.66219	-0.08543
C65	0.76431	0.31619	-0.03511
N66	0.76416	0.72838	-0.17841
C67	0.80538	0.75467	-0.17386
C68	0.79887	0.79682	0.04166
C69	0.83768	0.82393	0.05987
C70	0.88374	0.80866	-0.14132
C71	0.89037	0.76647	-0.35506
C72	0.85163	0.73961	-0.3713
C73	0.92251	0.83571	-0.1254
C74	0.91624	0.87835	0.08574
C75	0.87029	0.89262	0.29294
C76	0.83134	0.86591	0.27739
C77	0.9586	0.9061	0.10048
C78	0.96112	0.95203	0.05537
C79	0.08034	0.97618	0.0775

C80	0.46096	0.47576	0.01341
C81	0.46206	0.38518	0.13288
C82	0.41968	0.40561	0.1253
C83	0.4195	0.4501	0.05941
C84	0.37498	0.38075	0.19045
C85	0.33312	0.40132	0.42452
C86	0.2907	0.37843	0.48335
C87	0.28923	0.33459	0.30906
C88	0.33115	0.31379	0.07671
C89	0.37368	0.33667	0.01871
C90	0.24754	0.67785	-0.19856
N91	0.24067	0.27168	0.20171
C92	0.19889	0.24539	0.23059
C93	0.20311	0.20374	0.01022
C94	0.16375	0.17654	0.02282
C95	0.11951	0.19083	0.26479
C96	0.1152	0.23245	0.48572
C97	0.15444	0.25963	0.46867
C98	0.08034	0.1633	0.2821
C99	0.08419	0.12199	0.0533
C100	0.12796	0.10933	-0.19869
C101	0.16764	0.13565	-0.20701
C102	0.04124	0.09447	0.0579
C103	0.04056	0.04756	0.06659
C104	0.92559	0.02155	-0.04955
C105	-0.00044	0.11683	0.05435
C106	-0.00251	0.97557	0.07427
C107	-0.00706	0.88448	0.14909
C108	-0.00125	0.02387	0.05353

H109	0.46561	0.64345	-0.29278
H110	0.38596	0.52903	-0.01792
H111	0.34433	0.55171	-0.38089
H112	0.27156	0.58988	-0.39042
H113	0.32335	0.71542	0.02095
H114	0.3954	0.67735	0.04537
H115	0.21292	0.32912	0.55717
H116	0.22679	0.80564	0.16286
H117	0.09492	0.7438	-0.33607
H118	0.16325	0.69596	-0.37227
H119	0.0555	0.81762	-0.18474
H120	0.11087	0.9221	0.46581
H121	0.18185	0.87694	0.3865
H122	0.89342	0.95759	-0.07492
H123	0.54537	0.35224	0.11412
H124	0.61727	0.47564	0.07386
H125	0.67925	0.43359	-0.2388
H126	0.75251	0.39414	-0.27274
H127	0.67403	0.2933	0.30774
H128	0.60038	0.33442	0.37276
H129	0.79422	0.67379	-0.56041
H130	0.76635	0.2006	0.32757
H131	0.92626	0.24026	-0.3643
H132	0.85912	0.29041	-0.29127
H133	0.95885	0.17038	-0.27287
H134	0.87011	0.08487	0.41928
H135	0.80096	0.1322	0.45998
H136	0.11331	0.0394	0.09723
H137	0.54731	0.64674	-0.30444

H138	0.62233	0.53484	-0.13517
H139	0.67728	0.56552	-0.63974
H140	0.75077	0.60693	-0.70114
H141	0.67316	0.71921	0.02879
H142	0.59992	0.67772	0.09151
H143	0.79647	0.33177	-0.16415
H144	0.76339	0.80829	0.19623
H145	0.92555	0.75428	-0.51081
H146	0.85859	0.70733	-0.53876
H147	0.95774	0.82342	-0.27769
H148	0.86429	0.92457	0.46617
H149	0.79632	0.87796	0.43753
H150	0.11446	0.96051	0.04114
H151	0.46227	0.35065	0.18326
H152	0.38622	0.46317	0.04
H153	0.33331	0.43502	0.56513
H154	0.25895	0.39503	0.66552
H155	0.33105	0.28003	-0.06272
H156	0.40535	0.32026	-0.16614
H157	0.22022	0.65739	-0.27513
H158	0.23716	0.19292	-0.17502
H159	0.08152	0.24387	0.67338
H160	0.14945	0.29121	0.64643
H161	0.04678	0.17408	0.47398
H162	0.13149	0.07909	-0.39211
H163	0.20102	0.12465	-0.40063
H164	0.89674	0.03639	-0.12717
H165	-0.0002	0.1529	0.06849
H166	-0.01005	0.84989	0.19484

Supplementary Table 2. Fractional atomic coordinated for unit cell of NPy-Py-COF calculated using the Materials Studio modeling program after Pawley Refinement.

Space group	P-1		
Calculated cell parameters	a = 28.0 Å, b = 3.8 Å, c = 28.5 Å, α = 92.7°, β = 88.9°, γ =108.1°, R_{wp} = 5.77% and R_p = 4.32%		
Atoms	x	y	z
C1	0.54365	-0.04079	0.54657
C2	0.5498	0.01898	0.59636
C3	0.57857	-0.15734	0.51864
C4	0.68624	0.21736	0.6268
C5	0.63377	-0.11499	0.69081
N6	0.76579	-0.06763	0.76588
C7	0.76783	-0.22316	0.80495
C8	0.81574	-0.16331	0.82787
C9	0.85923	-0.27436	0.89593
C10	0.90722	-0.0589	0.87811
C11	0.9068	0.09351	0.83308
C12	0.04365	-0.01103	0.04793
C13	0.04811	0.04345	0.09787
C14	0.08053	-0.10979	0.02066
C15	0.18519	0.33229	0.12827
C16	0.1379	-0.028	0.19051
C17	0.27415	-0.05895	0.30285
C18	0.27846	0.08364	0.25831
C19	0.31672	-0.05772	0.32884
C20	0.35509	-0.22016	0.39674
C21	0.40499	-0.04446	0.37901
C22	0.40851	0.11308	0.33447
C23	0.53574	-0.10425	0.44774

C24	0.53427	-0.10452	0.39762
C25	0.57477	-0.18775	0.47086
C26	0.66636	0.10079	0.34915
C27	0.60594	-0.30496	0.29279
N28	0.78743	0.10439	0.25594
C29	0.78564	-0.07086	0.21552
C30	0.83124	-0.02374	0.18809
C31	0.86881	-0.18207	0.11577
C32	0.91803	0.0499	0.12958
C33	0.9213	0.2381	0.17481
C34	0.03931	-0.05544	0.94878
C35	0.03941	-0.04553	0.89867
C36	0.07845	-0.13093	0.97275
C37	0.1732	0.22444	0.85611
C38	0.12077	-0.18793	0.79785
C39	0.2533	-0.10448	0.71452
C40	0.30302	0.08596	0.72624
C41	0.34334	0.0869	0.69564
C42	0.37288	-0.11951	0.62088
C43	0.42343	0.0872	0.63188
C44	0.43204	0.29009	0.67644
C45	0.49733	-0.03027	0.47539
C46	0.50918	0.06701	0.62242
C47	0.99938	0.00243	0.97562
C48	0.00582	0.06851	0.12324
C49	0.28208	-0.31202	0.64003
C50	0.24392	-0.3022	0.67085
C51	0.36968	0.24832	0.26359
C52	0.32712	0.23403	0.2392

H53	0.61017	-0.22272	0.53434
H54	0.72341	0.35554	0.61156
H55	0.73433	-0.39753	0.823
H56	0.85666	-0.40998	0.92953
H57	0.94228	0.25839	0.81702
H58	0.11242	-0.16582	0.0371
H59	0.22081	0.49495	0.11222
H60	0.1352	-0.15801	0.22454
H61	0.23677	-0.1835	0.31859
H62	0.35016	-0.36006	0.43025
H63	0.44563	0.24387	0.31875
H64	0.60359	-0.27436	0.45108
H65	0.59767	-0.46258	0.25892
H66	0.75055	-0.2559	0.20003
H67	0.86349	-0.34448	0.08224
H68	0.95792	0.41765	0.18792
H69	0.10882	-0.20222	0.95358
H70	0.21	0.40297	0.86898
H71	0.11511	-0.34412	0.76383
H72	0.31272	0.24579	0.75978
H73	0.36443	-0.2877	0.5878
H74	0.47049	0.4522	0.6869
H75	0.5121	0.08959	0.66098
H76	0.00748	0.08495	0.16189
H77	0.27283	-0.47103	0.60637
H78	0.20517	-0.45694	0.66029
H79	0.40685	0.36978	0.24736
H80	0.33293	0.34565	0.20389

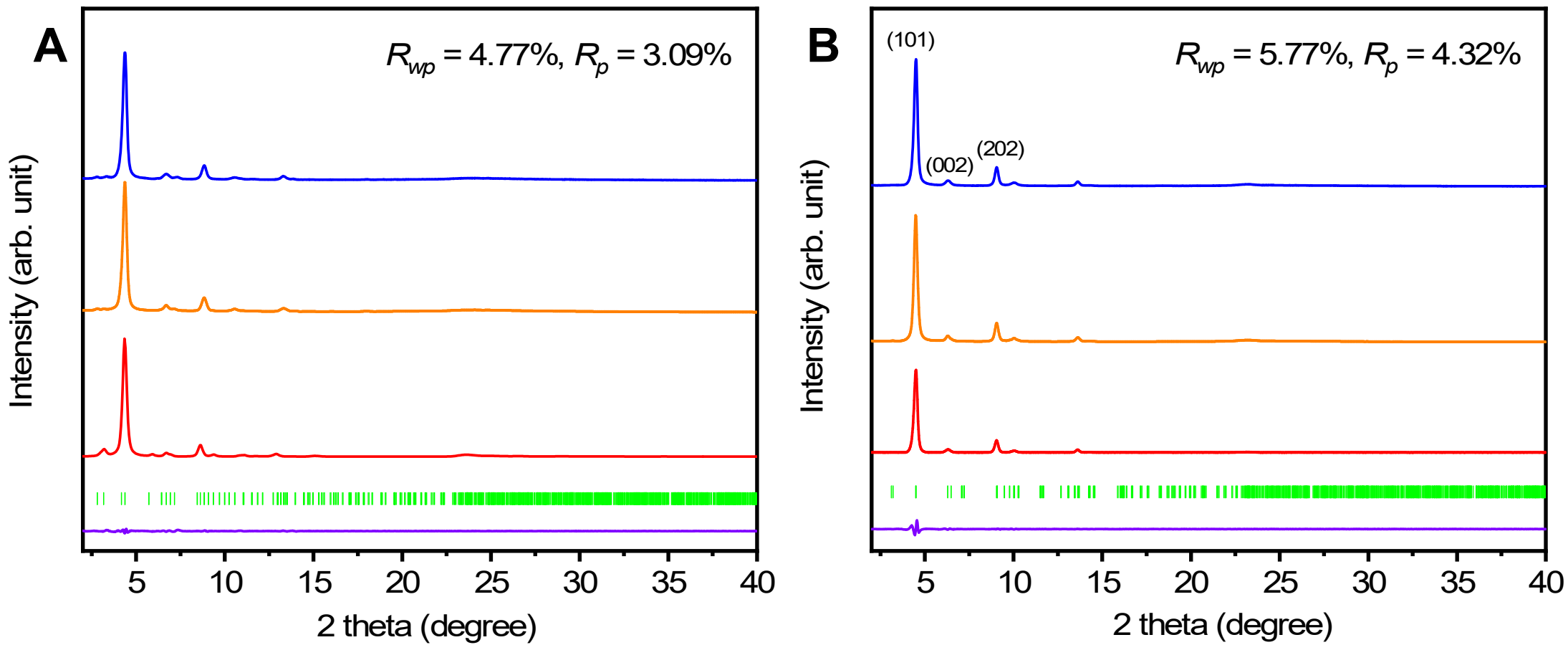

Supplementary Fig. 2. PXRD patterns of NPy-Per-COF (A) and NPy-Py-COF (B). The experimental data (blue), the Pawley refined profiles (orange), the calculated patterns of the eclipsed AA stacking (red), the Bragg diffractions (green) and the refinement differences (purple). arb. unit = arbitrary units.

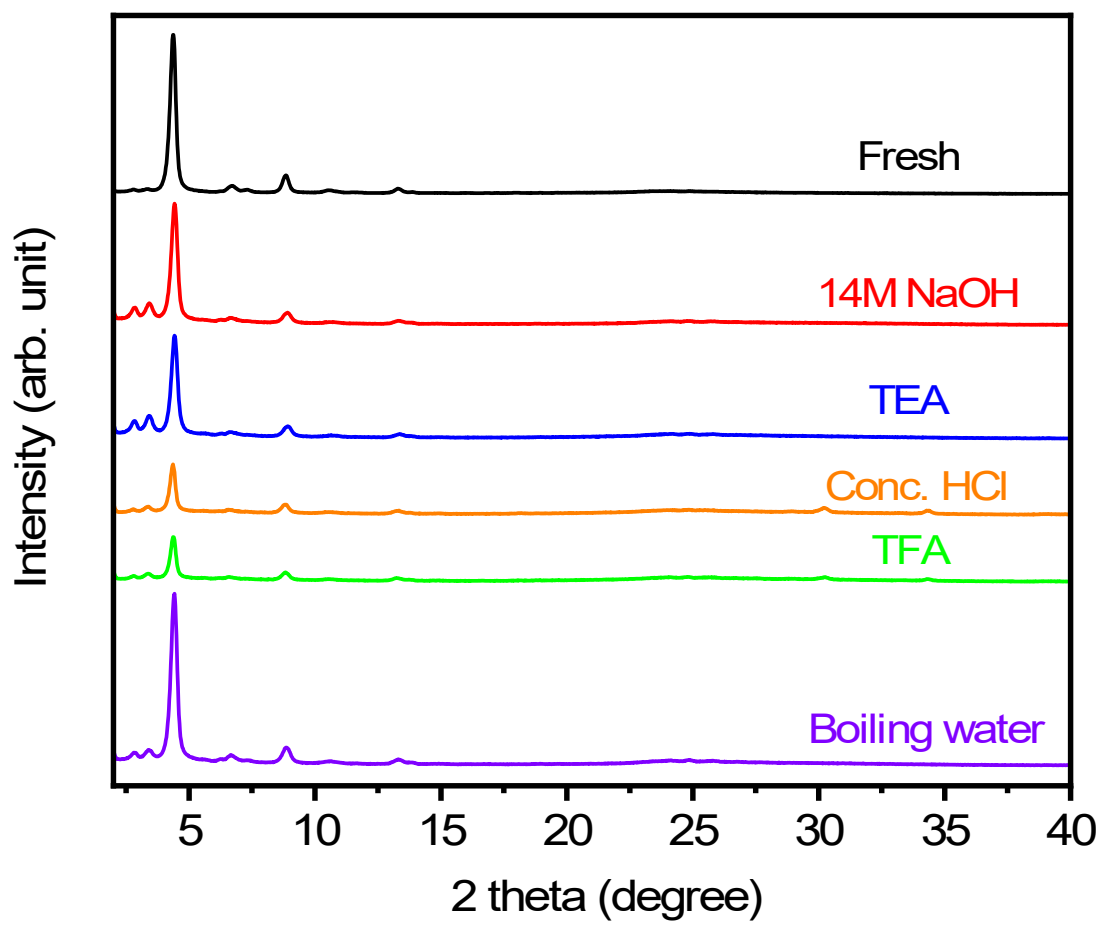

Supplementary Fig. 3. PXRD patterns of NPy-Per-COF before and after treatment in 14 N NaOH, triethylamine, Conc. HCl, trifluoroacetic acid and boiling water for 3 days. arb. units = arbitrary units.

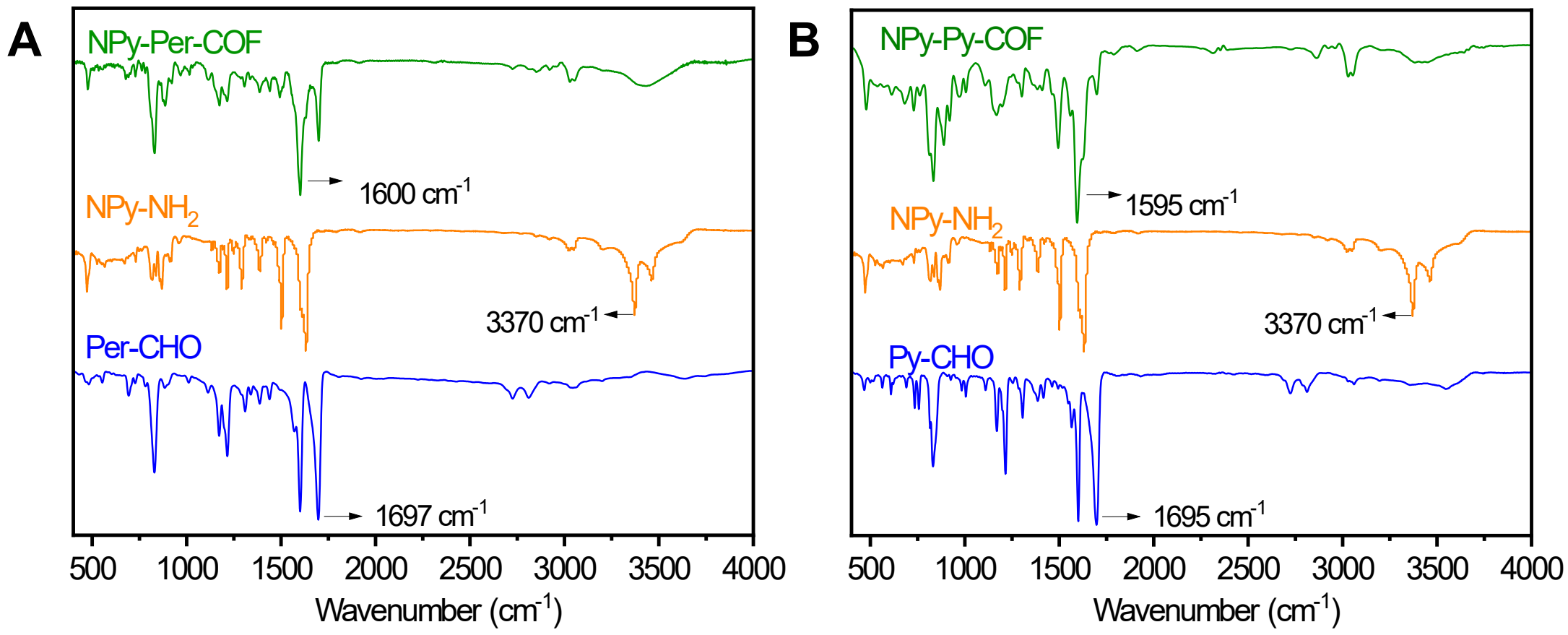

Supplementary Fig. 4. Fourier transform infrared (FT-IR) spectrum NPy-Per-COF (A) and NPy-Py-COF (B) together with the monomers.

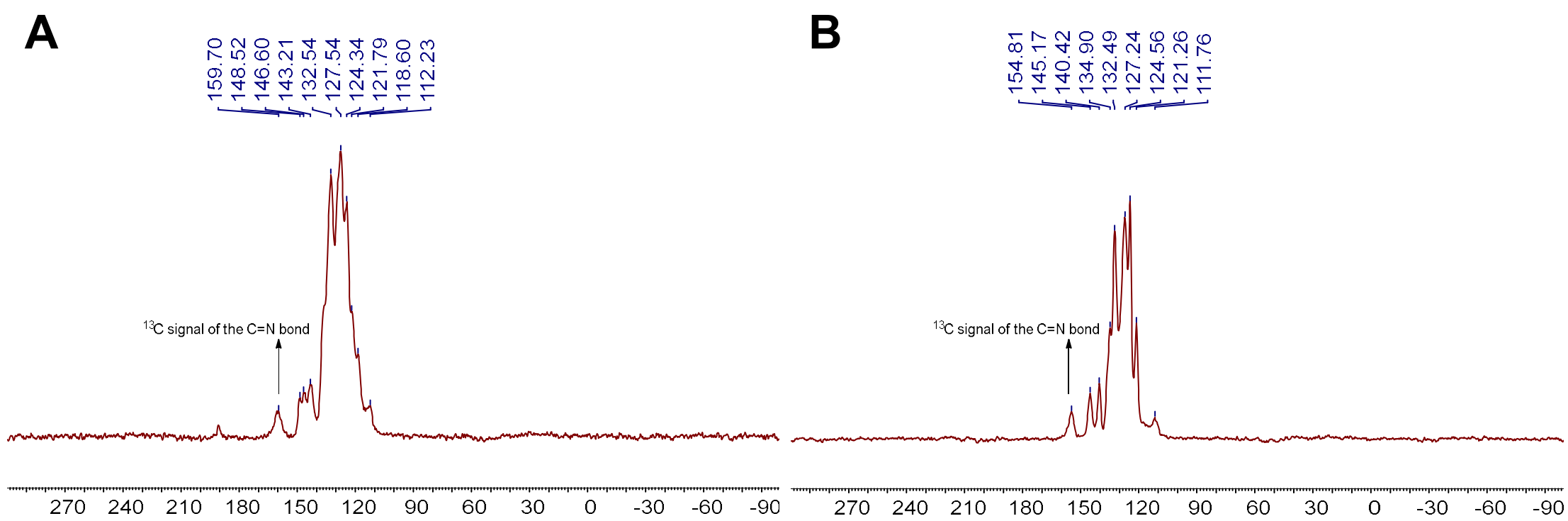

Supplementary Fig. 5. ^{13}C-CP/TOSS NMR spectra of NPy-Per-COF (A) and NPy-Py-COF (B).

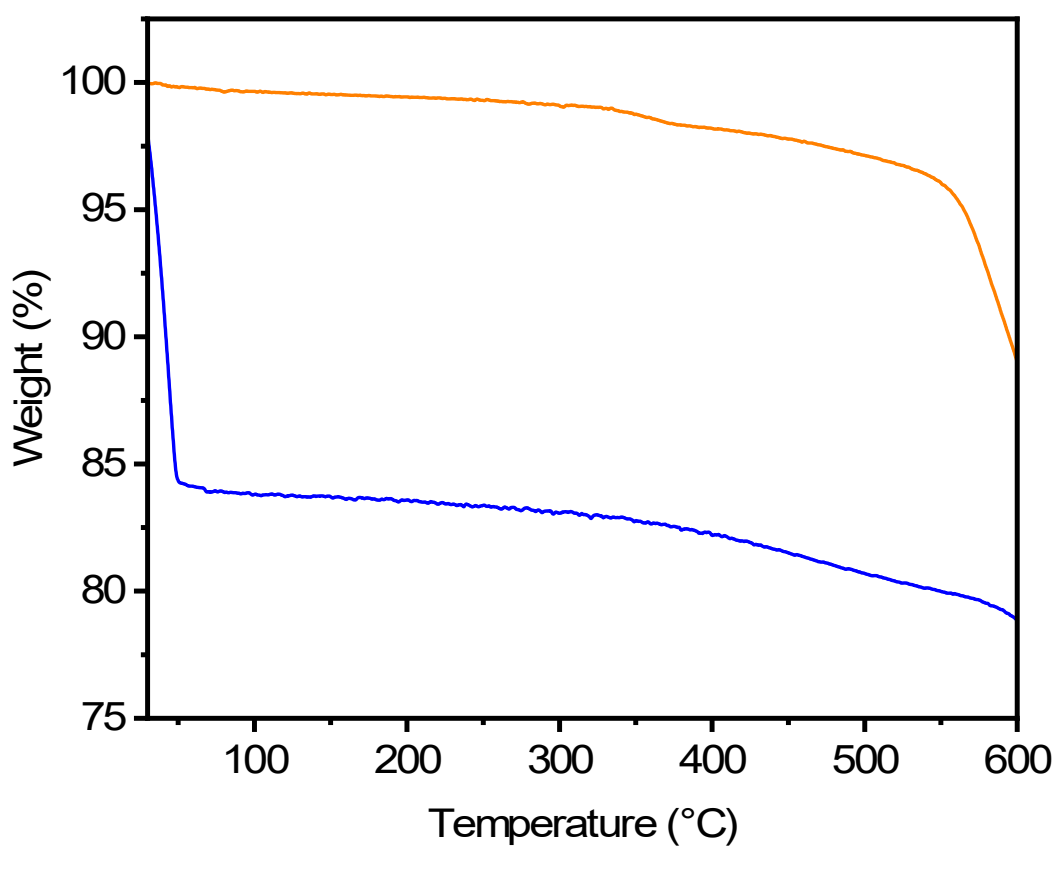

Supplementary Fig. 6. TGA of NPy-Per-COF (orange line) and NPy-Py-COF (blue line). TGA was performed with a temperature range from 30 to 600 °C and a heating rate of 10 K/min in nitrogen atmosphere.

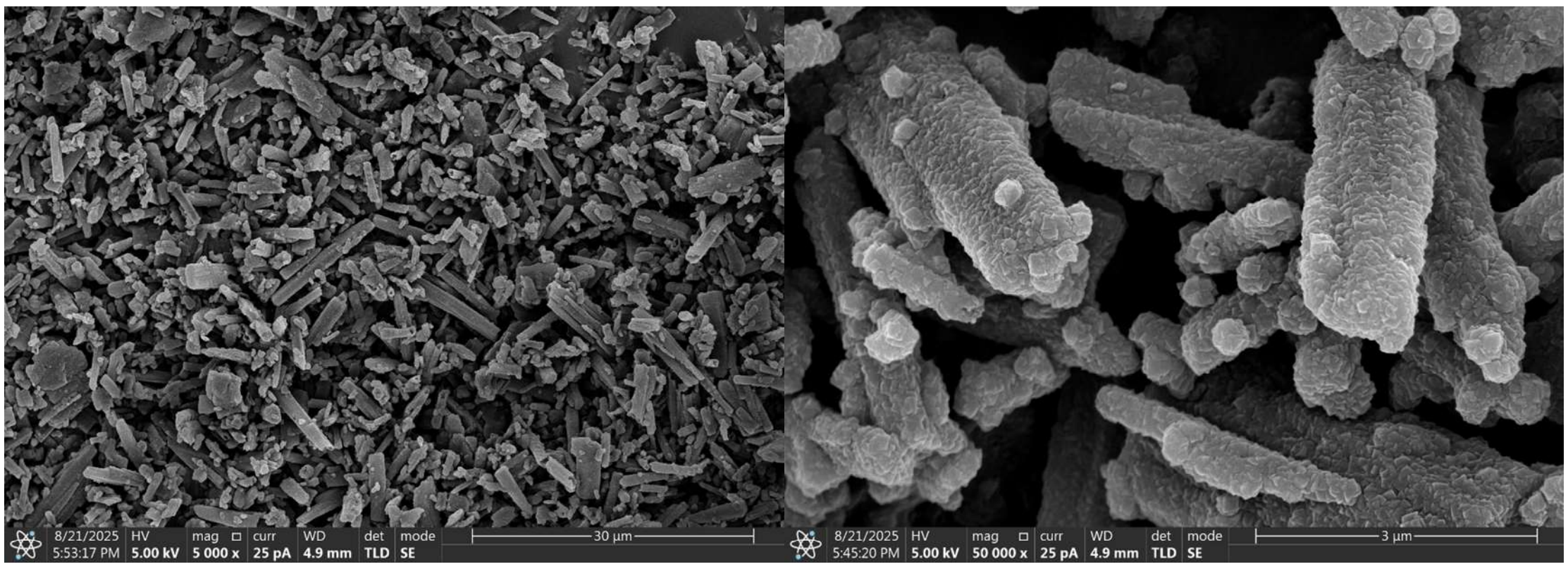

Supplementary Fig. 7. SEM images of NPy-Per-COF. Scale bars, 30 μm (left) and 3 μm (right).

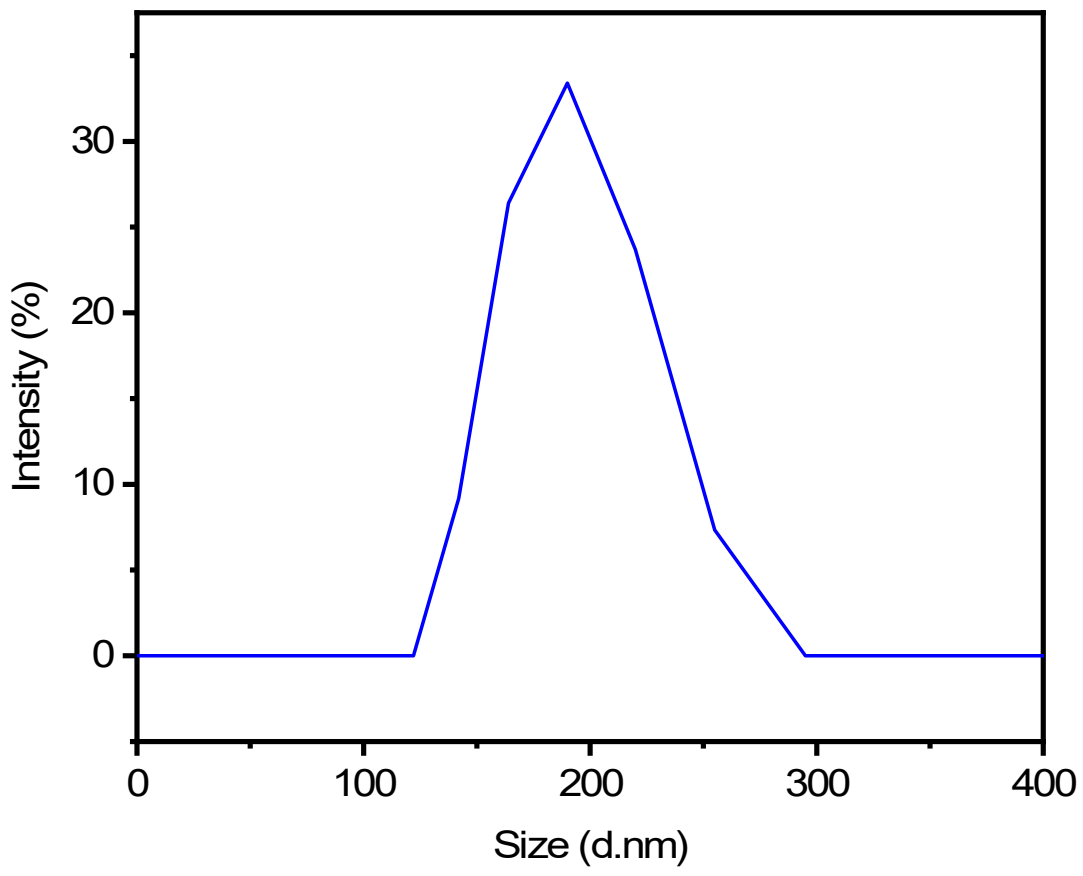

Supplementary Fig. 8. DLS number distributions of NPy-Per-COF. Z-average diameter is 186.9 nm and PdI value is 0.34.

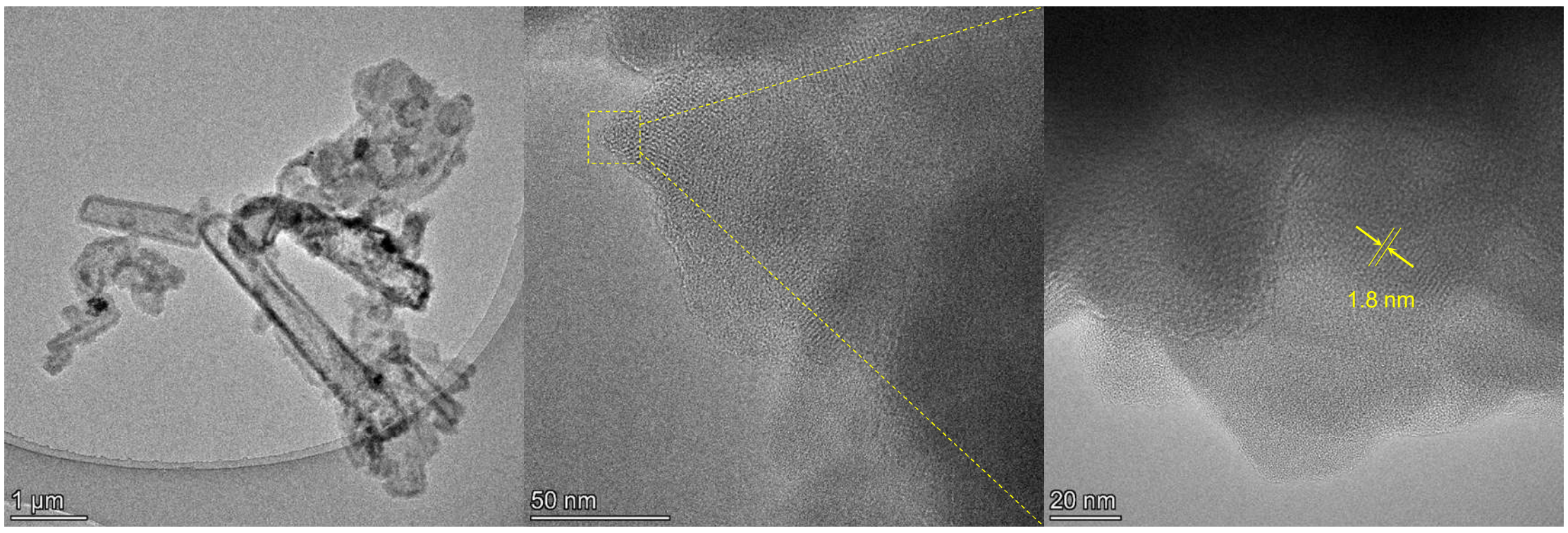

Supplementary Fig. 9. HR-TEM images of NPy-Per-COF. The pore structure with a periodicity of ~ 1.8 nm (right) is indicated by the yellow outline. Scale bars, 1 μm (left), 50 nm (middle) and 20 nm (right).

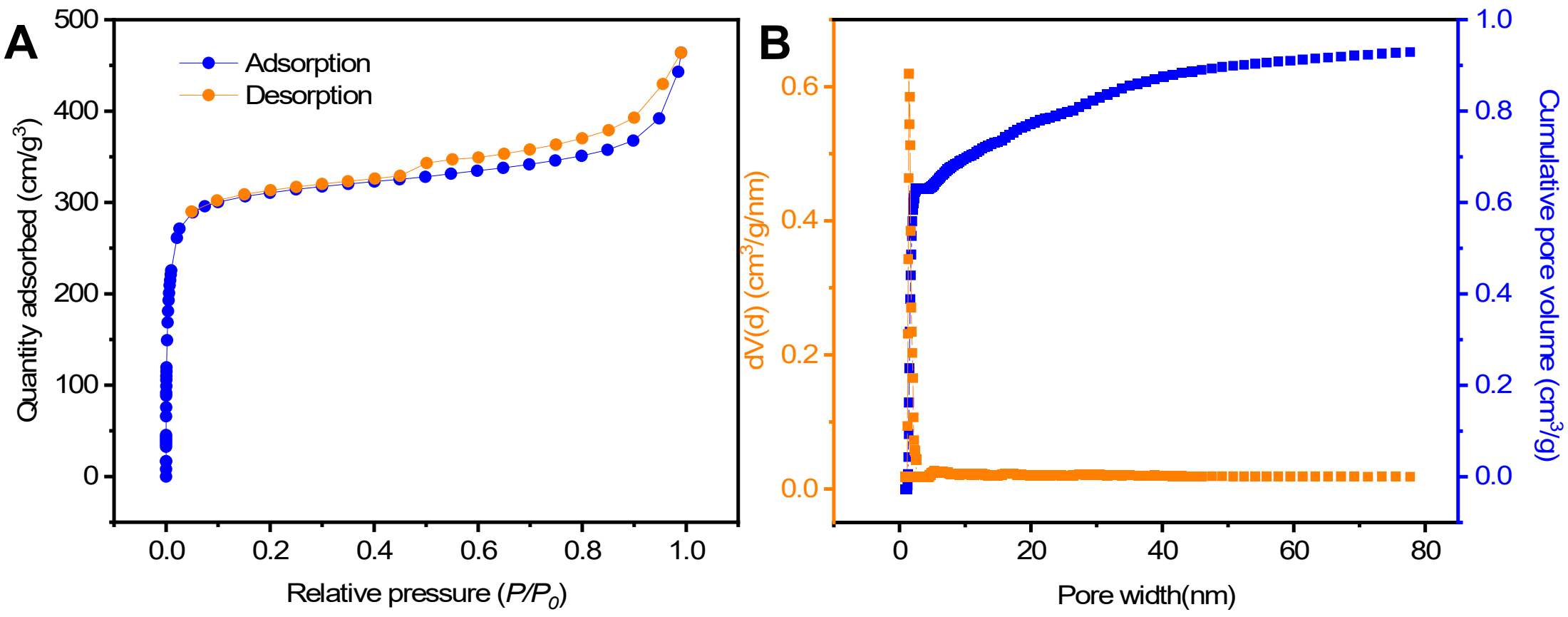

Supplementary Fig. 10. BET analysis of NPy-Per-COF. (A) Nitrogen adsorption (blue line) and desorption (orange line) isotherm at 77 K of NPy-Per-COF. (B) Pore size distribution of fresh NPy-Per-COF.

Theoretical Results

Ground states of photocatalysts

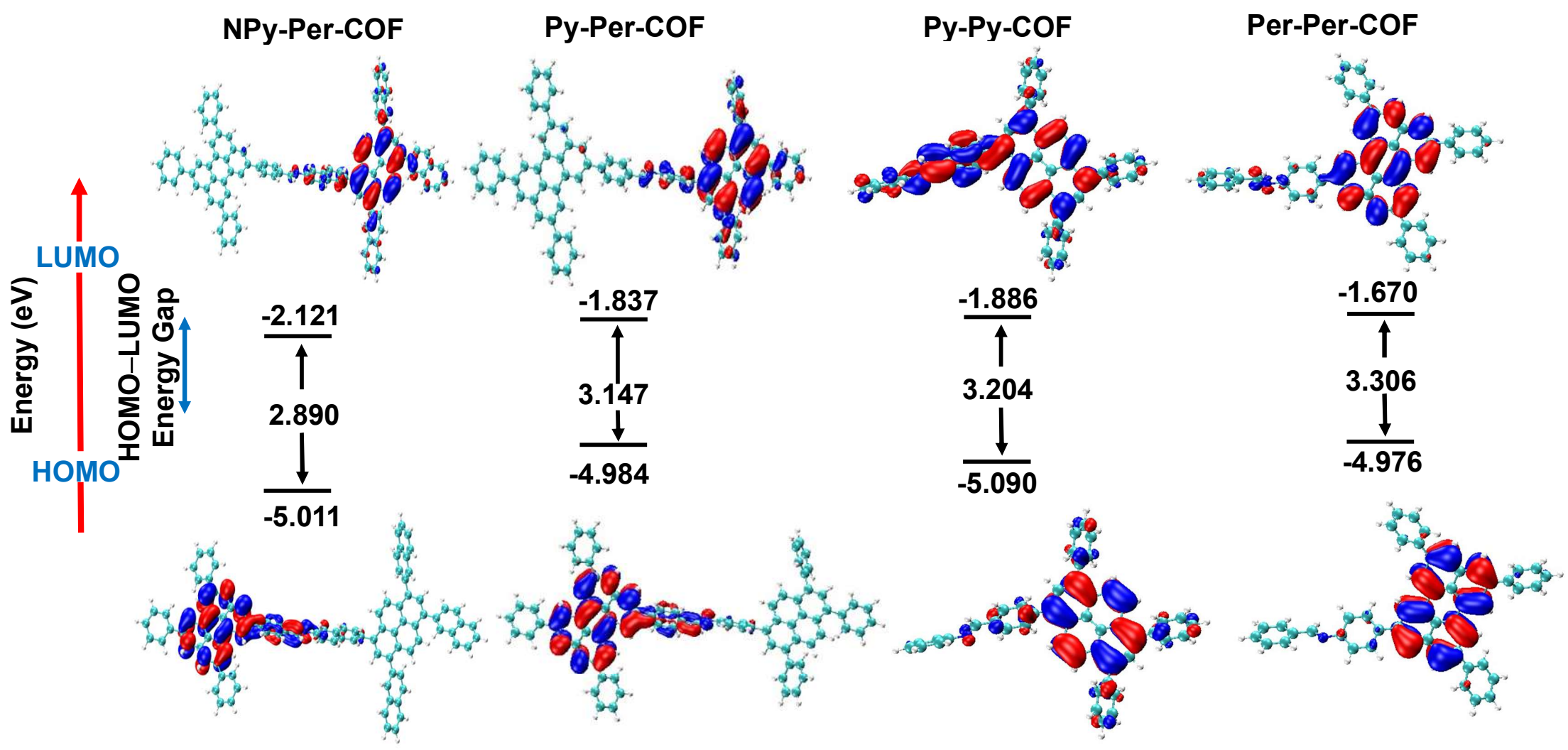

Supplementary Fig. 11. Comparative frontier electronic structures of the D–A pairs: HOMO and LUMO energy-level diagrams.

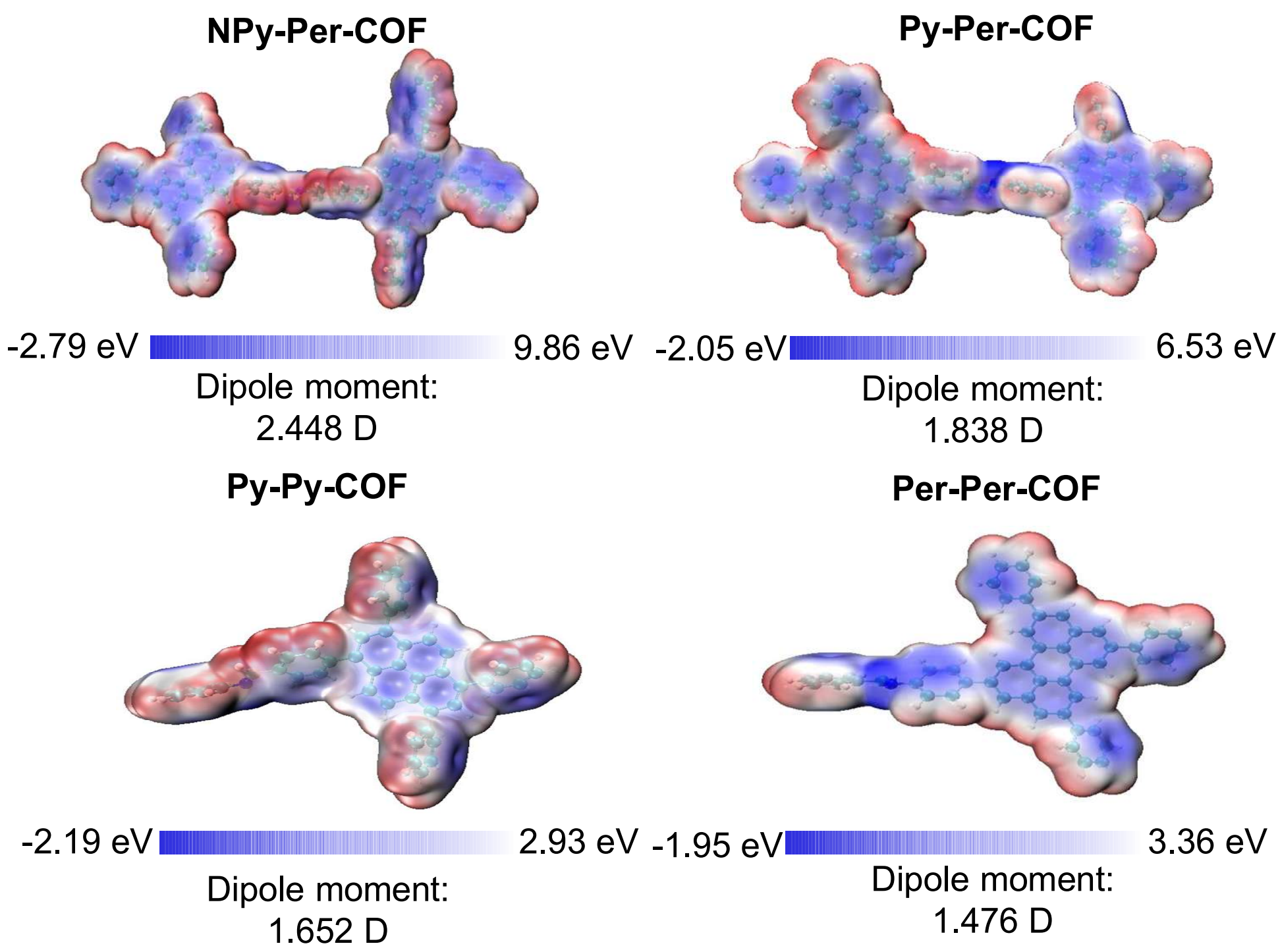

Supplementary Fig. 12. Comparative electrostatic potential (ESP) distributions and dipole moments of the D–A pairs.

Excited states of photocatalysts

Supplementary Table 3. Excitation characteristics (S_r and D) of the D–A pairs, derived from Multiwfn.

Item	D (Å)	S_r	S_r/D (Å)$^{-1}$
NPy-Per-COF	13.522	0.505	0.0373
Py-Per-COF	12.424	0.641	0.0516
Py-Py-COF	7.689	0.583	0.0758
Per-Per-COF	10.884	0.730	0.0671

The S_r represents the calculated overlap of hole-electron distribution, and D represents the calculated distance between hole and electron.

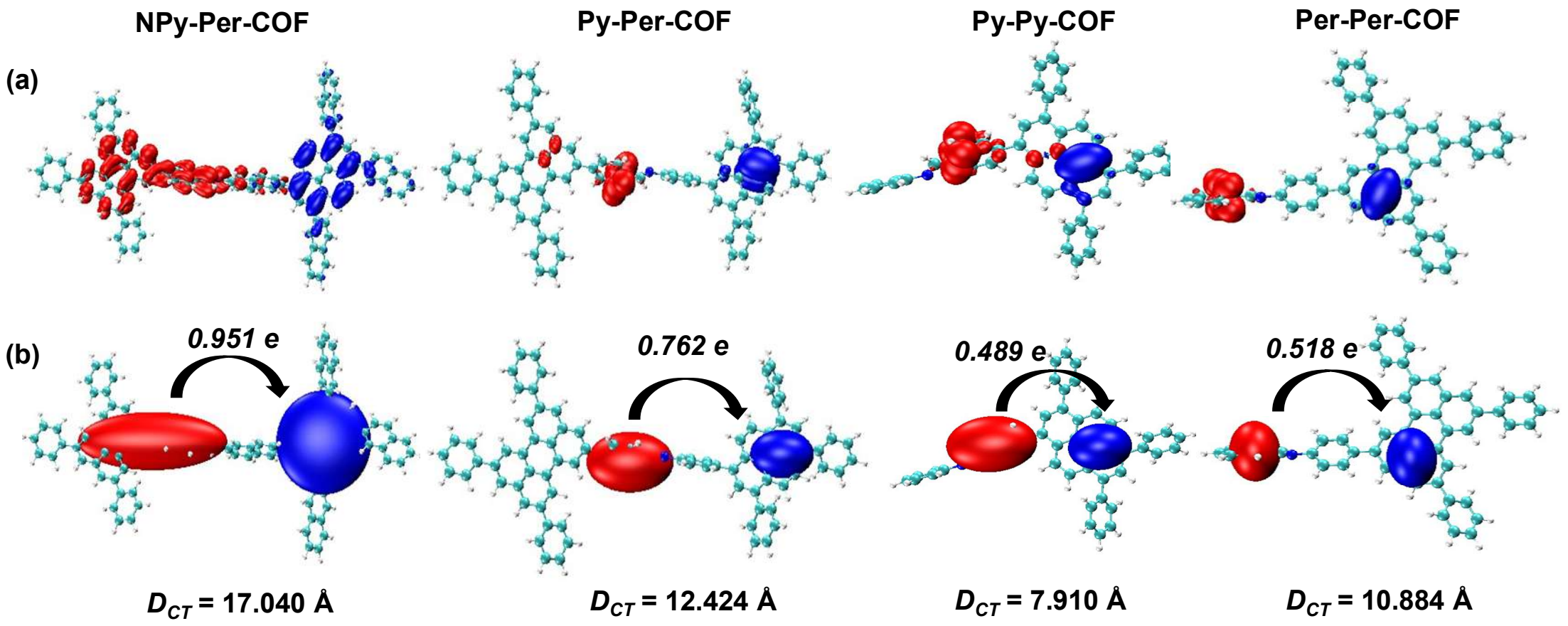

Supplementary Fig. 13. Comparative DFT analysis elucidating the origin of the activity differences among the D–A pairs. (a) Computed charge-density difference (Δρ) between the ground and excited states of the D–A pairs at an isovalue of 0.0005 a.u. (b) Charge centroids of the corresponding density redistribution. Blue and red denote an increase and a decrease in electron density, respectively.

Stabilization of the one-electron-reduced state

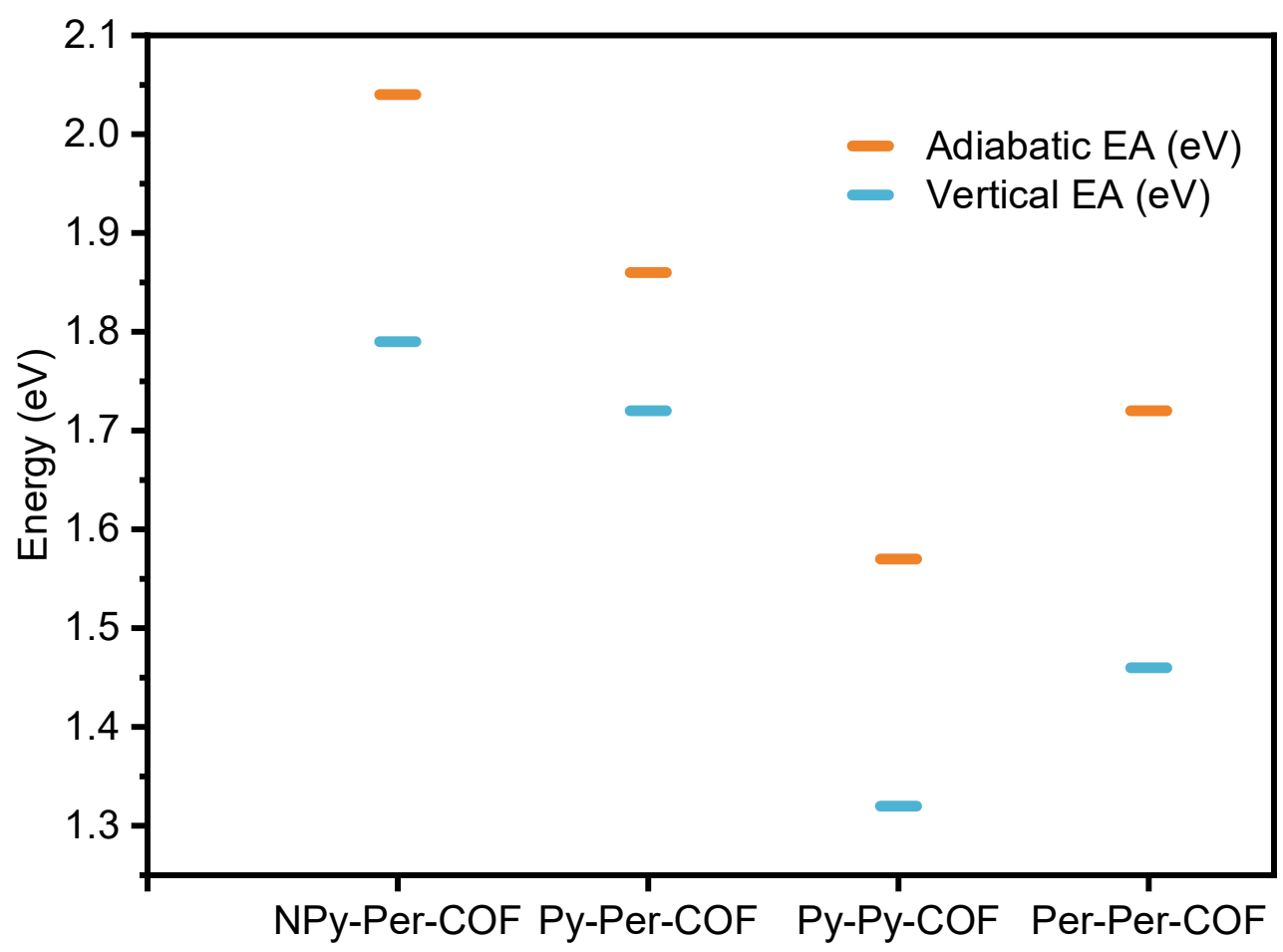

Supplementary Fig. 14. Calculated electron affinities of the D–A pairs.

Cyclic voltammetry

A standard three-electrode cell configuration was used to collect cyclic voltammograms at room temperature with a Chi600e electrochemical workstation. Each electrolyte solution contains 0.1 M tetrabutylammonium hexafluorophosphate ($TBAPF_6$).

To determine the cyclic voltammetry curve for NPy-Per-COF. The 5 mg as-prepared materials were dispersed in a mixture of ethanol (2.0 mL) and Nafion solution (0.5 mL) by sonication and then cast onto the glassy carbon disc electrode surface. NPy-Per-COF had a reduction potential at -0.93 V, due to the reduction potential of the $PC^*/PC^{\bullet-}$ couple. According to $E_{1/2}(PC^*/PC^{\bullet-}) = E^{0-0} + E_{1/2}(PC/PC^{\bullet-})$, the redox potential of the excited-state $PC^*/PC^{\bullet-}$ couple was calculated as 1.45 V based on an excitation energy change (E^{0-0}) of 2.38 eV. The redox potential of the excited-state $PC^*/PC^{\bullet-}$ couple of NPy-Py-COF was measured by this method.

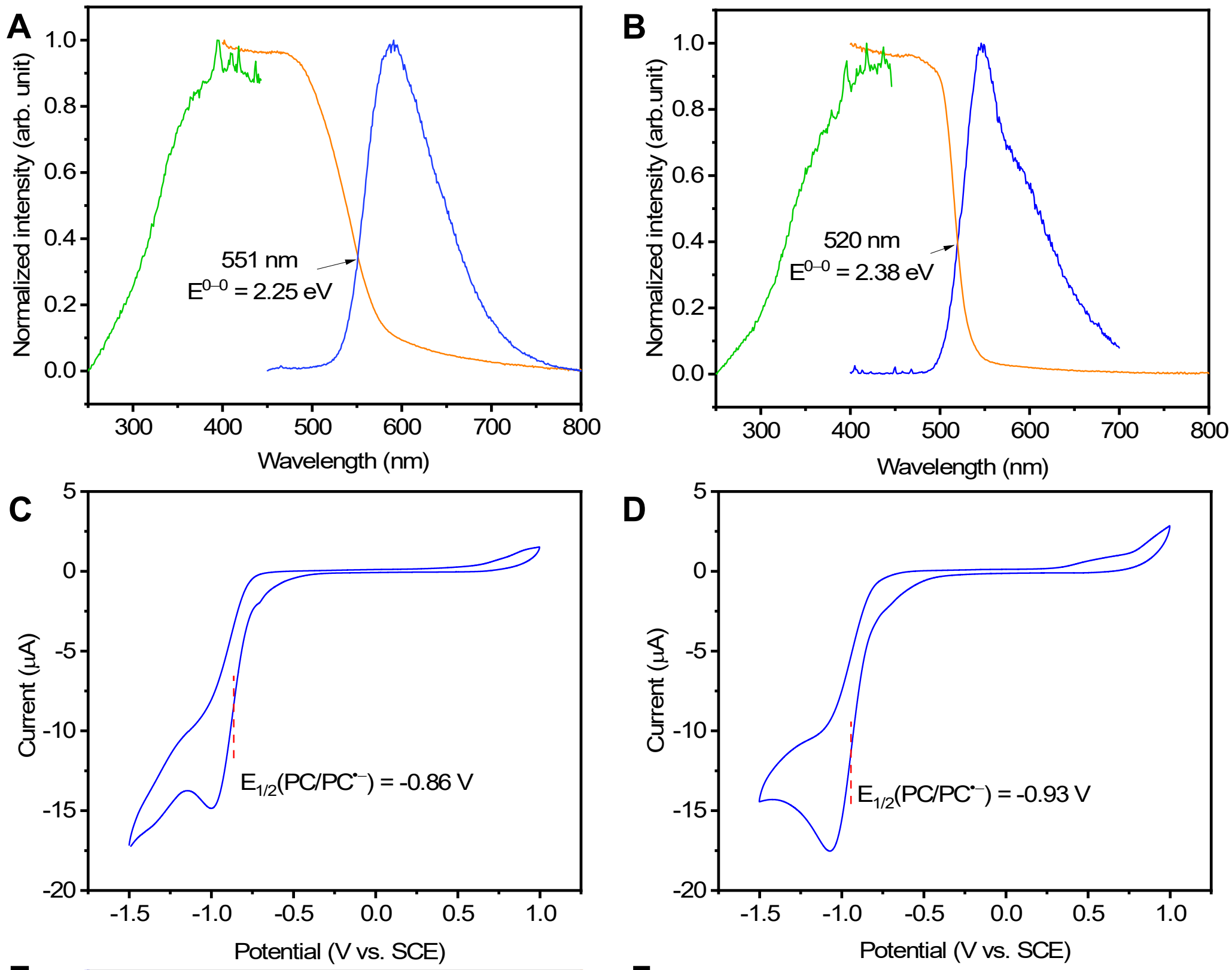

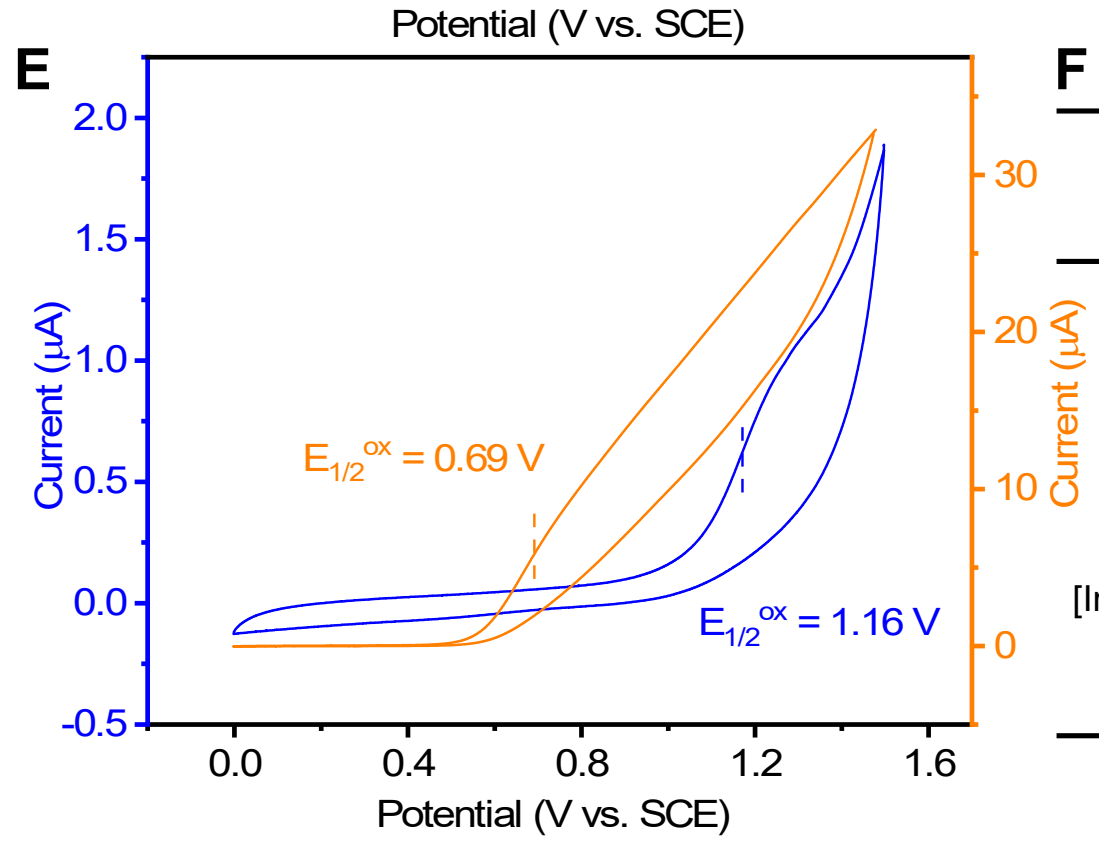

F

Photocatalyst	$E_{1/2}(PC^{*}/PC^{•-})$	E^{0-0}	$E_{1/2}(PC/PC^{•-})$
NPy-Per-COF	1.45 V	2.38 eV	-0.93 V
NPy-Py-COF	1.39 V	2.25 eV	-0.86 V
fac-Ir(ppy)$_3$	0.31 V	N. R.	-2.19 V
[Ir(ppy)$_2$(dtbbpy)]PF$_6$	0.66 V	N. R.	-1.50 V
4CzIPN	1.35 V	N. R.	-1.21 V

Supplementary Fig. 15. Redox potentials of different photocatalysts and oxidation potential of 2a. (A) Normalized absorption (red line), excitation (green line), and emission (blue line) spectra of NPy-Py-COF. (B) Normalized absorption (red line), excitation (green line), and emission (blue line) spectra of NPy-Per-COF. (C) Cyclic voltammogram of NPy-Py-COF in $CHCl_3$. PC = NPy-Py-COF. (D) Cyclic voltammogram of NPy-Per-COF in $CHCl_3$. PC = NPy-Per-COF. (E) Cyclic voltammograms of **2a** in $CHCl_3$ (blue line) and the mixture of **2a** and DABCO in $CHCl_3$ (red line). A 3 mm glassy carbon disc electrode as the working electrode, a saturated calomel electrode (SCE) as the reference electrode, and a platinum wire as the counter electrode were employed. The reduction potential was measured by cathodic scanning at a scan rate of 5 mV/s. (F) Redox potentials of different photocatalysts. $E_{1/2}$ vs SCE. arb. units = arbitrary

units.

Simulated UV−vis absorption spectrum of the NPy-Per-COF*

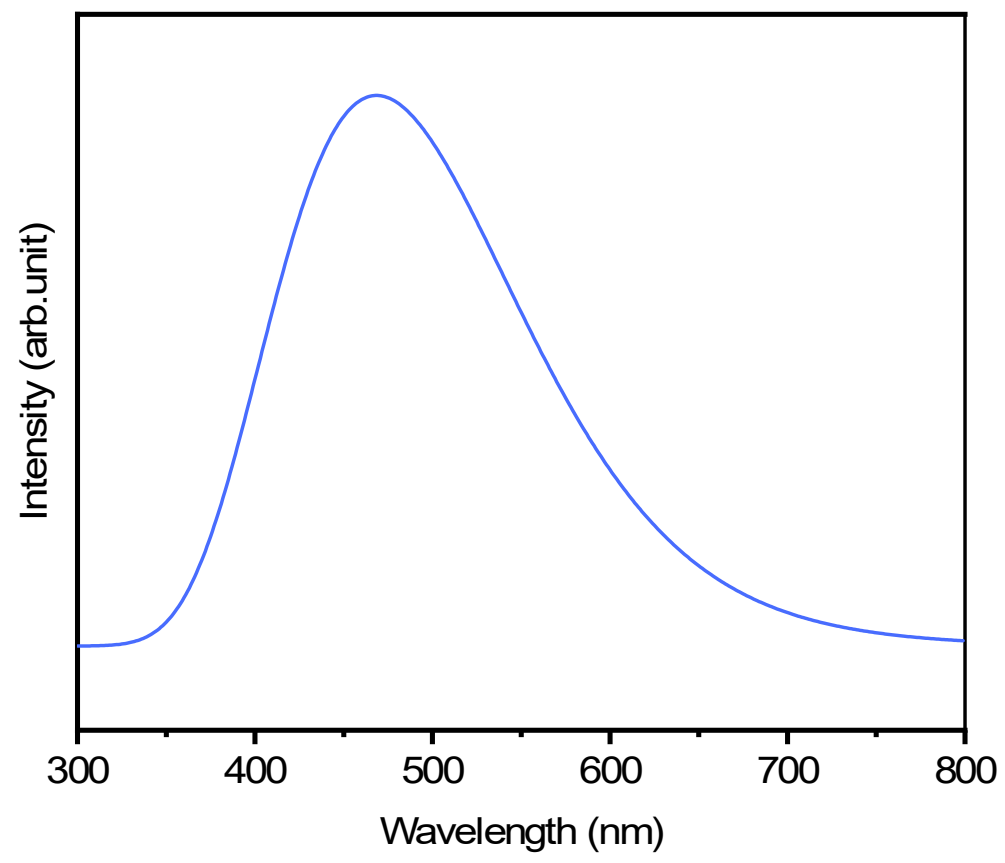

Supplementary Fig. 16. Simulated UV−vis absorption spectrum of the NPy-Per-COF* in chloroform. It indicates the dominant $S_0 \rightarrow S_1$ transition at 539 nm.

Band structure

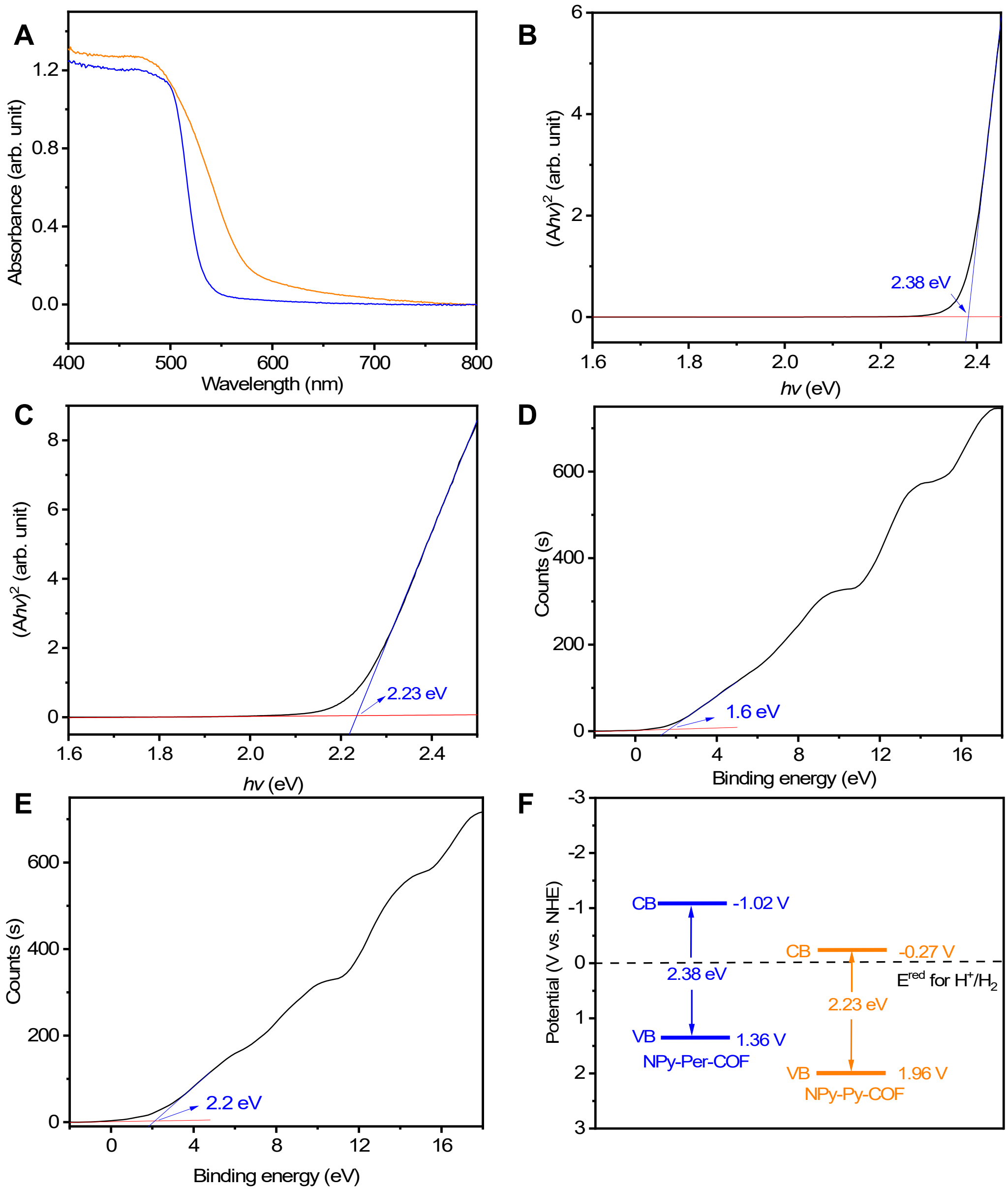

Supplementary Fig. 17. Energy band analyses. (A) UV−vis spectra of NPy-Per-COF (blue line) and NPy-Py-COF (orange line). (B) Tauc plots for NPy-Per-COF. (C) Tauc plots for NPy-Py-COF. (D) VB-XPS valence band spectra for NPy-Per-COF. (E) VB-XPS valence band spectra for NPy-Py-COF. (F) Energy band alignment. arb. units = arbitrary units.

Optimizations of the reaction

Supplementary Table 4. Optimizations of the reaction.

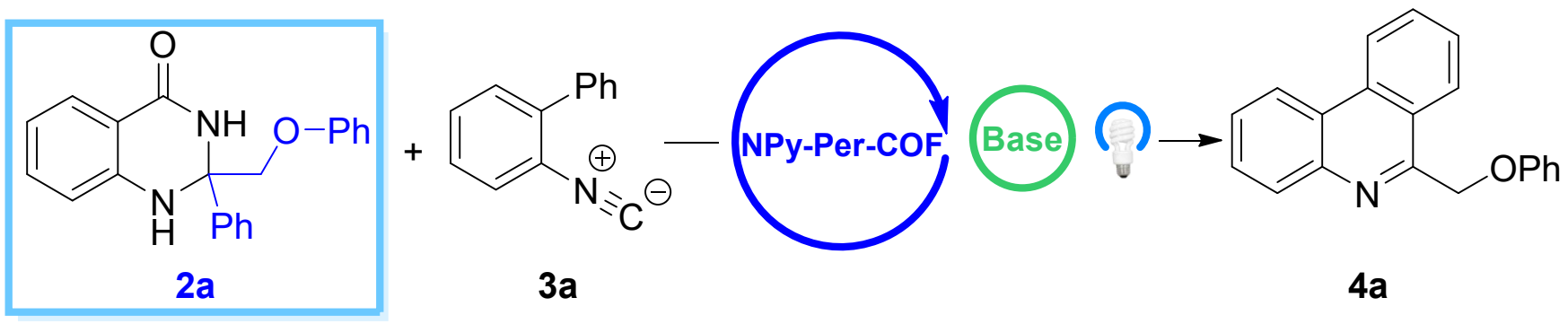

Entry	Base	Solvent	Yield of **4a**/%[a]
1	DIPEA	CH_3CN	0
2	DMAP	CH_3CN	0
3	Et_3N	CH_3CN	0
4	DABCO	CH_3CN	63
5	DBU	CH_3CN	13
6	DABCO	DMF	0
7	DABCO	DMSO	0
8	DABCO	DCE	68
9	DABCO	THF	0
10	DABCO	$CHCl_3$	82
11[b]	DABCO	$CHCl_3$	0
12[c]	DABCO	$CHCl_3$	0
13	-	$CHCl_3$	0
14[d]	DABCO	$CHCl_3$	42

All reactions were carried out on a 0.1 mmol scale with 1.0 eq. isocyanide **3a**, 2.0 eq. unstrained ketone **2a**, 2 mol% NPy-Per-COF and 2.0 eq. base in 1.0 mL solvent under irradiation of blue LEDs with N_2 protection at room temperature. DIPEA is diisopropylethylamine. DMAP is 4-dimethylaminopyridine. DABCO is 1,4-diazabicyclo[2.2.2]octane. DBU is 1,8-diazabicyclo[5.4.0]undec-7-ene. [a]Isolated yield. [b]The reaction was performed in the dark. [c]The reaction was performed under air. [d]1 mol% NPy-Per-COF was used.

PXRD and BET analyses of the recycled NPy-Per-COF

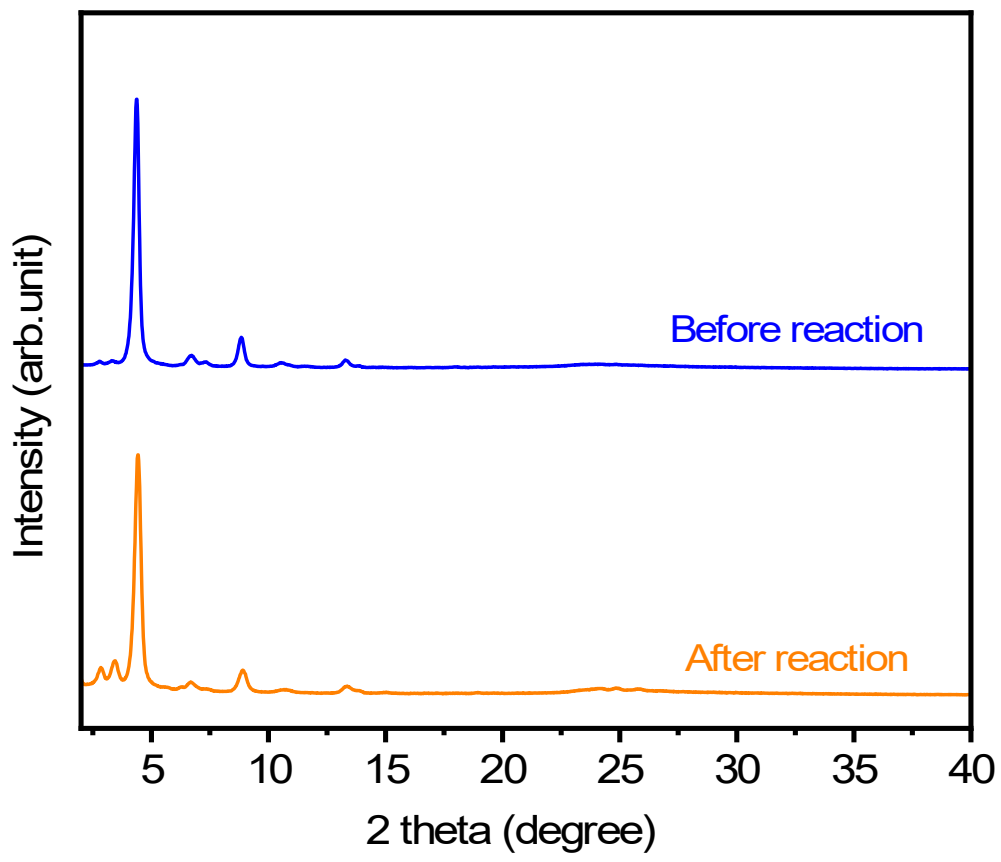

Supplementary Fig. 18. PXRD analysis of recycled NPy-Per-COF.

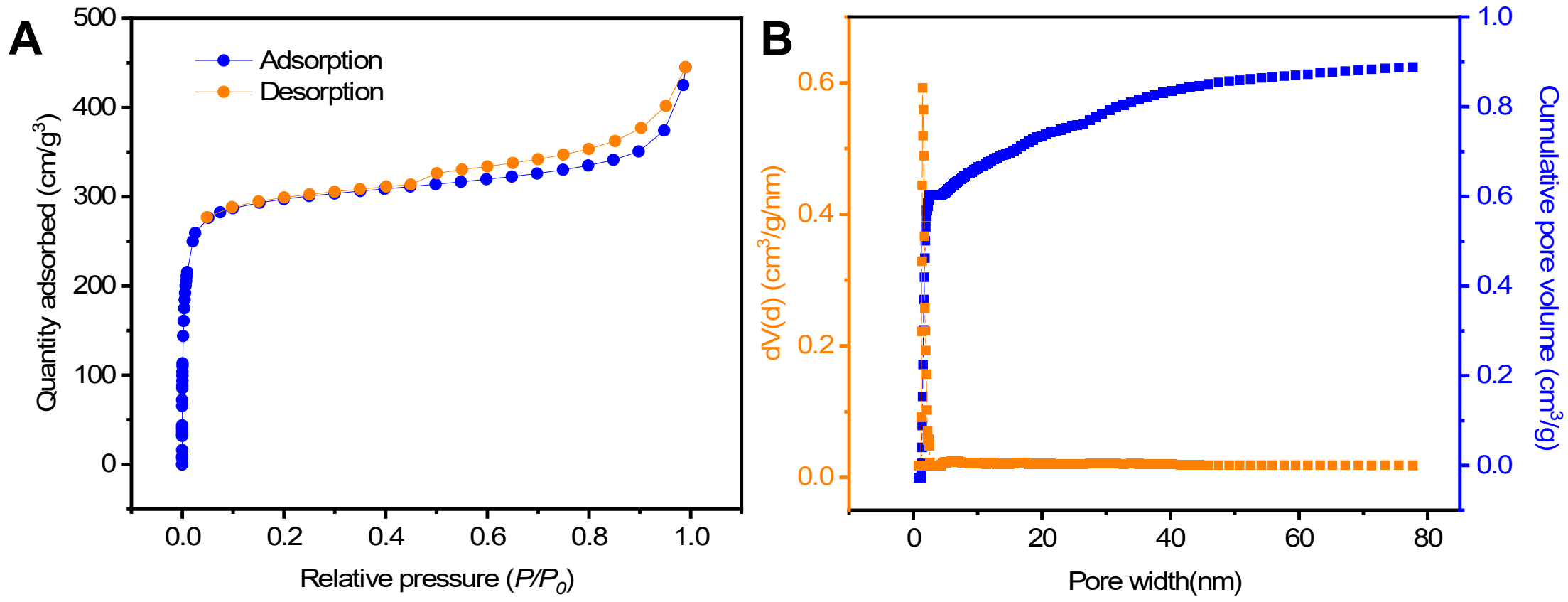

Supplementary Fig. 19. BET analysis of recycled NPy-Per-COF. (A) Nitrogen adsorption (blue line) and desorption (orange line) isotherm at 77 K of recycled NPy-Per-COF. (B) Pore size distribution of recycled NPy-Per-COF.

Supplementary Table 5. Surface area, pore volume and pore width of NPy-Per-COF before and after five consecutive photocatalytic cycles.

	Before reaction	After five photocatalytic cycles
Surface area (m^2/g)	1175	1123
Pore Volume (cm^3/g)	0.65	0.62
Pore width (nm)	1.43	1.43

Docking Results

Figure 6e was generated by Discovery Studio Visualizer and poses no copyright issues.

Supplementary Table 6. Summary of key protein-ligand interactions for the hit compounds and Sorafenib.

ID	Interacting residues	Interaction type	Distance (Å)	Rerank score (kcal/mol)
**4l**	ARG1027, ASP1046, ARG1027, ILE1044, GLU885, LYS868, ASP1046, ASP1046, CYS1045, VAL898, VAL899, LYS868, VAL899,VAL916	H-bond, H-bond, C–H bond, C–H bond, π-Cation, π-Anion, π-Anion, π-Sulfur, Alkyl, Alkyl, π-Alkyl, π-Alkyl, π-Alkyl	1.742, 2.092, 2.383, 2.995, 2.161, 4.231, 3.524, 4.194, 5.555, 3.485, 5.080, 5.074, 4.766, 4.391	-112.072
**4r**	ARG1027, ASP1046, ALA881, ILE1025, ASP814, ASP1046, ASP1046, HIS1026, ALA881, LEU889, VAL899, CYS1024, LEU889, LEU889, ILE892, VAL898, LEU1019	H-bond, H-bond, C–H bond, C–H bond, π-Anion, π-Anion, π-Anion, π-π T-shaped, π-π T-shaped, Alkyl, Alkyl, Alkyl, Alkyl, π-Alkyl, π-Alkyl, π-Alkyl, π-Alkyl	2.220, 2.237, 1.964, 1.585, 3.685, 4.603, 4.317, 5.322, 4.651, 3.719, 4.172, 4.306, 4.217, 5.272, 5.465, 5.036, 4.4189	-109.684
**4u**	CYS919, ASP1046, PHE918, VAL899, CYS1045, VAL848, ALA866, VAL899, VAL916, LEU1035, CYS1045, VAL848, ALA866, VAL899, VAL916, LEU1035, CYS1045, LEU840, LEU1035	H-bond, H-bond, π-π Stacked, Alkyl, Alkyl, π-Alkyl, π-Alkyl, π-Alkyl, π-Alkyl, π-Alkyl, π-Alkyl, π-Alkyl, π-Alkyl, π-Alkyl, π-Alkyl, π-Alkyl, π-Alkyl, π-Alkyl, π-Alkyl, π-Alkyl	2.147, 2.283, 5.362, 4.257, 4.748, 4.951, 5.395, 5.026, 5.184, 5.345, 4.264, 4.945, 3.790, 5.333, 5.177, 4.637, 5.436, 4.525, 5.385	-107.333
**5v**	ASP1046, ASP1046, LYS868, LYS868, CYS1045, CYS1045, PHE1047, LEU889, ILE892, LEU1019, ILE888, ILE892, CYS1024, HIS1026, LYS868, VAL899, VAL916, LYS868,	H-bond, H-bond, π-Cation, π-Cation, π-Sulfur, π-Sulfur, π-π T-shaped, Alkyl, Alkyl, Alkyl, Alkyl, Alkyl, Alkyl, π-Alkyl, π-Alkyl, π-Alkyl, π-Alkyl, π-Alkyl, π-Alkyl, π-Alkyl,	2.214, 2.667, 4.540, 4.641, 5.467, 4.738, 4.498, 5.100, 4.658, 4.845, 3.992, 5.285, 3.711, 5.297, 5.308, 4.492, 4.181, 4.112,	-105.763

	VAL914, VAL916, VAL848, ALA866, VAL899, VAL916	π-Alkyl, π-Alkyl, π-Alkyl, π-Alkyl	4.737, 3.641, 4.682, 5.250, 5.219, 5.112	
**7m**	LYS868, ASP1046, CYS817, CYS1024, SER884, GLU885, LEU1019, LEU889, ILE888	H-bond, H-bond, π-Sulfur, π-Sulfur, π-π T-shaped, π-Alkyl, π-Alkyl, π-Alkyl	2.800, 2.693, 5.130, 5.553, 5.217, 4.107, 5.307, 5.375, 4.790	-95.824
**Sorafenib**	LYS868, ASP1046, LYS868, ASP1046, LYS868, ALA866, VAL848, LYS868, VAL916, LEU889, VAL898, LEU1019, ILE1044, LYS868, VAL899, VAL916	H-bond, H-bond, π-Cation, π-Anion, π-Sigma, Alkyl, Alkyl, Alkyl, Alkyl, π-Alkyl, π-Alkyl, π-Alkyl, π-Alkyl, π-Alkyl, π-Alkyl, π-Alkyl	2.712, 2.44, 4.150, 4.895, 2.641, 3.490, 3.411, 4.670, 4.268, 5.438, 4.926, 5.136, 5.203, 4.505, 5.192, 4.034	-93.413

Supplementary Table 7. Docking scores of all the synthesized compounds against *VEGFR-2* (PDB ID: 4ASD)

ID	MolDock Score (kcal/mol)	Rerank Score (kcal/mol)	E-Inter (Protein-Ligand)	H-Bond Energy	Ligand Efficiency
**4a**	-97.144	-77.709	-117.948	-1.914	-4.416
**4b**	-111.454	-94.14	-133.75	-0.759	-4.644
**4c**	-107.284	-40.431	-128.999	0	-4.665
**4d**	-115.282	-79.673	-126.783	0	-4.803
**4e**	-125.851	-91.376	-135.304	-0.373	-4.84
**4f**	-125.307	-106.866	-148.188	-0.517	-4.641
**4g**	-105.13	-73.813	-124.458	0	-4.043
**4h**	-120.278	26.367	-144.137	-0.394	-4.009
**4i**	-102.077	-86.045	-125.298	-0.926	-4.64
**4j**	-112.229	-98.233	-135.854	0	-4.676
**4k**	-114.091	-89.519	-135.189	0	-4.96
**4l**	-130.149	-112.072	-150.086	-4.755	-5.006
**4m**	-108.879	-58.868	-131.464	-0.213	-4.734
**4n**	-106.161	-76.636	-120.614	0	-4.616

**4o**	-101.798	-83.268	-125.557	0	-4.426
**4p**	-120.618	-90.39	-146.822	0	-5.026
**4q**	-125.47	-48.777	-149.184	0	-4.327
**4r**	-149.508	-109.684	-153.122	-3.52	-4.531
**4s**	-140.039	-97.92	-153.737	0	-4.517
**4t**	-101.488	-88.455	-115.843	-2.748	-5.97
**4u**	-123.44	-107.333	-121.514	-4.241	-5.647
**4v**	-104.189	-88.238	-114.007	0	-5.788
**4w**	-105.907	-87.415	-114.406	0	-5.884
**4x**	-114.026	-95.729	-131.982	0	-5.183
**4y**	-109.663	-85.917	-120.835	-0.226	-4.985
**4z**	-104.445	-89.865	-117.978	-1.663	-5.803
**4aa**	-121.78	-40.554	-142.32	-0.588	-3.928
**4ab**	-141.739	-105.871	-170.333	-5.787	-3.543
**4ac**	-144.603	-86.098	-180.364	-0.016	-3.708
**5a**	-124.494	-103.917	-147.273	-1.477	-5.187
**5b**	-115.331	-91.496	-135.987	0	-5.014
**5c**	-116.03	-92.154	-138	-0.226	-5.045
**5d**	-126.363	-104.531	-149.978	-2.5	-5.265
**5e**	-107.821	-41.612	-132.469	0	-4.493
**5f**	-132.609	-93.03	-158.34	-2.5	-5.1
**5g**	-106.857	-81.223	-132.166	-2.448	-4.646
**5h**	-89.39	-75.804	-103.576	-2.5	-5.258
**5i**	-97.274	-7.849	-116.158	0	-4.632
**5j**	-111.484	-95.427	-134.153	0	-5.067
**5k**	-84.098	-61.132	-96.275	0	-4.672
**5l**	-102.508	-76.904	-119.578	0	-4.659
**5m**	-103.764	-81.593	-125.549	0	-4.324
**5n**	-102.533	-86.789	-125.197	-2.249	-4.272

**5o**	-116.173	-91.369	-136.074	0	-4.468
**5p**	-98.426	-77.605	-119.02	0	-4.279
**5q**	-117.296	-87.064	-144.831	0	-4.572
**5r**	-103.805	-81.576	-125.551	0	-4.325
**5s**	-84.705	-74.941	-114.552	0	-3.529
**5t**	-123.43	-39.491	-144.319	0	-4.747
**5u**	-114.111	-88.861	-138.946	-2.811	-4.755
**5v**	-135.23	-105.763	-154.25	-1.716	-5.201
**7a**	-98.385	-80.252	-118.223	-0.466	-5.178
**7b**	-106.081	-88.367	-128.632	0	-5.304
**7c**	-98.45	-84.314	-116.677	-1.633	-5.469
**7d**	-100.952	-89.643	-121.549	0	-5.608
**7e**	-104.309	-86.424	-123.084	-1.022	-5.49
**7f**	-107.361	-35.214	-119.676	-2.363	-5.112
**7g**	-115.988	-31.844	-123.538	0	-4.142
**7h**	-102.771	-57.883	-129.933	-1.021	-4.468
**7i**	-106.101	-71.281	-125.87	0	-4.081
**7j**	-102.366	-89.627	-123.263	0	-5.118
**7k**	-120.899	-98.427	-138.378	0	-5.495
**7l**	-113.964	-83.616	-123.708	0	-5.18
**7m**	-126.316	-95.824	-122.086	-2.070	-4.253
**7n**	-112.889	-96.608	-130.15	0	-4.516
**8**	-91.542	-63.201	-113.047	0	-3.98
**9**	-109.805	-89.75	-129.852	0	-4.575
**10**	-101.198	-80.939	-120.803	-0.08	-4.4
**11**	-73.683	-63.439	-88.971	-2.5	-4.912
**13**	-83.441	-65.679	-100.079	-3.46	-5.215
**Sorafenib**	-138.195	-93.413	-147.603	-3.821	-4.944

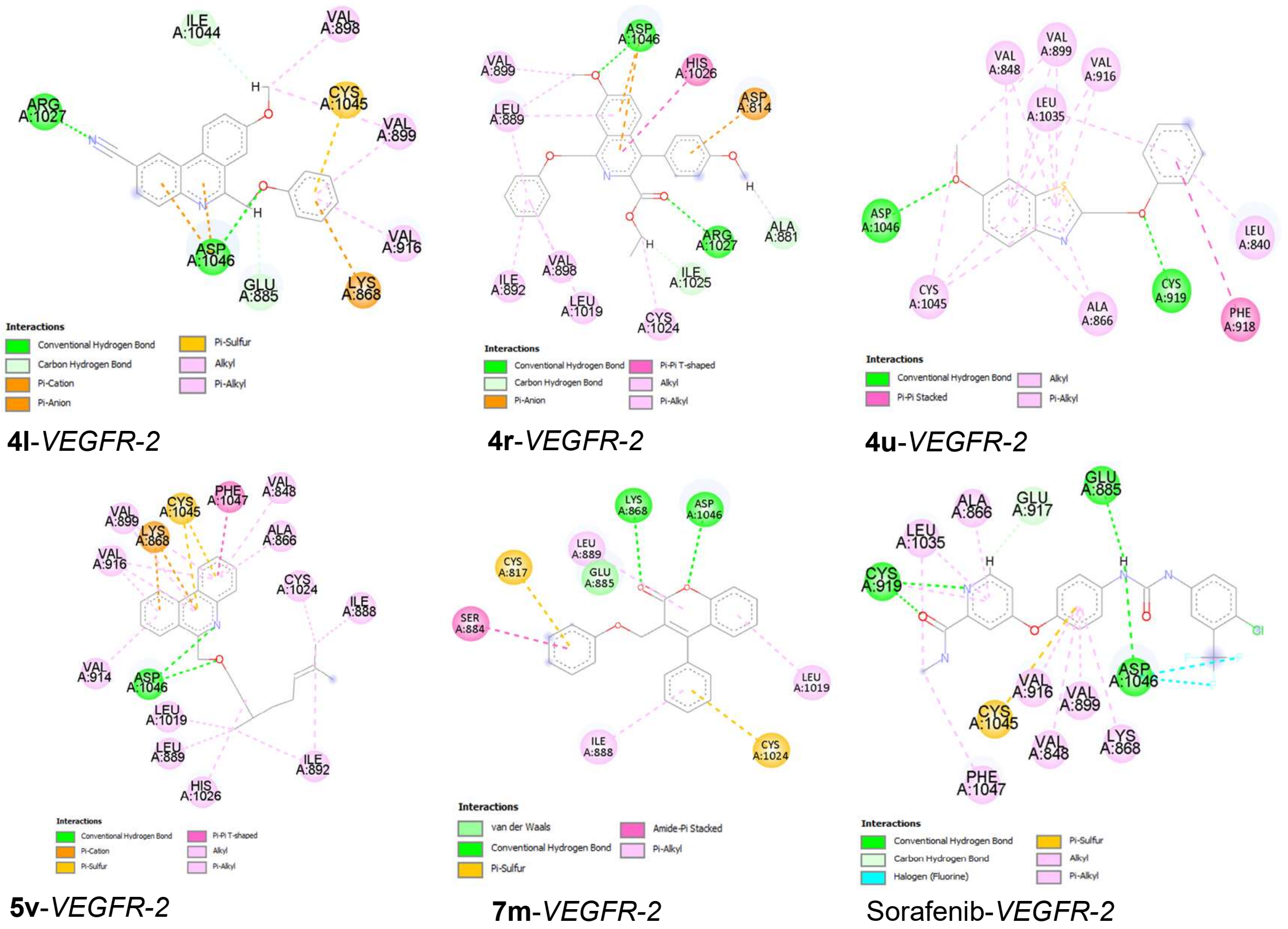

Supplementary Fig. 20. 2D interaction views of the docked poses of the hit compounds and the reference inhibitor (sorafenib) within the *VEGFR-2* (PDB ID: 4ASD) active site.

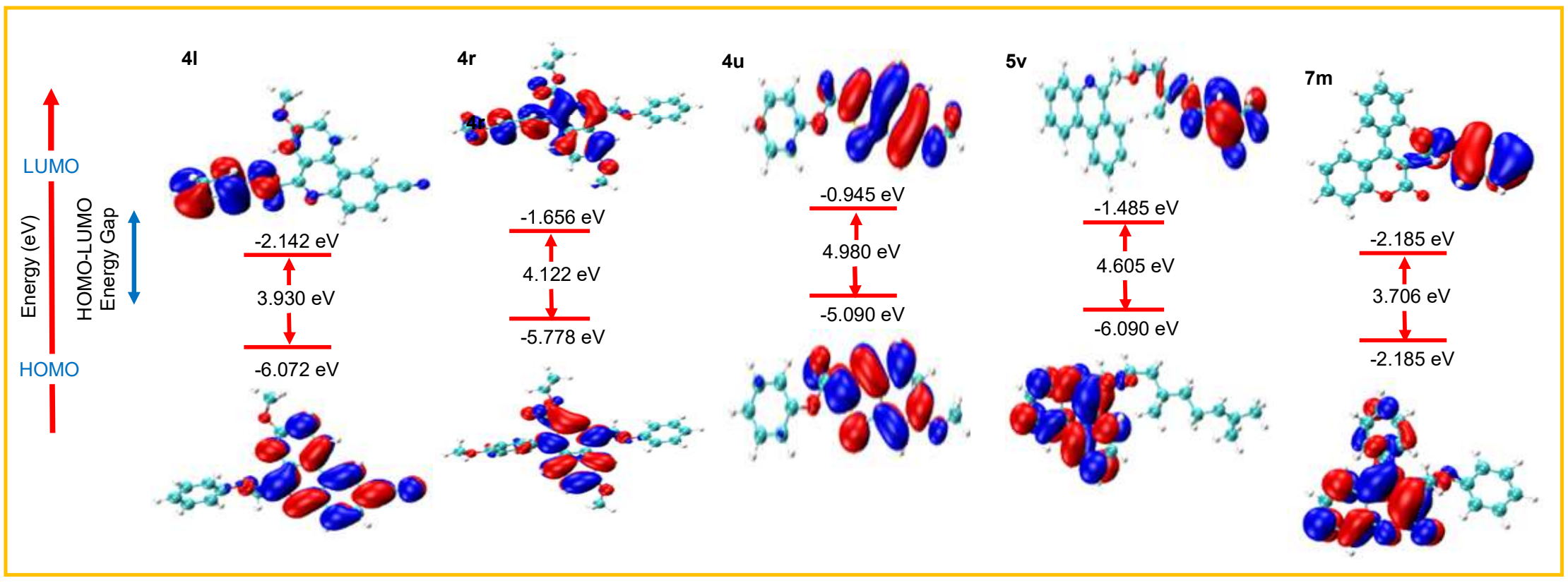

Supplementary Fig. 21. Frontier molecular orbitals (FMOs) and corresponding energy levels (HOMO and LUMO) for 4l, 4r, 4u, 5v and 7m.

Supplementary Table 8. Calculated global reactivity descriptors for 4l, 4r, 4u, 5v and 7m.

ID	HOMO (eV)	LUMO (eV)	ΔE (eV)	DM (Debye)	IP (eV)	EA (eV)	χ (eV)	η (eV)	σ (eV^{-1})	ω (eV)
**4l**	−6.072	−2.142	3.930	5.307	6.072	2.142	4.107	1.965	0.509	4.292
**4r**	−5.778	−1.656	4.122	3.335	5.778	1.656	3.717	2.061	0.485	3.352
**4u**	−5.925	−0.945	4.980	2.127	5.925	0.945	3.435	2.490	0.402	2.369
**5v**	−6.090	−1.485	4.605	2.143	6.090	1.485	3.788	2.302	0.434	3.115
**7m**	−5.891	−2.185	3.706	5.591	5.891	2.185	4.038	1.853	0.540	4.400

DM: dipole moment; IP: ionization potential; EA: electron affinity; χ: electronegativity; η: chemical hardness; σ: chemical softness; ω: electrophilicity.

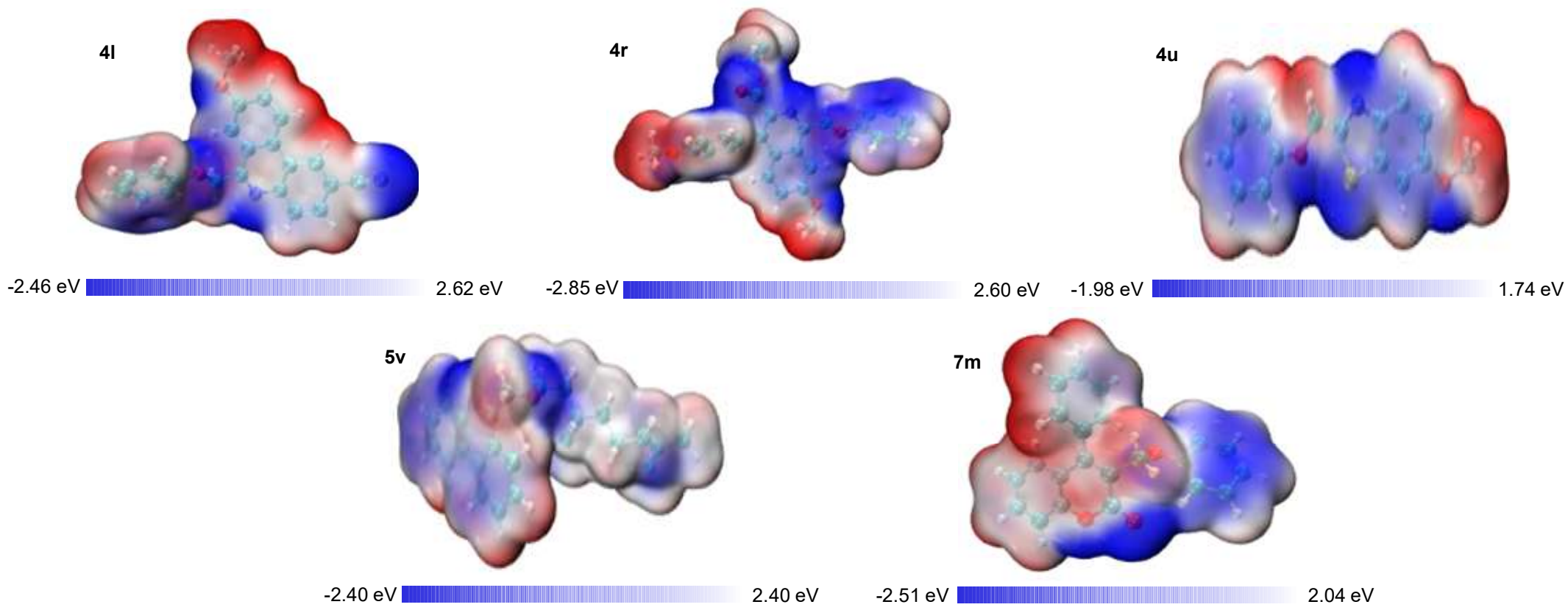

Supplementary Fig. 22. Molecular electrostatic potential (MEP) surfaces mapped over total electron density for the synthesized hit compounds.

Supplementary Table 9. Drug-likeness profiles of products.

ID	Lipinski Viol.	Ghose Viol.	Veber Viol.	Egan Viol.	Muegge Viol.	Bioavailability Score	PAINS Alerts	Synthetic Accessibility
**4a**	0	0	0	0	0	0.55	0	2.15
**4b**	0	0	0	0	0	0.55	0	2.37
**4c**	0	0	0	0	1	0.55	0	2.25
**4d**	0	0	0	0	0	0.55	0	2.44
**4e**	0	0	0	0	0	0.55	0	2.5
**4f**	0	0	0	0	1	0.55	0	2.57

**4g**	1	1	0	1	1	0.55	0	2.36
**4h**	1	1	0	1	1	0.55	0	2.58
**4i**	0	0	0	0	0	0.55	0	2.21
**4j**	0	0	0	0	0	0.55	0	2.34
**4k**	0	0	0	0	1	0.55	0	2.2
**4l**	0	0	0	0	0	0.55	0	2.59
**4m**	0	0	0	0	1	0.55	0	2.26
**4n**	0	0	0	0	1	0.55	0	2.24
**4o**	0	0	0	0	1	0.55	0	2.29
**4p**	0	0	0	0	1	0.55	0	2.52
**4q**	0	0	0	0	1	0.55	0	3.25
**4r**	0	0	0	0	1	0.55	0	3.56
**4s**	1	1	0	1	1	0.55	0	3.29
**4t**	0	0	0	0	0	0.55	0	2.54
**4u**	0	0	0	0	0	0.55	0	2.81
**4v**	0	0	0	0	0	0.55	0	2.68
**4w**	0	0	0	0	0	0.55	0	2.66
**4x**	0	1	0	1	1	0.55	0	2.88
**4y**	0	0	0	0	0	0.55	0	2.55
**4z**	0	0	0	0	0	0.55	0	2.63
**4aa**	0	0	0	0	1	0.55	0	3.13
**4ab**	1	4	0	1	1	0.55	0	3.77
**4ac**	2	3	0	1	1	0.17	0	3.95
**5a**	0	0	0	0	0	0.55	0	2.33
**5b**	1	0	0	0	1	0.55	0	2.26
**5c**	0	0	0	0	1	0.55	0	2.23
**5d**	0	0	0	0	0	0.55	0	2.44
**5e**	0	0	0	0	0	0.55	0	2.37
**5f**	1	1	0	1	1	0.55	0	2.36

**5g**	0	0	0	0	0	0.55	0	2.33
**5h**	0	0	0	0	0	0.55	0	1.74
**5i**	1	0	0	0	2	0.55	0	2.04
**5j**	1	0	0	0	2	0.55	0	2.2
**5k**	0	0	0	0	1	0.55	0	1.95
**5l**	0	0	0	0	1	0.55	0	1.96
**5m**	0	0	0	0	1	0.55	0	2.73
**5n**	0	0	0	0	0	0.55	0	2.91
**5o**	1	1	0	1	1	0.55	0	3.23
**5p**	0	0	0	0	0	0.55	0	2.84
**5q**	0	0	0	0	0	0.55	0	2.72
**5r**	0	0	0	0	1	0.55	0	2.38
**5s**	1	1	0	0	2	0.55	0	4.48
**5t**	1	1	0	1	1	0.55	0	3.92
**5u**	0	0	0	0	0	0.55	0	3.28
**5v**	1	1	0	1	1	0.55	0	3.54
**7a**	0	0	0	0	0	0.55	0	2.14
**7b**	0	0	0	0	0	0.55	0	2.31
**7c**	0	0	0	0	0	0.55	0	1.94
**7d**	0	0	0	0	0	0.55	0	2.15
**7e**	0	0	0	0	0	0.55	0	1.96
**7f**	0	0	0	0	0	0.55	0	2.69
**7g**	1	1	0	0	1	0.55	0	3.62
**7h**	0	0	0	0	0	0.55	0	2.93
**7i**	0	0	0	0	0	0.55	0	3.37
**7j**	0	0	0	0	0	0.55	0	3.05
**7k**	1	0	0	0	2	0.55	0	2.43
**7l**	0	0	0	0	0	0.55	0	3.46
**7m**	**0**	**0**	**0**	**0**	**0**	**0.55**	**0**	**3.43**

**7n**	0	0	0	0	1	0.55	0	3.21
**8**	0	0	0	0	0	0.55	1	2.43
**9**	1	0	0	0	1	0.55	0	3.4
**10**	1	0	0	0	1	0.55	0	3.19
**11**	0	0	0	0	2	0.55	0	2.71
**13**	0	0	0	0	0	0.55	0	1.41
**Sorafenib**	0	1	0	1	0	0.55	0	2.87

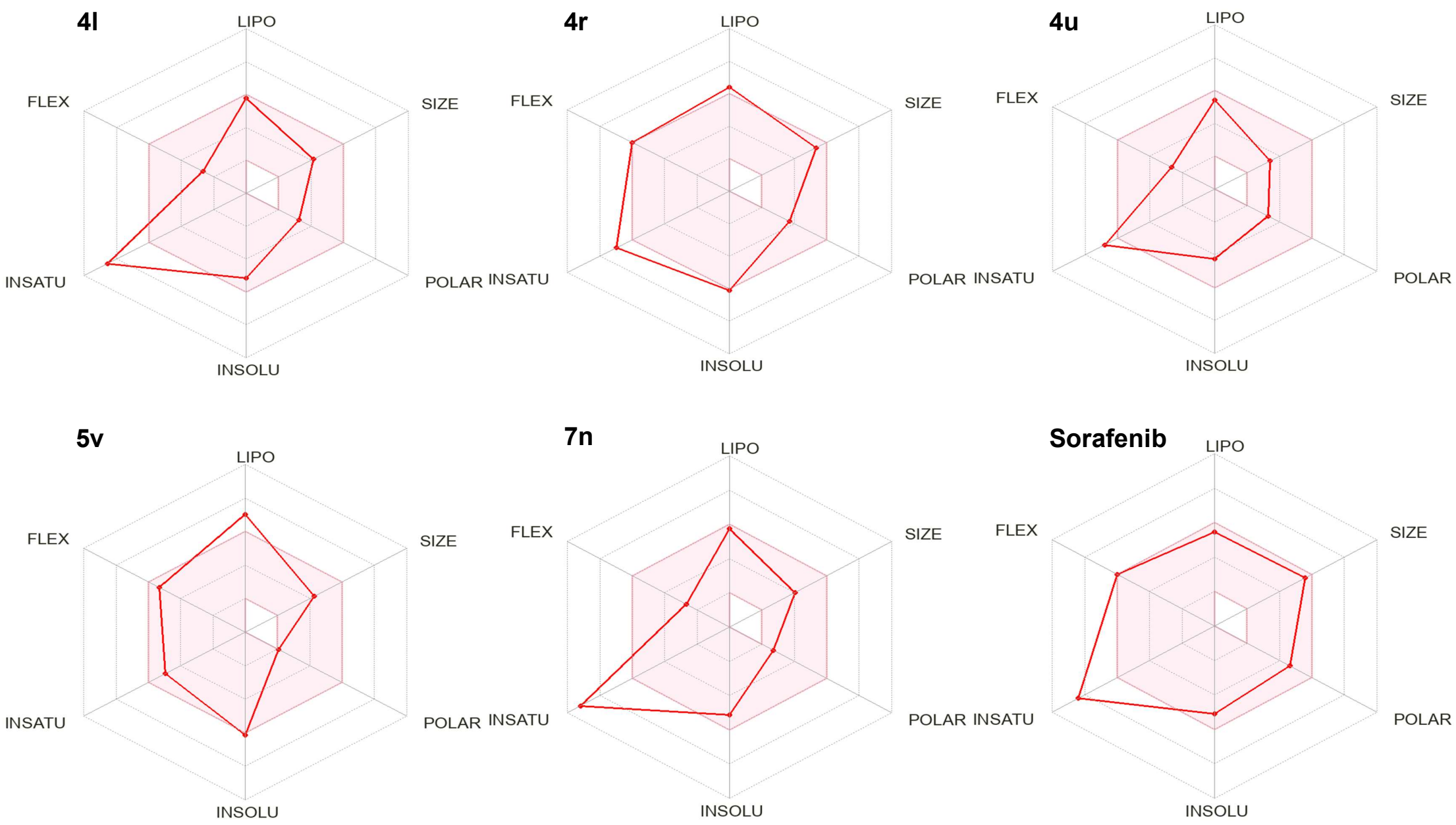

Supplementary Fig. 23. Radar plot of the major physicochemical properties for 4l, 4r, 4u, 5v, 7m and Sorafenib.

Supplementary Table 10. Computed pharmacokinetics and toxicity profiles of all products.

ID	Absorption	Distribution			Metabolism							Excretion	Toxicity
					Substrate		Inhibitor						
					CYP								
	Intestinal absorption	VDss (human)	BBB permeability	CNS permeability	2D6	3A4	1A2	2C19	2C9	2D6	3A4	Total clearance	AMES toxicity
	Numeric	Numeric	Numeric	Numeric	(yes/no)							Numeric (log	(yes/no)

	(%absorbed)	(log L kg^{-1})	(log BB)	(log PS)								mL min^{-1} kg^{-1})	
**4a**	97.825	-0.365	0.425	-0.732	No	Yes	Yes	Yes	No	No	No	0.723	No
**4b**	97.822	-0.481	0.369	-0.794	No	Yes	Yes	Yes	Yes	No	No	0.686	No
**4c**	96.248	0.008	0.377	-1.138	No	Yes	Yes	Yes	No	No	No	0.169	Yes
**4d**	98.072	-0.08	0.356	-1.156	No	Yes	Yes	Yes	Yes	No	No	0.809	Yes
**4e**	97.958	-0.666	0.176	-0.815	No	Yes	Yes	Yes	Yes	No	Yes	0.685	No
**4f**	97.855	-0.593	0.159	-0.853	No	Yes	Yes	Yes	Yes	No	Yes	0.741	No
**4g**	97.058	-0.452	0.405	-0.483	No	Yes	Yes	Yes	No	No	No	0.706	No
**4h**	96.29	-0.529	0.386	-0.233	No	Yes	Yes	No	No	No	No	0.754	No
**4i**	98.249	-0.543	0.325	-1.043	No	Yes	Yes	Yes	No	No	No	0.741	No
**4j**	97.822	-0.481	0.369	-0.794	No	Yes	Yes	Yes	Yes	No	No	0.685	No
**4k**	95.325	0.01	0.398	-1.091	No	Yes	Yes	Yes	Yes	No	No	0.169	Yes
**4l**	98.716	-0.228	0.084	-1.767	No	Yes	Yes	Yes	Yes	No	Yes	0.806	Yes
**4m**	95.562	0.02	0.412	-1.096	No	Yes	Yes	Yes	Yes	No	No	0.297	Yes
**4n**	95.859	0.021	0.367	-1.131	No	Yes	Yes	Yes	No	No	No	0.222	Yes
**4o**	94.458	-0.12	0.37	-1.09	No	Yes	Yes	Yes	Yes	No	No	0.353	Yes
**4p**	96.855	-0.051	0.464	-1.073	No	Yes	Yes	Yes	Yes	No	No	0.735	No
**4q**	95.207	-0.813	0.62	-1.227	No	Yes	No	Yes	Yes	No	Yes	0.936	No
**4r**	95.934	-0.971	-0.184	-3.162	No	Yes	No	Yes	Yes	No	Yes	0.845	No
**4s**	91.852	-0.735	0.604	-1.182	No	Yes	No	Yes	Yes	No	Yes	0.007	No
**4t**	94.722	0.023	0.216	-1.43	No	Yes	Yes	Yes	No	No	No	0.207	Yes
**4u**	95.178	-0.158	0.185	-1.482	No	Yes	Yes	Yes	No	No	No	0.302	No
**4v**	93.246	0.019	0.202	-1.379	No	Yes	Yes	Yes	No	No	No	0.142	No
**4w**	94.148	-0.151	0.253	-1.405	No	Yes	Yes	Yes	No	No	No	0.125	No
**4x**	91.698	-0.21	0.142	-1.44	No	Yes	Yes	Yes	Yes	No	No	0.19	No
**4y**	93.137	0.114	0.16	-1.375	No	Yes	Yes	Yes	No	No	No	0.143	Yes
**4z**	93.07	0.134	0.143	-1.375	No	Yes	Yes	Yes	No	No	No	0.121	Yes
**4aa**	97.951	-0.4	0.384	-2.044	No	Yes	Yes	Yes	Yes	No	Yes	0.796	Yes
**4ab**	100	-1.225	-0.914	-2.707	No	Yes	No	Yes	Yes	No	Yes	0.006	Yes

**4ac**	96.884	-1.04	0.083	-1.458	No	Yes	No	Yes	Yes	No	No	0.672	No
**5a**	97.822	-0.481	0.369	-0.794	No	Yes	Yes	Yes	Yes	No	No	0.676	No
**5b**	96.098	-0.373	0.349	-0.718	No	Yes	Yes	Yes	No	No	No	0.153	No
**5c**	97.067	-0.517	0.416	-0.745	No	Yes	Yes	Yes	No	No	No	0.543	No
**5d**	97.822	-0.481	0.369	-0.794	No	Yes	Yes	Yes	Yes	No	No	0.747	No
**5e**	95.797	-0.359	0.502	-1.191	No	Yes	Yes	Yes	Yes	No	No	0.768	No
**5f**	95.355	-0.38	0.431	-0.89	No	Yes	Yes	Yes	Yes	No	No	0.749	Yes
**5g**	95.076	-0.034	0.457	-1.124	No	Yes	Yes	Yes	Yes	No	No	0.827	No
**5h**	96.412	0.109	0.34	-1.503	No	Yes	Yes	Yes	Yes	Yes	No	0.925	Yes
**5i**	97.201	-0.225	0.778	-1.086	No	Yes	Yes	Yes	No	No	No	0.346	Yes
**5j**	96.262	-0.178	0.821	-1.016	No	Yes	Yes	Yes	No	No	No	0.353	Yes
**5k**	96.655	0.429	0.672	-1.416	No	Yes	Yes	Yes	Yes	No	No	0.436	Yes
**5l**	97.707	-0.484	0.487	-1.176	No	Yes	Yes	Yes	Yes	No	Yes	0.739	Yes
**5m**	96.07	-0.302	0.447	-1.163	No	Yes	Yes	Yes	Yes	No	No	0.788	Yes
**5n**	94.828	-0.437	0.36	-1.518	No	Yes	Yes	Yes	Yes	No	Yes	0.711	No
**5o**	95.527	-0.295	0.636	-1.227	No	Yes	Yes	Yes	Yes	No	No	0.864	Yes
**5p**	97.406	-0.507	0.516	-1.256	No	Yes	Yes	Yes	Yes	No	No	0.899	Yes
**5q**	92.006	0.349	0.329	-0.833	No	Yes	Yes	Yes	Yes	No	Yes	0.596	Yes
**5r**	96.174	-0.272	0.449	-1.149	No	Yes	Yes	Yes	Yes	No	No	0.776	Yes
**5s**	97.823	0.374	0.599	-0.695	No	Yes	Yes	Yes	No	No	No	0.51	No
**5t**	96.718	0.215	0.412	-0.855	No	Yes	Yes	Yes	No	No	No	0.823	No
**5u**	96.398	-0.415	0.305	-1.175	No	Yes	Yes	Yes	Yes	No	No	0.646	No
**5v**	93.076	0.746	0.618	-1.316	No	Yes	Yes	Yes	Yes	No	No	1.229	Yes
**7a**	96.424	0.087	0.305	-1.333	No	Yes	Yes	Yes	Yes	No	No	0.731	Yes
**7b**	96.382	0.206	0.314	-1.306	No	Yes	Yes	Yes	No	No	No	0.221	No
**7c**	98.593	-0.258	0.436	-1.116	No	No	Yes	Yes	No	No	No	0.224	No
**7d**	99.015	-0.504	0.288	-1.159	No	No	Yes	Yes	No	No	No	0.122	No
**7e**	98.391	-0.239	0.431	-1.035	No	No	Yes	Yes	No	No	No	0.295	No
**7f**	100	-0.043	0.463	-1.041	No	Yes	Yes	Yes	No	No	No	0.167	No

**7g**	98.069	-0.273	0.396	-0.682	No	Yes	Yes	Yes	Yes	No	No	0.194	No
**7h**	97.863	-0.202	0.168	-1.327	No	Yes	Yes	Yes	No	No	No	0.147	No
**7i**	99.935	-0.255	0.487	-0.624	No	Yes	Yes	Yes	Yes	No	No	0.201	No
**7j**	99.664	-0.195	0.275	-1.089	No	No	Yes	Yes	No	No	No	0.032	No
**7k**	98.284	-0.06	0.862	-0.566	No	Yes	Yes	Yes	No	No	No	0.189	No
**7l**	100	0.023	0.435	-0.955	No	Yes	Yes	Yes	No	No	No	0.055	No
**7m**	96.147	-0.396	0.274	-0.982	No	Yes	Yes	Yes	Yes	No	No	0.806	No
**7n**	97.261	-0.498	0.404	-0.46	No	Yes	Yes	Yes	Yes	No	No	0.036	No
**8**	99.046	0.185	0.334	-0.711	No	Yes	Yes	No	No	No	No	0.996	No
**9**	93.446	-0.08	0.393	-0.962	No	Yes	Yes	Yes	No	No	No	0.069	No
**10**	93.342	-0.109	0.39	-0.976	No	Yes	Yes	Yes	No	No	No	0.014	No
**11**	95.117	0.172	0.537	-1.242	No	No	Yes	No	No	No	No	0.282	No
**13**	96.698	-0.266	0.363	-1.339	No	No	Yes	No	No	No	No	0.727	Yes
**Sorafenib**	85.494	-0.009	-1.473	-2.025	No	Yes	No	Yes	Yes	No	Yes	-0.213	No

Radical prevention experiment

Under a nitrogen atmosphere, NPy-Per-COF (14.1 mg, 0.01 mmol based on the repeating unit), **2a** (132.0 mg, 0.4 mmol), **3a** (35.8 mg, 0.2 mmol), TEMPO (93.6 mg, 0.6 mmol) and DABCO (44.8 mg, 0.4 mmol) were mixed in $CHCl_3$ (4.0 mL) in a Schlenk tube. The resulting mixture was stirred under blue LED irradiation (420 nm) at room temperature for 48-96 hours. Upon completion, the residual was filtered through celite pad and filtrate was concentrated. TLC and crude NMR results indicated that the yield of **4a** is 0%. A TEMPO-alkyl radical adduct was detected by high-resolution mass spectrometry of the mixture.

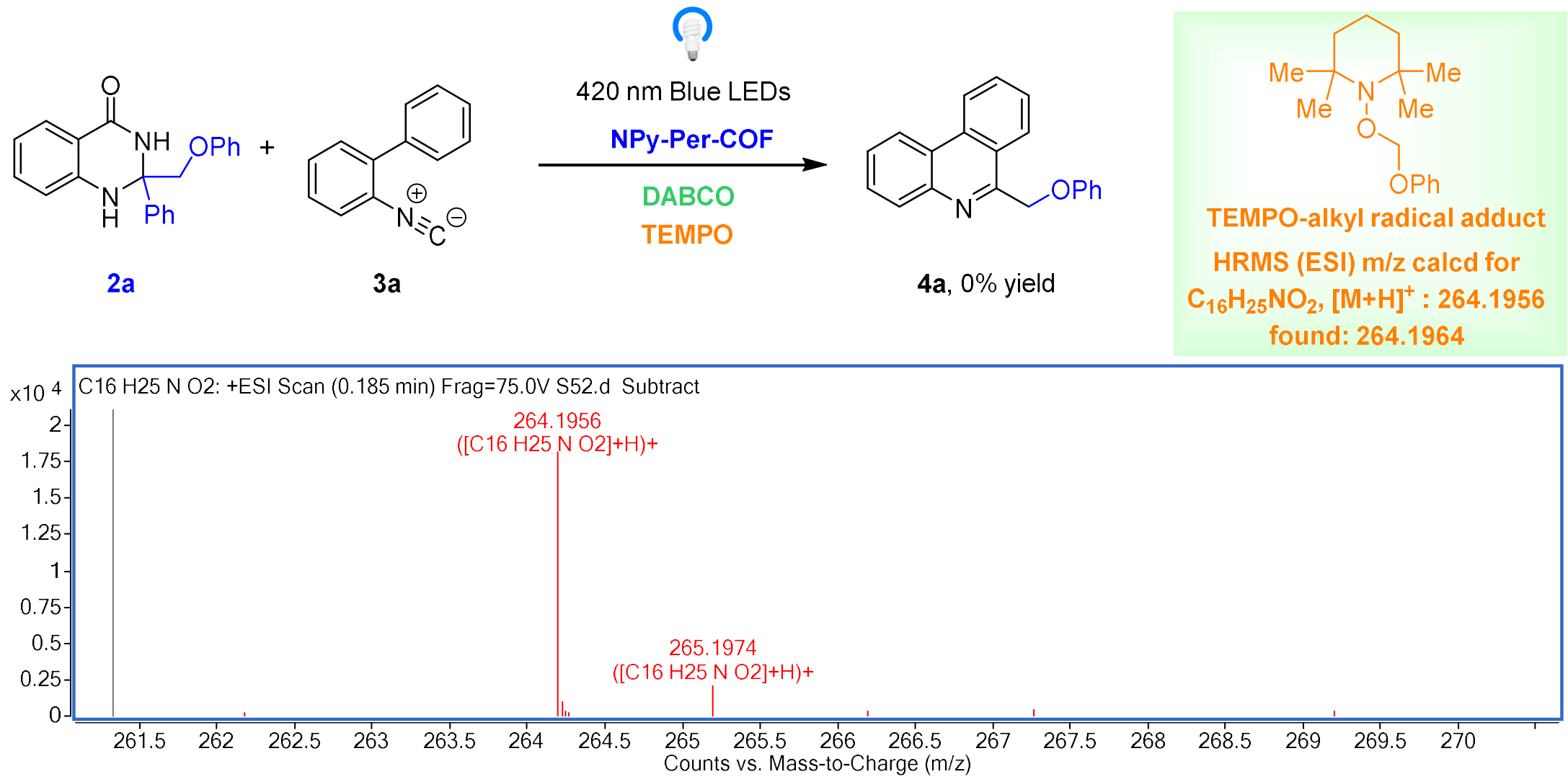

Supplementary Fig. 24. HRMS spectrum analysis of reaction in the presence of TEMPO. TEMPO is 2,2,6,6-tetramethylpiperidine 1-oxide.

EPR analysis

To gain more insight into the possible radical intermediates, we carried out EPR studies (X band, 9.8 GHz) by using 2,2-dimethyl-3,4-dihydro-2H-pyrrole 1-oxide (DMPO) as free radical spin-trapping agent. A mixture of **2a** (66.0 mg, 0.2 mmol), **3b** (17.9 mg, 0.1 mmol), Cs_2CO_3 (65.2 mg, 0.2 mmol) and DMPO (0.1 mmol) in 1 mL anhydrous $CHCl_3$ was prepared, then 20 uL of the mixture was transferred to a capillary tube, the tube was charged into a EPR tube and then the mixture was measured *in-situ*. The mixture was irradiated with 420 nm blue LEDs for 2 min, a signal was recorded, a carbon-centered radical adduct DMPO-**2a** (g = 2.00168, A_N = 15.2 G, A_H = 22.3 G). Measurement conditions: Frequency 9.8 GHz; Power 20 mW; Center field 3500 G; Sweep width 100 G; Modulation width 1 G; amplitude 1×1000; Sweep time 1 min; Time constant 1 s.

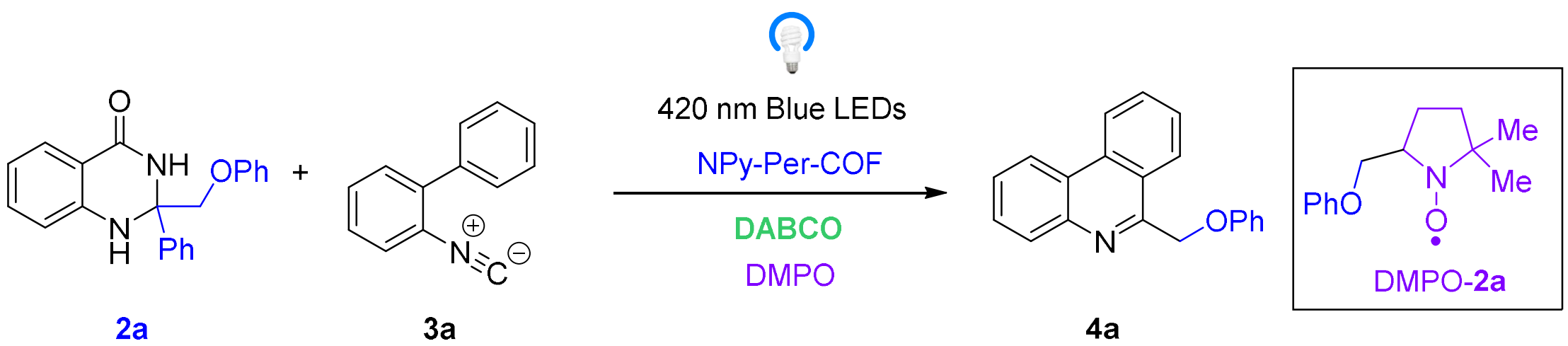

Supplementary Fig. 25. EPR studies.

KIE experiments

Under a nitrogen atmosphere, NPy-Per-COF (14.1 mg, 0.01 mmol based on the repeating unit), **2a** (0.4 mmol), **3a** (17.9 mg, 0.1 mmol), d_5-**3a** (18.4 mg, 0.1 mmol) and DABCO (44.8 mg, 0.4 mmol) were mixed in $CHCl_3$ (4.0 mL) in a Schlenk tube. The resulting mixture was stirred under blue LED irradiation (420 nm) at room temperature for 10 hours. Upon completion, the residual was filtered through celite pad and filtrate was concentrated. The mixture was purified by silica gel flash chromatography (petroleum ether : ethyl acetate, 10 : 1) to afford the mixtures **4a** and d_5-**4a**.

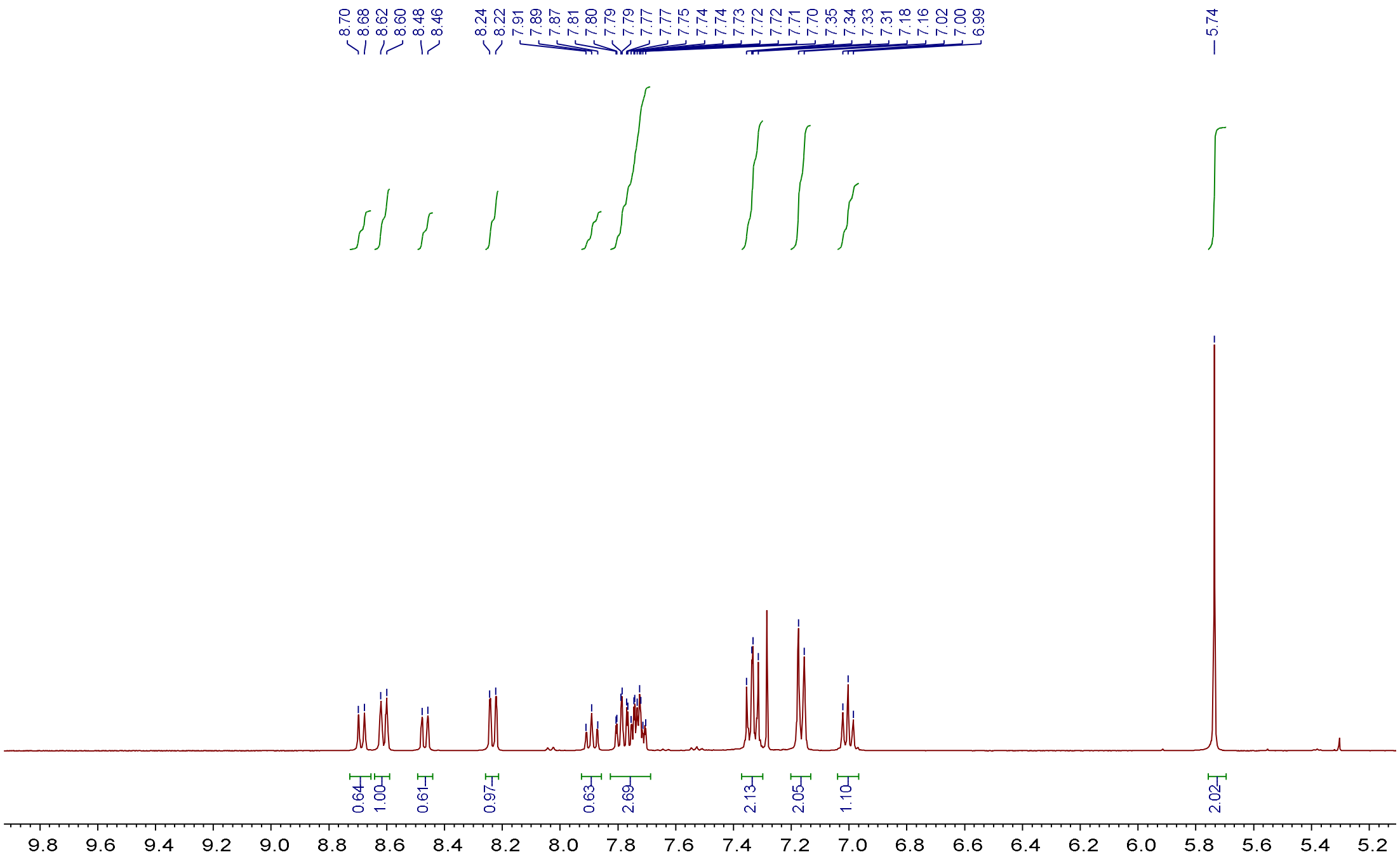

Supplementary Fig. 26. ^{1}H NMR spectrum of KIE experiments.

Stern–Volmer fluorescence quenching studies

Stern–Volmer experiments for all the components of the reaction mixture were carried out to monitor the emission intensity of solutions of NPy-Per-COF (0.5 μ M) containing variable amounts of the quencher in $CHCl_3$. The emission intensity at 465 nm was collected with excited wavelength of 329 nm in $CHCl_3$ using a LS55 spectrophotometer (Perkin-Elmer, USA). After degassing the sample with a stream of nitrogen for 10 minutes, the emission intensity of the sample was collected and plots were constructed according to the Stern-Volmer equation $I_0/I = 1 + K_q t_0[Q]$.

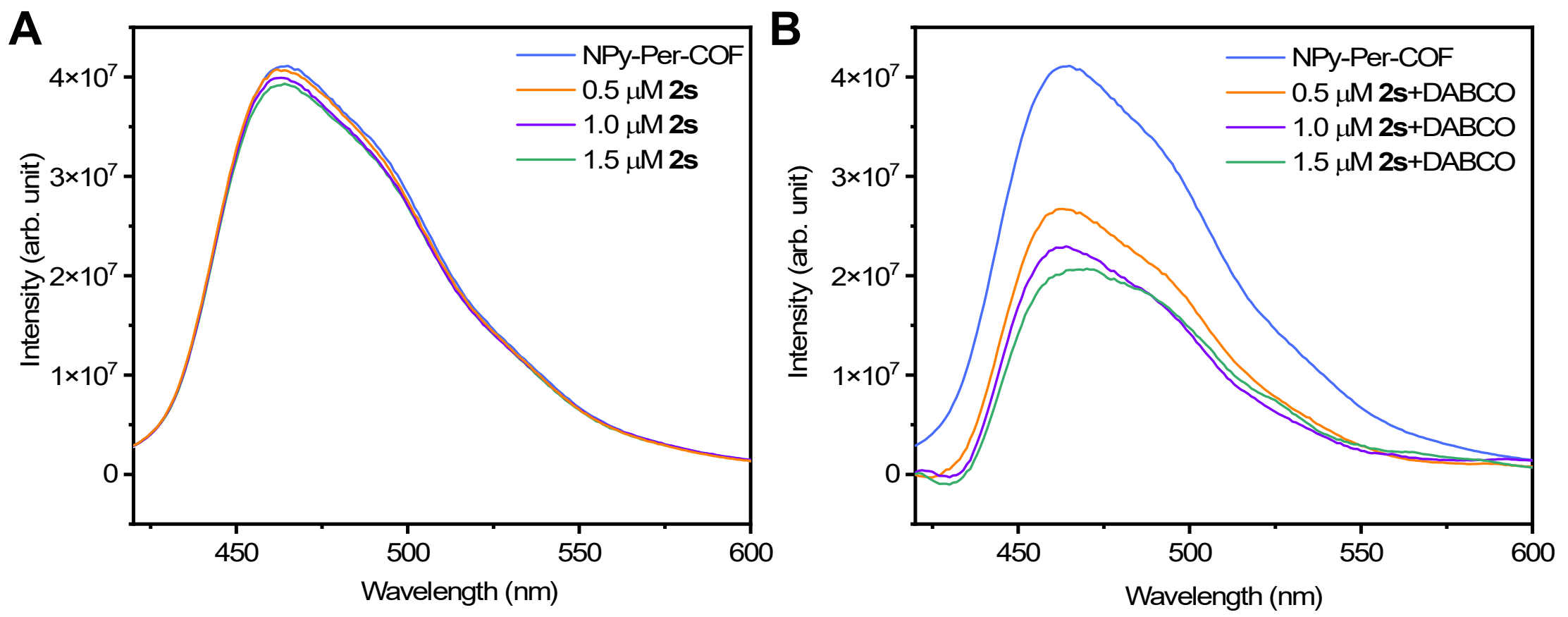

Supplementary Fig. 27. Fluorescence quenching experiments. (A) Fluorescence quenching of NPy-Per-COF by **2s** in anhydrous $CHCl_3$. (B) Fluorescence quenching of NPy-Per-COF by [**2s**+DABCO] mixture in anhydrous $CHCl_3$.

UV–vis spectra of NPy-Per-COF/$CHCl_3$ suspension

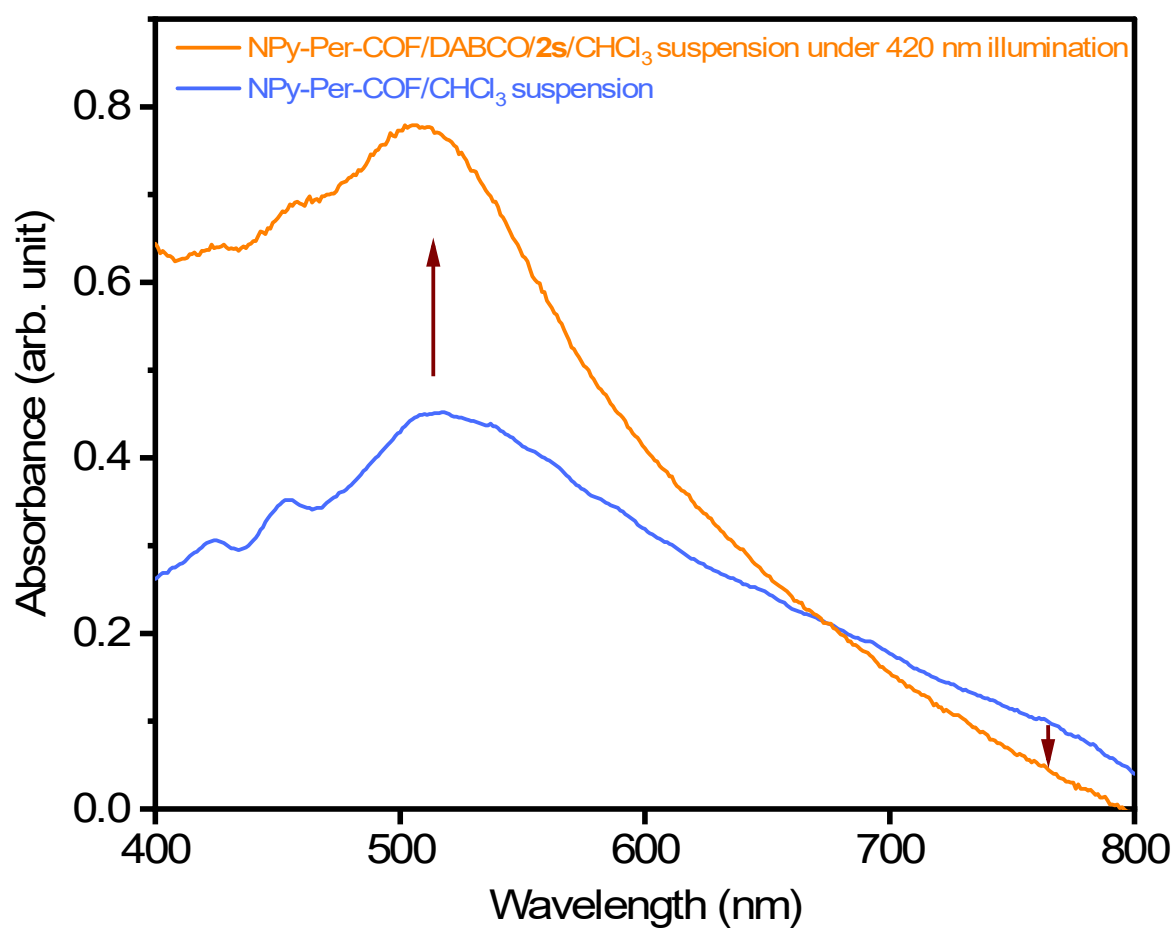

Supplementary Fig. 28. UV-vis spectra of NPy-Per-COF/$CHCl_3$ suspension. 1 mg NPy-Per-COF suspended in 3 mL of $CHCl_3$ (Blue line). 1 mg NPy-Per-COF, 0.05 mmol DABCO and 0.02 mmol **2s** suspended in 3 mL of $CHCl_3$ under 420 nm LED irradiation at N_2 atmosphere for 10 min (Orange line).

fs-TA spectra

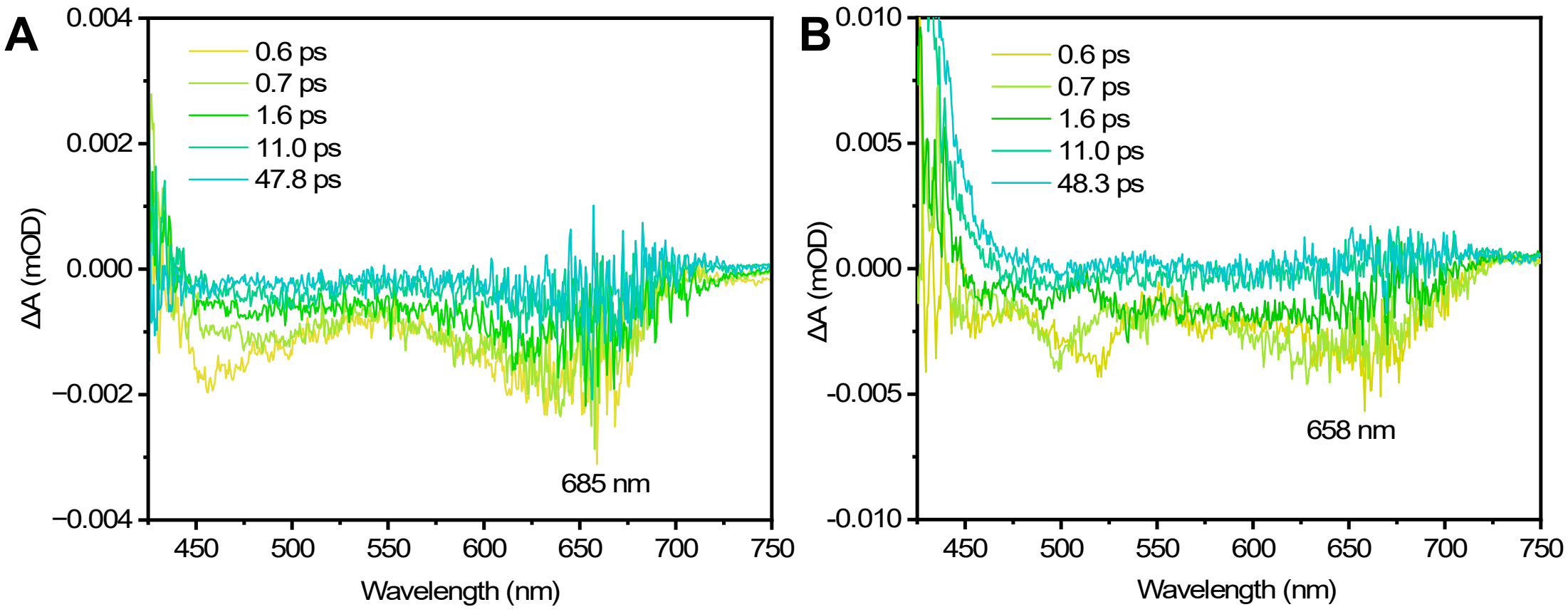

Supplementary Fig. 29. fs-TA spectra. (a) fs-TA spectra of NPy-Per-COF/$CHCl_3$ suspension. (b) fs-TA spectra of NPy-Per-COF/$CHCl_3$ suspension with **2s** and DABCO. λ_{exc} = 400 nm

X-ray crystallographic data of 7h

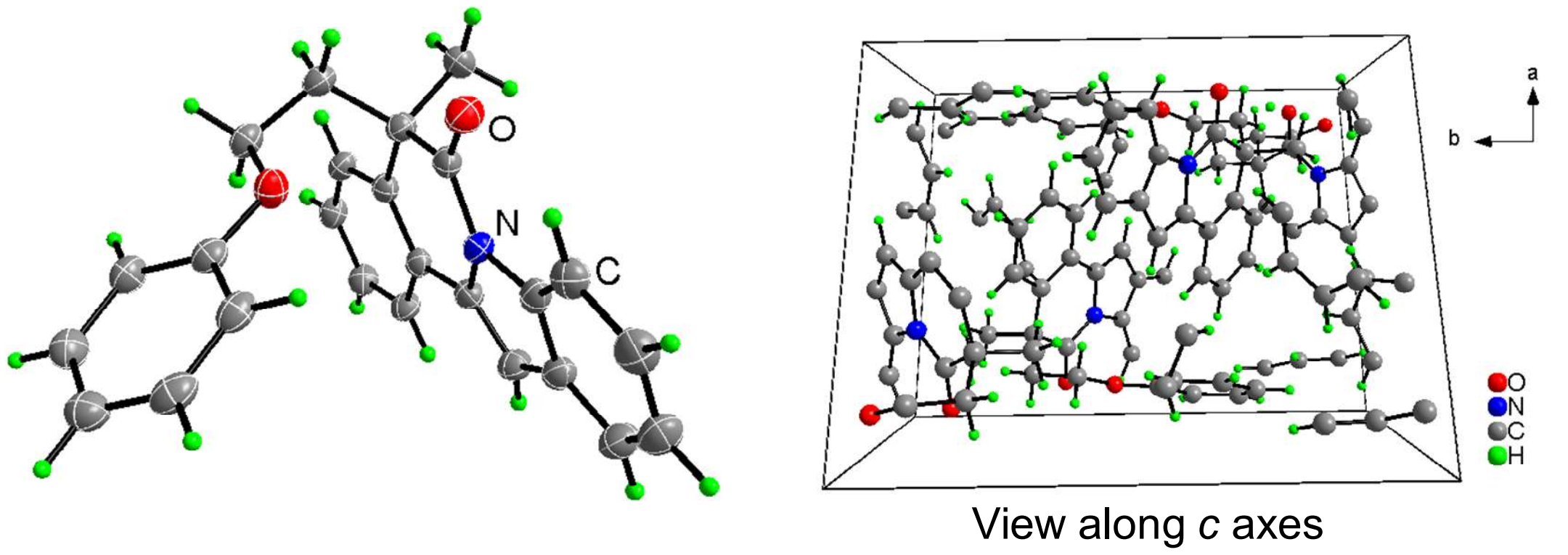

Supplementary Fig. 30. X-ray crystallography of 7h. CCDC: 2504819.

Supplementary Table 11. Crystal data and structure refinement for 7h.

Identification code	**7h**
Empirical formula	$C_{25}H_{21}NO_2$
Formula weight	367.43
Temperature/K	99.99(10)
Crystal system	monoclinic
Space group	$P2_1/c$

a/Å	11.9811(6)
b/Å	14.6199(5)
c/Å	11.4363(6)
α/°	90
β/°	113.005(6)
γ/°	90
Volume/$Å^3$	1843.89(16)
Z	4
ρ_{calc}g/cm^3	1.324
μ/mm^{-1}	0.084
F(000)	776.0
Crystal size/mm^3	0.1 × 0.09 × 0.09
Radiation	MoKα (λ = 0.71073)
2Θ range for data collection/°	3.694 to 60.448
Index ranges	-14 ≤ h ≤ 13, -14 ≤ k ≤ 19, -14 ≤ l ≤ 13
Reflections collected	15148
Independent reflections	4488 [R_{int} = 0.0347, R_{sigma} = 0.0400]
Data/restraints/parameters	4488/0/254
Goodness-of-fit on F^2	1.057
Final R indexes [I>=2σ (I)]	R_1 = 0.0444, wR_2 = 0.1005
Final R indexes [all data]	R_1 = 0.0655, wR_2 = 0.1101
Largest diff. peak/hole / e $Å^{-3}$	0.25/-0.24

Supplementary Table 12. Fractional atomic coordinates (×10^4) and equivalent isotropic displacement parameters (Å^2×10^3) for 7h. U_{eq} is defined as 1/3 of the trace of the orthogonalised U_{IJ} tensor.

Atom	*x*	*y*	*z*	U(eq)
O(1)	8679.3(9)	4494.2(6)	5859.8(9)	25.0(2)
O(2)	8864.2(9)	3415.6(7)	8165.6(9)	27.3(2)
N(1)	6995.6(10)	4043.4(7)	7522.4(11)	20.2(2)
C(1)	5339.4(12)	3605.5(9)	5501.9(13)	20.2(3)
C(2)	6134.7(12)	3024.0(9)	5228.4(13)	20.1(3)
C(3)	7841.1(13)	3461.4(9)	7362.6(13)	20.7(3)
C(4)	7189.4(13)	4606.2(9)	8587.3(13)	21.2(3)
C(5)	5760.1(12)	4091.9(9)	6705.6(13)	20.4(3)
C(6)	8369.0(13)	2890.8(9)	5597.5(14)	22.8(3)
C(7)	4536.6(13)	2690.7(10)	3182.1(14)	25.4(3)
C(8)	4151.5(13)	3717.2(9)	4613.6(14)	24.4(3)
C(9)	7409.0(12)	2837.9(9)	6192.3(13)	21.1(3)
C(10)	8373.1(13)	3780.0(9)	4927.6(13)	22.5(3)
C(11)	8696.8(12)	5369.9(9)	5441.4(14)	22.2(3)
C(12)	6051.1(13)	4975.9(9)	8427.5(14)	24.2(3)
C(13)	5179.3(13)	4641.3(9)	7243.6(14)	24.7(3)
C(14)	9009.8(12)	6042.5(10)	6386.1(14)	24.2(3)
C(15)	5703.4(13)	2566.6(9)	4058.6(14)	24.1(3)
C(16)	8429.7(13)	6532.2(10)	3876.4(15)	26.6(3)
C(17)	8244.5(13)	4813.0(9)	9628.2(14)	25.5(3)
C(18)	7436.9(14)	1857.4(9)	6718.3(14)	26.1(3)
C(19)	8417.3(12)	5613.4(9)	4183.6(14)	24.5(3)
C(20)	3753.9(13)	3270.5(10)	3460.3(14)	26.2(3)
C(21)	9012.9(13)	6951.6(10)	6054.3(15)	27.3(3)

C(22)	8136.4(15)	5410.8(10)	10525.7(15)	29.7(3)
C(23)	7018.4(15)	5779.8(10)	10385.9(15)	32.2(4)
C(24)	8718.9(13)	7203.1(10)	4797.8(15)	29.0(3)
C(25)	5977.2(15)	5571.0(10)	9350.5(15)	30.5(3)

Supplementary Table 13. Anisotropic displacement parameters (Å^2×10^3) for 7h. The anisotropic displacement factor exponent takes the form: $-2\pi^2[h^2a^{*2}U_{11}+2hka^*b^*U_{12}+\ldots]$.

Atom	U_{11}	U_{22}	U_{33}	U_{23}	U_{13}	U_{12}
O(1)	28.6(6)	25.7(5)	21.9(5)	-2.6(4)	11.2(5)	-2.7(4)
O(2)	22.2(6)	35.4(6)	21.5(5)	-1.1(4)	5.4(5)	4.8(4)
N(1)	19.1(6)	21.7(6)	20.8(6)	-1.0(4)	8.9(5)	-0.2(4)
C(1)	20.0(7)	21.8(7)	20.4(7)	1.6(5)	9.7(6)	-1.5(5)
C(2)	19.5(7)	20.4(7)	21.7(7)	2.2(5)	9.3(6)	-0.3(5)
C(3)	20.0(7)	24.3(7)	19.3(7)	3.3(5)	9.2(6)	1.6(5)
C(4)	25.6(8)	19.1(7)	21.6(7)	-0.3(5)	12.0(6)	-2.1(5)
C(5)	18.3(7)	21.5(7)	21.7(7)	2.0(5)	8.1(6)	0.4(5)
C(6)	19.3(7)	26.5(7)	23.7(7)	-2.3(5)	9.5(6)	2.4(5)
C(7)	24.6(8)	29.6(8)	21.1(7)	-3.2(5)	8.0(6)	-3.4(5)
C(8)	19.6(7)	29.1(7)	25.6(8)	0.2(6)	10.1(6)	1.7(5)
C(9)	19.9(7)	22.9(7)	21.3(7)	-0.6(5)	8.7(6)	1.9(5)
C(10)	20.1(7)	27.3(7)	20.6(7)	-4.6(5)	8.3(6)	-2.1(5)
C(11)	15.6(7)	26.3(7)	25.1(8)	-1.8(5)	8.4(6)	-0.7(5)
C(12)	26.8(8)	22.1(7)	25.4(8)	-1.0(5)	12.0(6)	0.0(5)
C(13)	22.0(7)	26.8(7)	25.6(8)	-0.2(5)	9.6(6)	2.6(5)
C(14)	16.7(7)	33.7(8)	22.4(8)	-3.9(6)	7.7(6)	0.1(5)
C(15)	25.2(8)	25.1(7)	25.0(8)	-2.9(5)	13.1(6)	0.6(5)
C(16)	20.6(8)	32.0(8)	25.0(8)	1.9(6)	6.7(6)	0.4(5)
C(17)	25.4(8)	25.2(7)	25.9(8)	-0.5(5)	10.1(6)	-2.9(5)

C(18)	27.2(8)	25.5(7)	25.1(8)	2.4(5)	9.8(7)	2.4(5)
C(19)	20.0(7)	29.4(8)	23.5(8)	-3.6(5)	7.8(6)	-1.9(5)
C(20)	18.1(7)	33.4(8)	24.3(8)	0.7(6)	5.1(6)	-1.6(5)
C(21)	18.9(8)	31.1(8)	32.8(9)	-9.4(6)	11.0(7)	-2.1(5)
C(22)	33.7(9)	25.9(8)	26.5(8)	-3.0(6)	8.4(7)	-5.6(6)
C(23)	41.0(10)	27.2(8)	29.4(8)	-7.7(6)	14.9(8)	-0.2(6)
C(24)	23.4(8)	26.4(8)	37.2(9)	-0.7(6)	11.9(7)	0.1(5)
C(25)	33.2(9)	29.0(8)	31.5(9)	-3.6(6)	15.0(7)	4.8(6)

Supplementary Table 14. Bond lengths for 7h.

Atom	Atom	Length/Å	Atom	Atom	Length/Å
O(1)	C(10)	1.4338(16)	C(6)	C(10)	1.5099(19)
O(1)	C(11)	1.3699(17)	C(7)	C(15)	1.377(2)
O(2)	C(3)	1.2127(17)	C(7)	C(20)	1.390(2)
N(1)	C(3)	1.3886(17)	C(8)	C(20)	1.379(2)
N(1)	C(4)	1.4119(17)	C(9)	C(18)	1.5499(18)
N(1)	C(5)	1.4100(17)	C(11)	C(14)	1.3993(19)
C(1)	C(2)	1.4006(18)	C(11)	C(19)	1.390(2)
C(1)	C(5)	1.4534(19)	C(12)	C(13)	1.435(2)
C(1)	C(8)	1.398(2)	C(12)	C(25)	1.3971(19)
C(2)	C(9)	1.5181(19)	C(14)	C(21)	1.383(2)
C(2)	C(15)	1.4013(19)	C(16)	C(19)	1.390(2)
C(3)	C(9)	1.5326(19)	C(16)	C(24)	1.381(2)
C(4)	C(12)	1.411(2)	C(17)	C(22)	1.392(2)
C(4)	C(17)	1.389(2)	C(21)	C(24)	1.389(2)
C(5)	C(13)	1.3575(19)	C(22)	C(23)	1.394(2)
C(6)	C(9)	1.5501(18)	C(23)	C(25)	1.377(2)

Supplementary Table 15. Bond Angles for 7h.

Atom	Atom	Atom	Angle/°	Atom	Atom	Atom	Angle/°
C(11)	O(1)	C(10)	117.09(11)	C(2)	C(9)	C(18)	108.09(11)
C(3)	N(1)	C(4)	126.03(12)	C(3)	C(9)	C(6)	107.35(11)
C(3)	N(1)	C(5)	125.07(11)	C(3)	C(9)	C(18)	105.22(11)
C(5)	N(1)	C(4)	108.56(10)	C(18)	C(9)	C(6)	108.02(11)
C(2)	C(1)	C(5)	119.61(12)	O(1)	C(10)	C(6)	107.48(11)
C(8)	C(1)	C(2)	119.80(12)	O(1)	C(11)	C(14)	115.03(12)
C(8)	C(1)	C(5)	120.59(12)	O(1)	C(11)	C(19)	124.79(12)
C(1)	C(2)	C(9)	121.71(12)	C(19)	C(11)	C(14)	120.17(13)
C(1)	C(2)	C(15)	118.27(13)	C(4)	C(12)	C(13)	107.58(12)
C(15)	C(2)	C(9)	119.91(12)	C(25)	C(12)	C(4)	118.95(14)
O(2)	C(3)	N(1)	120.51(12)	C(25)	C(12)	C(13)	133.44(14)
O(2)	C(3)	C(9)	121.67(12)	C(5)	C(13)	C(12)	108.45(13)
N(1)	C(3)	C(9)	117.69(12)	C(21)	C(14)	C(11)	119.49(14)
C(12)	C(4)	N(1)	106.67(12)	C(7)	C(15)	C(2)	121.51(13)
C(17)	C(4)	N(1)	130.82(12)	C(24)	C(16)	C(19)	121.19(14)
C(17)	C(4)	C(12)	122.51(13)	C(4)	C(17)	C(22)	116.91(13)
N(1)	C(5)	C(1)	119.25(11)	C(11)	C(19)	C(16)	119.15(13)
C(13)	C(5)	N(1)	108.72(12)	C(8)	C(20)	C(7)	119.73(13)
C(13)	C(5)	C(1)	132.02(13)	C(14)	C(21)	C(24)	120.78(13)
C(10)	C(6)	C(9)	114.57(11)	C(17)	C(22)	C(23)	121.37(14)
C(15)	C(7)	C(20)	119.83(13)	C(25)	C(23)	C(22)	121.31(14)
C(20)	C(8)	C(1)	120.84(13)	C(16)	C(24)	C(21)	119.20(14)
C(2)	C(9)	C(3)	115.35(11)	C(23)	C(25)	C(12)	118.95(14)
C(2)	C(9)	C(6)	112.37(11)				

Supplementary Table 16. Hydrogen atom coordinates (Å×10^4) and isotropic displacement parameters (Å^2×10^3) for 7h.

Atom	*x*	*y*	*z*	U(eq)
H(6A)	9181.99	2797.99	6277.63	27
H(6B)	8223.03	2383.27	4982.15	27
H(7)	4267.29	2380.83	2389.37	30
H(8)	3611.16	4105.75	4806.92	29
H(10A)	8976.18	3754.19	4534.11	27
H(10B)	7563.86	3894.5	4251.33	27
H(13)	4337.88	4780.57	6895.2	30
H(14)	9218.6	5875.73	7248.82	29
H(15)	6227.93	2161.55	3865.76	29
H(16)	8235.62	6701.67	3017.17	32
H(17)	9004.59	4558.26	9723.03	31
H(18A)	6871.5	1818.38	7146.04	39
H(18B)	8258.95	1716.04	7325.6	39
H(18C)	7197.91	1417.79	6016.02	39
H(19)	8220.42	5157.77	3542	29
H(20)	2948.43	3358.68	2858.5	31
H(21)	9218.63	7409.55	6693.4	33
H(22)	8839	5570.67	11248.35	36
H(23)	6973.75	6183.2	11017.46	39
H(24)	8716.78	7829.25	4574.73	35
H(25)	5220.81	5827.62	9265.23	37

4. Spectral data of products

6-(Phenoxymethyl)phenanthridine

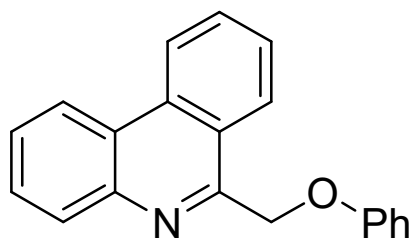

4a

White solid, 46.7 mg, 82%. ^{1}H NMR (400 MHz, Chloroform-*d*) δ 8.70 (d, *J* = 8.3 Hz, 1H), 8.62 (d, *J* = 7.9 Hz, 1H), 8.47 (d, *J* = 8.2 Hz, 1H), 8.23 (d, *J* = 7.9 Hz, 1H), 7.90 (t, *J* = 7.3 Hz, 1H), 7.85 – 7.67 (m, 3H), 7.39 – 7.30 (m, 2H), 7.16 (d, *J* = 8.2 Hz, 2H), 7.00 (t, *J* = 7.3 Hz, 1H), 5.74 (s, 2H). ^{13}C NMR (101 MHz, Chloroform-*d*) δ 158.7, 156.1, 156.1, 143.3, 133.3, 130.9, 130.2, 129.5, 128.8, 127.6, 127.5, 126.9, 125.2, 124.5, 122.4, 122.1, 121.2, 115.0, 71.7. HRMS (ESI) *m/z* calcd for $C_{20}H_{15}NO$, $[M+H]^+$: 286.1232, found: 286.1239.

8-Methoxy-6-(phenoxymethyl)phenanthridine

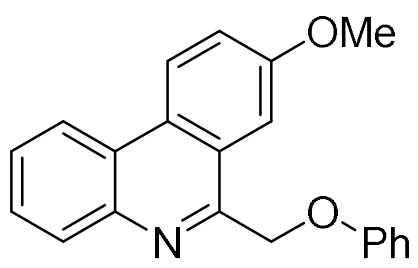

4b

White solid, 50.4 mg, 80%. ^{1}H NMR (400 MHz, Chloroform-*d*) δ 8.58 (d, *J* = 9.1 Hz, 1H), 8.52 (dd, *J* = 7.9, 1.7 Hz, 1H), 8.20 (dd, *J* = 8.0, 1.7 Hz, 1H), 7.82 (d, *J* = 2.7 Hz, 1H), 7.76 – 7.65 (m, 2H), 7.51 (dd, *J* = 9.1, 2.6 Hz, 1H), 7.32 (dd, *J* = 8.7, 7.3 Hz, 2H), 7.17 (d, *J* = 7.7 Hz, 2H), 6.99 (t, *J* = 7.3 Hz, 1H), 5.73 (s, 2H), 3.96 (s, 3H). ^{13}C NMR (101 MHz, Chloroform-*d*) δ 158.8, 158.6, 155.2, 142.5, 130.1, 129.6, 127.8, 127.7, 127.5, 126.5, 124.6, 124.0, 121.6, 121.6, 121.3, 115.0, 106.6, 72.2, 55.6. HRMS (ESI) *m/z* calcd for $C_{21}H_{17}NO_2$, $[M+H]^+$: 316.1338, found: 316.1341.

8-Chloro-6-(phenoxymethyl)phenanthridine

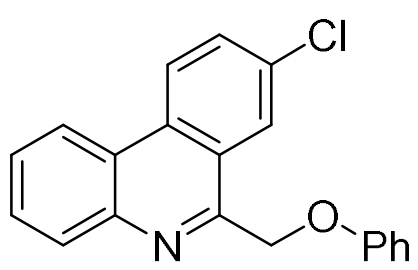

4c

White solid, 45.9 mg, 72%. ^{1}H NMR (400 MHz, Chloroform-*d*) δ 8.60 (d, *J* = 8.9 Hz, 1H), 8.54 (d, *J* = 7.9 Hz, 1H), 8.47 (d, *J* = 1.9 Hz, 1H), 8.22 (d, *J* = 8.1 Hz, 1H), 7.86 – 7.69 (m, 3H), 7.34 (t, *J*

= 7.9 Hz, 2H), 7.17 (d, *J* = 8.0 Hz, 2H), 7.01 (t, *J* = 7.3 Hz, 1H), 5.70 (s, 2H). ^{13}C NMR (101 MHz, $CDCl_3$) δ 158.5, 155.1, 143.2, 133.6, 131.7, 131.4, 130.4, 129.6, 129.1, 127.9, 126.2, 126.1, 124.1, 123.9, 121.9, 121.4, 115.1, 71.7. HRMS (ESI) *m/z* calcd for $C_{20}H_{14}ClNO$, $[M+H]^+$: 320.0842, found: 320.0847.

6-(Phenoxymethyl)phenanthridine-8-carbonitrile

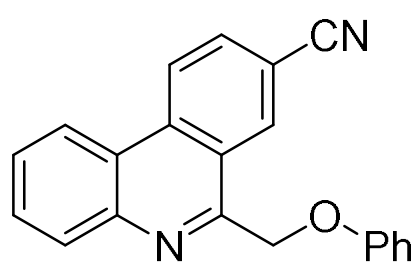

4d

White solid, 42.2 mg, 68%. ^{1}H NMR (400 MHz, Chloroform-*d*) δ 8.89 (s, 1H), 8.75 (d, *J* = 8.6 Hz, 1H), 8.59 (d, *J* = 8.2 Hz, 1H), 8.26 (d, *J* = 8.2 Hz, 1H), 8.05 (d, *J* = 8.6 Hz, 1H), 7.89 (t, *J* = 7.6 Hz, 1H), 7.79 (t, *J* = 7.7 Hz, 1H), 7.33 (t, *J* = 7.8 Hz, 2H), 7.15 (d, *J* = 8.4 Hz, 2H), 7.01 (t, *J* = 7.4 Hz, 1H), 5.73 (s, 2H). ^{13}C NMR (101 MHz, Chloroform-*d*) δ 158.2, 155.6, 144.2, 135.9, 132.5, 132.0, 130.6, 130.6, 129.7, 128.4, 124.6, 123.7, 123.2, 122.6, 121.6, 118.6, 115.0, 111.1, 71.6. HRMS (ESI) *m/z* calcd for $C_{21}H_{14}N_2O$, $[M+H]^+$: 311.1184, found: 311.1188.

6-(Phenoxymethyl)phenanthridin-8-yl acetate

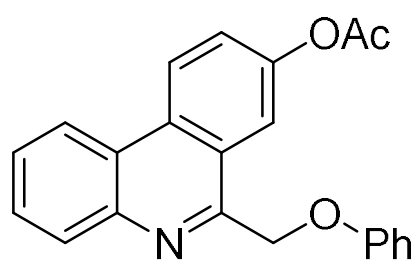

4e

White solid, 35.6 mg, 52%. ^{1}H NMR (400 MHz, Chloroform-*d*) δ 8.70 (d, *J* = 9.0 Hz, 1H), 8.57 (d, *J* = 8.1 Hz, 1H), 8.22 (d, *J* = 8.1 Hz, 1H), 8.17 (d, *J* = 2.4 Hz, 1H), 7.82 – 7.69 (m, 2H), 7.66 (d, *J* = 9.4 Hz, 1H), 7.33 (t, *J* = 7.8 Hz, 2H), 7.16 (d, *J* = 8.1 Hz, 2H), 7.00 (t, *J* = 7.3 Hz, 1H), 5.70 (s, 2H), 2.41 (s, 3H). ^{13}C NMR (101 MHz, Chloroform-*d*) δ 169.5, 158.5, 155.5, 149.6, 131.3, 130.3, 129.5 (2C), 128.9, 127.8, 126.0, 125.6, 124.1, 124.0, 122.0, 121.3, 118.6, 115.1, 71.6, 21.2. HRMS (ESI) *m/z* calcd for $C_{22}H_{17}NO_3$, $[M+H]^+$: 344.1287, found: 344.1289.

Ethyl 6-(phenoxymethyl)phenanthridine-8-carboxylate

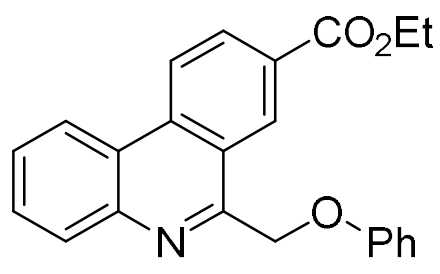

4f

White solid, 48.6 mg, 68%. ^{1}H NMR (400 MHz, Chloroform-*d*) δ 9.20 (d, *J* = 1.4 Hz, 1H), 8.68 (d, *J* = 8.7 Hz, 1H), 8.59 (d, *J* = 7.9 Hz, 1H), 8.47 (dd, *J* = 8.7, 1.6 Hz, 1H), 8.24 (d, *J* = 7.7 Hz, 1H), 7.82 (t, *J* = 8.1 Hz, 1H), 7.73 (t, *J* = 8.1 Hz, 1H), 7.33 (t, *J* = 8.0 Hz, 2H), 7.18 (d, *J* = 8.0 Hz, 2H), 7.00 (t, *J* = 7.3 Hz, 1H), 5.78 (s, 2H), 4.48 (q, *J* = 7.1 Hz, 2H), 1.45 (t, *J* = 7.1 Hz, 3H). ^{13}C NMR (101 MHz, Chloroform-*d*) δ 166.0, 158.6, 156.6, 144.0, 136.2, 130.6, 130.3, 129.9, 129.5, 129.3, 129.1, 127.8, 124.6, 123.8, 122.6, 122.6, 121.4, 115.3, 71.7, 61.5, 14.4. HRMS (ESI) *m/z* calcd for $C_{23}H_{19}NO_3$, $[M+H]^+$: 358.1443, found: 358.1445.

5-(Phenoxymethyl)benzo[i]phenanthridine

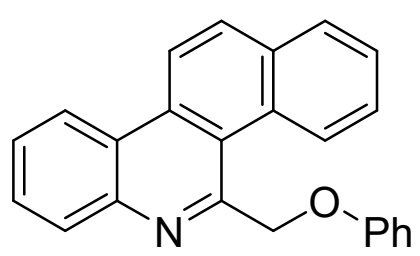

4g

White solid, 48.2 mg, 72%. ^{1}H NMR (400 MHz, Chloroform-*d*) δ 8.85 (d, *J* = 8.5 Hz, 1H), 8.71 – 8.60 (m, 1H), 8.31 (d, *J* = 8.8 Hz, 1H), 8.20 (d, *J* = 9.0 Hz, 1H), 8.04 (d, *J* = 7.4 Hz, 1H), 7.84 (t, *J* = 7.5 Hz, 1H), 7.76 (t, *J* = 7.0 Hz, 1H), 7.72 – 7.62 (m, 2H), 7.39 (t, *J* = 8.8 Hz, 1H), 7.19 (d, *J* = 7.9 Hz, 2H), 7.07 (t, *J* = 7.3 Hz, 1H), 5.85 (s, 2H). ^{13}C NMR (101 MHz, Chloroform-*d*) δ 158.4, 153.7, 143.9, 134.4, 133.1, 132.4, 129.8 (2C), 129.6, 129.1, 128.9, 127.8, 127.5, 127.2, 126.8, 124.2, 123.2, 122.6, 121.4, 120.2, 115.2, 74.1. HRMS (ESI) *m/z* calcd for $C_{24}H_{17}NO$, $[M+H]^+$: 336.1388, found: 336.1391.

5-(Phenoxymethyl)dibenzo[i,k]phenanthridine

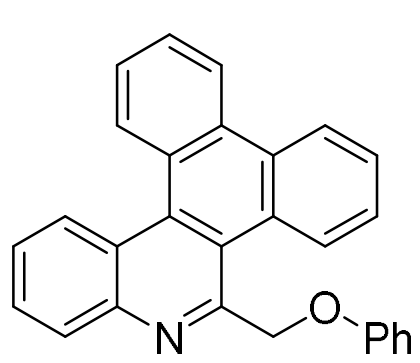

4h

White solid, 30.8 mg, 40%. ^{1}H NMR (400 MHz, Chloroform-*d*) δ 8.84 (d, *J* = 8.1 Hz, 1H), 8.79 –

8.69 (m, 3H), 8.61 (d, *J* = 8.9 Hz, 1H), 8.35 (d, *J* = 8.2 Hz, 1H), 7.85 – 7.77 (m, 2H), 7.76 – 7.66 (m, 3H), 7.59 (t, *J* = 8.3 Hz, 1H), 7.37 (dd, *J* = 8.6, 7.4 Hz, 2H), 7.14 (d, *J* = 7.8 Hz, 2H), 7.06 (t, *J* = 7.4 Hz, 1H), 5.71 (s, 2H). ^{13}C NMR (101 MHz, Chloroform-*d*) δ 158.5, 152.5, 135.4, 132.5, 130.6, 130.0, 129.5, 128.92, 128.88, 128.8, 128.6, 128.1, 127.8, 127.6, 127.4, 127.1, 126.8, 123.8, 123.6, 123.41, 123.38, 121.4, 115.2, 73.0. HRMS (ESI) *m/z* calcd for $C_{28}H_{19}NO$, $[M+H]^+$: 386.1545, found: 386.1548.

5-(Phenoxymethyl)benzo[c][2,7]naphthyridine

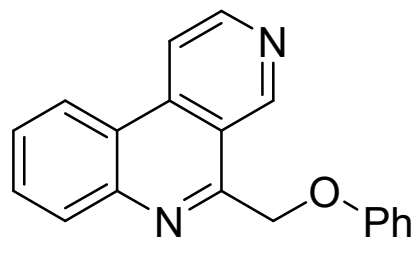

4i

White solid, 30.7 mg, 54%. ^{1}H NMR (400 MHz, Chloroform-*d*) δ 9.88 (s, 1H), 8.99 (s, 1H), 8.59 (d, *J* = 8.1 Hz, 1H), 8.43 (d, *J* = 5.3 Hz, 1H), 8.26 (d, *J* = 8.2 Hz, 1H), 7.91 (t, *J* = 7.6 Hz, 1H), 7.79 (t, *J* = 7.1 Hz, 1H), 7.32 (t, *J* = 8.0 Hz, 2H), 7.15 (d, *J* = 7.9 Hz, 2H), 7.00 (t, *J* = 7.3 Hz, 1H), 5.78 (s, 2H). ^{13}C NMR (101 MHz, Chloroform-*d*) δ 158.3, 156.1, 150.9, 148.6, 144.6, 138.2, 131.1, 130.5 (2C), 129.6, 128.1, 122.6, 122.4, 121.5, 115.0 (2C), 71.2. HRMS (ESI) *m/z* calcd for $C_{19}H_{14}N_2O$, $[M+H]^+$: 287.1184, found: 287.1188.

2-Methoxy-6-(phenoxymethyl)phenanthridine

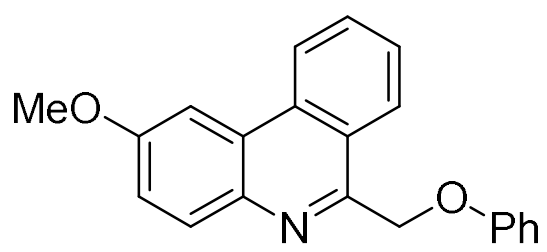

4j

White solid, 26.5 mg, 46%. ^{1}H NMR (400 MHz, Chloroform-*d*) δ 8.61 (d, *J* = 8.3 Hz, 1H), 8.44 (d, *J* = 8.3 Hz, 1H), 8.15 (d, *J* = 9.0 Hz, 1H), 7.94 (d, *J* = 2.7 Hz, 1H), 7.87 (t, *J* = 7.7 Hz, 1H), 7.73 (t, *J* = 7.6 Hz, 1H), 7.41 (dd, *J* = 9.0, 2.7 Hz, 1H), 7.33 (t, *J* = 8.0 Hz, 2H), 7.15 (d, *J* = 8.1 Hz, 2H), 7.01 (d, *J* = 7.3 Hz, 1H), 5.70 (s, 2H), 4.06 (s, 3H). ^{13}C NMR (101 MHz, Chloroform-*d*) δ 158.9, 158.7, 153.4, 132.8, 131.6, 130.4, 129.5, 127.7, 126.9, 125.7, 125.4, 122.4, 121.2, 118.7, 115.3, 115.0, 103.0, 71.6, 55.7. HRMS (ESI) *m/z* calcd for $C_{21}H_{17}NO_2$, $[M+H]^+$: 316.1338, found: 316.1335.

2-Chloro-6-(phenoxymethyl)phenanthridine

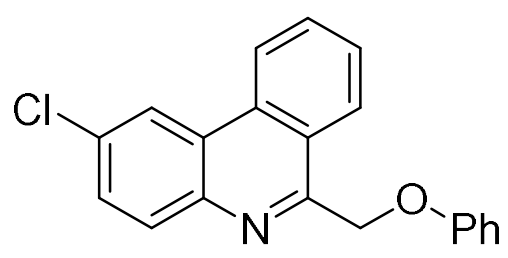

4k

White solid, 52.3 mg, 82%. ^{1}H NMR (400 MHz, Chloroform-*d*) δ 8.59 – 8.48 (m, 2H), 8.44 (d, *J* = 8.2 Hz, 1H), 8.13 (d, *J* = 8.7 Hz, 1H), 7.87 (t, *J* = 7.4 Hz, 1H), 7.79 – 7.66 (m, 2H), 7.34 (t, *J* = 7.9 Hz, 2H), 7.15 (d, *J* = 8.0 Hz, 2H), 7.01 (t, *J* = 7.3 Hz, 1H), 5.69 (s, 2H). ^{13}C NMR (101 MHz, Chloroform-*d*) δ 158.6, 156.3, 141.7, 133.4, 132.2, 131.7, 131.1, 129.6, 129.3, 128.2, 126.9, 125.6, 125.3, 122.4, 121.7, 121.3, 115.0, 71.5. HRMS (ESI) *m/z* calcd for $C_{20}H_{14}ClNO$, $[M+H]^+$: 320.0842, found: 320.0844.

8-Methoxy-6-(phenoxymethyl)phenanthridine-2-carbonitrile

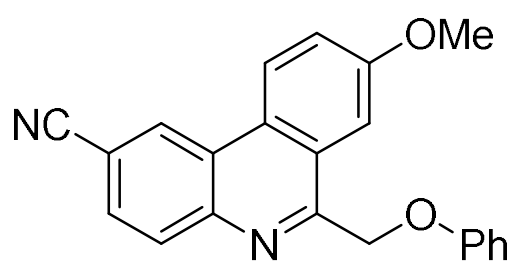

4l

White solid, 49.0 mg, 72%. ^{1}H NMR (400 MHz, Chloroform-*d*) δ 8.83 (s, 1H), 8.52 (d, *J* = 9.1 Hz, 1H), 8.25 (d, *J* = 8.5 Hz, 1H), 7.92 – 7.77 (m, 2H), 7.58 (dd, *J* = 9.1, 2.6 Hz, 1H), 7.32 (t, *J* = 7.8 Hz, 2H), 7.14 (d, *J* = 8.1 Hz, 2H), 7.00 (t, *J* = 7.4 Hz, 1H), 5.73 (s, 2H), 3.98 (s, 3H). ^{13}C NMR (101 MHz, Chloroform-*d*) δ 159.7, 158.6, 158.4, 144.0, 131.3, 130.0, 129.3, 127.4, 127.0, 126.5, 124.7, 123.9, 122.6, 121.5, 119.1, 115.0, 110.8, 107.1, 71.9, 55.7. HRMS (ESI) *m/z* calcd for $C_{22}H_{16}N_2O_2$, $[M+H]^+$: 341.1290, found: 341.1295.

1-Chloro-6-(phenoxymethyl)phenanthridine

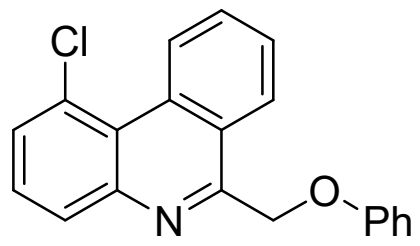

4m

White solid, 42.7 mg, 67%. ^{1}H NMR (400 MHz, Chloroform-*d*) δ 9.94 (d, *J* = 8.6 Hz, 1H), 8.49 (dd, *J* = 8.2, 1.1 Hz, 1H), 8.18 (dd, *J* = 8.1, 1.4 Hz, 1H), 7.90 (ddd, *J* = 8.6, 7.0, 1.4 Hz, 1H), 7.82

– 7.74 (m, 2H), 7.65 (t, *J* = 7.9 Hz, 1H), 7.33 (dd, *J* = 8.7, 7.4 Hz, 2H), 7.15 (d, *J* = 7.8 Hz, 2H), 7.01 (t, *J* = 7.3 Hz, 1H), 5.71 (s, 2H). ^{13}C NMR (101 MHz, Chloroform-*d*) δ 158.6, 156.8, 145.5, 132.6, 131.2, 130.7, 130.3, 130.1, 129.6, 128.0, 127.9, 126.6, 126.6, 126.0, 122.2, 121.3, 115.0, 71.4. HRMS (ESI) *m/z* calcd for $C_{20}H_{14}ClNO$, $[M+H]^+$: 320.0842, found: 320.0847.

3-Chloro-6-(phenoxymethyl)phenanthridine

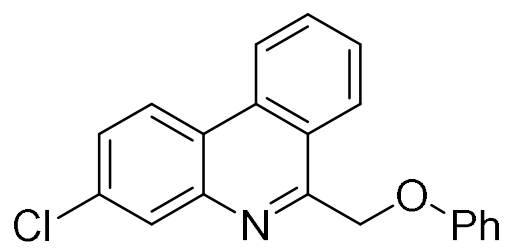

4n

White solid, 48.5 mg, 76%. ^{1}H NMR (400 MHz, Chloroform-*d*) δ 8.60 (d, *J* = 8.3 Hz, 1H), 8.53 – 8.43 (m, 2H), 8.21 (d, *J* = 2.2 Hz, 1H), 7.89 (t, *J* = 7.2 Hz, 1H), 7.74 (t, *J* = 7.6 Hz, 1H), 7.66 (dd, *J* = 8.8, 2.2 Hz, 1H), 7.33 (d, *J* = 8.6 Hz, 2H), 7.15 (d, *J* = 7.9 Hz, 2H), 7.00 (t, *J* = 7.3 Hz, 1H), 5.71 (s, 2H). ^{13}C NMR (101 MHz, Chloroform-*d*) δ 158.5, 157.4, 144.0, 134.4, 132.9, 131.2, 129.6, 129.4, 128.0, 127.9, 127.0, 125.1, 123.4, 123.0, 122.3, 121.3, 115.0, 71.5. HRMS (ESI) *m/z* calcd for $C_{20}H_{14}ClNO$, $[M+H]^+$: 320.0842, found: 320.0840.

4-Chloro-6-(phenoxymethyl)phenanthridine

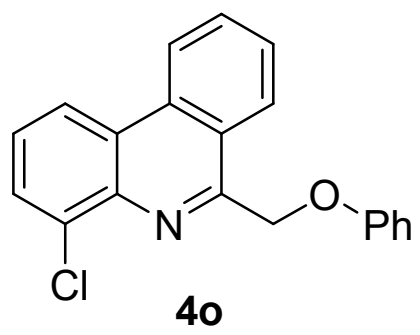

4o

White solid, 48.5 mg, 76%. ^{1}H NMR (400 MHz, Chloroform-*d*) δ 8.65 (d, *J* = 8.3 Hz, 1H), 8.54 – 8.48 (m, 2H), 7.94 – 7.85 (m, 2H), 7.76 (t, *J* = 7.6 Hz, 1H), 7.61 (t, *J* = 8.0 Hz, 1H), 7.34 (T, *J* = 8.7 Hz, 2H), 7.19 (d, *J* = 7.9 Hz, 2H), 7.00 (t, *J* = 7.3 Hz, 1H), 5.79 (s, 2H). ^{13}C NMR (101 MHz, Chloroform-*d*) δ 158.6, 156.9, 139.6, 134.7, 133.1, 131.2, 129.6, 129.2, 128.2, 127.3, 127.1, 126.2, 125.3, 122.6, 121.2, 121.0, 115.0, 71.7. HRMS (ESI) *m/z* calcd for $C_{20}H_{14}ClNO$, $[M+H]^+$: 320.0842, found: 320.0848.

2-(Phenoxymethyl)-4-phenylquinoline

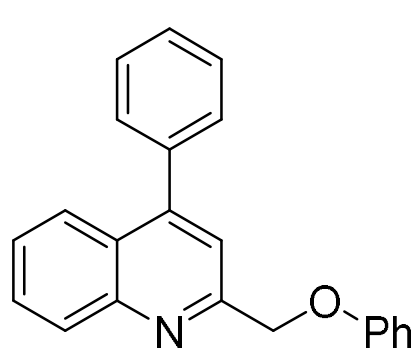

4p

White solid, 33.6 mg, 76%. ^{1}H NMR (400 MHz, Chloroform-*d*) δ 8.18 (d, *J* = 8.4 Hz, 1H), 7.95 (d, *J* = 8.4 Hz, 1H), 7.77 (t, *J* = 7.6 Hz, 1H), 7.67 (s, 1H), 7.62 – 7.45 (m, 5H), 7.32 (dd, *J* = 8.8, 7.3 Hz, 3H), 7.07 (d, *J* = 8.0 Hz, 2H), 7.00 (t, *J* = 7.4 Hz, 1H), 5.44 (s, 2H). ^{13}C NMR (101 MHz, Chloroform-*d*) δ 158.5, 157.5, 149.5, 148.1, 138.1, 129.7, 129.62, 129.59, 129.3, 128.6, 128.5, 126.6, 126.2, 125.9, 121.2, 119.4, 114.9, 71.3. HRMS (ESI) *m/z* calcd for $C_{22}H_{17}NO$, $[M+H]^+$: 312.1388, found: 312.1389.

Ethyl 1-(phenoxymethyl)-4-phenylisoquinoline-3-carboxylate

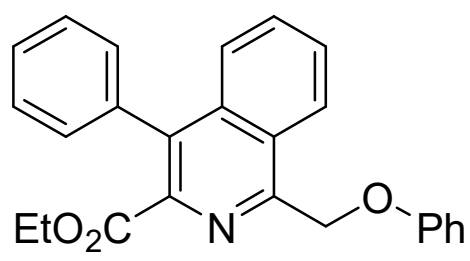

4q

White solid, 52.8 mg, 76%. ^{1}H NMR (400 MHz, Chloroform-*d*) δ 8.52 – 8.43 (m, 1H), 7.75 – 7.65 (m, 3H), 7.57 – 7.48 (m, 3H), 7.42 – 7.31 (m, 4H), 7.15 (d, *J* = 7.5 Hz, 2H), 7.02 (t, *J* = 7.3 Hz, 1H), 5.75 (s, 2H), 4.16 (q, *J* = 7.1 Hz, 2H), 1.01 (t, *J* = 7.1 Hz, 3H). ^{13}C NMR (101 MHz, Chloroform-*d*) δ 167.3, 158.5, 155.4, 141.2, 136.3, 136.1, 134.4, 130.8, 129.8, 129.6, 128.8, 128.2, 128.1, 127.9, 127.0, 126.0, 121.3, 114.9, 71.1, 61.4, 13.7. HRMS (ESI) *m/z* calcd for $C_{25}H_{21}NO_3$, $[M+H]^+$: 384.1600, found: 384.1604.

Ethyl 7-methoxy-4-(4-methoxyphenyl)-1-(phenoxymethyl)isoquinoline-3-carboxylate

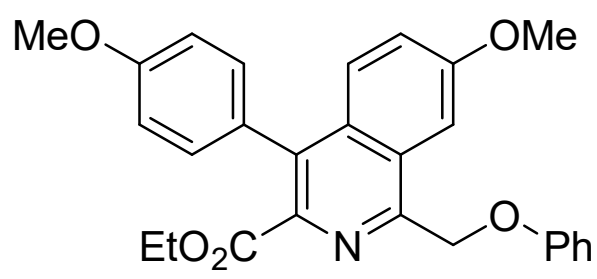

4r

White solid, 52.8 mg, 76%. ^{1}H NMR (400 MHz, Chloroform-*d*) δ 7.71 (d, *J* = 2.5 Hz, 1H), 7.64 (d, *J* = 9.3 Hz, 1H), 7.38 – 7.25 (m, 5H), 7.15 (d, *J* = 8.0 Hz, 2H), 7.07 – 6.99 (m, 3H), 5.72 (s, 2H), 4.19 (q, *J* = 7.1 Hz, 2H), 3.96 – 3.88 (m, 6H), 1.07 (t, *J* = 7.1 Hz, 3H). ^{13}C NMR (101 MHz,

Chloroform-*d*) δ 167.5, 159.4, 159.4, 158.6, 153.3, 139.7, 134.3, 132.0, 130.9, 129.6, 129.5, 128.7, 128.4, 123.4, 121.3, 114.91, 113.7, 103.7, 71.6, 61.3, 55.6, 55.4, 13.9. HRMS (ESI) *m/z* calcd for $C_{27}H_{25}NO_5$, $[M+H]^+$: 444.1811, found: 444.1817.

Ethyl 7-chloro-4-(4-chlorophenyl)-1-(phenoxymethyl)isoquinoline-3-carboxylate

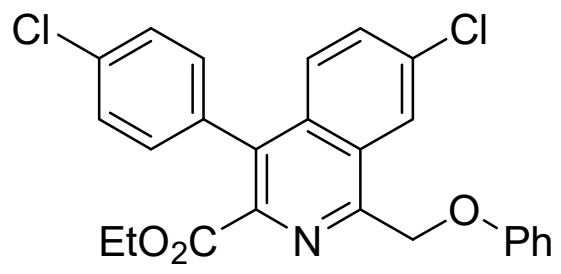

4s

White solid, 57.7 mg, 64%. ^{1}H NMR (400 MHz, Chloroform-*d*) δ 8.49 (s, 1H), 7.66 – 7.55 (m, 2H), 7.51 (d, *J* = 8.3 Hz, 2H), 7.36 (t, *J* = 7.9 Hz, 2H), 7.29 (d, *J* = 6.6 Hz, 2H), 7.14 (d, *J* = 8.3 Hz, 2H), 7.03 (t, *J* = 7.2 Hz, 1H), 5.70 (s, 2H), 4.20 (q, *J* = 7.2 Hz, 2H), 1.08 (t, *J* = 7.1 Hz, 3H). ^{13}C NMR (101 MHz, Chloroform-*d*) δ 166.6, 158.3, 155.1, 141.2, 135.2, 134.6, 134.5, 134.1, 133.1, 132.1, 131.1, 129.7, 128.7, 128.5, 128.5, 125.2, 121.5, 114.9, 71.0, 61.7, 13.8. HRMS (ESI) *m/z* calcd for $C_{25}H_{19}Cl_2NO_3$, $[M+H]^+$: 452.0820, found: 452.0821.

2-(Phenoxymethyl)benzo[d]thiazole

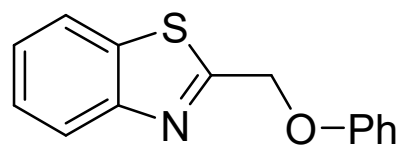

4t

White solid, 33.2 mg, 69%. ^{1}H NMR (400 MHz, Chloroform-*d*) δ 8.06 (d, *J* = 8.1 Hz, 1H), 7.92 (d, *J* = 7.6 Hz, 1H), 7.53 (ddd, *J* = 8.3, 7.3, 1.2 Hz, 1H), 7.46 – 7.40 (m, 1H), 7.39 – 7.31 (m, 2H), 7.12 – 6.97 (m, 3H), 5.52 (s, 2H). ^{13}C NMR (101 MHz, Chloroform-*d*) δ 168.7, 157.8, 153.0, 135.1, 129.7, 126.2, 125.3, 123.1, 121.9, 121.9, 115.0, 67.8. HRMS (ESI) *m/z* calcd for $C_{14}H_{11}NOS$, $[M+H]^+$: 242.0640, found: 242.0641.

6-Methoxy-2-(phenoxymethyl)benzo[d]thiazole

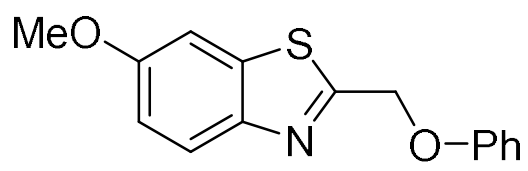

4u

White solid, 36.3 mg, 67%. ^{1}H NMR (400 MHz, Chloroform-*d*) δ 7.75 (d, *J* = 8.8 Hz, 1H), 7.53 (d,

J = 2.5 Hz, 1H), 7.34 (t, J = 8.0 Hz, 2H), 7.10 – 6.99 (m, 4H), 5.49 (s, 2H), 3.92 (s, 3H). ^{13}C NMR (101 MHz, Chloroform-*d*) δ 169.8, 159.0, 157.8, 154.3, 129.7, 126.9, 122.1, 121.9, 115.5, 114.9, 105.5, 67.8, 55.6. HRMS (ESI) *m/z* calcd for $C_{15}H_{13}NO2S$, $[M+H]^+$: 272.0745, found: 272.0747.

6-Chloro-2-(phenoxymethyl)benzo[d]thiazole

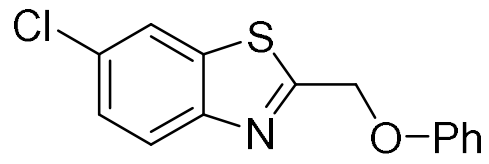

4v

White solid, 30.8 mg, 56%. ^{1}H NMR (400 MHz, Chloroform-*d*) δ 7.96 (d, J = 8.7 Hz, 1H), 7.89 (d, J = 2.1 Hz, 1H), 7.48 (dd, J = 8.7, 2.1 Hz, 1H), 7.35 (dd, J = 8.9, 7.0 Hz, 2H), 7.11 – 6.99 (m, 3H), 5.49 (s, 2H). ^{13}C NMR (101 MHz, $CDCl_3$) δ 169.3, 157.7, 151.6, 136.2, 131.3, 129.8, 127.1, 123.9, 122.1, 121.5, 114.9, 67.7. HRMS (ESI) *m/z* calcd for $C_{14}H_{10}ClNOS$, $[M+H]^+$: 276.0250, found: 276.0253.

6-Fluoro-2-(phenoxymethyl)benzo[d]thiazole

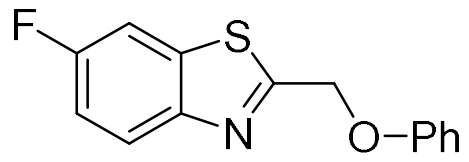

4w

White solid, 32.1 mg, 62%. ^{1}H NMR (400 MHz, Chloroform-*d*) δ 7.99 (dd, J = 8.9, 4.8 Hz, 1H), 7.59 (dd, J = 8.1, 2.6 Hz, 1H), 7.35 (dd, J = 8.9, 7.2 Hz, 2H), 7.28 – 7.21 (m, 1H), 7.08 – 7.01 (m, 3H), 5.48 (s, 2H). ^{13}C NMR (101 MHz, Chloroform-*d*) δ 168.4 (d, J = 3.4 Hz), 160.5 (d, J = 245.8 Hz), 157.7, 149.6 (d, J = 1.7 Hz), 136.1 (d, J = 11.2 Hz), 129.7, 124.1 (d, J = 9.5 Hz), 122.0, 114.922 (d, J = 25.8 Hz), 114.917, 108.0 (d, J = 26.8 Hz), 67.7. ^{19}F NMR (376 MHz, Chloroform-*d*) δ -115.77. HRMS (ESI) *m/z* calcd for $C_{14}H_{10}FNOS$, $[M+H]^+$: 260.0545, found: 260.0547.

2-(Phenoxymethyl)-6-(trifluoromethoxy)benzo[d]thiazole

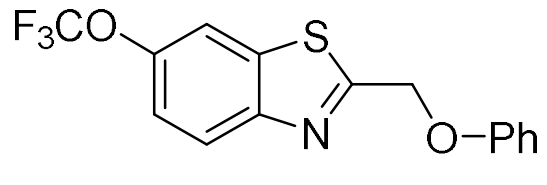

4x

White solid, 42.2 mg, 65%. ^{1}H NMR (400 MHz, Chloroform-*d*) δ 8.05 (d, J = 8.9 Hz, 1H), 7.79 (d,

J = 1.2 Hz, 1H), 7.43 – 7.31 (m, 3H), 7.10 – 7.02 (m, 3H), 5.51 (s, 2H). ^{13}C NMR (101 MHz, Chloroform-*d*) δ 170.1, 157.6, 151.5, 146.5 (q, J = 2.0 Hz), 135.9, 129.8, 124.0, 122.1, 120.5 (q, J = 257.6 Hz), 120.2, 114.9, 114.4, 67.7. ^{19}F NMR (376 MHz, Chloroform-*d*) δ -58.01. HRMS (ESI) *m/z* calcd for $C_{15}H_{10}F_3NO_2S$, $[M+H]^+$: 326.0463, found: 326.0467.

5-Chloro-2-(phenoxymethyl)benzo[d]thiazole

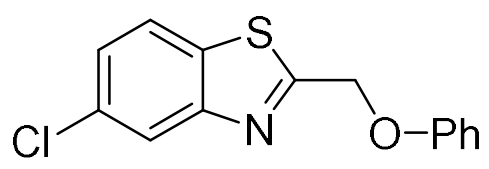

4y

White solid, 36.9 mg, 67%. ^{1}H NMR (400 MHz, Chloroform-*d*) δ 8.04 (d, J = 2.1 Hz, 1H), 7.82 (d, J = 8.5 Hz, 1H), 7.44 – 7.32 (m, 3H), 7.11 – 6.99 (m, 3H), 5.50 (s, 2H). ^{13}C NMR (101 MHz, Chloroform-*d*) δ 170.9, 157.7, 153.9, 133.3, 132.3, 129.8, 125.8, 123.0, 122.6, 122.1, 114.9, 67.7. HRMS (ESI) *m/z* calcd for $C_{14}H_{10}ClNOS$, $[M+H]^+$: 276.0250, found: 276.0249.

5-Bromo-2-(phenoxymethyl)benzo[d]thiazole

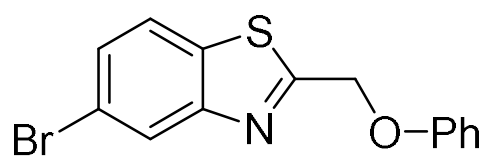

4z

White solid, 35.6 mg, 56%. ^{1}H NMR (400 MHz, Chloroform-*d*) δ 8.20 (d, J = 1.9 Hz, 1H), 7.78 (d, J = 8.5 Hz, 1H), 7.54 (dd, J = 8.5, 1.9 Hz, 1H), 7.35 (dd, J = 8.7, 7.4 Hz, 2H), 7.10 – 7.03 (m, 3H), 5.50 (s, 2H). ^{13}C NMR (101 MHz, Chloroform-*d*) δ 170.7, 157.7, 154.2, 133.8, 129.8, 128.4, 126.0, 122.9, 122.1, 119.8, 114.9, 67.7. HRMS (ESI) *m/z* calcd for $C_{14}H_{10}ClNOS$, $[M+H]^+$: 319.9745, found: 319.9743.

Ethyl 2-methyl-2-((6-(phenoxymethyl)phenanthridin-8-yl)oxy)propanoate

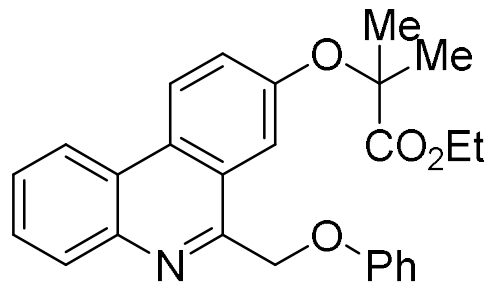

4aa

White solid, 68.1 mg, 82%. ^{1}H NMR (400 MHz, Chloroform-*d*) δ 8.56 (d, J = 9.1 Hz, 1H), 8.51 (d,

J = 7.7 Hz, 1H), 8.19 (d, J = 8.0 Hz, 1H), 7.82 (d, J = 2.5 Hz, 1H), 7.77 – 7.59 (m, 2H), 7.49 (dd, J = 9.0, 2.5 Hz, 1H), 7.31 (t, J = 7.9 Hz, 2H), 7.16 (d, J = 8.1 Hz, 2H), 6.98 (t, J = 7.3 Hz, 1H), 5.65 (s, 8H), 4.19 (q, J = 7.1 Hz, 2H), 1.72 (s, 6H), 1.20 (t, J = 7.1 Hz, 3H). ^{13}C NMR (101 MHz, Chloroform-*d*) δ 173.8, 158.5, 155.3, 154.6, 142.7, 130.1, 129.5, 128.3, 128.0, 127.6, 126.1, 124.5, 124.4, 123.8, 121.7, 121.2, 115.0, 113.4, 79.7, 71.9, 61.7, 25.4, 14.1. HRMS (ESI) *m/z* calcd for $C_{26}H_{25}NO_4$, $[M+H]^+$: 416.1862, found: 416.1859.

Isopropyl 2-methyl-2-(4-(6-(phenoxymethyl)phenanthridine-8-carbonyl)phenoxy)propanoate

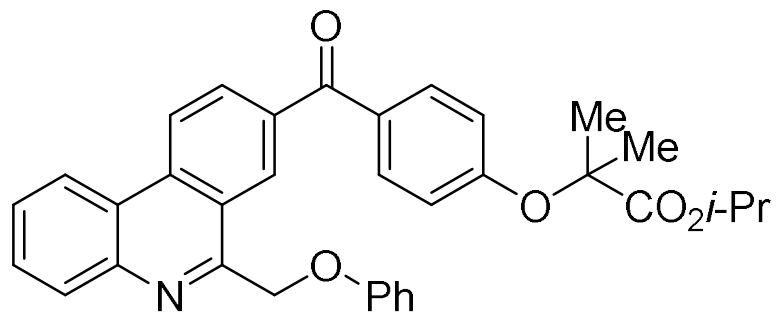

4ab

White solid, 84.2 mg, 79%. ^1H NMR (400 MHz, Chloroform-*d*) δ 8.92 (d, J = 1.7 Hz, 1H), 8.76 (d, J = 8.6 Hz, 1H), 8.64 (d, J = 7.4 Hz, 1H), 8.29 (dd, J = 8.6, 1.7 Hz, 1H), 8.25 (dd, J = 8.2, 1.4 Hz, 1H), 7.90 (d, J = 8.9 Hz, 2H), 7.84 (ddd, J = 8.2, 7.0, 1.4 Hz, 1H), 7.76 (ddd, J = 8.3, 7.0, 1.4 Hz, 1H), 7.30 (dd, J = 8.5, 7.0 Hz, 2H), 7.12 (d, J = 7.7 Hz, 2H), 6.97 (t, J = 7.3 Hz, 1H), 6.90 (d, J = 8.8 Hz, 2H), 5.72 (s, 2H), 5.11 (hept, J = 6.2 Hz, 1H), 1.68 (s, 6H), 1.22 (d, J = 6.3 Hz, 6H). ^{13}C NMR (101 MHz, Chloroform-*d*) δ 194.5, 173.1, 159.9, 158.4, 156.7, 144.0, 136.7, 135.6, 132.3, 131.1, 130.4, 130.1, 129.8, 129.6, 129.2, 127.9, 124.3, 123.9, 122.8, 122.6, 121.3, 117.3, 115.0, 79.5, 71.7, 69.4, 25.4, 21.5. HRMS (ESI) *m/z* calcd for $C_{34}H_{31}NO_5$, $[M+H]^+$: 534.2280, found: 534.2285.

6-(Phenoxymethyl)phenanthridin-8-yl (*S*)-2-(7-methoxynaphthalen-2-yl)propanoate

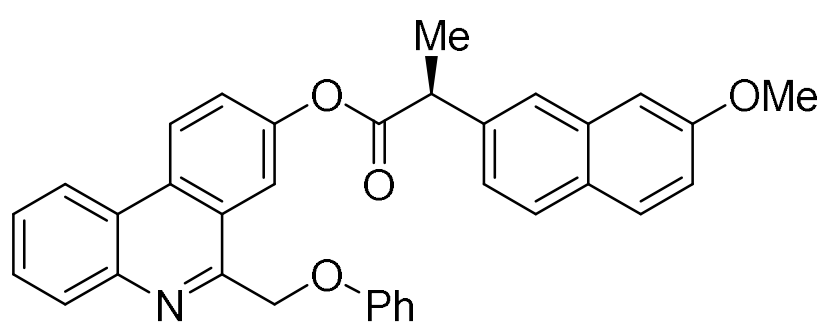

4ac

White solid, 82.1 mg, 80%. ^1H NMR (400 MHz, Chloroform-*d*) δ 8.61 (d, J = 9.0 Hz, 1H), 8.52 (d, J = 8.1 Hz, 1H), 8.20 (d, J = 8.0 Hz, 1H), 8.09 (d, J = 2.1 Hz, 1H), 7.83 (s, 1H), 7.80 – 7.65 (m, 4H), 7.55 (d, J = 7.4 Hz, 1H), 7.51 (dd, J = 9.0, 2.1 Hz, 1H), 7.31 (t, J = 7.9 Hz, 2H), 7.22 – 7.14

(m, 2H), 7.11 (d, J = 8.2 Hz, 2H), 7.01 (t, J = 7.3 Hz, 1H), 5.70 – 5.60 (m, 2H), 4.21 (q, J = 7.1 Hz, 1H), 3.96 (s, 3H), 1.76 (d, J = 7.1 Hz, 3H). ^{13}C NMR (101 MHz, Chloroform-*d*) δ 173.3, 158.5, 157.8, 155.5, 149.8, 143.1, 134.9, 133.9, 131.2, 130.2, 129.6, 129.4, 129.0, 128.8, 127.8, 127.6, 126.3, 126.1, 125.9, 125.5, 124.1, 123.9, 122.0, 121.3, 119.3, 118.4, 115.1, 105.6, 71.6, 55.4, 45.6, 18.6. HRMS (ESI) *m/z* calcd for $C_{34}H_{27}NO_4$, $[M+H]^+$: 514.2018, found: 514.2020.

6-((4-Methoxyphenoxy)methyl)phenanthridine

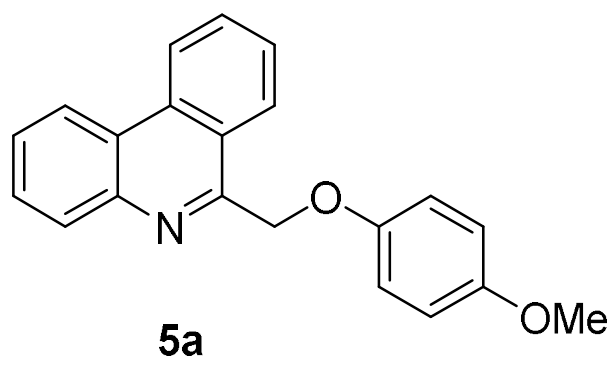

5a

White solid, 29.0 mg, 46%. ^{1}H NMR (400 MHz, Chloroform-*d*) δ 8.69 (d, J = 8.3 Hz, 1H), 8.61 (d, J = 8.1 Hz, 1H), 8.48 (d, J = 8.3 Hz, 1H), 8.22 (d, J = 8.1 Hz, 1H), 7.89 (t, J = 7.7 Hz, 1H), 7.82 – 7.69 (m, 3H), 7.08 (d, J = 9.0 Hz, 2H), 6.86 (d, J = 9.0 Hz, 2H), 5.69 (s, 2H), 3.78 (s, 3H). ^{13}C NMR (101 MHz, Chloroform-*d*) δ 156.3, 154.1, 152.8, 133.3, 130.8, 130.2, 128.8, 127.6, 127.5, 126.9, 125.2, 124.5, 122.3, 122.1, 116.0, 114.7 (2C), 72.4, 55.7. HRMS (ESI) *m/z* calcd for $C_{21}H_{17}NO_2$, $[M+H]^+$: 316.1338, found: 316.1340.

6-((4-Bromophenoxy)methyl)phenanthridine

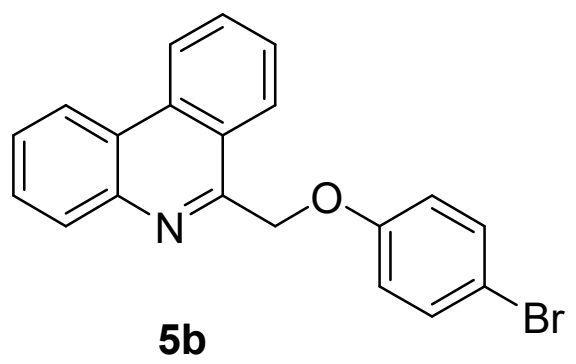

5b

White solid, 52.3 mg, 72%. ^{1}H NMR (400 MHz, Chloroform-*d*) δ 8.69 (d, J = 8.4 Hz, 1H), 8.61 (d, J = 8.0 Hz, 1H), 8.42 (d, J = 8.2 Hz, 1H), 8.22 (d, J = 8.0 Hz, 1H), 7.90 (t, J = 7.6 Hz, 1H), 7.83 – 7.68 (m, 3H), 7.39 (d, J = 8.9 Hz, 2H), 7.04 (d, J = 8.9 Hz, 2H), 5.71 (s, 2H). ^{13}C NMR (101 MHz, Chloroform-*d*) δ ^{13}C NMR (101 MHz, $CDCl_3$) δ 157.7, 155.6, 143.2, 133.3, 132.3, 130.9, 130.2, 128.9, 127.64, 127.63, 126.6, 125.0, 124.5, 122.4, 122.1, 116.9, 113.5, 71.9. HRMS (ESI) *m/z* calcd for $C_{20}H_{14}BrNO$, $[M+H]^+$: 364.0337, found: 364.0338.

6-((4-Fluorophenoxy)methyl)phenanthridine

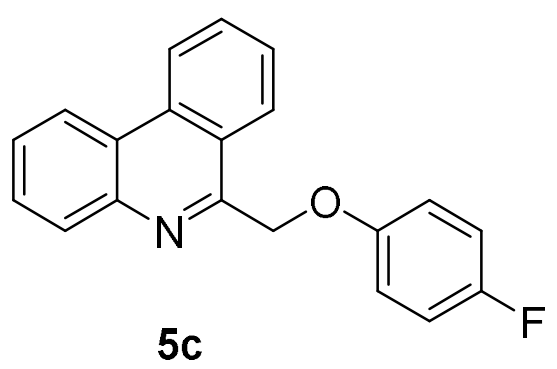

5c

White solid, 47.2 mg, 78%. ^{1}H NMR (400 MHz, Chloroform-*d*) δ 8.69 (d, *J* = 8.3 Hz, 1H), 8.62 (d, *J* = 8.1 Hz, 1H), 8.45 (d, *J* = 8.2 Hz, 1H), 8.22 (d, *J* = 8.1 Hz, 1H), 7.90 (t, *J* = 7.7 Hz, 1H), 7.83 – 7.70 (m, 3H), 7.13 – 7.06 (m, 2H), 7.02 – 6.95 (m, 2H), 5.70 (s, 2H). ^{13}C NMR (101 MHz, Chloroform-*d*) δ 157.5 (d, *J* = 238.7 Hz), 155.9, 154.7 (d, *J* = 2.2 Hz), 143.2, 133.3, 130.9, 130.2, 128.9, 127.64, 127.59, 126.7, 125.1, 124.5, 122.4, 122.1, 116.1 (d, *J* = 8.2 Hz), 115.9 (d, *J* = 23.2 Hz), 72.3. ^{19}F NMR (376 MHz, Chloroform-*d*) δ -123.42. HRMS (ESI) *m/z* calcd for $C_{20}H_{14}FNO$, $[M+H]^+$: 304.1138, found: 304.1141.

6-((3-Methoxyphenoxy)methyl)phenanthridine

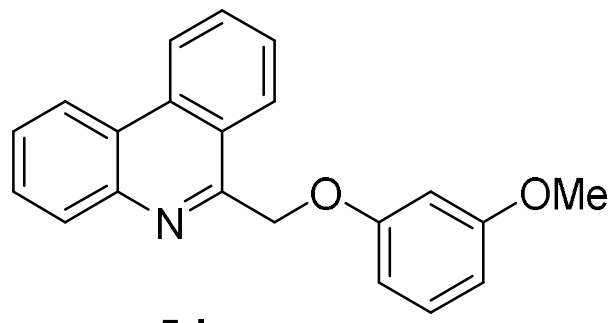

5d

White solid, 42.8 mg, 68%. ^{1}H NMR (400 MHz, Chloroform-*d*) δ 8.68 (d, *J* = 8.4 Hz, 1H), 8.61 (d, *J* = 7.9 Hz, 1H), 8.44 (d, *J* = 8.2 Hz, 1H), 8.22 (d, *J* = 7.9 Hz, 1H), 7.89 (t, *J* = 7.3 Hz, 1H), 7.84 – 7.67 (m, 3H), 7.23 (t, *J* = 8.1 Hz, 1H), 6.82 – 6.71 (m, 2H), 6.57 (dd, *J* = 8.3, 1.7 Hz, 1H), 5.72 (s, 2H), 3.80 (s, 3H). ^{13}C NMR (101 MHz, Chloroform-*d*) δ 160.8, 159.9, 156.0, 143.3, 133.3, 130.9, 130.2, 130.0, 128.9, 127.6, 127.5, 126.8, 125.2, 124.5, 122.4, 122.1, 107.1, 107.0, 101.3, 71.7, 55.3. HRMS (ESI) *m/z* calcd for $C_{21}H_{17}NO_2$, $[M+H]^+$: 316.1338, found: 316.1342.

6-((2-Methoxyphenoxy)methyl)phenanthridine

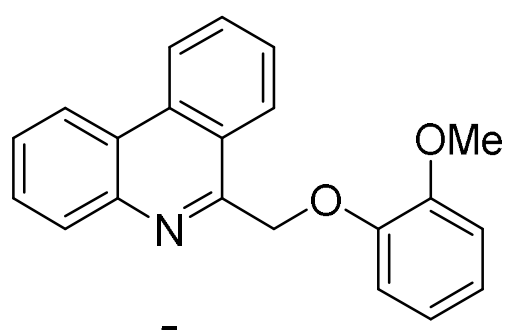

5e

White solid, 39.6 mg, 36%. ^{1}H NMR (400 MHz, Chloroform-*d*) δ 8.72 – 8.58 (m, 3H), 8.22 (d, *J* = 8.1 Hz, 1H), 7.89 (t, *J* = 7.7 Hz, 1H), 7.82 – 7.69 (m, 3H), 7.26 (d, *J* = 7.5 Hz, 1H), 7.00 – 6.84 (m, 3H), 5.79 (s, 2H), 3.88 (s, 3H). ^{13}C NMR (101 MHz, Chloroform-*d*) δ 156.3, 149.8, 148.1,

143.3, 133.3, 130.8, 130.2, 128.8, 127.6, 127.4, 127.1, 125.3, 124.5, 122.3, 122.1, 121.9, 120.9, 115.0, 111.9, 73.0, 56.0. HRMS (ESI) *m/z* calcd for $C_{21}H_{17}NO_2$, $[M+H]^+$: 316.1338, found: 316.1335.

6-((Naphthalen-2-yloxy)methyl)phenanthridine

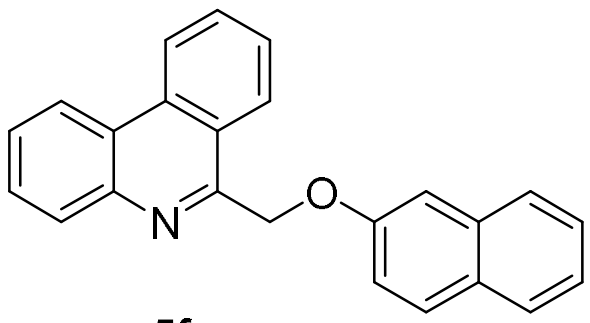

5f

White solid, 37.54 mg, 56%. ^{1}H NMR (400 MHz, Chloroform-*d*) δ 8.71 (d, *J* = 8.3 Hz, 1H), 8.63 (d, *J* = 7.1 Hz, 1H), 8.49 (d, *J* = 8.0 Hz, 1H), 8.27 (d, *J* = 8.9 Hz, 1H), 7.91 (ddd, *J* = 8.3, 7.0, 1.3 Hz, 1H), 7.83 – 7.71 (m, 6H), 7.54 (d, *J* = 2.6 Hz, 1H), 7.47 (ddd, *J* = 8.1, 6.9, 1.3 Hz, 1H), 7.37 (ddd, *J* = 8.2, 6.9, 1.2 Hz, 1H), 7.29 (dd, *J* = 8.9, 2.7 Hz, 1H), 5.86 (s, 2H). ^{13}C NMR (101 MHz, Chloroform-*d*) δ 156.6, 155.9, 143.3, 134.5, 133.3, 130.9, 130.2, 129.5, 129.2, 128.9, 127.68, 127.66, 127.6, 127.0, 126.8, 126.5, 125.2, 124.6, 123.9, 122.4, 122.1, 119.0, 107.6, 71.6. HRMS (ESI) *m/z* calcd for $C_{24}H_{17}NO$, $[M+H]^+$: 336.1388, found: 336.1383.

6-((Benzyloxy)methyl)phenanthridine

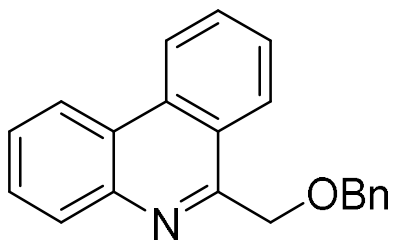

5g

White solid, 43.0 mg, 72%. ^{1}H NMR (400 MHz, Chloroform-*d*) δ 8.66 (d, *J* = 8.3 Hz, 1H), 8.60 (d, *J* = 8.1 Hz, 1H), 8.23 (d, *J* = 8.2 Hz, 2H), 7.85 – 7.74 (m, 2H), 7.69 (ddd, *J* = 8.3, 7.0, 1.4 Hz, 1H), 7.61 (ddd, *J* = 8.2, 7.0, 1.2 Hz, 1H), 7.34 (t, *J* = 7.2 Hz, 2H), 7.29 – 7.24 (m, 3H), 7.19 (t, *J* = 7.3 Hz, 1H), 4.79 (s, 2H). ^{13}C NMR (101 MHz, Chloroform-*d*) δ 160.2, 143.7, 139.1, 133.3, 130.5, 129.8, 128.8, 128.59, 128.57, 127.4, 127.1, 126.8, 126.4, 125.4, 124.0, 122.6, 122.0, 77.3, 43.1. HRMS (ESI) *m/z* calcd for $C_{21}H_{17}NO$, $[M+H]^+$: 300.1388, found: 300.1391.

6-(Methoxymethyl)phenanthridine

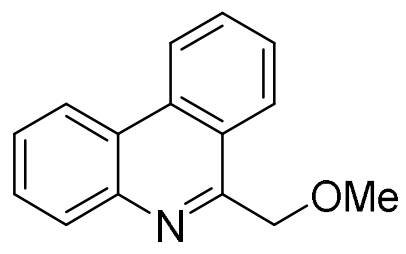

5h

White solid, 31.6 mg, 71%. ^{1}H NMR (400 MHz, Chloroform-*d*) δ 8.68 (d, *J* = 8.3 Hz, 1H), 8.60 (d, *J* = 7.8 Hz, 1H), 8.44 (d, *J* = 8.2 Hz, 1H), 8.22 (d, *J* = 8.1 Hz, 1H), 7.89 (t, *J* = 7.7 Hz, 1H), 7.82 – 7.66 (m, 3H), 5.17 (s, 2H), 3.55 (s, 3H). ^{13}C NMR (101 MHz, Chloroform-*d*) δ 157.2, 143.3, 133.1, 130.8, 130.2, 128.7, 127.5, 127.2, 126.6, 125.0, 124.4, 122.3, 122.0, 75.7, 58.7. HRMS (ESI) *m/z* calcd for $C_{15}H_{13}NO$, $[M+H]^+$: 224.1075, found: 224.1079.

6-Benzylphenanthridine

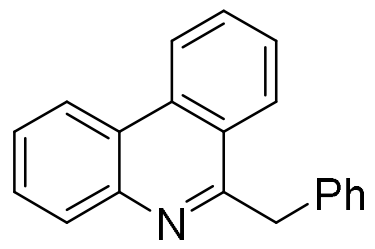

5i

White solid, 37.1 mg, 69%. ^{1}H NMR (400 MHz, Chloroform-*d*) δ 8.66 (d, *J* = 8.3 Hz, 1H), 8.60 (d, *J* = 8.1 Hz, 1H), 8.23 (d, *J* = 8.2 Hz, 2H), 7.85 – 7.74 (m, 2H), 7.69 (ddd, *J* = 8.3, 7.0, 1.4 Hz, 1H), 7.61 (ddd, *J* = 8.2, 7.0, 1.2 Hz, 1H), 7.34 (d, *J* = 7.2 Hz, 2H), 7.27 (t, *J* = 6.4 Hz, 2H), 7.19 (t, *J* = 7.3 Hz, 1H), 4.79 (s, 2H). ^{13}C NMR (101 MHz, Chloroform-*d*) δ 160.2, 143.7, 139.1, 133.3, 130.5, 129.8, 128.8, 128.59, 128.57, 127.4, 127.1, 126.8, 126.4, 125.4, 124.0, 122.6, 122.0, 43.1. HRMS (ESI) *m/z* calcd for $C_{20}H_{15}N$, $[M+H]^+$: 270.1283, found: 270.1288.

6-Phenethylphenanthridine

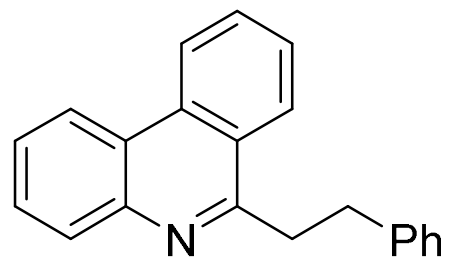

5j

White solid, 23.8 mg, 42%. ^{1}H NMR (400 MHz, Chloroform-*d*) δ 8.69 (d, *J* = 8.2 Hz, 1H), 8.59 (d, *J* = 8.1 Hz, 1H), 8.30 (d, *J* = 8.2 Hz, 1H), 8.19 (d, *J* = 8.1 Hz, 1H), 7.87 (t, *J* = 7.6 Hz, 1H), 7.80 – 7.62 (m, 3H), 7.47 – 7.30 (m, 4H), 7.26 (t, *J* = 7.5 Hz, 1H), 3.71 (t, *J* = 7.0 Hz, 1H), 3.31 (t, *J* = 7.0 Hz, 1H). ^{13}C NMR (101 MHz, Chloroform-*d*) δ 161.1, 143.7, 142.0, 133.0, 130.4, 129.6, 128.7, 128.5 (2C), 127.4, 126.5, 126.1, 126.1, 125.2, 123.7, 122.6, 122.0, 38.0, 35.1. HRMS (ESI)

m/z calcd for $C_{21}H_{17}N$, $[M+H]^+$: 284.1439, found: 284.1435.

6-(But-3-en-1-yl)phenanthridine

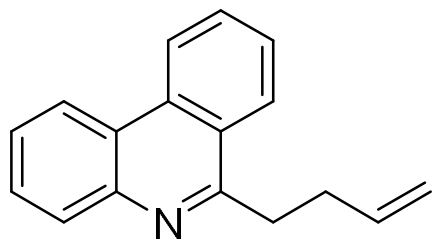

5k

White solid, 36.8 mg, 79%. ^1H NMR (400 MHz, Chloroform-*d*) δ 8.68 (d, *J* = 8.3 Hz, 1H), 8.58 (d, *J* = 8.1 Hz, 1H), 8.28 (d, *J* = 8.3 Hz, 1H), 8.15 (d, *J* = 8.1 Hz, 1H), 7.87 (t, *J* = 7.6 Hz, 1H), 7.79 – 7.69 (m, 2H), 7.65 (t, *J* = 7.5 Hz, 1H), 6.16 – 5.99 (m, 1H), 5.19 (d, *J* = 17.4 Hz, 1H), 5.07 (d, *J* = 10.0 Hz, 1H), 3.57 – 3.41 (m, 2H), 2.81 – 2.66 (m, 2H). ^{13}C NMR (101 MHz, Chloroform-*d*) δ 161.4, 143.7, 138.0, 132.9, 130.4, 129.6, 128.7, 127.4, 126.4, 126.2, 125.2, 123.7, 122.6, 122.0, 115.2, 35.5, 33.3. HRMS (ESI) *m/z* calcd for $C_{17}H_{15}N$, $[M+H]^+$: 234.1283, found: 234.1282.

Phenanthridin-6-yl(phenyl)methanone

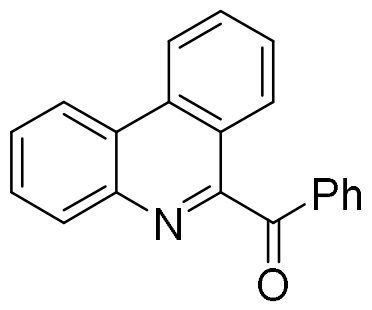

5l

White solid, 37.9 mg, 67%. ^1H NMR (400 MHz, Chloroform-*d*) δ 8.75 (d, *J* = 8.4 Hz, 1H), 8.68 (d, *J* = 7.0 Hz, 1H), 8.28 – 8.21 (m, 1H), 8.16 (d, *J* = 8.2 Hz, 1H), 8.07 (d, *J* = 7.3 Hz, 2H), 7.92 (t, *J* = 7.7 Hz, 1H), 7.87 – 7.76 (m, 2H), 7.73 – 7.61 (m, 2H), 7.50 (t, *J* = 7.7 Hz, 2H). ^{13}C NMR (101 MHz, Chloroform-*d*) δ 194.9, 157.5, 142.7, 136.1, 134.1, 133.3, 131.3, 130.9, 130.7, 129.2, 128.7, 128.2, 127.9, 127.4, 124.5, 123.8, 122.4, 122.2. HRMS (ESI) *m/z* calcd for $C_{20}H_{13}NO$, $[M+H]^+$:284.1075, found: 284.1078.

6-(1-Phenoxyethyl)phenanthridine

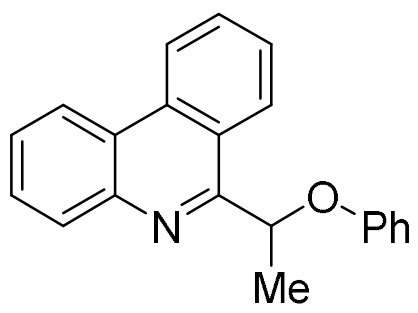

5m

White solid, 46.6 mg, 78%. ^1H NMR (400 MHz, Chloroform-*d*) δ 8.83 (d, *J* = 8.3 Hz, 1H), 8.66 (d,

J = 8.3 Hz, 1H), 8.57 (d, J = 8.0 Hz, 1H), 8.22 (d, J = 8.0 Hz, 1H), 7.84 (t, J = 7.6 Hz, 1H), 7.77 (t, J = 7.5 Hz, 1H), 7.73 – 7.66 (m, 2H), 7.18 (t, J = 7.5 Hz, 2H), 7.07 (d, J = 8.1 Hz, 2H), 6.86 (t, J = 7.3 Hz, 1H), 6.09 (q, J = 6.7 Hz, 1H), 2.02 (d, J = 6.7 Hz, 3H). ^{13}C NMR (101 MHz, Chloroform-*d*) δ 160.5, 158.0, 143.2, 133.7, 130.6, 130.1, 129.4, 128.7, 127.3, 127.2, 126.6, 124.1, 123.7, 122.6, 122.0, 120.8, 115.6, 78.9, 21.5. HRMS (ESI) *m/z* calcd for $C_{21}H_{17}NO$, $[M+H]^+$: 300.1388, found: 300.1387.

2-(Phenanthridin-6-yl)-2-phenoxyethan-1-ol

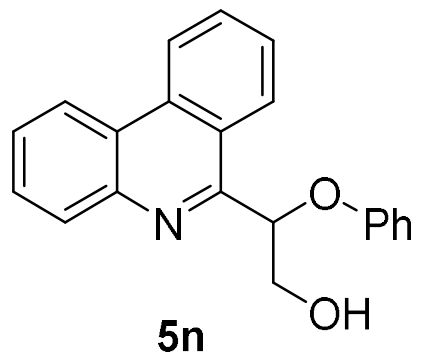

5n

White solid, 33.4 mg, 53%. ^{1}H NMR (400 MHz, Chloroform-*d*) δ 8.70 (d, J = 8.2 Hz, 1H), 8.61 (d, J = 8.1 Hz, 1H), 8.44 (d, J = 8.1 Hz, 1H), 8.20 (d, J = 8.1 Hz, 1H), 7.88 (t, J = 7.5 Hz, 1H), 7.81 – 7.66 (m, 3H), 7.27 (t, J = 6.1 Hz, 2H), 7.10 (d, J = 8.5 Hz, 2H), 6.98 (t, J = 7.2 Hz, 1H), 6.13 (t, J = 5.4 Hz, 1H), 4.52 – 4.37 (m, 2H), 3.74 (s, 1H). ^{13}C NMR (101 MHz, Chloroform-*d*) δ 157.5, 157.1, 142.8, 133.5, 131.0, 130.2, 129.7, 128.9, 127.7, 127.7, 126.0, 124.7, 124.2, 122.6, 122.1, 121.6, 115.7, 78.9, 63.4. HRMS (ESI) *m/z* calcd for $C_{21}H_{17}NO_2$, $[M+H]^+$: 316.1338, found: 316.1342.

6-(1-Phenoxypentyl)phenanthridine

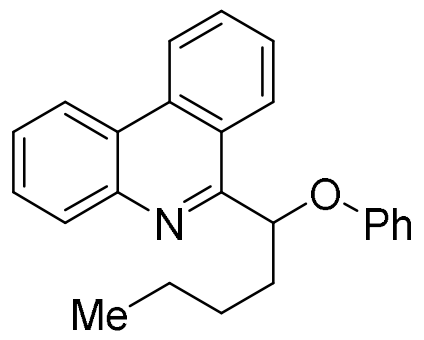

5o

White solid, 54.5 mg, 80%. ^{1}H NMR (400 MHz, Chloroform-*d*) δ 8.87 (d, J = 8.3 Hz, 1H), 8.66 (d, J = 8.3 Hz, 1H), 8.57 (d, J = 8.1 Hz, 1H), 8.24 (d, J = 8.1 Hz, 1H), 7.84 (t, J = 7.6 Hz, 1H), 7.77 (t, J = 8.1 Hz, 1H), 7.75 – 7.65 (m, 2H), 7.16 (t, J = 8.0 Hz, 2H), 7.06 (d, J = 8.0 Hz, 2H), 6.84 (t, J = 7.3 Hz, 1H), 5.86 (dd, J = 9.1, 4.9 Hz, 1H), 2.53 – 2.38 (m, 1H), 2.27 – 2.13 (m, 1H), 1.85 – 1.75 (m, 1H), 1.56 – 1.41 (m, 3H), 0.94 (t, J = 7.1 Hz, 3H). ^{13}C NMR (101 MHz, Chloroform-*d*) δ 160.4, 158.5, 143.3, 133.7, 130.6, 130.1, 129.4, 128.7, 127.3, 127.1, 126.7, 124.0, 123.9, 122.6,

122.0, 120.7, 115.6, 83.6, 35.9, 28.7, 22.6, 14.1. HRMS (ESI) *m/z* calcd for $C_{24}H_{23}NO$, $[M+H]^+$: 342.1858, found: 342.1860.

6-(Methoxy(phenyl)methyl)phenanthridine

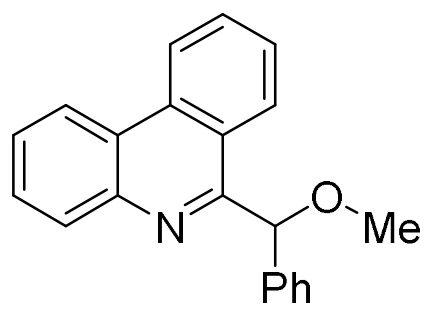

5p

White solid, 31.6 mg, 53%. ^{1}H NMR (400 MHz, Chloroform-*d*) δ 8.65 (d, *J* = 8.3 Hz, 1H), 8.60 (d, *J* = 8.2 Hz, 1H), 8.54 (d, *J* = 8.4 Hz, 1H), 8.30 (d, *J* = 8.1 Hz, 1H), 7.83 – 7.75 (m, 2H), 7.72 (t, *J* = 7.2 Hz, 1H), 7.58 – 7.51 (m, 3H), 7.31 (t, *J* = 7.2 Hz, 2H), 7.23 (t, *J* = 7.3 Hz, 1H), 6.08 (s, 1H), 3.57 (s, 3H). ^{13}C NMR (101 MHz, Chloroform-*d*) δ 159.6, 143.2, 140.4, 133.7, 130.4, 130.3, 128.7, 128.3, 127.4 (2C), 127.2, 127.1, 126.3, 124.3, 124.0, 122.3, 122.0, 88.0, 57.7. HRMS (ESI) *m/z* calcd for $C_{21}H_{17}NO$, $[M+H]^+$: 300.1388, found: 300.1389.

***tert*-Butyl 4-(phenanthridin-6-yl)piperidine-1-carboxylate**

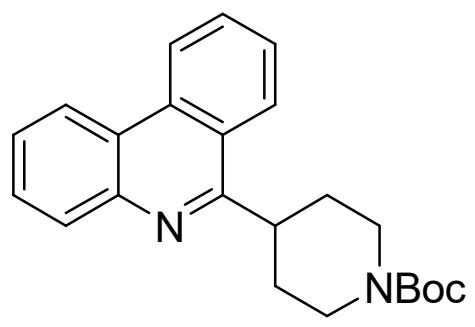

5q

White solid, 44.8 mg, 77%. ^{1}H NMR (400 MHz, Chloroform-*d*) δ 8.70 (d, *J* = 8.2 Hz, 1H), 8.57 (d, *J* = 8.1 Hz, 1H), 8.32 (d, *J* = 8.2 Hz, 1H), 8.13 (d, *J* = 8.1 Hz, 1H), 7.86 (t, *J* = 7.6 Hz, 1H), 7.77 – 7.71 (m, 2H), 7.65 (t, *J* = 7.5 Hz, 1H), 4.48 – 4.24 (m, 2H), 3.85 – 3.73 (m, 1H), 3.12 – 2.95 (m, 2H), 2.21 – 1.99 (m, 4H), 1.52 (s, 9H). ^{13}C NMR (101 MHz, Chloroform-*d*) δ 163.2, 154.9, 143.7, 133.1, 130.2, 130.0, 128.6, 127.3, 126.5, 125.2, 124.5, 123.4, 122.8, 121.9, 79.5, 40.0, 28.6. HRMS (ESI) *m/z* calcd for $C_{23}H_{26}N_2O_2$, $[M+H]^+$: 363.2073, found: 363.2073.

6-(2-Phenoxypropan-2-yl)phenanthridine

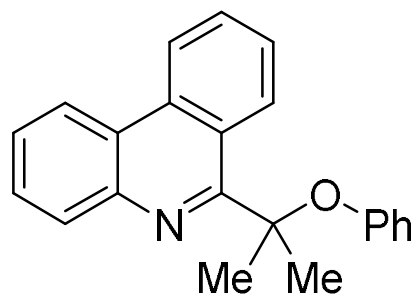

5r

White solid, 41.8 mg, 67%. ^{1}H NMR (400 MHz, Chloroform-*d*) δ 9.18 (d, *J* = 8.5 Hz, 1H), 8.67 (d, *J* = 8.3 Hz, 1H), 8.60 (d, *J* = 8.2 Hz, 1H), 8.21 (d, *J* = 9.0 Hz, 1H), 7.78 (t, *J* = 7.7 Hz, 2H), 7.71 (t, *J* = 6.9 Hz, 1H), 7.61 (t, *J* = 8.3 Hz, 1H), 7.05 – 6.96 (m, 2H), 6.78 (t, *J* = 7.3 Hz, 1H), 6.70 (d, *J* = 7.8 Hz, 2H), 2.09 (s, 6H). ^{13}C NMR (101 MHz, Chloroform-*d*) δ 163.2, 156.2, 142.9, 133.6, 130.5, 130.2, 129.1, 128.5, 128.3, 127.23, 127.17, 124.15, 124.08, 122.4, 121.9, 120.9, 118.3, 84.6, 28.7. HRMS (ESI) *m/z* calcd for $C_{22}H_{19}NO$, $[M+H]^+$: 314.1545, found: 314.1546.

6-((3*r*,5*r*,7*r*)-Adamantan-1-yl)phenanthridine

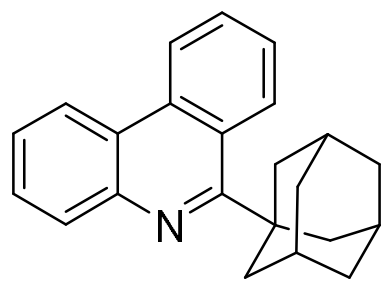

5s

White solid, 50.0 mg, 80%. ^{1}H NMR (400 MHz, Chloroform-*d*) δ 8.89 (d, *J* = 8.6 Hz, 1H), 8.72 (d, *J* = 8.3 Hz, 1H), 8.55 (d, *J* = 8.2 Hz, 1H), 8.15 (d, *J* = 8.1 Hz, 1H), 7.84 – 7.58 (m, 4H), 2.60 – 2.44 (m, 6H), 2.31 – 2.21 (m, 3H), 2.04 – 1.86 (m, 6H). ^{13}C NMR (101 MHz, Chloroform-*d*) δ 166.1, 143.2, 134.1, 130.3, 129.2, 128.4, 128.0, 126.4, 125.7, 124.5, 123.3, 123.1, 121.6, 43.0, 42.1, 37.2, 29.3. HRMS (ESI) *m/z* calcd for $C_{23}H_{23}N$, $[M+H]^+$: 314.1909, found: 314.1910.

6-((((1*R*,2*S*,5*R*)-2-isopropyl-5-methylcyclohexyl)oxy)methyl)phenanthridine

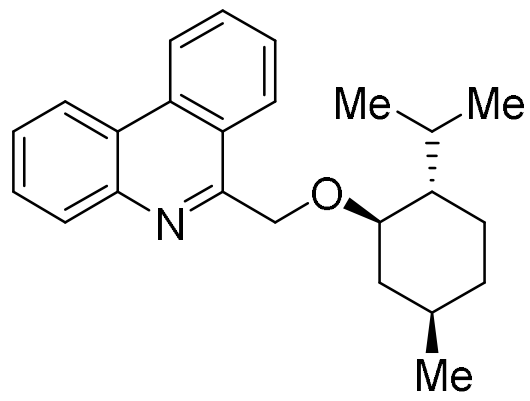

5t

White solid, 55.5 mg, 80%. ^{1}H NMR (400 MHz, Chloroform-*d*) δ 8.65 (d, *J* = 8.3 Hz, 1H), 8.59 (d, *J* = 8.1 Hz, 1H), 8.50 (d, *J* = 8.2 Hz, 1H), 8.20 (d, *J* = 8.1 Hz, 1H), 7.87 (t, *J* = 7.6 Hz, 1H), 7.79 – 7.66 (m, 3H), 5.33 (d, *J* = 11.3 Hz, 1H), 5.05 (d, *J* = 11.2 Hz, 1H), 3.34 (td, *J* = 10.5, 4.1 Hz, 1H), 2.42 (d, *J* = 11.8 Hz, 1H), 2.22 – 2.07 (m, 1H), 1.69 – 1.56 (m, 2H), 1.45 – 1.34 (m, 1H), 1.33 –

1.23 (m, 1H), 1.09 – 0.75 (m, 9H), 0.42 (d, *J* = 6.9 Hz, 3H). ^{13}C NMR (101 MHz, Chloroform-*d*) δ 158.1, 143.3, 133.1, 130.6, 130.1, 128.6, 127.6, 127.2, 127.1, 125.4, 124.5, 122.1, 122.0, 78.8, 71.6, 48.4, 40.1, 34.5, 31.5, 25.1, 22.9, 22.4, 21.1, 15.5. HRMS (ESI) *m/z* calcd for $C_{24}H_{29}NO$, $[M+H]^+$: 348.2327, found: 348.2324.

6-(2,3-Dihydrobenzo[b][1,4]dioxin-2-yl)phenanthridine

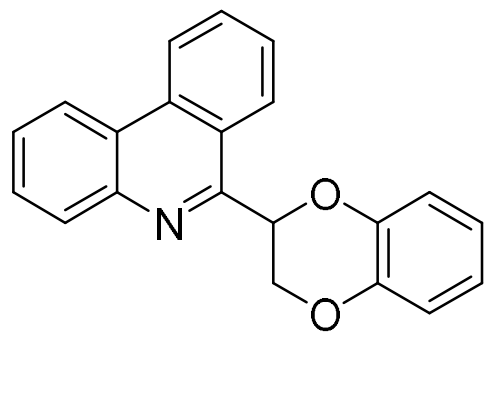

5u

White solid, 55.5 mg, 64%. ^{1}H NMR (400 MHz, Chloroform-*d*) δ 8.73 (d, *J* = 8.3 Hz, 1H), 8.63 (d, *J* = 8.0 Hz, 1H), 8.51 (d, *J* = 8.3 Hz, 1H), 8.21 (d, *J* = 7.9 Hz, 1H), 7.93 (t, *J* = 7.7 Hz, 1H), 7.83 – 7.65 (m, 3H), 7.05 (d, *J* = 7.2 Hz, 2H), 6.99 – 6.87 (m, 2H), 5.99 (dd, *J* = 6.7, 4.1 Hz, 1H), 5.00 – 4.87 (m, 2H). ^{13}C NMR (101 MHz, Chloroform-*d*) δ 154.5, 143.7, 143.6, 143.1, 133.4, 130.8, 130.6, 128.8, 127.8, 127.7, 126.2, 124.7, 124.3, 122.6, 122.0, 121.9, 121.4, 117.7, 117.3, 73.5, 67.2. HRMS (ESI) *m/z* calcd for $C_{21}H_{15}NO_2$, $[M+H]^+$: 314.1181, found: 314.1179.

(*S*)-6-(((3,7-dimethyloct-6-en-1-yl)oxy)methyl)phenanthridine

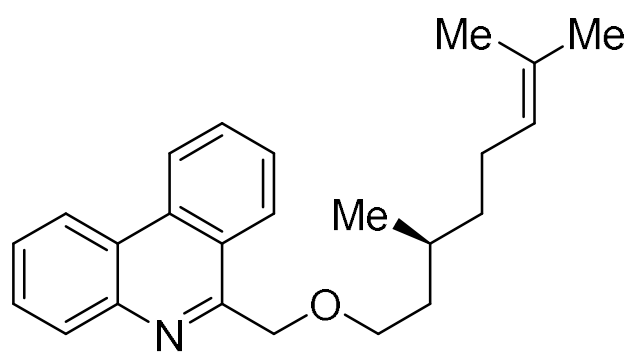

5v

White solid, 55.5 mg, 80%. ^{1}H NMR (400 MHz, Chloroform-*d*) δ 8.67 (d, *J* = 8.3 Hz, 1H), 8.60 (d, *J* = 7.9 Hz, 1H), 8.51 (d, *J* = 8.2 Hz, 1H), 8.20 (d, *J* = 8.1 Hz, 1H), 7.88 (t, *J* = 8.0 Hz, 1H), 7.82 – 7.65 (m, 3H), 5.18 (s, 2H), 5.04 (t, *J* = 7.1 Hz, 1H), 3.77 – 3.59 (m, 2H), 2.02 – 1.39 (m, 11H), 1.35 – 1.29 (m, 1H), 1.16 – 1.04 (m, 1H), 0.84 (d, *J* = 6.5 Hz, 3H). ^{13}C NMR (101 MHz, Chloroform-*d*) δ 157.7, 143.3, 133.1, 131.1, 130.7, 130.1, 128.7, 127.4, 127.2, 127.1, 125.2, 124.8, 124.4, 122.2, 122.0, 74.5, 69.2, 37.1, 36.8, 29.5, 25.8, 25.4, 19.5, 17.7. HRMS (ESI) *m/z* calcd for $C_{24}H_{29}NO$, $[M+H]^+$: 348.2327, found: 348.2328.

4-Methyl-2-(phenoxymethyl)quinoline

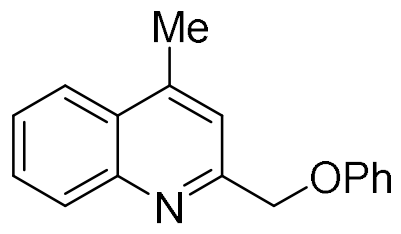

7a

Colorless oil, 45.8 mg, 90%. ^{1}H NMR (400 MHz, Chloroform-*d*) δ 8.12 (d, *J* = 8.3 Hz, 1H), 8.01 (d, *J* = 9.0 Hz, 1H), 7.81 – 7.69 (m, 1H), 7.63 – 7.51 (m, 2H), 7.37 – 7.30 (m, 2H), 7.07 (d, *J* = 7.9 Hz, 2H), 7.00 (t, *J* = 7.3 Hz, 1H), 5.37 (s, 2H), 2.73 (s, 3H). ^{13}C NMR (101 MHz, $CDCl_3$) δ 158.5, 157.6, 147.4, 145.4, 129.6, 129.51, 129.46, 127.7, 126.3, 123.8, 121.2, 119.8, 114.9, 71.3, 19.0. HRMS (ESI) *m/z* calcd for $C_{17}H_{15}NO$, $[M+H]^+$: 250.1232, found: 250.1226.

2-(Phenoxymethyl)-4-phenylpyridine

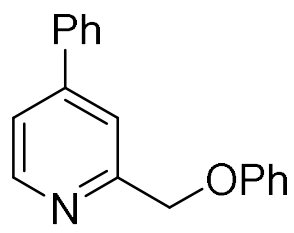

7b

Colorless oil, 37.5 mg, 72%. ^{1}H NMR (400 MHz, Chloroform-*d*) δ 8.66 (d, *J* = 5.2 Hz, 1H), 7.82 – 7.74 (m, 1H), 7.72 – 7.63 (m, 2H), 7.57 – 7.41 (m, 5H), 7.33 (dd, *J* = 8.7, 7.4 Hz, 2H), 7.10 – 6.95 (m, 3H), 5.29 (s, 2H). ^{13}C NMR (101 MHz, Chloroform-*d*) δ 158.4, 157.8, 149.7, 149.4, 138.2, 129.6, 129.2, 129.1, 127.1, 121.2, 120.7, 119.2, 114.9, 70.6. HRMS (ESI) *m/z* calcd for $C_{18}H_{15}NO$, $[M+H]^+$: 262.1232, found: 262.1229.

1-(Phenoxymethyl)isoquinoline

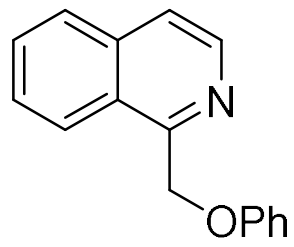

7c

Colorless oil, 42.3 mg, 90%. ^{1}H NMR (400 MHz, Chloroform-*d*) δ 8.55 (d, *J* = 5.8 Hz, 1H), 8.38 (d, *J* = 8.4 Hz, 1H), 7.88 (d, *J* = 8.1 Hz, 1H), 7.76 – 7.62 (m, 3H), 7.33 (dd, *J* = 8.7, 7.2 Hz, 2H), 7.13 (d, *J* = 7.7 Hz, 2H), 7.00 (t, *J* = 7.3 Hz, 1H), 5.68 (s, 2H). ^{13}C NMR (101 MHz, Chloroform-*d*) δ 158.6, 155.8, 141.7, 136.6, 130.3, 129.5, 127.7, 127.4, 127.3, 125.6, 121.6, 121.2, 115.0, 71.0. HRMS (ESI) *m/z* calcd for $C_{16}H_{13}NO$, $[M+H]^+$: 236.1075, found: 236.1073.

1-(Phenoxymethyl)phthalazine

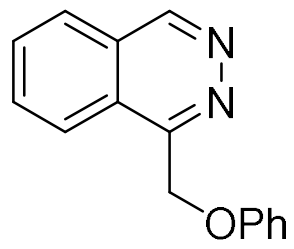

7d

Colorless oil, 27.3 mg, 58%. ^{1}H NMR (400 MHz, Chloroform-*d*) δ 9.53 (s, 1H), 8.42 (d, *J* = 6.7 Hz, 1H), 8.09 – 7.88 (m, 3H), 7.32 (dd, *J* = 8.7, 7.2 Hz, 2H), 7.14 (d, *J* = 7.8 Hz, 2H), 7.00 (t, *J* = 7.3 Hz, 1H), 5.80 (s, 2H). ^{13}C NMR (101 MHz, Chloroform-*d*) δ 158.2, 155.7, 152.0, 133.0, 132.6, 129.6, 126.9, 126.1, 124.9, 121.5, 115.0, 114.9, 69.5. HRMS (ESI) *m/z* calcd for $C_{15}H_{12}N_2O$, $[M+H]^+$: 237.1028, found: 237.1029.

2-Methyl-4-(phenoxymethyl)quinoline

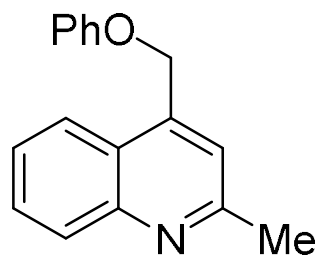

7e

Colorless oil, 20.9 mg, 58%. ^{1}H NMR (400 MHz, Chloroform-*d*) δ 8.11 (d, *J* = 8.2 Hz, 1H), 7.96 (d, *J* = 8.3 Hz, 1H), 7.78 – 7.70 (m, 1H), 7.61 – 7.53 (m, 1H), 7.50 (s, 1H), 7.36 (dd, *J* = 8.7, 7.3 Hz, 2H), 7.12 – 7.02 (m, 3H), 5.53 (s, 2H), 2.78 (s, 3H). ^{13}C NMR (101 MHz, Chloroform-*d*) δ 159.0, 158.4, 147.9, 142.0, 129.7, 129.5, 129.4, 126.0, 124.2, 122.7, 121.6, 120.3, 114.9, 66.8, 25.5. HRMS (ESI) *m/z* calcd for $C_{17}H_{15}NO$, $[M+H]^+$: 250.1232, found: 250.1232.

1,3-Dimethyl-3-(2-phenoxyethyl)indolin-2-one

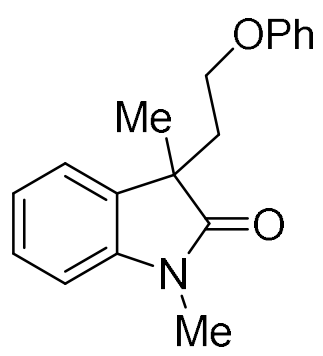

7f

Colorless oil, 51.7 mg, 92%. ^{1}H NMR (400 MHz, Chloroform-*d*) δ 7.32 – 7.17 (m, 4H), 7.10 (t, *J* = 7.8 Hz, 1H), 6.89 (t, *J* = 7.3 Hz, 1H), 6.83 (d, *J* = 7.7 Hz, 1H), 6.67 (d, *J* = 7.8 Hz, 2H), 3.79 – 3.67 (m, 2H), 3.22 (s, 3H), 2.58 – 2.48 (m, 1H), 2.31 – 2.20 (m, 1H), 1.46 (s, 3H). ^{13}C NMR (101 MHz, Chloroform-*d*) δ 180.3, 158.4, 143.2, 133.2, 129.3, 128.0, 122.7, 122.5, 120.6, 114.3, 108.2, 64.0, 46.5, 37.1, 26.3, 24.5. HRMS (ESI) *m/z* calcd for $C_{18}H_{19}NO_2$, $[M+H]^+$: 282.1494, found: 282.1487.

5-Methyl-5-(2-phenoxyethyl)indolo[2,1-a]isoquinolin-6(5H)-one

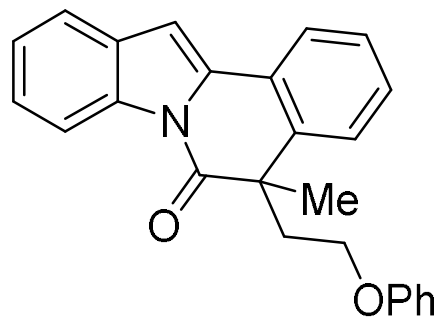

7g

White solid, 38.1 mg, 52%. ^{1}H NMR (400 MHz, Chloroform-*d*) δ 8.67 (d, *J* = 8.1 Hz, 1H), 7.83 (d, *J* = 7.8 Hz, 1H), 7.60 (d, *J* = 7.6 Hz, 1H), 7.48 – 7.34 (m, 5H), 7.03 – 6.96 (m, 3H), 6.76 (t, *J* = 7.3 Hz, 1H), 6.27 (d, *J* = 7.9 Hz, 2H), 3.83 – 3.74 (m, 1H), 3.57 – 3.50 (m, 1H), 3.17 – 3.05 (m, 1H), 2.42 – 2.33 (m, 1H), 1.80 (s, 3H). ^{13}C NMR (101 MHz, Chloroform-*d*) δ 173.0, 158.0, 137.2, 135.4, 135.3, 130.6, 129.04, 129.00, 128.8, 127.4, 126.3, 125.2, 124.9, 124.5, 123.9, 120.4, 116.8, 113.9, 103.0, 63.9, 46.5, 41.3, 29.7. HRMS (ESI) *m/z* calcd for $C_{25}H_{21}NO_2$, $[M+H]^+$: 368.1651, found: 368.1646.

2,4-Dimethyl-4-(2-phenoxyethyl)isoquinoline-1,3(2H,4H)-dione

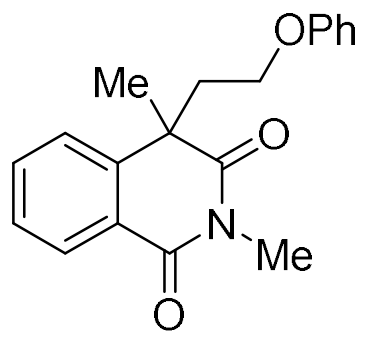

7h

Colorless oil, 46.3 mg, 75%. ^{1}H NMR (400 MHz, Chloroform-*d*) δ 8.25 (dd, *J* = 7.9, 1.2 Hz, 1H), 7.71 – 7.61 (m, 1H), 7.50 – 7.41 (m, 2H), 7.17 (dd, *J* = 8.6, 7.4 Hz, 2H), 6.88 (t, *J* = 7.3 Hz, 1H), 6.57 (d, *J* = 7.9 Hz, 2H), 3.75 (ddd, *J* = 10.2, 6.3, 4.2 Hz, 1H), 3.55 – 3.40 (m, 4H), 3.05 – 2.91 (m, 1H), 2.34 (ddd, *J* = 14.1, 5.5, 4.2 Hz, 1H), 1.71 (s, 3H). ^{13}C NMR (101 MHz, Chloroform-*d*) δ 176.3, 164.2, 158.0, 142.6, 133.8, 129.3, 129.1, 127.5, 125.3, 124.9, 120.9, 114.1, 64.0, 45.5, 41.4, 30.3, 27.3. HRMS (ESI) *m/z* calcd for $C_{19}H_{19}NO_3$, $[M+H]^+$: 310.1443, found: 310.1442.

1-Methyl-3-(phenoxymethyl)-4-phenyl-3,4-dihydroquinolin-2(1H)-one

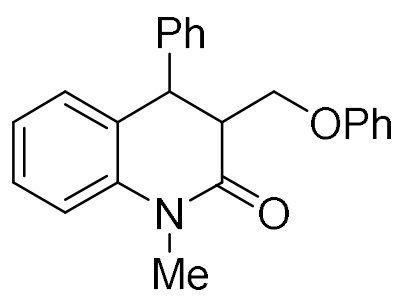

7i

Colorless oil, 23.3 mg, 34%. ^{1}H NMR (400 MHz, Chloroform-*d*) δ 7.36 – 7.30 (m, 3H), 7.25 (dd, *J* = 8.8, 7.4 Hz, 3H), 7.19 – 7.15 (m, 2H), 7.10 (d, *J* = 8.0 Hz, 1H), 7.05 – 7.00 (m, 1H), 6.98 – 6.91 (m, 2H), 6.86 (dd, *J* = 8.8, 1.1 Hz, 2H), 4.56 (d, *J* = 8.1 Hz, 1H), 4.33 (dd, *J* = 9.2, 5.8 Hz, 1H), 3.92 (dd, *J* = 9.2, 3.9 Hz, 1H), 3.47 (s, 3H), 3.28 (ddd, *J* = 8.1, 5.8, 3.9 Hz, 1H). ^{13}C NMR (101 MHz, Chloroform-*d*) δ 168.7, 158.6, 140.7, 139.7, 129.4, 129.2, 128.9, 128.3, 128.0, 127.6, 127.2, 123.3, 121.0, 114.8, 114.7, 65.1, 48.3, 43.4, 30.0. HRMS (ESI) *m/z* calcd for $C_{23}H_{21}NO_2$, $[M+Na]^+$: 366.1470, found: 366.1464.

3-(2-Phenoxyethyl)chroman-4-one

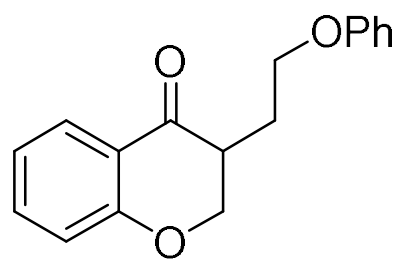

7j

Colorless oil, 22.5 mg, 42%. ^{1}H NMR (400 MHz, Chloroform-*d*) δ 7.93 (dd, *J* = 7.9, 1.8 Hz, 1H), 7.55 – 7.45 (m, 1H), 7.34 – 7.29 (m, 2H), 7.08 – 6.89 (m, 5H), 4.70 (dd, *J* = 11.5, 4.8 Hz, 1H), 4.41 – 4.31 (m, 1H), 4.24 – 4.10 (m, 2H), 3.14 – 2.99 (m, 1H), 2.49 – 2.36 (m, 1H), 2.04 – 1.87 (m, 1H). ^{13}C NMR (101 MHz, Chloroform-*d*) δ 194.1, 161.6, 158.6, 135.9, 129.5, 127.4, 121.4, 120.9, 120.6, 117.8, 114.5, 70.8, 65.5, 43.5, 25.9. HRMS (ESI) *m/z* calcd for $C_{17}H_{16}O_3$, $[M+Na]^+$: 291.0997, found: 291.0993.

(3-Phenoxyprop-1-ene-1,1-diyl)dibenzene

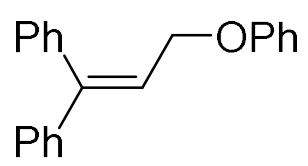

7k

Colorless oil, 36.6 mg, 64%. ^{1}H NMR (400 MHz, Chloroform-*d*) δ 7.45 – 7.28 (m, 12H), 6.97 (t, *J* = 7.3 Hz, 1H), 6.89 (d, *J* = 8.2 Hz, 2H), 6.38 (t, *J* = 6.6 Hz, 1H), 4.64 (d, *J* = 6.6 Hz, 2H). ^{13}C NMR (101 MHz, Chloroform-*d*) δ 158.6, 145.5, 141.7, 139.0, 129.7, 129.5, 128.4, 128.2, 127.82, 127.79, 127.75, 124.0, 120.9, 114.8, 65.9. HRMS (ESI) *m/z* calcd for $C_{21}H_{18}O$, $[M+H]^+$: 287.1436, found: 287.1435.

1'-Methyl-3-(phenoxymethyl)spiro[cyclobutane-1,3'-indolin]-2'-one

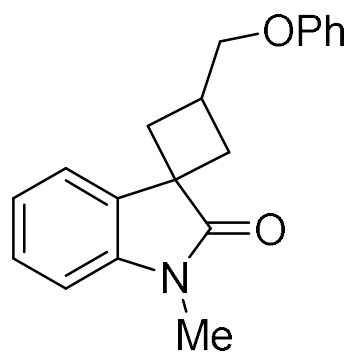

7l

Colorless oil, 41.6 mg, 71%, >20:1 d.r.. ^{1}H NMR (400 MHz, Chloroform-*d*) δ 7.58 (d, *J* = 6.7 Hz, 1H), 7.36 – 7.29 (m, 3H), 7.18 – 7.10 (m, 1H), 7.02 – 6.93 (m, 3H), 6.84 (d, *J* = 7.7 Hz, 1H), 4.24 (d, *J* = 7.1 Hz, 2H), 3.28 – 3.14 (m, 4H), 2.64 – 2.56 (m, 2H), 2.52 – 2.42 (m, 2H). ^{13}C NMR (101 MHz, Chloroform-*d*) δ 179.3, 159.0, 143.1, 134.6, 129.5, 127.9, 122.7, 122.1, 120.7, 114.6, 107.8, 71.7, 45.0, 34.3, 29.1, 26.3. HRMS (ESI) *m/z* calcd for $C_{19}H_{19}NO_2$, $[M+Na]^+$: 316.1313, found: 316.1306.

3-(Phenoxymethyl)-4-phenyl-2H-chromen-2-one

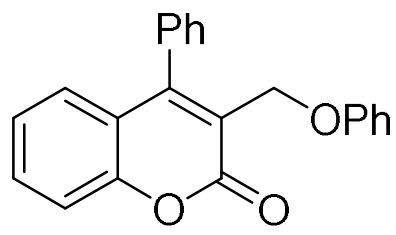

7m

White solid, 36.7 mg, 56%.^{1}H NMR (400 MHz, Chloroform-*d*) δ 7.60 – 7.53 (m, 1H), 7.53 – 7.47 (m, 3H), 7.46 – 7.37 (m, 3H), 7.28 – 7.13 (m, 4H), 6.95 (t, *J* = 7.4 Hz, 1H), 6.87 (d, *J* = 8.1 Hz, 2H), 4.77 (s, 2H). ^{13}C NMR (101 MHz, Chloroform-*d*) δ 161.3, 158.5, 155.6, 153.5, 133.3, 132.2, 129.4, 129.3, 128.63, 128.57, 128.2, 124.3, 121.2, 121.1, 120.3, 116.9, 115.1, 63.3. HRMS (ESI) *m/z* calcd for $C_{22}H_{16}O_3$, $[M+Na]^+$: 351.0997, found: 351.0992.

3-(Phenoxymethyl)-2-phenyl-4H-thiochromen-4-one

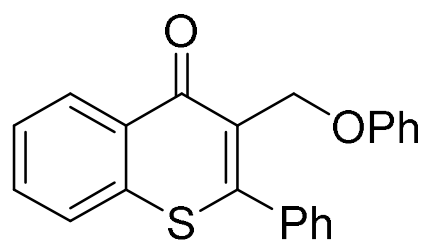

7n

White solid, 61.9 mg, 90%. ^{1}H NMR (400 MHz, Chloroform-*d*) δ 8.63 (dd, *J* = 8.1, 1.5 Hz, 1H), 7.71 – 7.57 (m, 5H), 7.51 – 7.41 (m, 3H), 7.27 (dd, *J* = 8.6, 7.4 Hz, 2H), 7.01 – 6.91 (m, 3H), 4.94 (s, 2H). ^{13}C NMR (101 MHz, $CDCl_3$) δ 179.6, 158.7, 155.4, 137.2, 135.7, 131.7, 130.7, 130.1, 129.5, 129.4, 129.3, 128.7, 128.6, 127.9, 125.8, 120.9, 115.1, 62.9. HRMS (ESI) *m/z* calcd for $C_{22}H_{16}O_2S$, $[M+Na]^+$: 367.0769, found: 367.0766.

6-(Phenoxymethyl)phenanthridine 5-oxide

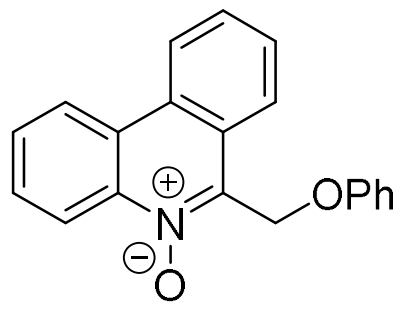

8

White solid, 45.7 mg, 76%. ^{1}H NMR (400 MHz, Chloroform-*d*) δ 8.58 (d, *J* = 7.9 Hz, 1H), 8.31 (d, *J* = 8.1 Hz, 1H), 8.25 (d, *J* = 7.6 Hz, 1H), 7.83 (t, *J* = 7.3 Hz, 1H), 7.69 – 7.54 (m, 2H), 7.42 – 7.31 (m, 4H), 7.20 – 7.06 (m, 3H), 6.03 (s, 2H). ^{13}C NMR (101 MHz, Chloroform-*d*) δ 157.9, 157.1, 137.3, 133.3, 132.9, 129.9, 129.7, 128.6, 128.1, 126.0, 123.4, 122.9, 122.8, 122.1, 118.1, 115.8, 113.8, 96.6. HRMS (ESI) *m/z* calcd for $C_{20}H_{15}NO_2$, $[M+H]^+$: 302.1181, found: 302.1175.

1-Chloro-6-methyl-6-(phenoxymethyl)-5,6-dihydrophenanthridine

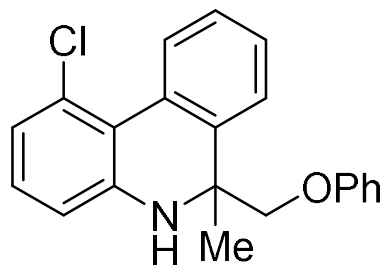

9

White solid, 57.5 mg, 86%. ^{1}H NMR (400 MHz, Chloroform-*d*) δ 8.58 (d, *J* = 7.7 Hz, 1H), 7.47 – 7.35 (m, 3H), 7.25 (d, *J* = 8.2 Hz, 2H), 7.03 – 6.89 (m, 3H), 6.85 (d, *J* = 8.0 Hz, 2H), 6.66 (d, *J* = 7.8 Hz, 1H), 4.57 (s, 1H), 4.12 (d, *J* = 9.0 Hz, 1H), 3.80 (d, *J* = 9.0 Hz, 1H), 1.77 (s, 3H). ^{13}C NMR (101 MHz, Chloroform-*d*) δ 158.7, 146.1, 138.6, 131.3, 129.9, 129.5, 128.9, 127.7, 127.4, 127.0, 123.7, 122.4, 121.1, 120.0, 114.73, 114.68, 71.1, 55.6, 23.5. HRMS (ESI) *m/z* calcd for $C_{21}H_{18}ClNO$, $[M+H]^+$: 336.1155, found: 336.1154.

2-Chloro-6-(phenoxymethyl)-5,6-dihydrophenanthridine

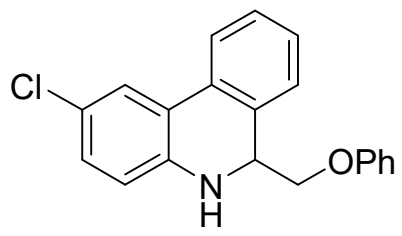

10

White solid, 38.5 mg, 60%. ^{1}H NMR (400 MHz, Chloroform-*d*) δ 7.74 (d, *J* = 7.8 Hz, 1H), 7.66 (d, *J* = 2.3 Hz, 1H), 7.42 (t, *J* = 7.6 Hz, 1H), 7.36 – 7.30 (m, 2H), 7.28 – 7.21 (m, 2H), 7.06 (dd, *J* = 8.5, 2.3 Hz, 1H), 6.98 (t, *J* = 7.3 Hz, 1H), 6.88 (d, *J* = 8.1 Hz, 2H), 6.64 (d, *J* = 8.5 Hz, 1H), 4.89 –

4.76 (m, 2H), 4.10 (t, *J* = 9.7 Hz, 1H), 3.86 (dd, *J* = 9.3, 3.3 Hz, 1H). ^{13}C NMR (101 MHz, Chloroform-*d*) δ 158.4, 141.3, 131.4, 130.8, 129.6, 128.9, 128.7, 128.0, 127.2, 123.9, 123.2, 122.7, 122.1, 121.3, 117.2, 114.6, 69.6, 55.1. HRMS (ESI) *m/z* calcd for $C_{20}H_{16}ClNO$, $[M+H]^+$: 322.0999, found: 322.0997.

6-Methyl-5,6-dihydrophenanthridine

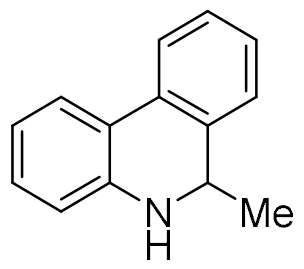

11

Colorless oil, 19.9 mg, 51%. ^{1}H NMR (400 MHz, Chloroform-*d*) δ 7.79 – 7.67 (m, 2H), 7.34 (t, *J* = 7.6 Hz, 1H), 7.26 (d, *J* = 7.3 Hz, 1H), 7.19 – 7.09 (m, 2H), 6.86 (t, *J* = 7.5 Hz, 1H), 6.69 (d, *J* = 7.9 Hz, 1H), 4.57 (q, *J* = 6.5 Hz, 1H), 3.98 (s, 1H), 1.47 (d, *J* = 6.5 Hz, 3H). ^{13}C NMR (101 MHz, Chloroform-*d*) δ 144.0, 137.2, 131.0, 128.9, 127.4, 127.3, 125.3, 123.4, 122.5, 121.3, 119.0, 115.4, 50.9, 22.8. HRMS (ESI) *m/z* calcd for $C_{14}H_{13}N$, $[M+H]^+$: 196.1126, found: 196.1130.

Phenanthridin-6-ylmethanol

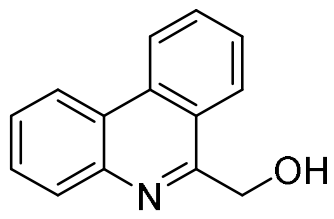

13

White solid, 26.3 mg, total yield: 63%. ^{1}H NMR (400 MHz, Chloroform-*d*) δ 8.69 (d, *J* = 8.3 Hz, 1H), 8.60 (d, *J* = 8.0 Hz, 1H), 8.19 (d, *J* = 8.1 Hz, 1H), 8.02 (d, *J* = 8.2 Hz, 1H), 7.91 (t, *J* = 8.2 Hz, 1H), 7.82 – 7.65 (m, 3H), 5.32 (s, 2H), 1.73 (s, 1H). ^{13}C NMR (101 MHz, Chloroform-*d*) δ 157.4, 142.2, 132.8, 131.2, 129.3, 129.0, 127.7, 127.0, 124.1, 124.0, 123.4, 122.7, 122.2, 61.6. HRMS (ESI) *m/z* calcd for $C_{14}H_{13}N$, $[M+H]^+$: 210.0919, found: 210.0911.

3-(2-Hydroxyethyl)-1,3-dimethylindolin-2-one

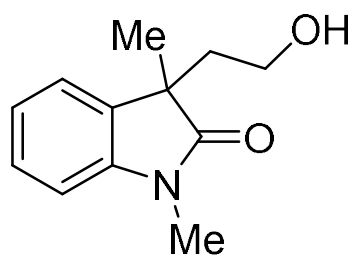

15

White solid, 38.1 mg, total yield for two steps: 62%. ^{1}H NMR (400 MHz, Chloroform-*d*) δ 7.29 (td, *J* = 7.7, 1.3 Hz, 1H), 7.19 (dd, *J* = 7.3, 1.3 Hz, 1H), 7.09 (td, *J* = 7.5, 1.0 Hz, 1H), 6.87 (d, *J* = 7.7 Hz, 1H), 3.64 (ddd, *J* = 11.3, 6.8, 5.6 Hz, 1H), 3.45 (ddd, *J* = 11.3, 6.7, 5.7 Hz, 1H), 3.23 (s, 3H), 2.18 (ddd, *J* = 14.2, 6.7, 5.6 Hz, 1H), 2.00 (ddd, *J* = 14.2, 6.8, 5.7 Hz, 1H), 1.41 (s, 3H). ^{13}C NMR (101 MHz, Chloroform-*d*) δ 181.6, 142.9, 134.1, 128.0, 122.8, 122.5, 108.3, 59.3, 47.0, 40.1, 26.4, 23.6. HRMS (ESI) *m/z* calcd for $C_{12}H_{15}NO_2$, $[M+H]^+$: 206.1181, found: 206.1184.

3a,8-Dimethyl-3,3a,8,8a-tetrahydro-2H-furo[2,3-b]indole

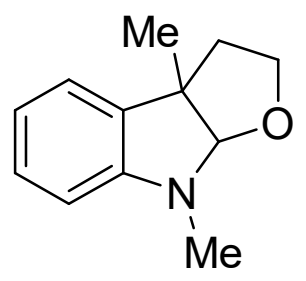

16

Colorless oil, 29.8 mg, 85%. ^{1}H NMR (400 MHz, Chloroform-*d*) δ 7.13 (t, *J* = 7.7 Hz, 1H), 7.08 (d, *J* = 7.2 Hz, 1H), 6.71 (t, *J* = 7.4 Hz, 1H), 6.40 (d, *J* = 7.8 Hz, 1H), 5.10 (s, 1H), 3.98 (ddd, *J* = 8.8, 7.1, 1.6 Hz, 1H), 3.49 (ddd, *J* = 11.1, 8.6, 5.3 Hz, 1H), 2.95 (s, 3H), 2.22 – 2.04 (m, 2H), 1.49 (s, 3H). ^{13}C NMR (101 MHz, $CDCl_3$) δ 150.5, 134.5, 128.1, 122.5, 117.3, 105.0, 104.9, 67.4, 52.3, 41.7, 30.9, 24.8. HRMS (ESI) *m/z* calcd for $C_{12}H_{15}NO$, $[M+H]^+$: 190.1232, found: 190.1226.

3-(3-Hydroxy-2-(2-methoxyphenoxy)propyl)-1,3-dimethylindolin-2-one

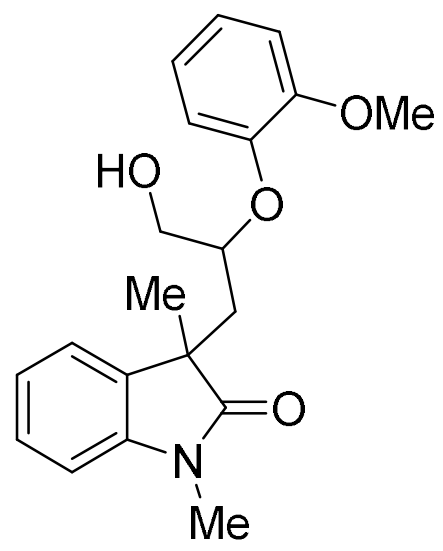

17

White solid, 42.9 mg, 63%. ^{1}H NMR (400 MHz, Chloroform-*d*) δ 7.34 (t, *J* = 7.6 Hz, 1H), 7.26 (d, *J* = 8.2 Hz, 1H), 7.13 (t, *J* = 7.5 Hz, 1H), 6.93 (t, *J* = 7.7 Hz, 1H), 6.83 (d, *J* = 7.8 Hz, 2H), 6.71 (t, *J* = 7.3 Hz, 1H), 6.09 (d, *J* = 7.5 Hz, 1H), 3.89 – 3.75 (m, 4H), 3.40 (dd, *J* = 12.4, 3.1 Hz, 1H), 3.19 (dd, *J* = 11.8, 1.8 Hz, 1H), 3.08 (s, 3H), 2.93 (dd, *J* = 14.6, 9.8 Hz, 1H), 2.03 (dd, *J* = 14.6, 3.8 Hz, 2H), 1.47 (s, 3H). ^{13}C NMR (101 MHz, Chloroform-*d*) δ 180.6, 151.6, 146.6, 143.8, 133.4, 128.1, 123.3, 122.9, 122.3, 121.3, 119.5, 112.1, 108.2, 80.2, 62.9, 56.0, 46.3, 39.2, 26.1, 25.2.

HRMS (ESI) *m/z* calcd for $C_{20}H_{23}NO_4$, $[M+Na]^+$: 364.1525, found: 364.1520.

3-(2-(2,6-dimethoxyphenoxy)-3-hydroxypropyl)-1,3-dimethylindolin-2-one

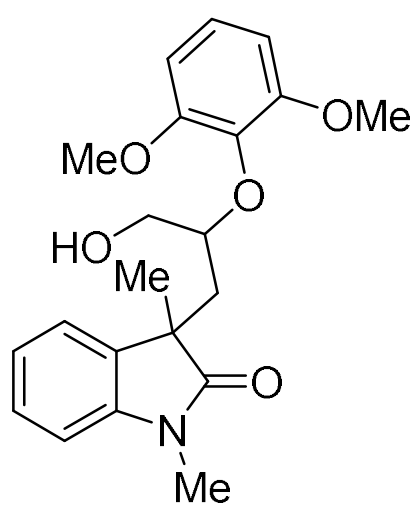

18

White solid, 39.3 mg, 53%. ^{1}H NMR (400 MHz, Chloroform-*d*) δ 7.29 (t, *J* = 6.3 Hz, 2H), 7.08 (t, *J* = 7.5 Hz, 1H), 6.97 (t, *J* = 8.4 Hz, 1H), 6.86 (d, *J* = 7.9 Hz, 1H), 6.53 (d, *J* = 8.4 Hz, 2H), 4.12 (td, *J* = 4.9, 2.5 Hz, 1H), 3.74 (s, 6H), 3.46 – 3.29 (m, 2H), 3.18 (s, 3H), 3.02 – 2.89 (m, 1H), 2.76 (dd, *J* = 14.6, 7.1 Hz, 1H), 2.21 (dd, *J* = 14.6, 4.9 Hz, 1H), 1.49 (s, 3H). ^{13}C NMR (101 MHz, Chloroform-*d*) δ 180.3, 153.7, 143.6, 134.3, 134.2, 127.7, 123.8, 123.0, 122.2, 107.9, 105.4, 78.3, 63.2, 56.1, 46.5, 39.6, 26.2, 24.7. HRMS (ESI) *m/z* calcd for $C_{21}H_{25}NO_5$, $[M+Na]^+$: 394.1630, found: 394.1627.

5. NMR spectra

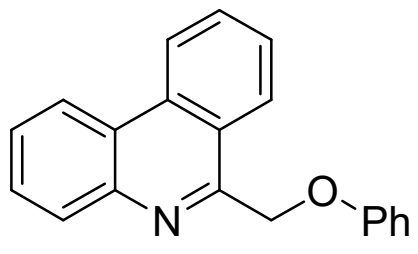

4a

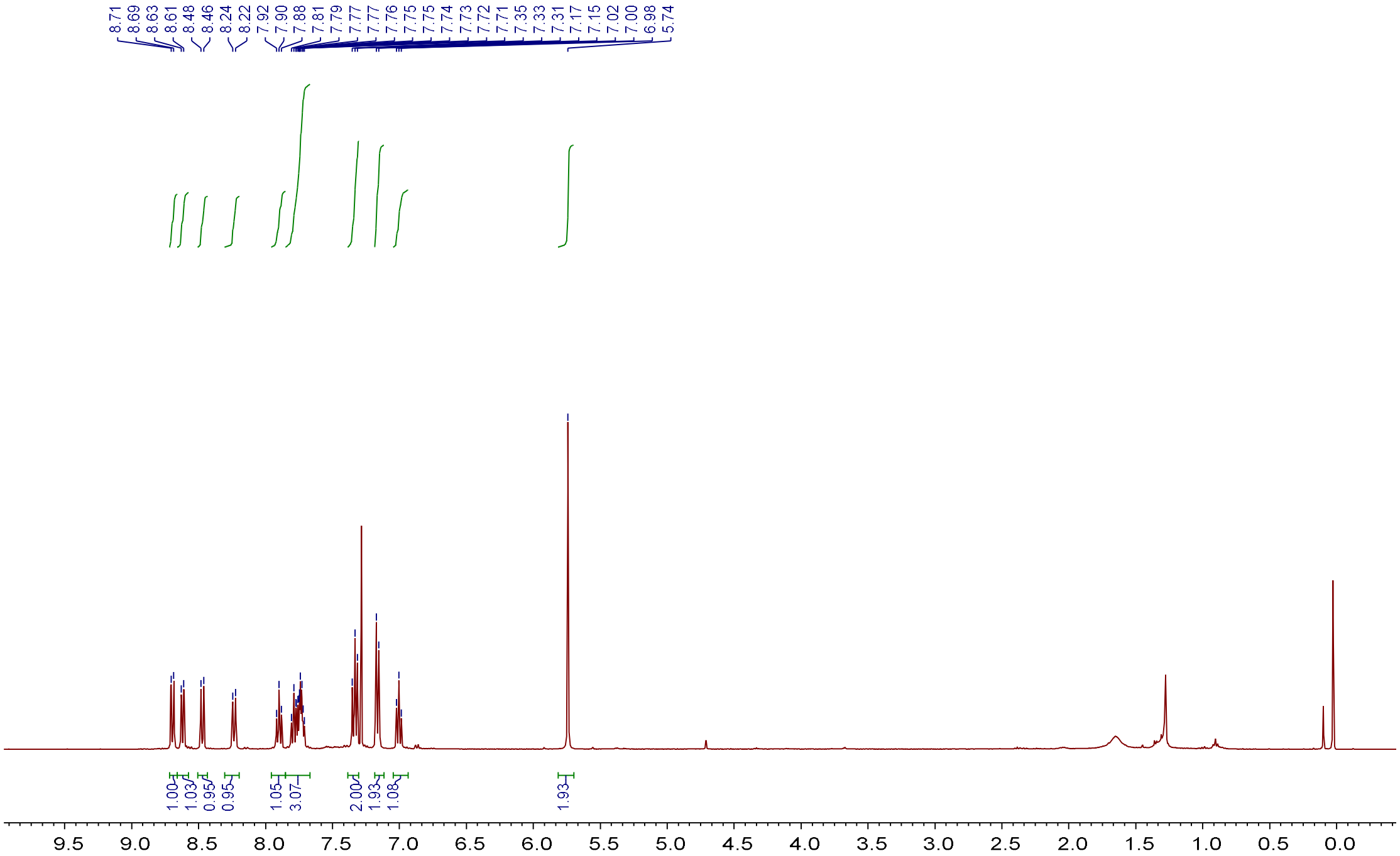

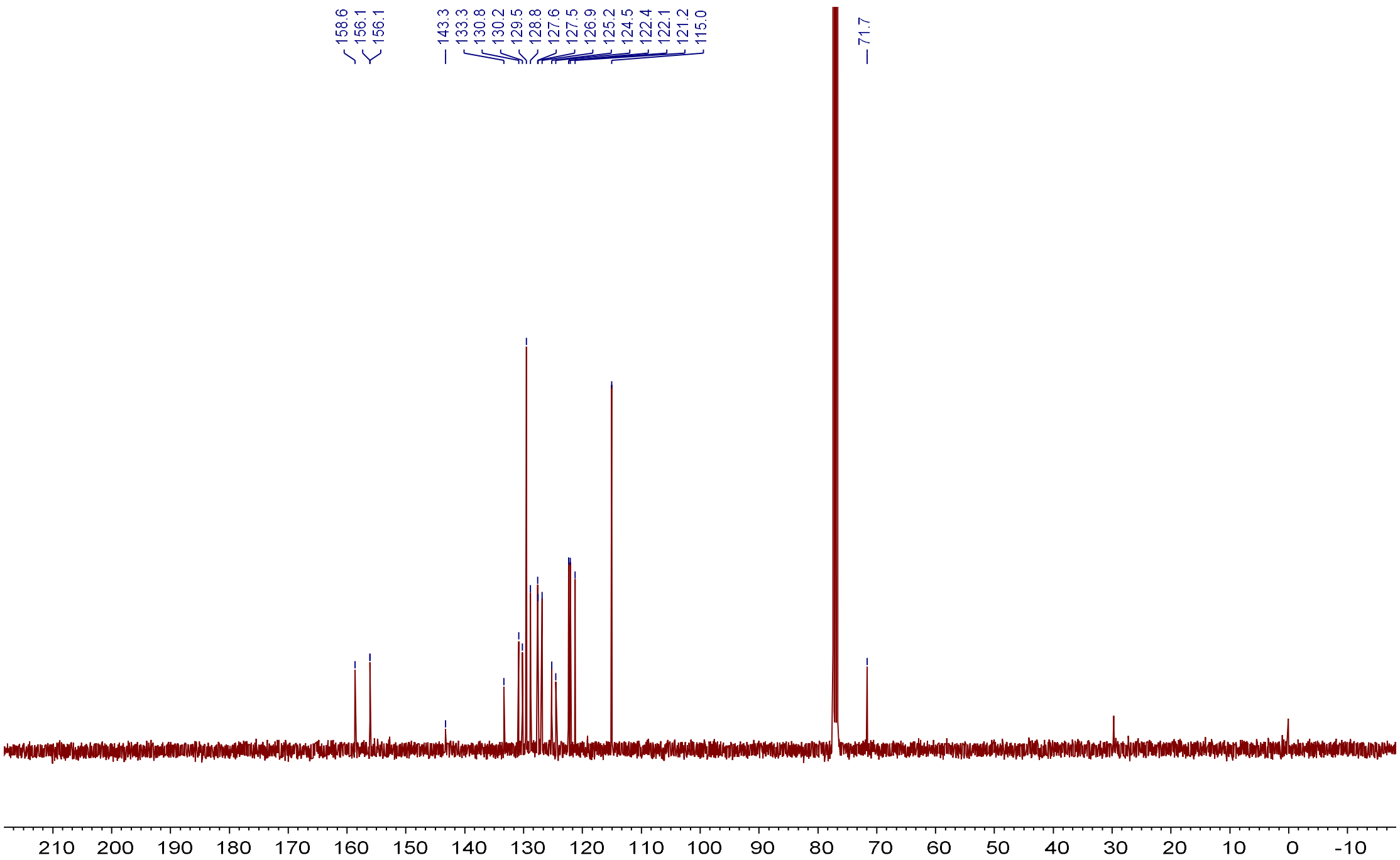

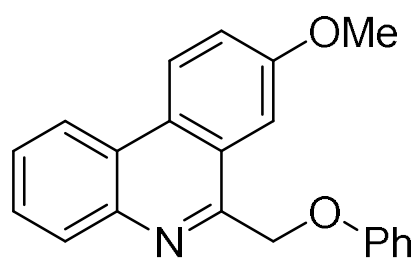

4b

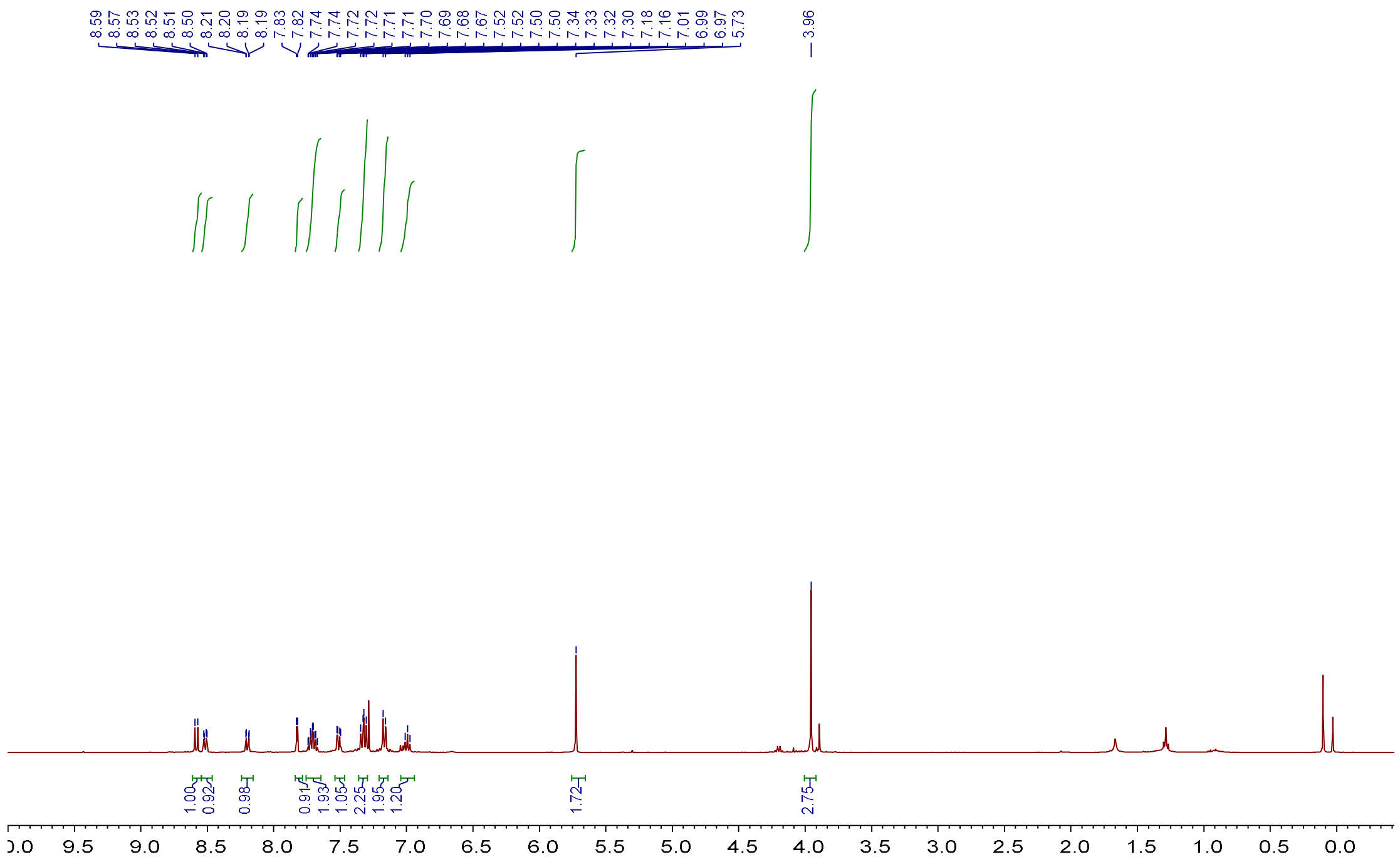

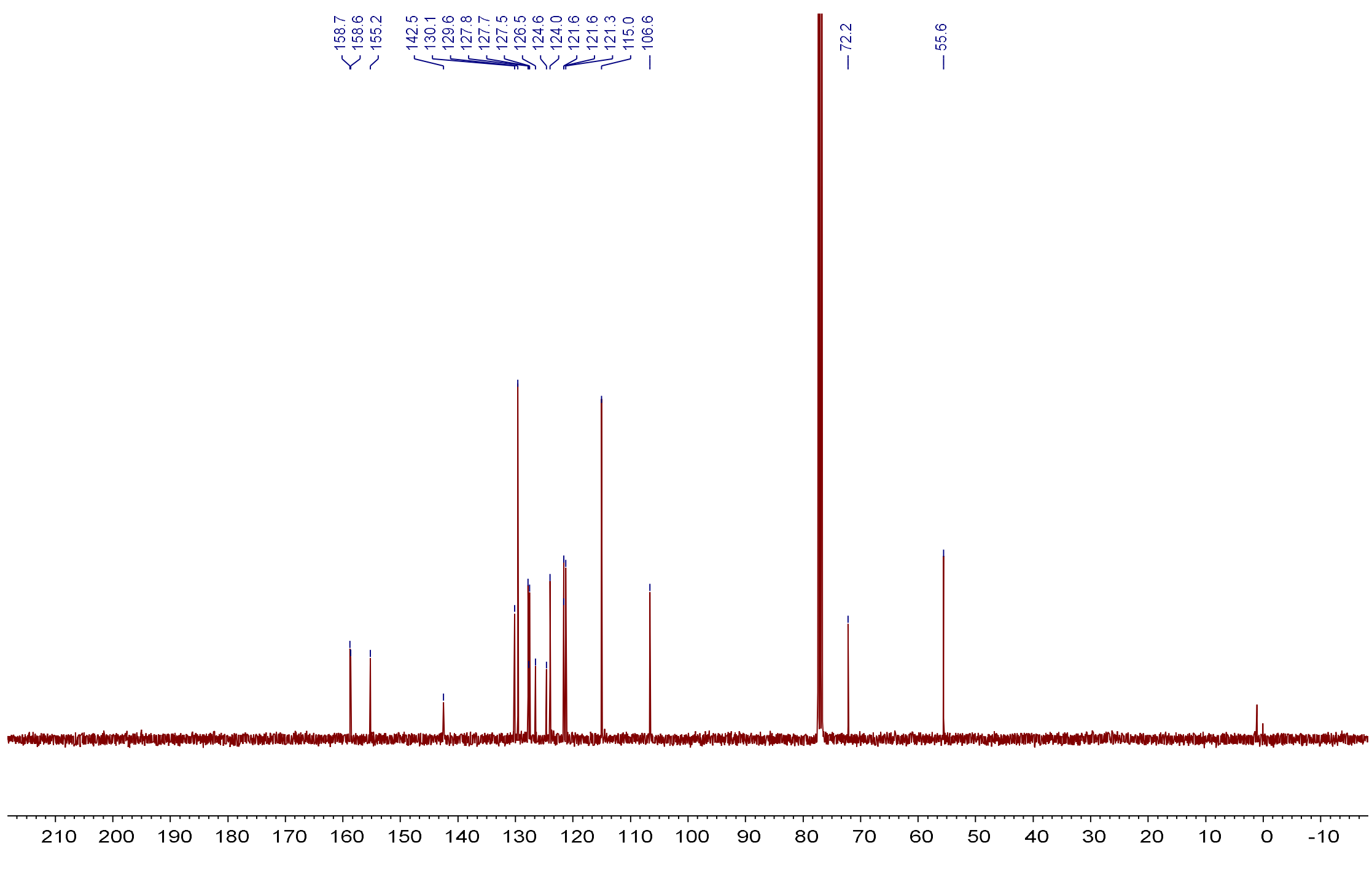

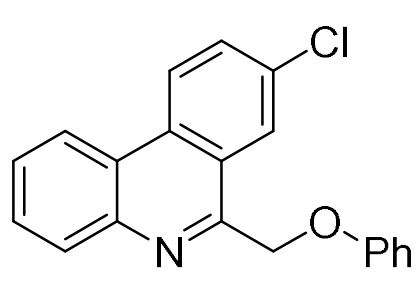

4c

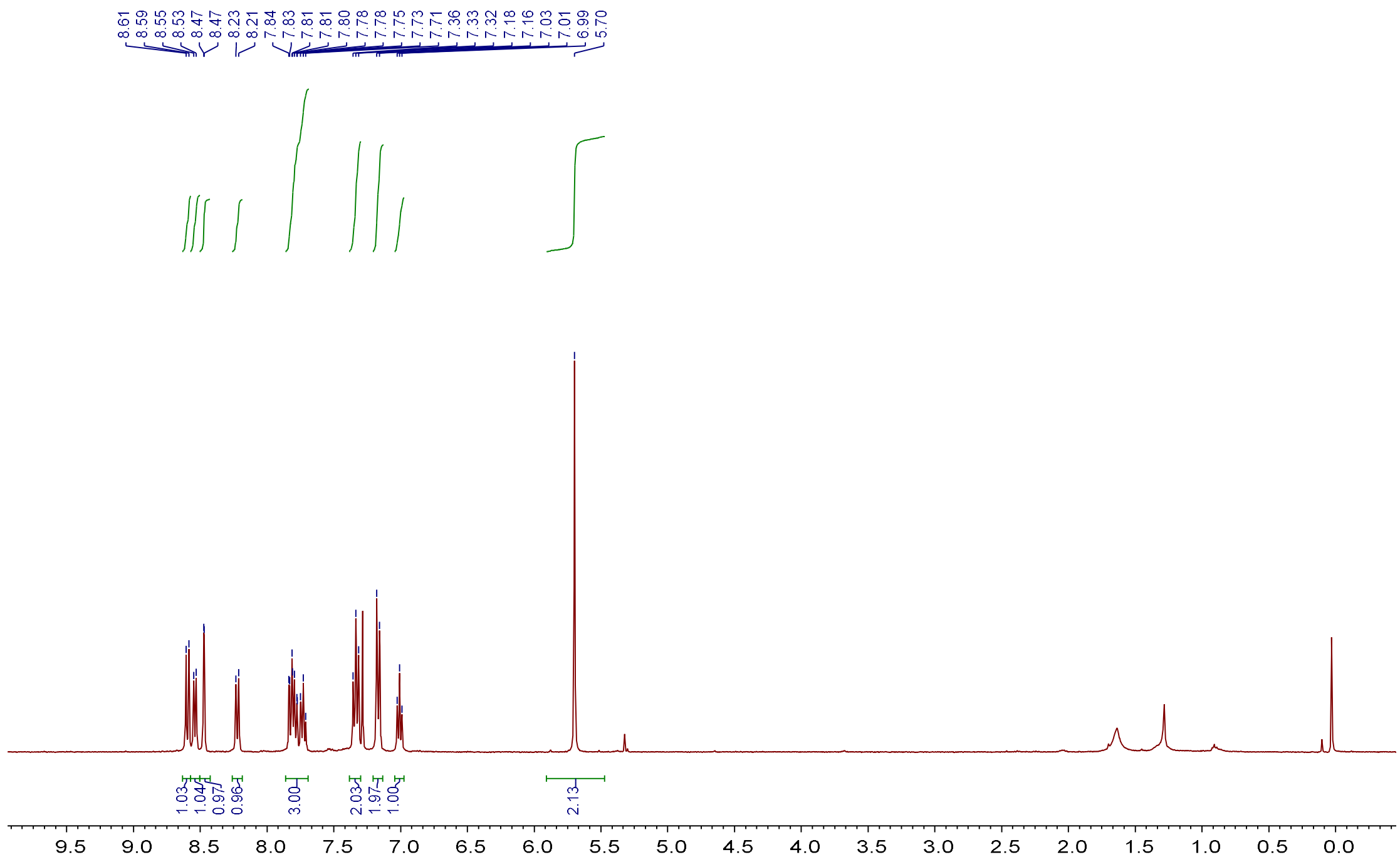

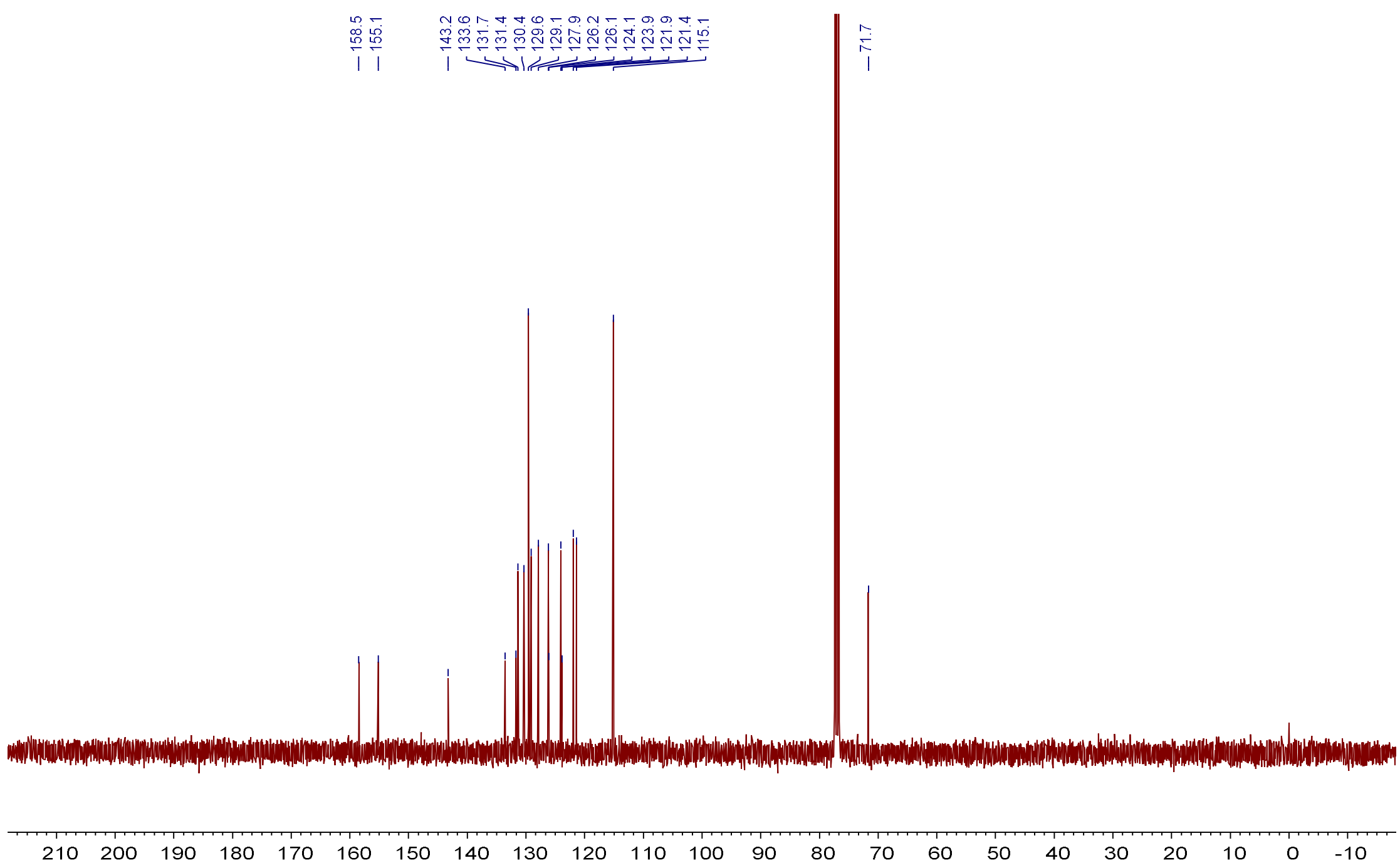

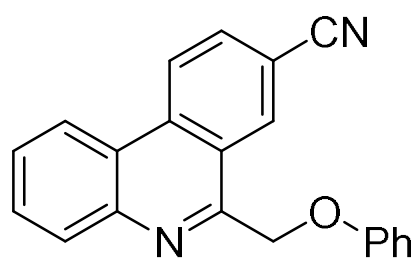

CN
N
O
Ph

4d

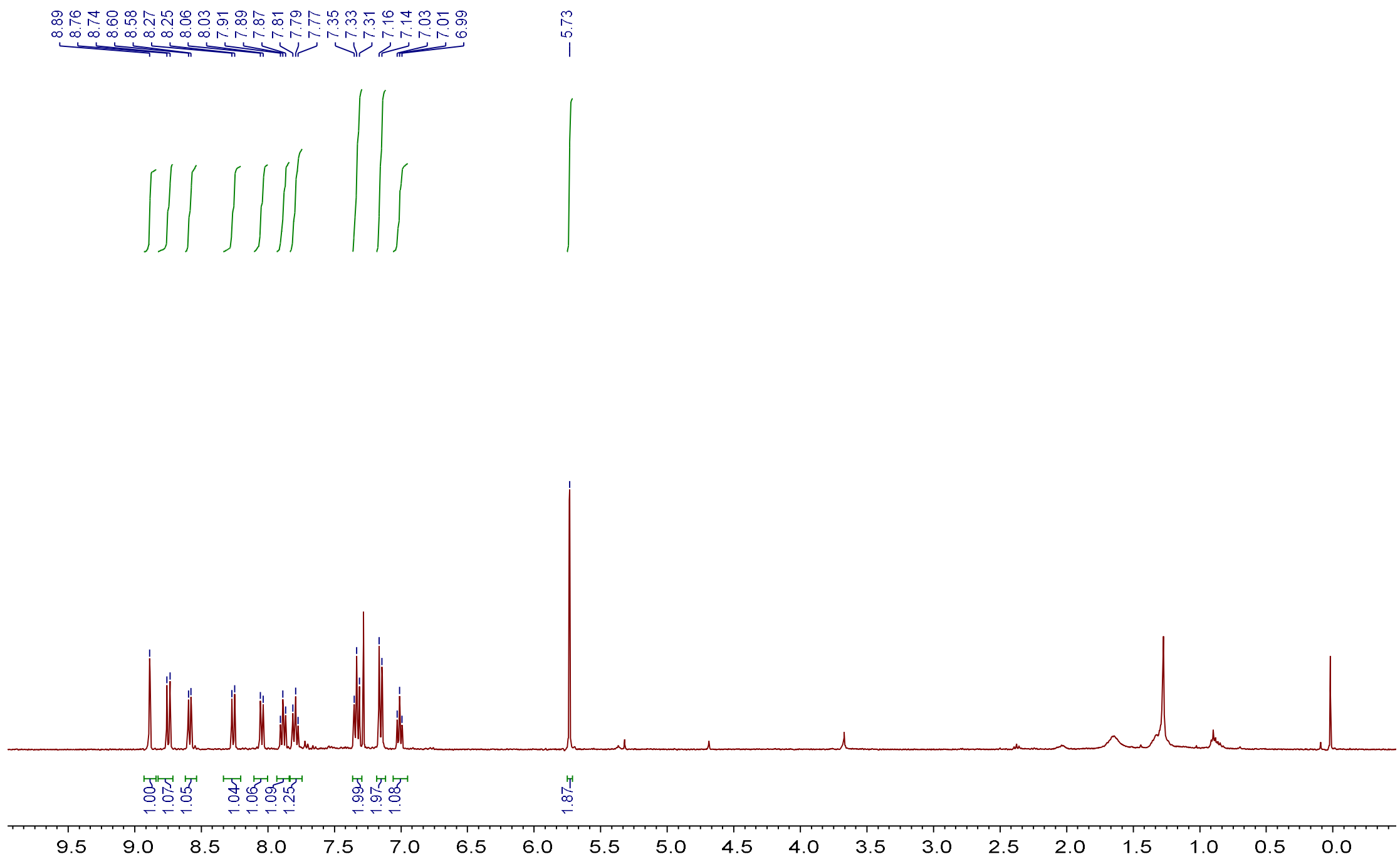

8.89
8.76
8.74
8.60
8.58
8.27
8.25
8.06
8.03
7.91
7.89
7.87
7.81
7.79
7.77
7.35
7.33
7.31
7.16
7.14
7.03
7.01
6.99
5.73
1.00
1.07
1.05
1.04
1.06
1.09
1.25
1.99
1.97
1.08
1.87
9.5 9.0 8.5 8.0 7.5 7.0 6.5 6.0 5.5 5.0 4.5 4.0 3.5 3.0 2.5 2.0 1.5 1.0 0.5 0.0

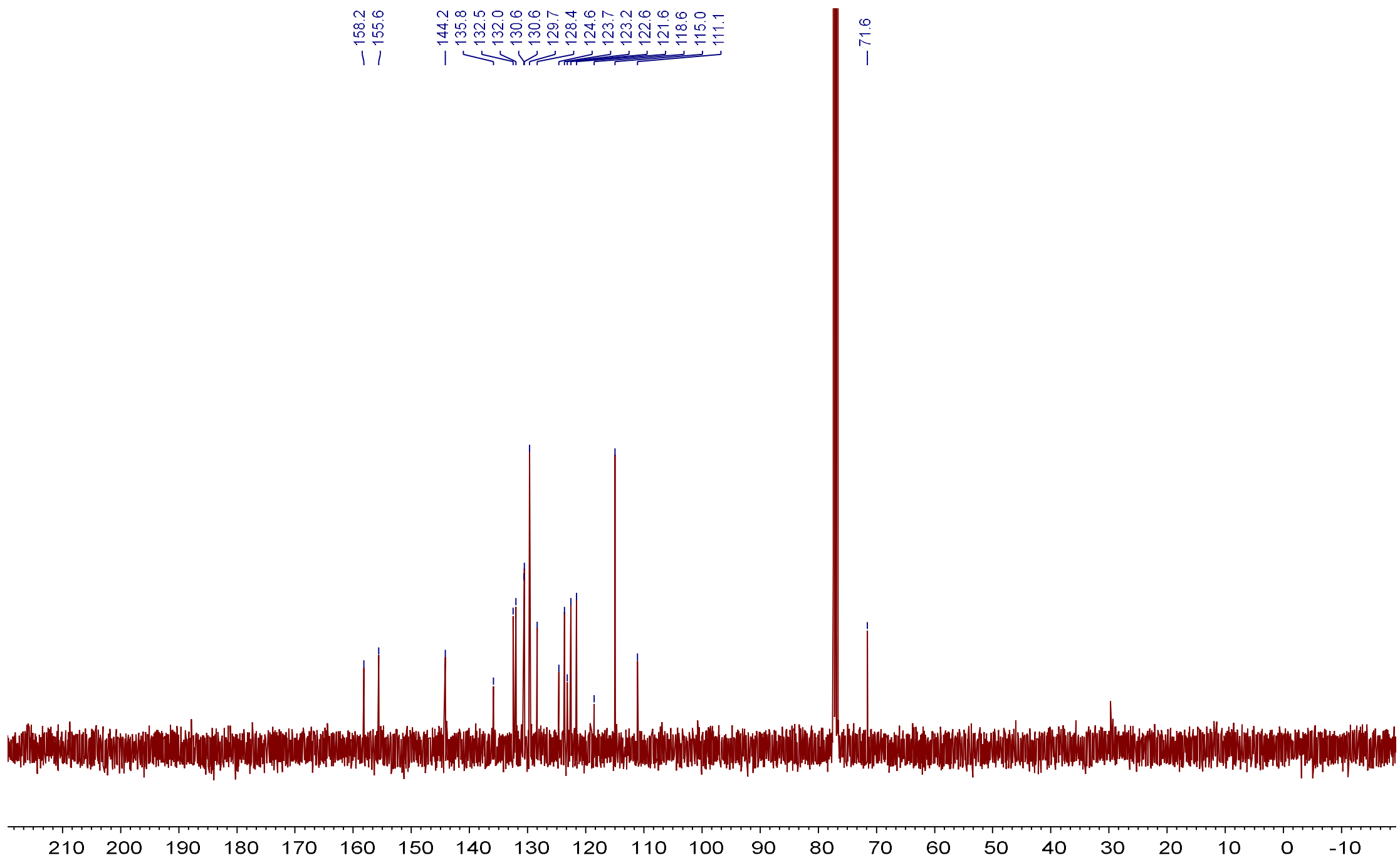

158.2
155.6
144.2
135.8
132.5
132.0
130.6
130.6
129.7
128.4
124.6
123.7
123.2
122.6
121.6
118.6
115.0
111.1
71.6
210 200 190 180 170 160 150 140 130 120 110 100 90 80 70 60 50 40 30 20 10 0 -10

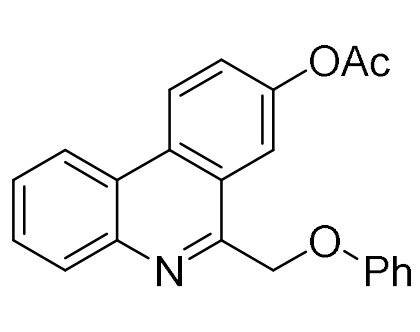
OAc
N
O
Ph

4e

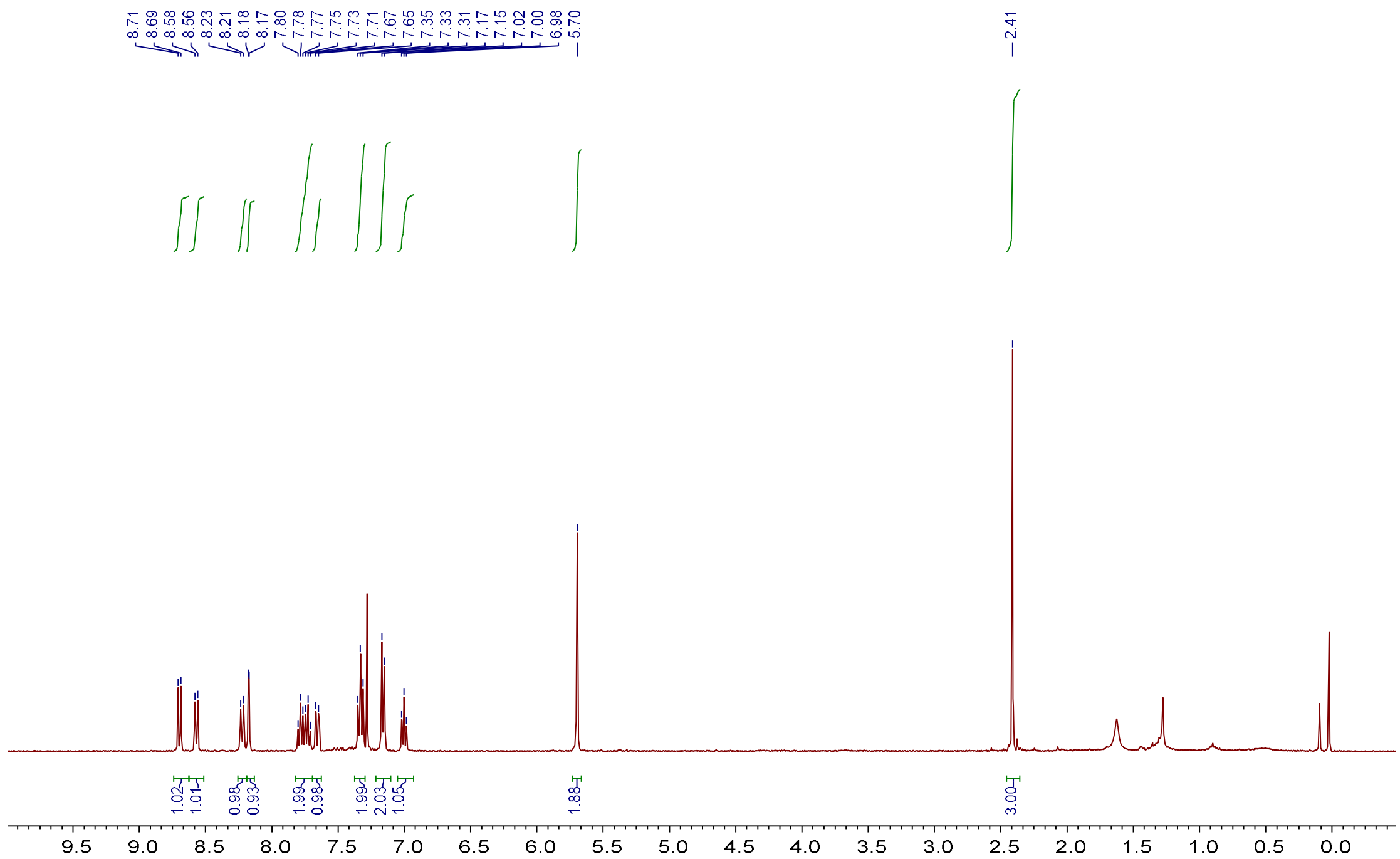
8.71
8.69
8.58
8.56
8.23
8.21
8.18
8.17
7.80
7.78
7.77
7.75
7.73
7.71
7.67
7.65
7.35
7.33
7.31
7.17
7.15
7.02
7.00
6.98
5.70
2.41
1.02
1.01
0.98
0.93
1.99
0.98
1.99
2.03
1.05
1.88
3.00
9.5 9.0 8.5 8.0 7.5 7.0 6.5 6.0 5.5 5.0 4.5 4.0 3.5 3.0 2.5 2.0 1.5 1.0 0.5 0.0

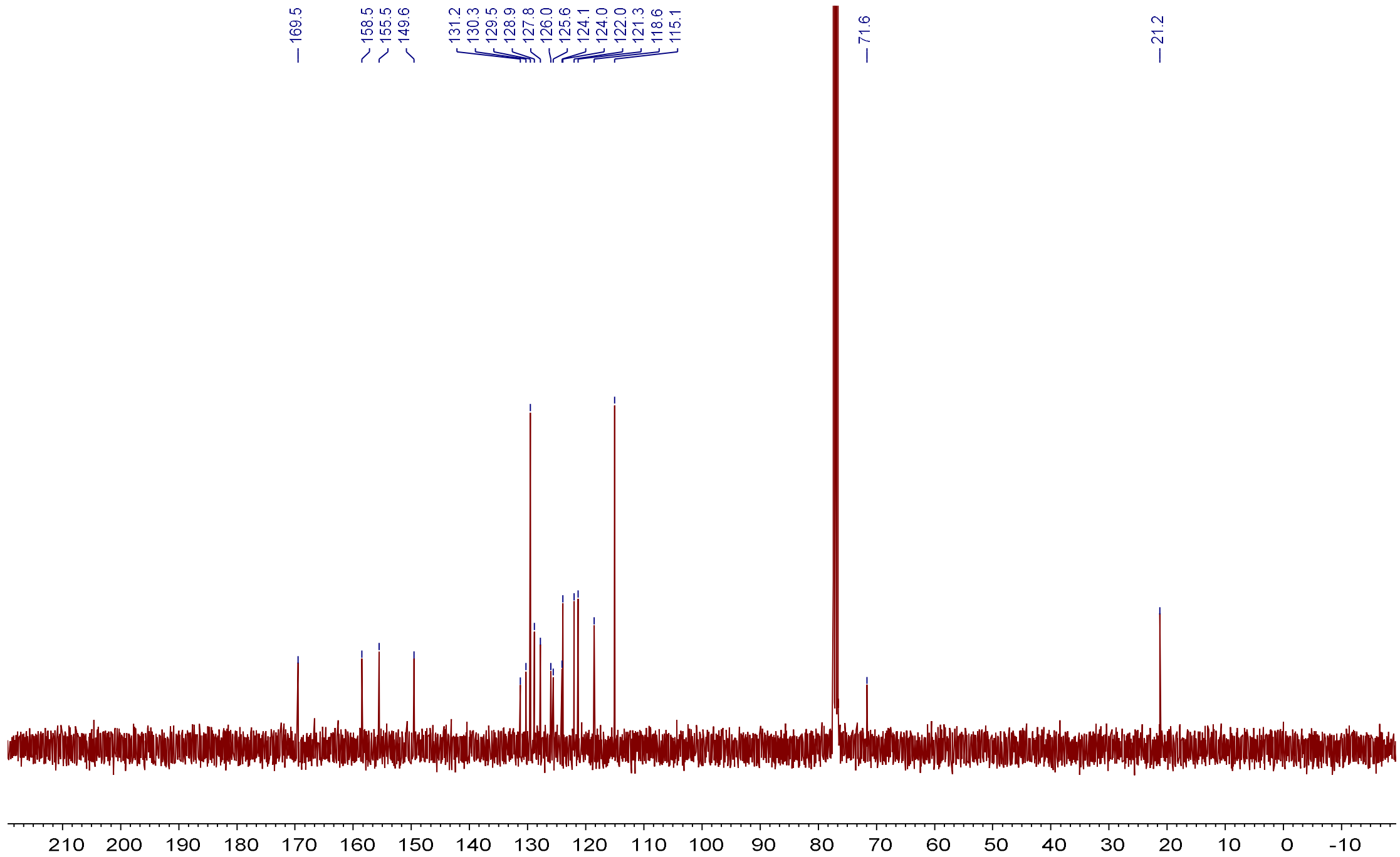
169.5
158.5
155.5
149.6
131.2
130.3
129.5
128.9
127.8
126.0
125.6
124.1
124.0
122.0
121.3
118.6
115.1
71.6
21.2
210 200 190 180 170 160 150 140 130 120 110 100 90 80 70 60 50 40 30 20 10 0 -10

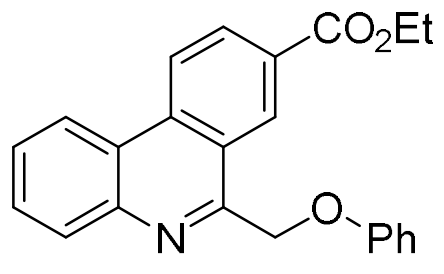
CO2Et
N
O
Ph

4f

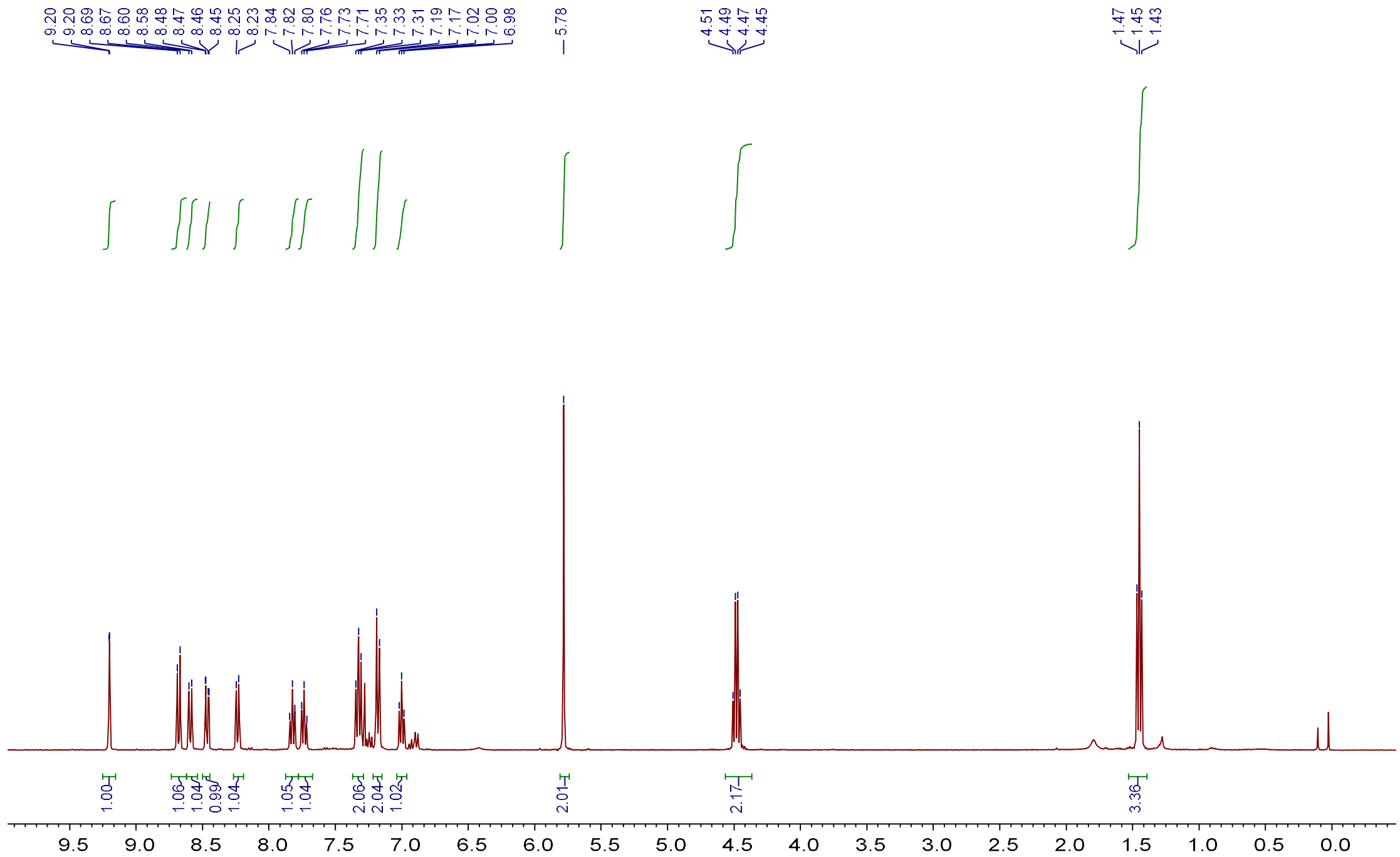
9.20 9.20 8.69 8.67 8.60 8.58 8.48 8.47 8.46 8.45 8.25 8.23 7.84 7.82 7.80 7.76 7.73 7.71 7.35 7.33 7.31 7.19 7.17 7.02 7.00 6.98
5.78
4.51 4.49 4.47 4.45
1.47 1.45 1.43
1.00 1.06 1.04 0.99 1.04 1.05 1.04 2.06 2.04 1.02 2.01 2.17 3.36
9.5 9.0 8.5 8.0 7.5 7.0 6.5 6.0 5.5 5.0 4.5 4.0 3.5 3.0 2.5 2.0 1.5 1.0 0.5 0.0

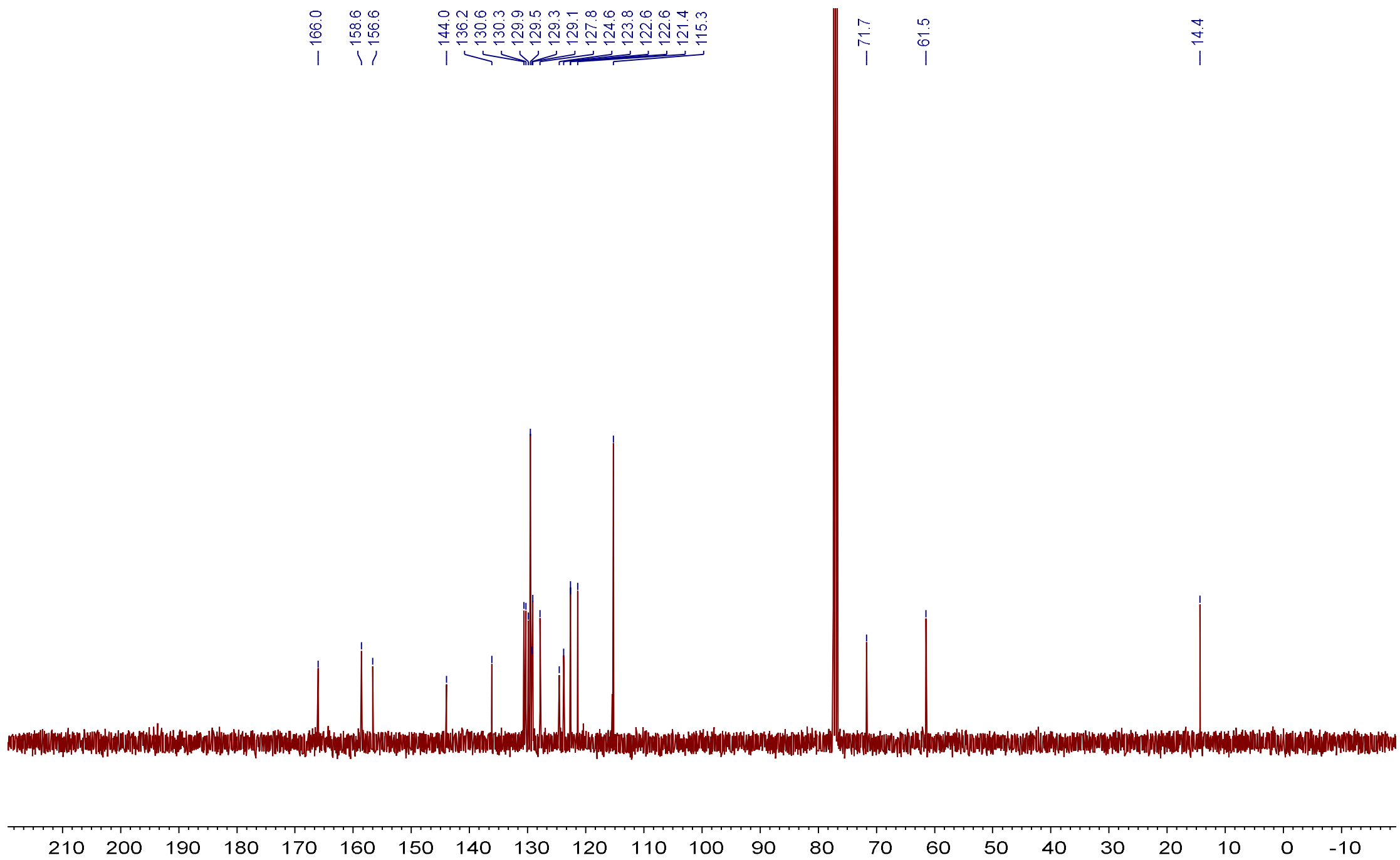
166.0
158.6 156.6
144.0 136.2 130.6 130.3 129.9 129.5 129.3 129.1 127.8 124.6 123.8 122.6 122.6 121.4 115.3
71.7
61.5
14.4
210 200 190 180 170 160 150 140 130 120 110 100 90 80 70 60 50 40 30 20 10 0 -10

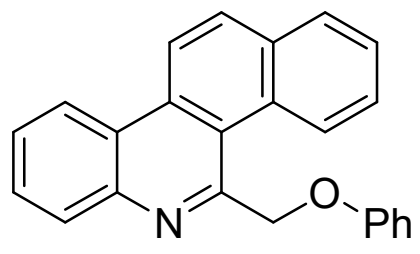
N
O
Ph

4g

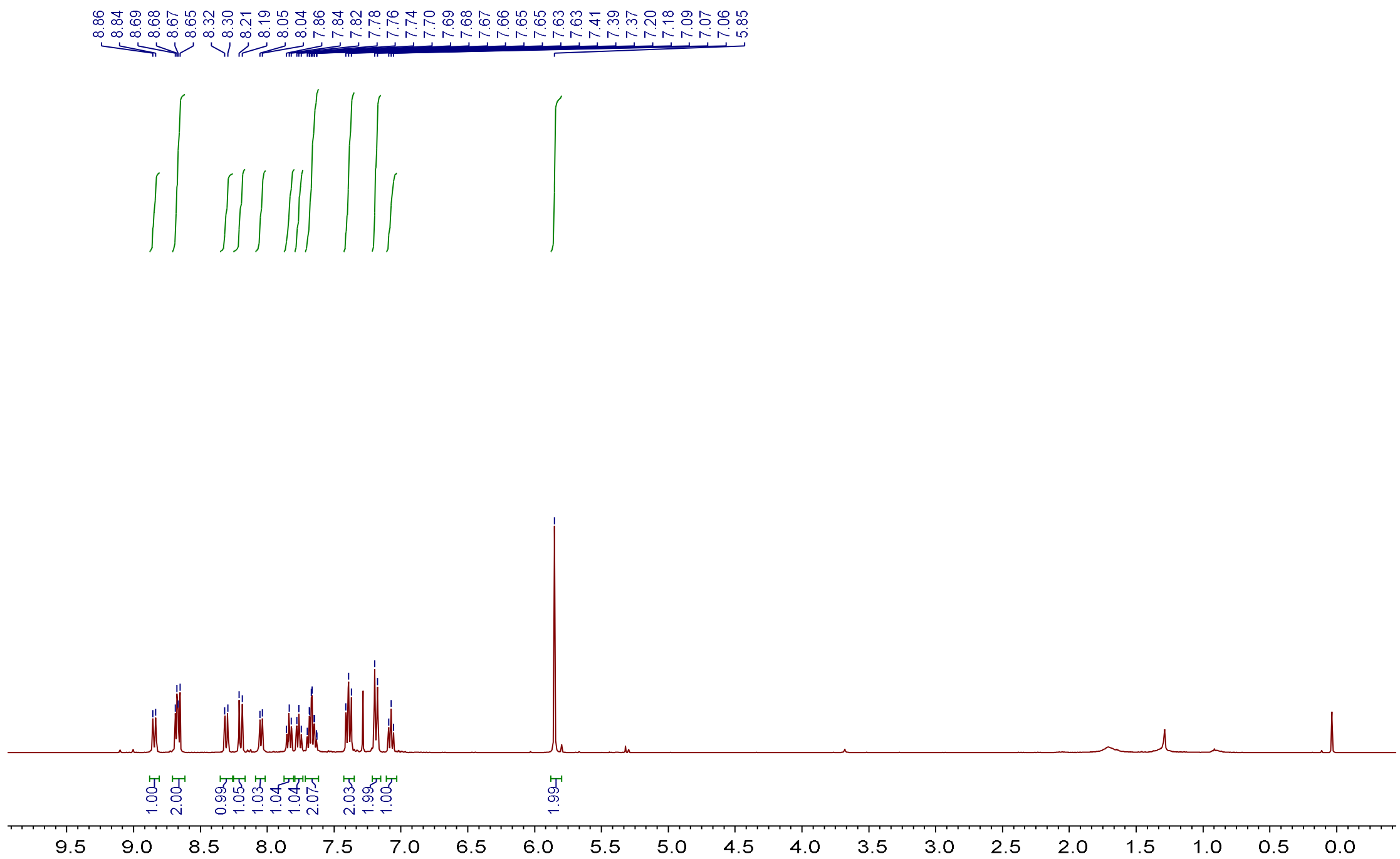
8.86
8.84
8.69
8.68
8.67
8.65
8.32
8.30
8.21
8.19
8.05
8.04
7.86
7.84
7.82
7.78
7.76
7.74
7.70
7.69
7.68
7.67
7.66
7.65
7.65
7.63
7.63
7.41
7.39
7.37
7.20
7.18
7.09
7.07
7.06
5.85
1.00
2.00
0.99
1.05
1.03
1.04
1.04
2.07
2.03
1.99
1.00
1.99
9.5
9.0
8.5
8.0
7.5
7.0
6.5
6.0
5.5
5.0
4.5
4.0
3.5
3.0
2.5
2.0
1.5
1.0
0.5
0.0

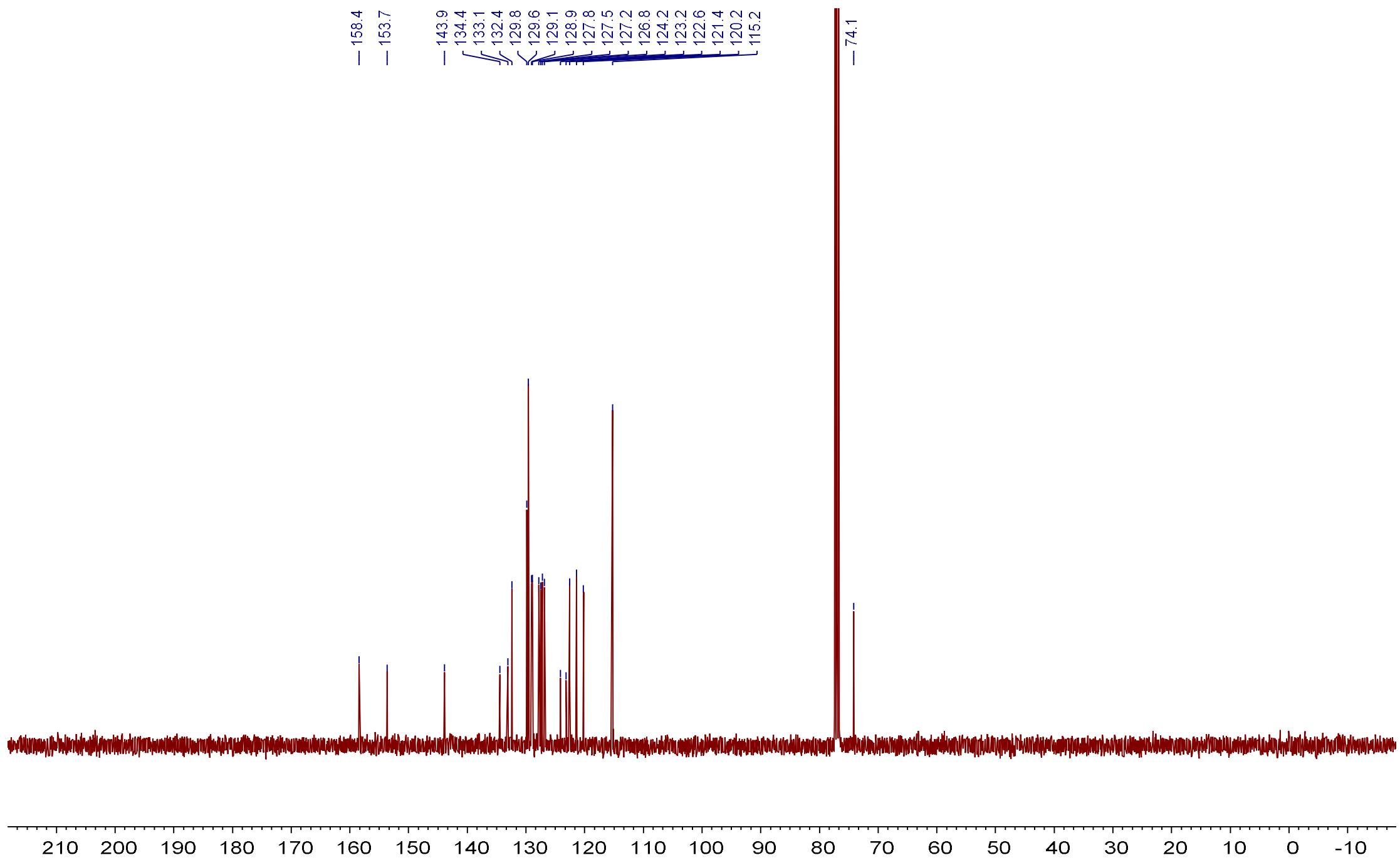
158.4
153.7
143.9
134.4
133.1
132.4
129.8
129.6
129.1
128.9
127.8
127.5
127.2
126.8
124.2
123.2
122.6
121.4
120.2
115.2
74.1
210
200
190
180
170
160
150
140
130
120
110
100
90
80
70
60
50
40
30
20
10
0
-10

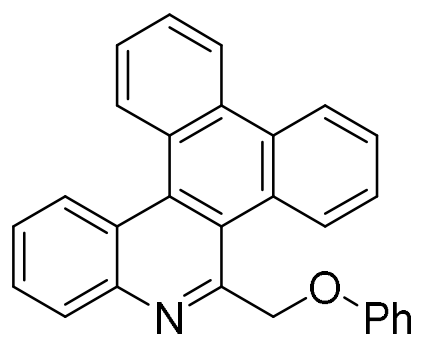
O
Ph
N

4h

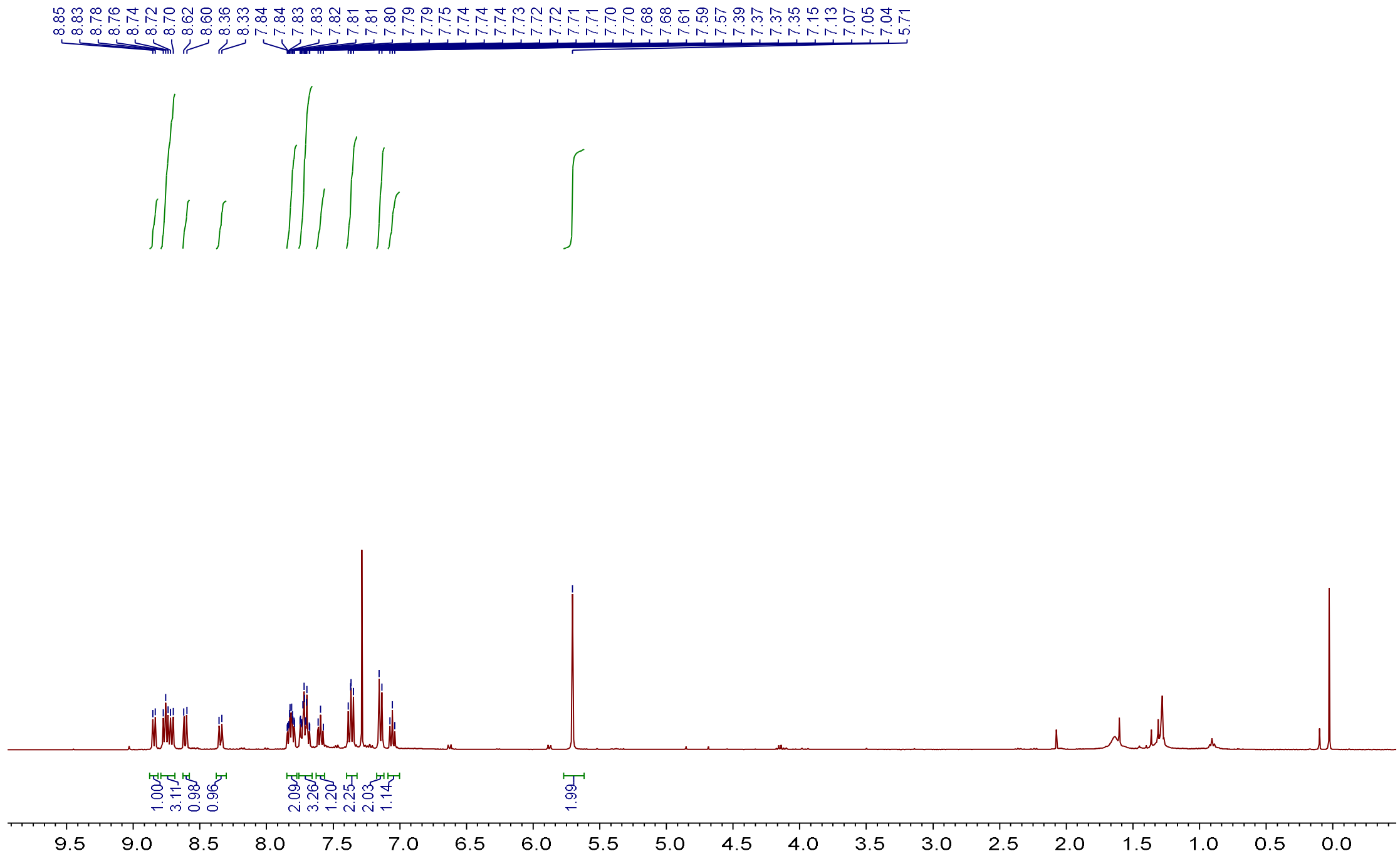
8.85 8.83 8.78 8.76 8.74 8.72 8.70 8.62 8.60 8.36 8.33 7.84 7.83 7.83 7.82 7.81 7.81 7.80 7.79 7.79 7.75 7.74 7.74 7.74 7.73 7.73 7.72 7.72 7.71 7.71 7.70 7.70 7.68 7.68 7.61 7.59 7.57 7.39 7.37 7.37 7.35 7.15 7.13 7.07 7.05 7.04 5.71
1.00 3.11 0.98 0.96 2.09 3.26 1.20 2.25 2.03 1.14 1.99
9.5 9.0 8.5 8.0 7.5 7.0 6.5 6.0 5.5 5.0 4.5 4.0 3.5 3.0 2.5 2.0 1.5 1.0 0.5 0.0

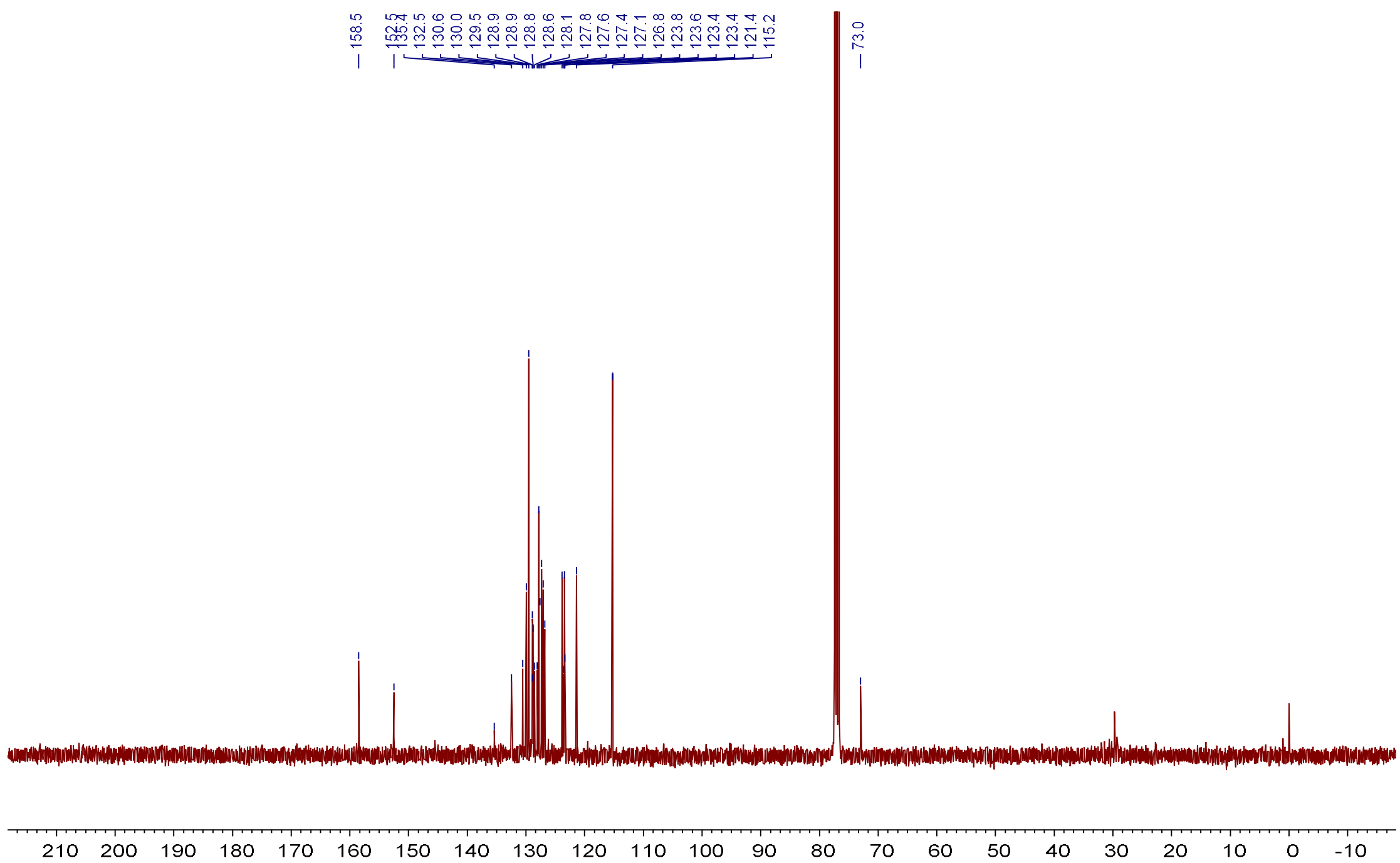
158.5 152.5 135.4 132.5 130.6 130.0 129.5 128.9 128.9 128.8 128.6 128.1 127.8 127.6 127.4 127.1 126.8 123.8 123.6 123.4 123.4 121.4 115.2 73.0
210 200 190 180 170 160 150 140 130 120 110 100 90 80 70 60 50 40 30 20 10 0 -10

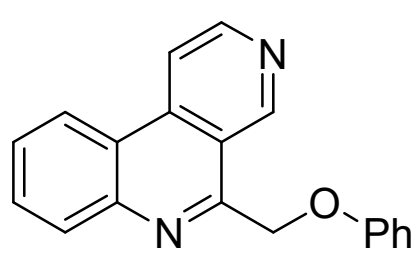

4i

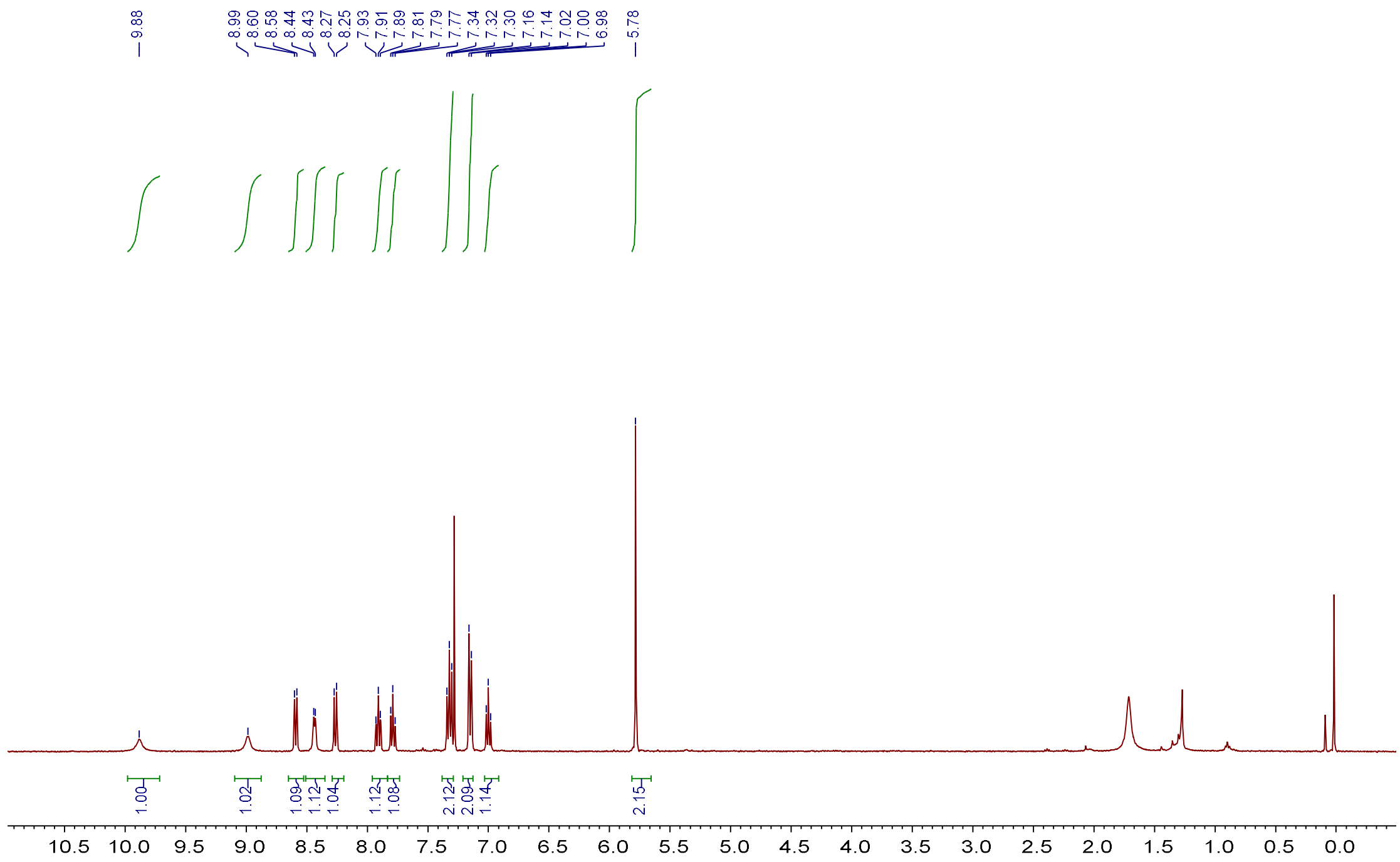

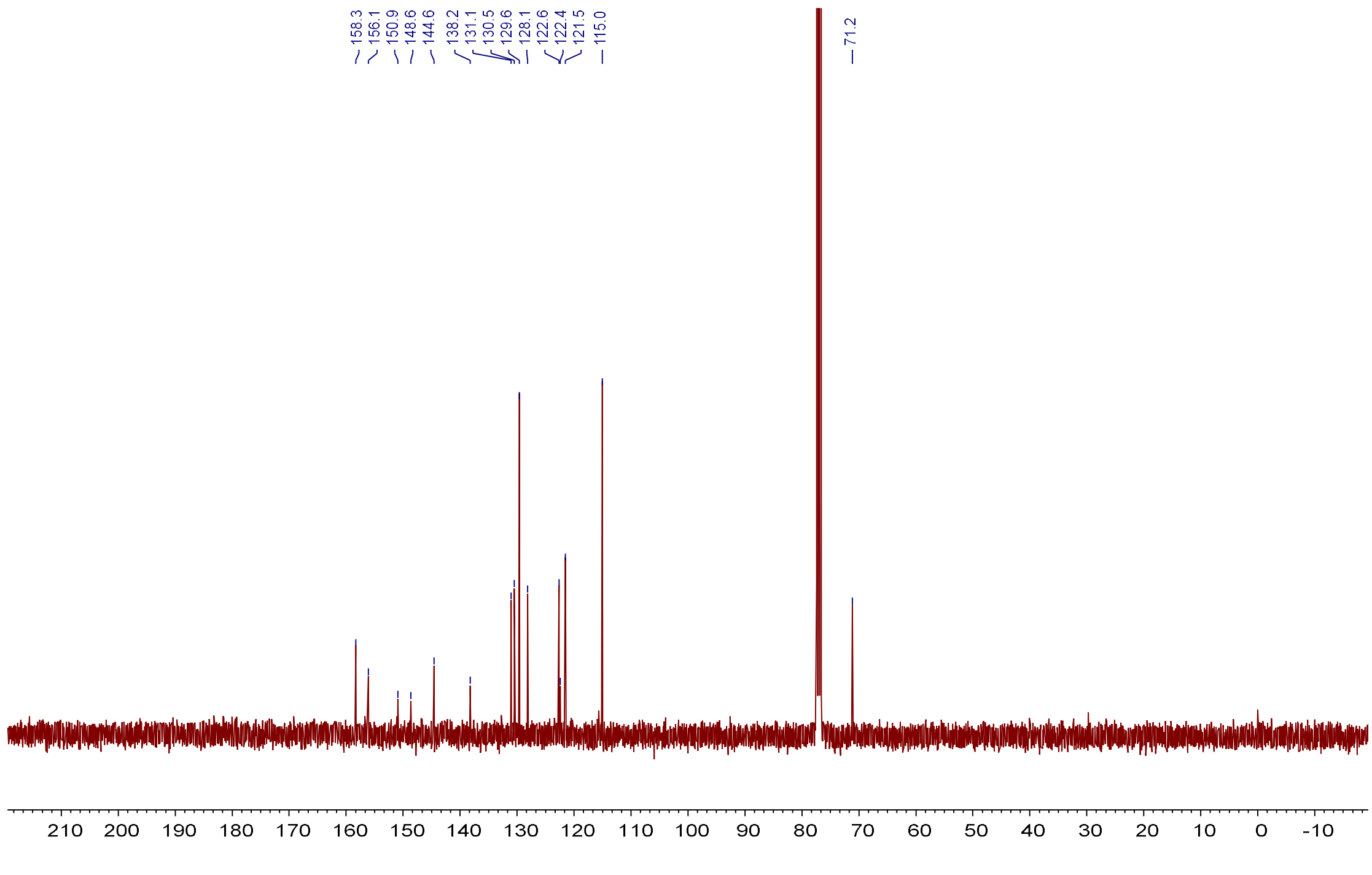

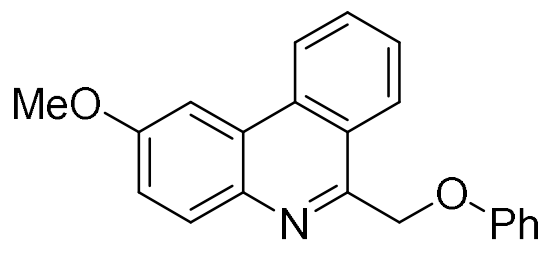

4j

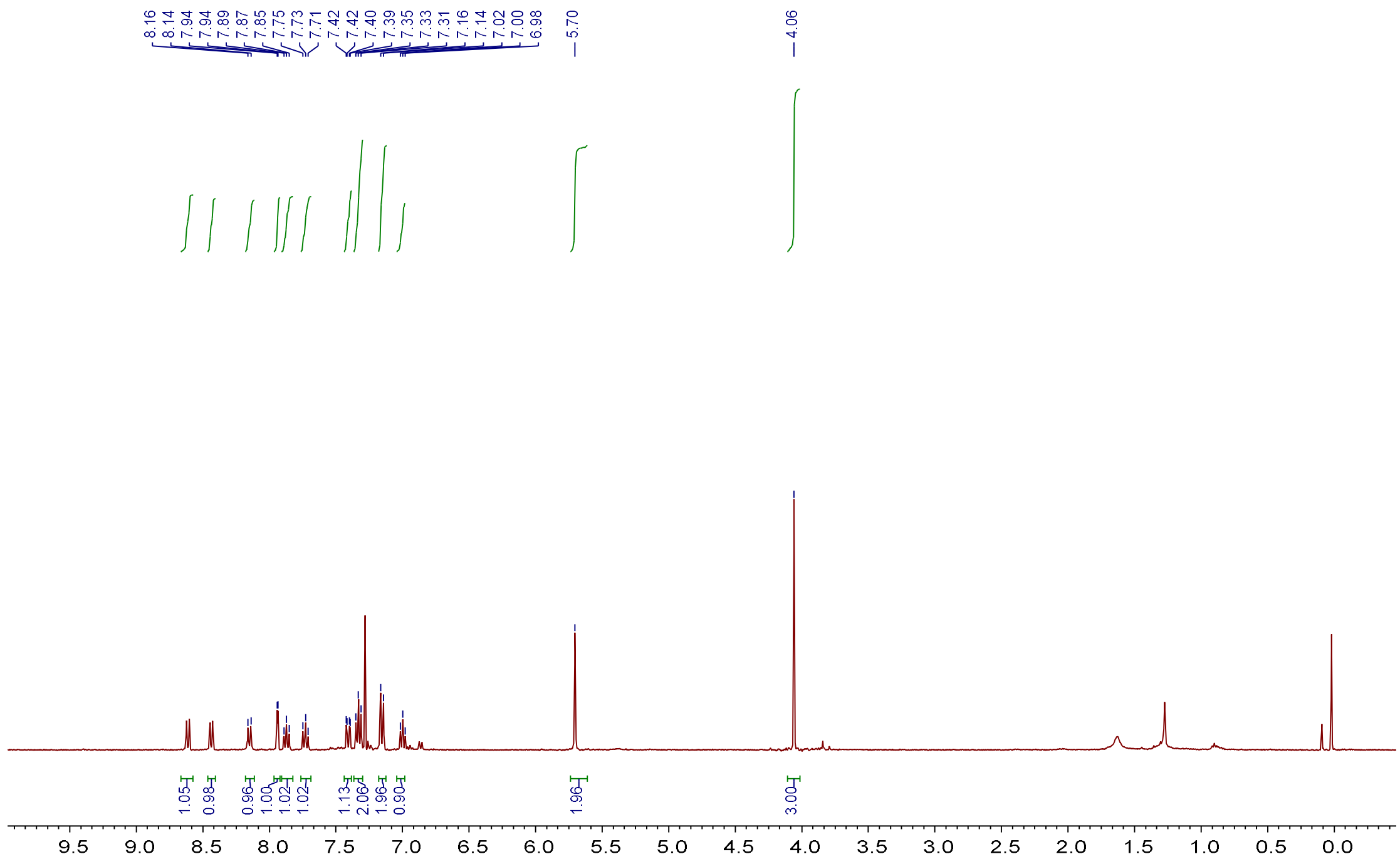

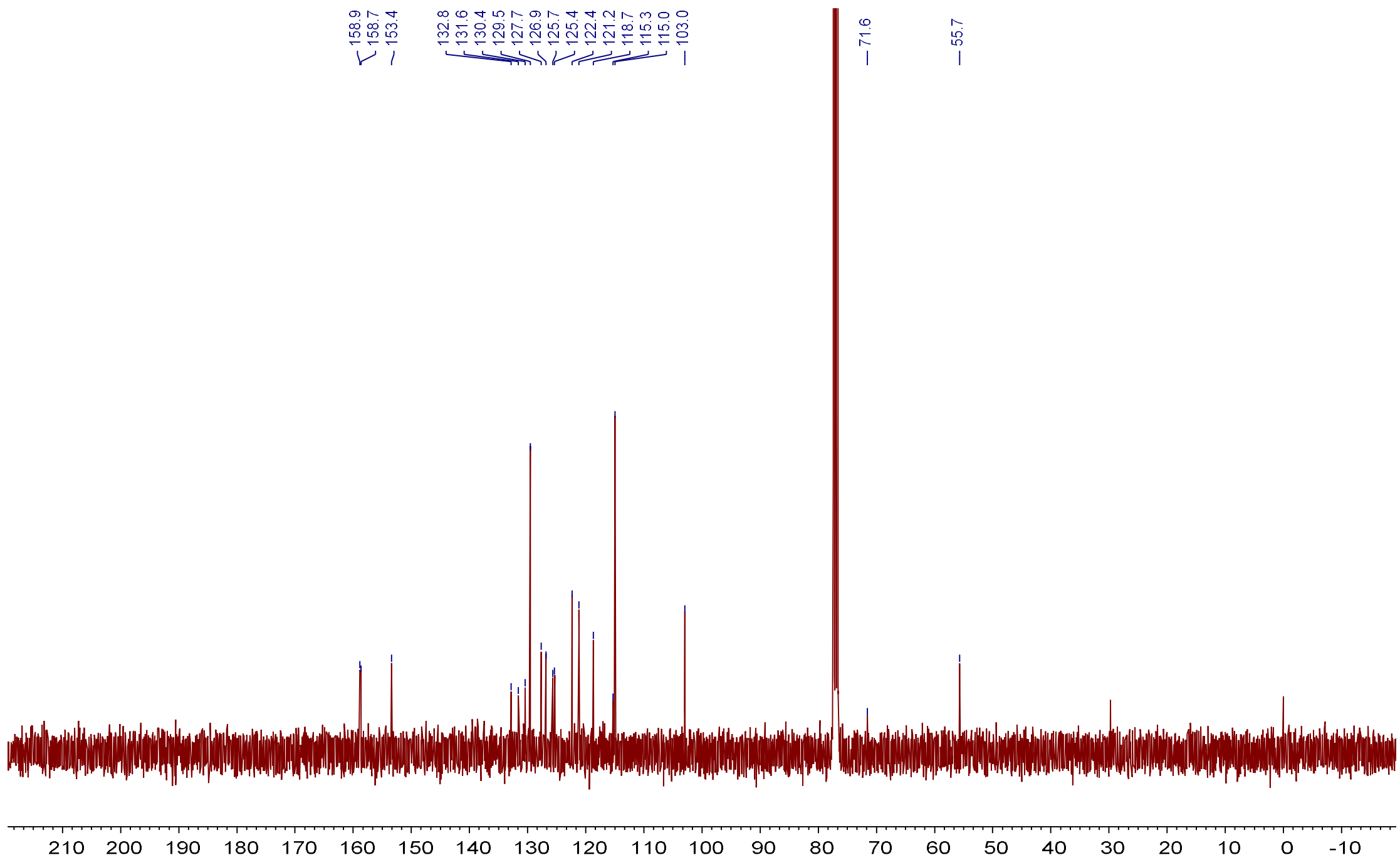

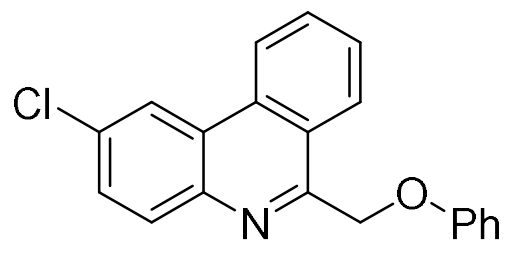
Cl
N
O
Ph

4k

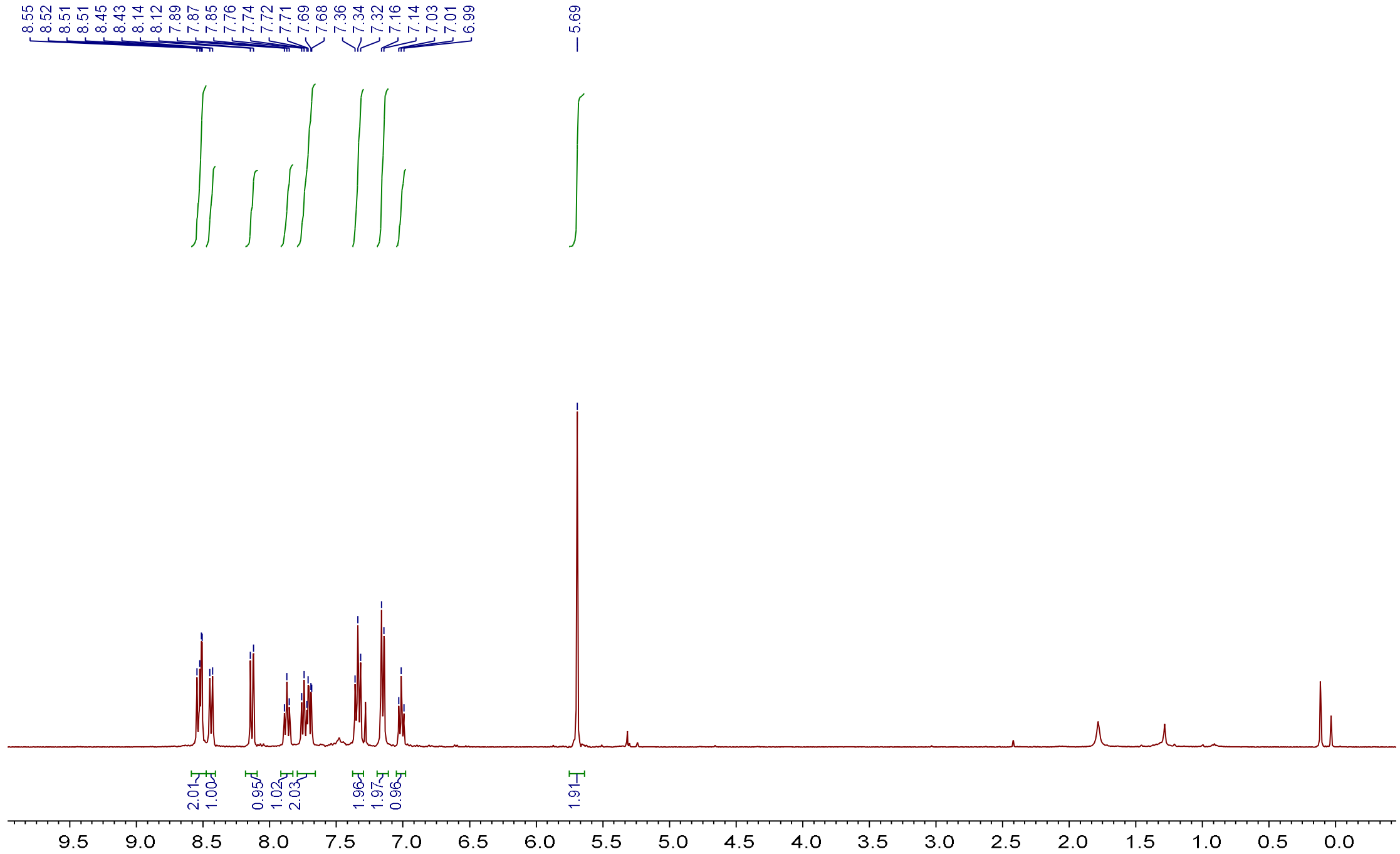
8.55
8.52
8.51
8.51
8.45
8.43
8.14
8.12
7.89
7.87
7.85
7.76
7.74
7.72
7.71
7.69
7.68
7.36
7.34
7.32
7.16
7.14
7.03
7.01
6.99
5.69
2.01
1.00
0.95
1.02
2.03
1.96
1.97
0.96
1.91
9.5
9.0
8.5
8.0
7.5
7.0
6.5
6.0
5.5
5.0
4.5
4.0
3.5
3.0
2.5
2.0
1.5
1.0
0.5
0.0

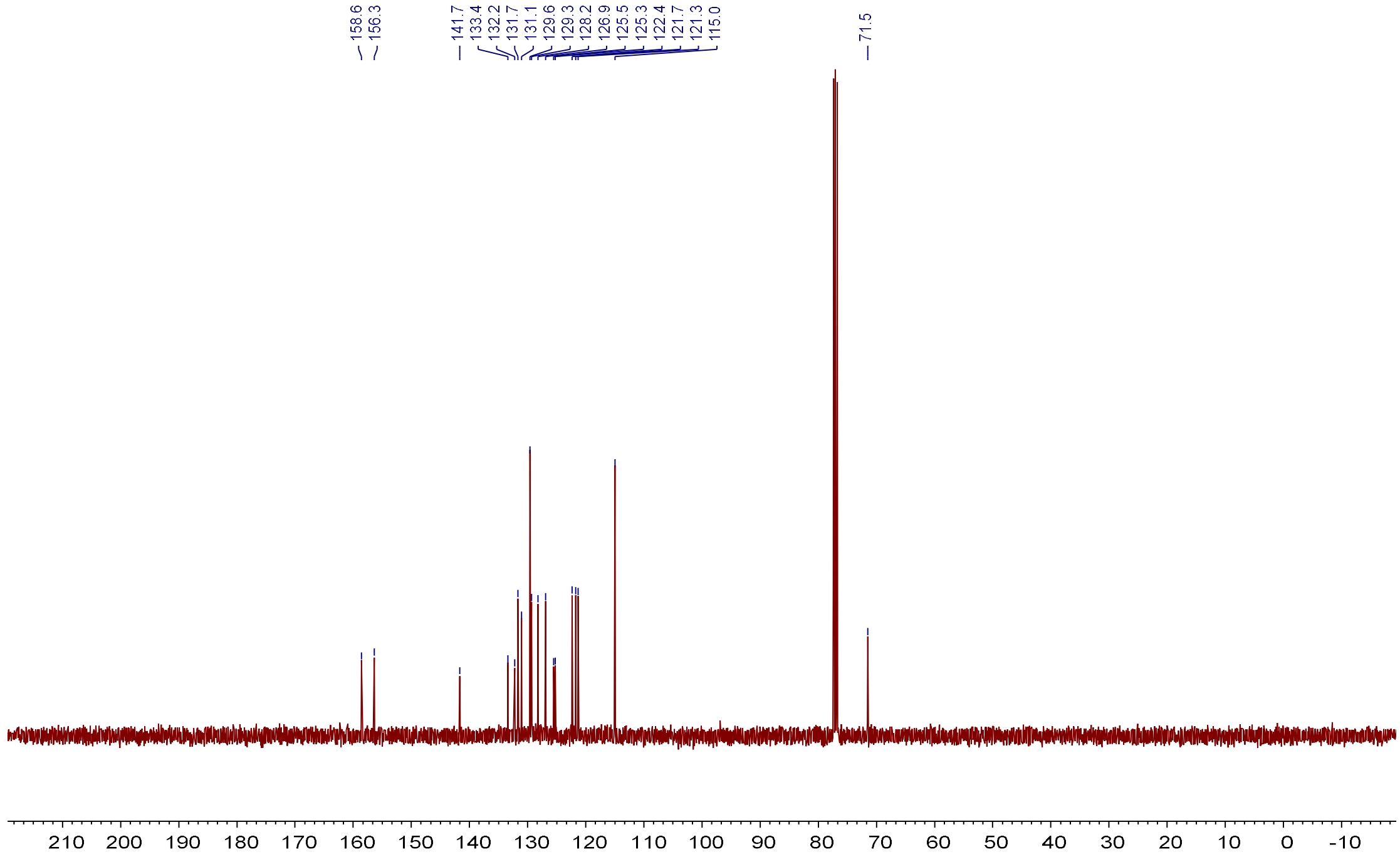
158.6
156.3
141.7
133.4
132.2
131.7
131.1
129.6
129.3
128.2
126.9
125.5
125.3
122.4
121.7
121.3
115.0
71.5
210
200
190
180
170
160
150
140
130
120
110
100
90
80
70
60
50
40
30
20
10
0
-10

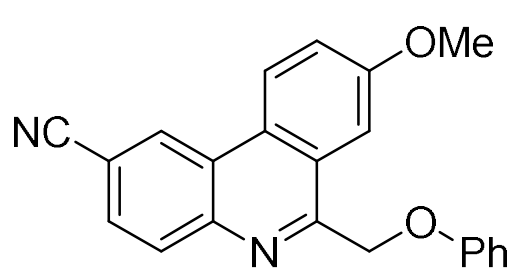

OMe
NC
N
O
Ph

4l

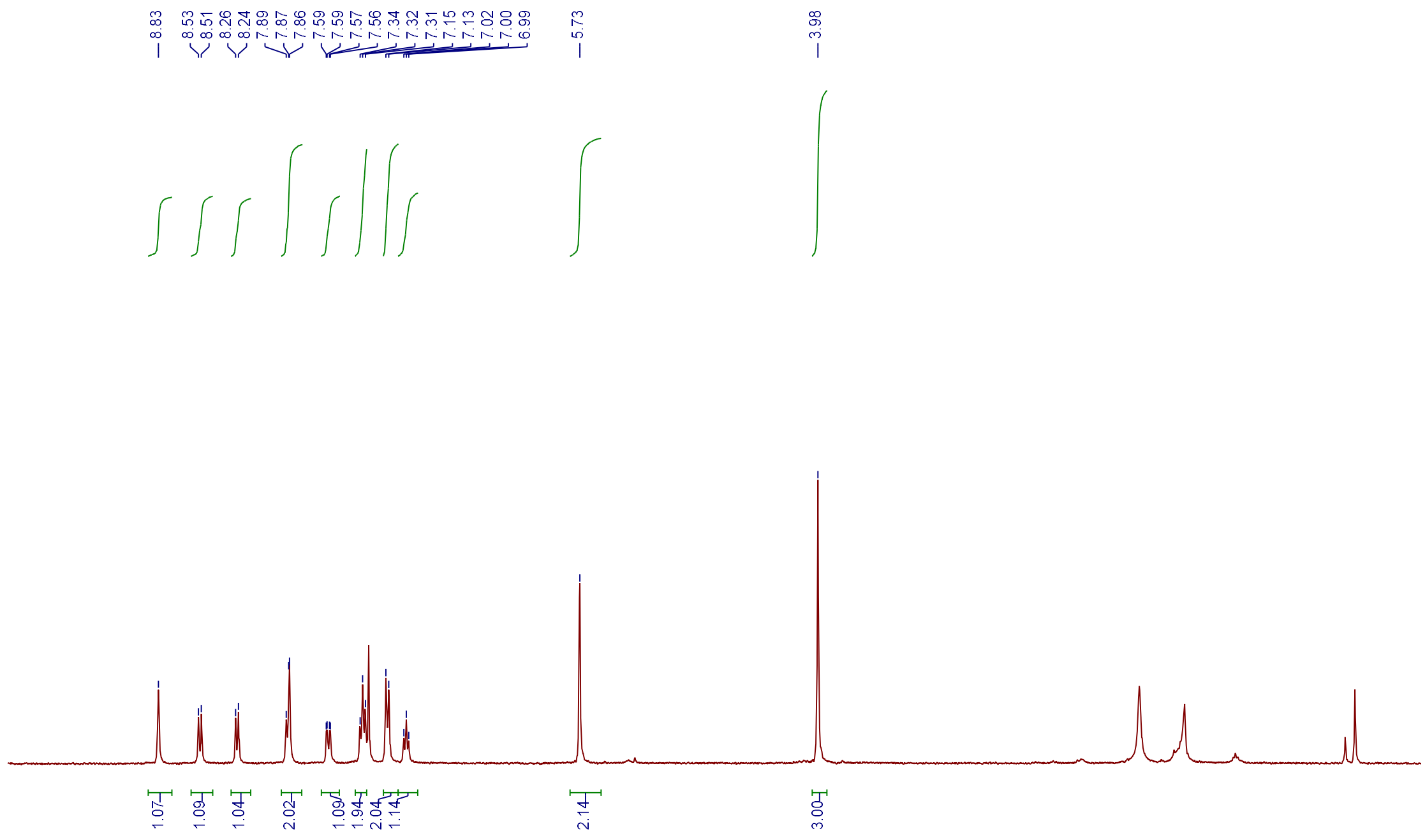

8.83
8.53
8.51
8.26
8.24
7.89
7.87
7.86
7.59
7.59
7.57
7.56
7.34
7.32
7.31
7.15
7.13
7.02
7.00
6.99
5.73
3.98
1.07
1.09
1.04
2.02
1.09
1.94
2.04
1.14
2.14
3.00
9.5 9.0 8.5 8.0 7.5 7.0 6.5 6.0 5.5 5.0 4.5 4.0 3.5 3.0 2.5 2.0 1.5 1.0 0.5 0.0

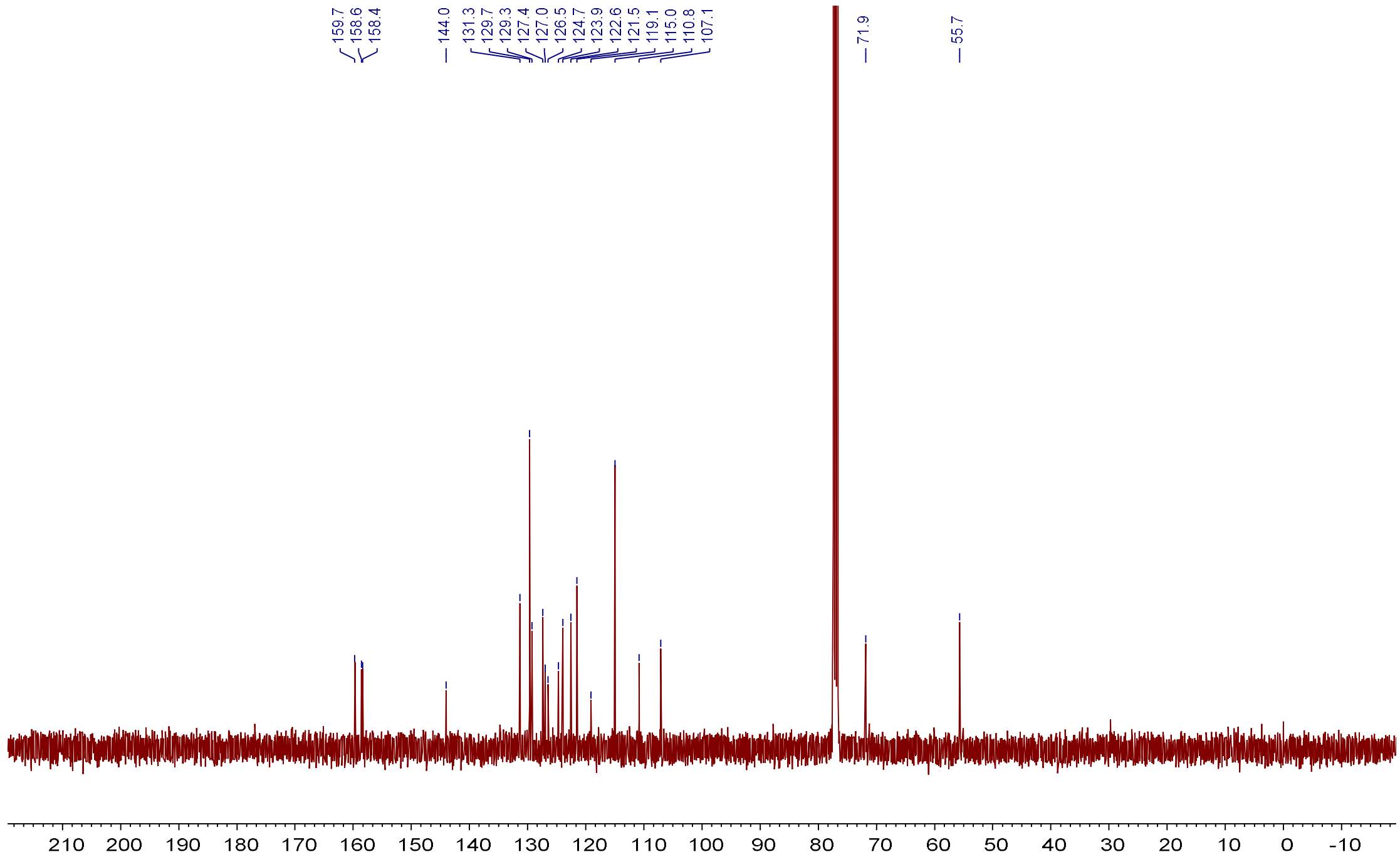

159.7
158.6
158.4
144.0
131.3
129.7
129.3
127.4
127.0
126.5
124.7
123.9
122.6
121.5
119.1
115.0
110.8
107.1
71.9
55.7
210 200 190 180 170 160 150 140 130 120 110 100 90 80 70 60 50 40 30 20 10 0 -10

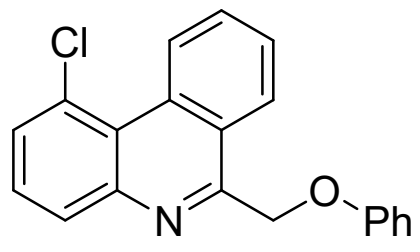

Cl
N
O
Ph

4m

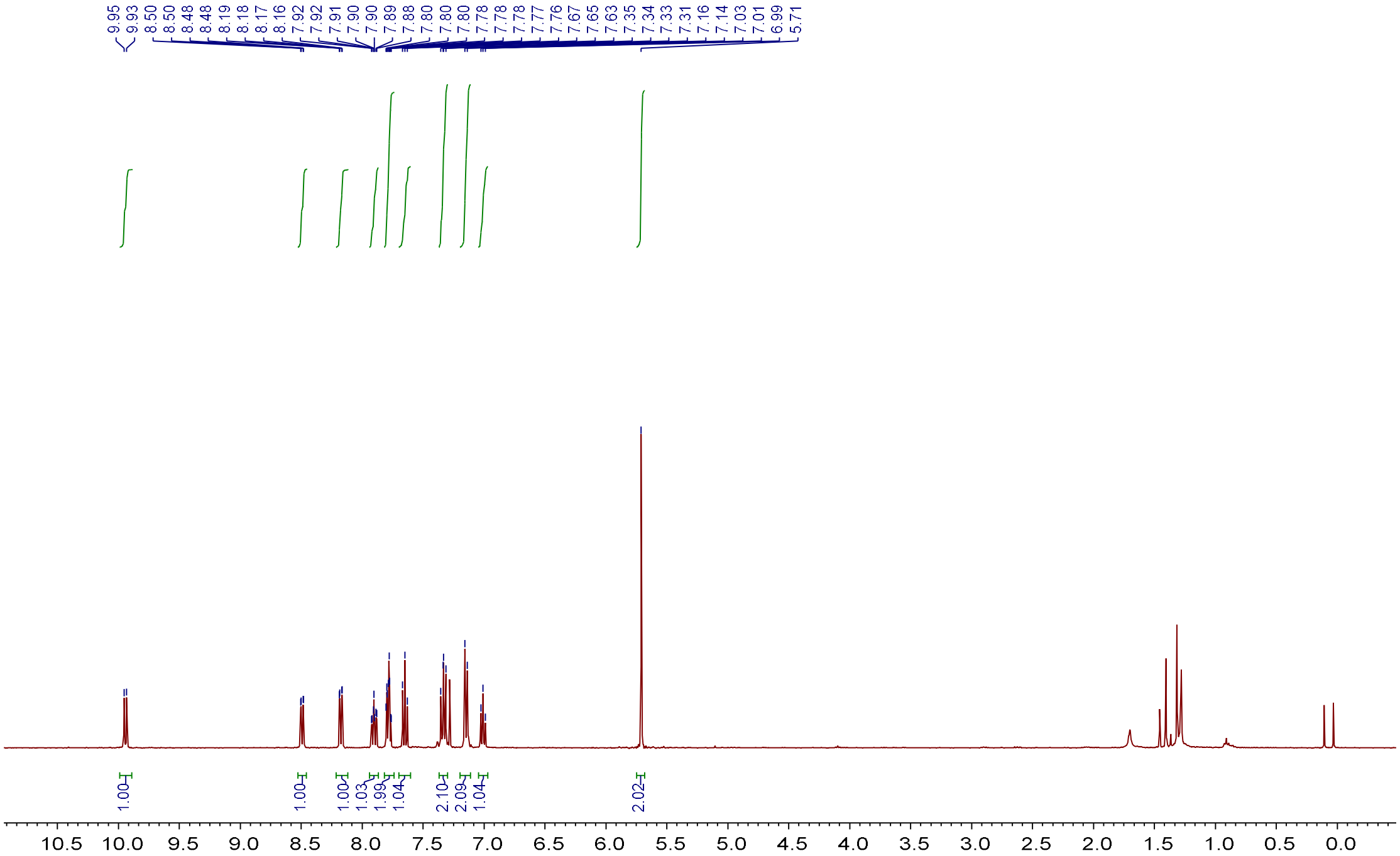

9.95
9.93
8.50
8.50
8.48
8.48
8.19
8.18
8.17
8.16
7.92
7.92
7.91
7.90
7.90
7.89
7.88
7.80
7.80
7.80
7.78
7.78
7.78
7.77
7.76
7.67
7.65
7.63
7.35
7.34
7.33
7.31
7.16
7.14
7.03
7.01
6.99
5.71
1.00
1.00
1.00
1.03
1.99
1.04
2.10
2.09
1.04
2.02
10.5 10.0 9.5 9.0 8.5 8.0 7.5 7.0 6.5 6.0 5.5 5.0 4.5 4.0 3.5 3.0 2.5 2.0 1.5 1.0 0.5 0.0

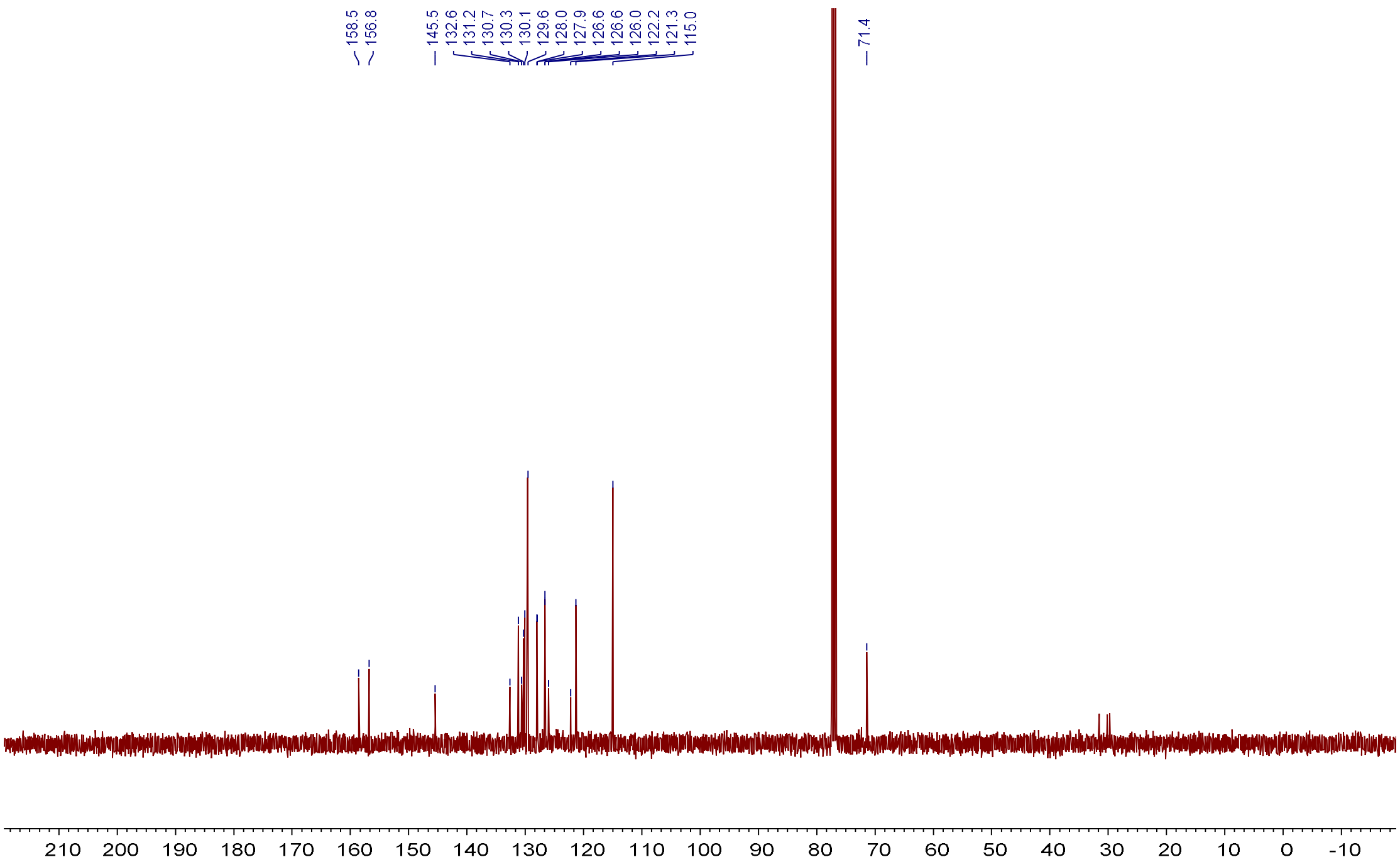

158.5
156.8
145.5
132.6
131.2
130.7
130.3
130.1
129.6
128.0
127.9
126.6
126.6
126.0
122.2
121.3
115.0
71.4
210 200 190 180 170 160 150 140 130 120 110 100 90 80 70 60 50 40 30 20 10 0 -10

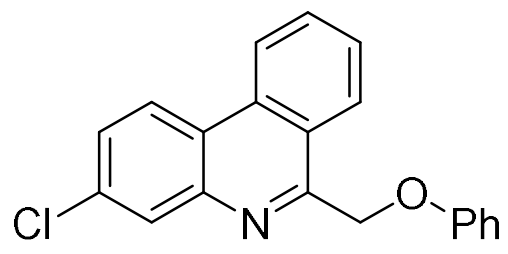
Cl
N
O
Ph

4n

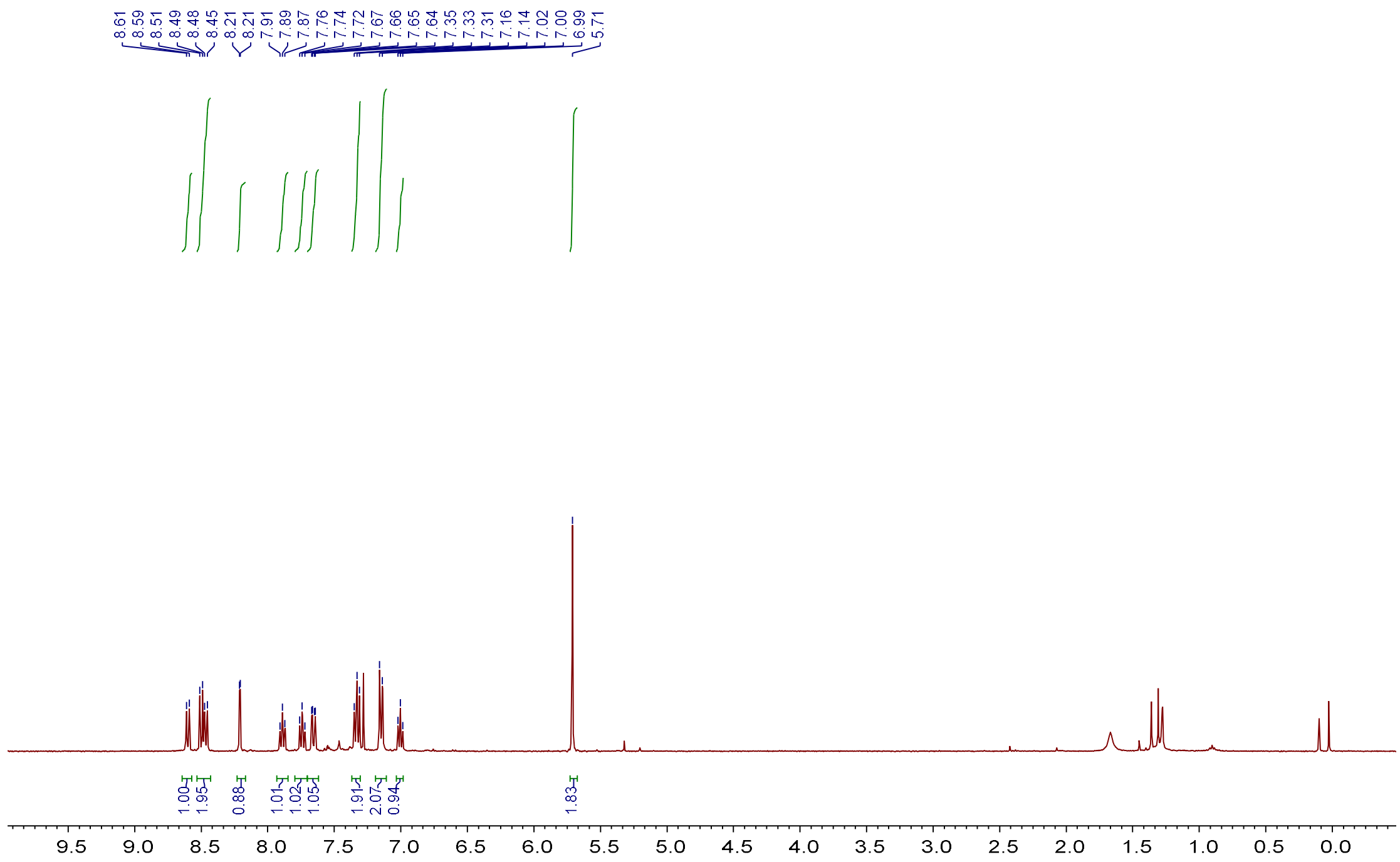
8.61 8.59 8.51 8.49 8.48 8.45 8.21 8.21 7.91 7.89 7.87 7.76 7.74 7.72 7.67 7.66 7.65 7.64 7.35 7.33 7.31 7.16 7.14 7.02 7.00 6.99 5.71
1.00 1.95 0.88 1.01 1.02 1.05 1.91 2.07 0.94 1.83
9.5 9.0 8.5 8.0 7.5 7.0 6.5 6.0 5.5 5.0 4.5 4.0 3.5 3.0 2.5 2.0 1.5 1.0 0.5 0.0

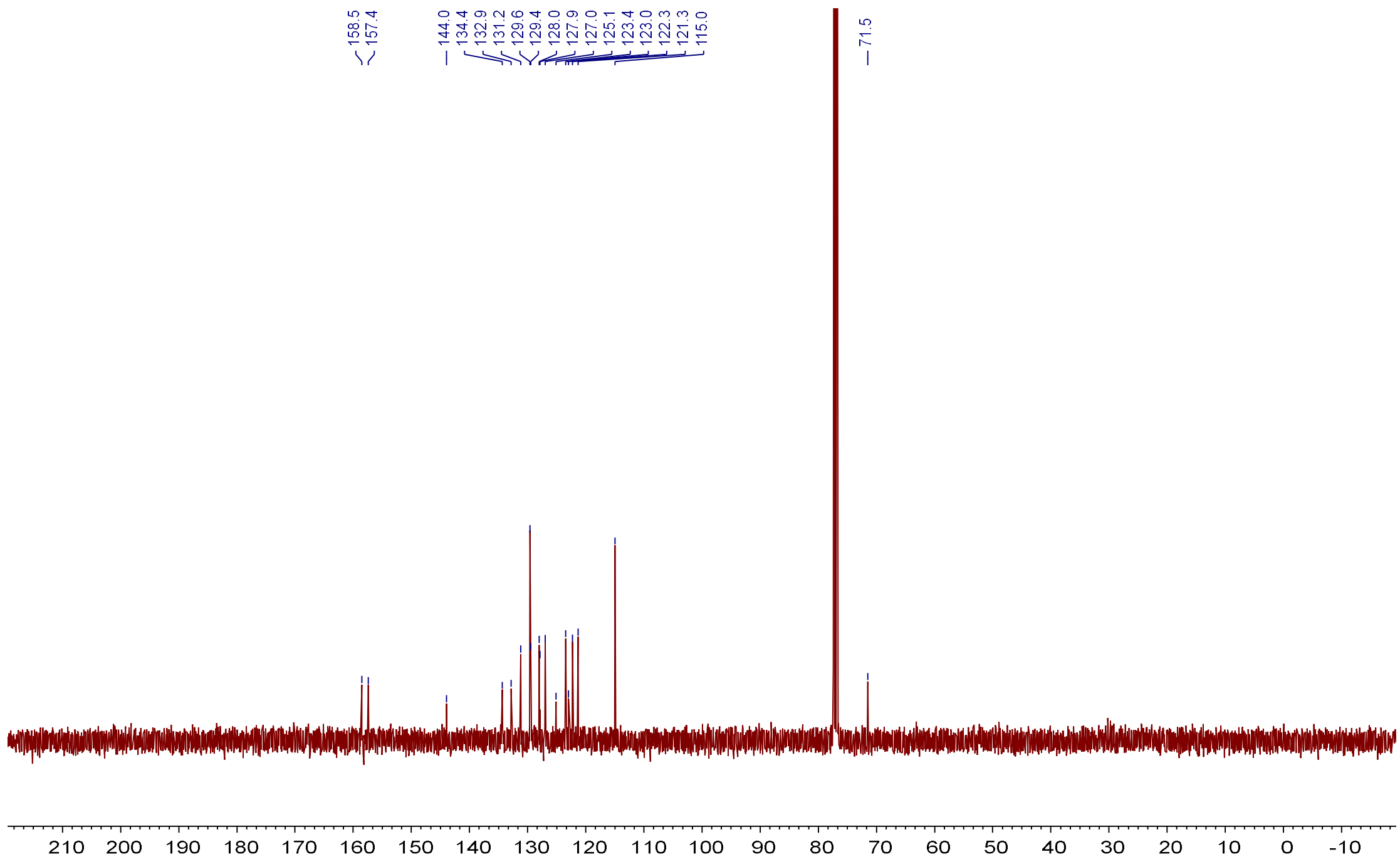
158.5 157.4
144.0 134.4 132.9 131.2 129.6 129.4 128.0 127.9 127.0 125.1 123.4 123.0 122.3 121.3 115.0
71.5
210 200 190 180 170 160 150 140 130 120 110 100 90 80 70 60 50 40 30 20 10 0 -10

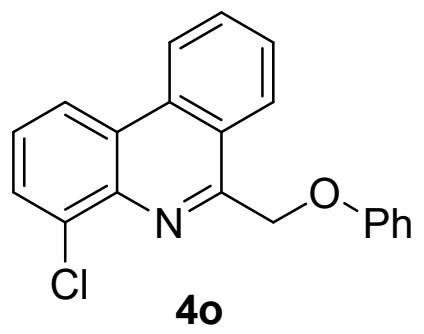
N
O
Ph
Cl

4o

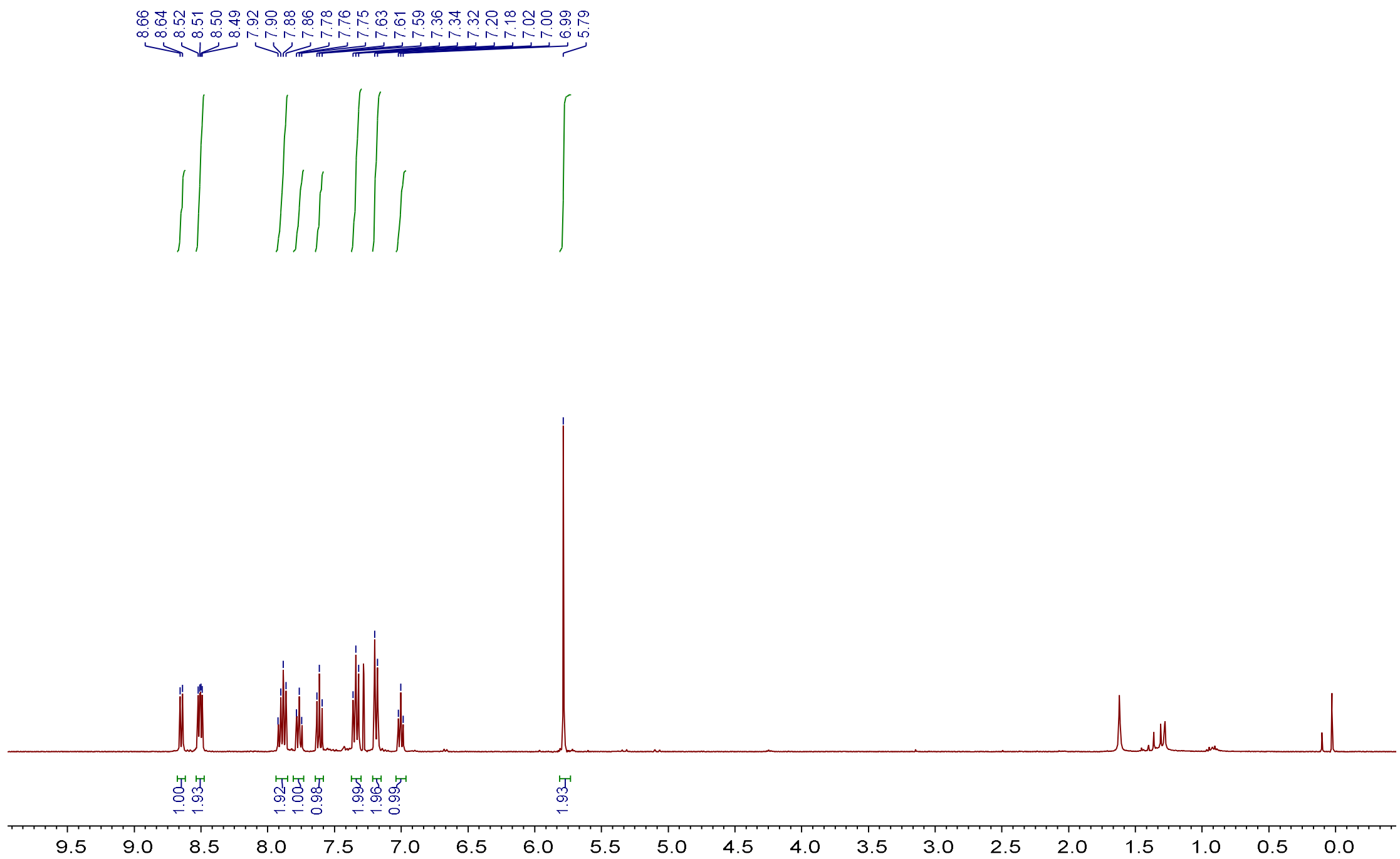
8.66
8.64
8.52
8.51
8.50
8.49
7.92
7.90
7.88
7.86
7.78
7.76
7.75
7.63
7.61
7.59
7.36
7.34
7.32
7.20
7.18
7.02
7.00
6.99
5.79
1.00
1.93
1.92
1.00
0.98
1.99
1.96
0.99
1.93
9.5
9.0
8.5
8.0
7.5
7.0
6.5
6.0
5.5
5.0
4.5
4.0
3.5
3.0
2.5
2.0
1.5
1.0
0.5
0.0

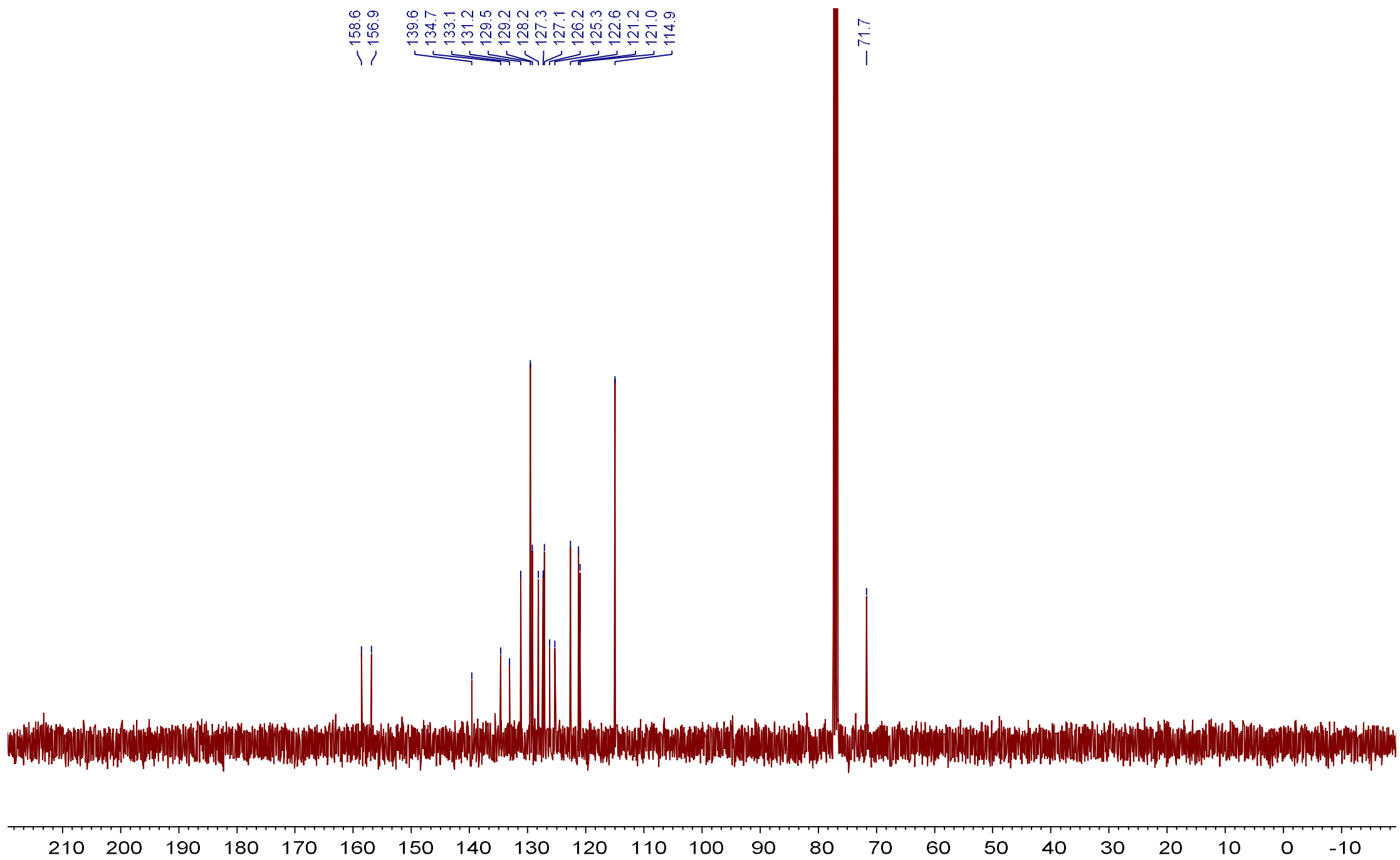
158.6
156.9
139.6
134.7
133.1
131.2
129.5
129.2
128.2
127.3
127.1
126.2
125.3
122.6
121.2
121.0
114.9
71.7
210
200
190
180
170
160
150
140
130
120
110
100
90
80
70
60
50
40
30
20
10
0
-10

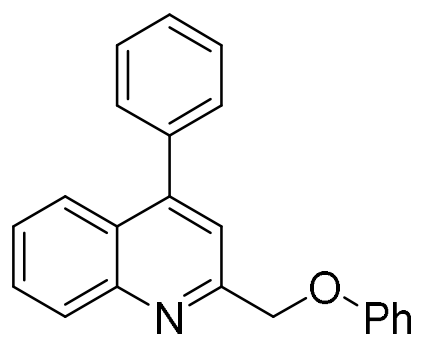
N
O
Ph

4p

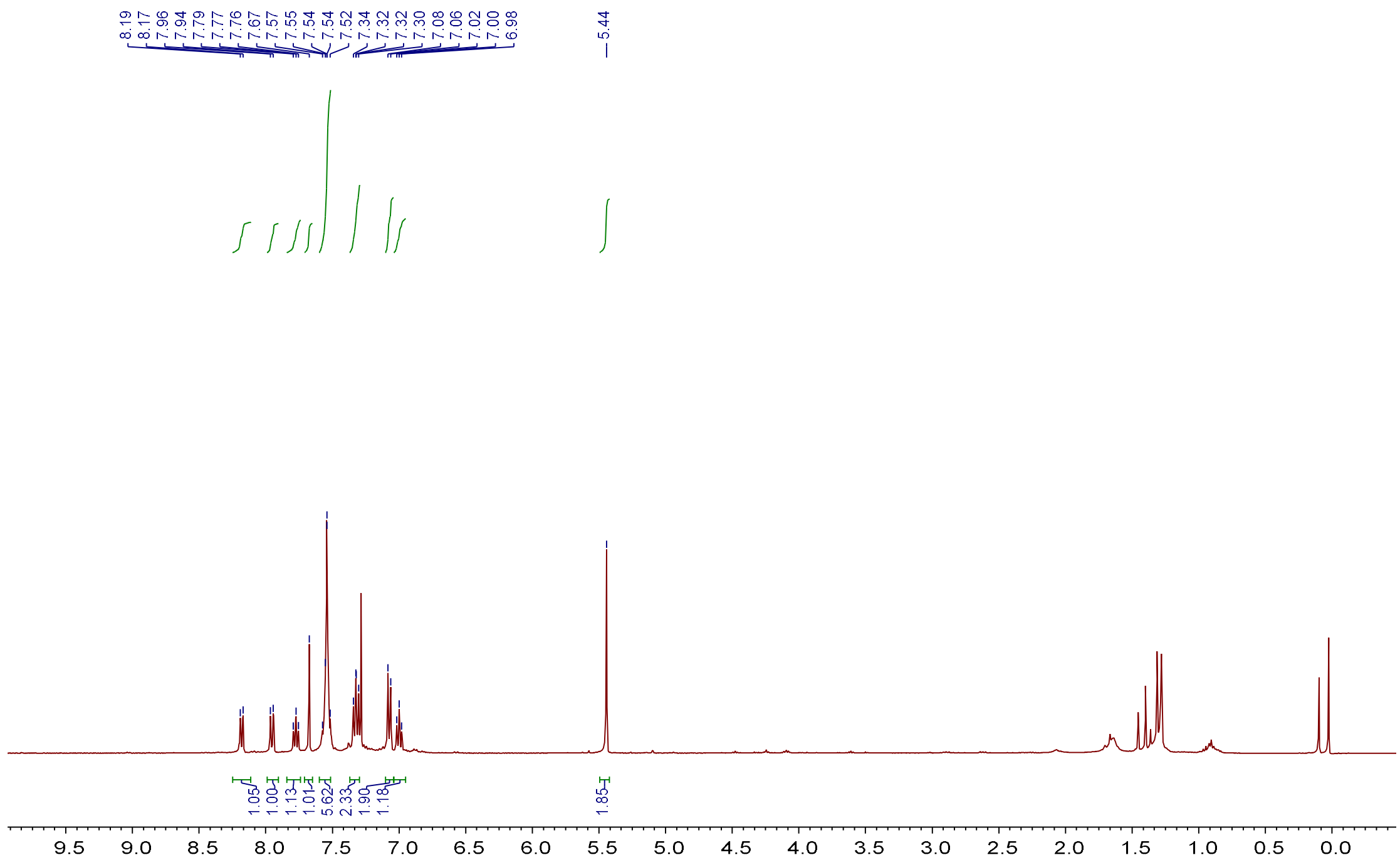
8.19
8.17
7.96
7.94
7.79
7.77
7.76
7.67
7.57
7.55
7.54
7.54
7.52
7.34
7.32
7.32
7.30
7.08
7.06
7.02
7.00
6.98
5.44
1.05
1.00
1.13
1.01
5.62
2.33
1.90
1.18
1.85
9.5 9.0 8.5 8.0 7.5 7.0 6.5 6.0 5.5 5.0 4.5 4.0 3.5 3.0 2.5 2.0 1.5 1.0 0.5 0.0

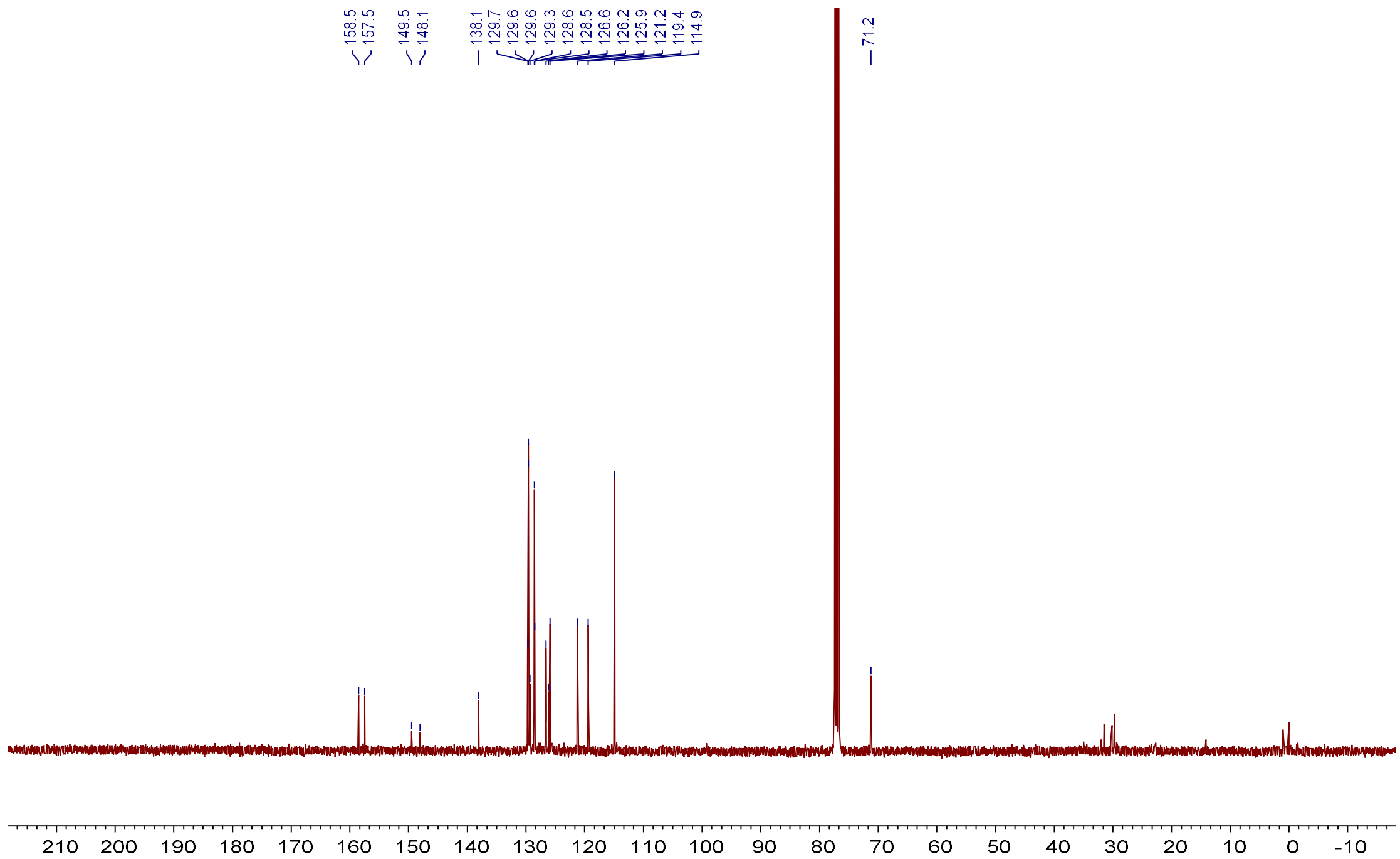
158.5
157.5
149.5
148.1
138.1
129.7
129.6
129.6
129.3
128.6
128.5
126.6
126.2
125.9
121.2
119.4
114.9
71.2
210 200 190 180 170 160 150 140 130 120 110 100 90 80 70 60 50 40 30 20 10 0 -10

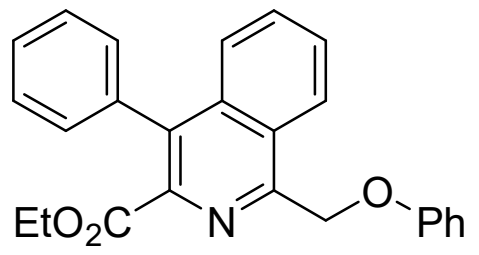
EtO2C
N
O
Ph

4q

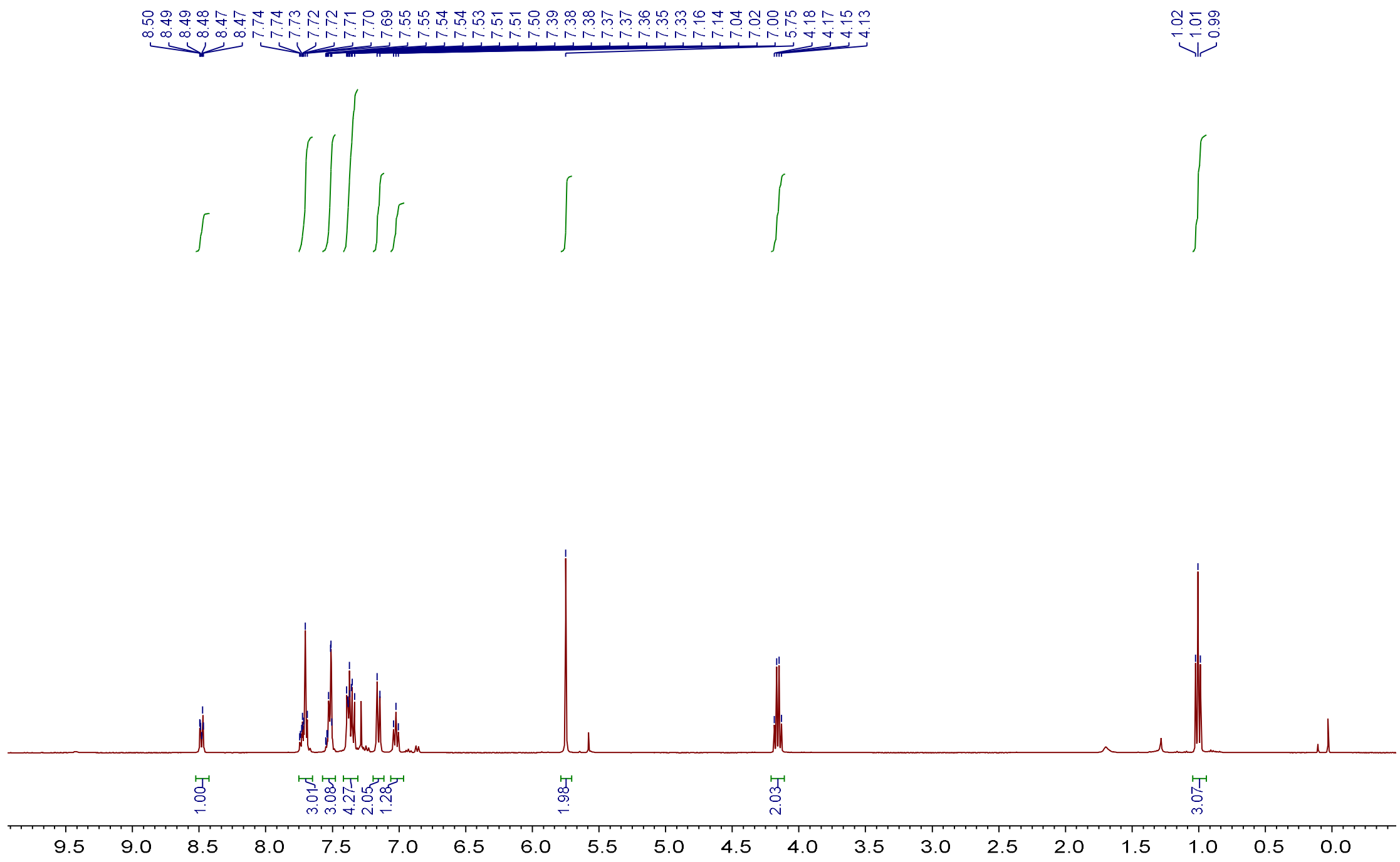
8.50
8.49
8.49
8.48
8.47
8.47
7.74
7.74
7.73
7.72
7.72
7.71
7.70
7.69
7.55
7.55
7.54
7.54
7.53
7.51
7.51
7.50
7.39
7.38
7.38
7.37
7.37
7.36
7.35
7.33
7.16
7.14
7.04
7.02
7.00
5.75
4.18
4.17
4.15
4.13
1.02
1.01
0.99
1.00
3.01
3.08
4.27
2.05
1.28
1.98
2.03
3.07
9.5 9.0 8.5 8.0 7.5 7.0 6.5 6.0 5.5 5.0 4.5 4.0 3.5 3.0 2.5 2.0 1.5 1.0 0.5 0.0

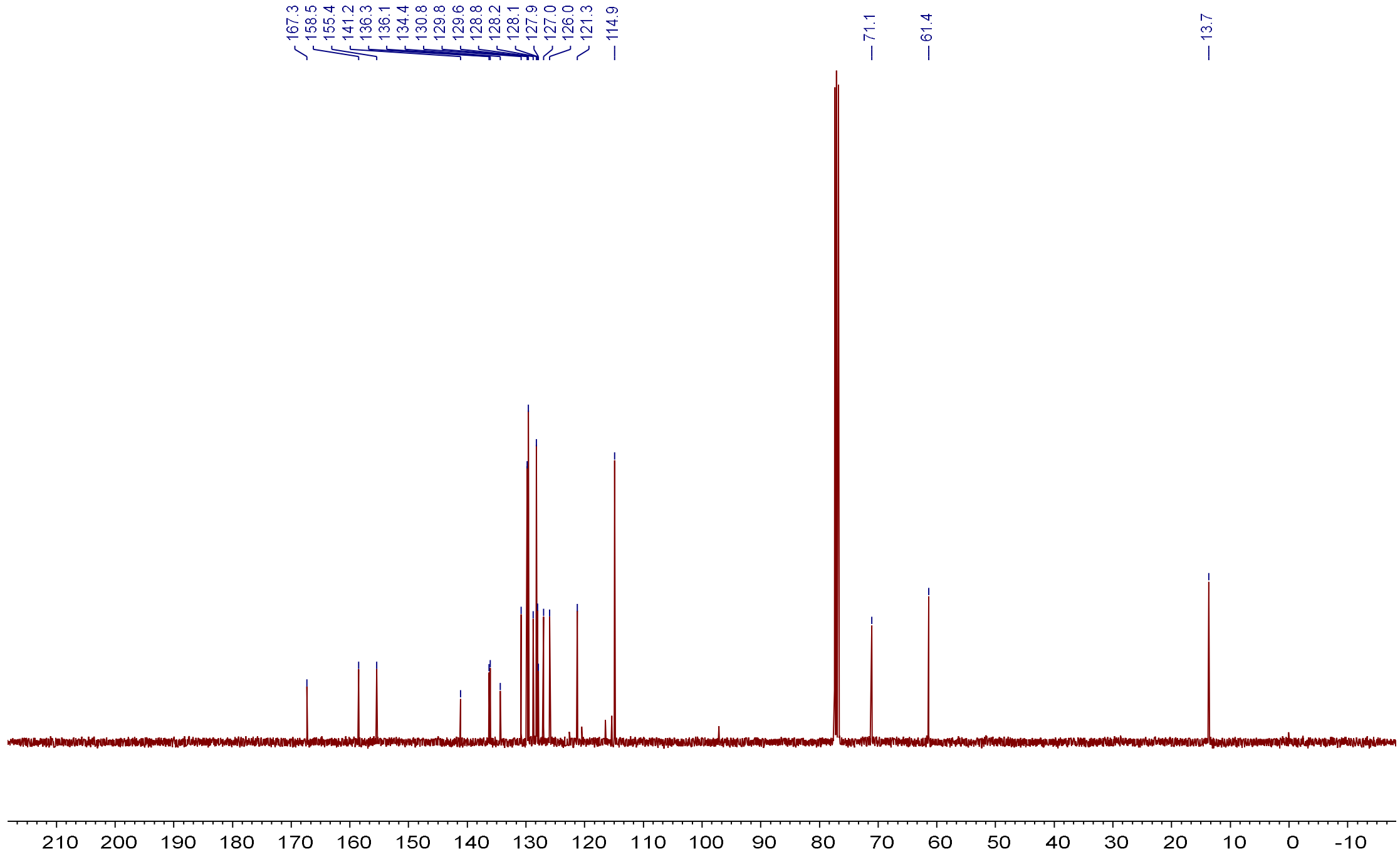
167.3
158.5
155.4
141.2
136.3
136.1
134.4
130.8
129.8
129.6
128.8
128.2
128.1
127.9
127.0
126.0
121.3
114.9
71.1
61.4
13.7
210 200 190 180 170 160 150 140 130 120 110 100 90 80 70 60 50 40 30 20 10 0 -10

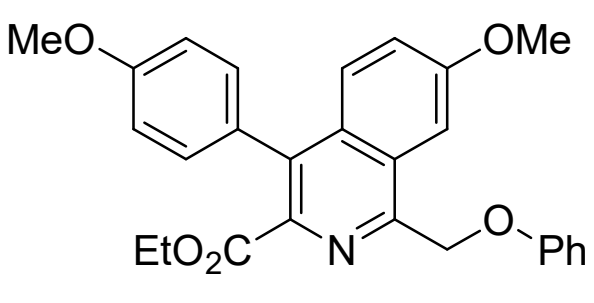
MeO
OMe
EtO2C
N
O
Ph

4r

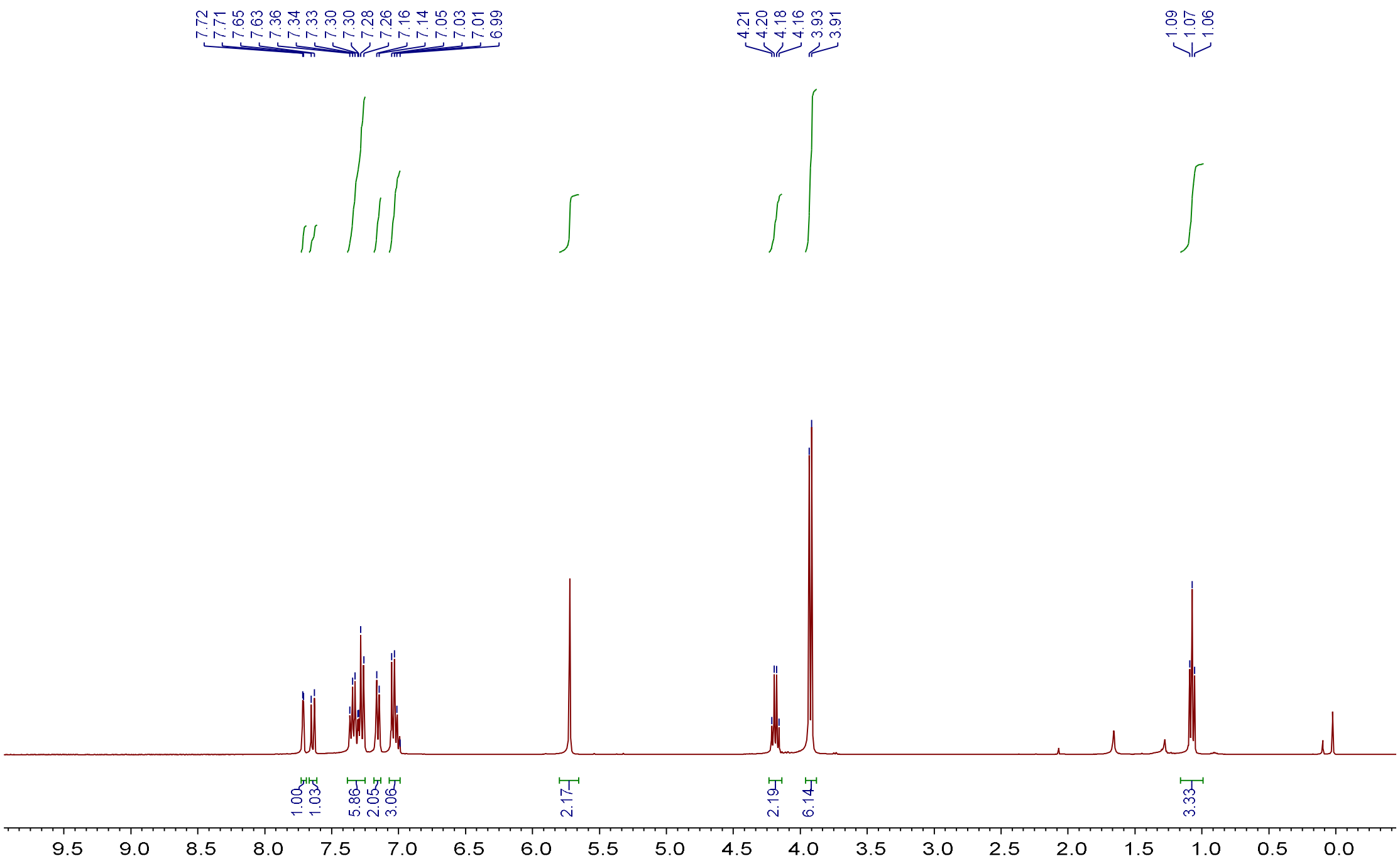
7.72
7.71
7.65
7.63
7.36
7.34
7.33
7.30
7.30
7.28
7.26
7.16
7.14
7.05
7.03
7.01
6.99
4.21
4.20
4.18
4.16
3.93
3.91
1.09
1.07
1.06
1.00
1.03
5.86
2.05
3.06
2.17
2.19
6.14
3.33
9.5 9.0 8.5 8.0 7.5 7.0 6.5 6.0 5.5 5.0 4.5 4.0 3.5 3.0 2.5 2.0 1.5 1.0 0.5 0.0

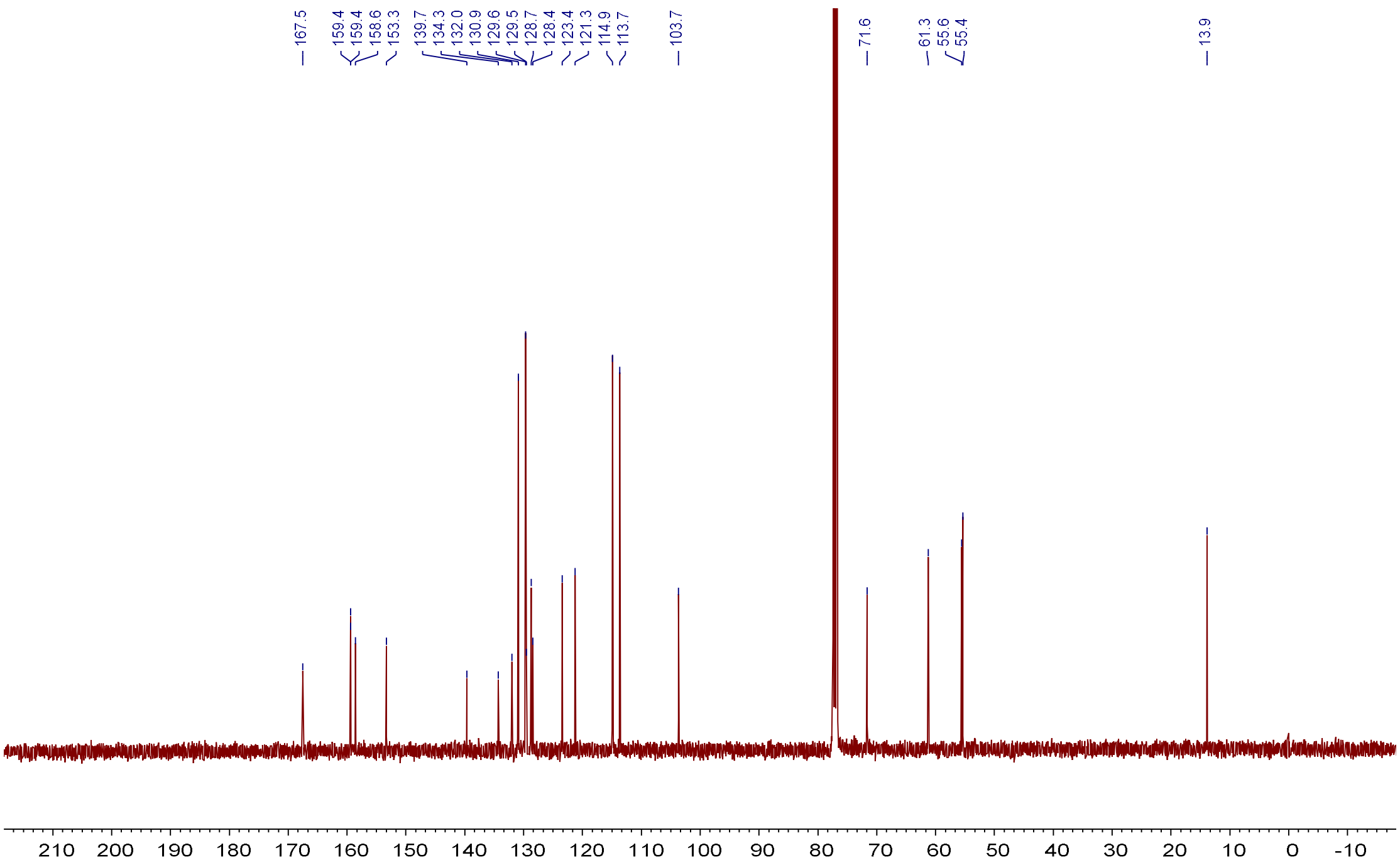
167.5
159.4
159.4
158.6
153.3
139.7
134.3
132.0
130.9
129.6
129.5
128.7
128.4
123.4
121.3
114.9
113.7
103.7
71.6
61.3
55.6
55.4
13.9
210 200 190 180 170 160 150 140 130 120 110 100 90 80 70 60 50 40 30 20 10 0 -10

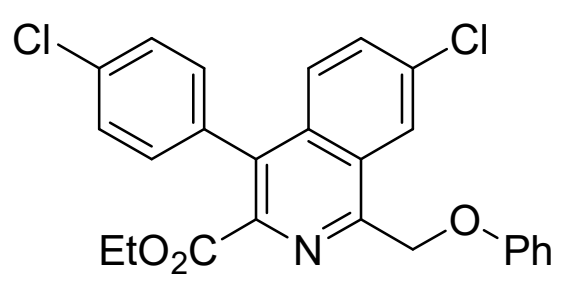
Cl
Cl
EtO2C
N
O
Ph

4s

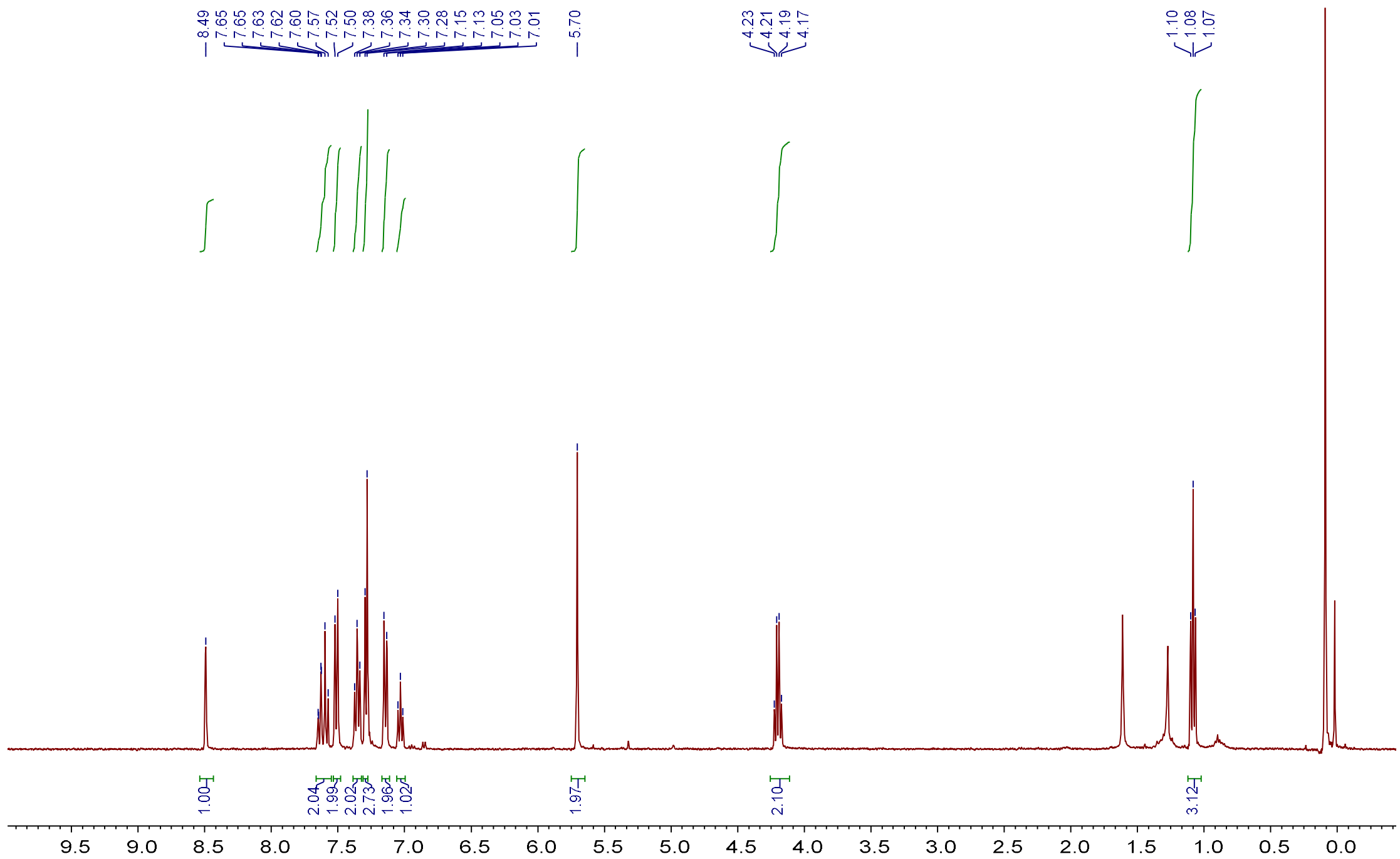
8.49
7.65
7.63
7.62
7.60
7.57
7.52
7.50
7.38
7.36
7.34
7.30
7.28
7.15
7.13
7.05
7.03
7.01
5.70
4.23
4.21
4.19
4.17
1.10
1.08
1.07
1.00
2.04
1.99
2.02
2.73
1.96
1.02
1.97
2.10
3.12
9.5 9.0 8.5 8.0 7.5 7.0 6.5 6.0 5.5 5.0 4.5 4.0 3.5 3.0 2.5 2.0 1.5 1.0 0.5 0.0

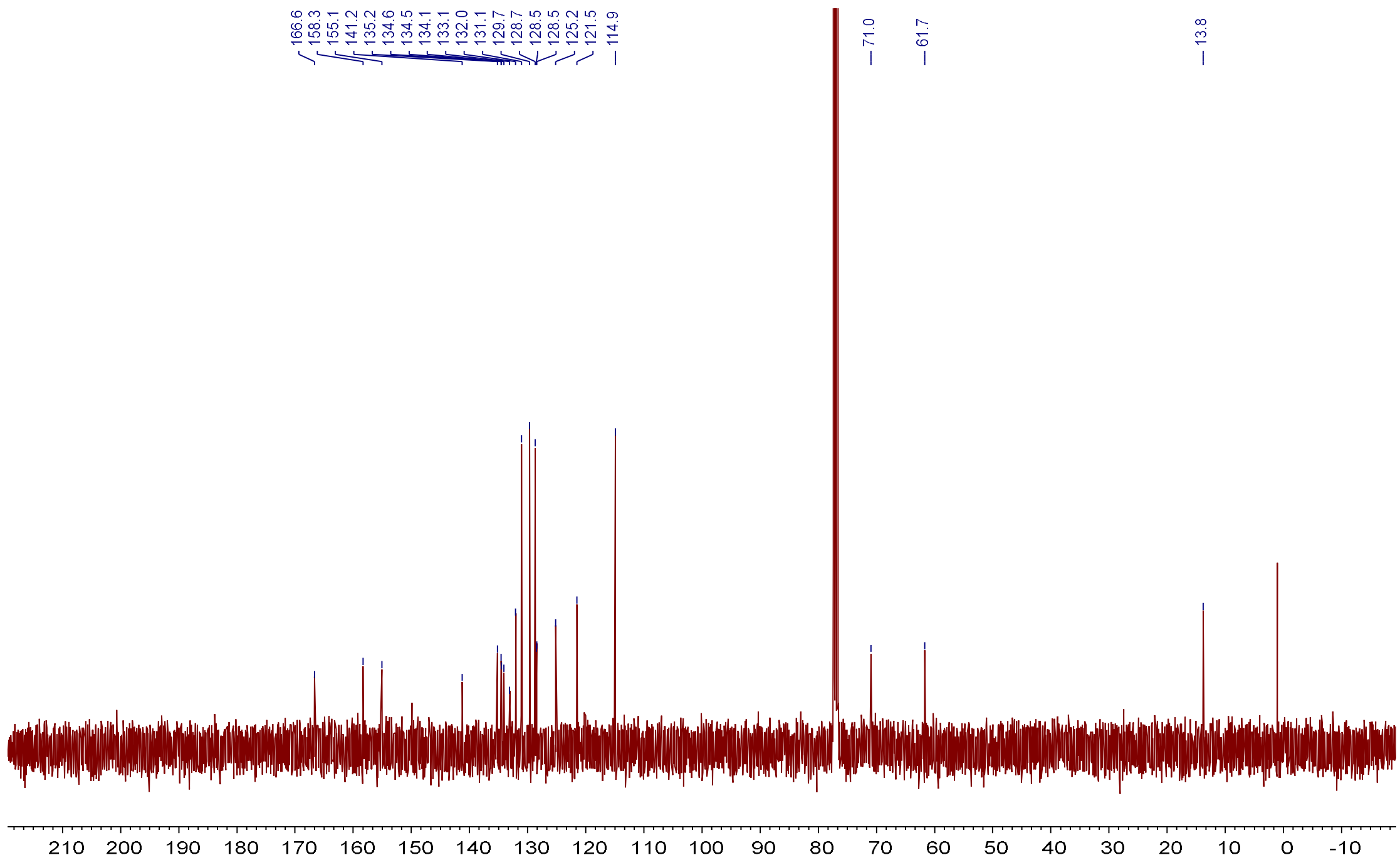
166.6
158.3
155.1
141.2
135.2
134.6
134.5
134.1
133.1
132.0
131.1
129.7
128.7
128.5
125.2
121.5
114.9
71.0
61.7
13.8
210 200 190 180 170 160 150 140 130 120 110 100 90 80 70 60 50 40 30 20 10 0 -10

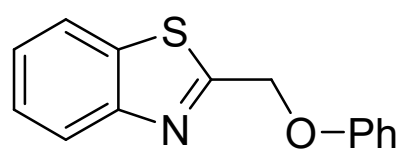

4t

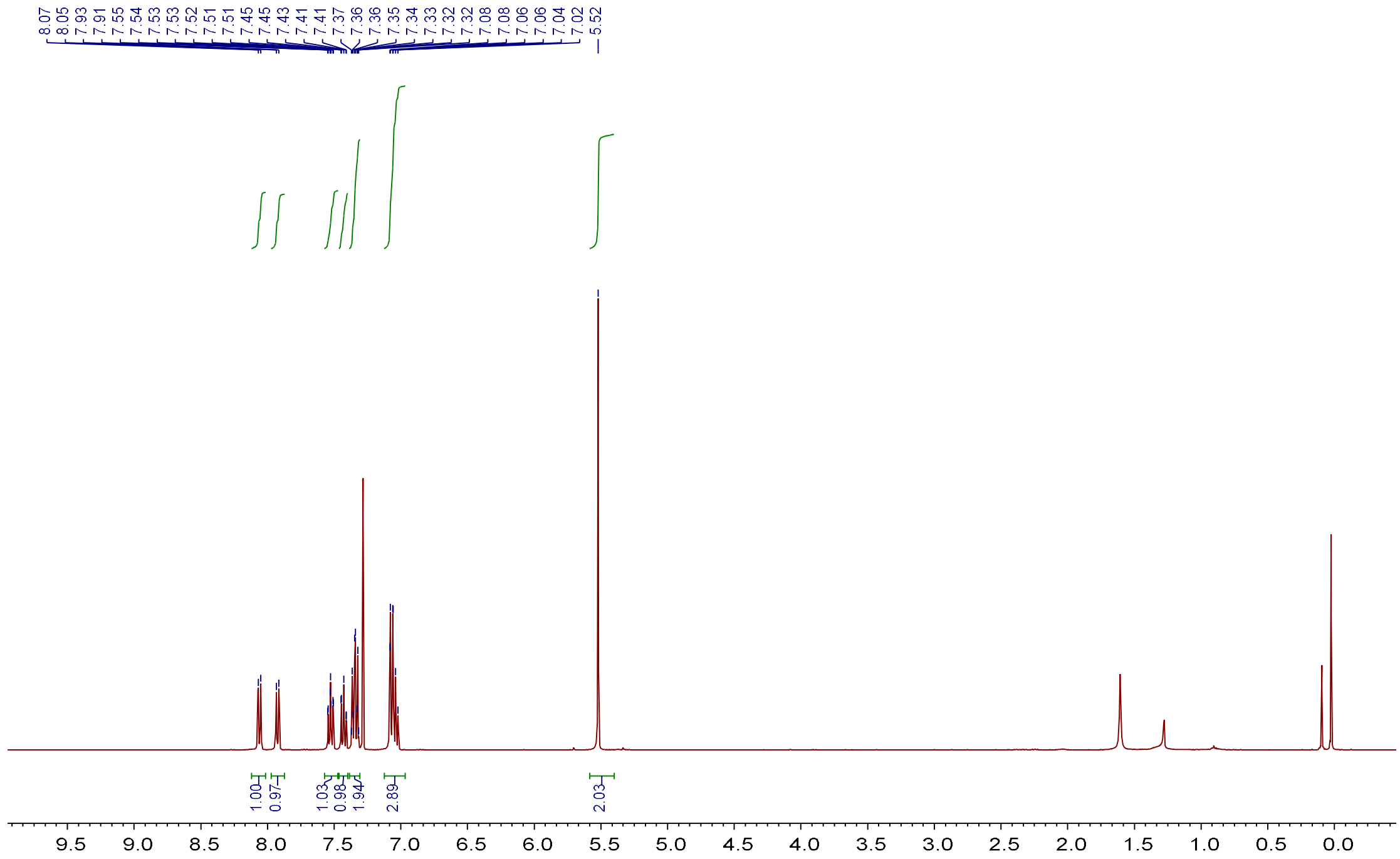

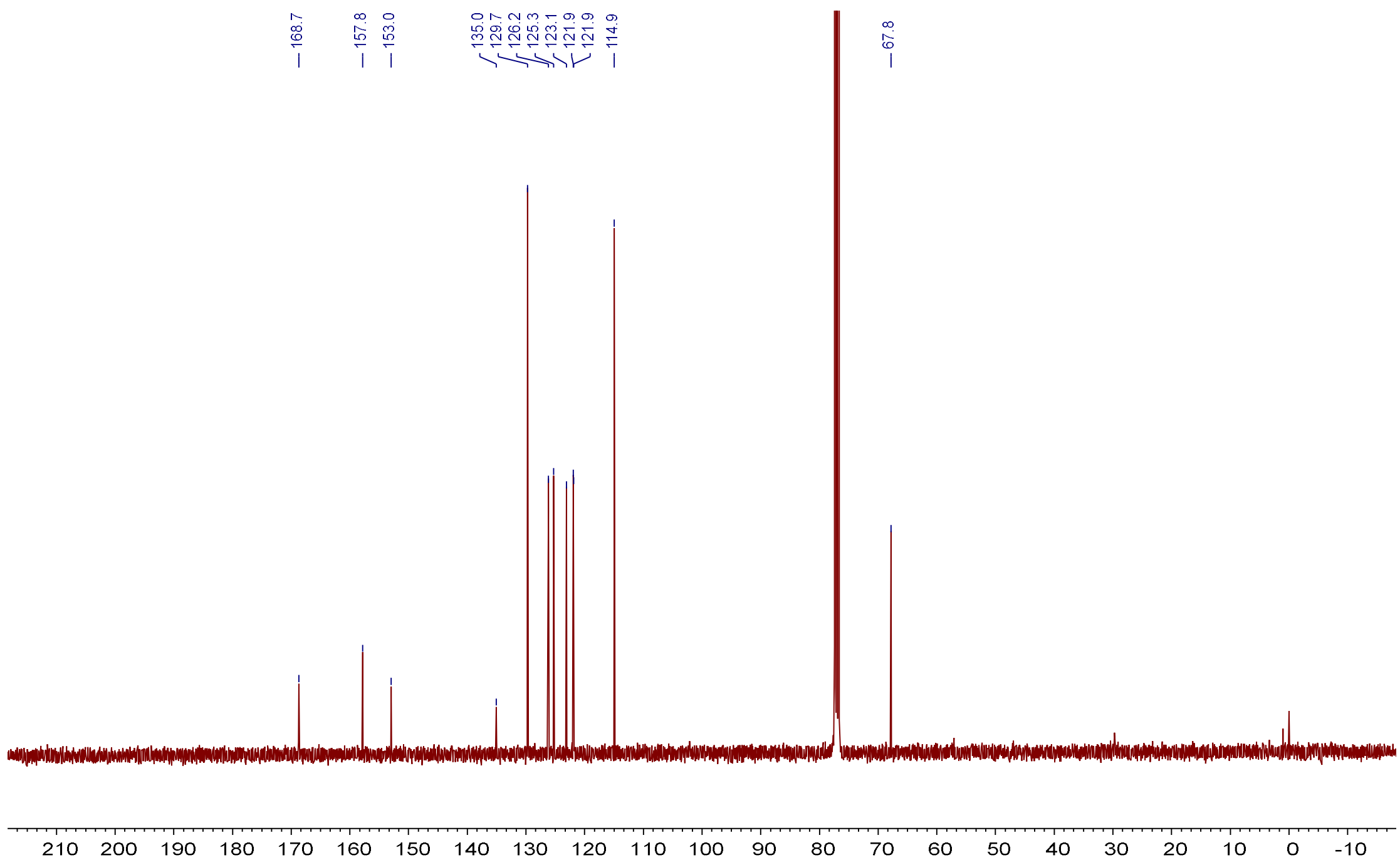

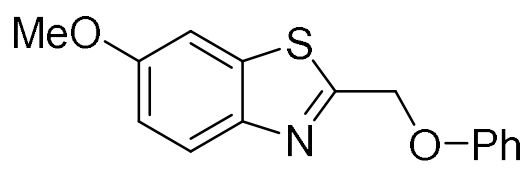

4u

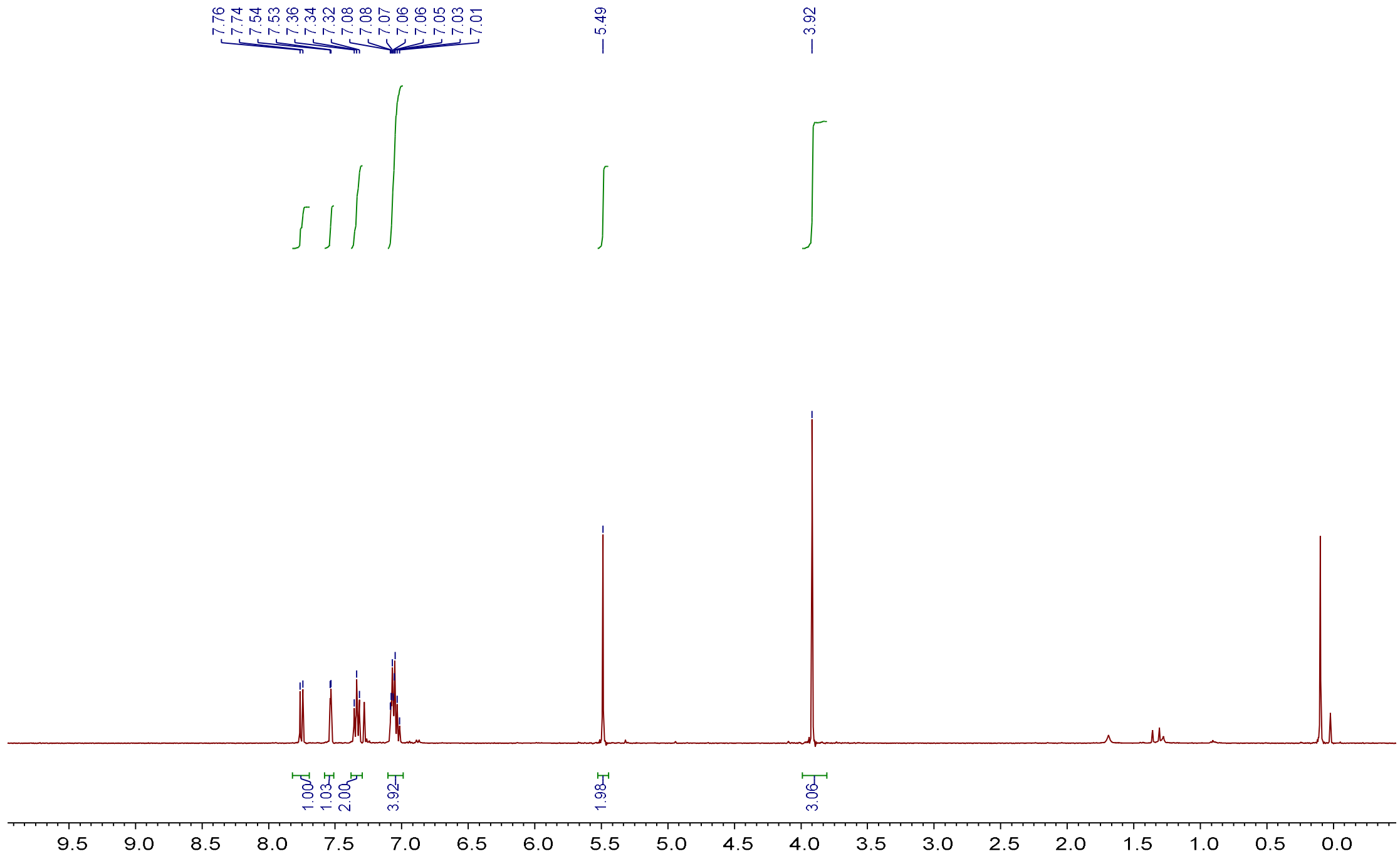

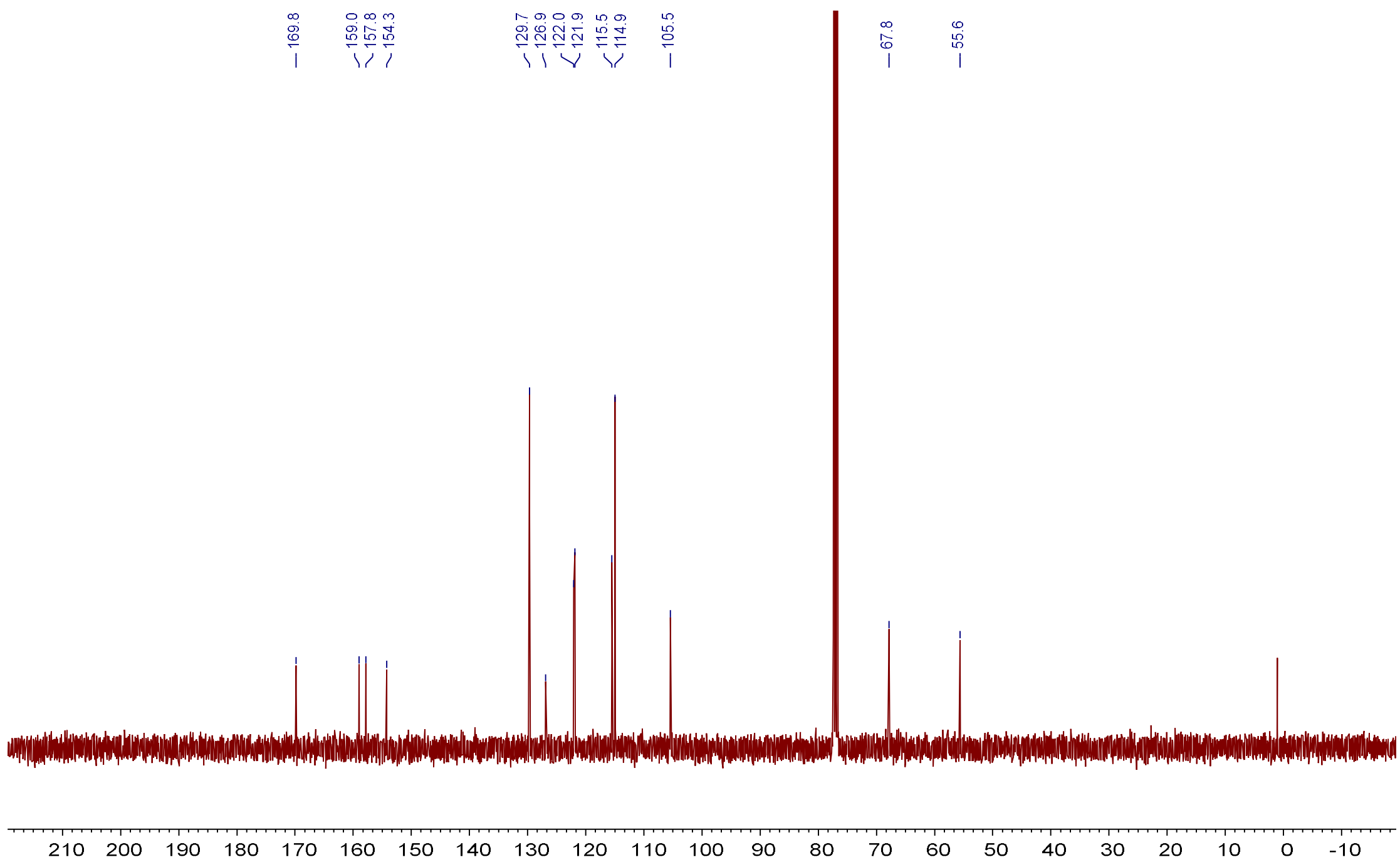

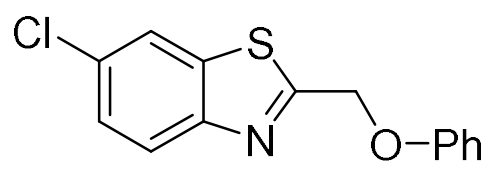
Cl
S
N
O–Ph

4v

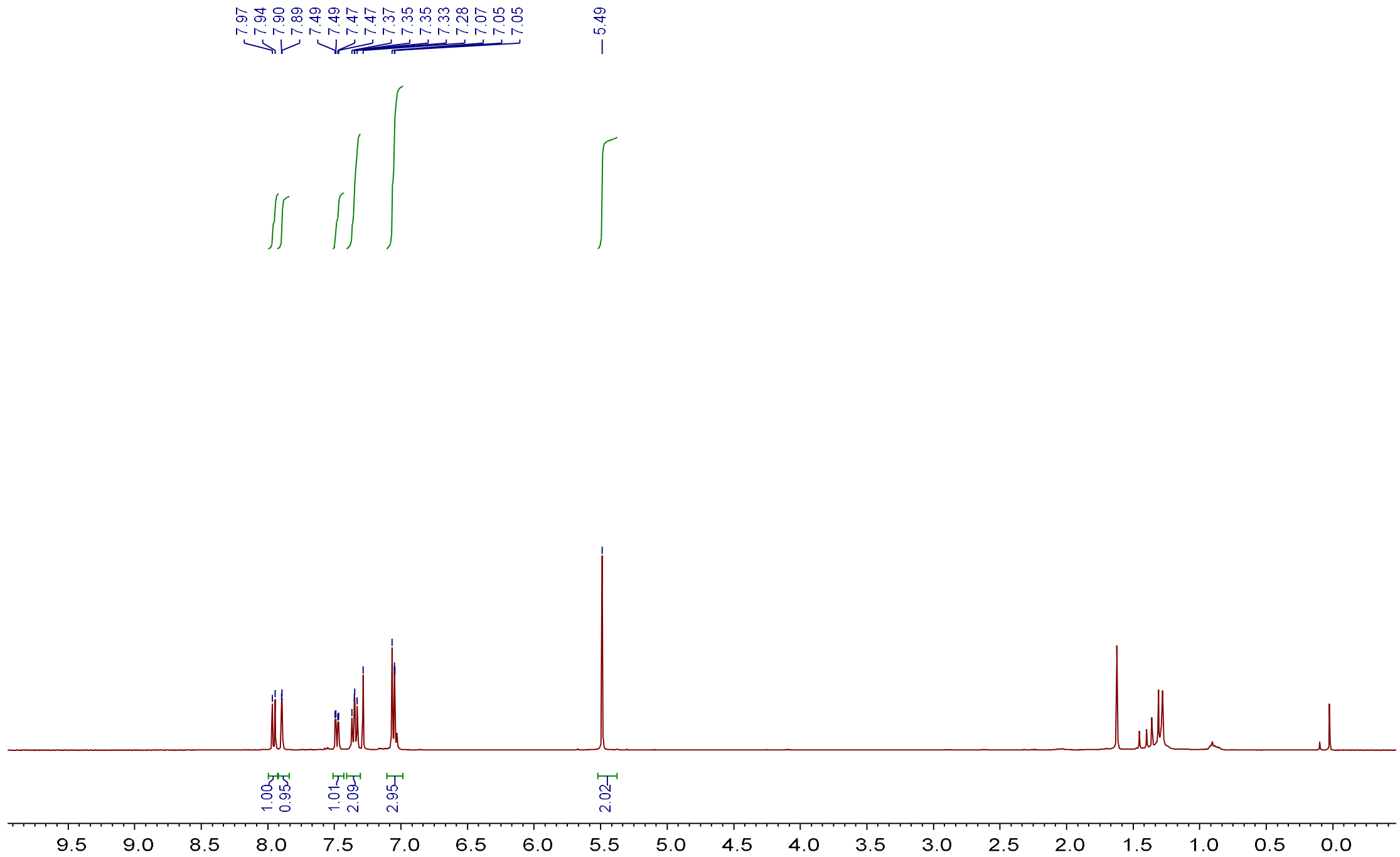
7.97
7.94
7.90
7.89
7.49
7.47
7.47
7.37
7.35
7.35
7.33
7.28
7.07
7.05
7.05
5.49
1.00
0.95
1.01
2.09
2.95
2.02
9.5 9.0 8.5 8.0 7.5 7.0 6.5 6.0 5.5 5.0 4.5 4.0 3.5 3.0 2.5 2.0 1.5 1.0 0.5 0.0

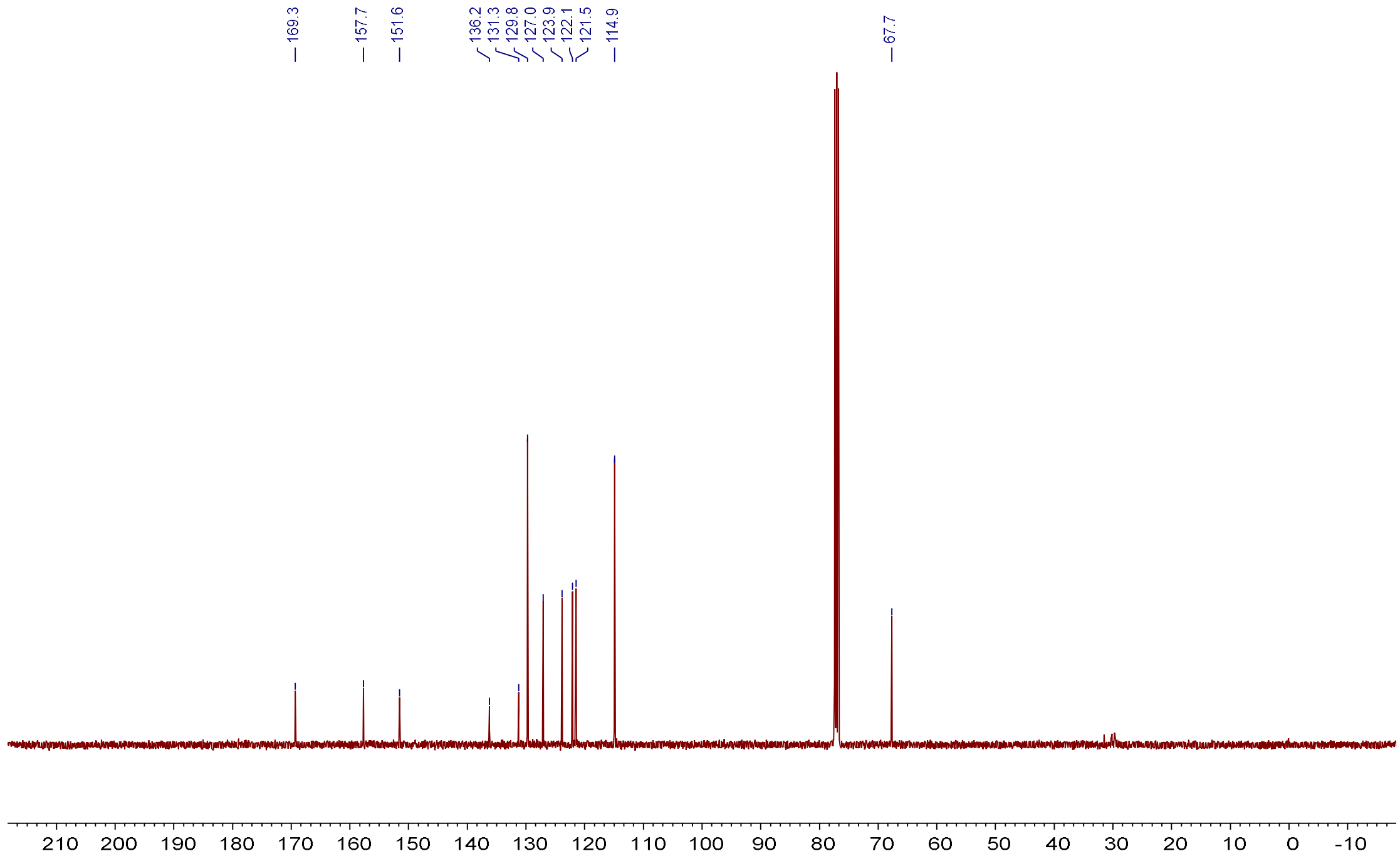
169.3
157.7
151.6
136.2
131.3
129.8
127.0
123.9
122.1
121.5
114.9
67.7
210 200 190 180 170 160 150 140 130 120 110 100 90 80 70 60 50 40 30 20 10 0 -10

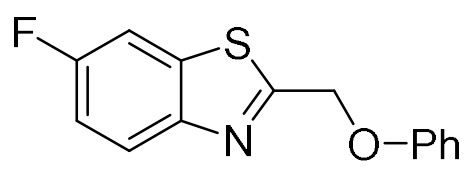

4w

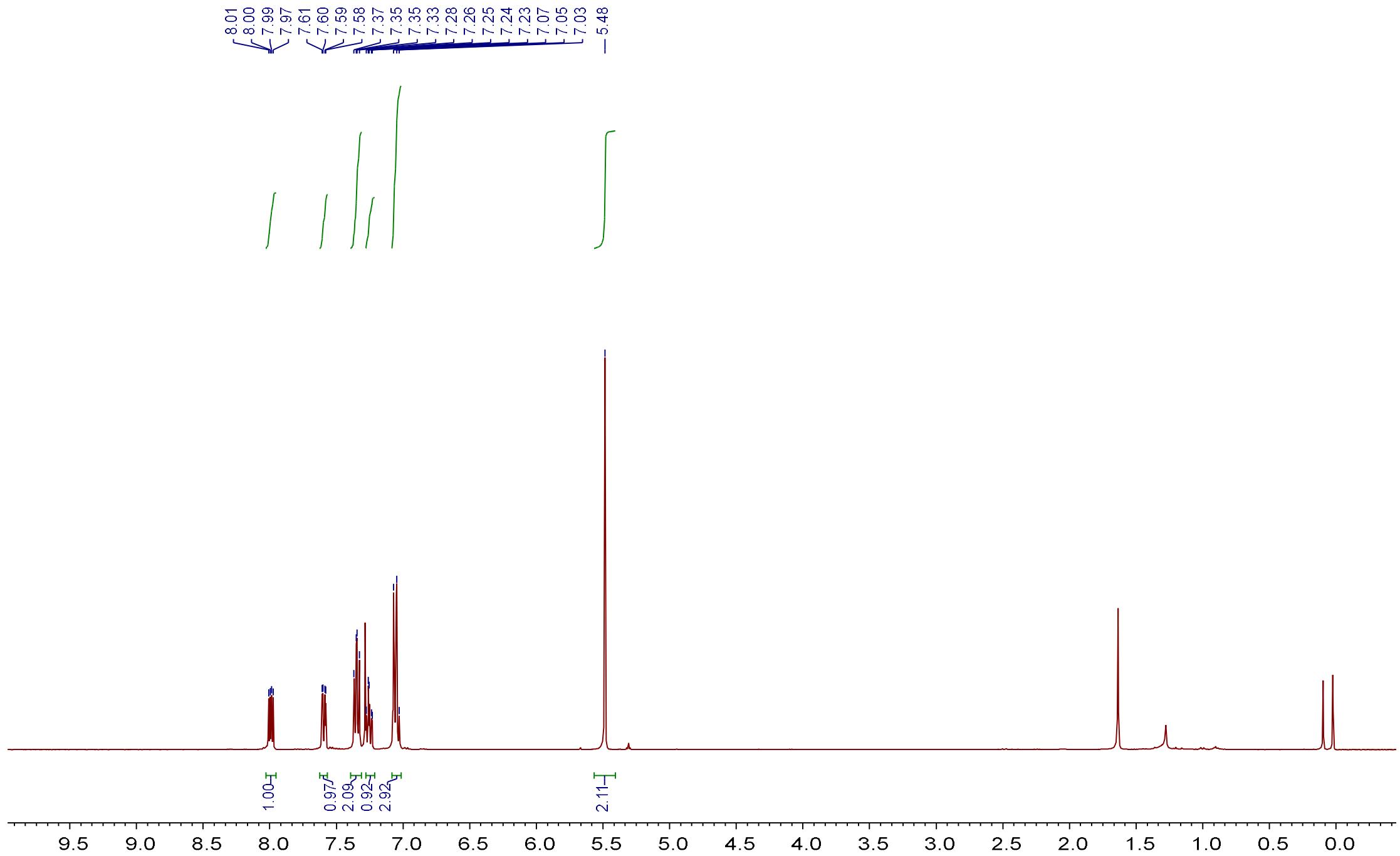

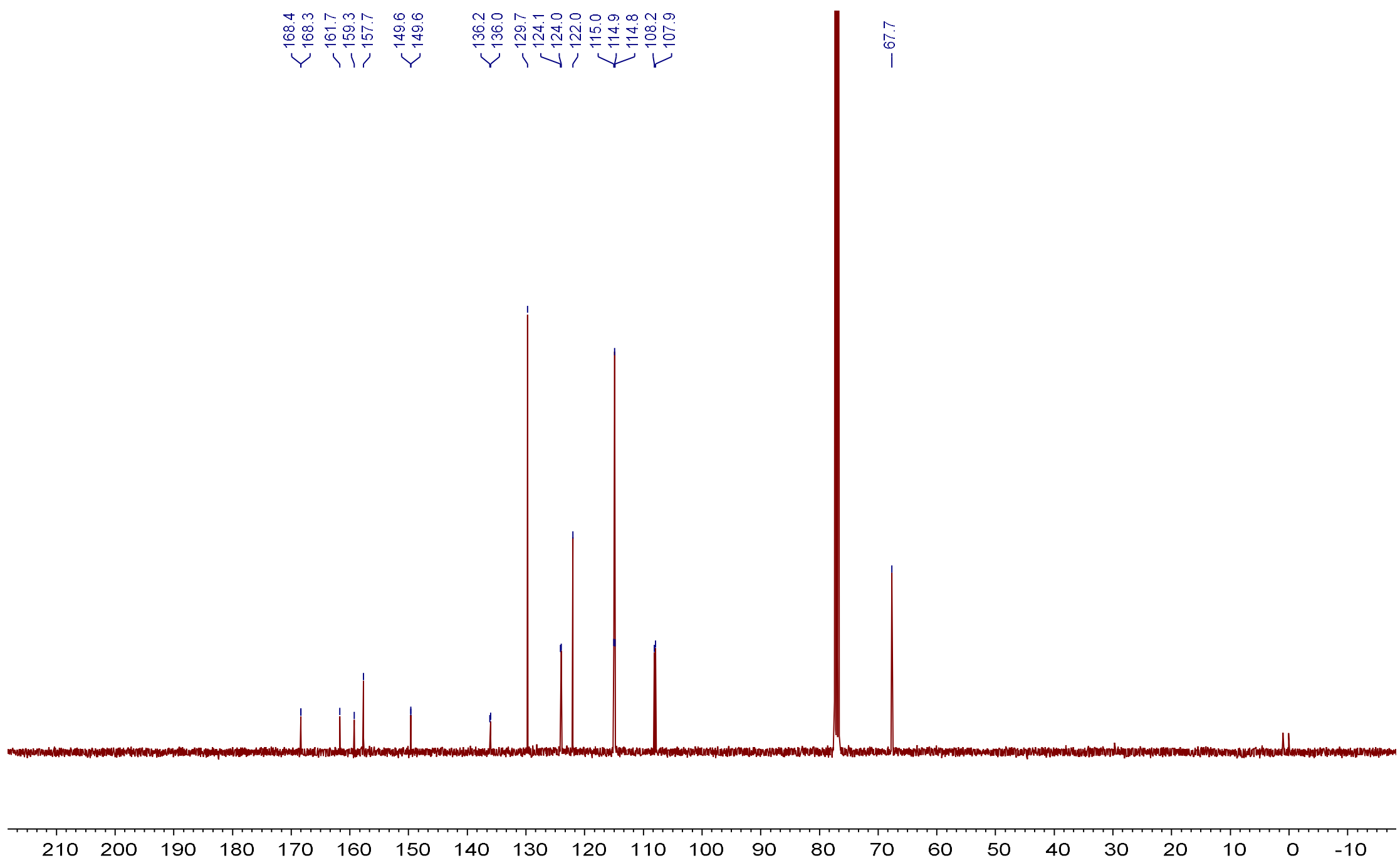

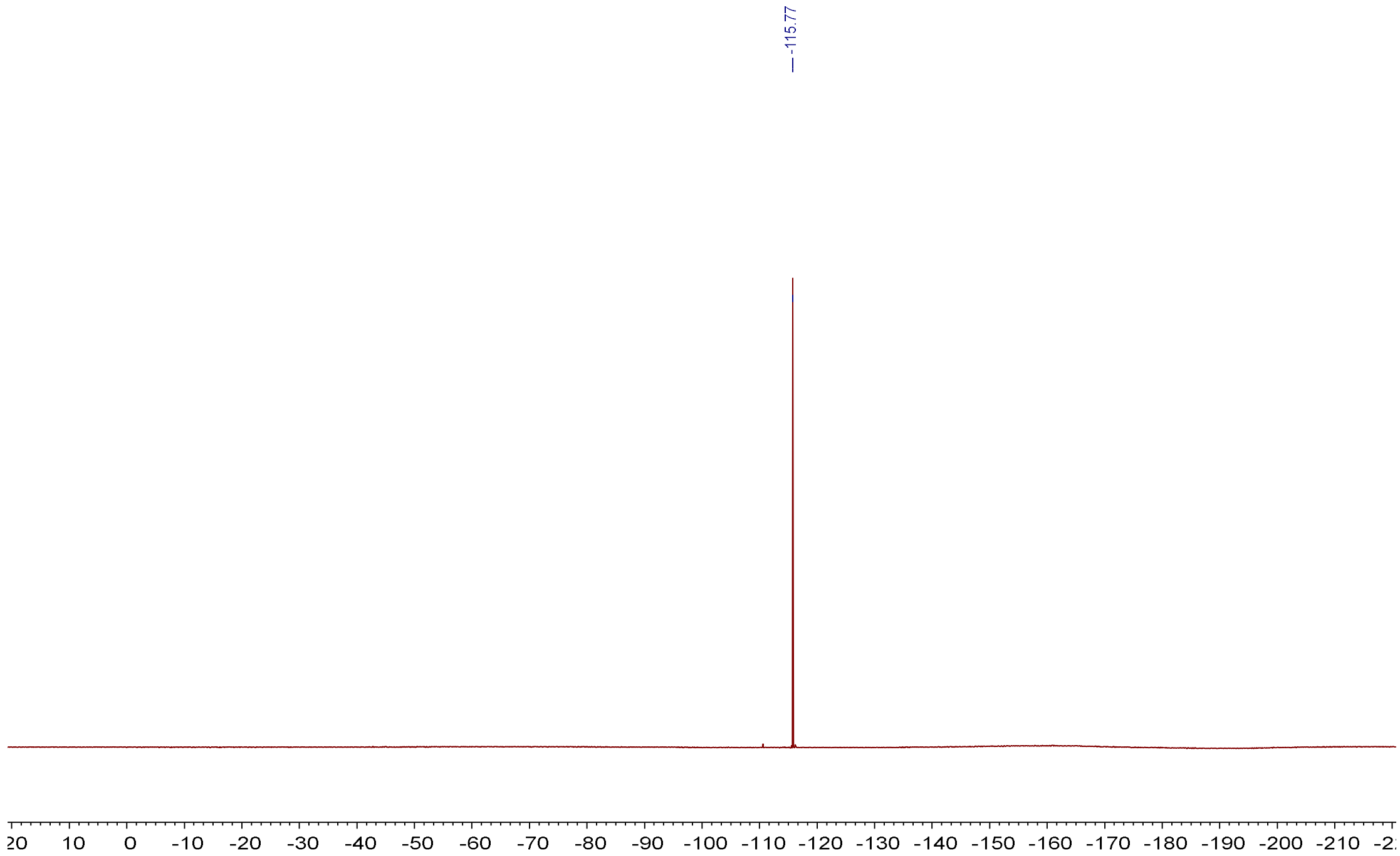

-115.77
20 10 0 -10 -20 -30 -40 -50 -60 -70 -80 -90 -100 -110 -120 -130 -140 -150 -160 -170 -180 -190 -200 -210

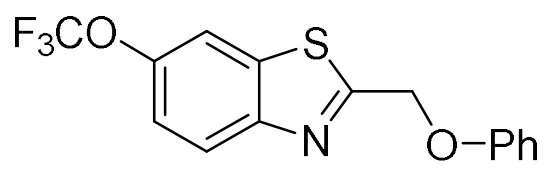

4x

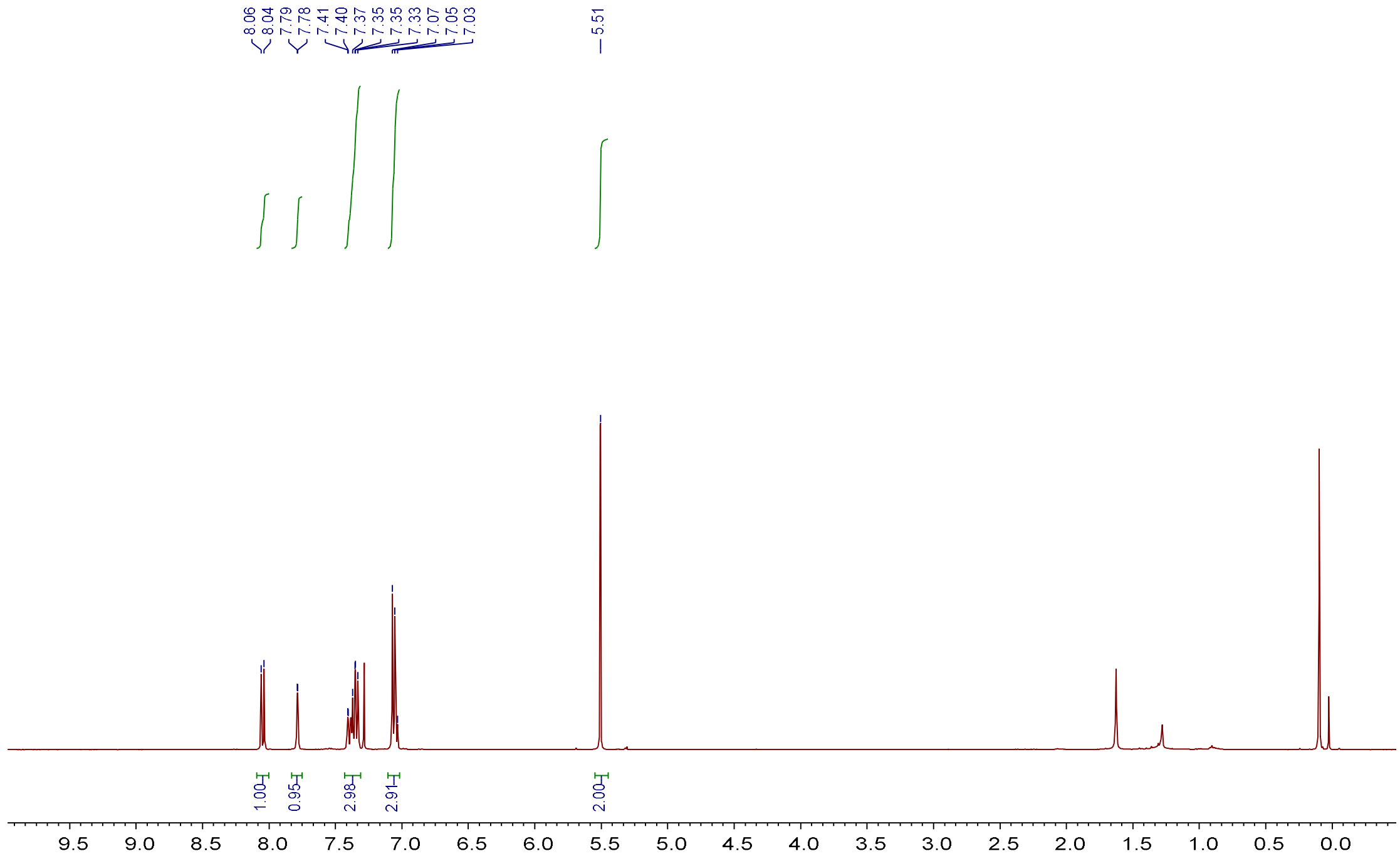

8.06
8.04
7.79
7.78
7.41
7.40
7.37
7.35
7.35
7.33
7.07
7.05
7.03
5.51
1.00
0.95
2.98
2.91
2.00
9.5 9.0 8.5 8.0 7.5 7.0 6.5 6.0 5.5 5.0 4.5 4.0 3.5 3.0 2.5 2.0 1.5 1.0 0.5 0.0

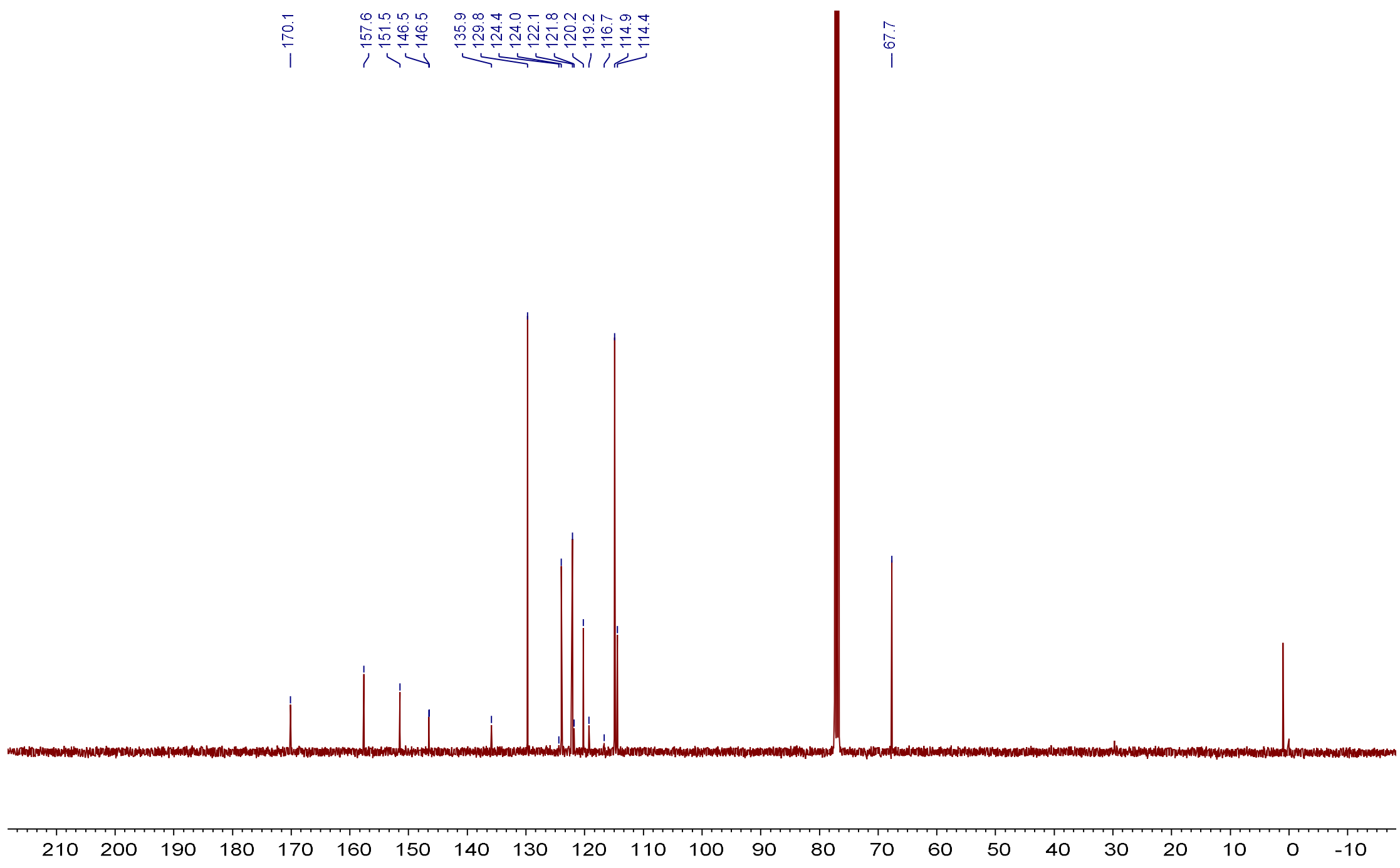

170.1
157.6
151.5
146.5
146.5
135.9
129.8
124.4
124.0
122.1
121.8
120.2
119.2
116.7
114.9
114.4
67.7
210 200 190 180 170 160 150 140 130 120 110 100 90 80 70 60 50 40 30 20 10 0 -10

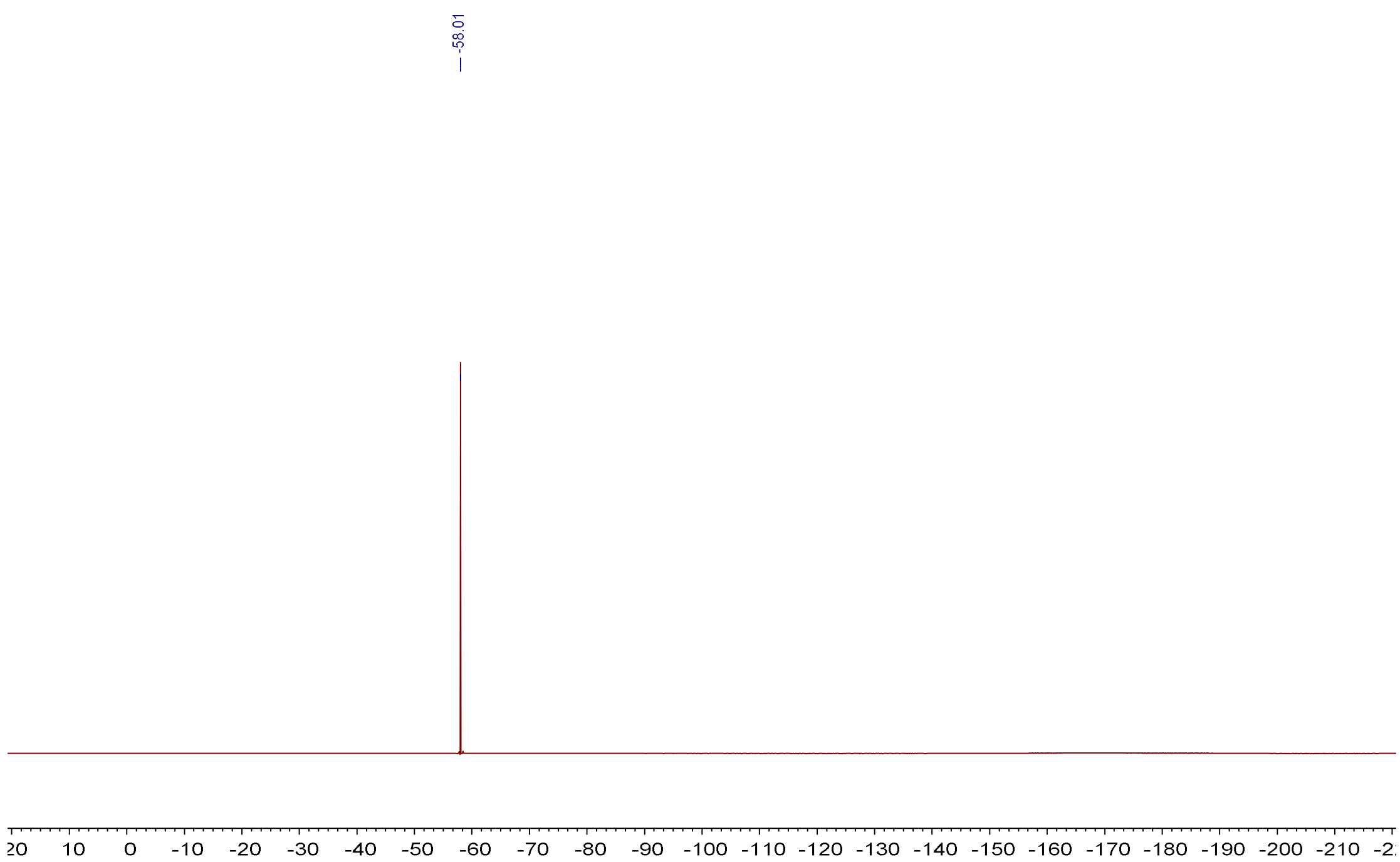

-58.01
20 10 0 -10 -20 -30 -40 -50 -60 -70 -80 -90 -100 -110 -120 -130 -140 -150 -160 -170 -180 -190 -200 -210

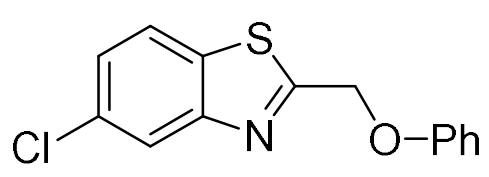

4y

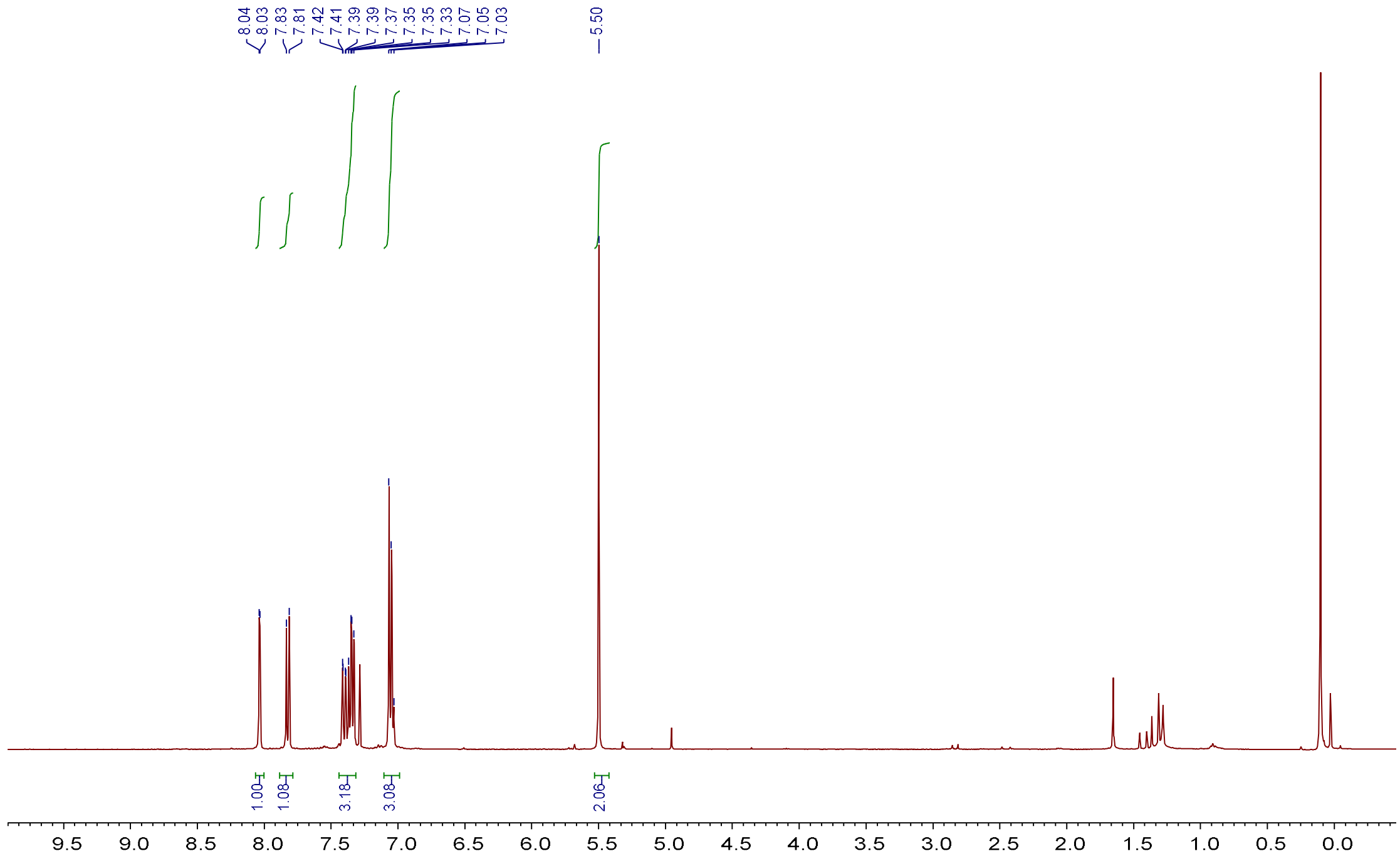

8.04
8.03
7.83
7.81
7.42
7.41
7.39
7.39
7.37
7.35
7.35
7.33
7.07
7.05
7.03
5.50
1.00
1.08
3.18
3.08
2.06
9.5
9.0
8.5
8.0
7.5
7.0
6.5
6.0
5.5
5.0
4.5
4.0
3.5
3.0
2.5
2.0
1.5
1.0
0.5
0.0

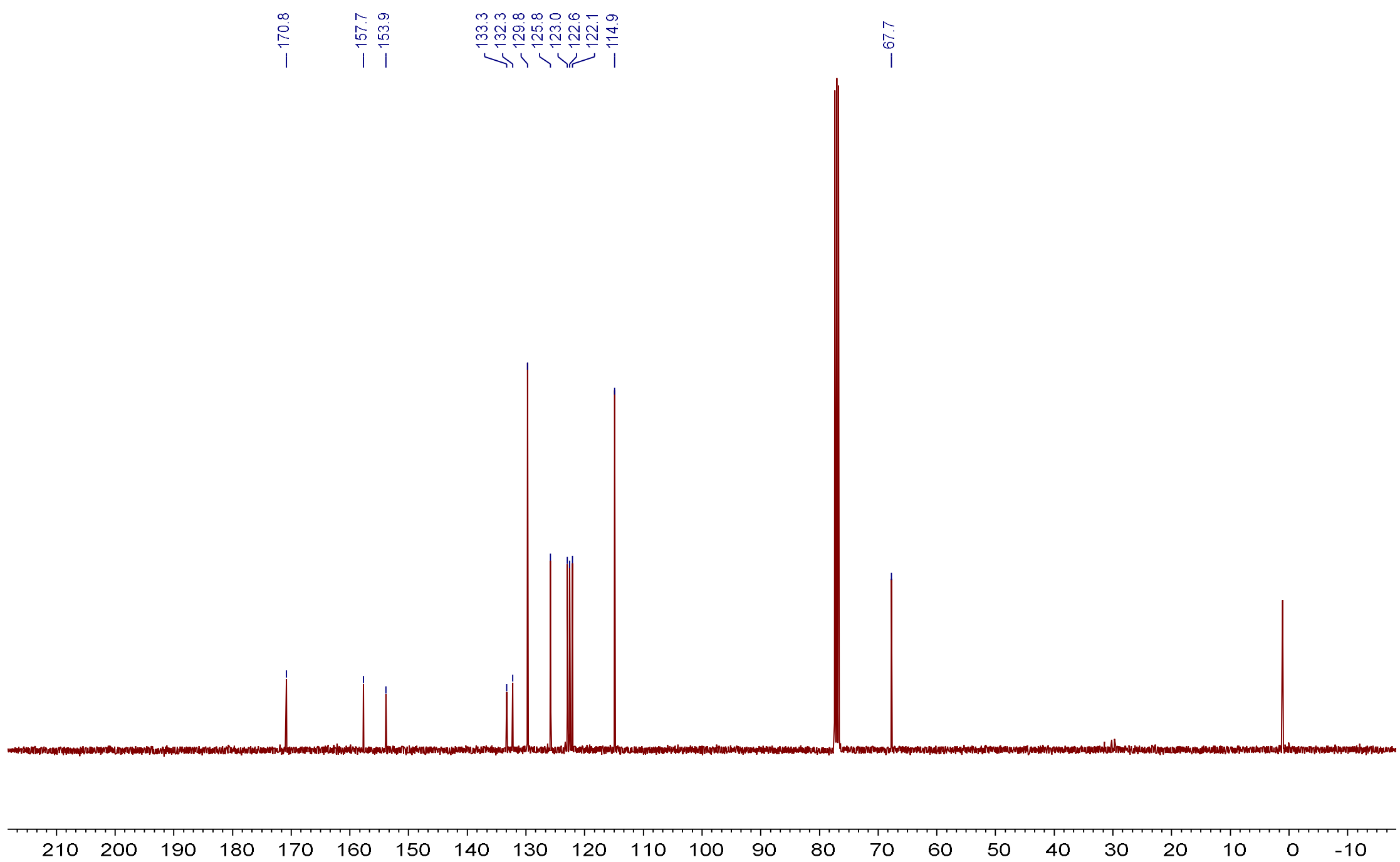

170.8
157.7
153.9
133.3
132.3
129.8
125.8
123.0
122.6
122.1
114.9
67.7
210
200
190
180
170
160
150
140
130
120
110
100
90
80
70
60
50
40
30
20
10
0
-10

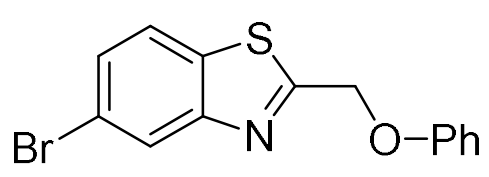

4z

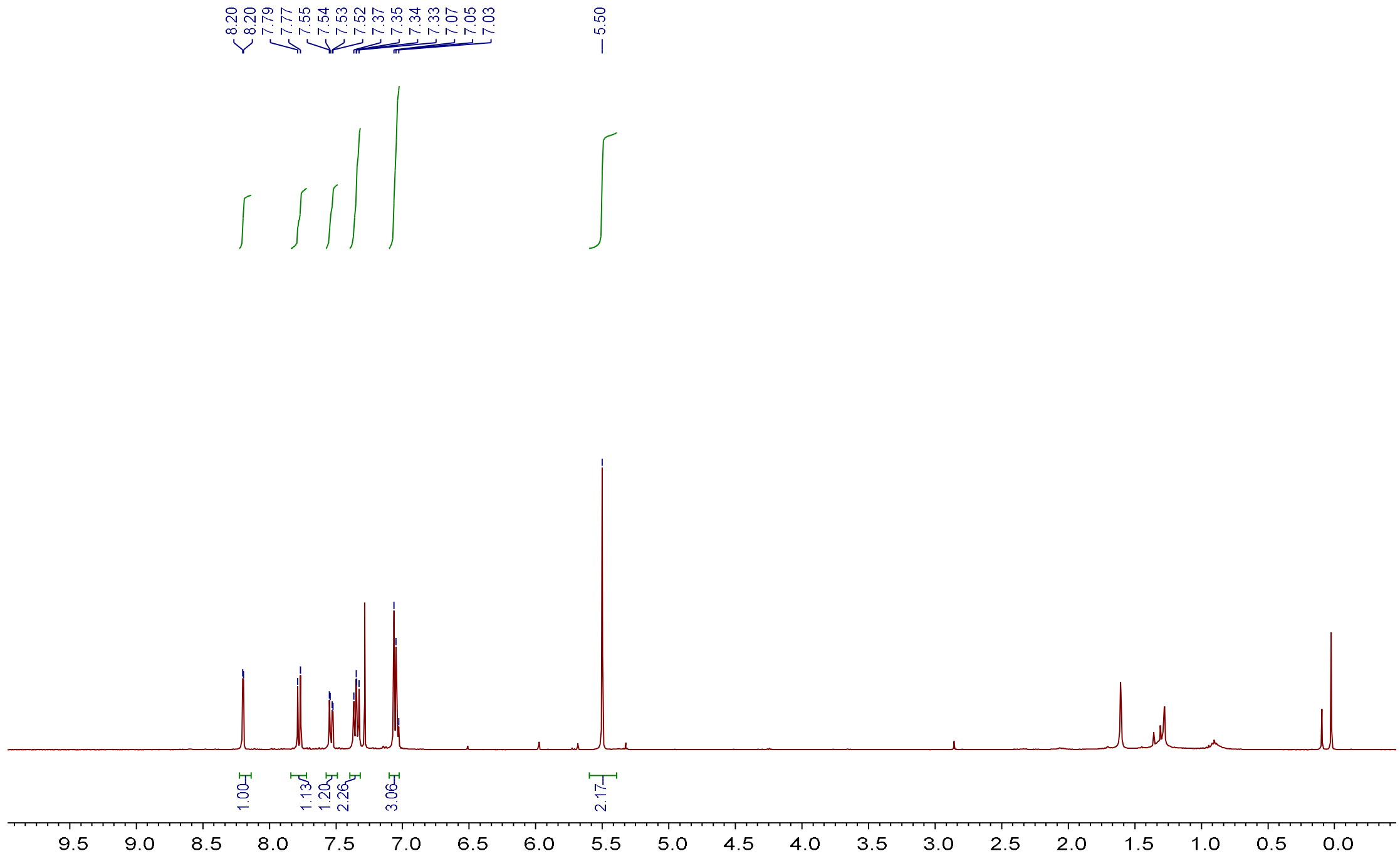

8.20
8.20
7.79
7.77
7.55
7.54
7.53
7.52
7.37
7.35
7.34
7.33
7.07
7.05
7.03
5.50
1.00
1.13
1.20
2.26
3.06
2.17
9.5
9.0
8.5
8.0
7.5
7.0
6.5
6.0
5.5
5.0
4.5
4.0
3.5
3.0
2.5
2.0
1.5
1.0
0.5
0.0

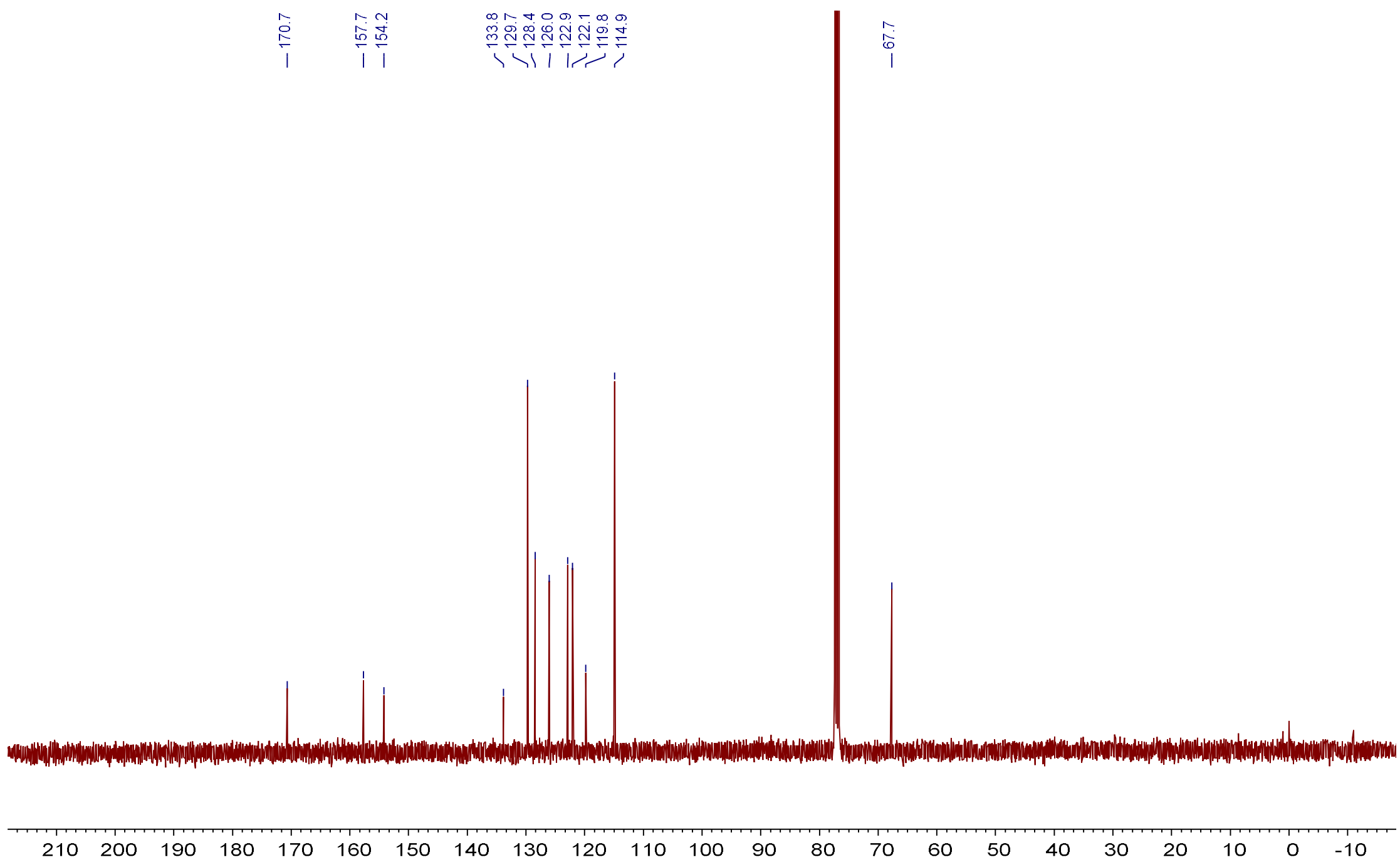

170.7
157.7
154.2
133.8
129.7
128.4
126.0
122.9
122.1
119.8
114.9
67.7
210
200
190
180
170
160
150
140
130
120
110
100
90
80
70
60
50
40
30
20
10
0
-10

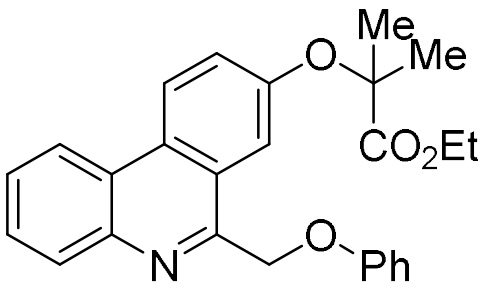
Me
Me
O
CO2Et
N
O
Ph

4aa

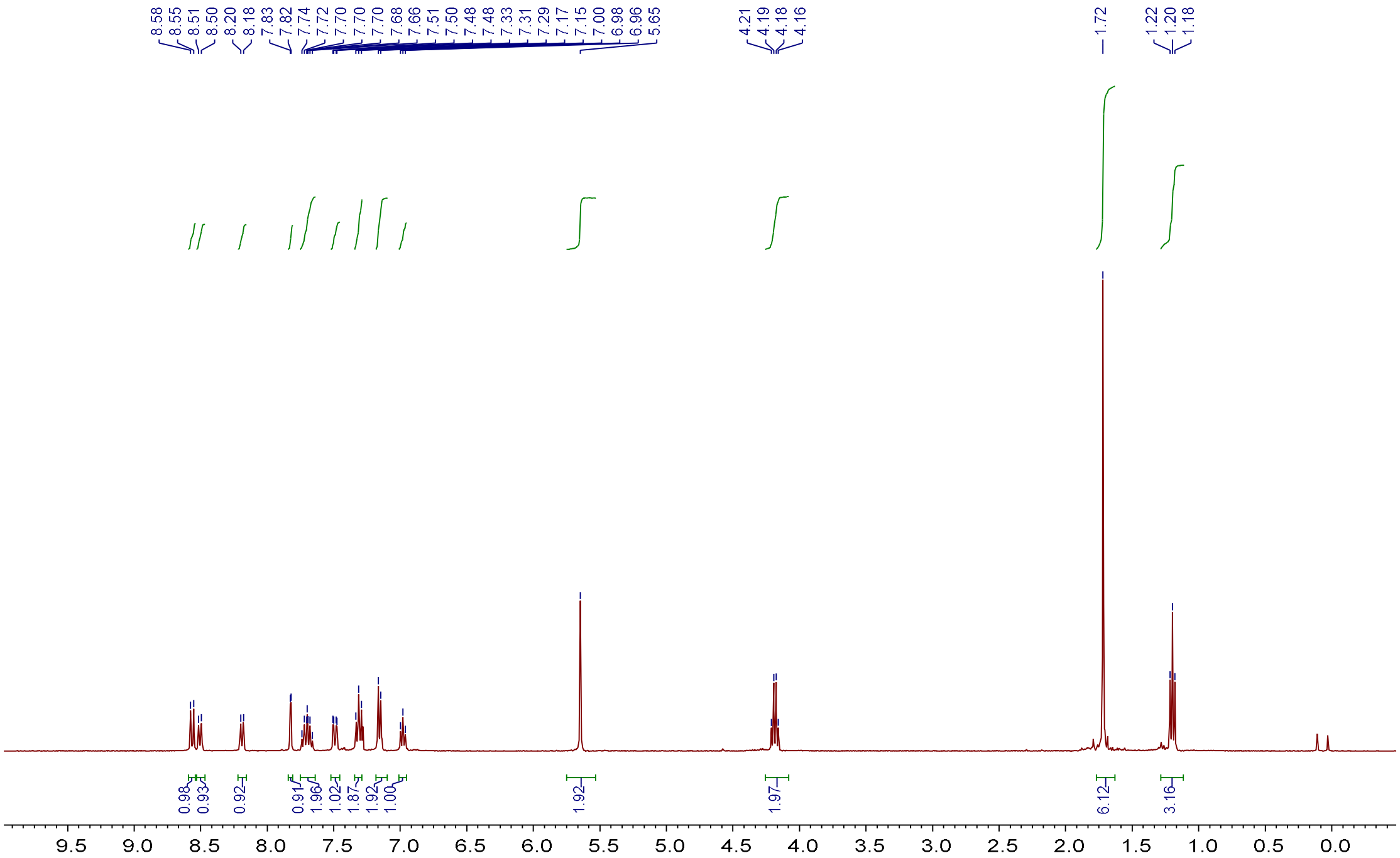
8.58
8.55
8.51
8.50
8.20
8.18
7.83
7.82
7.74
7.72
7.70
7.70
7.70
7.68
7.66
7.51
7.50
7.48
7.48
7.33
7.31
7.29
7.17
7.15
7.00
6.98
6.96
5.65
4.21
4.19
4.18
4.16
1.72
1.22
1.20
1.18
0.98
0.93
0.92
0.91
1.96
1.02
1.87
1.92
1.00
1.92
1.97
6.12
3.16
9.5
9.0
8.5
8.0
7.5
7.0
6.5
6.0
5.5
5.0
4.5
4.0
3.5
3.0
2.5
2.0
1.5
1.0
0.5
0.0

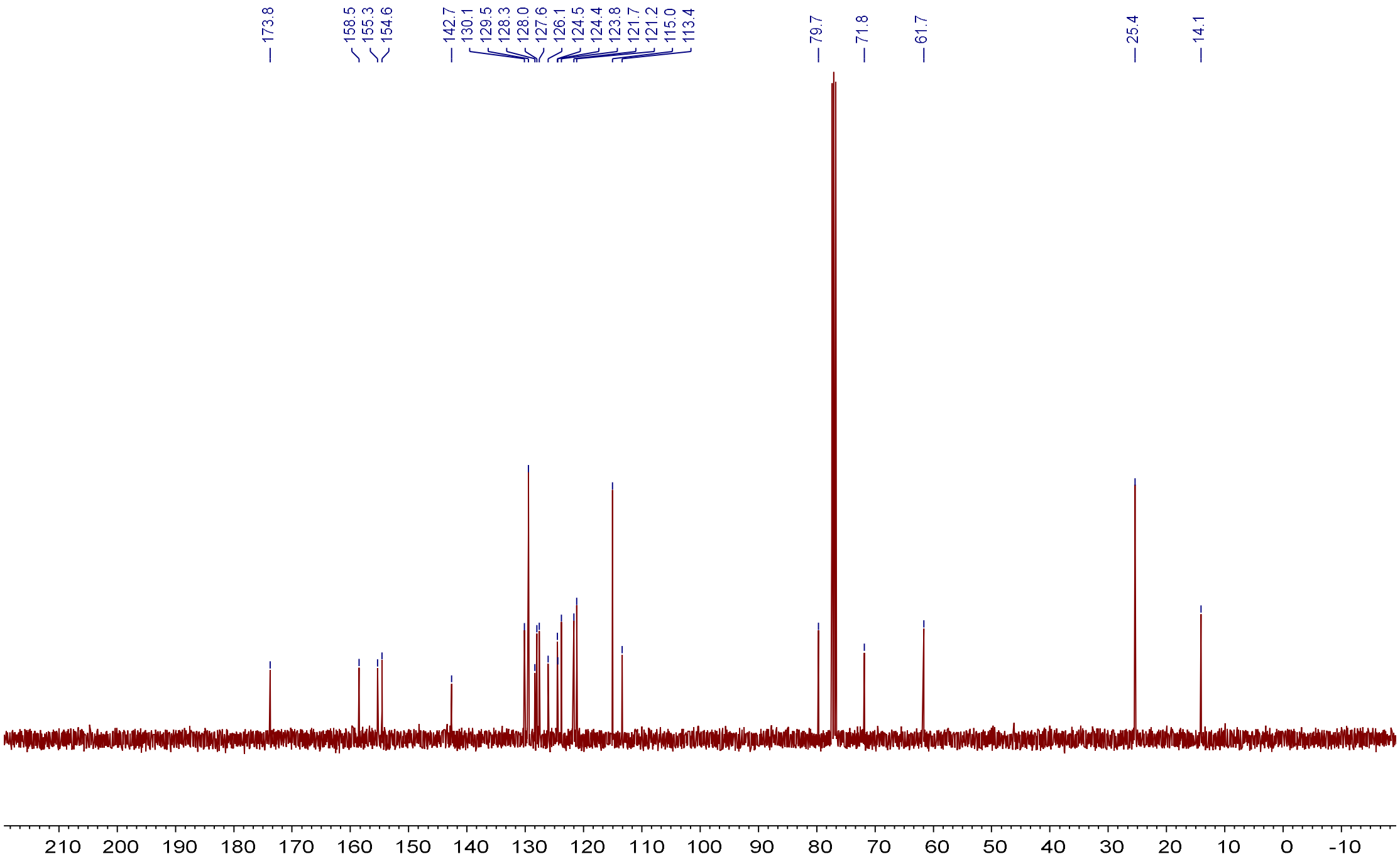
173.8
158.5
155.3
154.6
142.7
130.1
129.5
128.3
128.0
127.6
126.1
124.5
124.4
123.8
121.7
121.2
115.0
113.4
79.7
71.8
61.7
25.4
14.1
210
200
190
180
170
160
150
140
130
120
110
100
90
80
70
60
50
40
30
20
10
0
-10

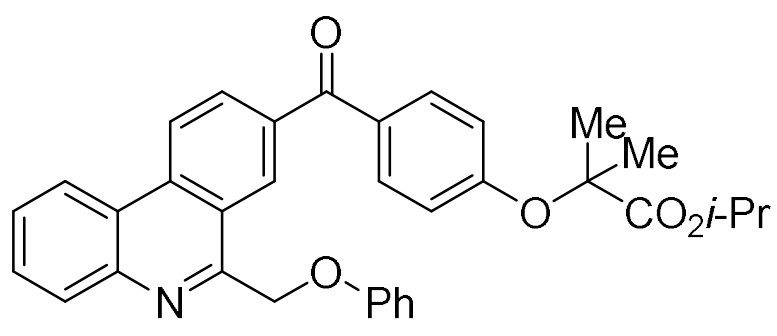
O
Me
Me
O
CO2i-Pr
N
O
Ph

4ab

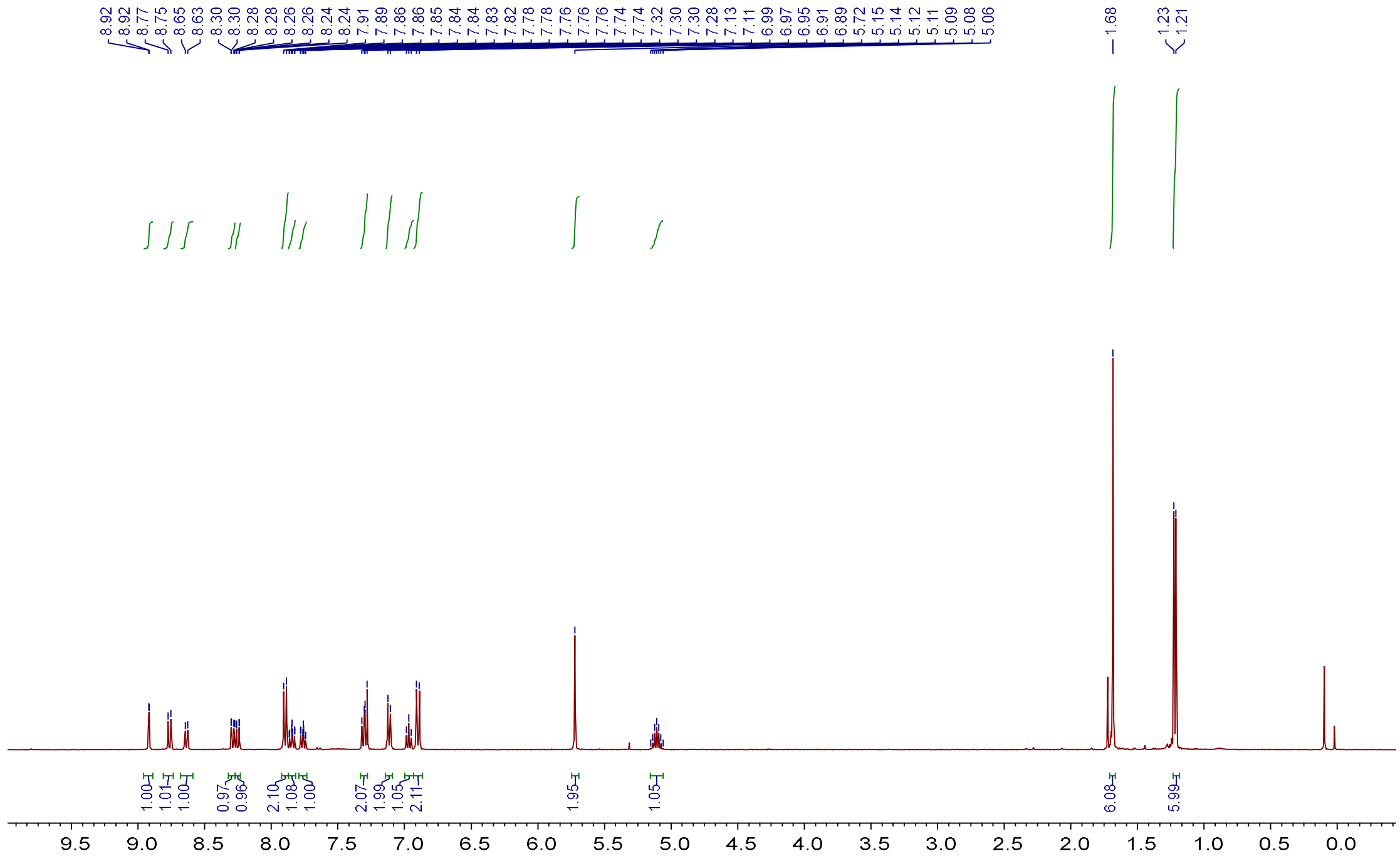
8.92 8.92 8.77 8.75 8.65 8.63 8.30 8.30 8.28 8.28 8.26 8.26 8.24 8.24 7.91 7.89 7.86 7.86 7.85 7.84 7.84 7.83 7.82 7.78 7.78 7.76 7.76 7.76 7.74 7.74 7.32 7.30 7.30 7.28 7.13 7.11 6.99 6.97 6.95 6.91 6.89 5.72 5.15 5.14 5.12 5.11 5.09 5.08 5.06
1.68
1.23 1.21
1.00 1.01 1.00 0.97 0.96 2.10 1.08 1.00 2.07 1.99 1.05 2.11 1.95 1.05 6.08 5.99
9.5 9.0 8.5 8.0 7.5 7.0 6.5 6.0 5.5 5.0 4.5 4.0 3.5 3.0 2.5 2.0 1.5 1.0 0.5 0.0

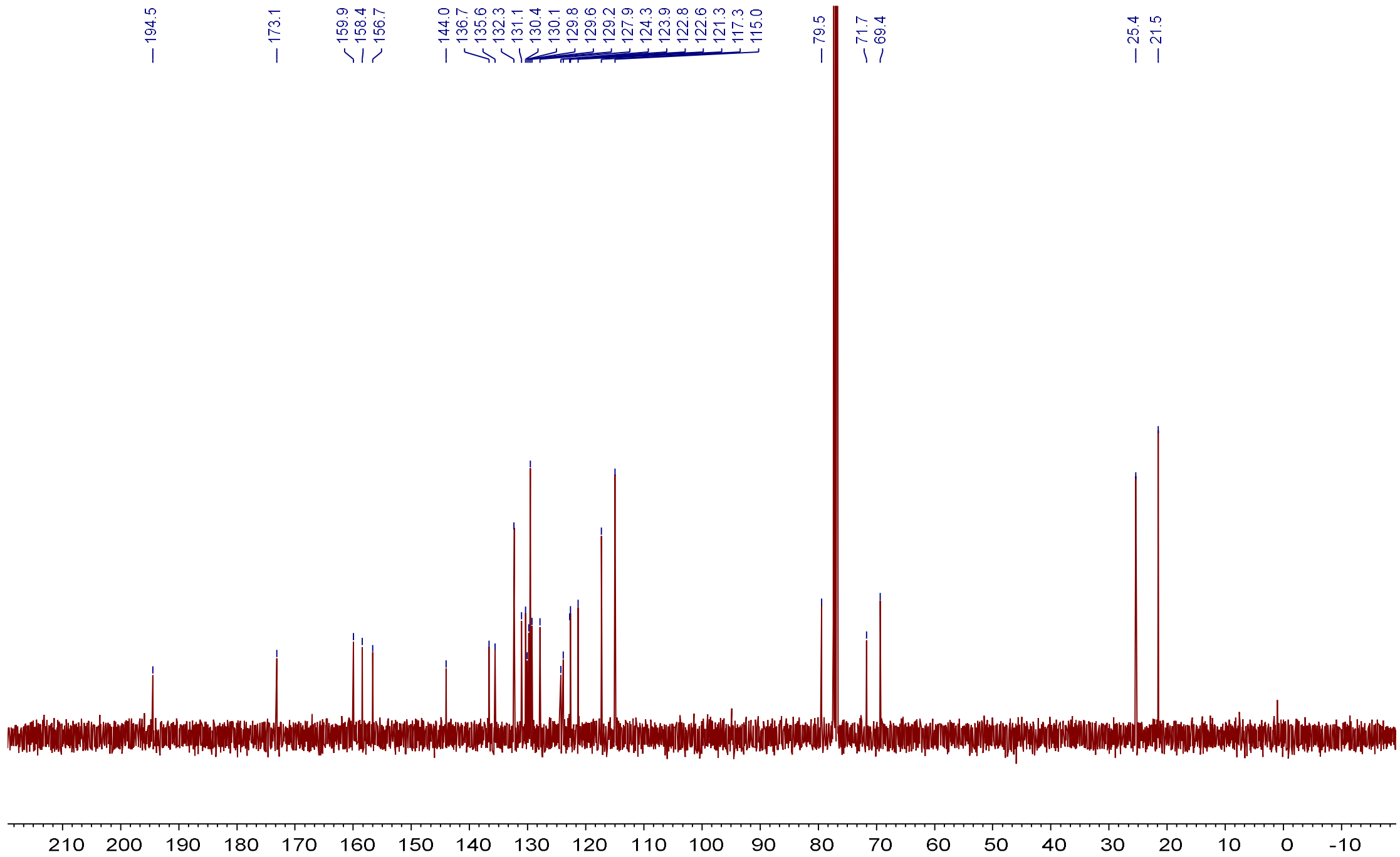
194.5
173.1
159.9 158.4 156.7
144.0 136.7 135.6 132.3 131.1 130.4 130.1 129.8 129.6 129.2 127.9 124.3 123.9 122.8 122.6 121.3 117.3 115.0
79.5
71.7 69.4
25.4 21.5
210 200 190 180 170 160 150 140 130 120 110 100 90 80 70 60 50 40 30 20 10 0 -10

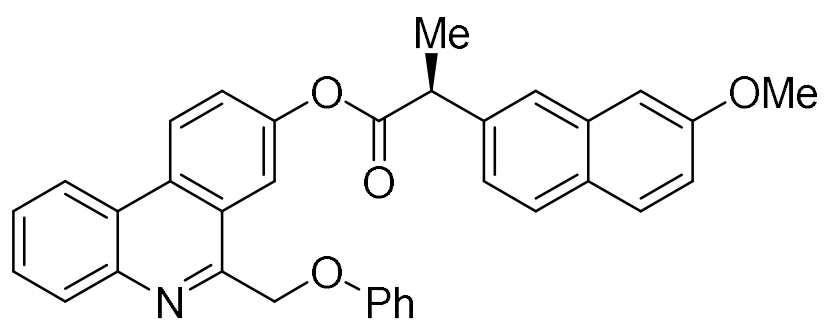
Me
O
O
OMe
N
O
Ph

4ac

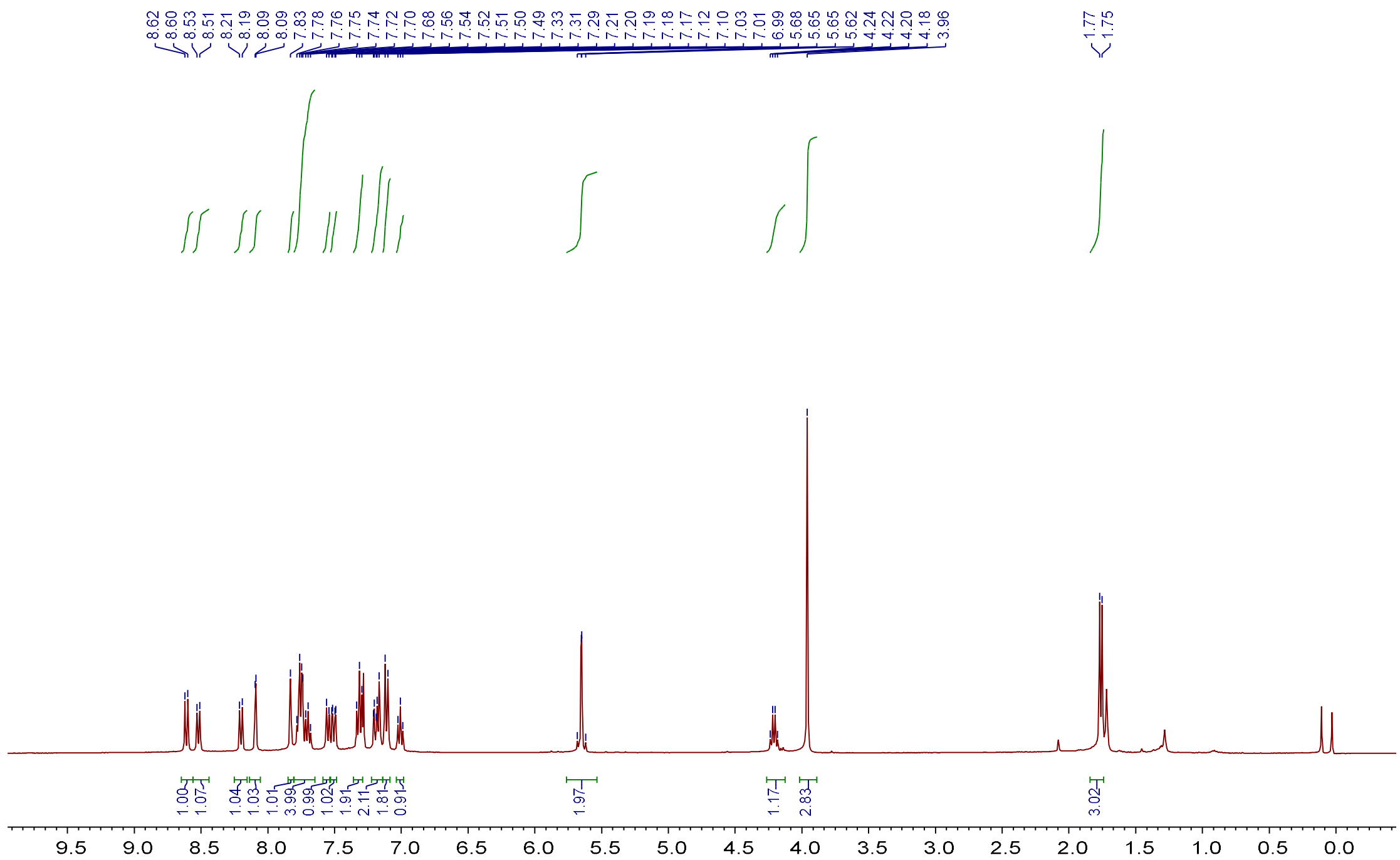
8.62 8.60 8.53 8.51 8.21 8.19 8.09 8.09 7.83 7.78 7.76 7.75 7.74 7.72 7.70 7.68 7.56 7.54 7.52 7.51 7.50 7.49 7.33 7.31 7.29 7.21 7.20 7.19 7.18 7.17 7.12 7.10 7.03 7.01 6.99 5.68 5.65 5.65 5.62 4.24 4.22 4.20 4.18 3.96
1.77 1.75
1.00 1.07 1.04 1.03 1.01 3.99 0.99 1.02 1.91 2.11 1.81 0.91
1.97
1.17 2.83
3.02
9.5 9.0 8.5 8.0 7.5 7.0 6.5 6.0 5.5 5.0 4.5 4.0 3.5 3.0 2.5 2.0 1.5 1.0 0.5 0.0

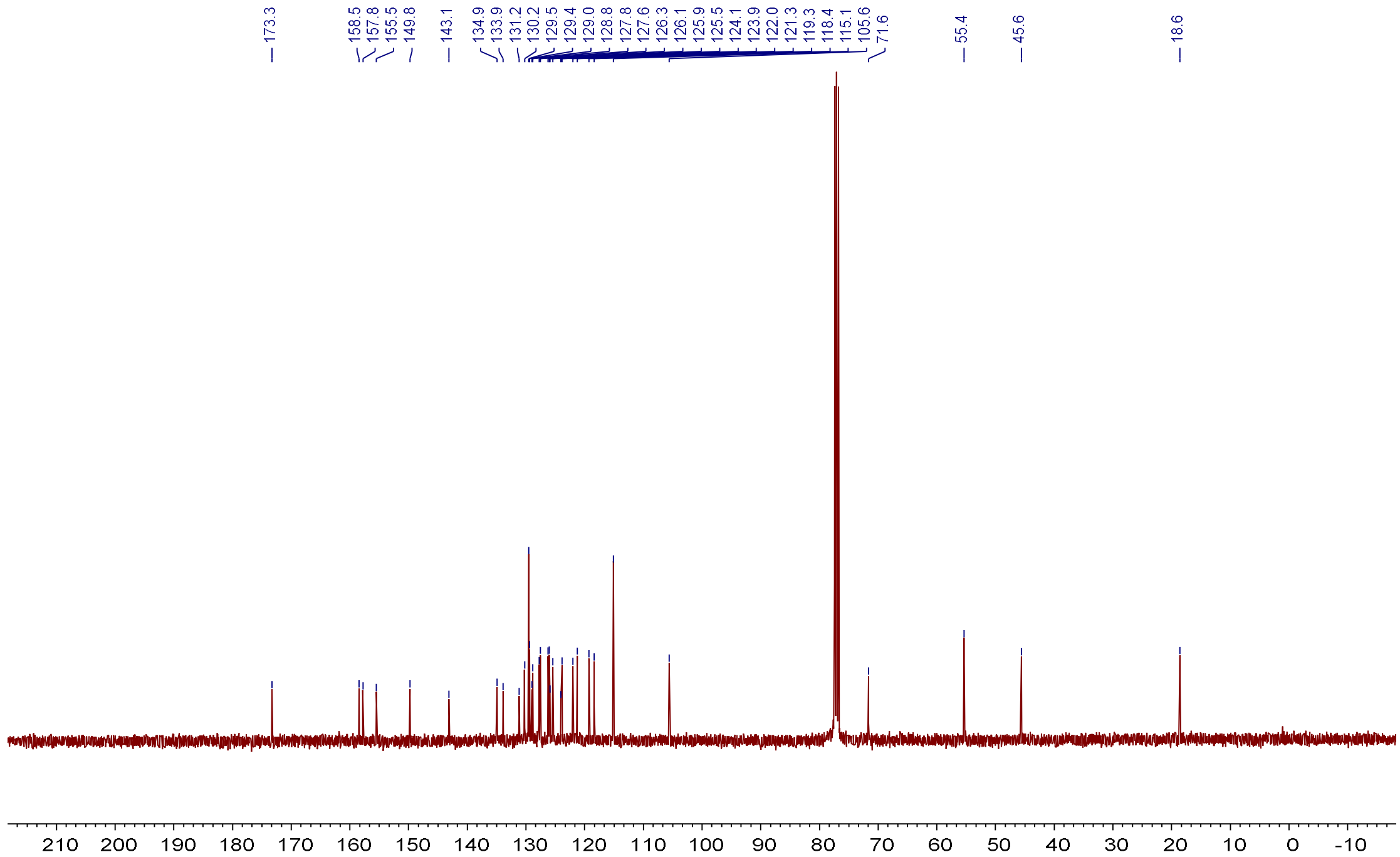
173.3
158.5 157.8 155.5 149.8
143.1
134.9 133.9 131.2 130.2 129.5 129.4 129.0 128.8 127.8 127.6 126.3 126.1 125.9 125.5 124.1 123.9 122.0 121.3 119.3 118.4 115.1 105.6 71.6
55.4
45.6
18.6
210 200 190 180 170 160 150 140 130 120 110 100 90 80 70 60 50 40 30 20 10 0 -10

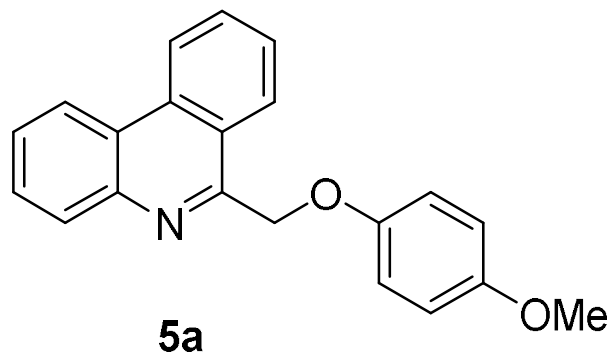
N
O
OMe

5a

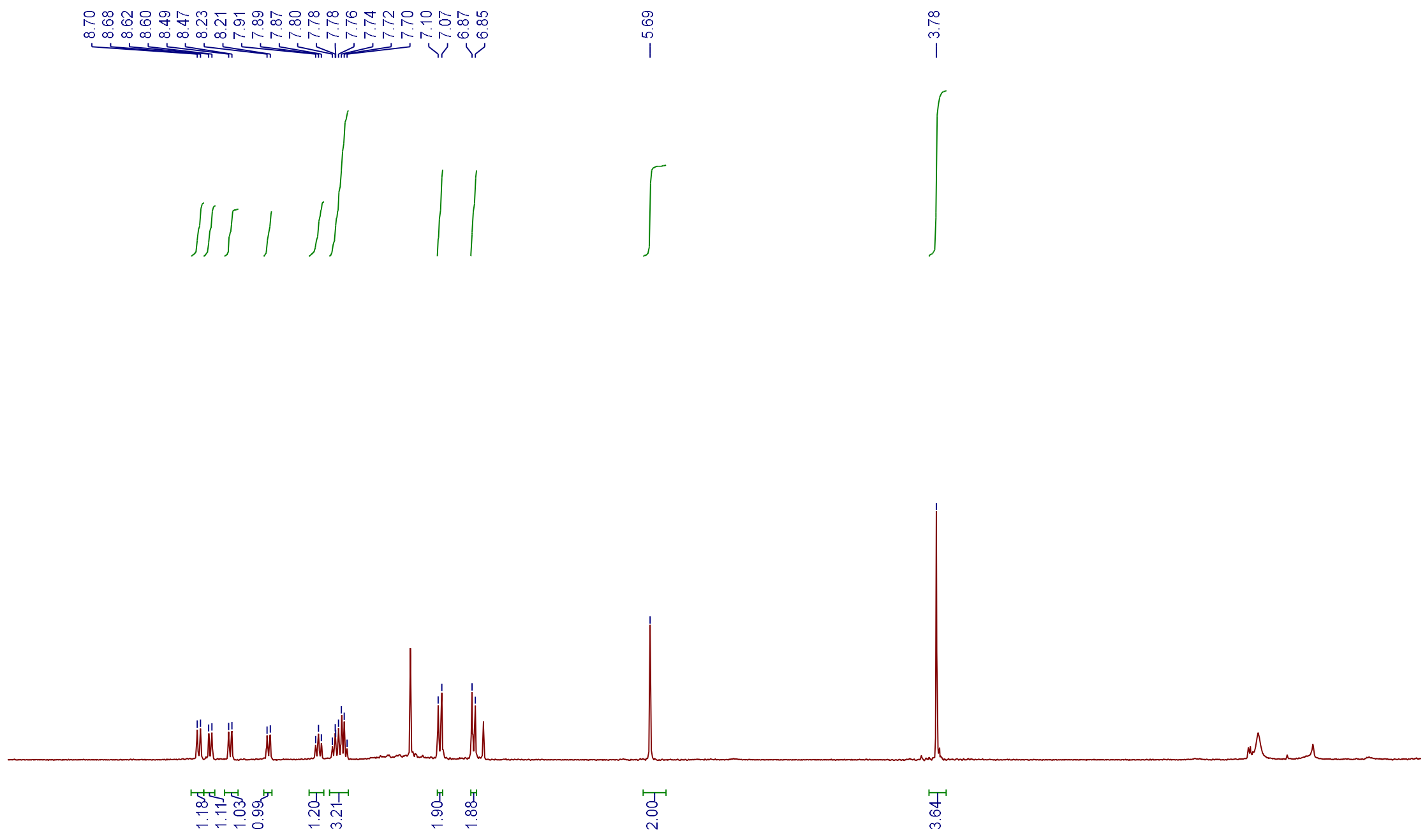
8.70 8.68 8.62 8.60 8.49 8.47 8.23 8.21 7.91 7.89 7.87 7.80 7.78 7.78 7.76 7.74 7.72 7.70 7.10 7.07 6.87 6.85
5.69
3.78
1.18 1.11 1.03 0.99 1.20 3.21 1.90 1.88 2.00 3.64
9.5 9.0 8.5 8.0 7.5 7.0 6.5 6.0 5.5 5.0 4.5 4.0 3.5 3.0 2.5 2.0 1.5 1.0

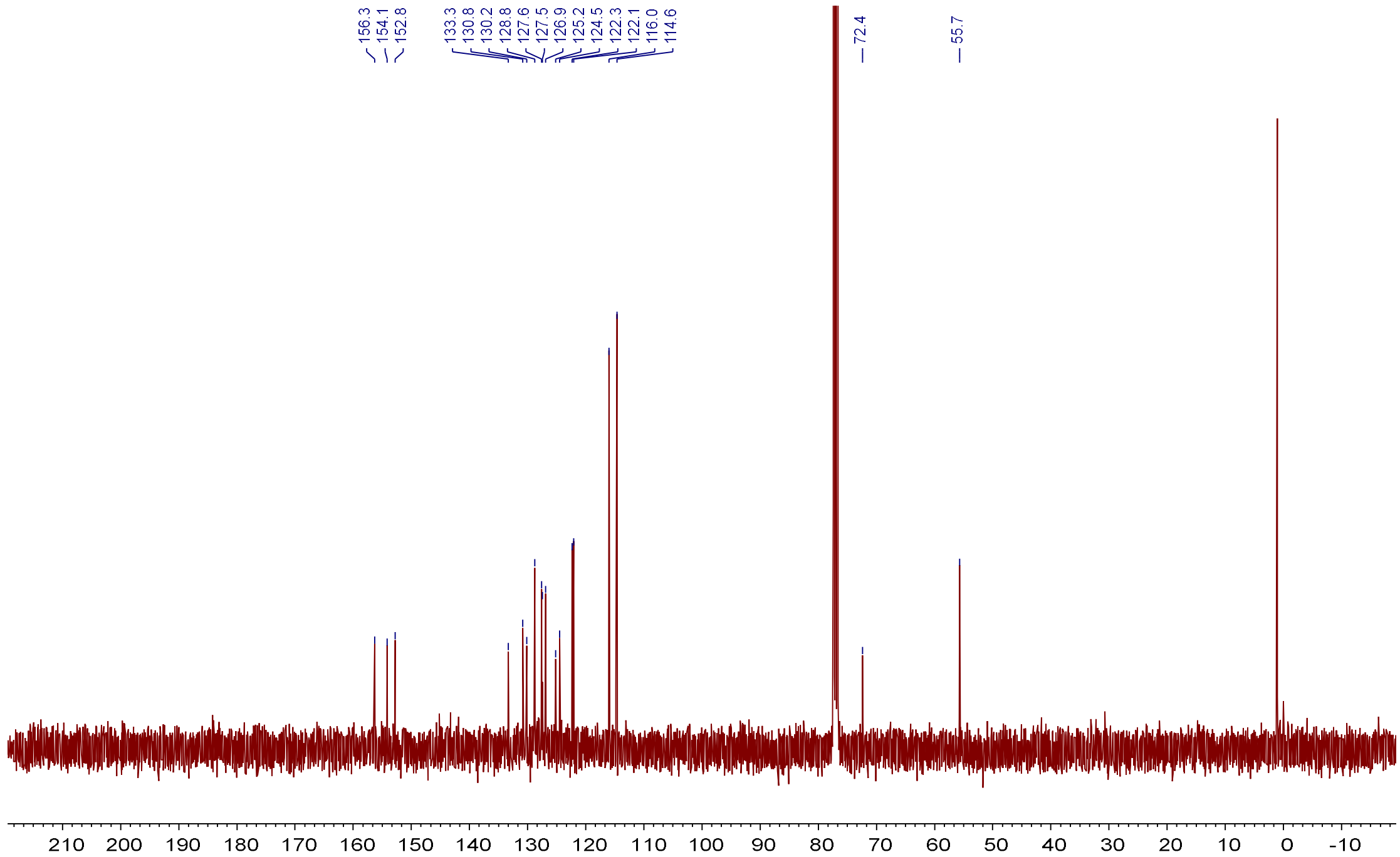
156.3 154.1 152.8
133.3 130.8 130.2 128.8 127.6 127.5 126.9 125.2 124.5 122.3 122.1 116.0 114.6
72.4
55.7
210 200 190 180 170 160 150 140 130 120 110 100 90 80 70 60 50 40 30 20 10 0 -10

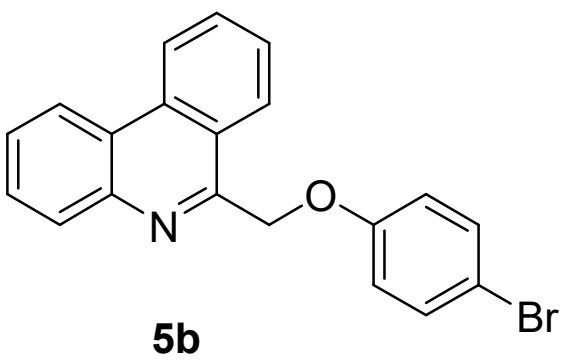
N
O
Br

5b

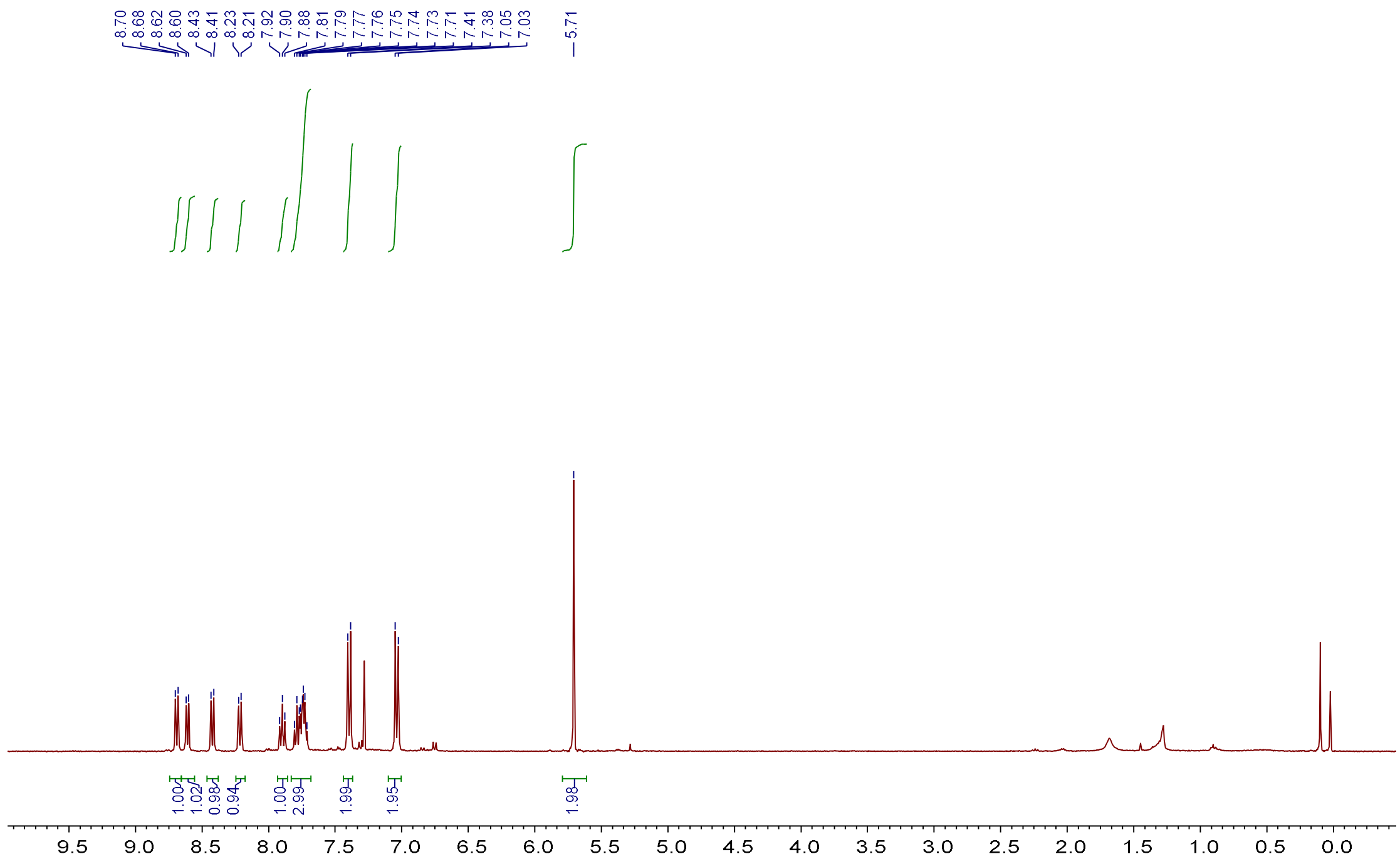
8.70 8.68 8.62 8.60 8.43 8.41 8.23 8.21 7.92 7.90 7.88 7.81 7.79 7.77 7.76 7.75 7.74 7.73 7.71 7.41 7.38 7.05 7.03
5.71
1.00 1.02 0.98 0.94 1.00 2.99 1.99 1.95 1.98
9.5 9.0 8.5 8.0 7.5 7.0 6.5 6.0 5.5 5.0 4.5 4.0 3.5 3.0 2.5 2.0 1.5 1.0 0.5 0.0

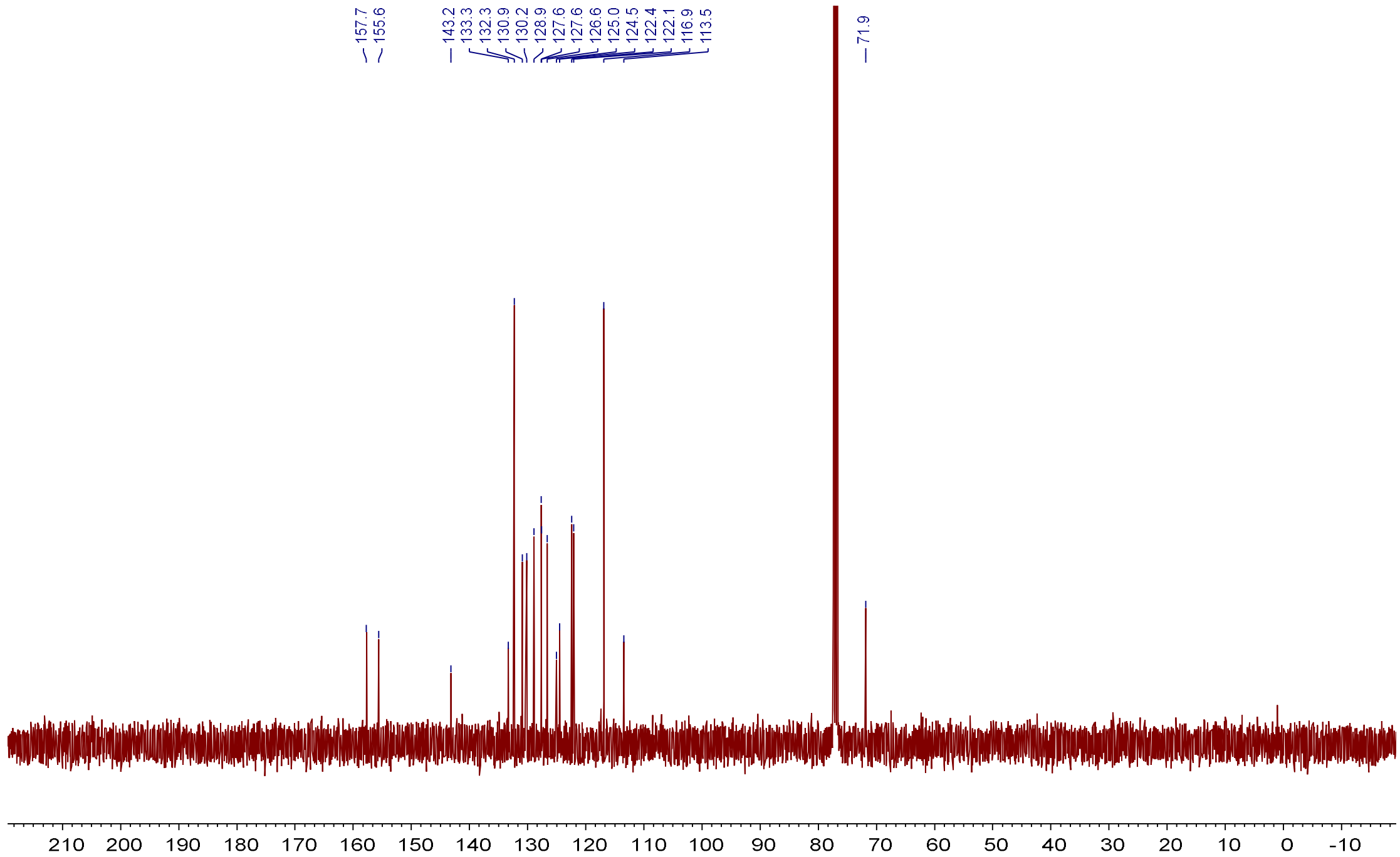
157.7 155.6
143.2 133.3 132.3 130.9 130.2 128.9 127.6 126.6 125.0 124.5 122.4 122.1 116.9 113.5
71.9
210 200 190 180 170 160 150 140 130 120 110 100 90 80 70 60 50 40 30 20 10 0 -10

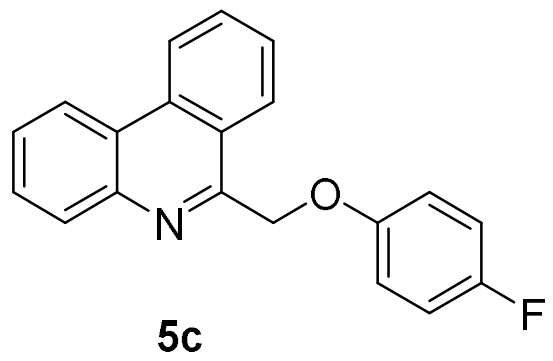
O
N
F

5c

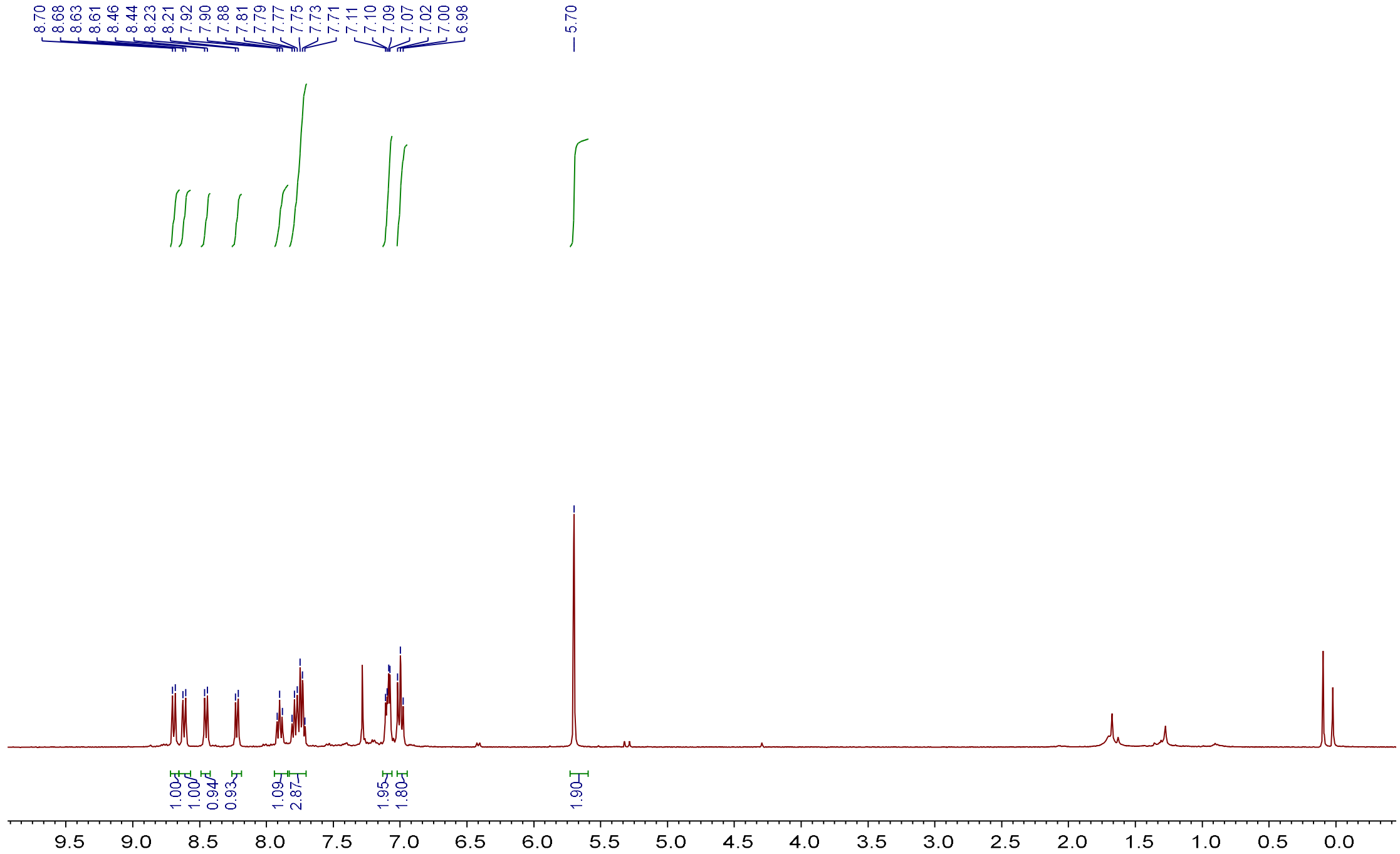
8.70
8.68
8.63
8.61
8.46
8.44
8.23
8.21
7.92
7.90
7.88
7.81
7.79
7.77
7.75
7.73
7.71
7.11
7.10
7.09
7.07
7.02
7.00
6.98
5.70
1.00
1.00
0.94
0.93
1.09
2.87
1.95
1.80
1.90
9.5 9.0 8.5 8.0 7.5 7.0 6.5 6.0 5.5 5.0 4.5 4.0 3.5 3.0 2.5 2.0 1.5 1.0 0.5 0.0

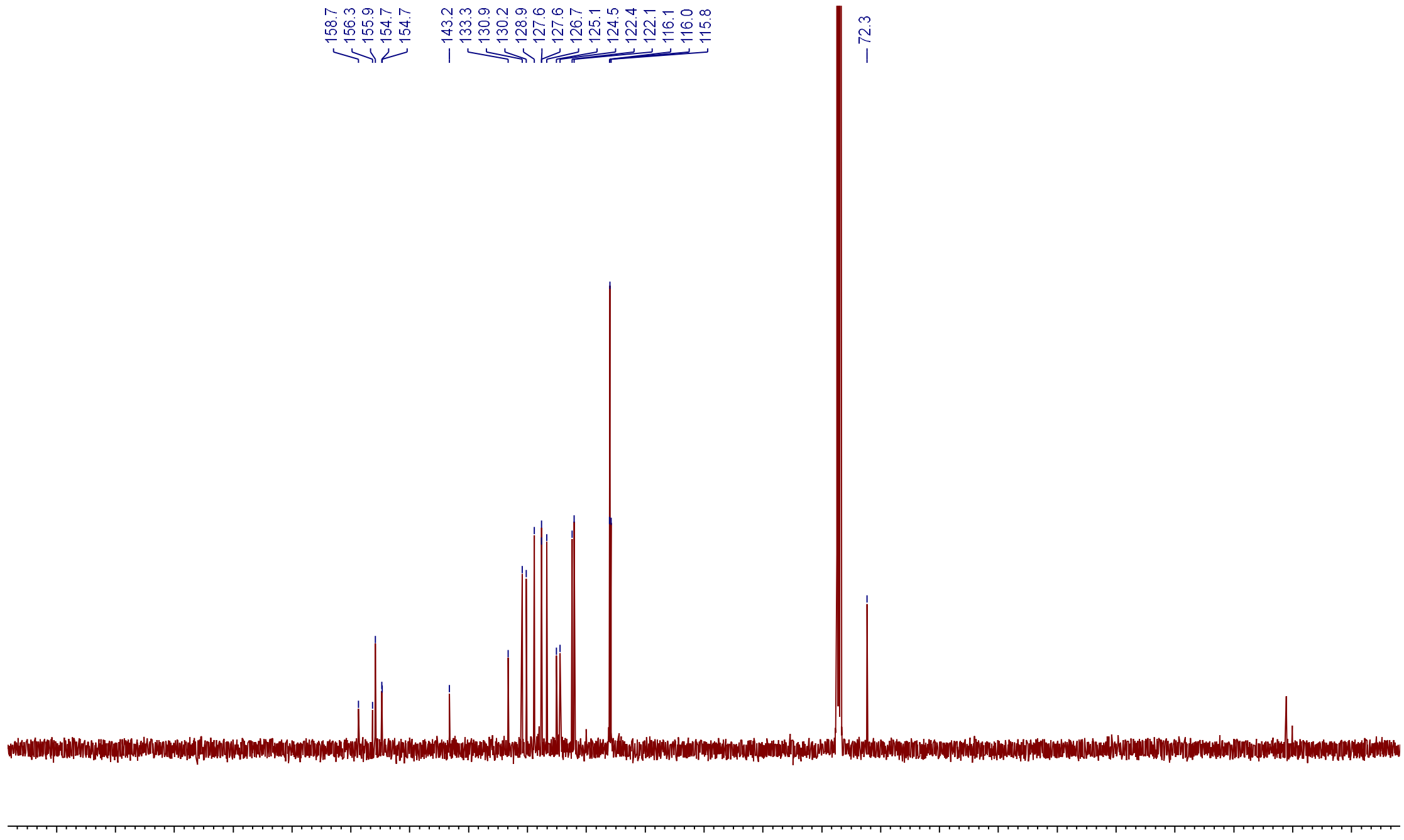
158.7
156.3
155.9
154.7
154.7
143.2
133.3
130.9
130.2
128.9
127.6
126.7
125.1
124.5
122.4
122.1
116.1
116.0
115.8
72.3
210 200 190 180 170 160 150 140 130 120 110 100 90 80 70 60 50 40 30 20 10 0 -10

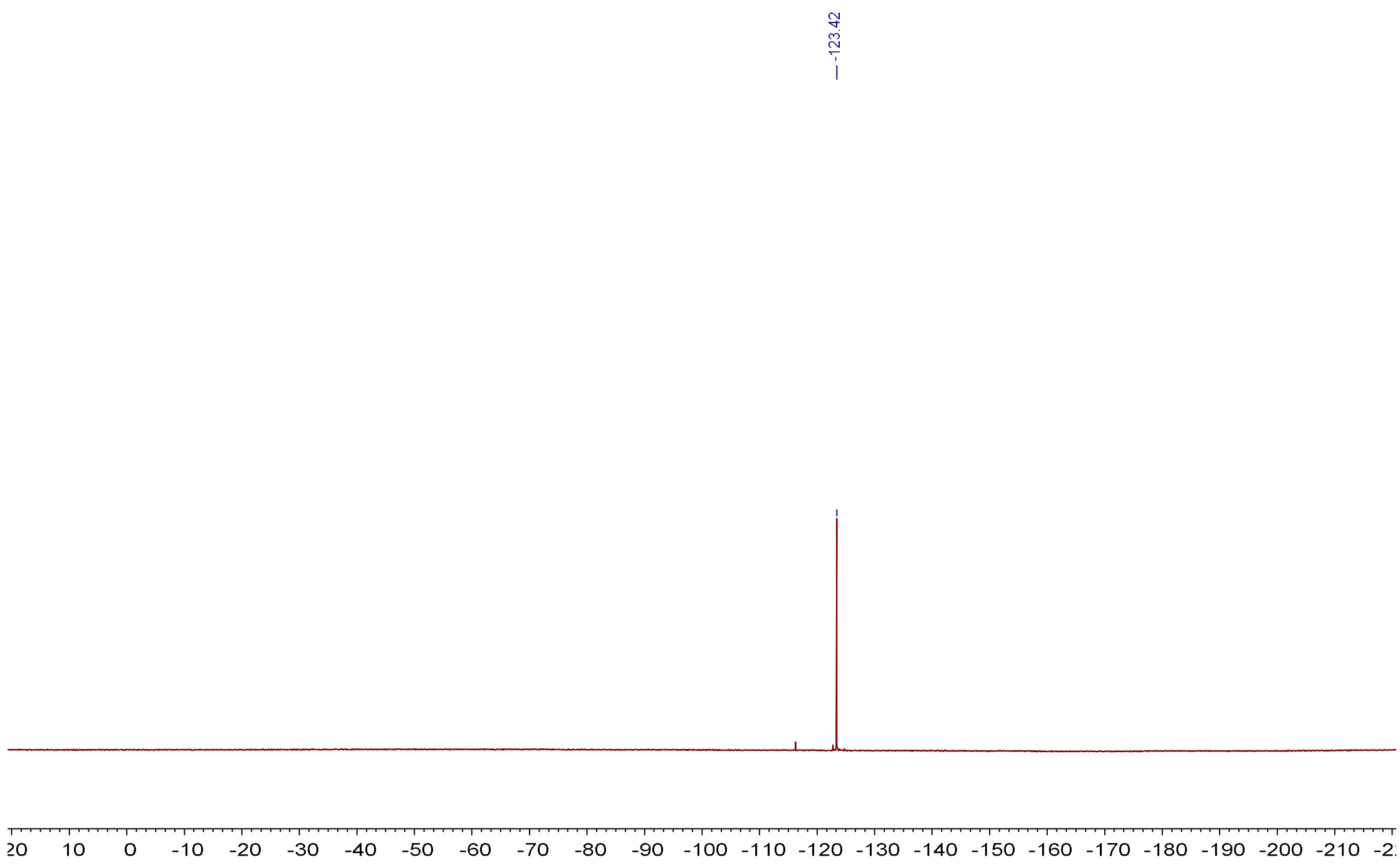
-123.42
20 10 0 -10 -20 -30 -40 -50 -60 -70 -80 -90 -100 -110 -120 -130 -140 -150 -160 -170 -180 -190 -200 -210

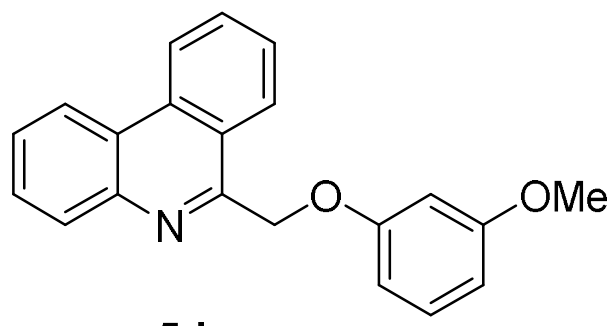

OMe
N
O

5d

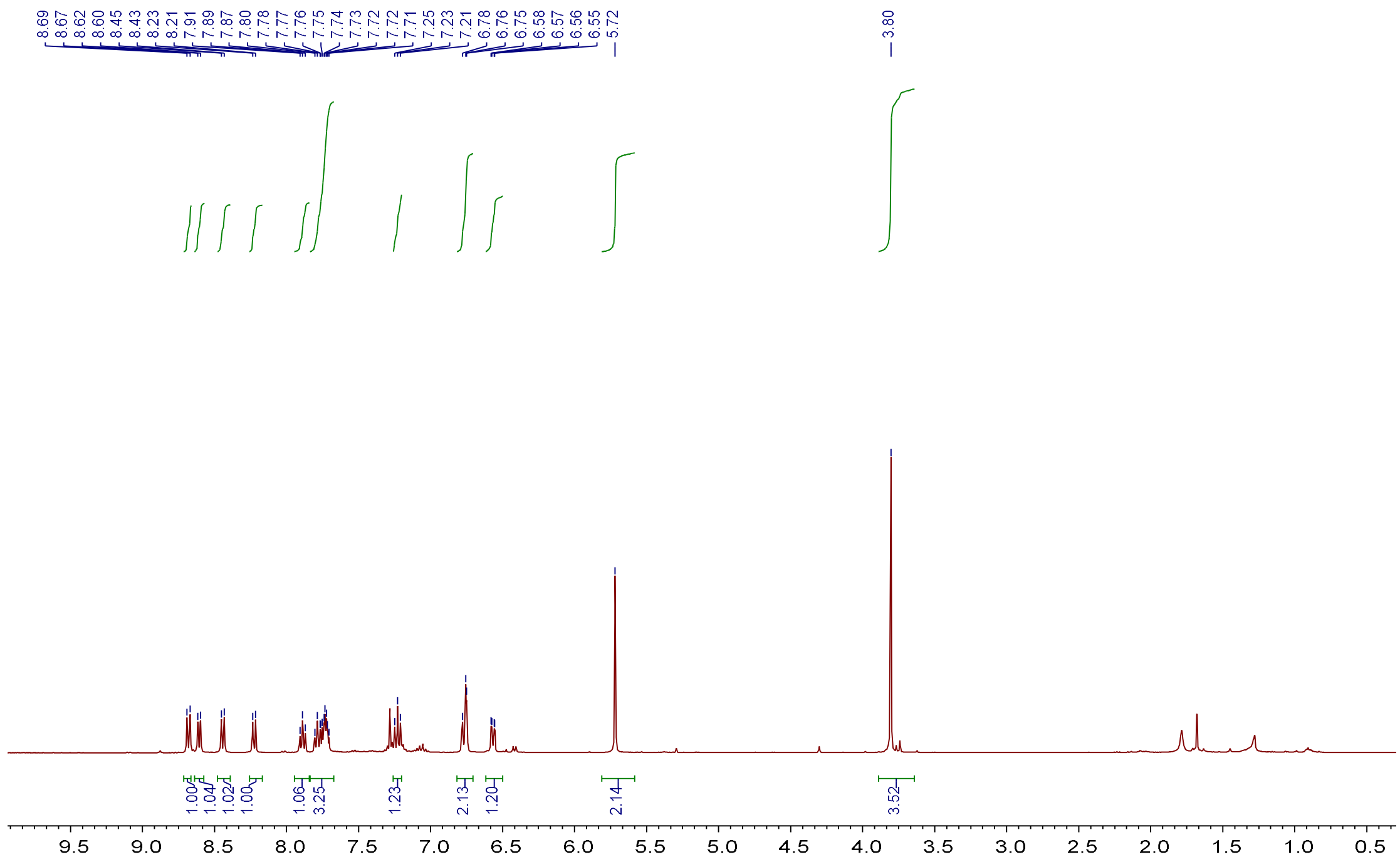

8.69
8.67
8.62
8.60
8.45
8.43
8.23
8.21
7.91
7.89
7.87
7.80
7.78
7.77
7.76
7.75
7.74
7.73
7.72
7.72
7.71
7.25
7.23
7.21
6.78
6.76
6.75
6.58
6.57
6.56
6.55
5.72
3.80
1.00
1.04
1.02
1.00
1.06
3.25
1.23
2.13
1.20
2.14
3.52
9.5 9.0 8.5 8.0 7.5 7.0 6.5 6.0 5.5 5.0 4.5 4.0 3.5 3.0 2.5 2.0 1.5 1.0 0.5

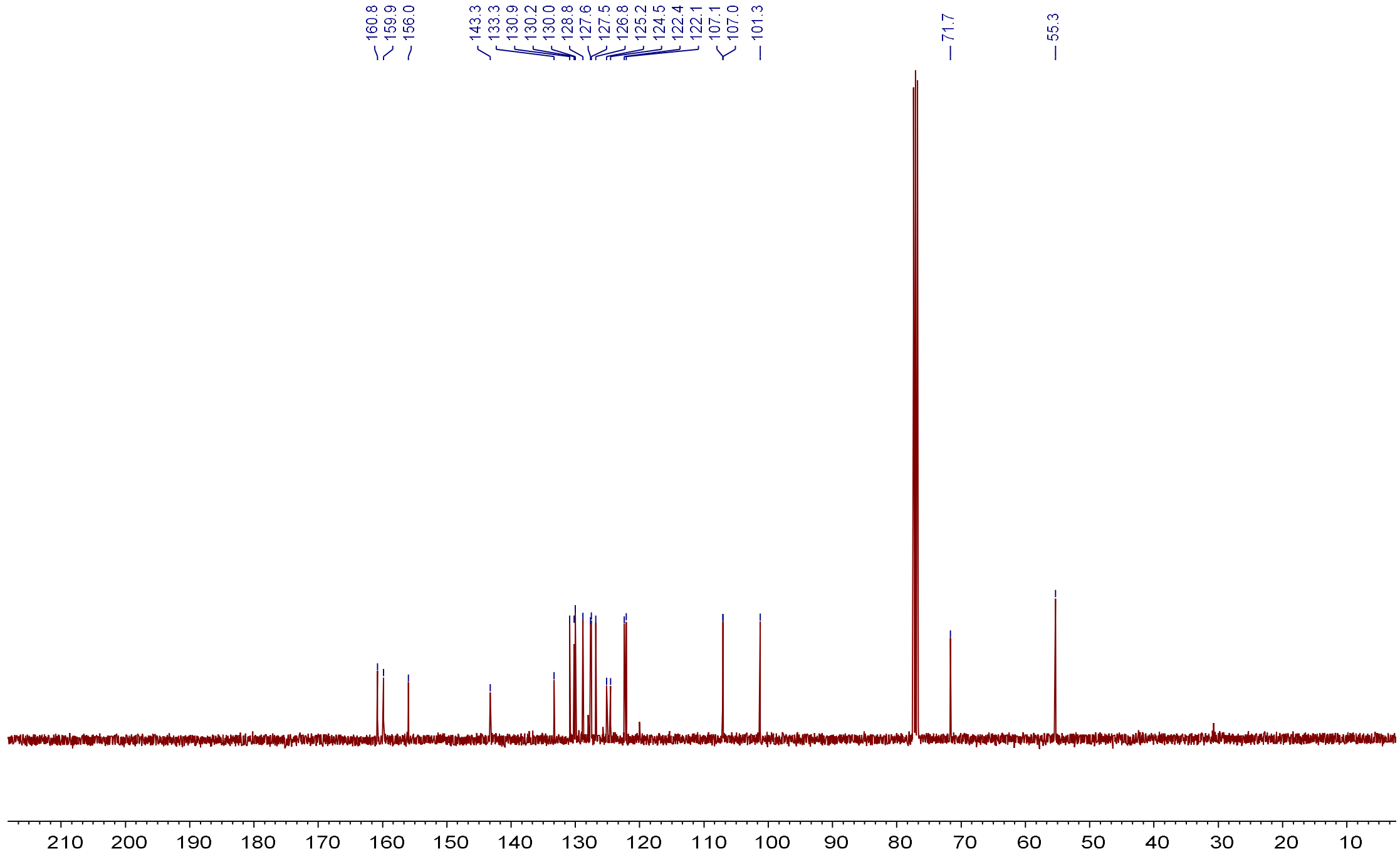

160.8
159.9
156.0
143.3
133.3
130.9
130.2
130.0
128.8
127.6
127.5
126.8
125.2
124.5
122.4
122.1
107.1
107.0
101.3
71.7
55.3
210 200 190 180 170 160 150 140 130 120 110 100 90 80 70 60 50 40 30 20 10

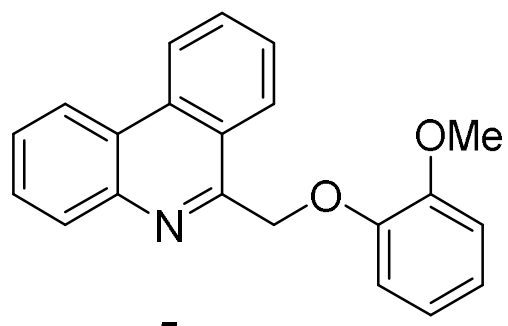
OMe
N
O

5e

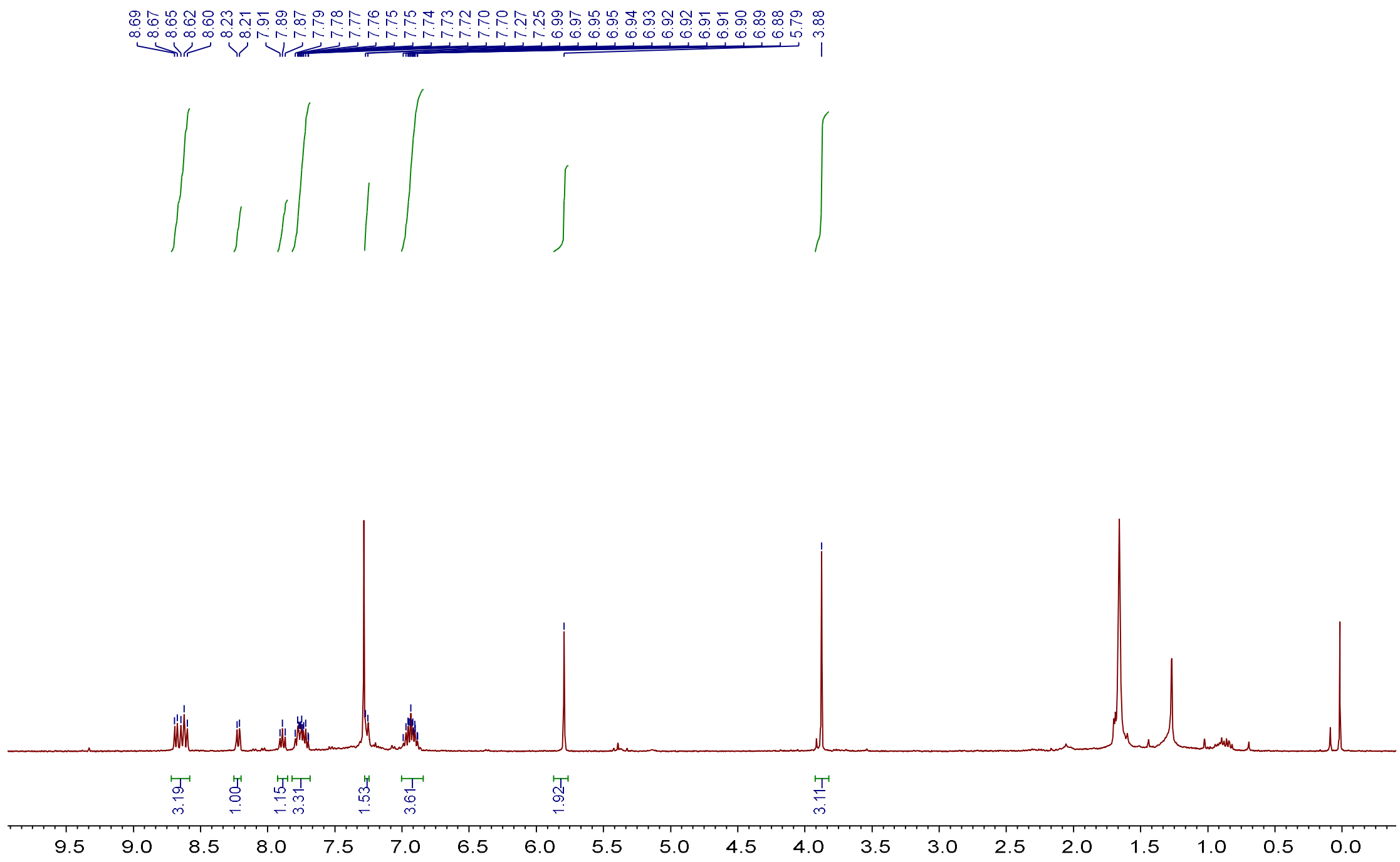
8.69
8.67
8.65
8.62
8.60
8.23
8.21
7.91
7.89
7.87
7.79
7.78
7.77
7.76
7.75
7.75
7.74
7.73
7.72
7.70
7.70
7.27
7.25
6.99
6.97
6.95
6.95
6.94
6.93
6.93
6.92
6.92
6.91
6.91
6.90
6.89
6.88
5.79
3.88
3.19
1.00
1.15
3.31
1.53
3.61
1.92
3.11
9.5
9.0
8.5
8.0
7.5
7.0
6.5
6.0
5.5
5.0
4.5
4.0
3.5
3.0
2.5
2.0
1.5
1.0
0.5
0.0

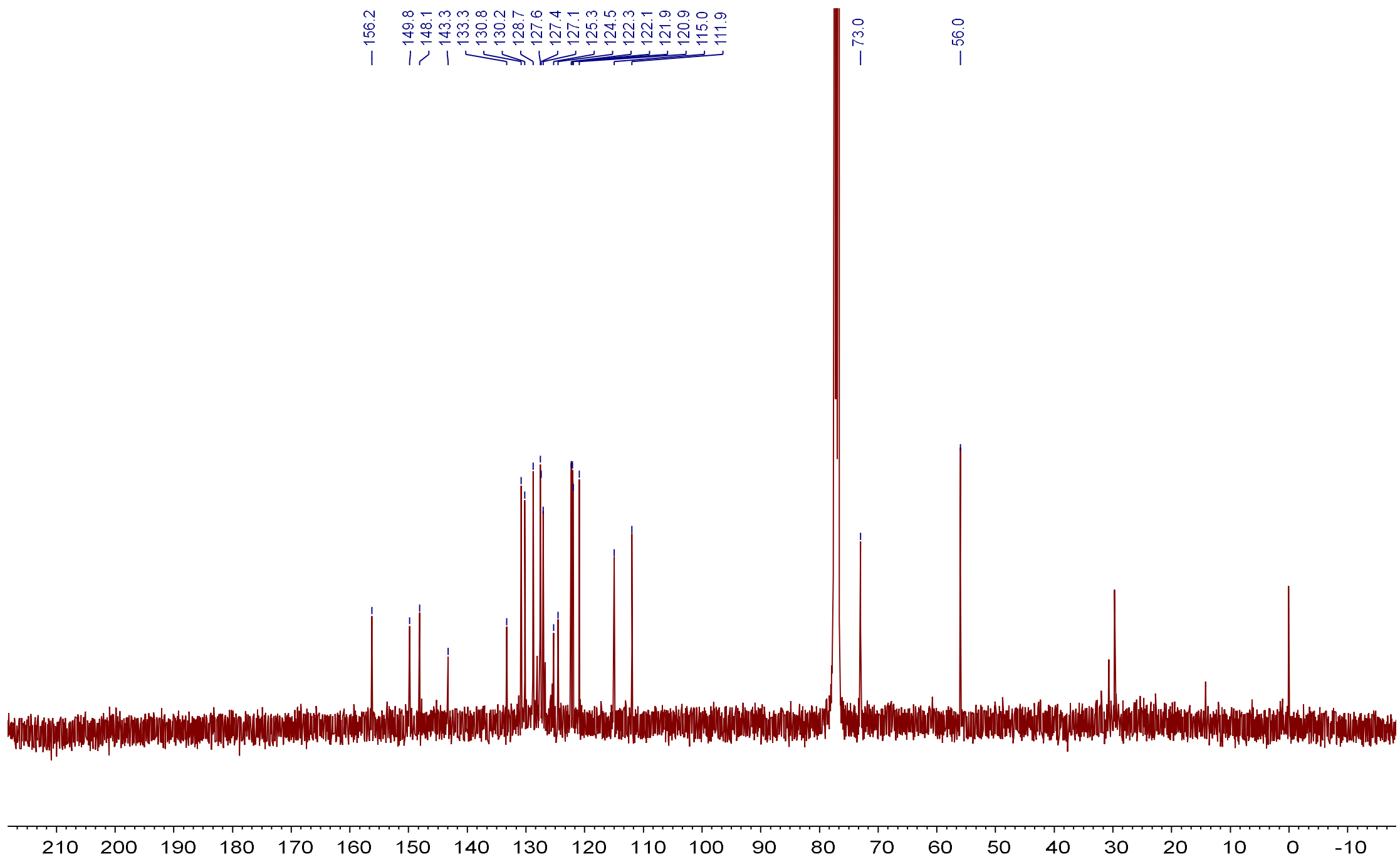
156.2
149.8
148.1
143.3
133.3
130.8
130.2
128.7
127.6
127.4
127.1
125.3
124.5
122.3
122.1
121.9
120.9
115.0
111.9
73.0
56.0
210
200
190
180
170
160
150
140
130
120
110
100
90
80
70
60
50
40
30
20
10
0
-10

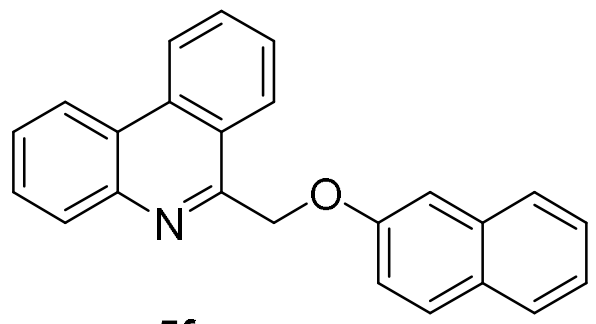

5f

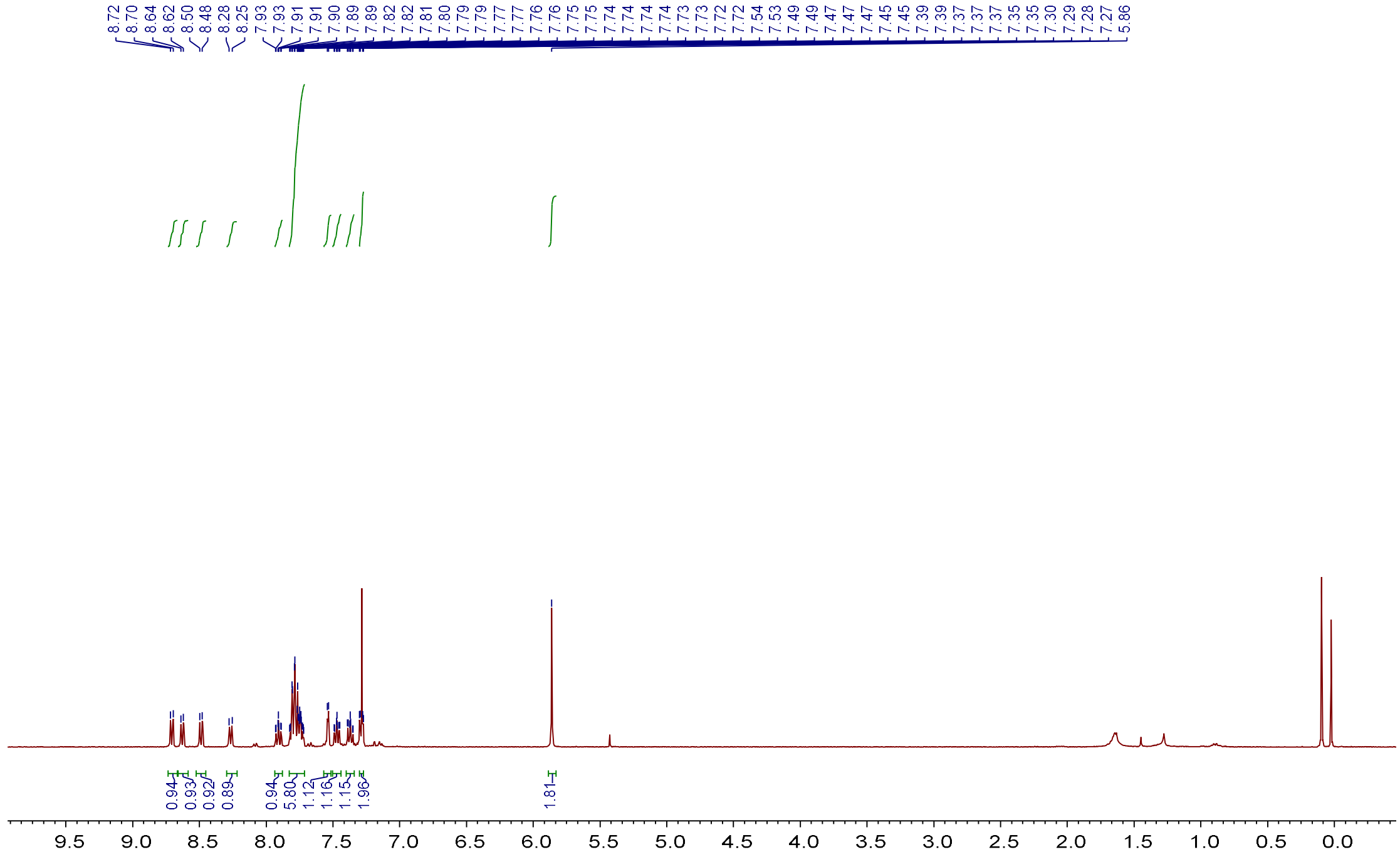

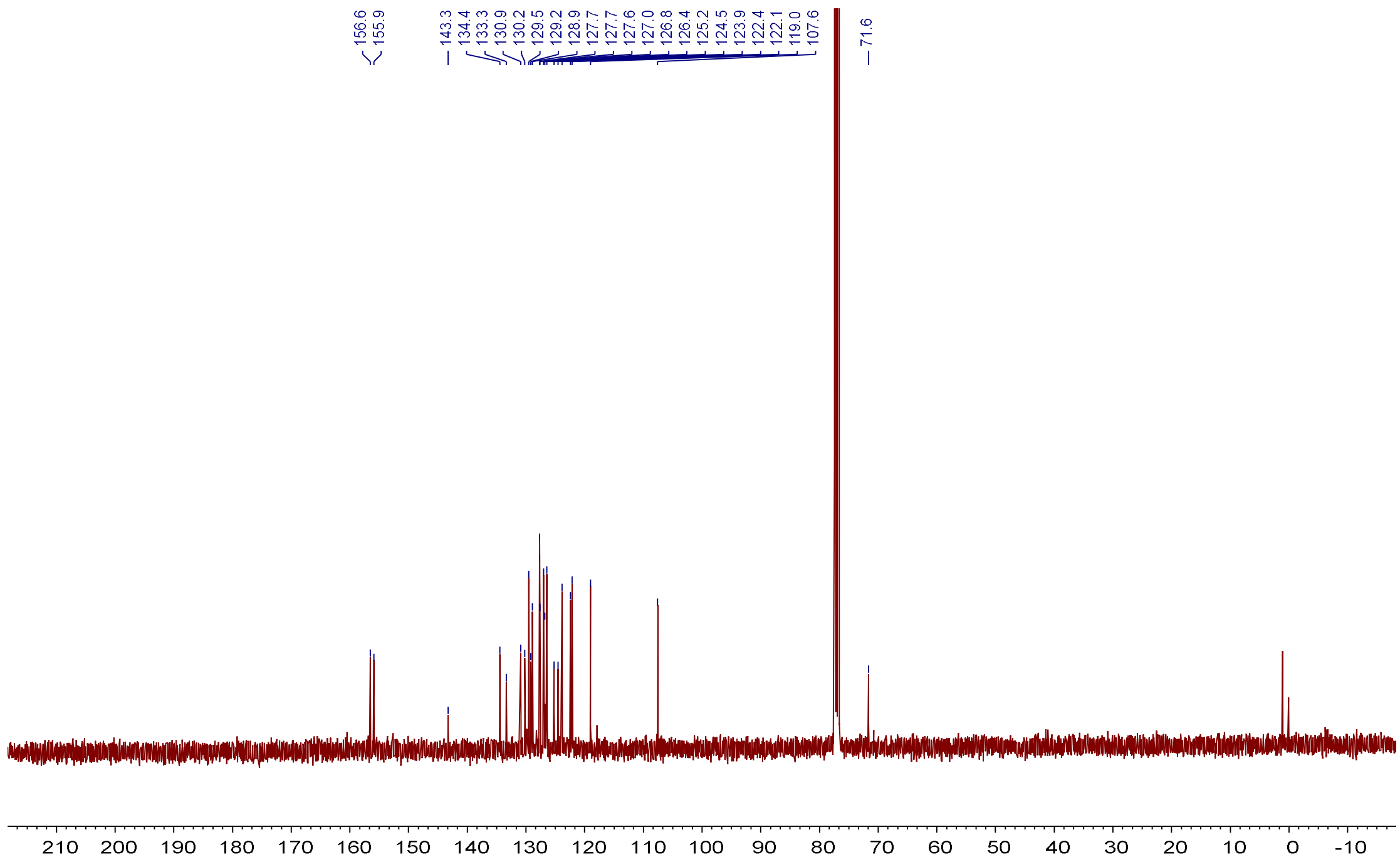

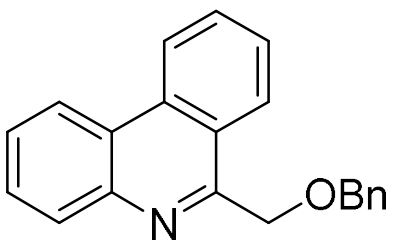

5g

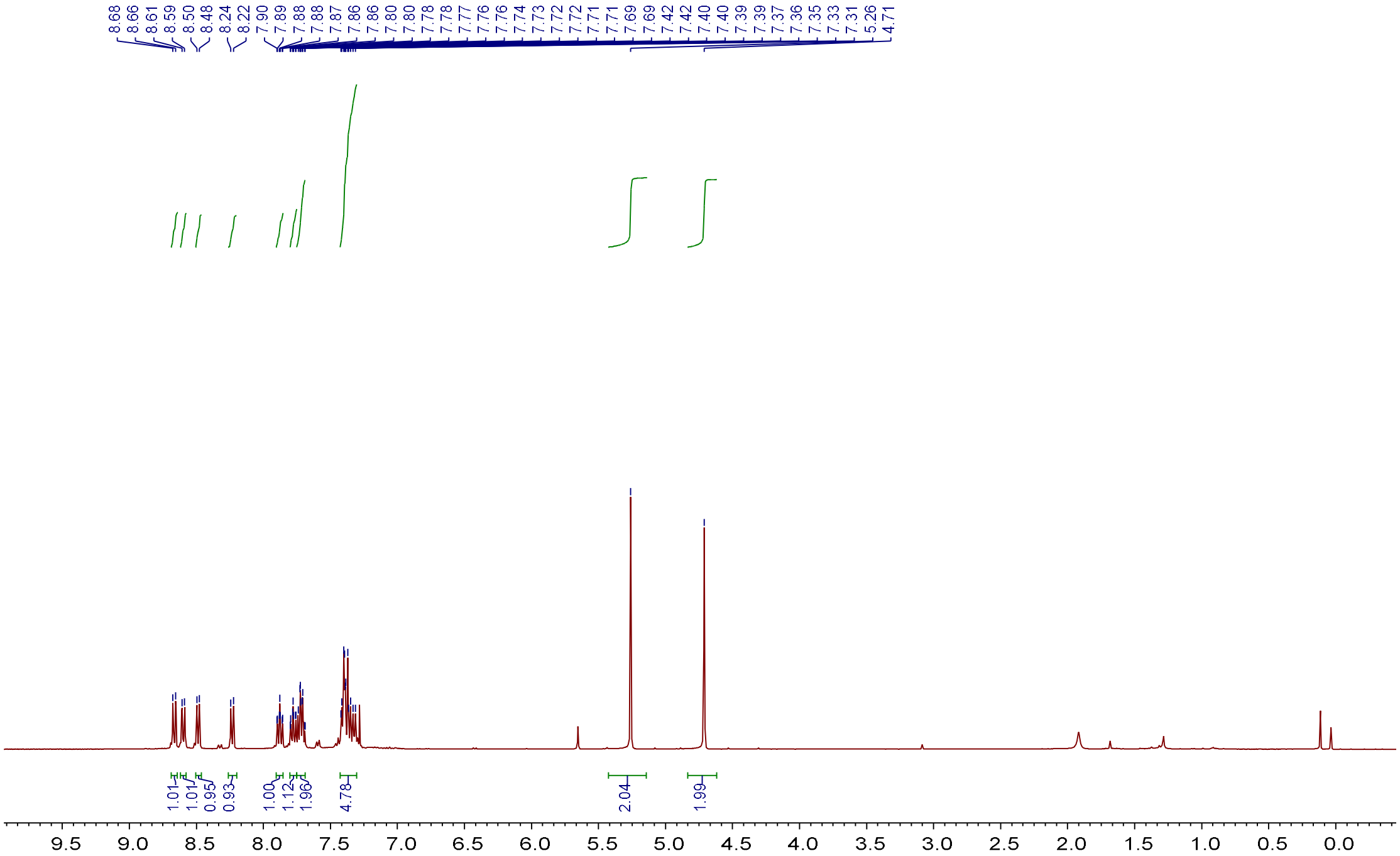

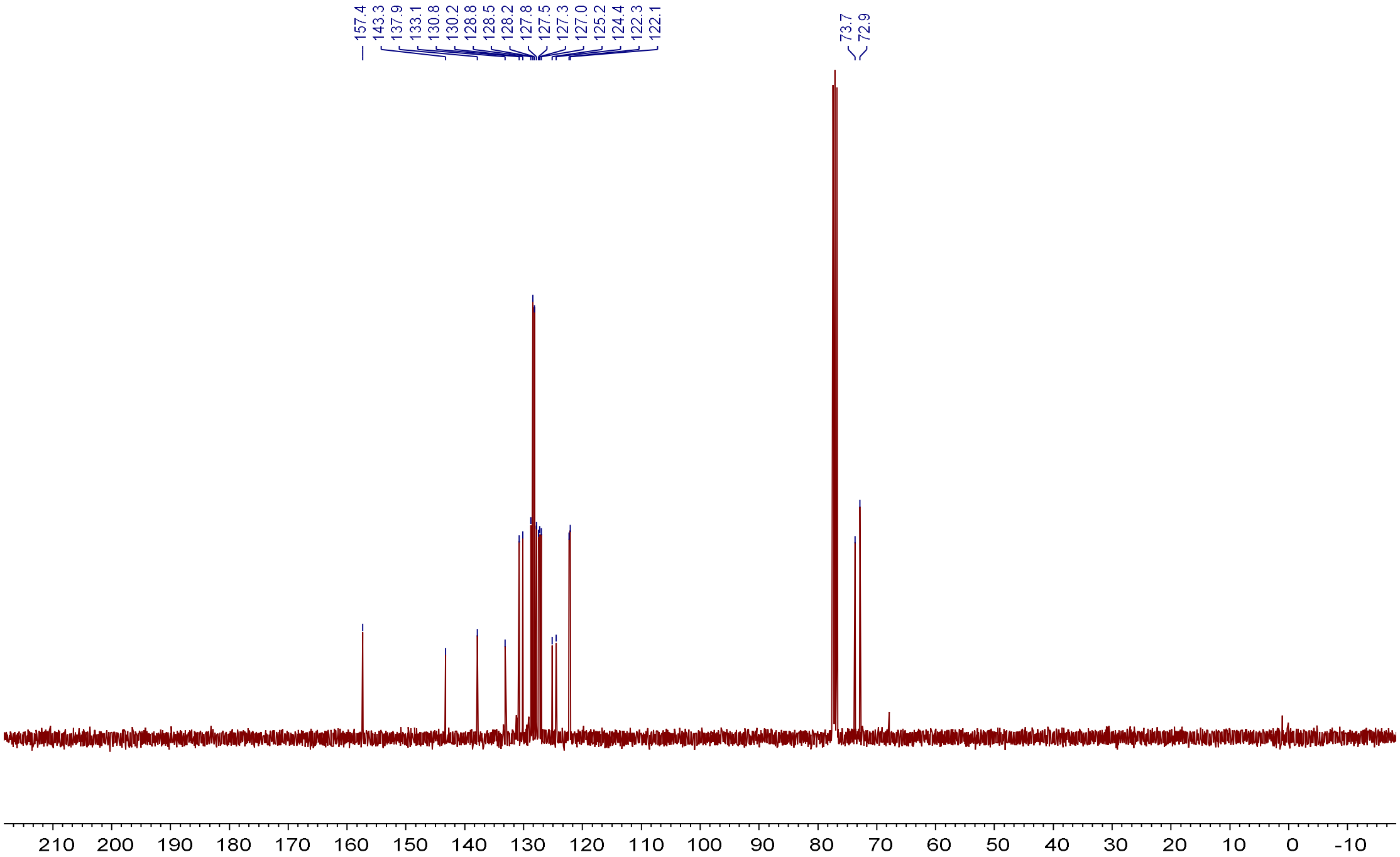

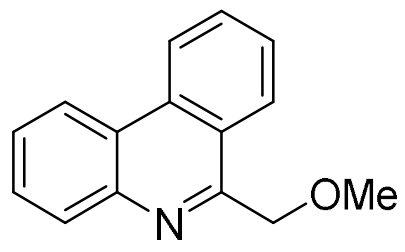
N
OMe

5h

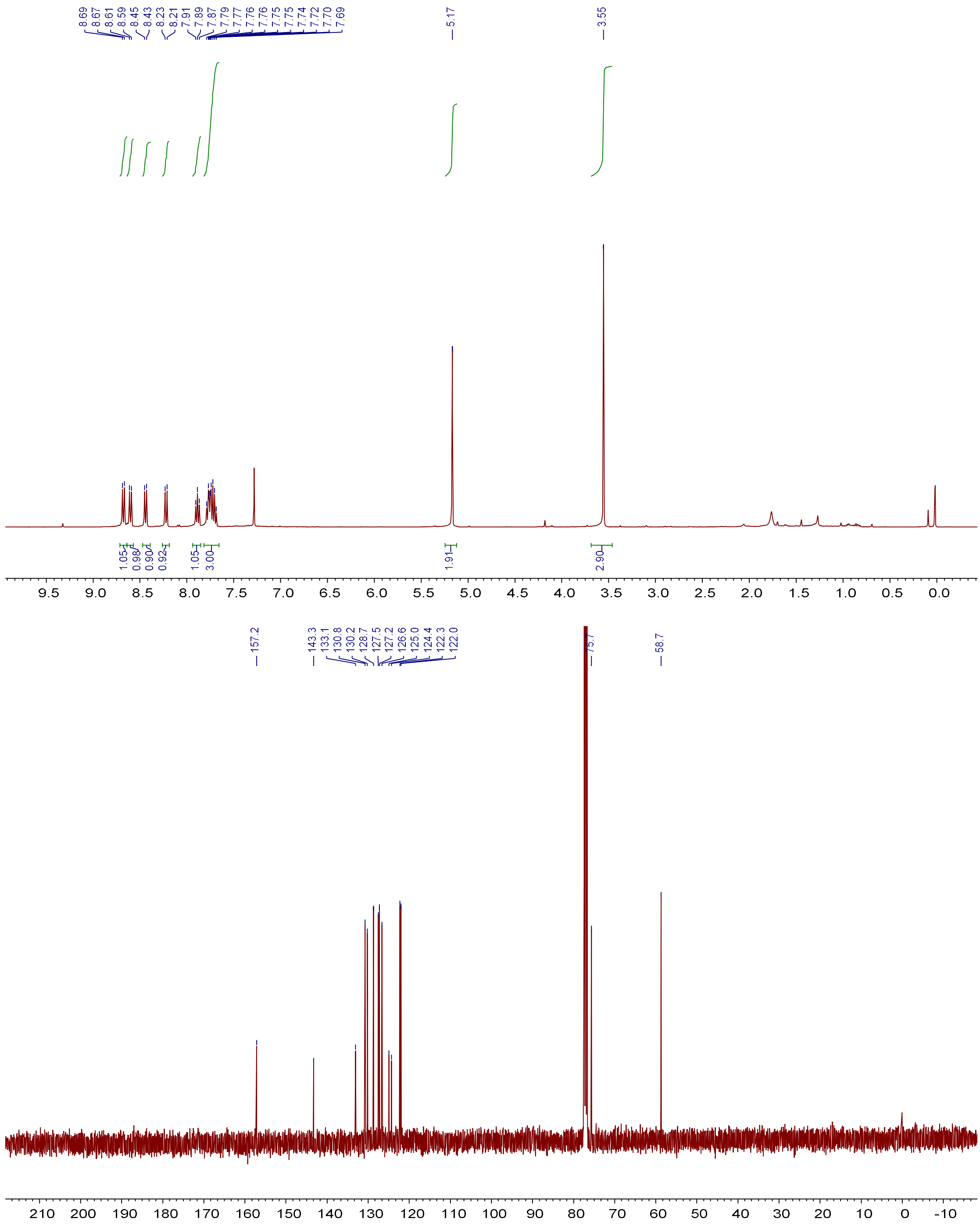
8.69 8.67 8.61 8.59 8.45 8.43 8.23 8.21 7.91 7.89 7.87 7.79 7.77 7.76 7.76 7.75 7.75 7.74 7.72 7.70 7.69
5.17
3.55
1.05 0.98 0.90 0.92 1.05 3.00
1.91
2.90
9.5 9.0 8.5 8.0 7.5 7.0 6.5 6.0 5.5 5.0 4.5 4.0 3.5 3.0 2.5 2.0 1.5 1.0 0.5 0.0
157.2
143.3 133.1 130.8 130.2 128.7 127.5 127.2 126.6 125.0 124.4 122.3 122.0
75.7
58.7
210 200 190 180 170 160 150 140 130 120 110 100 90 80 70 60 50 40 30 20 10 0 -10

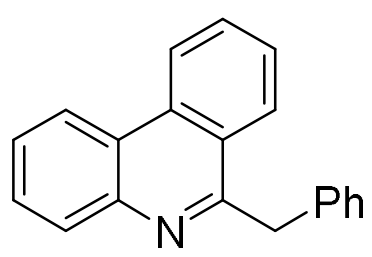

N
Ph

5i

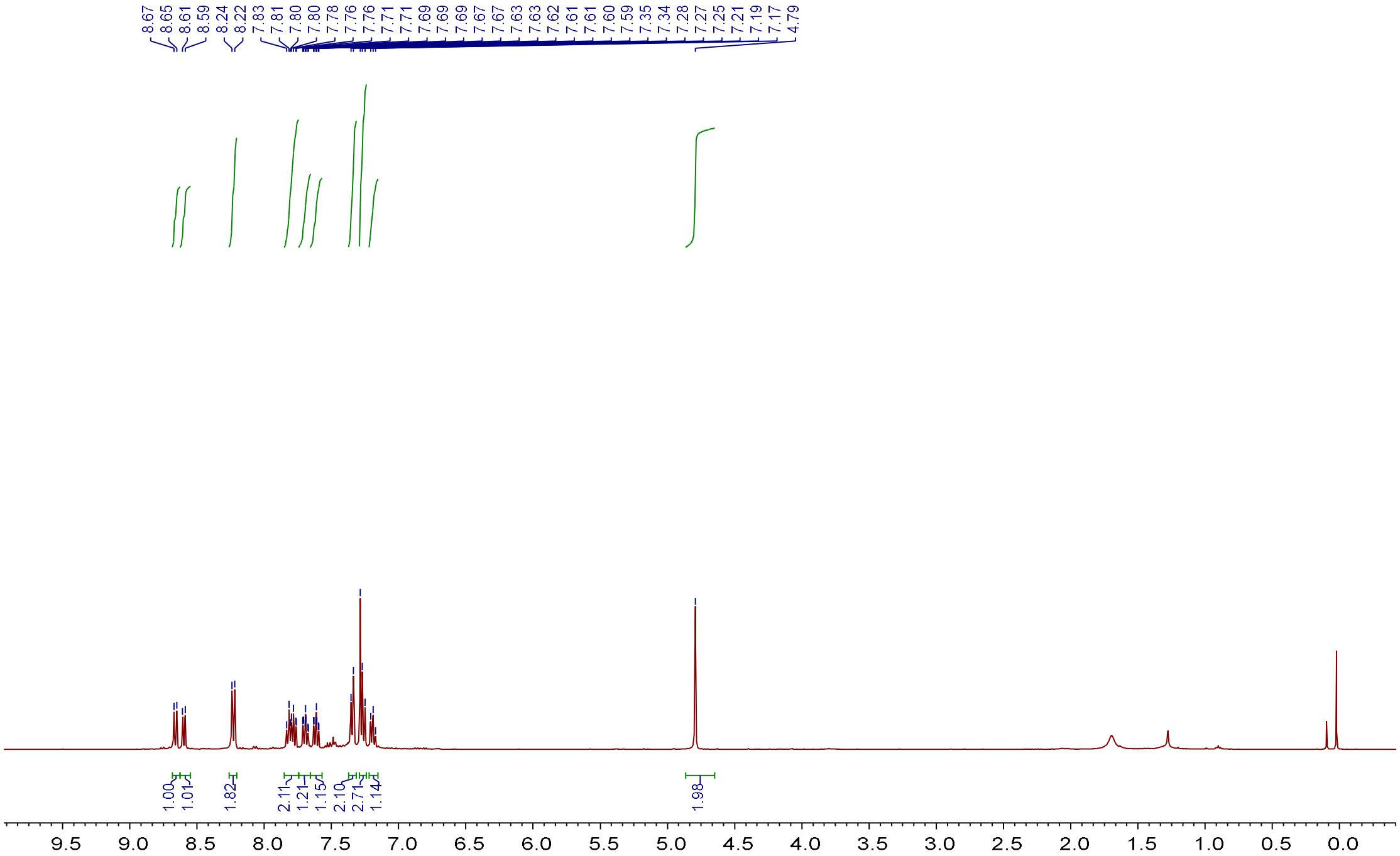

8.67
8.65
8.61
8.59
8.24
8.22
7.83
7.81
7.80
7.80
7.78
7.76
7.76
7.71
7.71
7.69
7.69
7.69
7.67
7.67
7.63
7.63
7.62
7.61
7.61
7.60
7.59
7.35
7.34
7.28
7.27
7.25
7.21
7.19
7.17
4.79
1.00
1.01
1.82
2.11
1.21
1.15
2.10
2.71
1.14
1.98
9.5 9.0 8.5 8.0 7.5 7.0 6.5 6.0 5.5 5.0 4.5 4.0 3.5 3.0 2.5 2.0 1.5 1.0 0.5 0.0

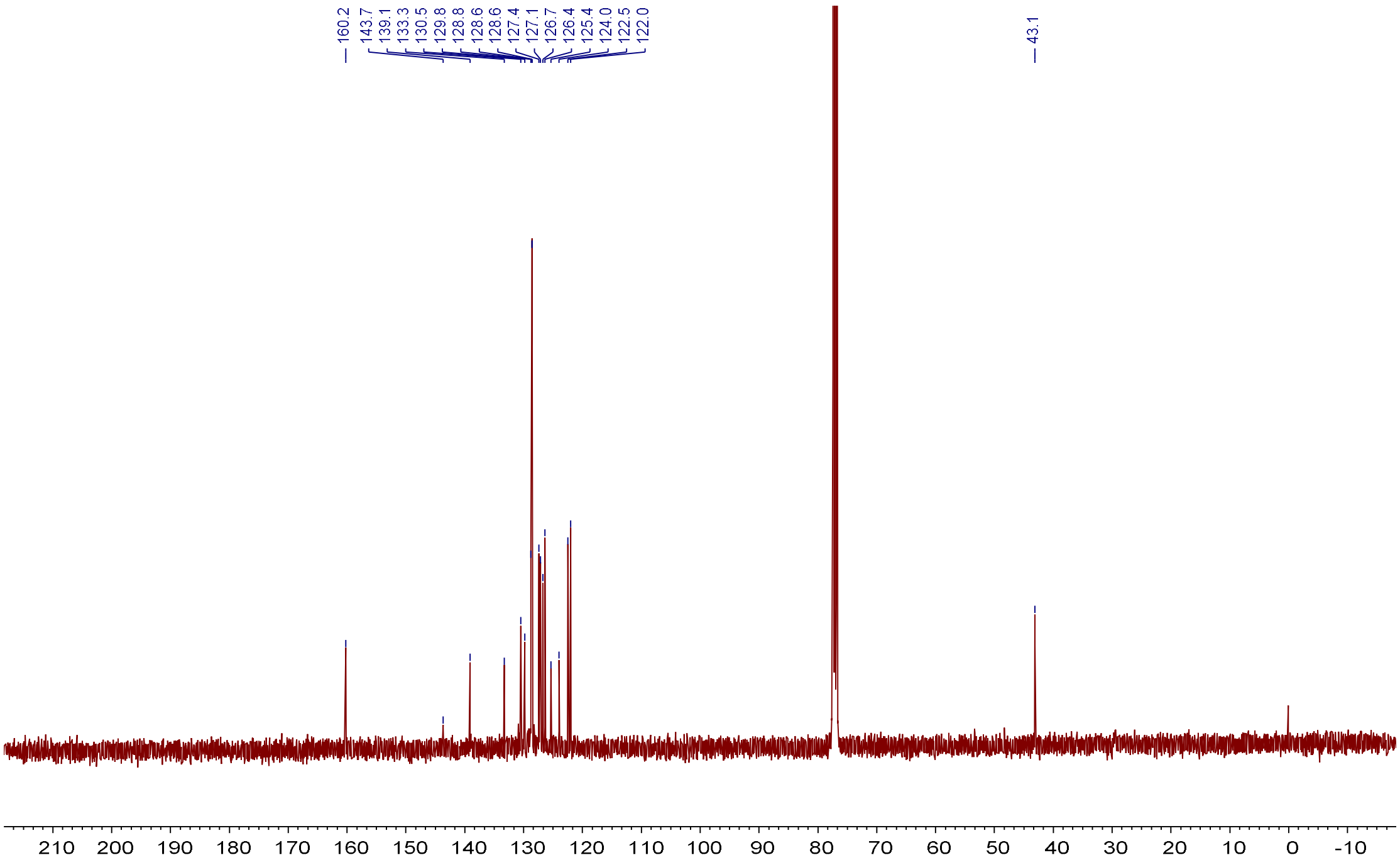

160.2
143.7
139.1
133.3
130.5
129.8
128.8
128.6
128.6
127.4
127.1
126.7
126.4
125.4
124.0
122.5
122.0
43.1
210 200 190 180 170 160 150 140 130 120 110 100 90 80 70 60 50 40 30 20 10 0 -10

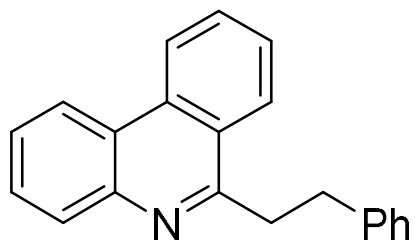
N
Ph

5j

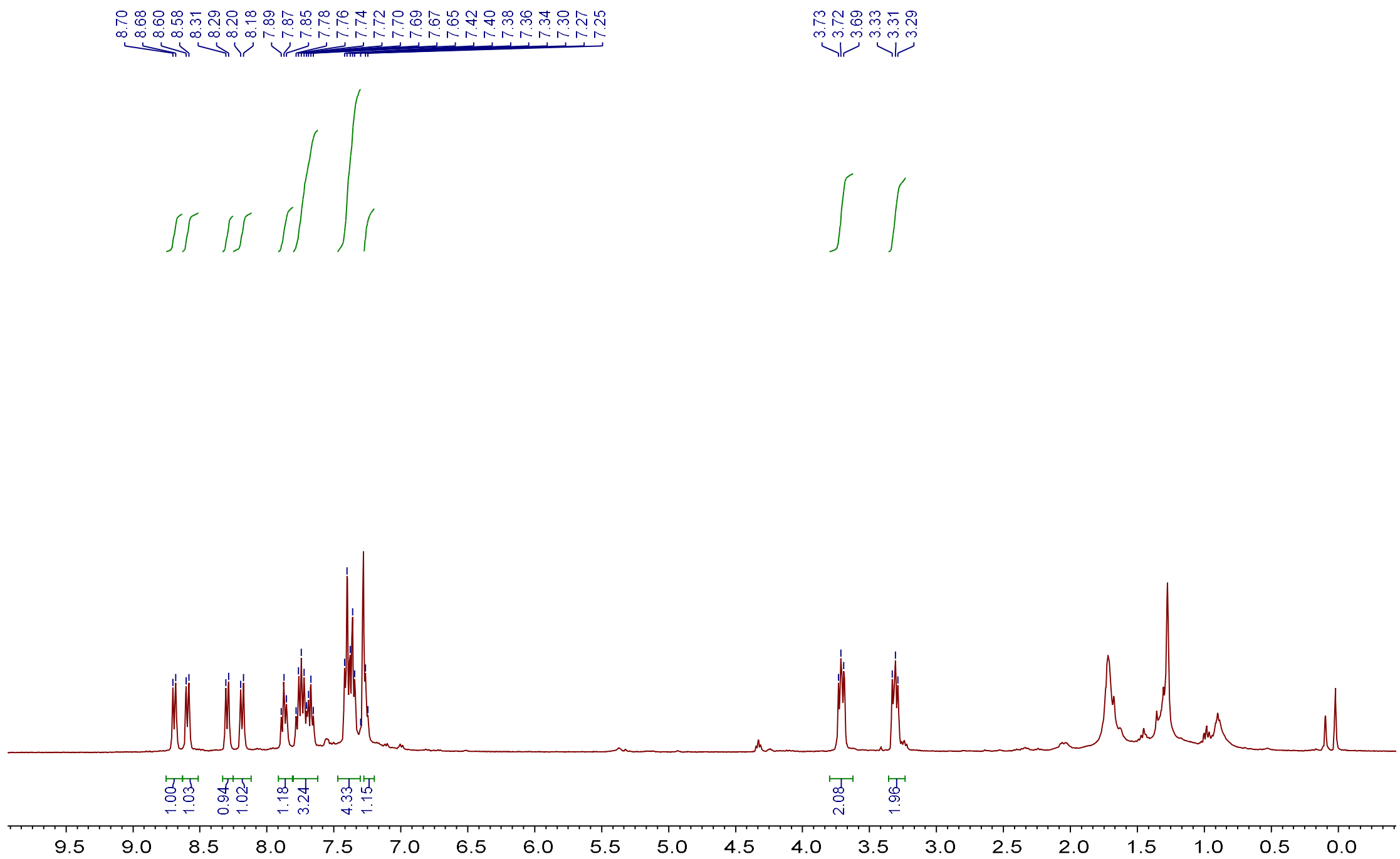
8.70
8.68
8.60
8.58
8.31
8.29
8.20
8.18
7.89
7.87
7.85
7.78
7.76
7.74
7.72
7.70
7.69
7.67
7.65
7.42
7.40
7.38
7.36
7.34
7.30
7.27
7.25
3.73
3.72
3.69
3.33
3.31
3.29
1.00
1.03
0.94
1.02
1.18
3.24
4.33
1.15
2.08
1.96
9.5 9.0 8.5 8.0 7.5 7.0 6.5 6.0 5.5 5.0 4.5 4.0 3.5 3.0 2.5 2.0 1.5 1.0 0.5 0.0

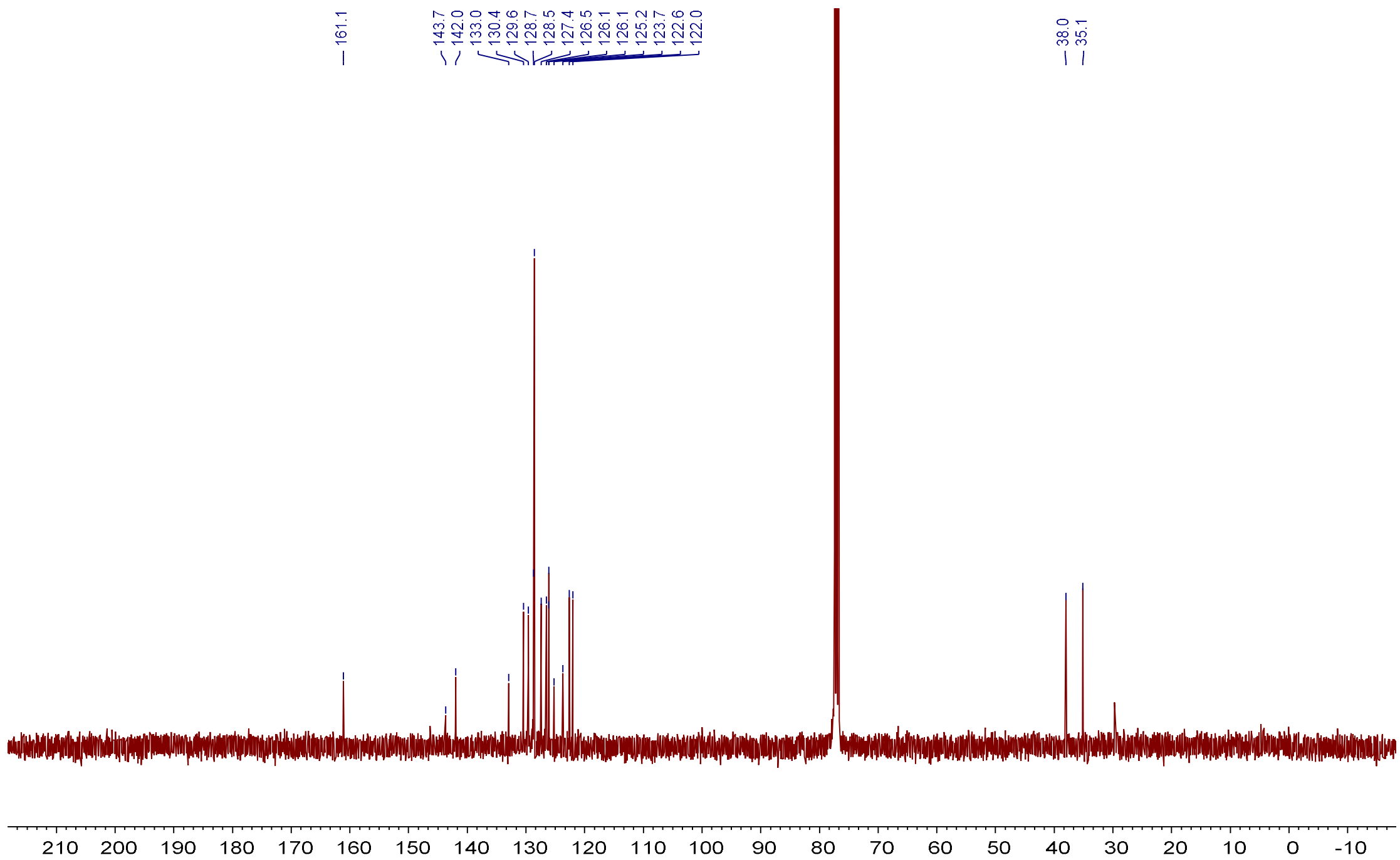
161.1
143.7
142.0
133.0
130.4
129.6
128.7
128.5
127.4
126.5
126.1
126.1
125.2
123.7
122.6
122.0
38.0
35.1
210 200 190 180 170 160 150 140 130 120 110 100 90 80 70 60 50 40 30 20 10 0 -10

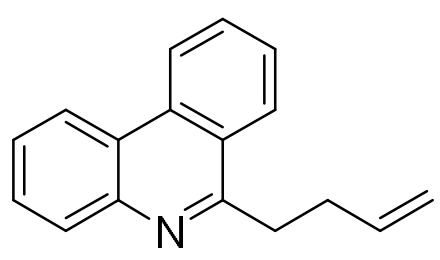

5k

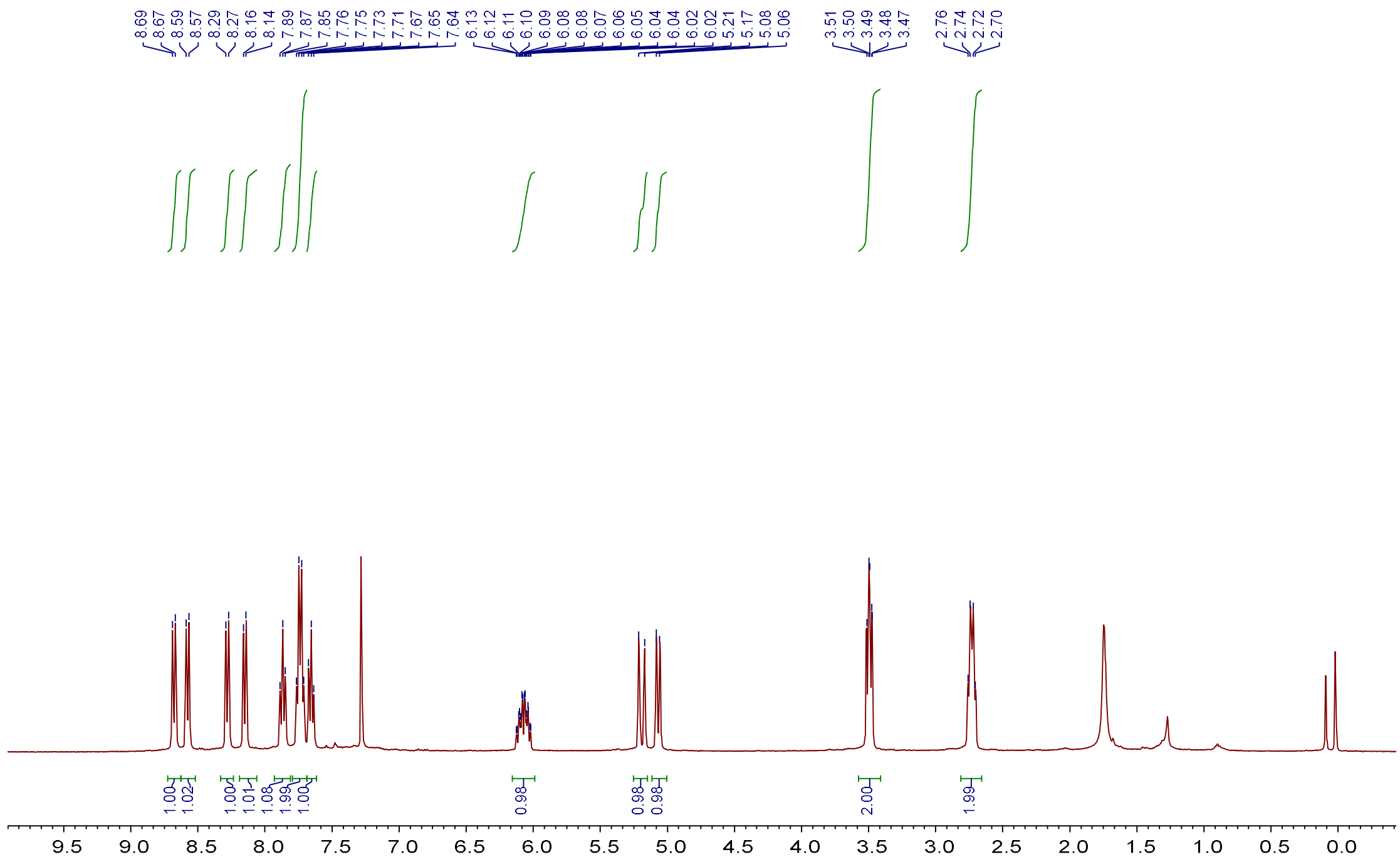

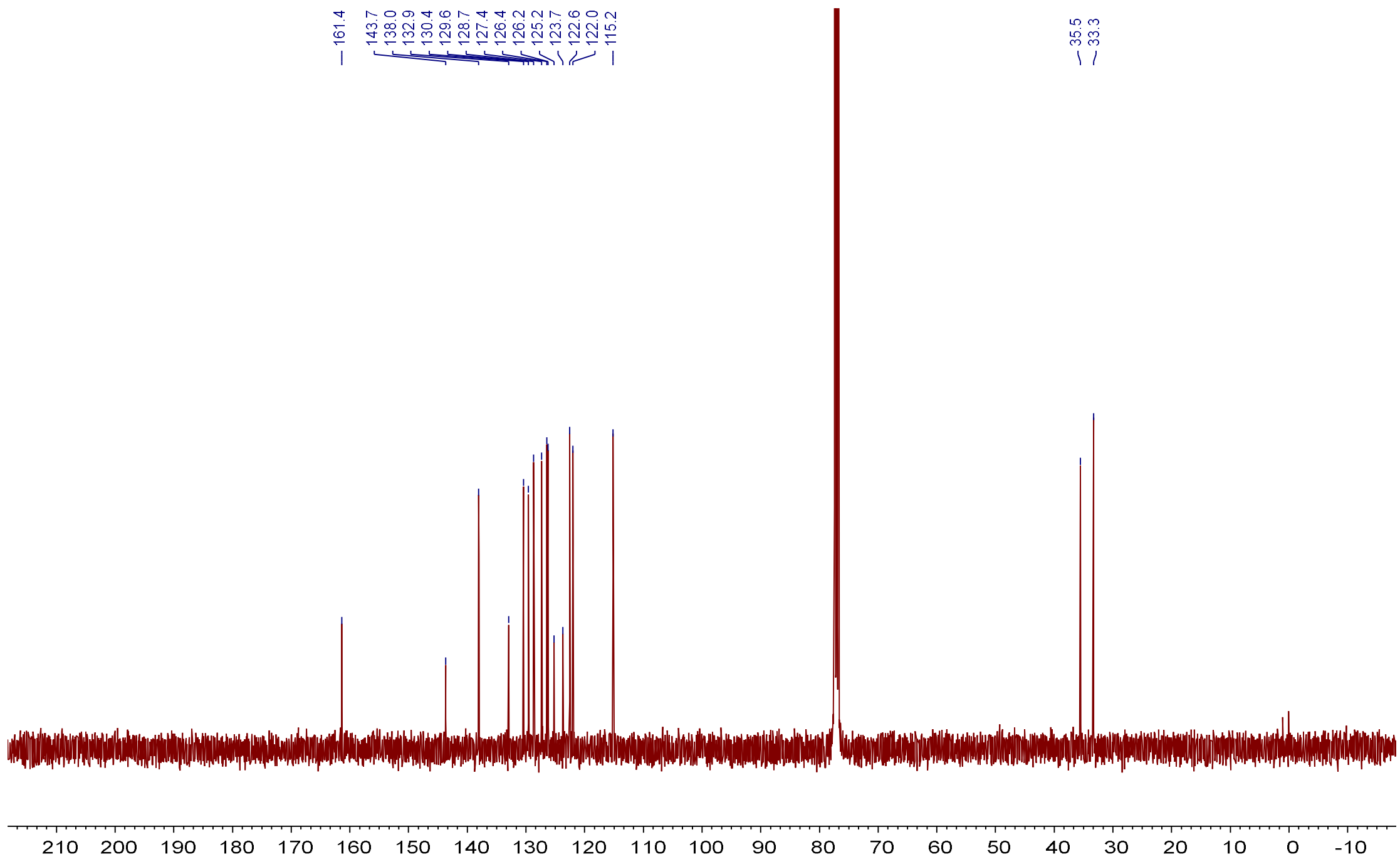

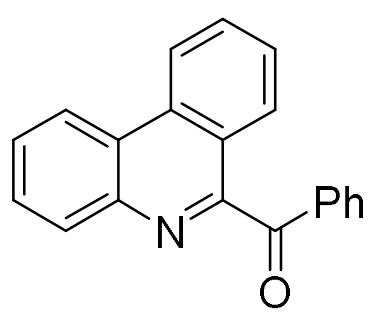

5l

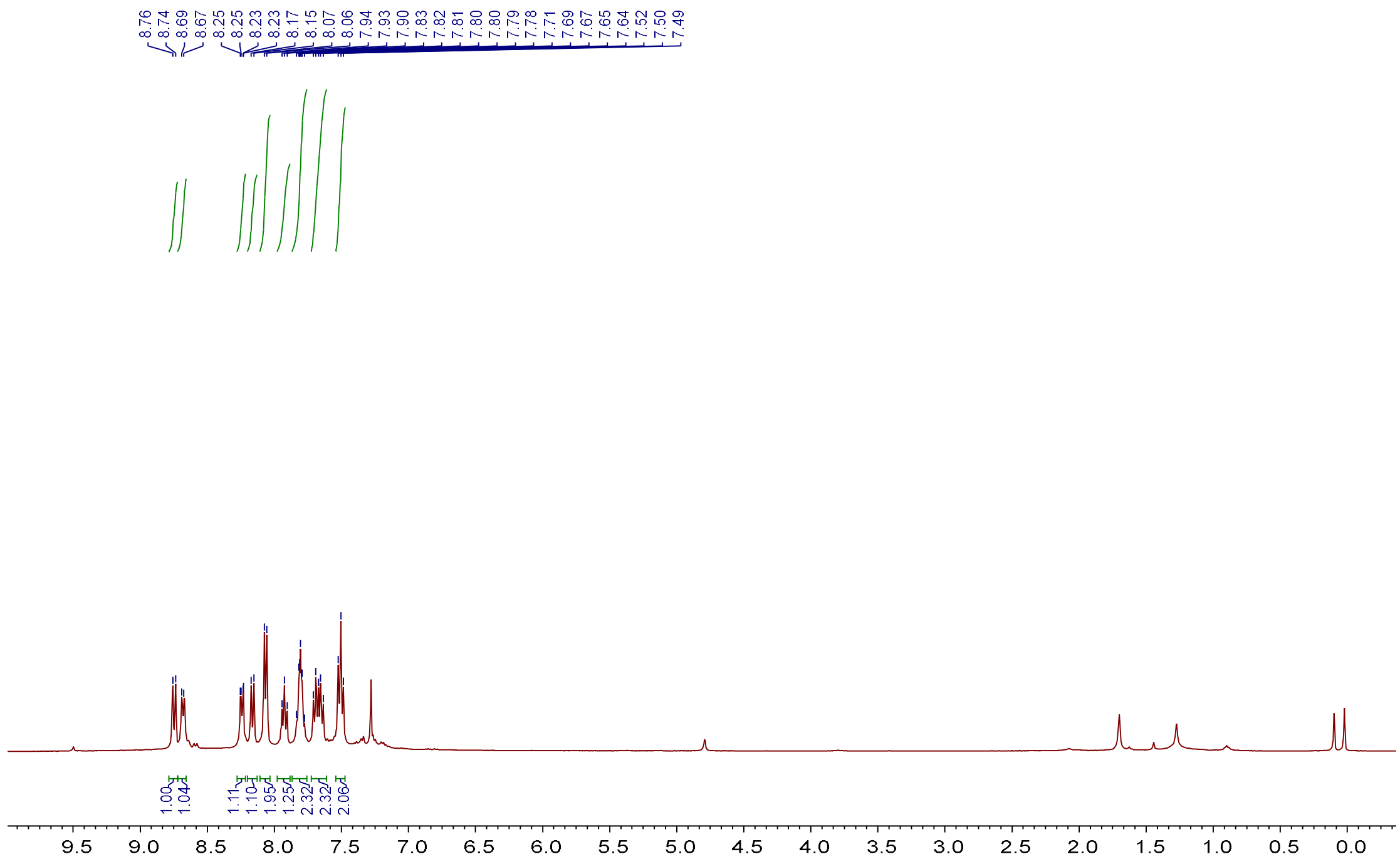

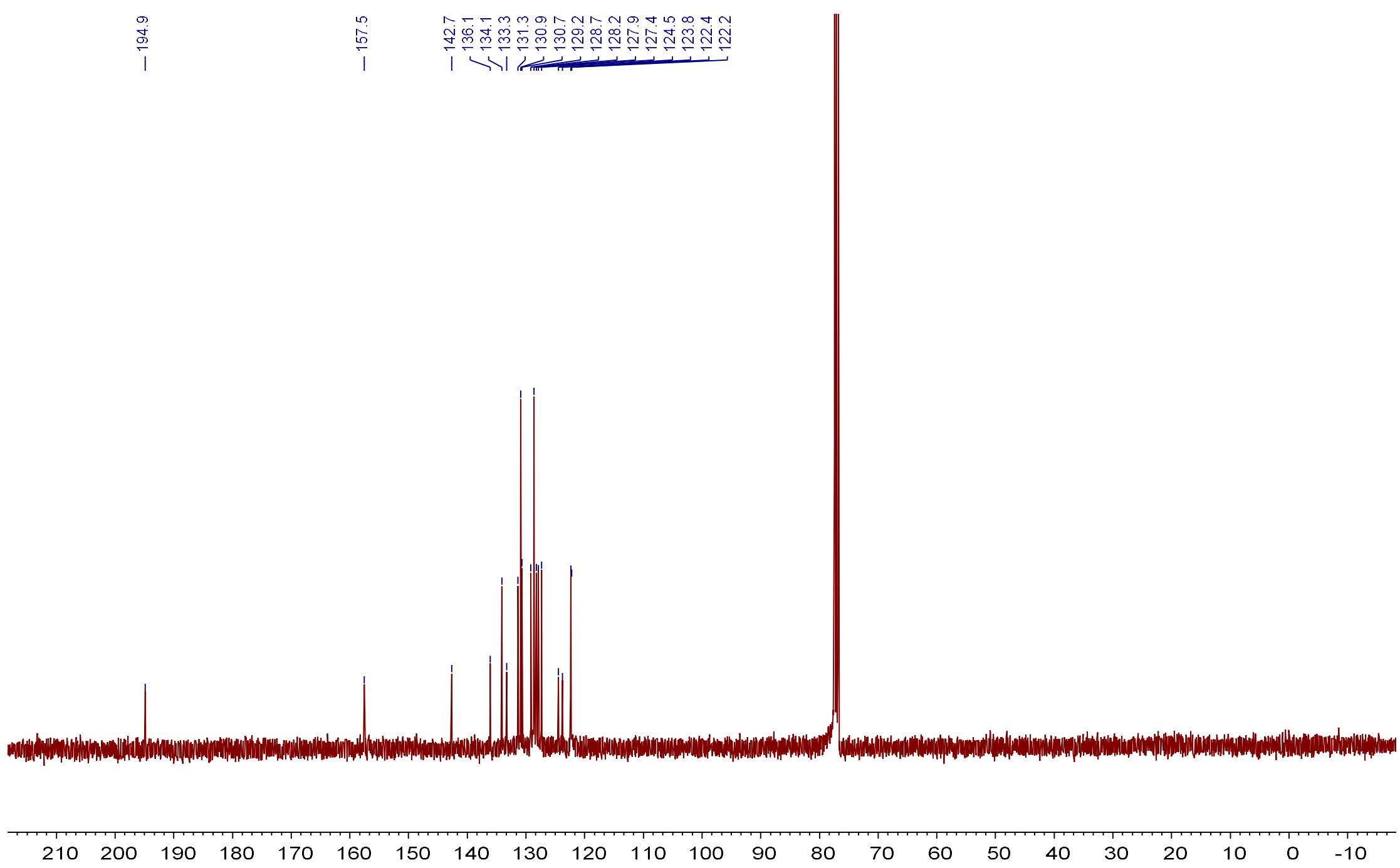

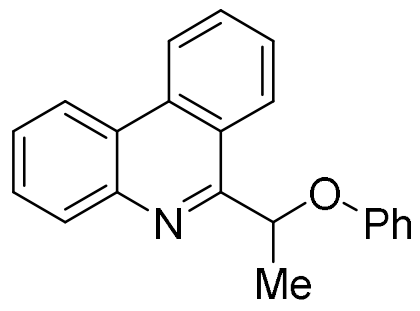
N
O
Ph
Me

5m

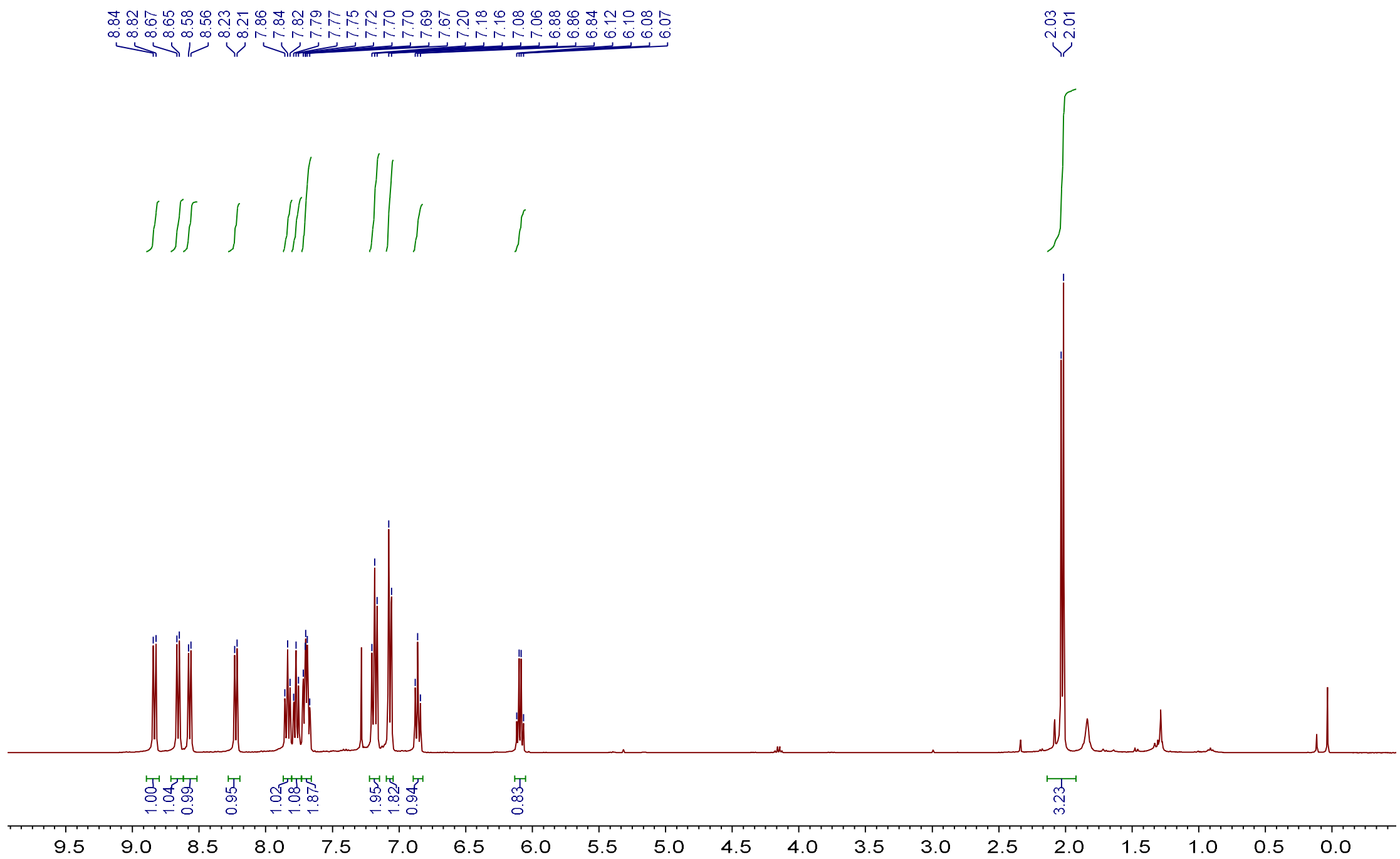
8.84 8.82 8.67 8.65 8.58 8.56 8.23 8.21 7.86 7.84 7.82 7.79 7.77 7.75 7.72 7.70 7.69 7.67 7.20 7.18 7.16 7.08 7.06 6.88 6.86 6.84 6.12 6.10 6.08 6.07
2.03 2.01
1.00 1.04 0.99 0.95 1.02 1.08 1.87 1.95 1.82 0.94 0.83 3.23
9.5 9.0 8.5 8.0 7.5 7.0 6.5 6.0 5.5 5.0 4.5 4.0 3.5 3.0 2.5 2.0 1.5 1.0 0.5 0.0

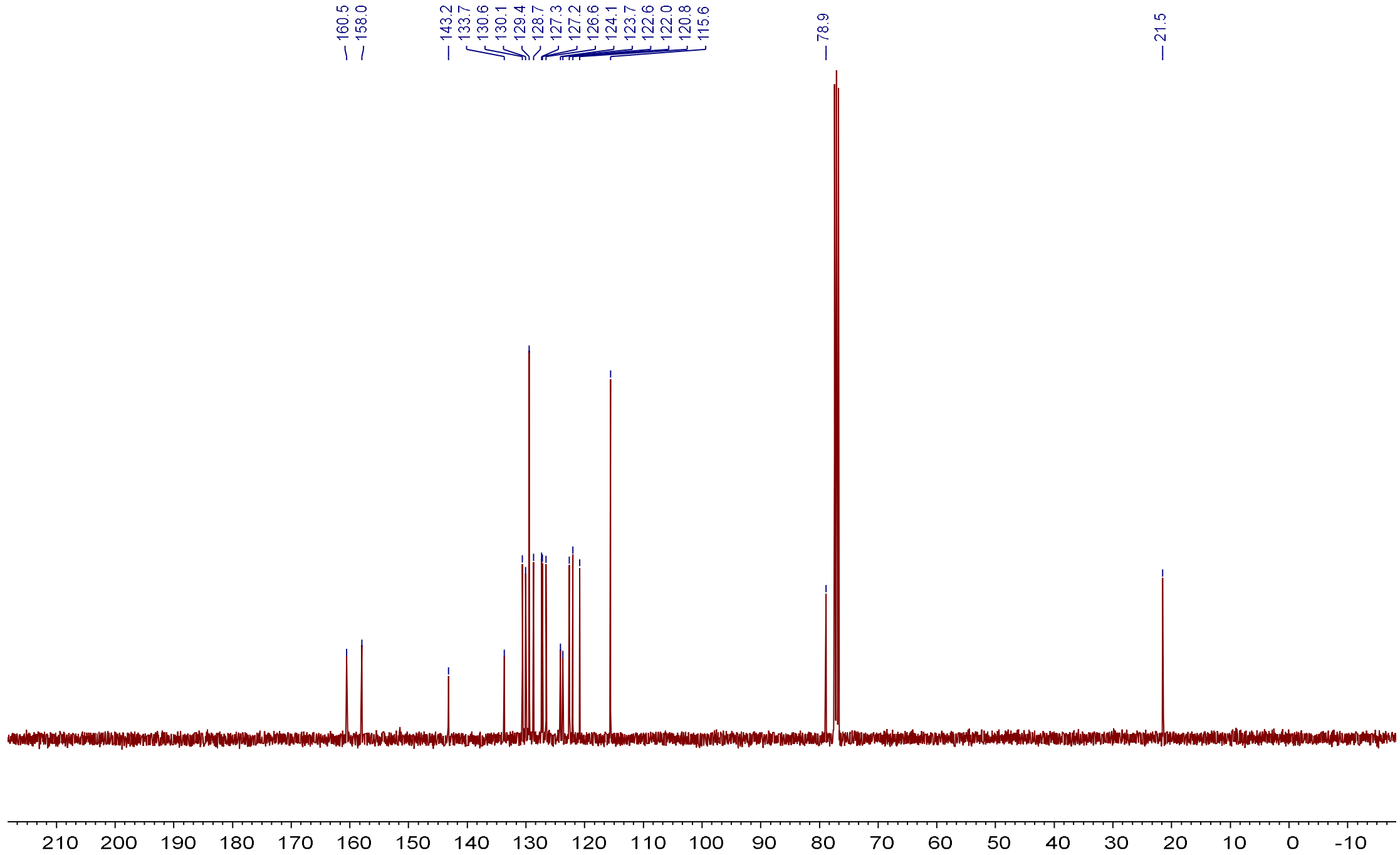
160.5 158.0
143.2 133.7 130.6 130.1 129.4 128.7 127.3 127.2 126.6 124.1 123.7 122.6 122.0 120.8 115.6
78.9
21.5
210 200 190 180 170 160 150 140 130 120 110 100 90 80 70 60 50 40 30 20 10 0 -10

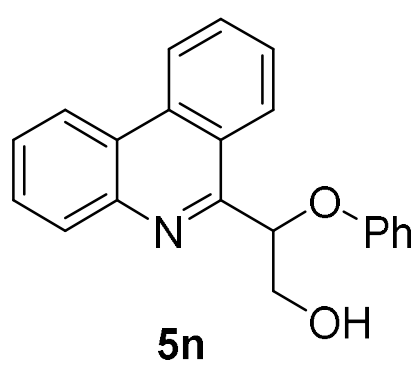

5n

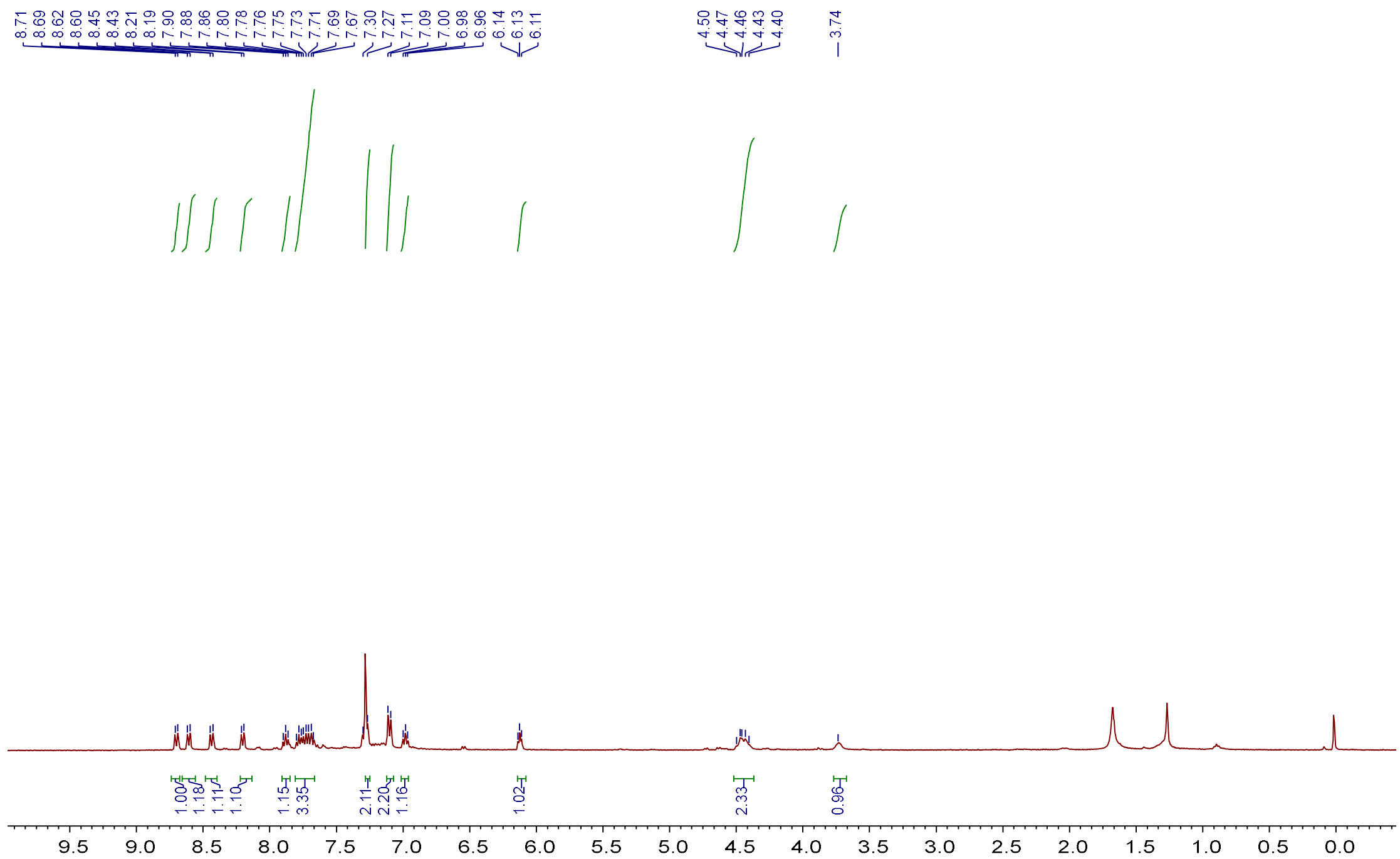

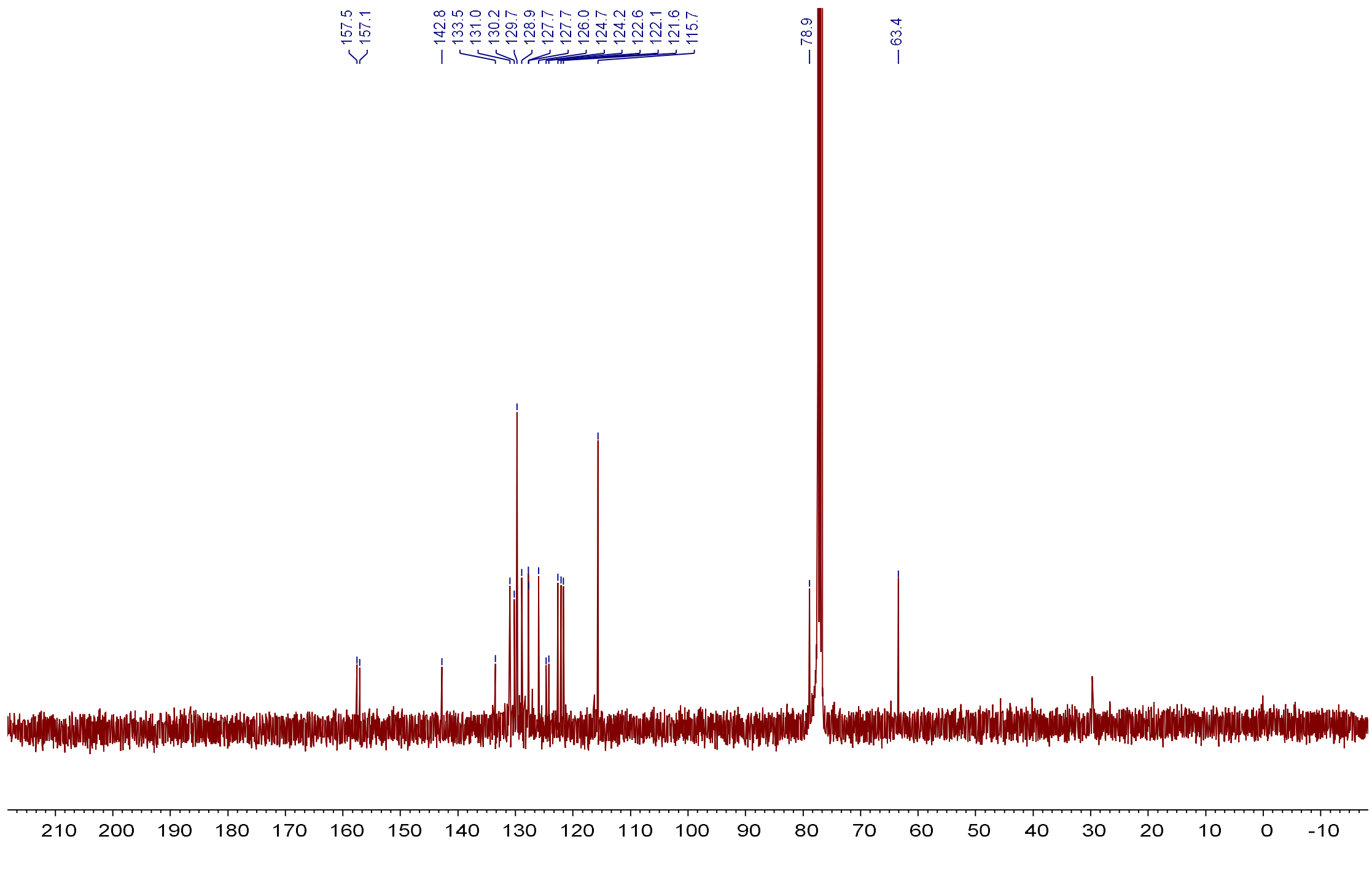

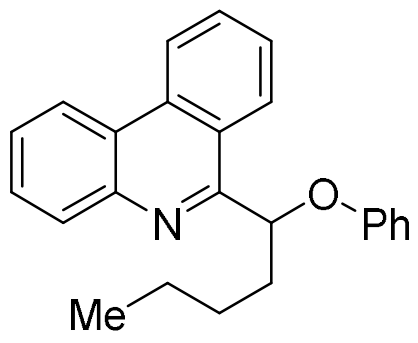
O
Ph
N
Me

5o

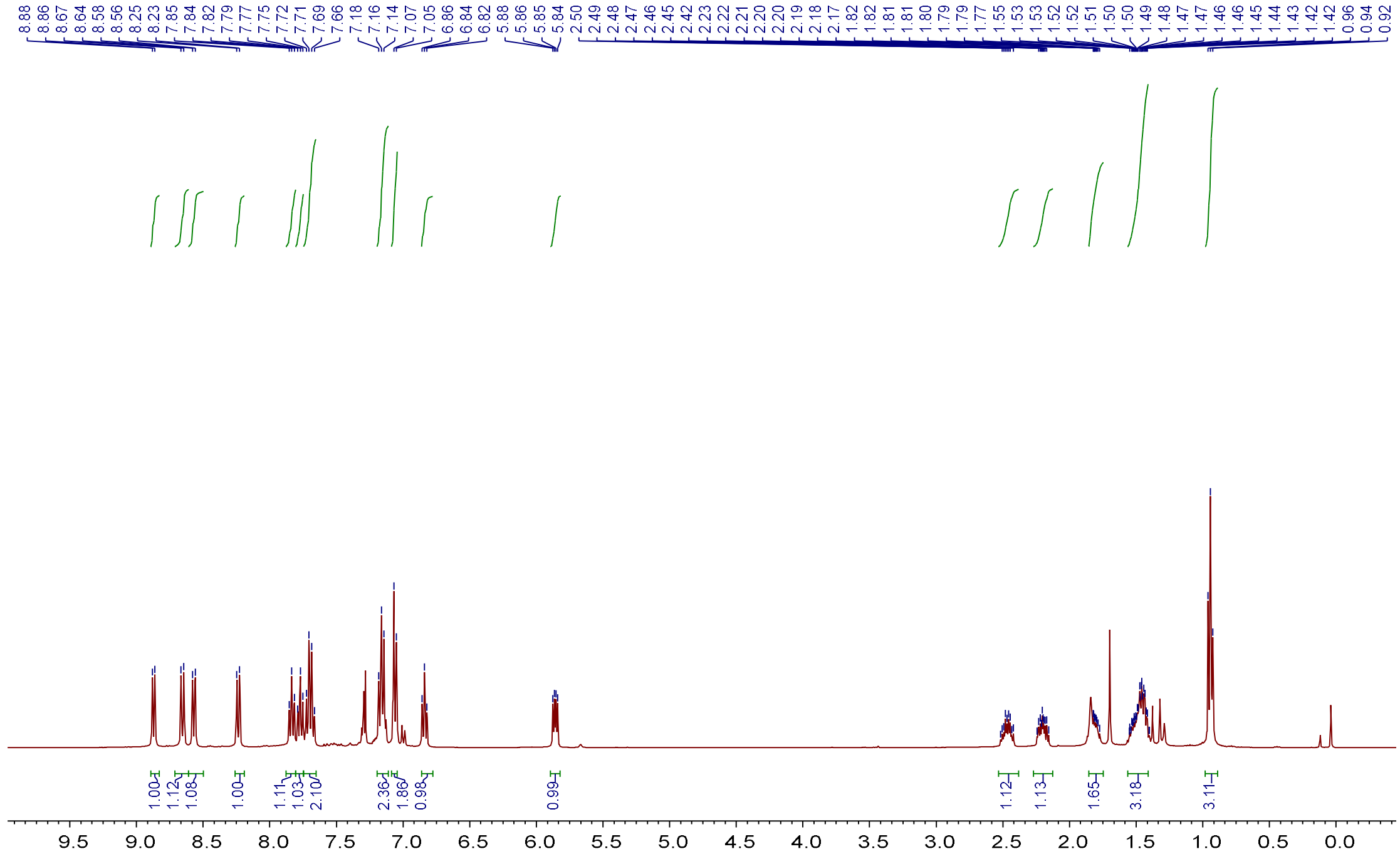
8.88 8.86 8.67 8.64 8.58 8.56 8.25 8.23 7.85 7.84 7.82 7.79 7.77 7.75 7.72 7.71 7.69 7.66 7.18 7.16 7.14 7.07 7.05 6.86 6.84 6.82 5.88 5.86 5.85 5.84 2.50 2.49 2.48 2.47 2.46 2.45 2.42 2.23 2.22 2.21 2.20 2.19 2.18 2.17 1.82 1.82 1.81 1.81 1.80 1.79 1.79 1.77 1.55 1.53 1.53 1.52 1.52 1.51 1.50 1.50 1.49 1.48 1.47 1.47 1.46 1.46 1.45 1.44 1.43 1.42 1.42 0.96 0.94 0.92
1.00 1.12 1.08 1.00 1.11 1.03 2.10 2.36 1.86 0.98 0.99 1.12 1.13 1.65 3.18 3.11
9.5 9.0 8.5 8.0 7.5 7.0 6.5 6.0 5.5 5.0 4.5 4.0 3.5 3.0 2.5 2.0 1.5 1.0 0.5 0.0

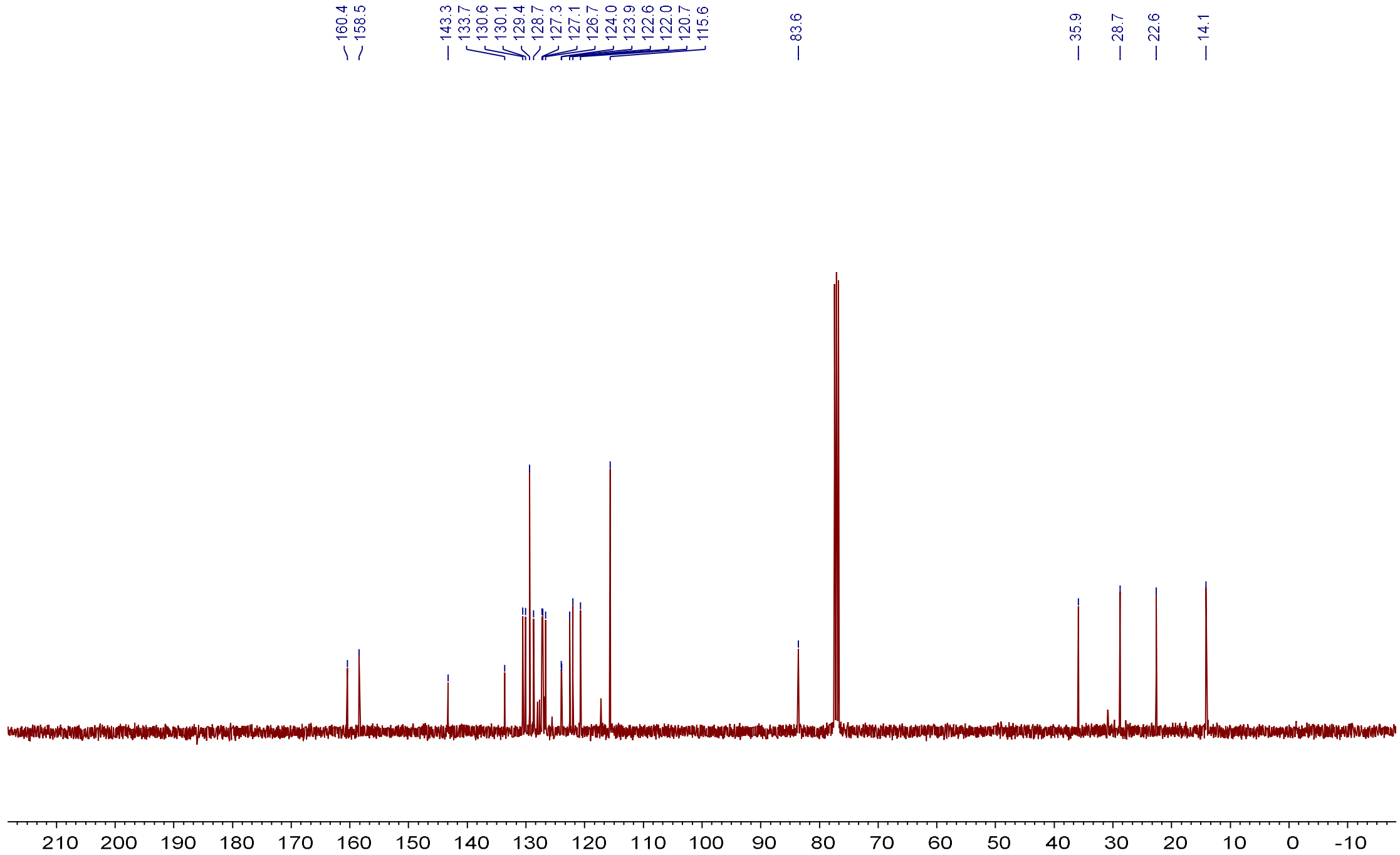
160.4 158.5 143.3 133.7 130.6 130.1 129.4 128.7 127.3 127.1 126.7 124.0 123.9 122.6 122.0 120.7 115.6 83.6 35.9 28.7 22.6 14.1
210 200 190 180 170 160 150 140 130 120 110 100 90 80 70 60 50 40 30 20 10 0 -10

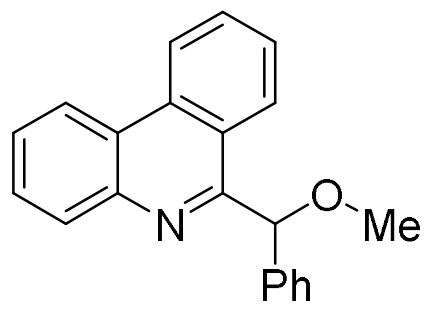

5p

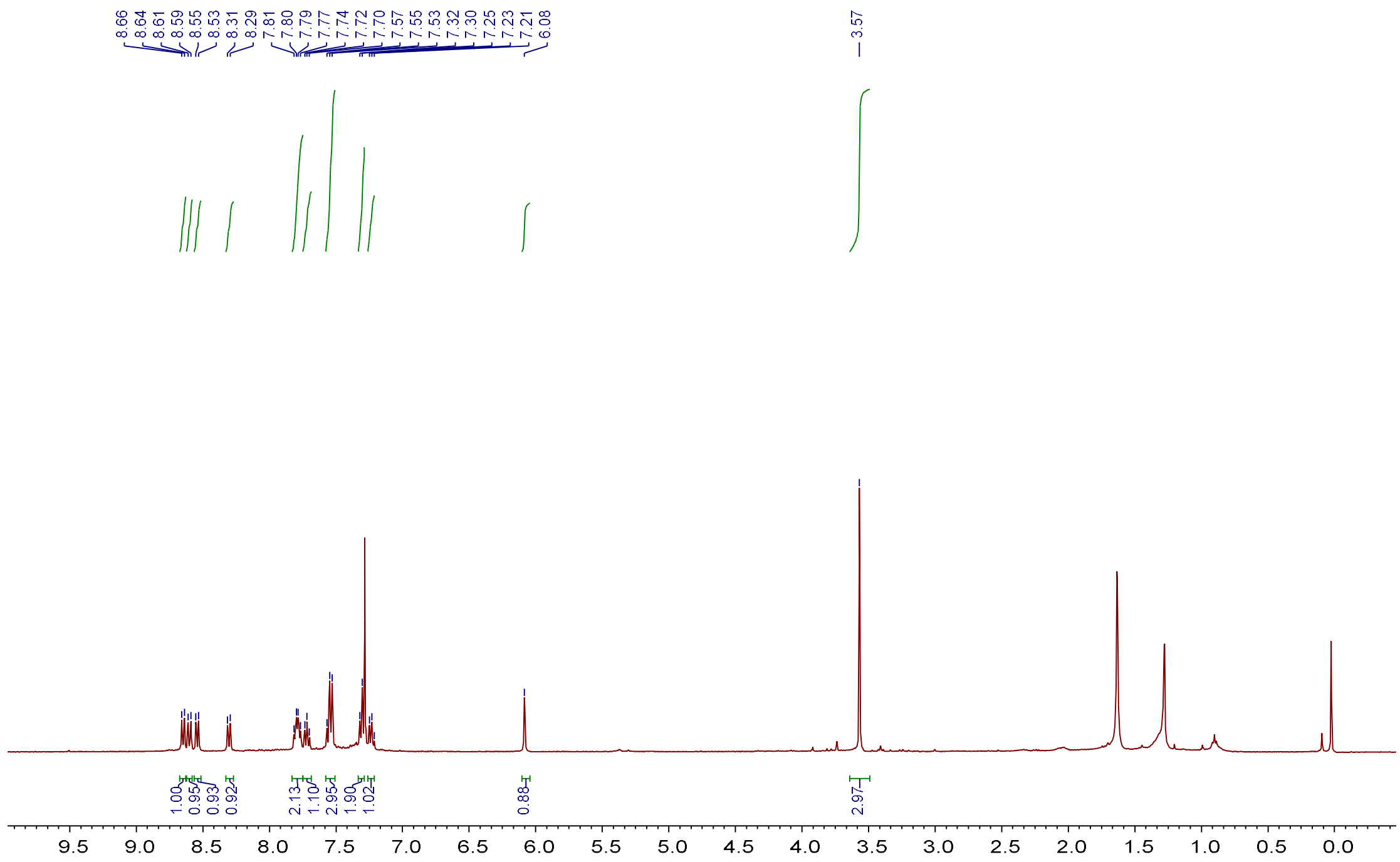

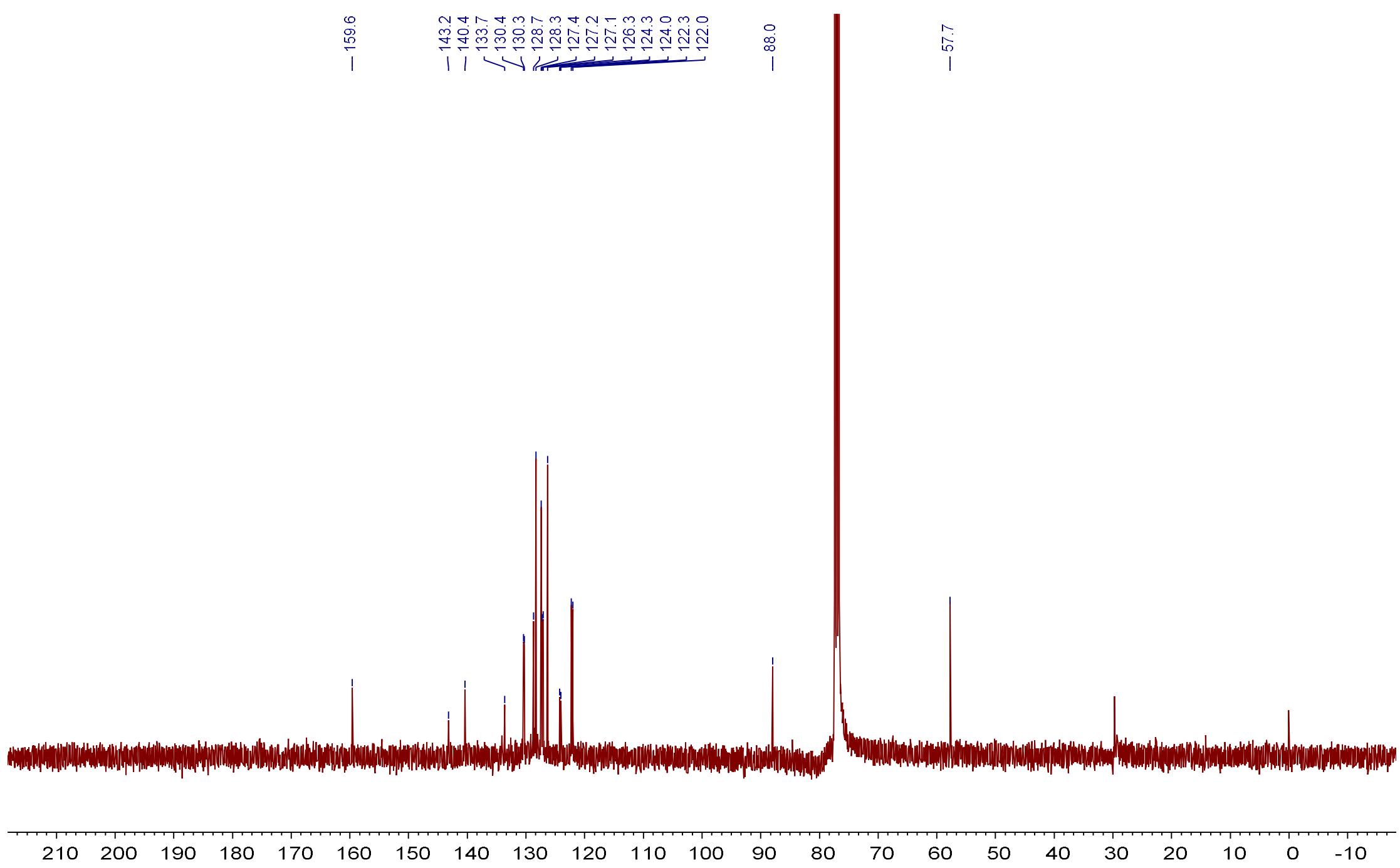

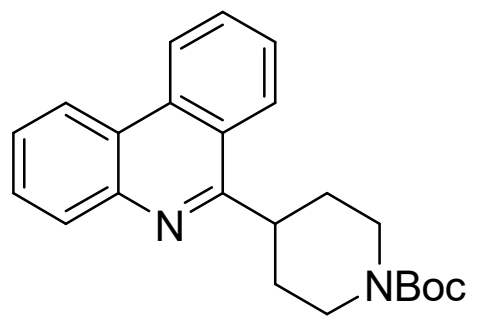
N
NBoc

5q

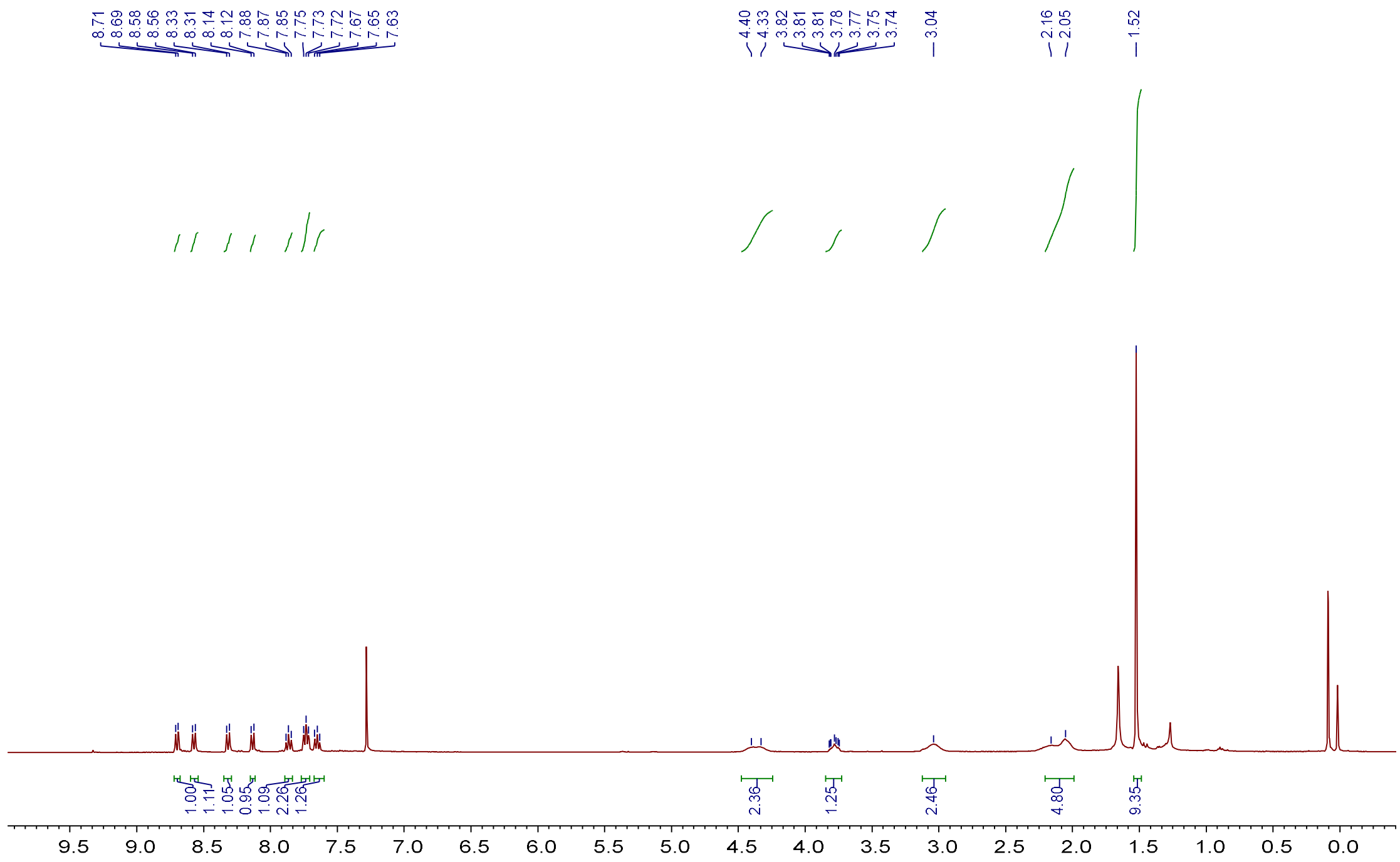
8.71
8.69
8.58
8.56
8.33
8.31
8.14
8.12
7.88
7.87
7.85
7.75
7.73
7.72
7.67
7.65
7.63
4.40
4.33
3.82
3.81
3.81
3.78
3.77
3.75
3.74
3.04
2.16
2.05
1.52
1.00
1.11
1.05
0.95
1.09
2.26
1.26
2.36
1.25
2.46
4.80
9.35
9.5
9.0
8.5
8.0
7.5
7.0
6.5
6.0
5.5
5.0
4.5
4.0
3.5
3.0
2.5
2.0
1.5
1.0
0.5
0.0

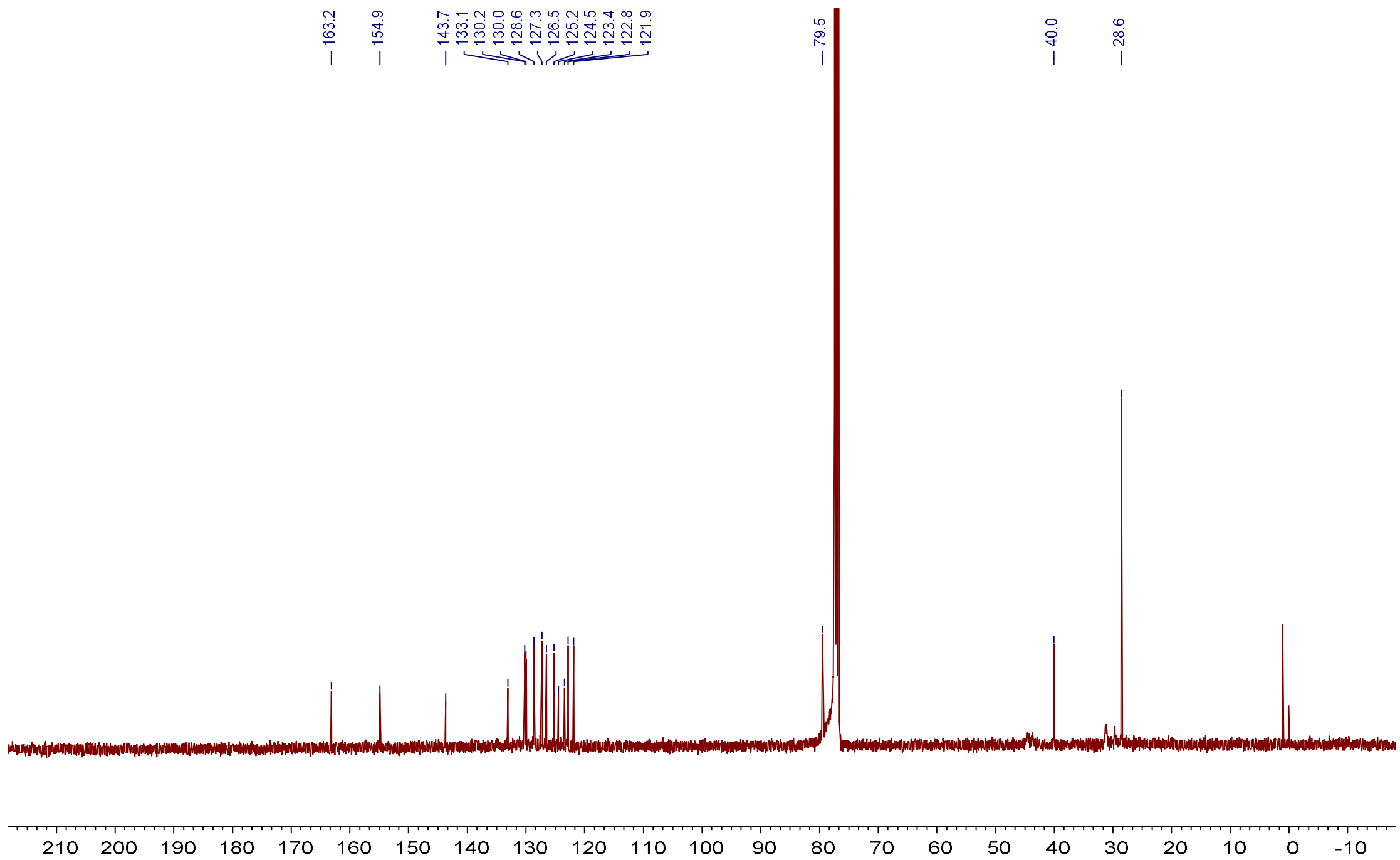
163.2
154.9
143.7
133.1
130.2
130.0
128.6
127.3
126.5
125.2
124.5
123.4
122.8
121.9
79.5
40.0
28.6
210
200
190
180
170
160
150
140
130
120
110
100
90
80
70
60
50
40
30
20
10
0
-10

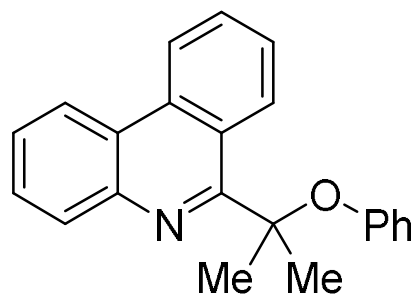
N
O
Ph
Me
Me

5r

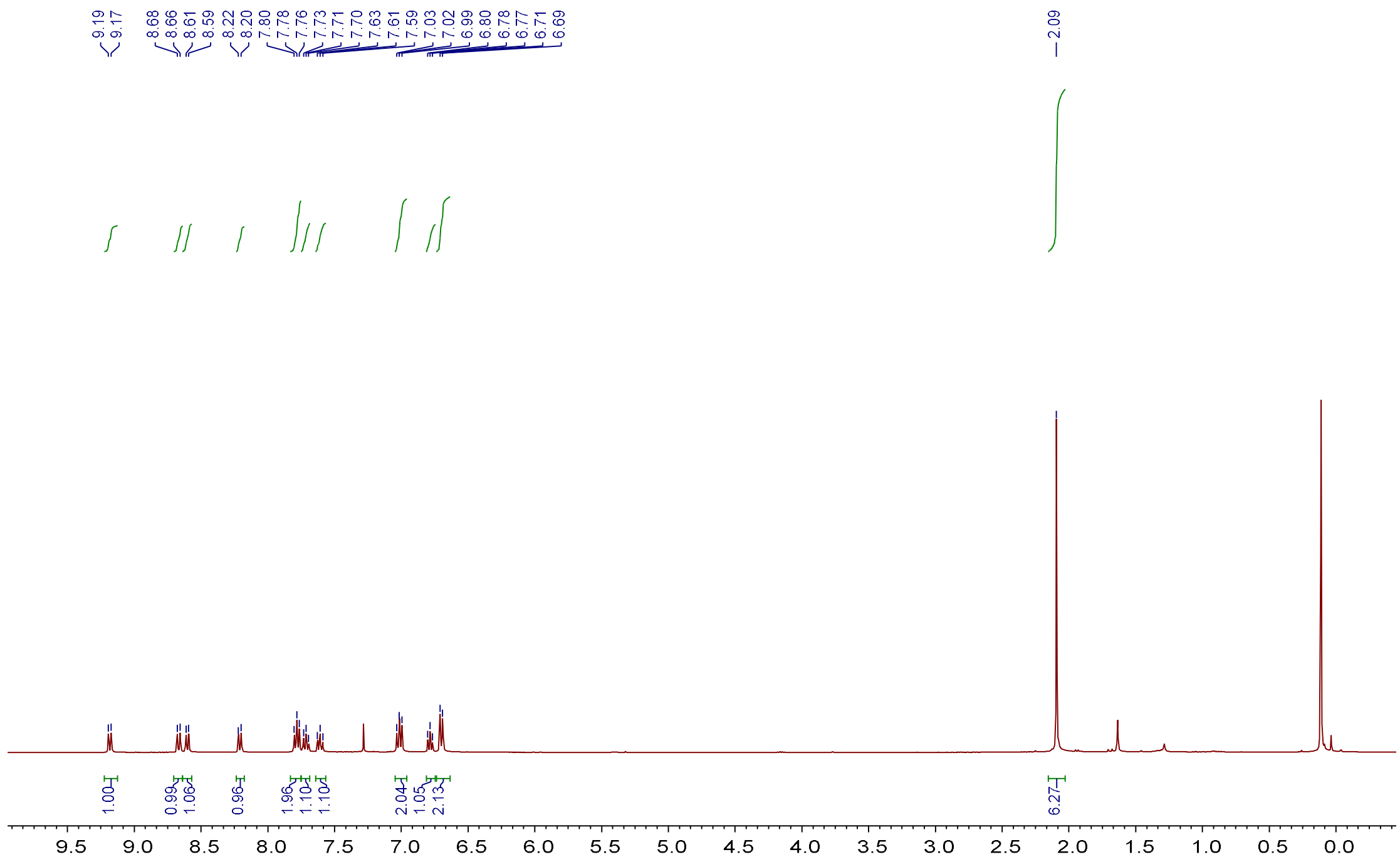
9.19
9.17
8.68
8.66
8.61
8.59
8.22
8.20
7.80
7.78
7.76
7.73
7.71
7.70
7.63
7.61
7.59
7.03
7.02
6.99
6.80
6.78
6.77
6.71
6.69
2.09
1.00
0.99
1.06
0.96
1.96
1.10
1.10
2.04
1.05
2.13
6.27
9.5 9.0 8.5 8.0 7.5 7.0 6.5 6.0 5.5 5.0 4.5 4.0 3.5 3.0 2.5 2.0 1.5 1.0 0.5 0.0

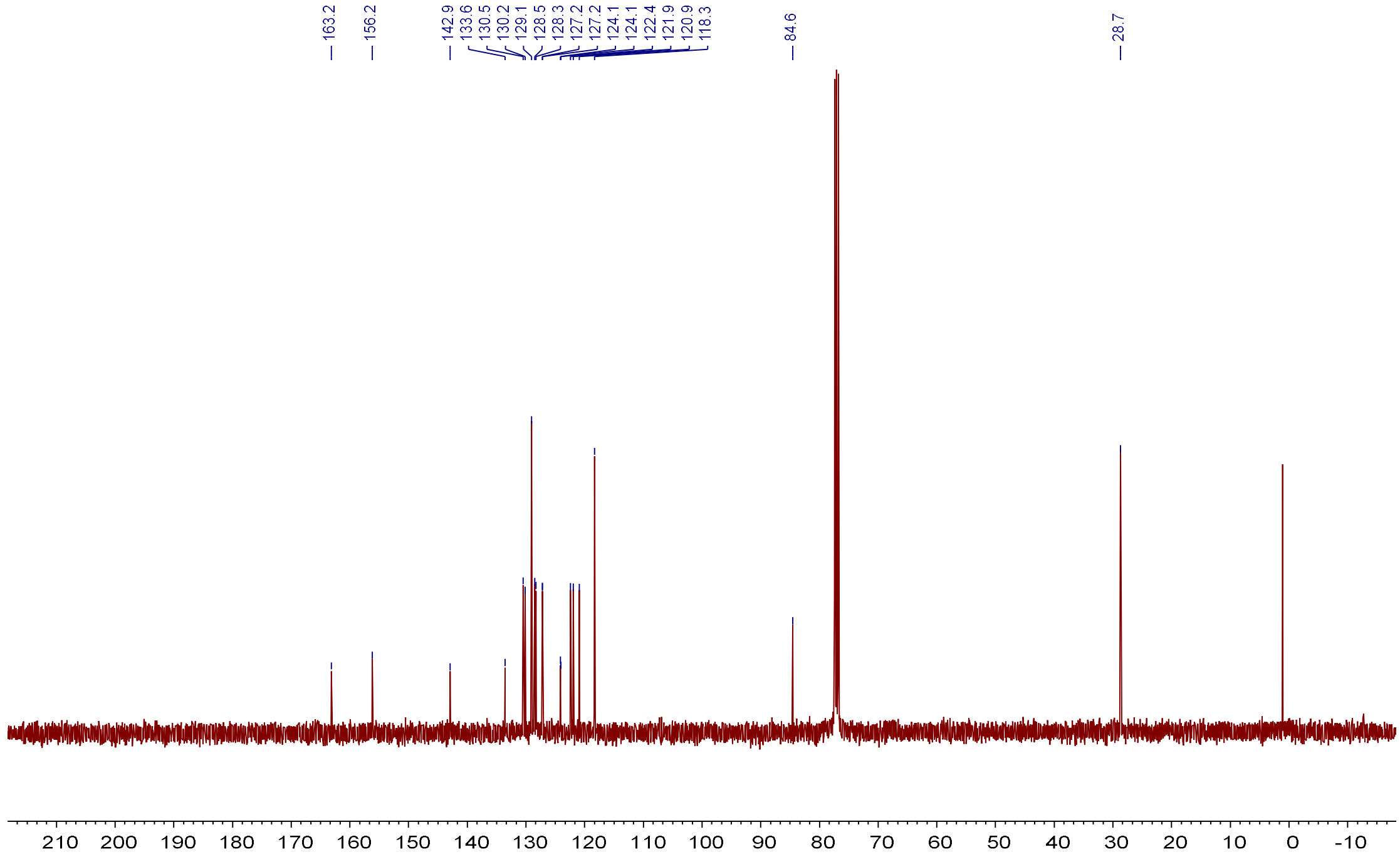
163.2
156.2
142.9
133.6
130.5
130.2
129.1
128.5
128.3
127.2
127.2
124.1
124.1
122.4
121.9
120.9
118.3
84.6
28.7
210 200 190 180 170 160 150 140 130 120 110 100 90 80 70 60 50 40 30 20 10 0 -10

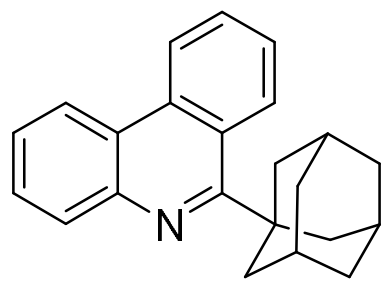

5s

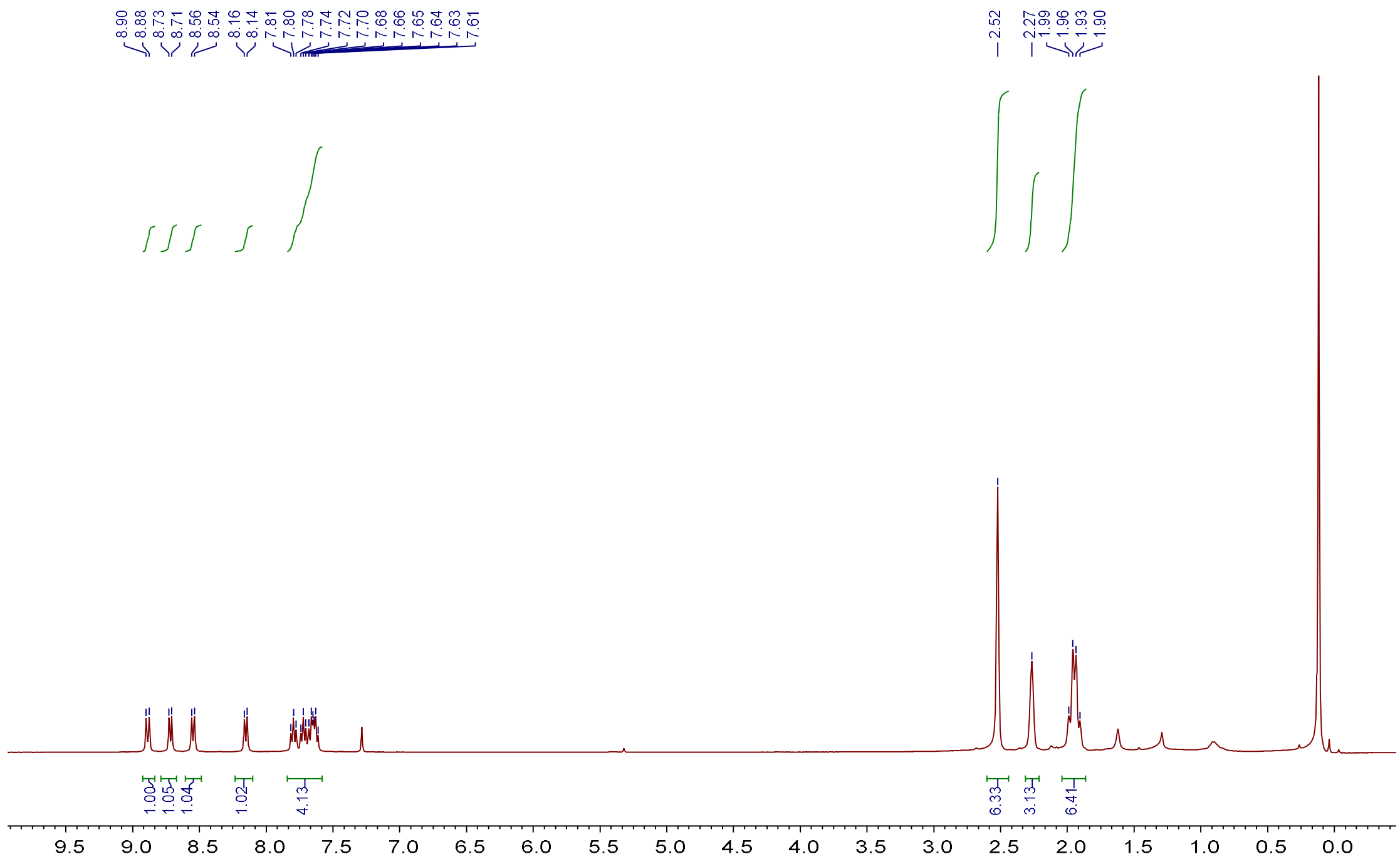
8.90
8.88
8.73
8.71
8.56
8.54
8.16
8.14
7.81
7.80
7.78
7.74
7.72
7.70
7.68
7.66
7.65
7.64
7.63
7.61
2.52
2.27
1.99
1.96
1.93
1.90
1.00
1.05
1.04
1.02
4.13
6.33
3.13
6.41
9.5 9.0 8.5 8.0 7.5 7.0 6.5 6.0 5.5 5.0 4.5 4.0 3.5 3.0 2.5 2.0 1.5 1.0 0.5 0.0

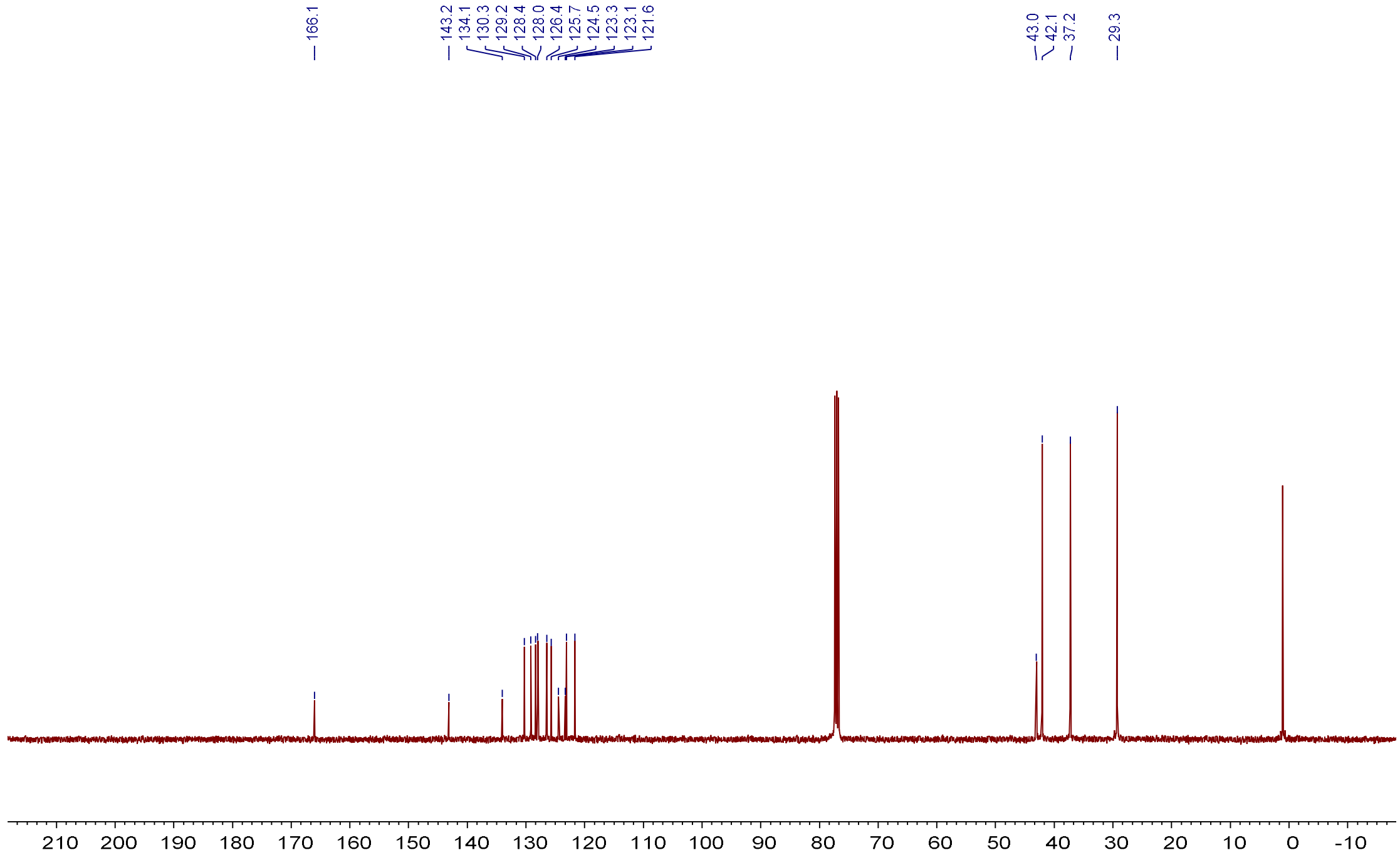
166.1
143.2
134.1
130.3
129.2
128.4
128.0
126.4
125.7
124.5
123.3
123.1
121.6
43.0
42.1
37.2
29.3
210 200 190 180 170 160 150 140 130 120 110 100 90 80 70 60 50 40 30 20 10 0 -10

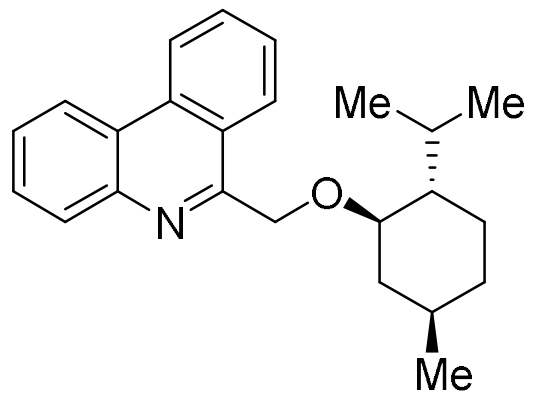

5t

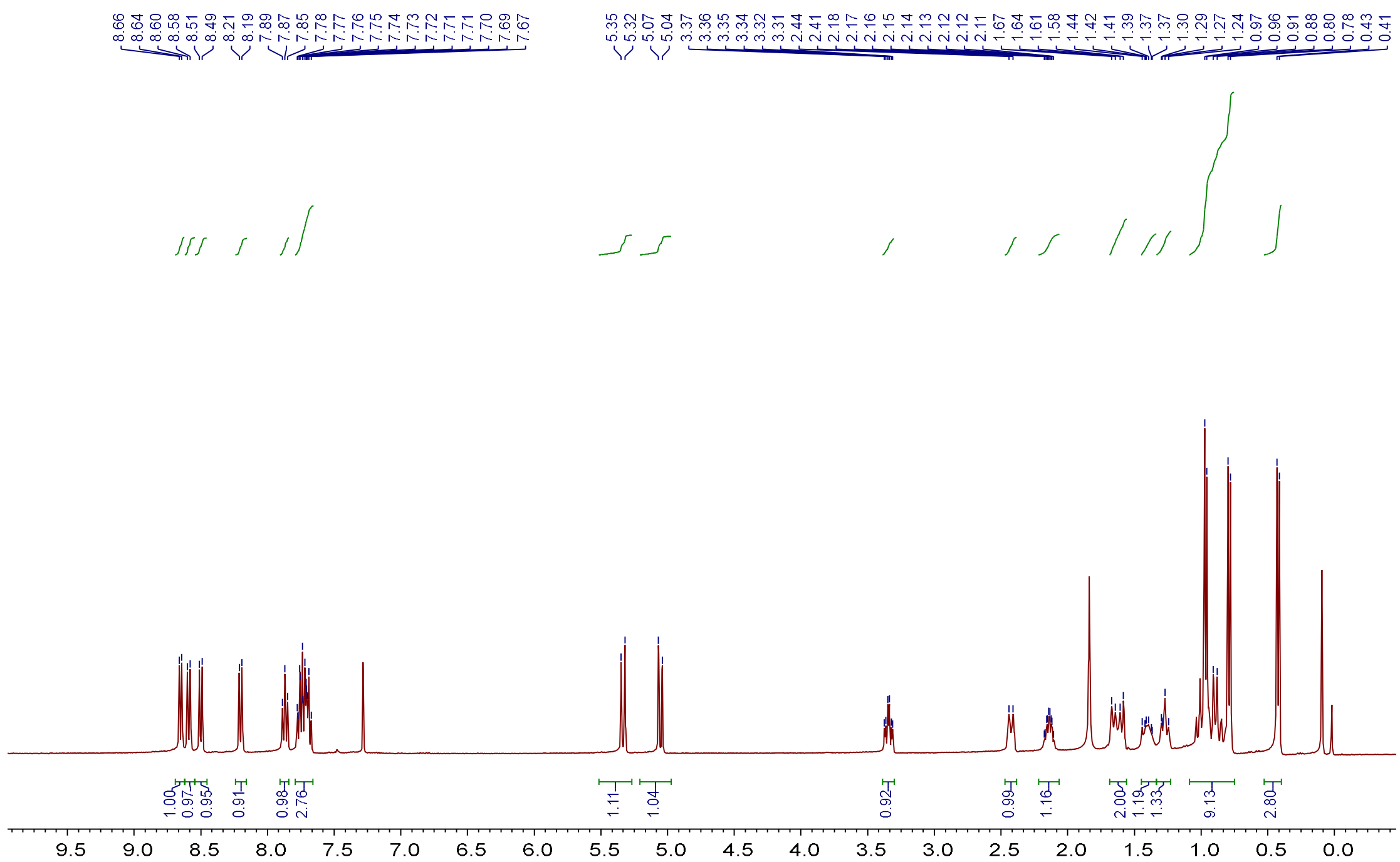

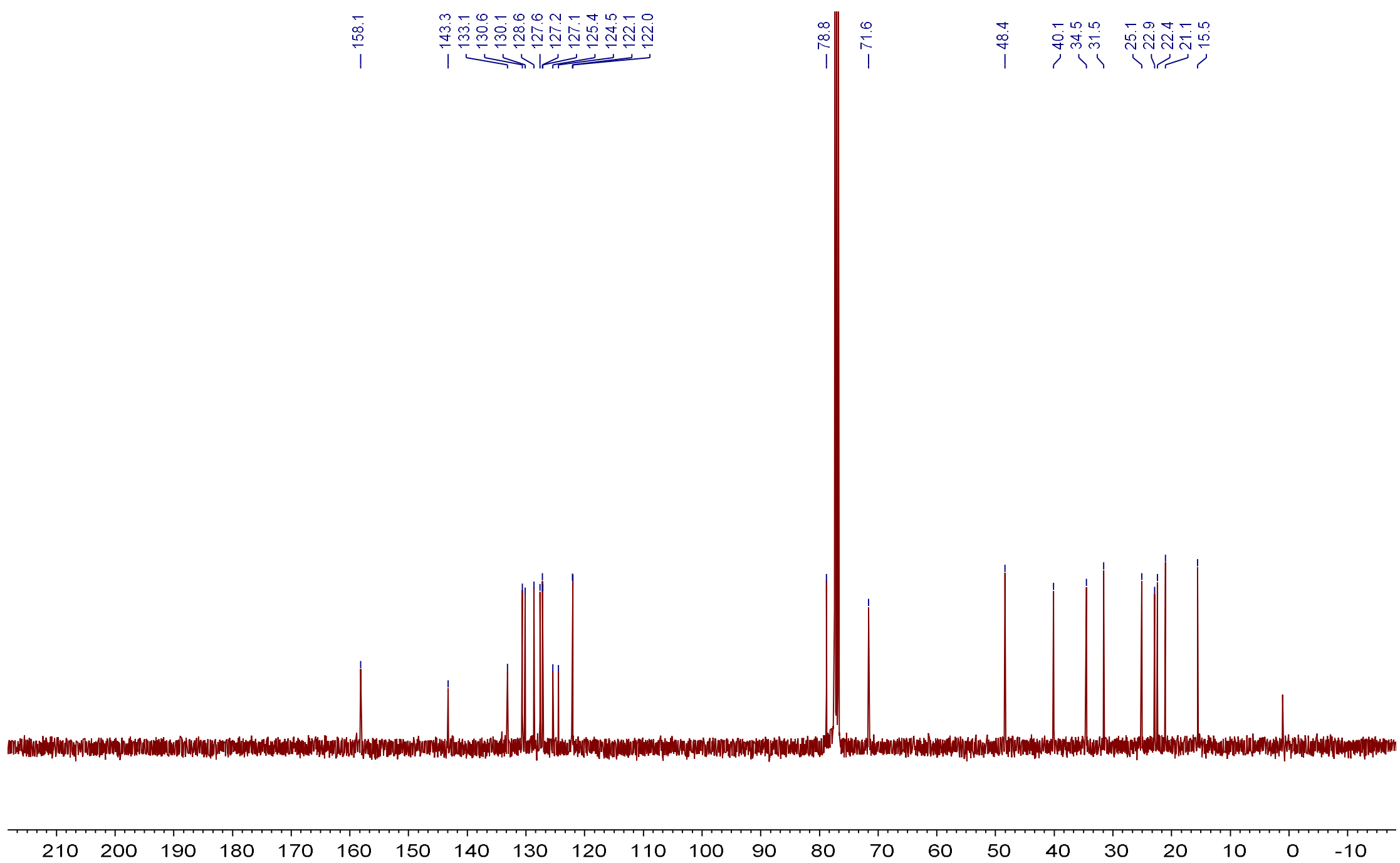

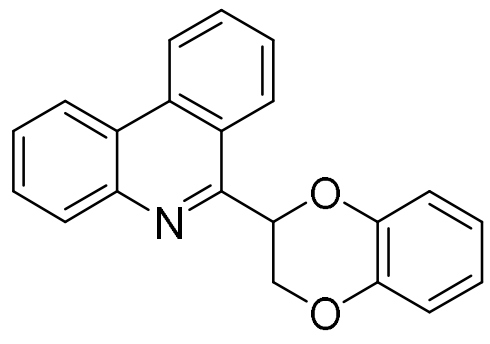
N
O
O

5u

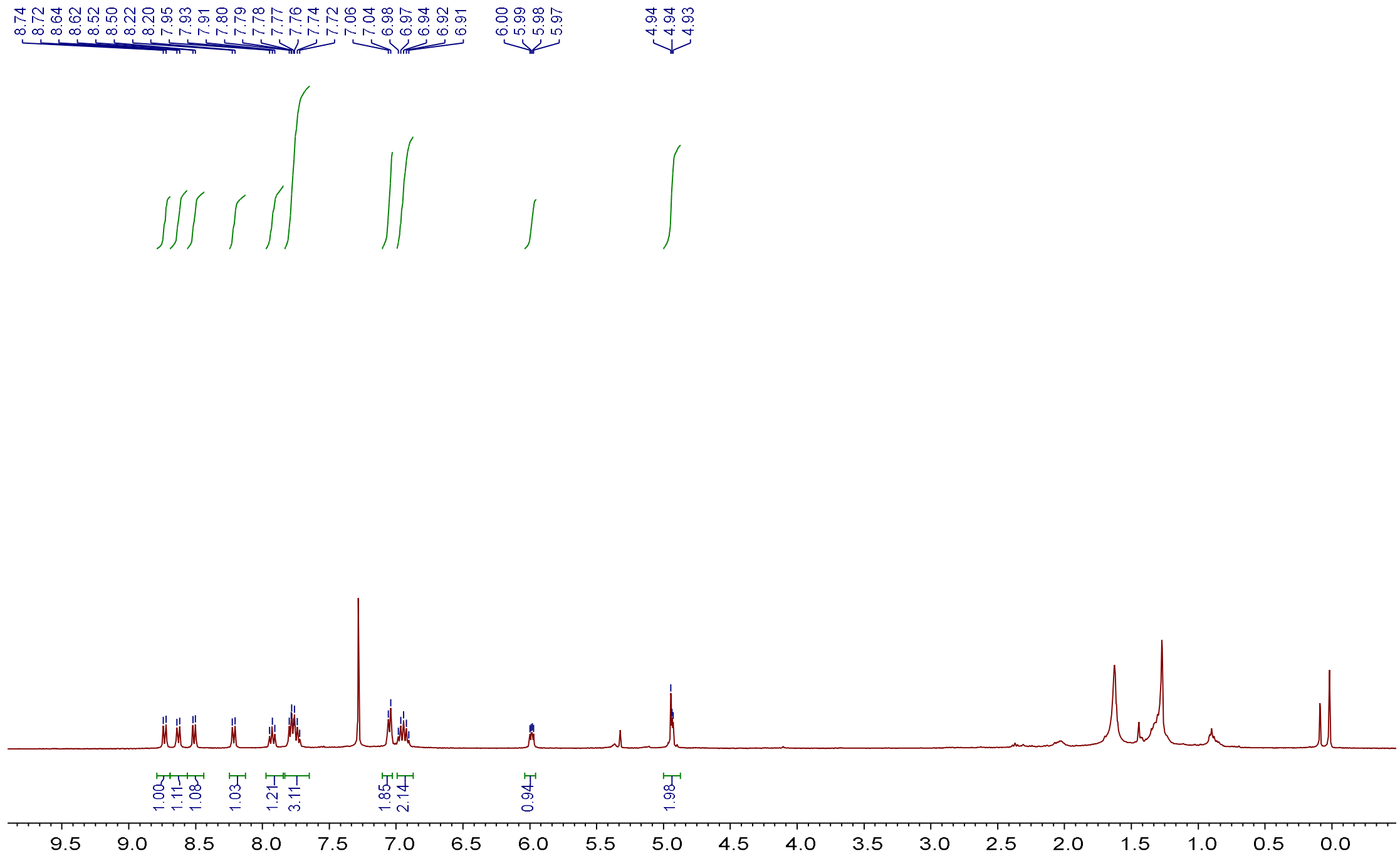
8.74 8.72 8.64 8.62 8.52 8.50 8.22 8.20 7.95 7.93 7.91 7.80 7.79 7.78 7.77 7.76 7.74 7.72 7.06 7.04 6.98 6.97 6.94 6.92 6.91 6.00 5.99 5.98 5.97 4.94 4.94 4.93
1.00 1.11 1.08 1.03 1.21 3.11 1.85 2.14 0.94 1.98
9.5 9.0 8.5 8.0 7.5 7.0 6.5 6.0 5.5 5.0 4.5 4.0 3.5 3.0 2.5 2.0 1.5 1.0 0.5 0.0

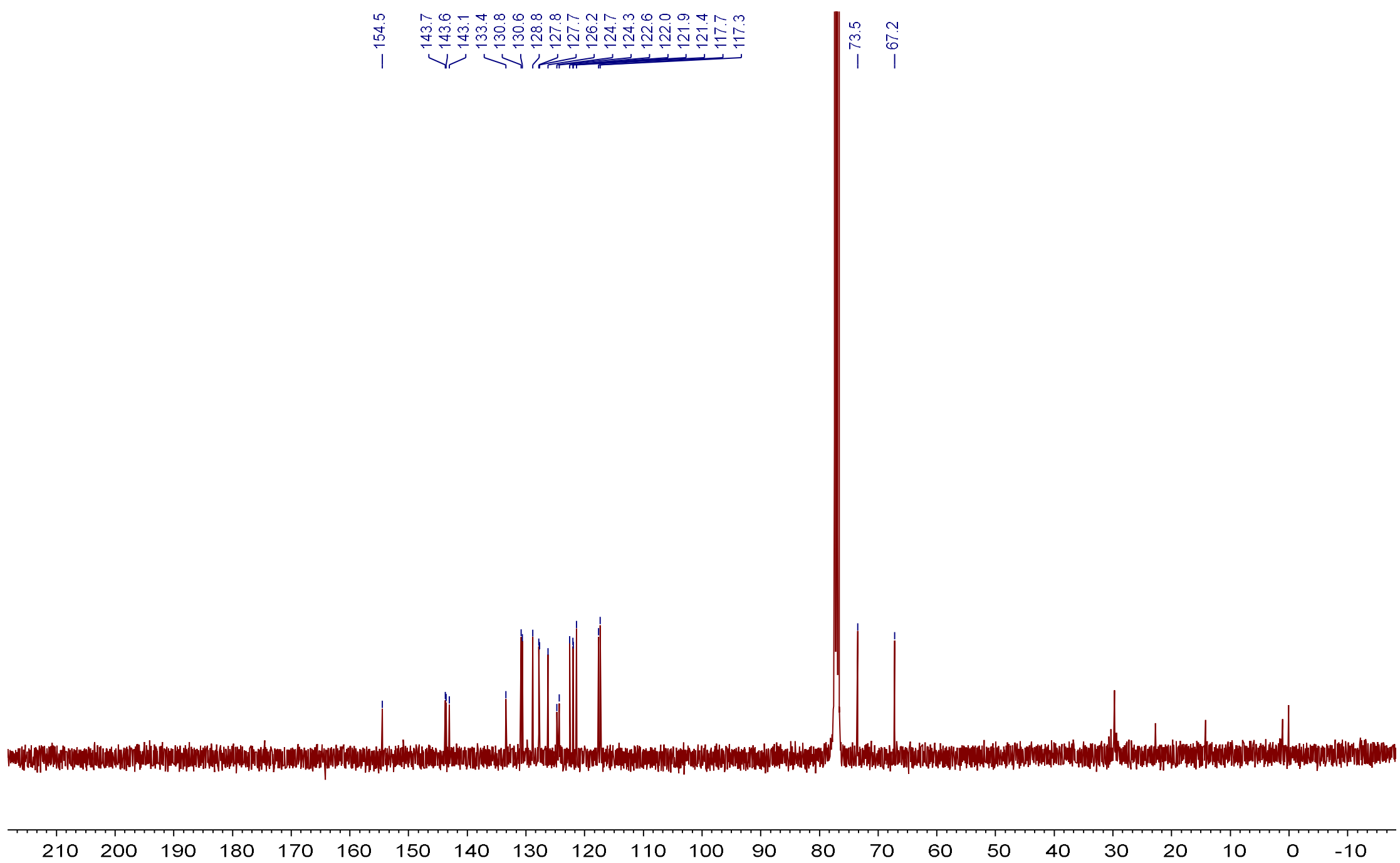
154.5
143.7 143.6 143.1 133.4 130.8 130.6 128.8 127.8 127.7 126.2 124.7 124.3 122.6 122.0 121.9 121.4 117.7 117.3
73.5
67.2
210 200 190 180 170 160 150 140 130 120 110 100 90 80 70 60 50 40 30 20 10 0 -10

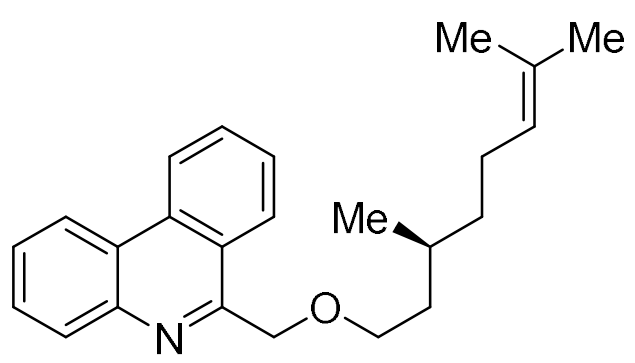
Me
Me
Me
N
O

5v

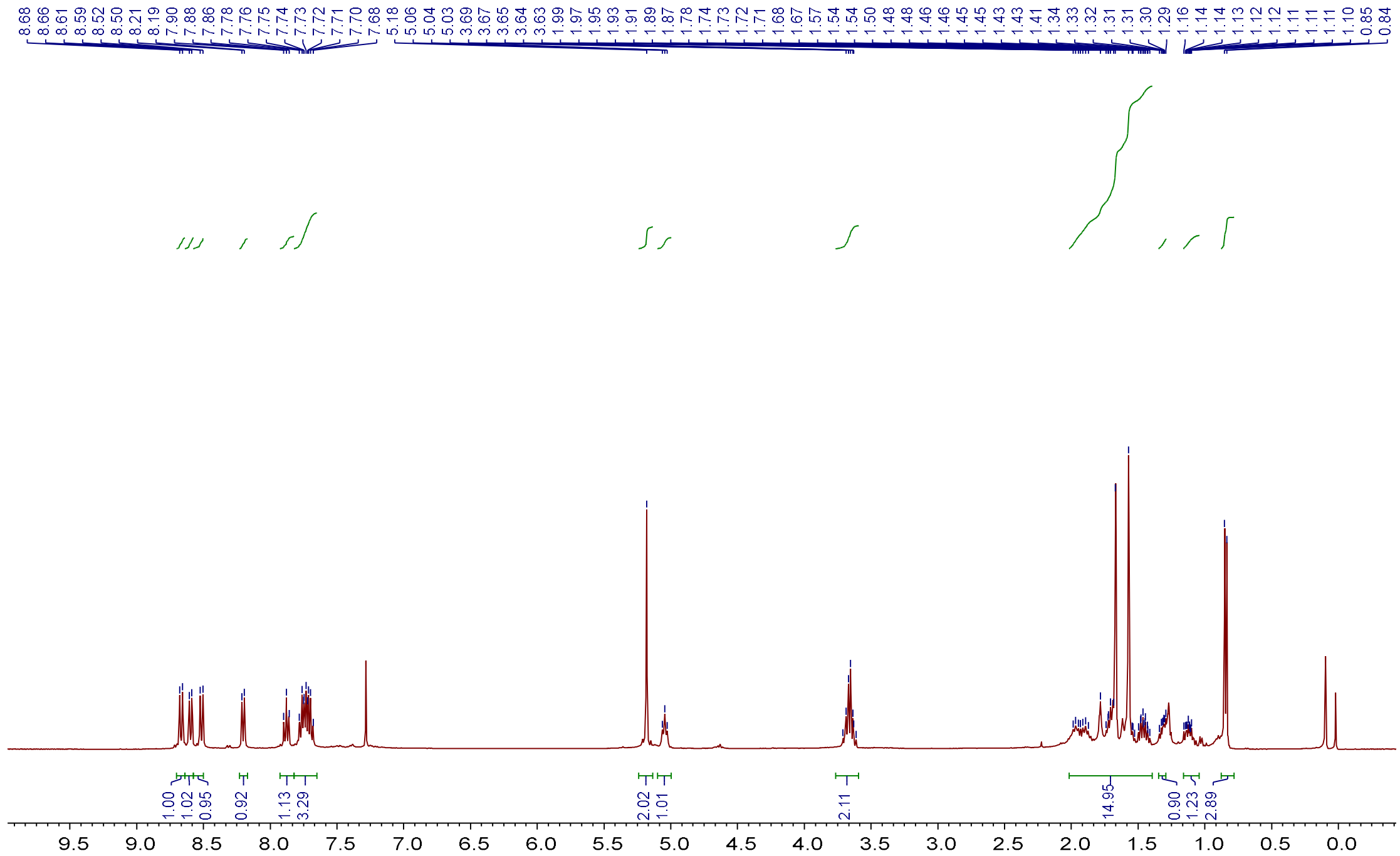
8.68 8.66 8.61 8.59 8.52 8.50 8.21 8.19 7.90 7.88 7.86 7.78 7.76 7.75 7.74 7.73 7.72 7.71 7.70 7.68 5.18 5.06 5.04 5.03 3.69 3.67 3.65 3.64 3.63 1.99 1.97 1.95 1.93 1.91 1.89 1.87 1.78 1.74 1.73 1.72 1.71 1.68 1.67 1.57 1.54 1.54 1.50 1.48 1.48 1.46 1.46 1.45 1.45 1.43 1.43 1.41 1.34 1.33 1.32 1.31 1.31 1.30 1.29 1.16 1.14 1.14 1.13 1.12 1.12 1.11 1.11 1.11 1.10 0.85 0.84
1.00 1.02 0.95 0.92 1.13 3.29 2.02 1.01 2.11 14.95 0.90 1.23 2.89
9.5 9.0 8.5 8.0 7.5 7.0 6.5 6.0 5.5 5.0 4.5 4.0 3.5 3.0 2.5 2.0 1.5 1.0 0.5 0.0

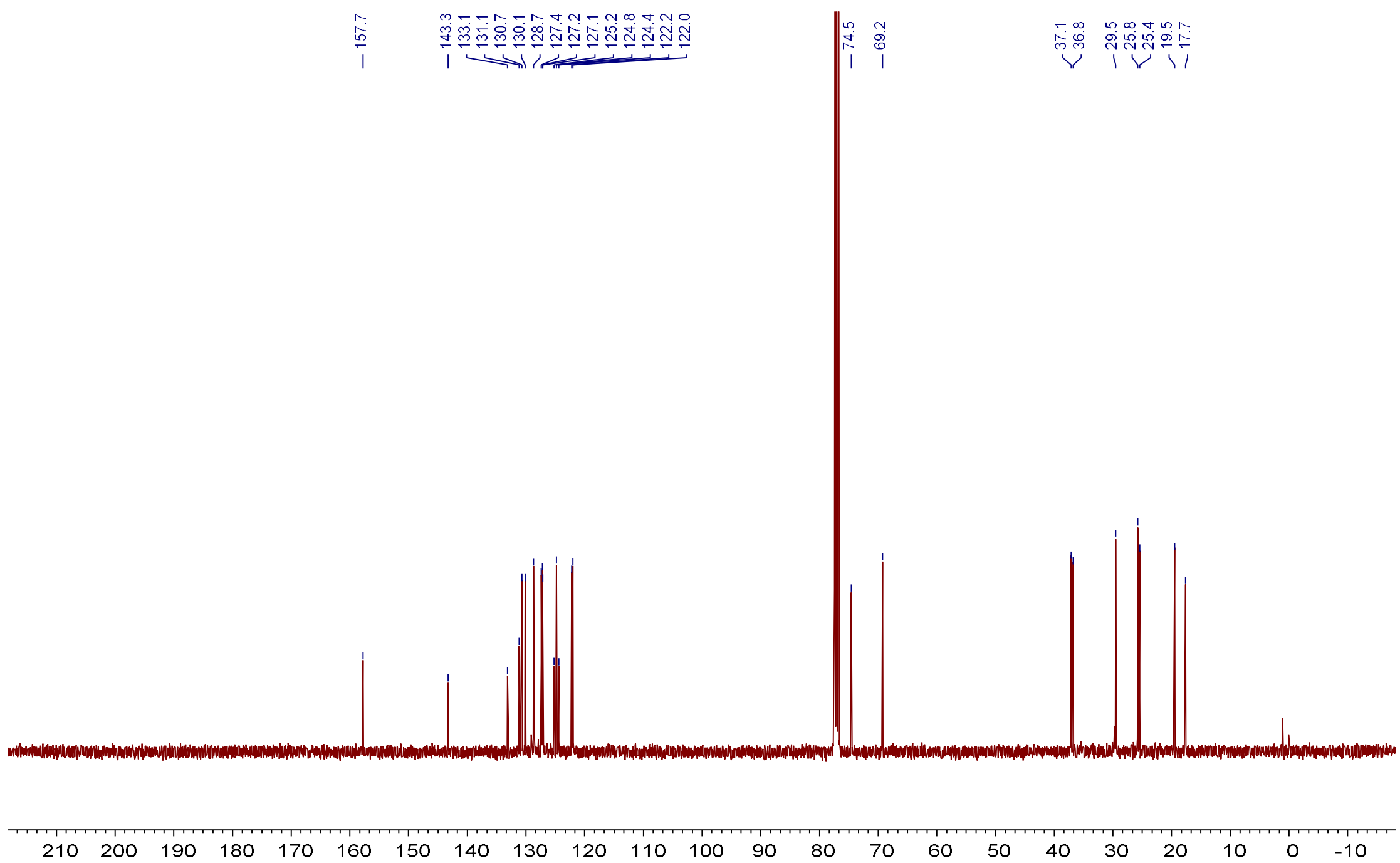
157.7
143.3 133.1 131.1 130.7 130.1 128.7 127.4 127.2 127.1 125.2 124.8 124.4 122.2 122.0
74.5 69.2
37.1 36.8 29.5 25.8 25.4 19.5 17.7
210 200 190 180 170 160 150 140 130 120 110 100 90 80 70 60 50 40 30 20 10 0 -10

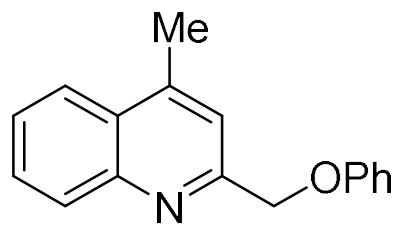
Me
N
OPh

7a

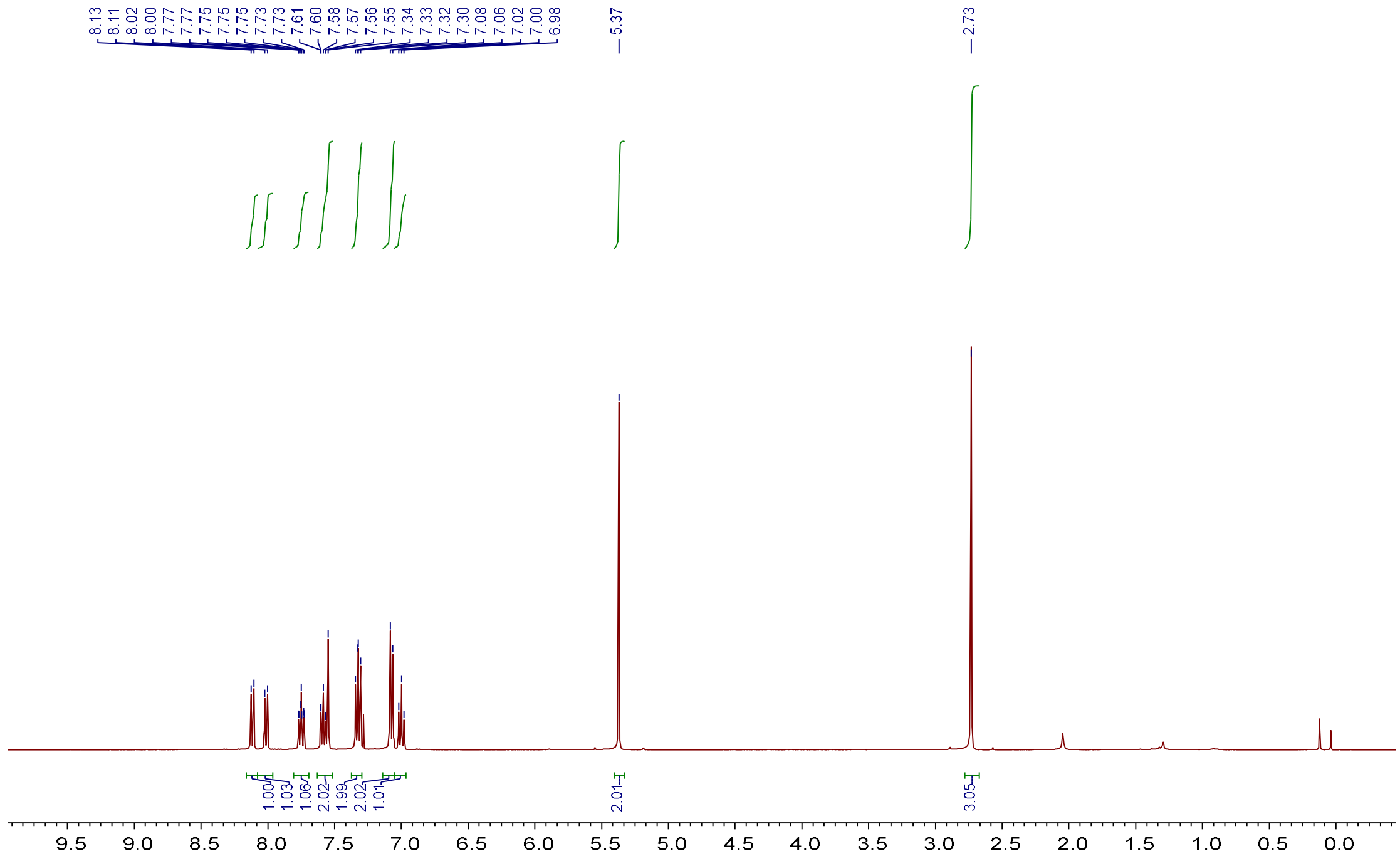
8.13
8.11
8.02
8.00
7.77
7.77
7.75
7.75
7.75
7.73
7.73
7.61
7.60
7.58
7.57
7.56
7.55
7.34
7.33
7.32
7.30
7.08
7.06
7.02
7.00
6.98
5.37
2.73
1.00
1.03
1.06
2.02
1.99
2.02
1.01
2.01
3.05
9.5
9.0
8.5
8.0
7.5
7.0
6.5
6.0
5.5
5.0
4.5
4.0
3.5
3.0
2.5
2.0
1.5
1.0
0.5
0.0

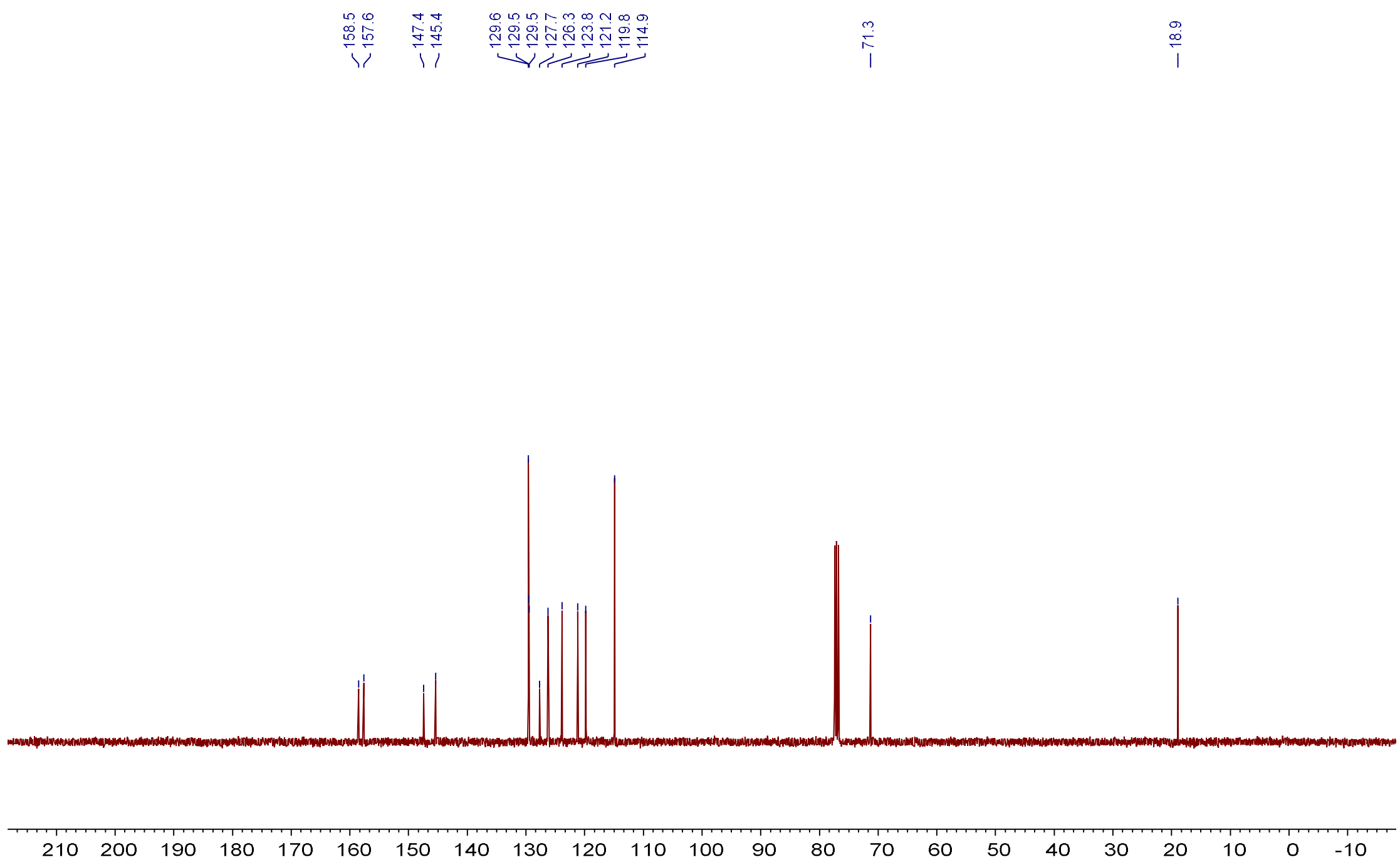
158.5
157.6
147.4
145.4
129.6
129.5
129.5
127.7
126.3
123.8
121.2
119.8
114.9
71.3
18.9
210
200
190
180
170
160
150
140
130
120
110
100
90
80
70
60
50
40
30
20
10
0
-10

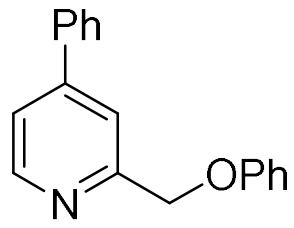

7b

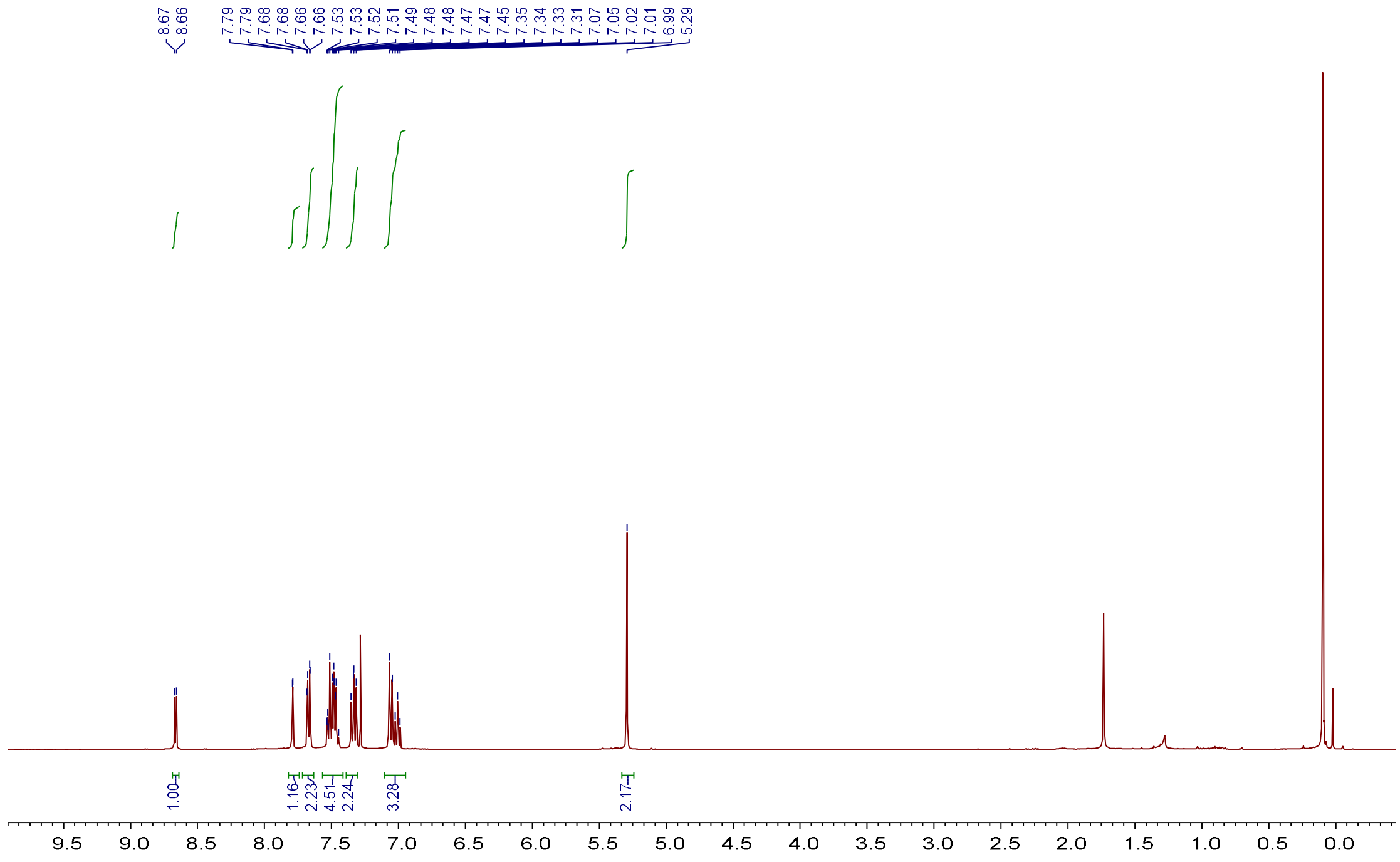

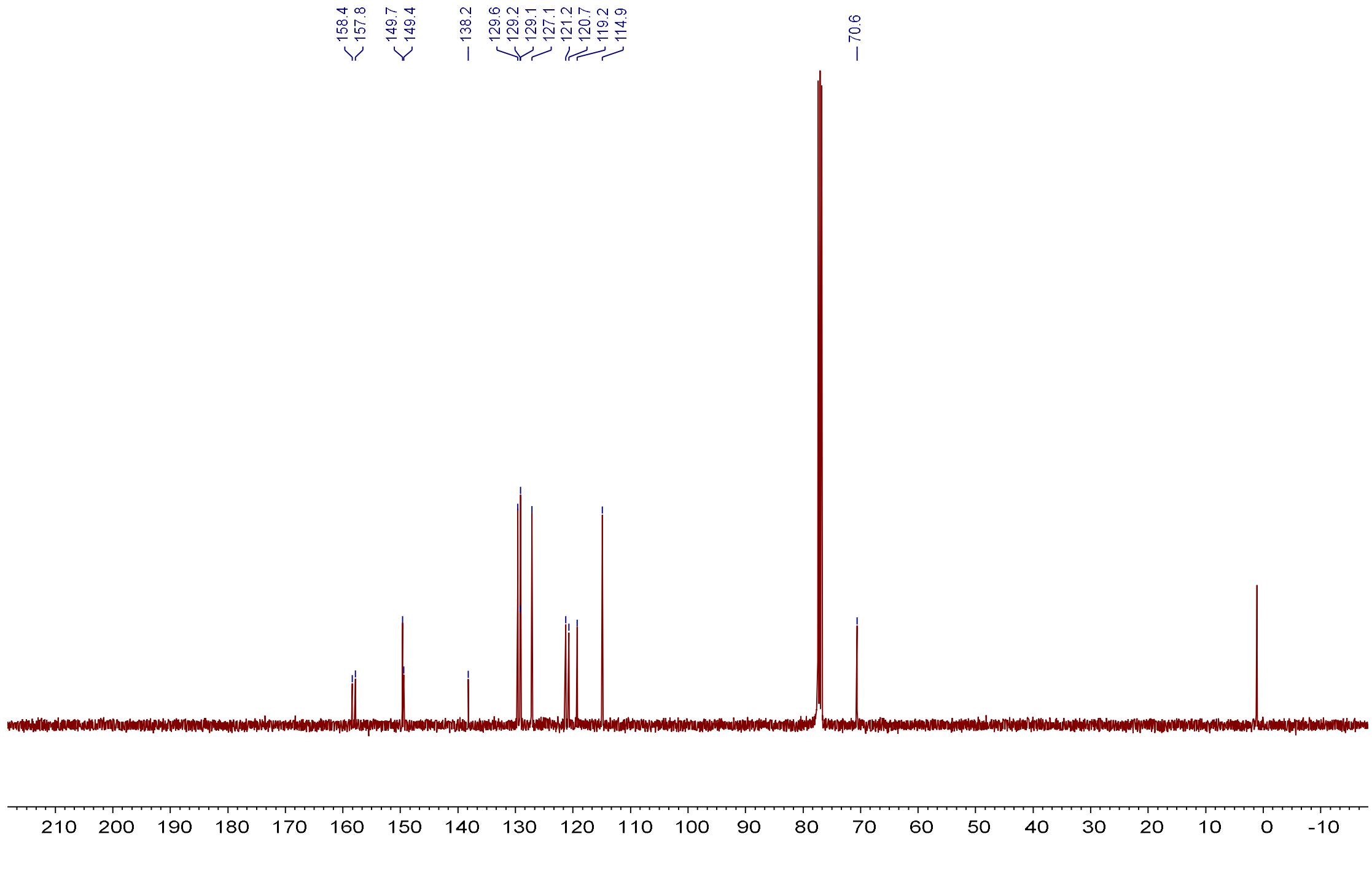

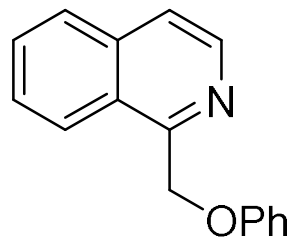
N
OPh

7c

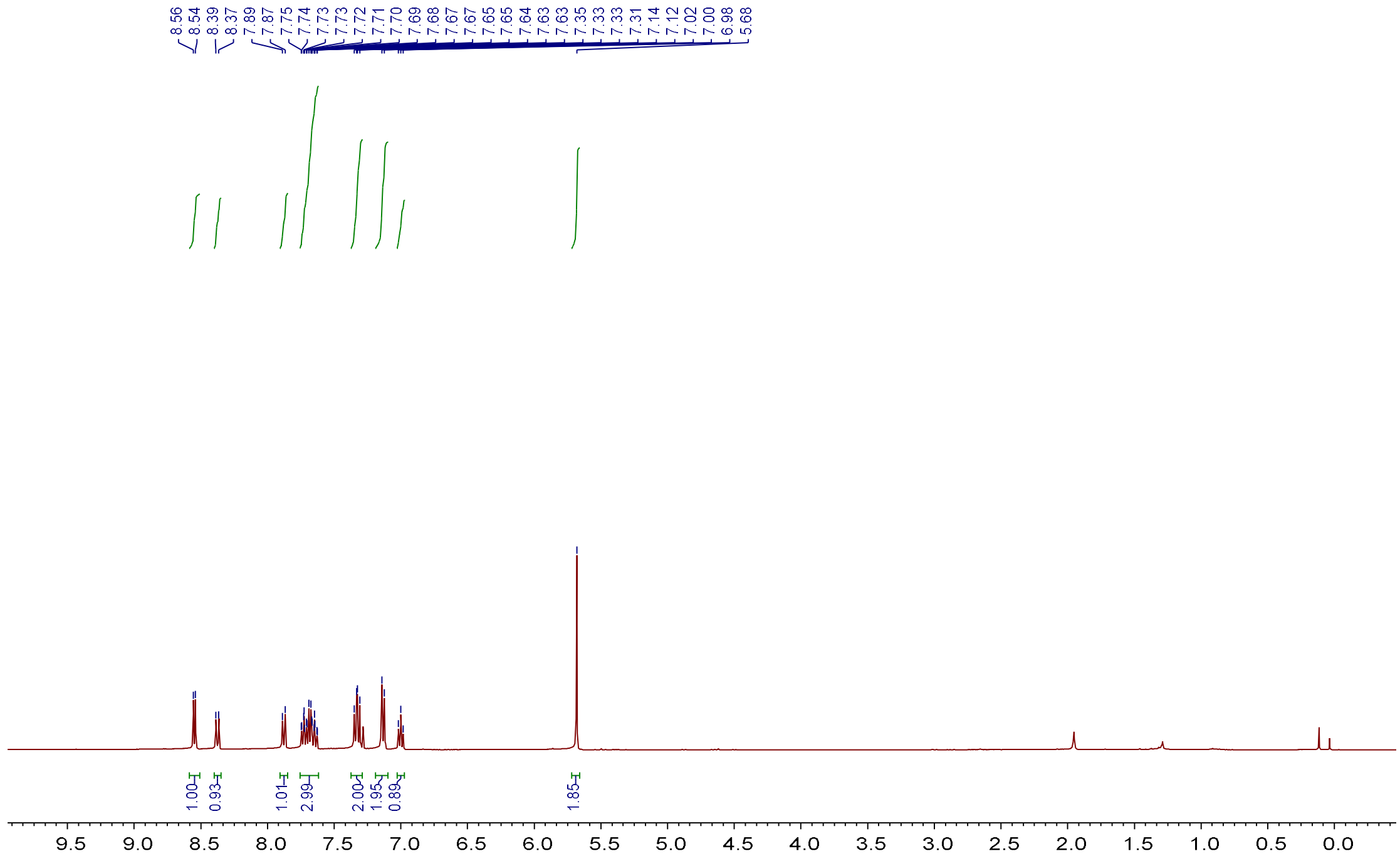
8.56 8.54 8.39 8.37 7.89 7.87 7.75 7.74 7.73 7.73 7.72 7.71 7.70 7.69 7.68 7.67 7.67 7.65 7.65 7.64 7.63 7.63 7.35 7.33 7.33 7.31 7.14 7.12 7.02 7.00 6.98 5.68
1.00 0.93 1.01 2.99 2.00 1.95 0.89 1.85
9.5 9.0 8.5 8.0 7.5 7.0 6.5 6.0 5.5 5.0 4.5 4.0 3.5 3.0 2.5 2.0 1.5 1.0 0.5 0.0

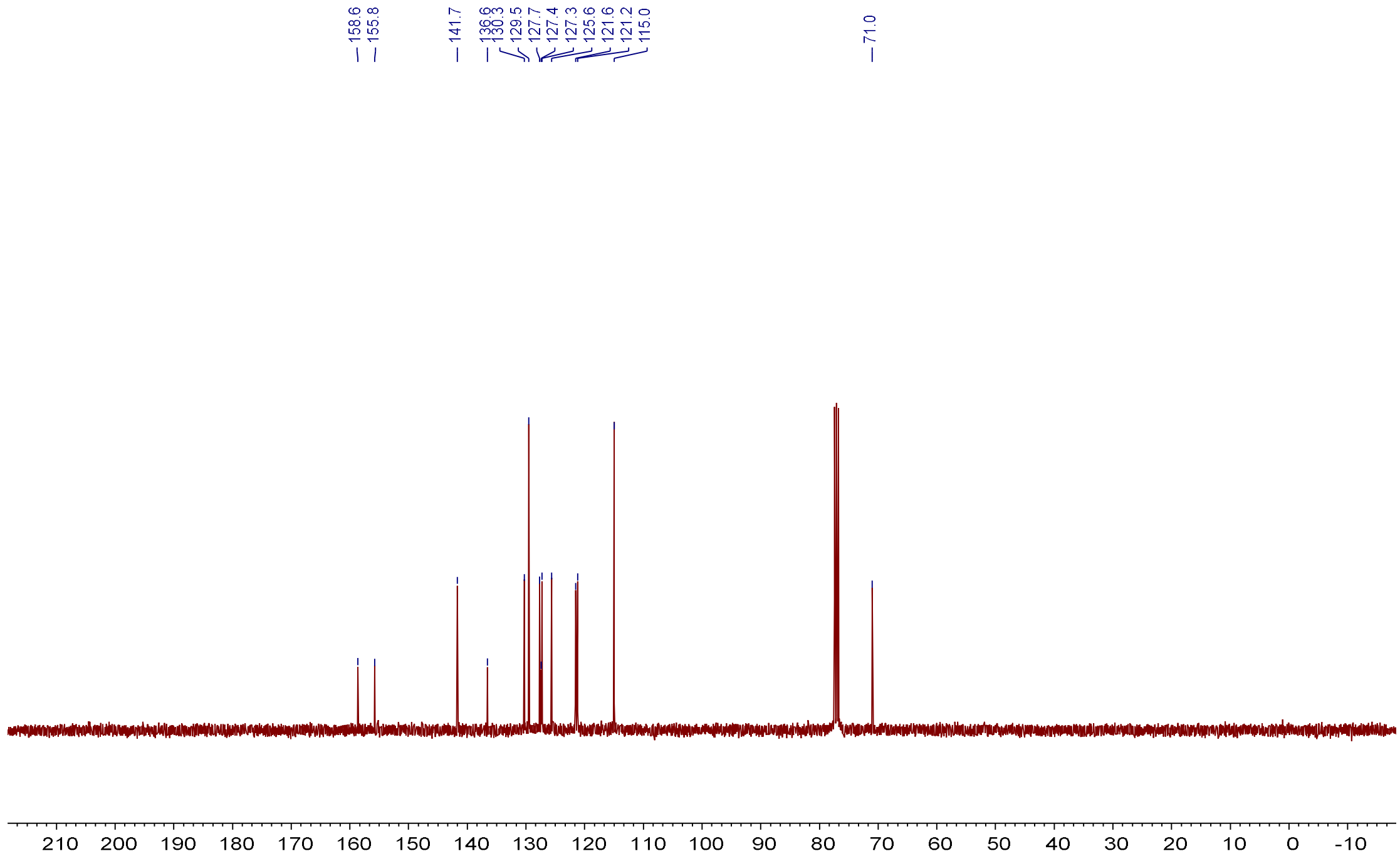
158.6 155.8 141.7 136.6 130.3 129.5 127.7 127.4 127.3 125.6 121.6 121.2 115.0 71.0
210 200 190 180 170 160 150 140 130 120 110 100 90 80 70 60 50 40 30 20 10 0 -10

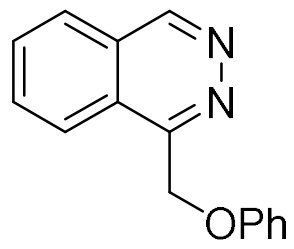
N
N
OPh

7d

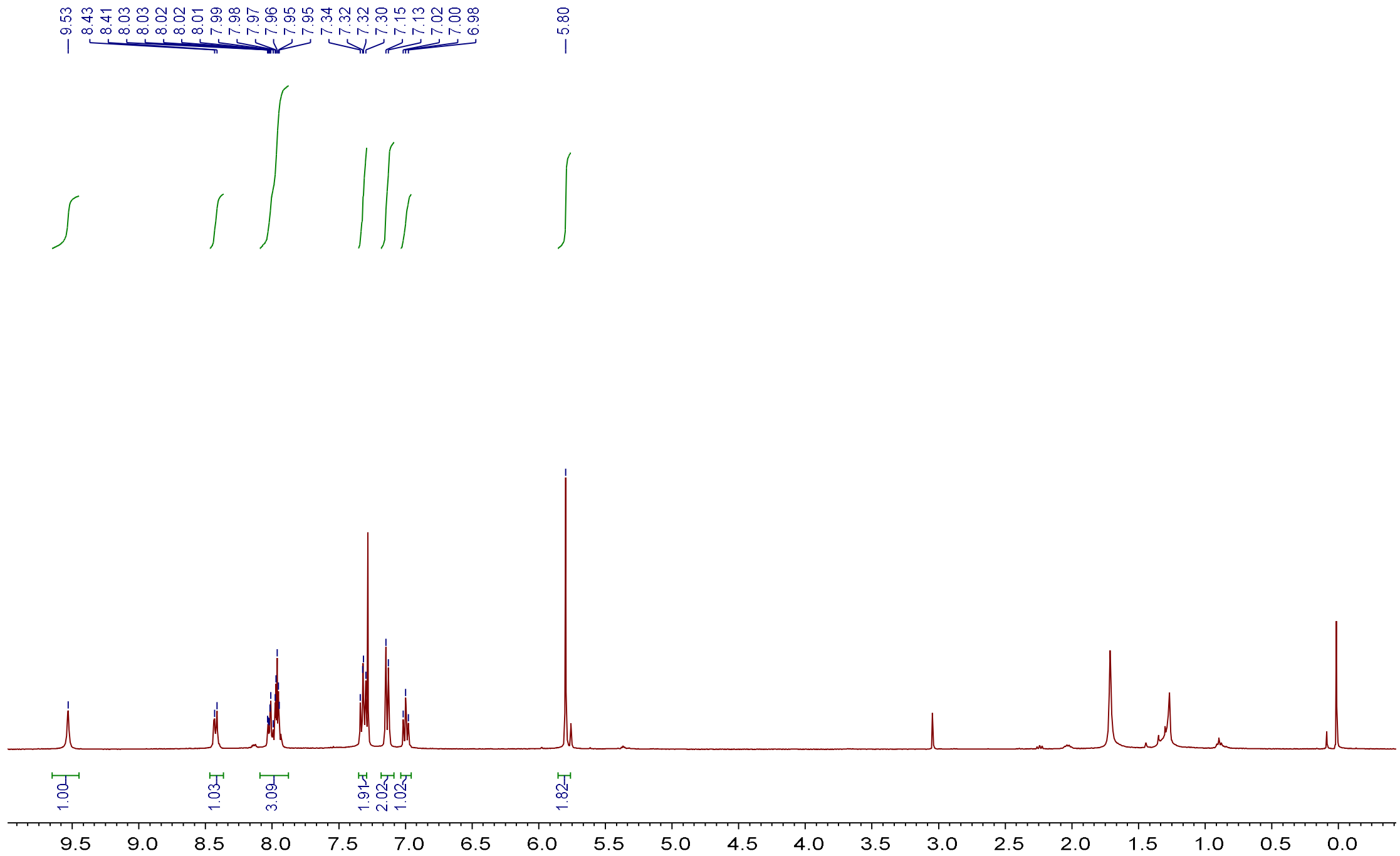
9.53
8.43
8.41
8.03
8.03
8.02
8.02
8.01
7.99
7.98
7.97
7.96
7.95
7.95
7.34
7.32
7.32
7.30
7.15
7.13
7.02
7.00
6.98
5.80
1.00
1.03
3.09
1.91
2.02
1.02
1.82
9.5 9.0 8.5 8.0 7.5 7.0 6.5 6.0 5.5 5.0 4.5 4.0 3.5 3.0 2.5 2.0 1.5 1.0 0.5 0.0

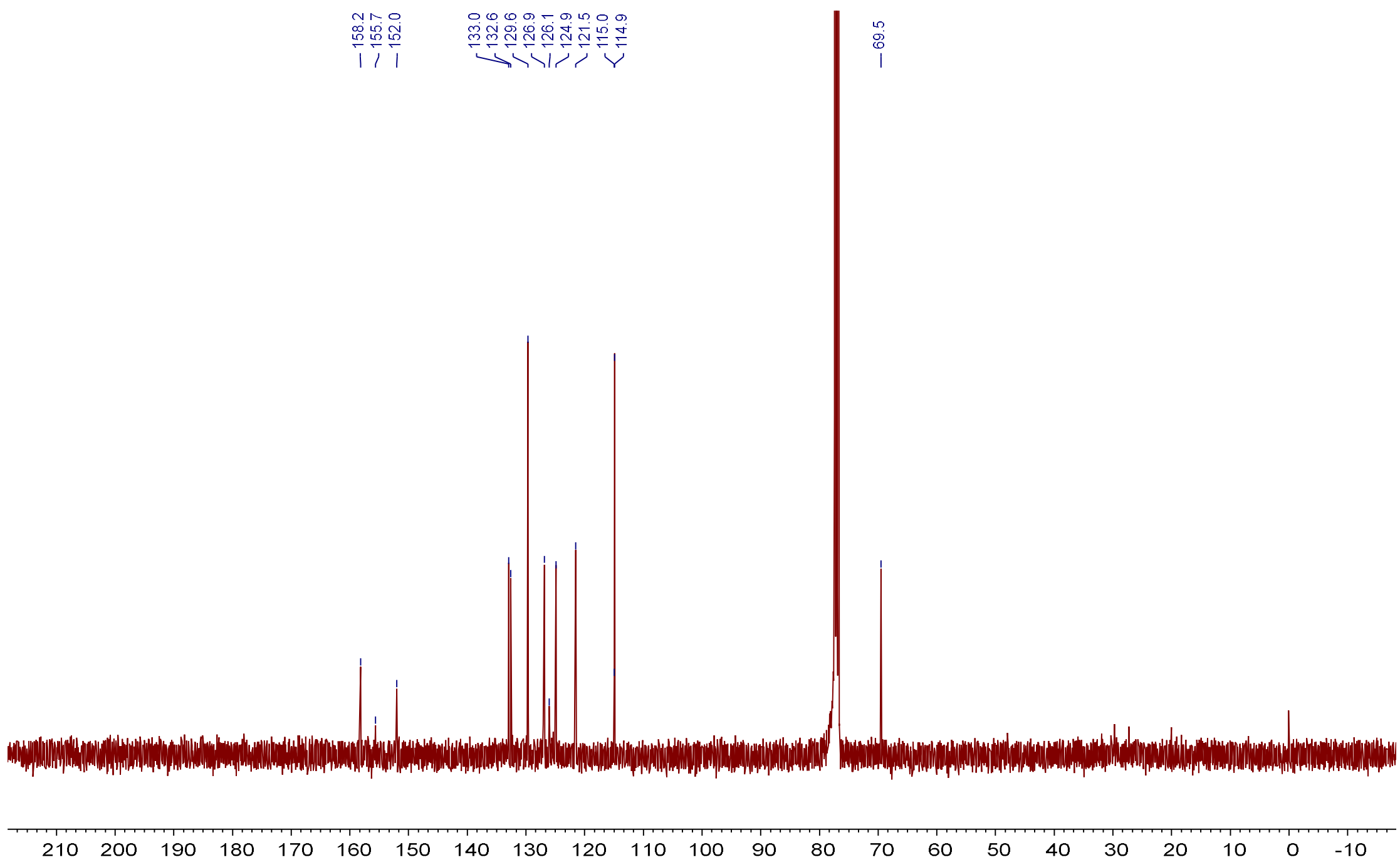
158.2
155.7
152.0
133.0
132.6
129.6
126.9
126.1
124.9
121.5
115.0
114.9
69.5
210 200 190 180 170 160 150 140 130 120 110 100 90 80 70 60 50 40 30 20 10 0 -10

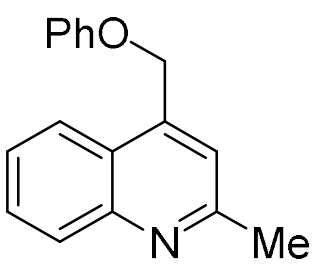
PhO
N
Me

7e

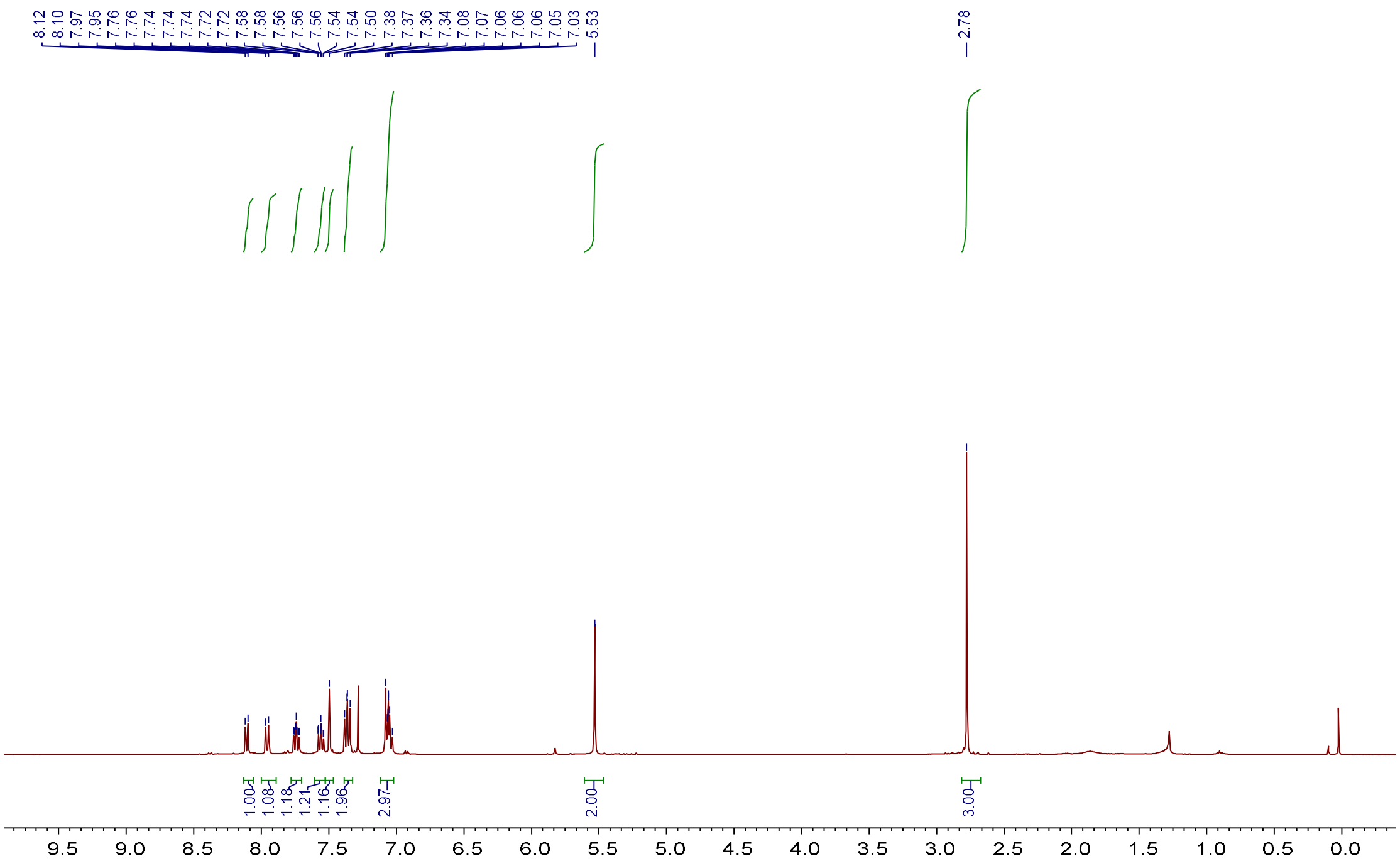
8.12
8.10
7.97
7.95
7.76
7.76
7.74
7.74
7.74
7.72
7.72
7.58
7.58
7.56
7.56
7.56
7.54
7.54
7.50
7.38
7.37
7.36
7.34
7.08
7.07
7.06
7.06
7.06
7.05
7.03
5.53
2.78
1.00
1.08
1.18
1.21
1.16
1.96
2.97
2.00
3.00
9.5 9.0 8.5 8.0 7.5 7.0 6.5 6.0 5.5 5.0 4.5 4.0 3.5 3.0 2.5 2.0 1.5 1.0 0.5 0.0

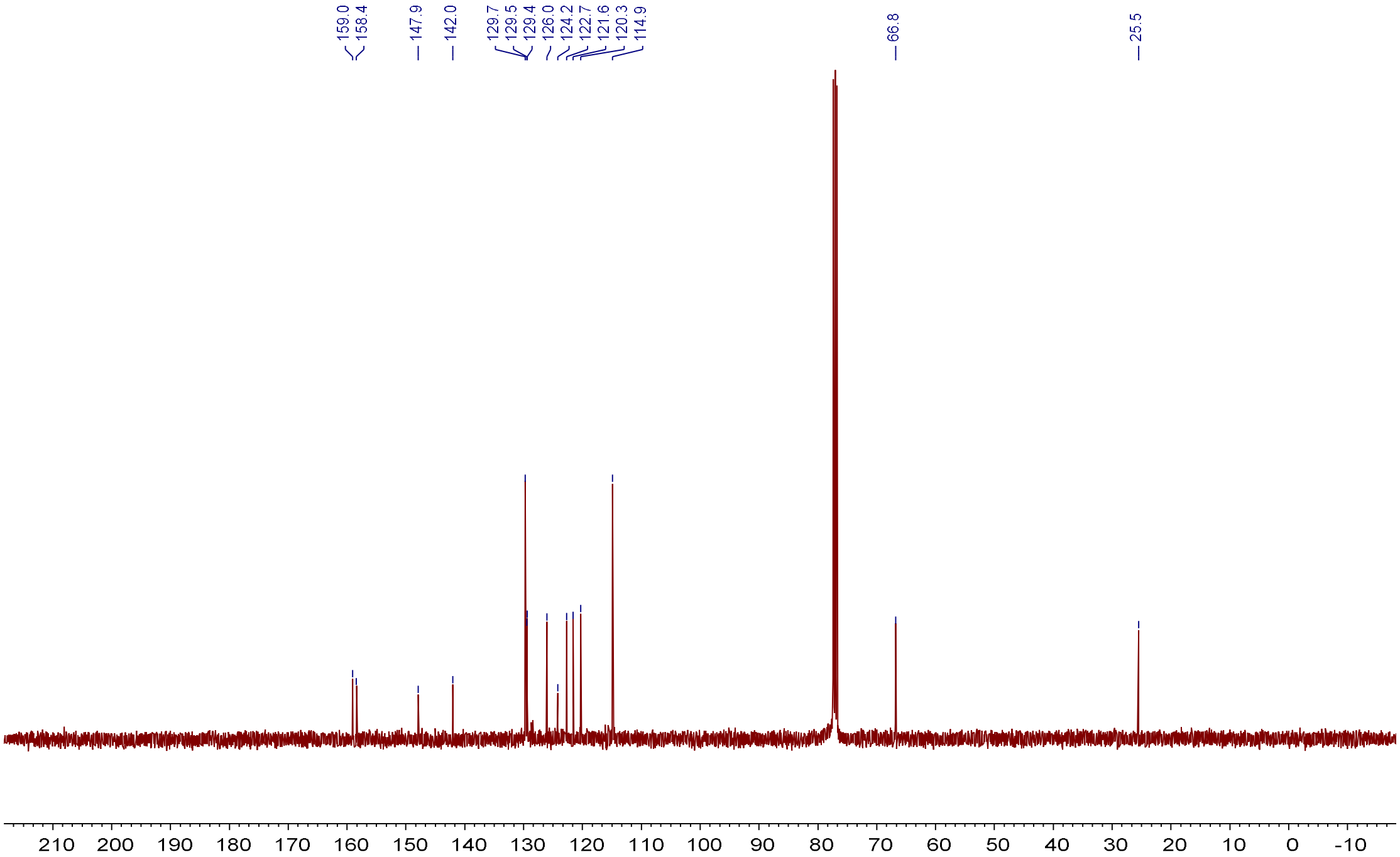
159.0
158.4
147.9
142.0
129.7
129.5
129.4
126.0
124.2
122.7
121.6
120.3
114.9
66.8
25.5
210 200 190 180 170 160 150 140 130 120 110 100 90 80 70 60 50 40 30 20 10 0 -10

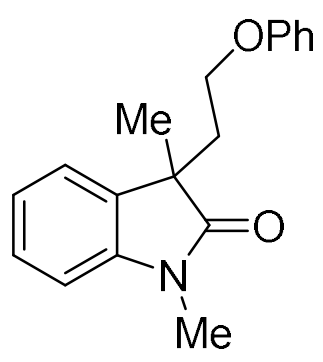

7f

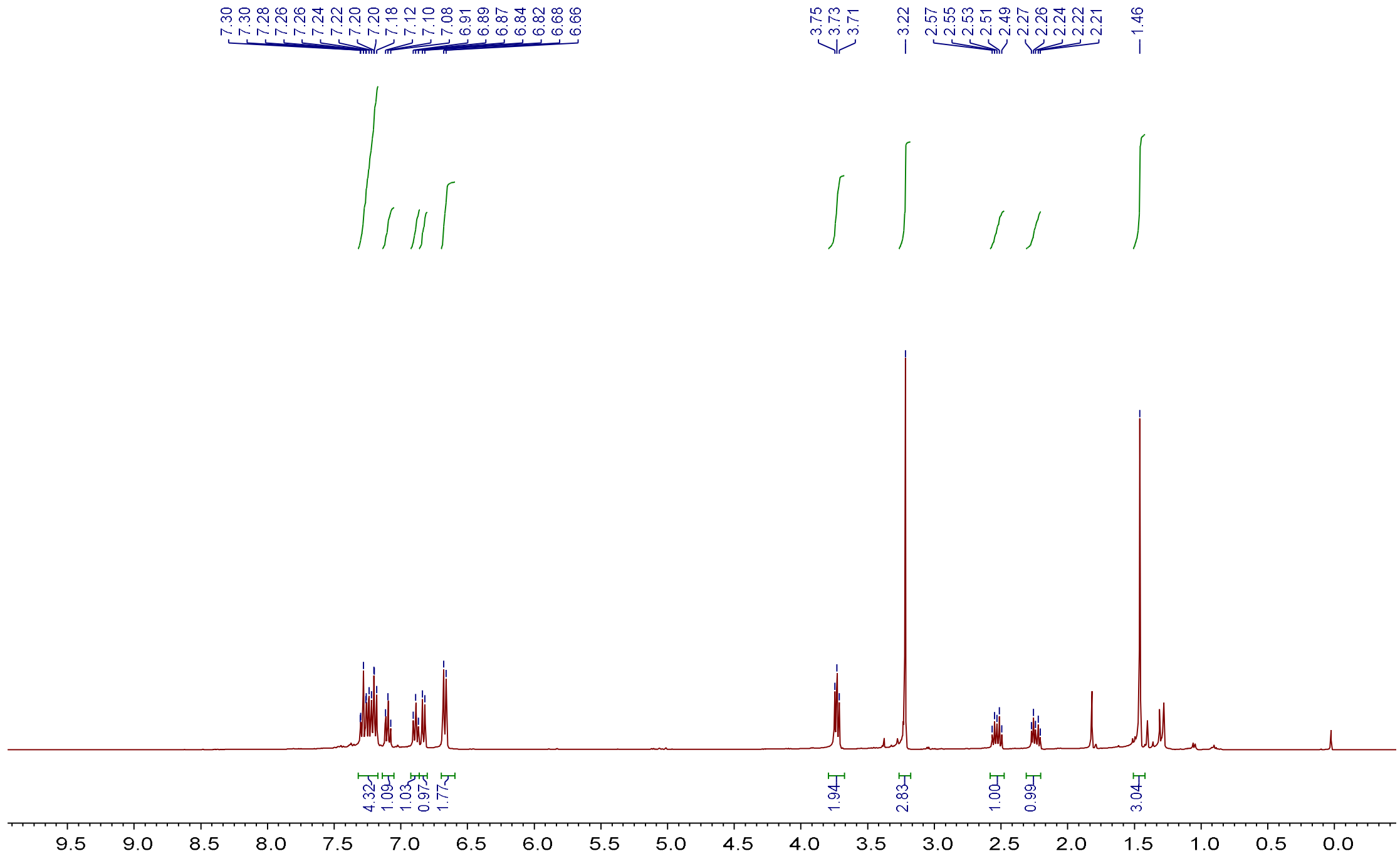

7.30 7.30 7.28 7.26 7.26 7.24 7.22 7.22 7.20 7.20 7.18 7.12 7.10 7.08 6.91 6.89 6.87 6.84 6.82 6.68 6.66
3.75 3.73 3.71
3.22
2.57 2.55 2.53 2.51 2.49 2.27 2.26 2.24 2.22 2.21
1.46
4.32 1.09 1.03 0.97 1.77
1.94
2.83
1.00
0.99
3.04
9.5 9.0 8.5 8.0 7.5 7.0 6.5 6.0 5.5 5.0 4.5 4.0 3.5 3.0 2.5 2.0 1.5 1.0 0.5 0.0

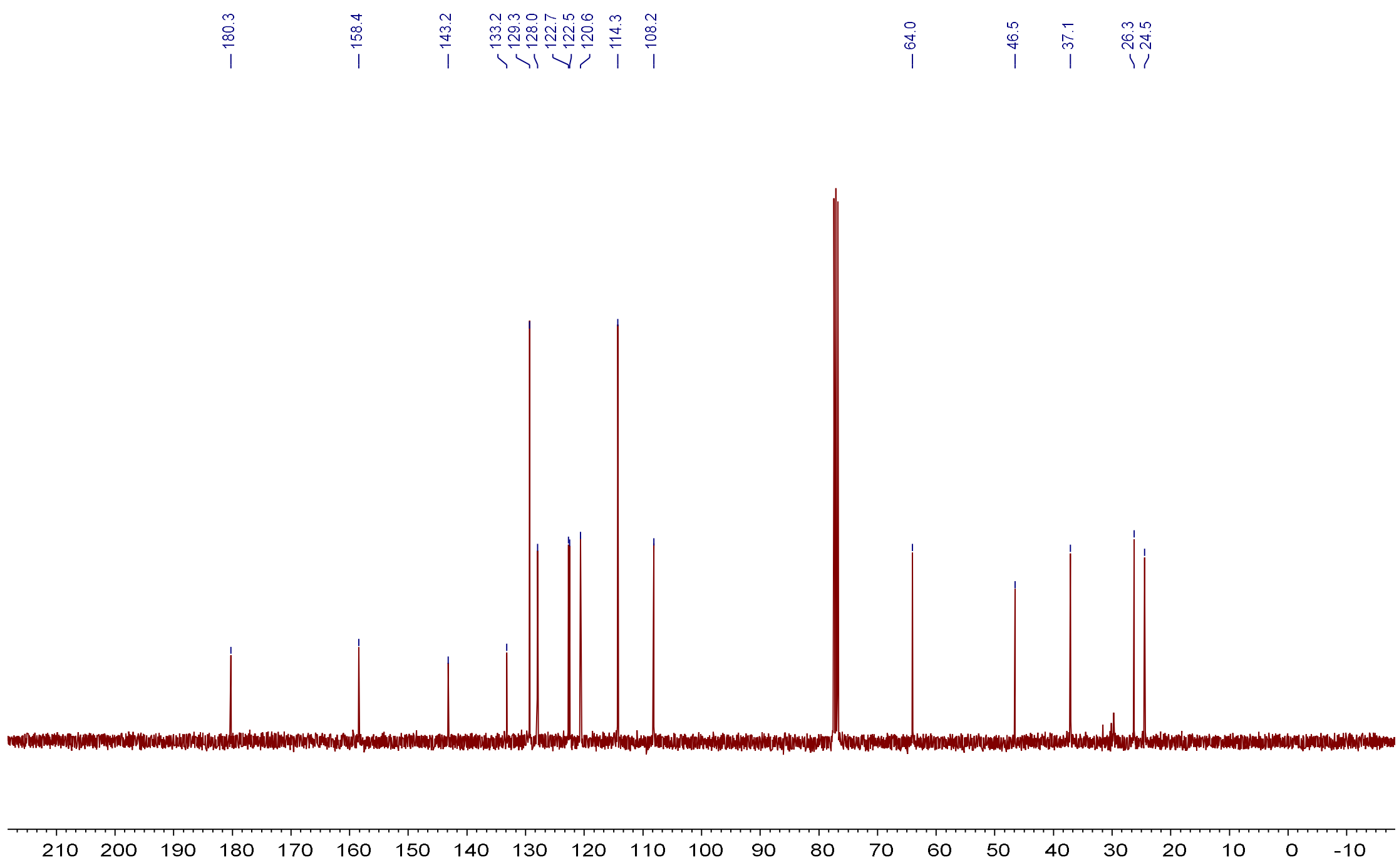

180.3
158.4
143.2
133.2 129.3 128.0 122.7 122.5 120.6
114.3
108.2
64.0
46.5
37.1
26.3 24.5
210 200 190 180 170 160 150 140 130 120 110 100 90 80 70 60 50 40 30 20 10 0 -10

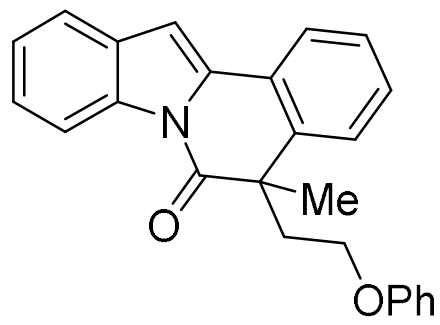
N
Me
O
OPh

7g

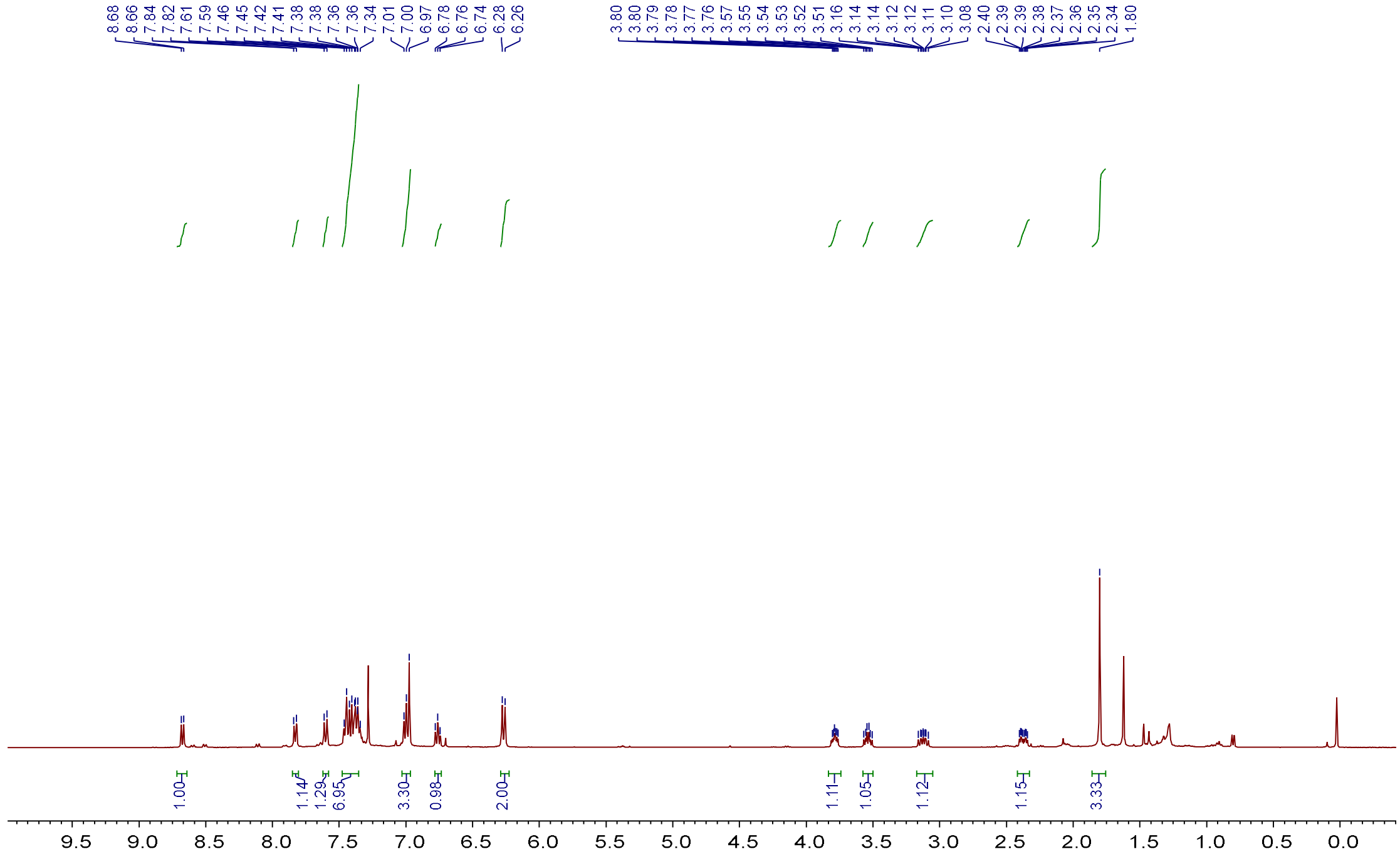
8.68 8.66 7.84 7.82 7.61 7.59 7.46 7.45 7.42 7.41 7.38 7.38 7.36 7.36 7.34 7.01 7.00 6.97 6.78 6.76 6.74 6.28 6.26
3.80 3.80 3.79 3.78 3.77 3.76 3.57 3.55 3.54 3.53 3.52 3.51 3.16 3.14 3.14 3.12 3.12 3.11 3.10 3.08 2.40 2.39 2.39 2.38 2.37 2.36 2.35 2.34 1.80
1.00 1.14 1.29 6.95 3.30 0.98 2.00 1.11 1.05 1.12 1.15 3.33
9.5 9.0 8.5 8.0 7.5 7.0 6.5 6.0 5.5 5.0 4.5 4.0 3.5 3.0 2.5 2.0 1.5 1.0 0.5 0.0

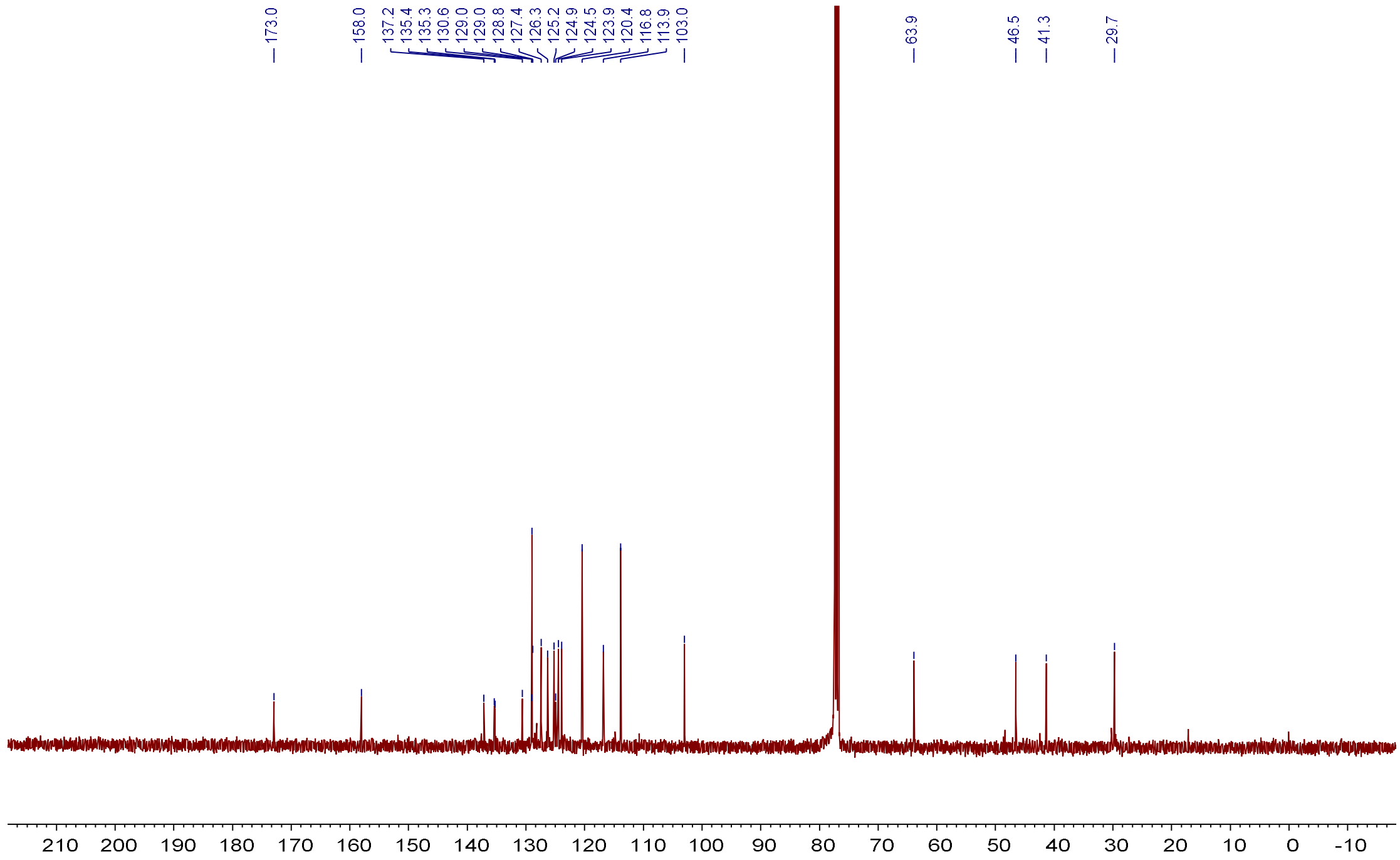
173.0
158.0
137.2 135.4 135.3 130.6 129.0 129.0 128.8 127.4 126.3 125.2 124.9 124.5 123.9 120.4 116.8 113.9 103.0
63.9
46.5 41.3
29.7
210 200 190 180 170 160 150 140 130 120 110 100 90 80 70 60 50 40 30 20 10 0 -10

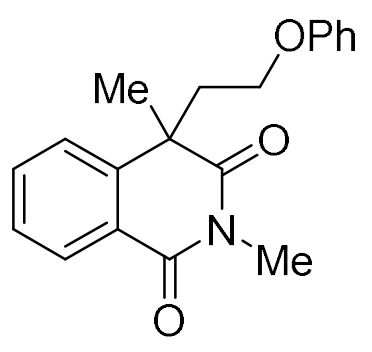

7h

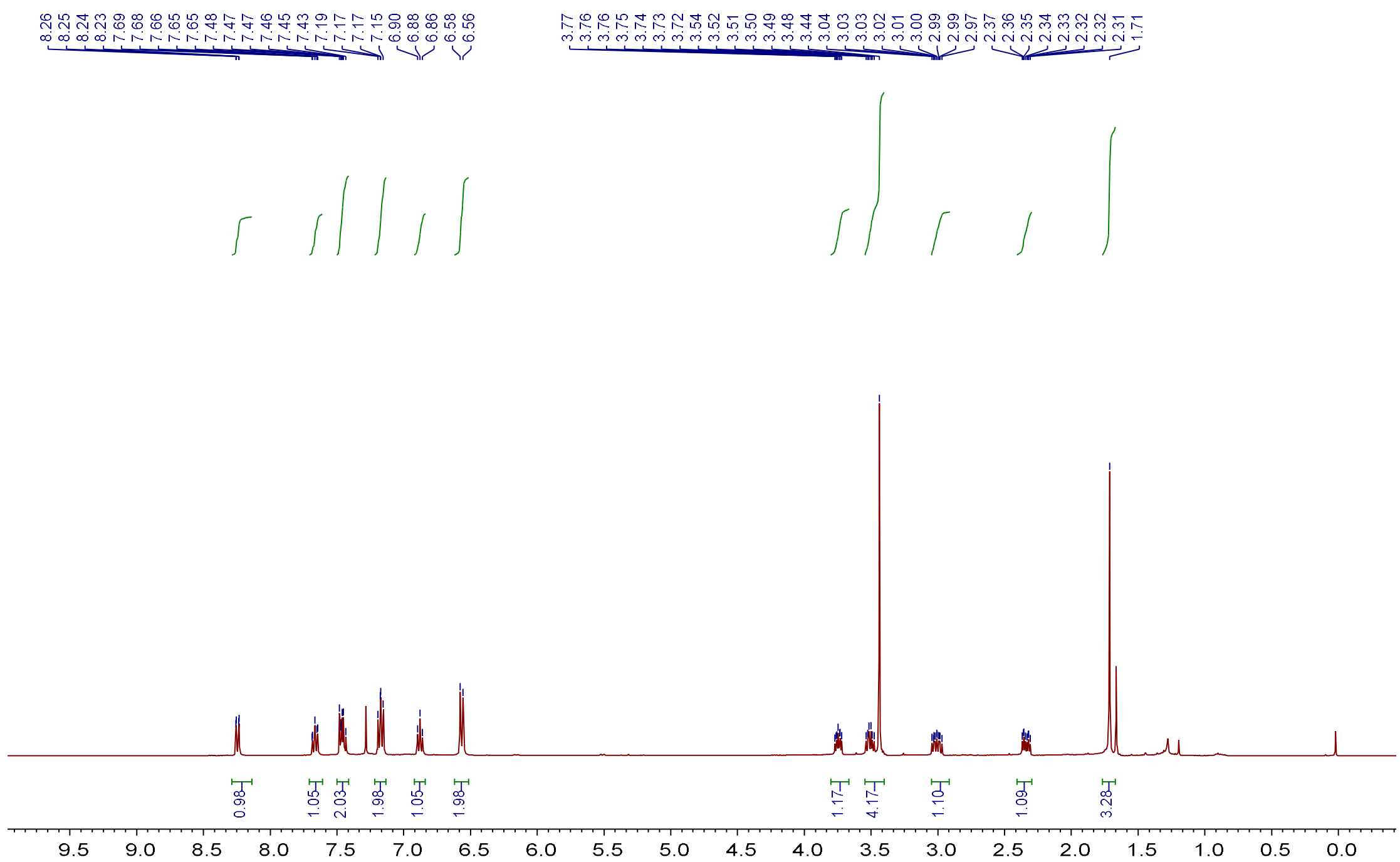

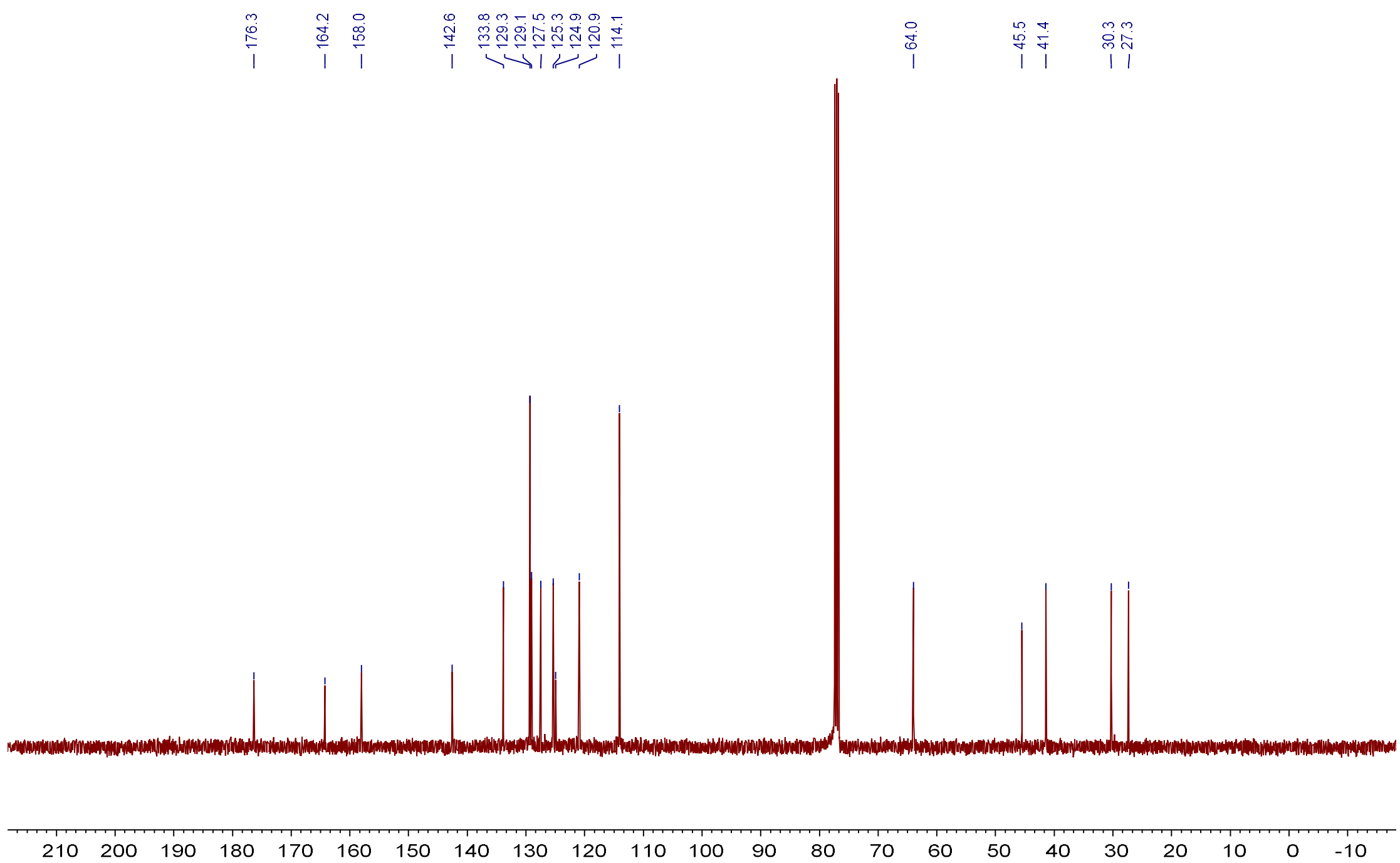

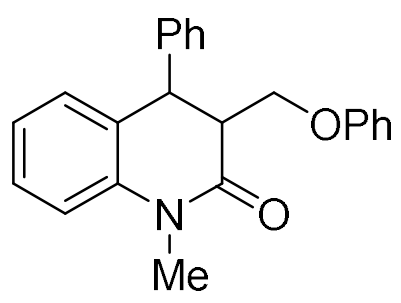

7i

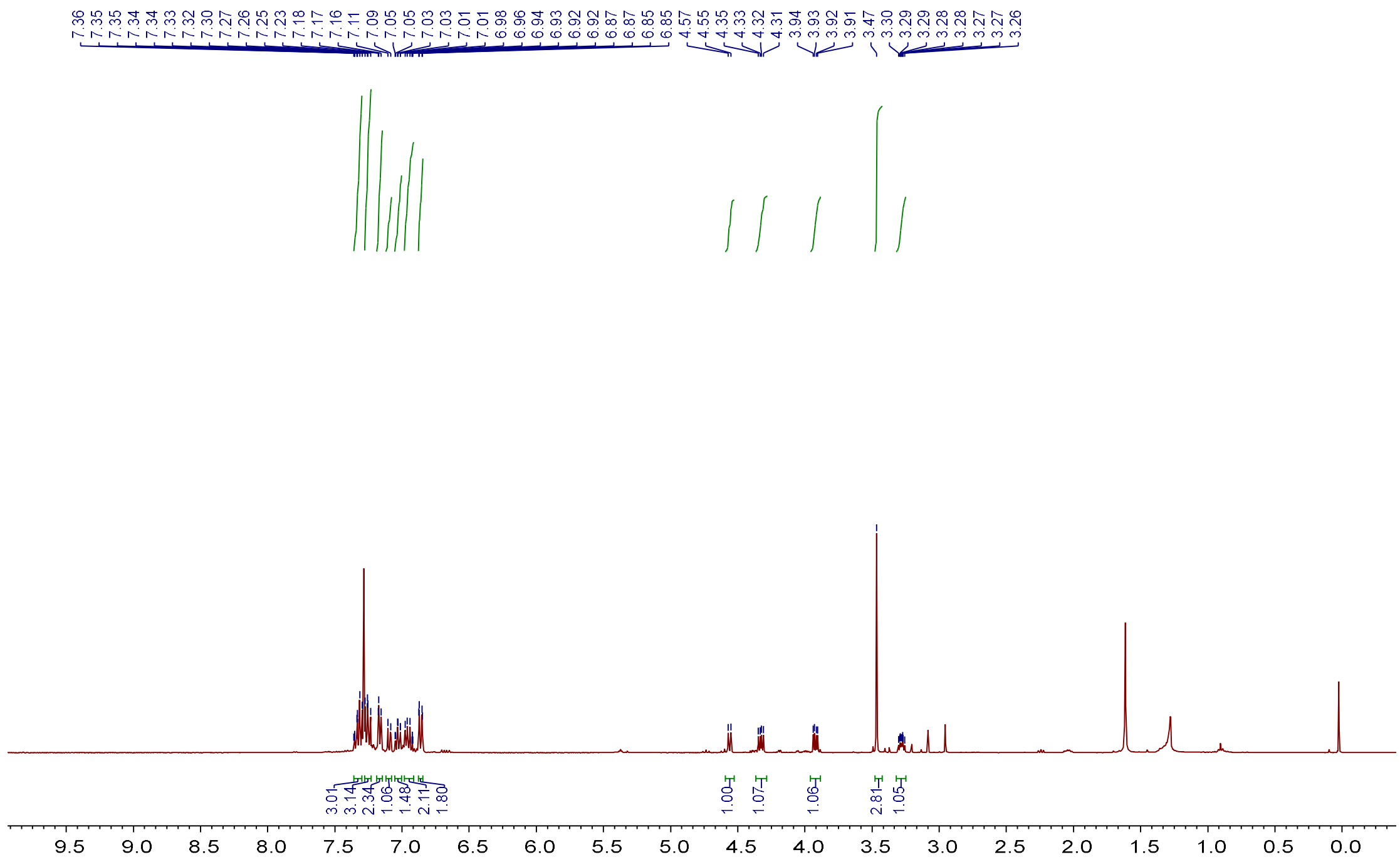

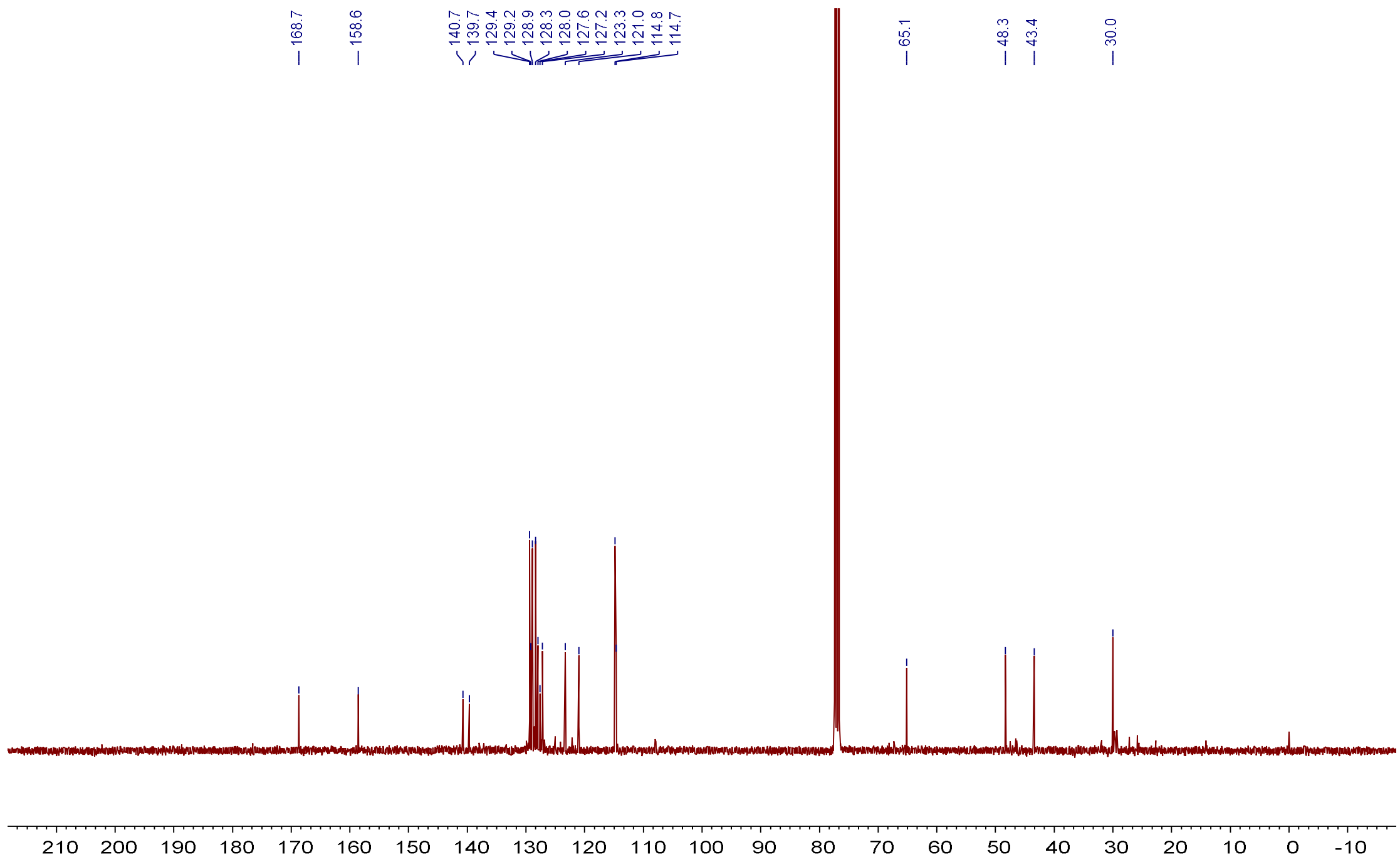

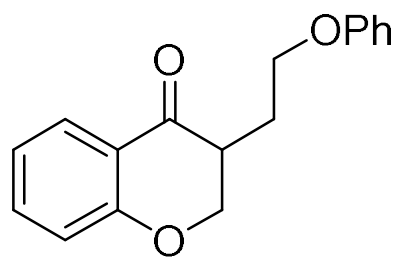

7j

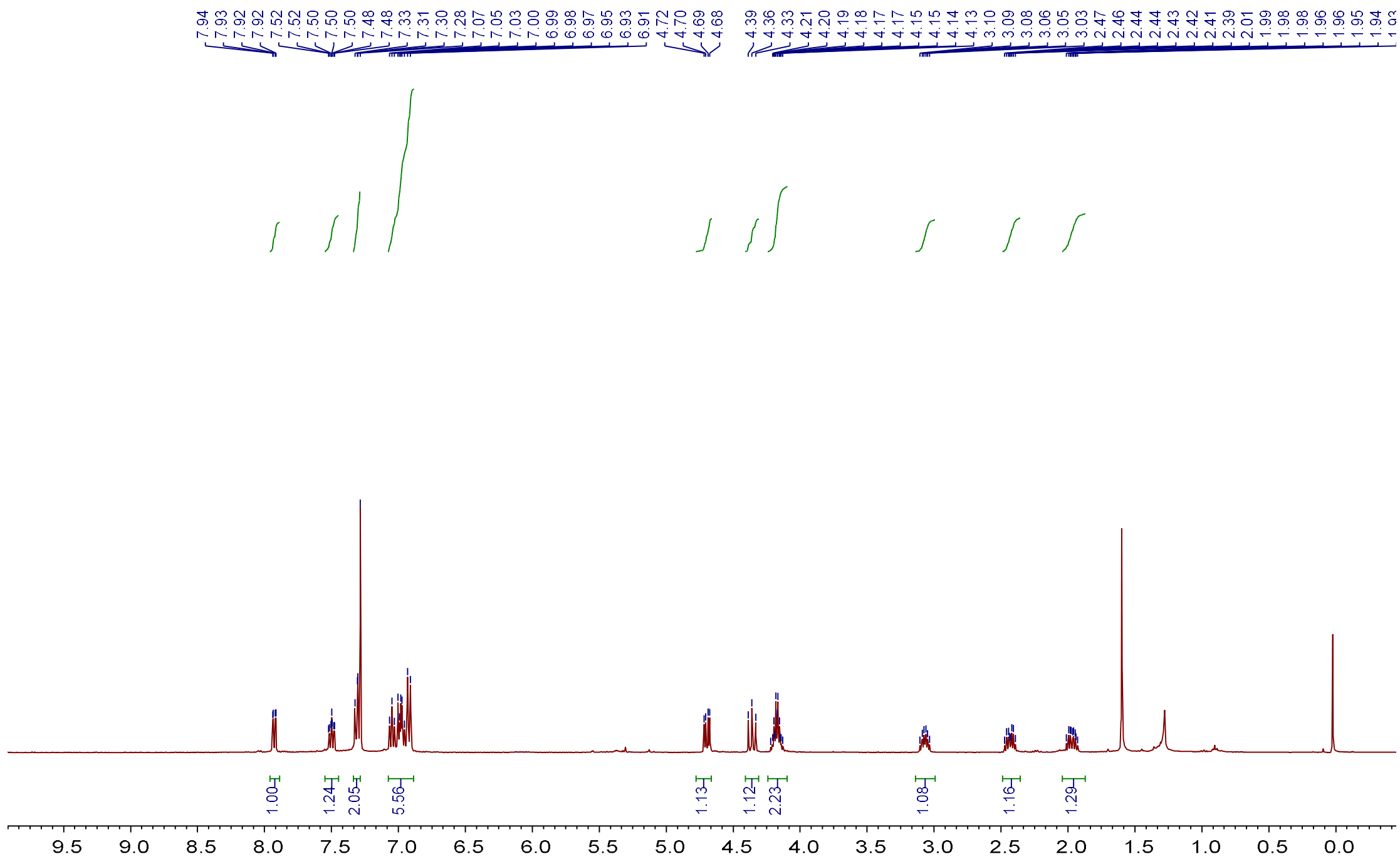

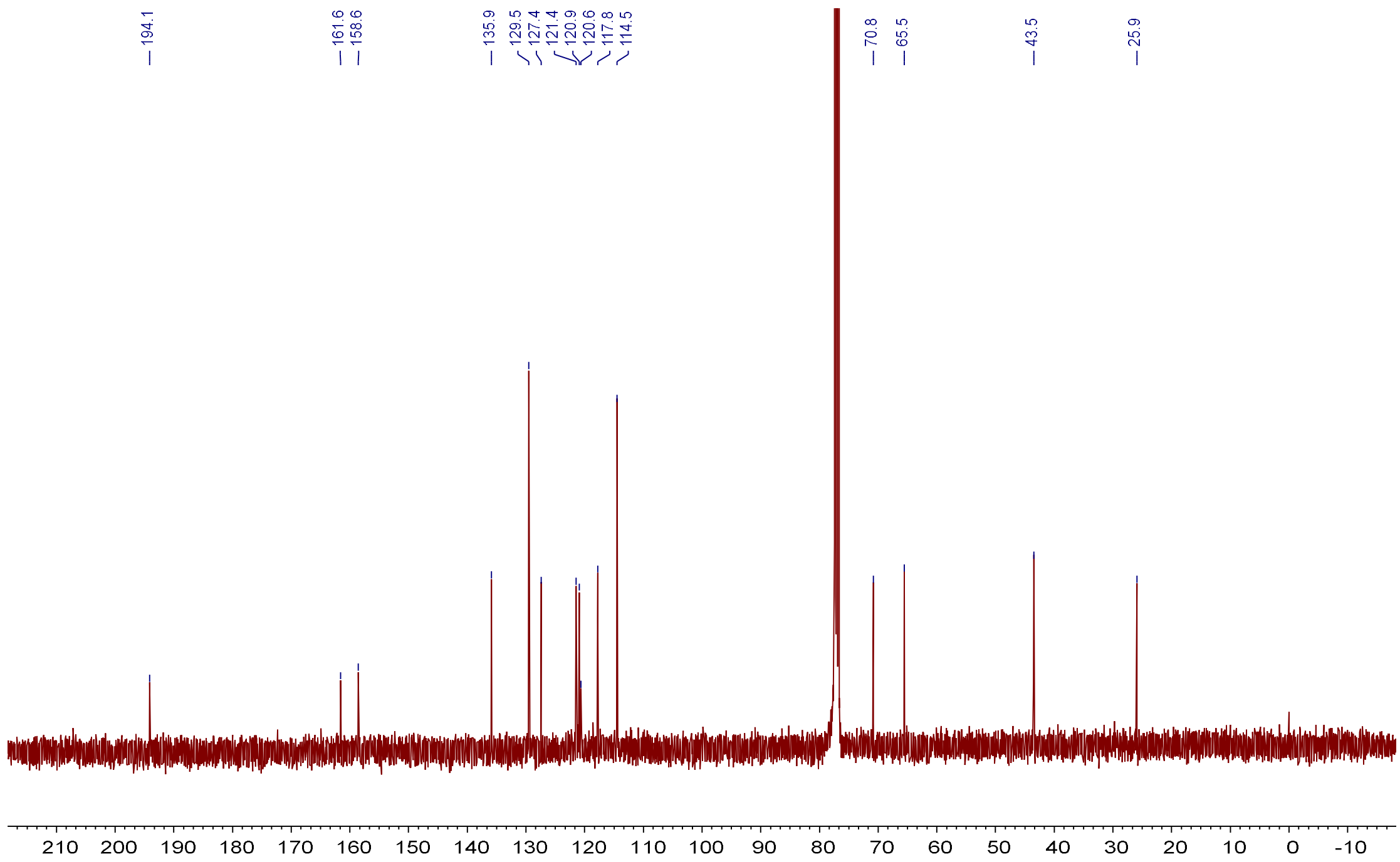

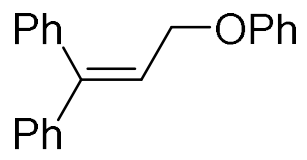
Ph
OPh
Ph

7k

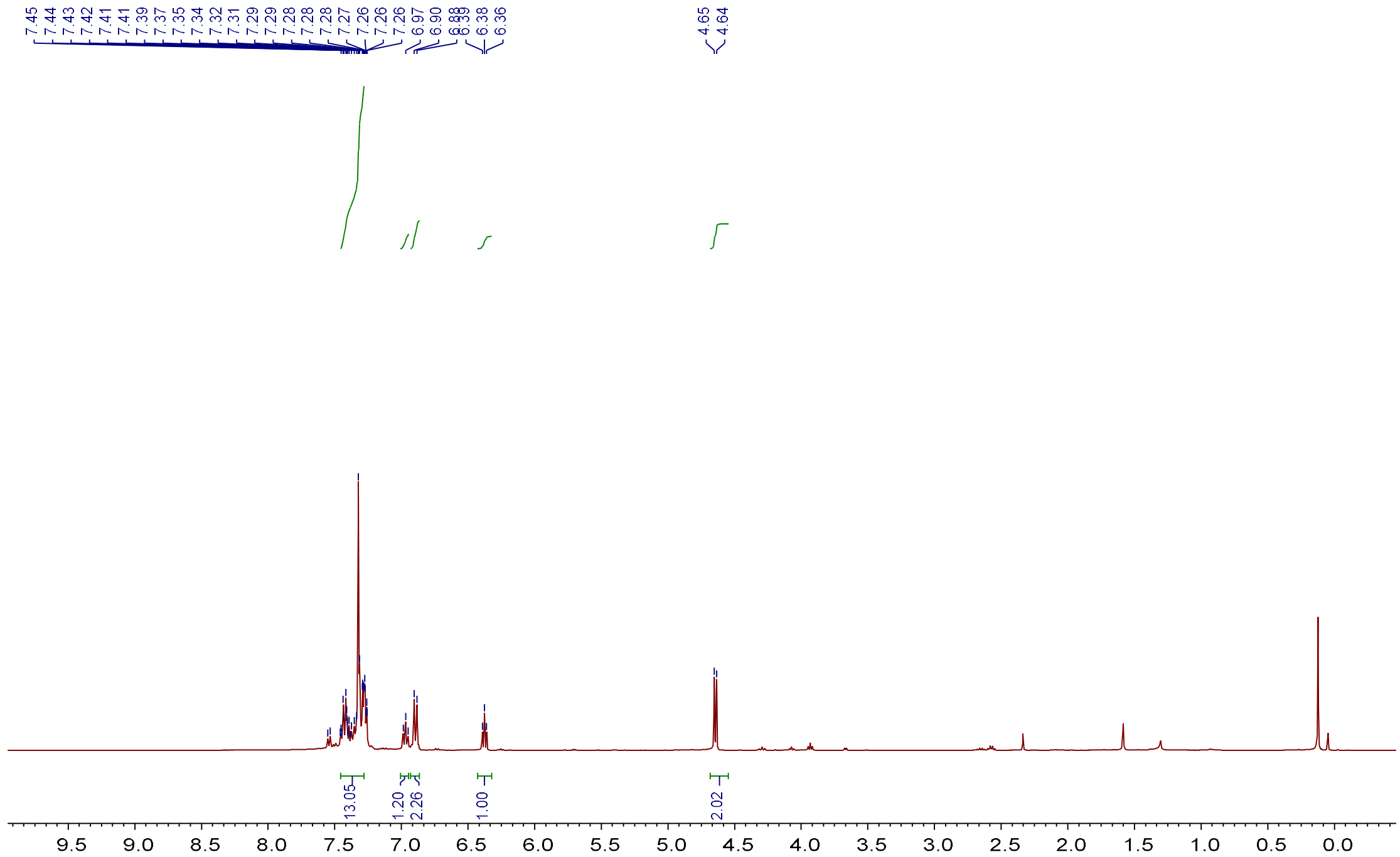
7.45
7.44
7.43
7.42
7.41
7.41
7.39
7.37
7.35
7.34
7.32
7.31
7.29
7.29
7.28
7.28
7.28
7.28
7.27
7.26
7.26
7.26
6.97
6.90
6.88
6.89
6.38
6.36
4.65
4.64
13.05
1.20
2.26
1.00
2.02
9.5 9.0 8.5 8.0 7.5 7.0 6.5 6.0 5.5 5.0 4.5 4.0 3.5 3.0 2.5 2.0 1.5 1.0 0.5 0.0

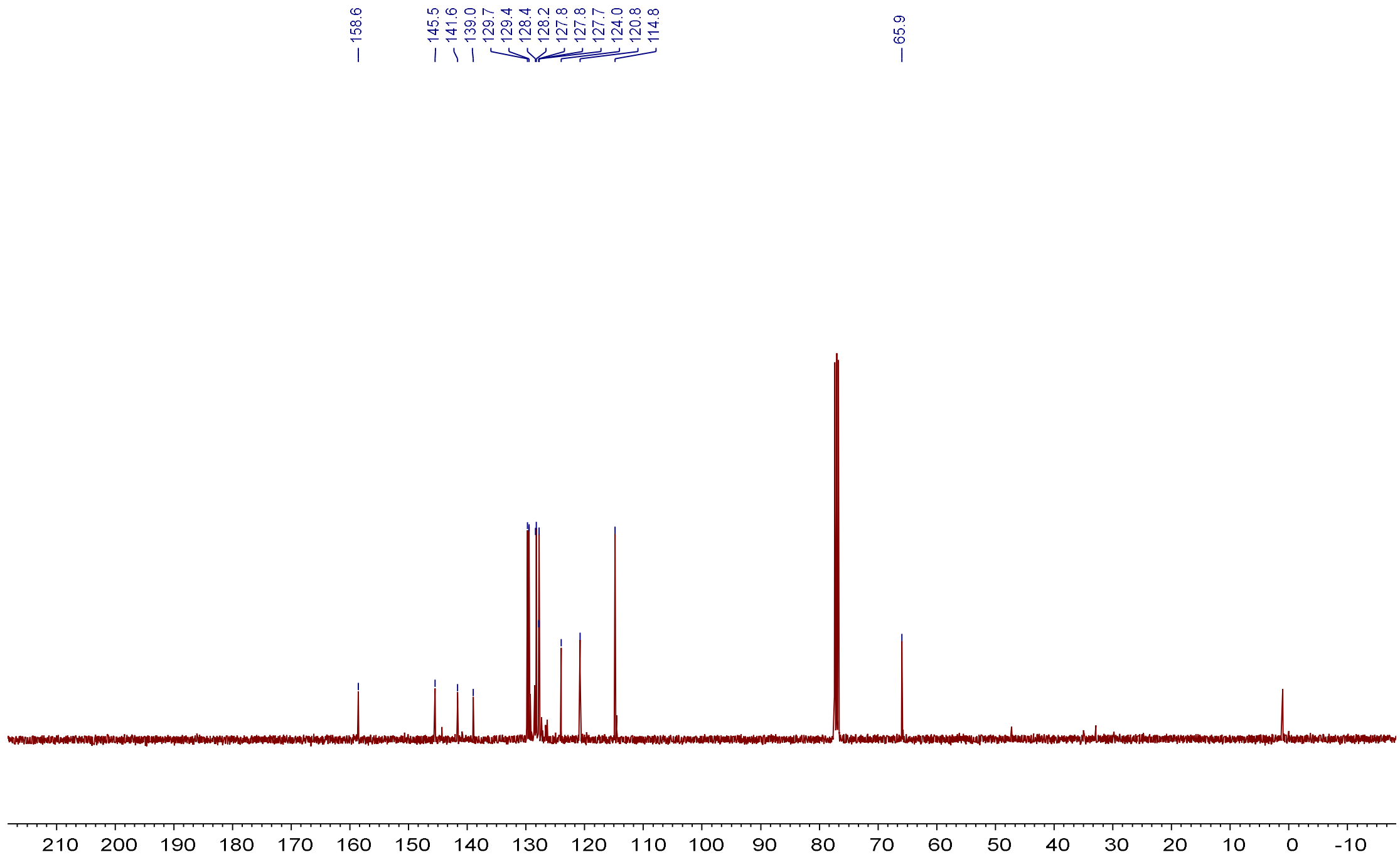
158.6
145.5
141.6
139.0
129.7
129.4
128.4
128.2
127.8
127.8
127.7
124.0
120.8
114.8
65.9
210 200 190 180 170 160 150 140 130 120 110 100 90 80 70 60 50 40 30 20 10 0 -10

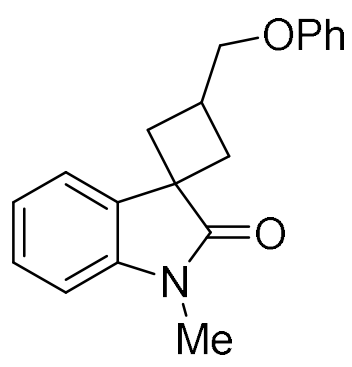

7l

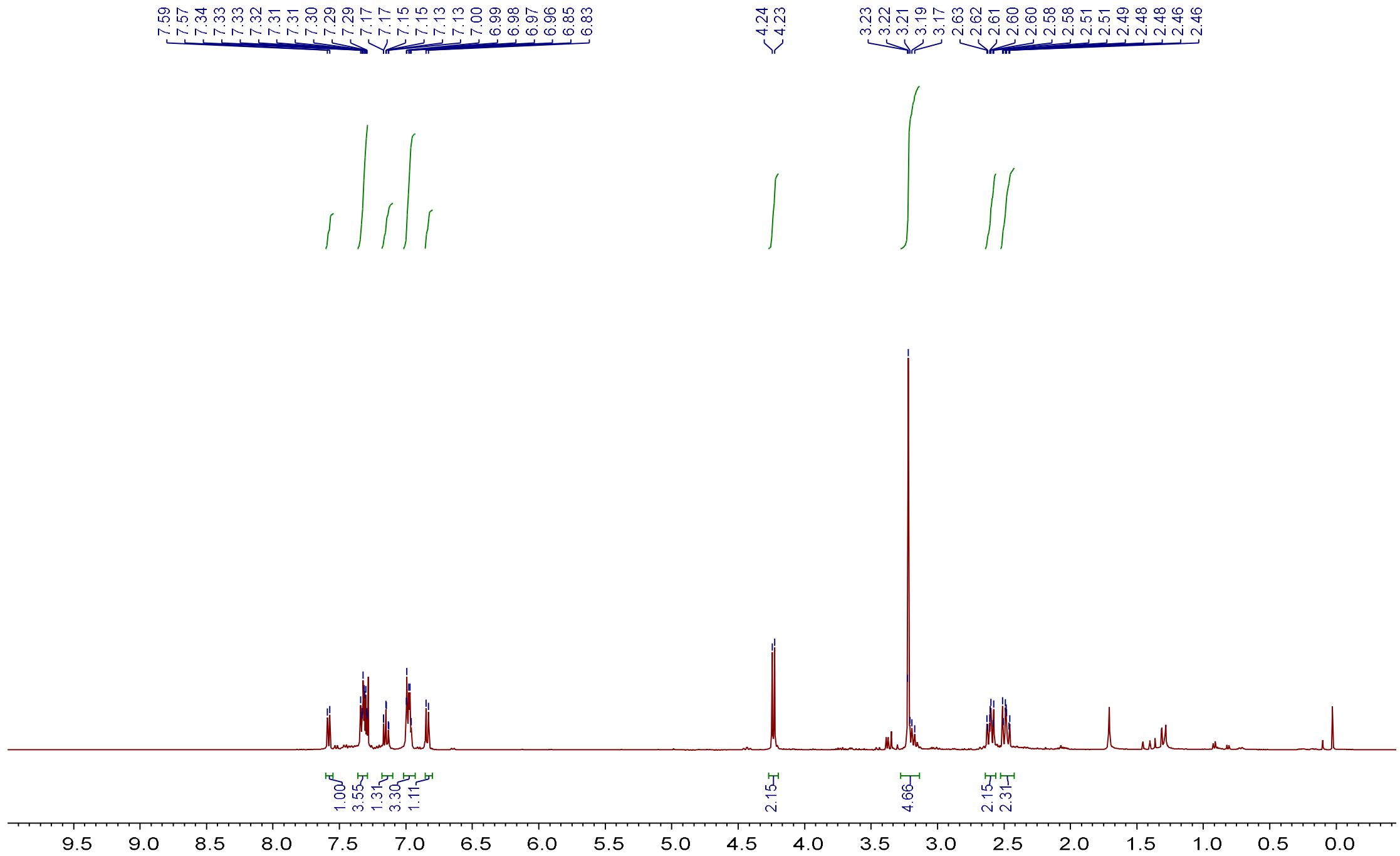

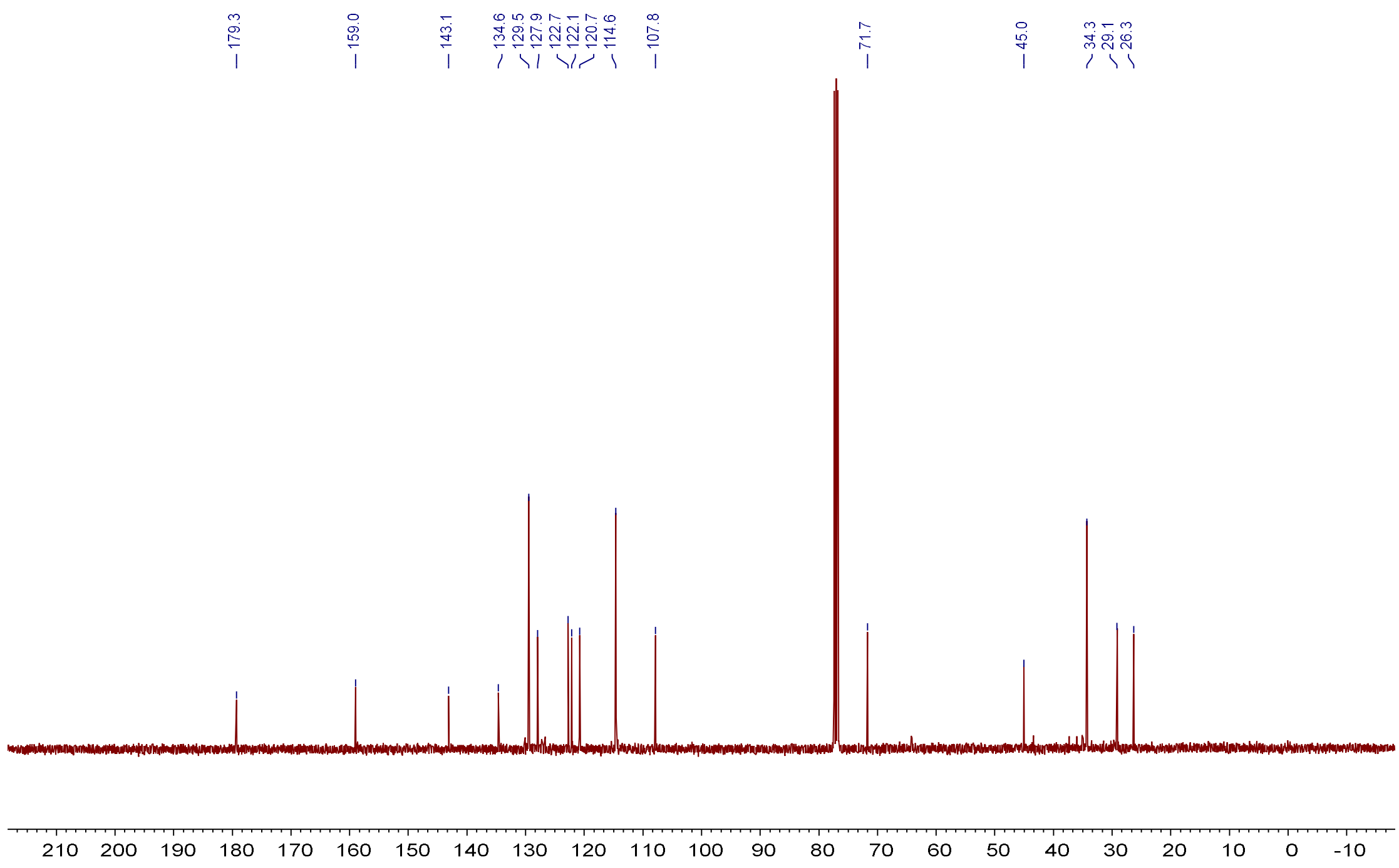

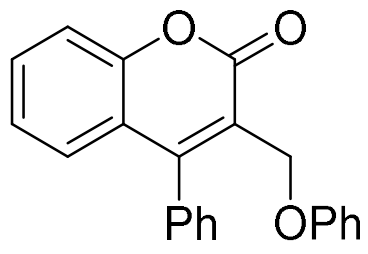
O
O
Ph
OPh

7m

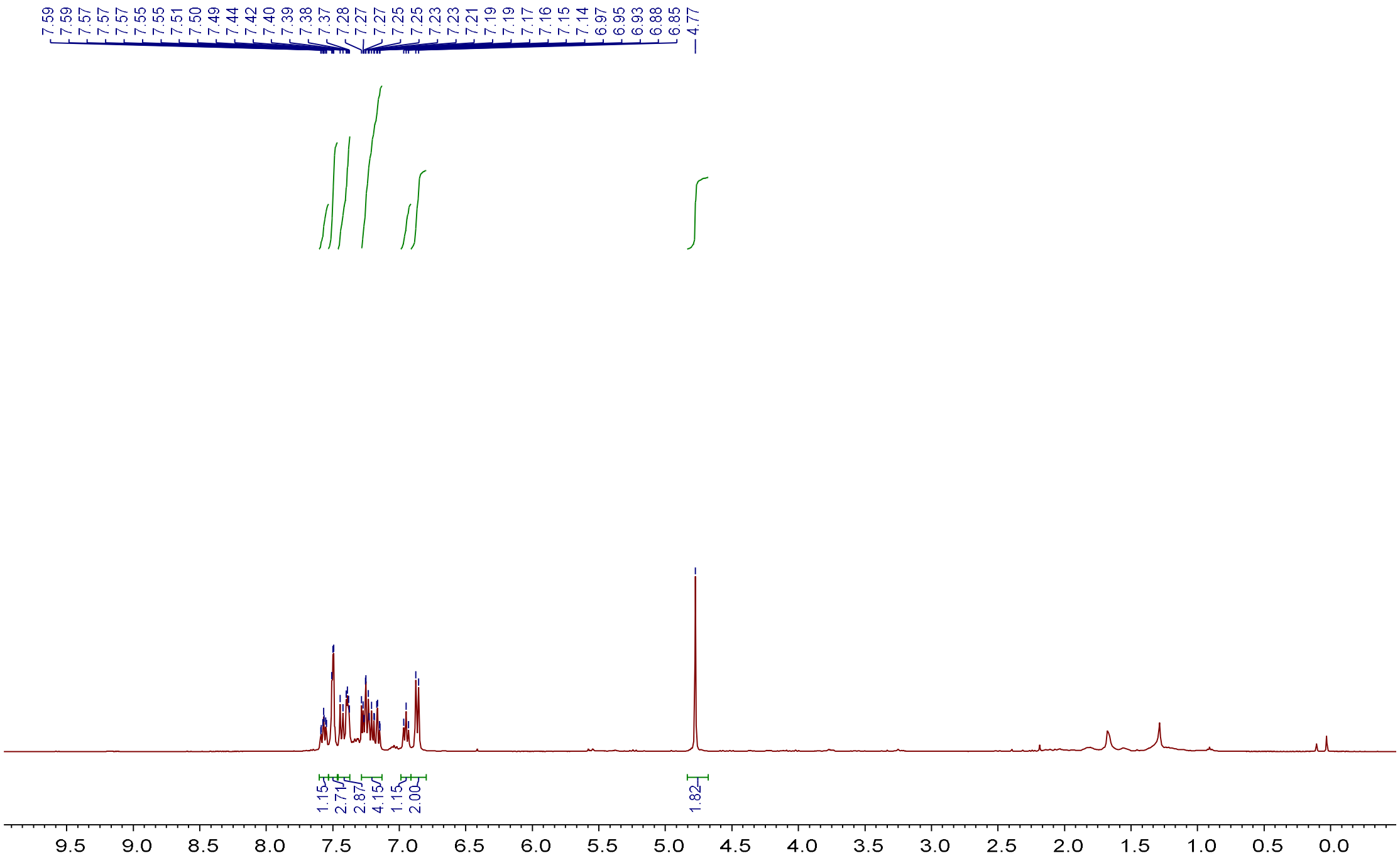
7.59
7.59
7.57
7.57
7.55
7.55
7.51
7.50
7.49
7.44
7.42
7.40
7.39
7.38
7.37
7.28
7.27
7.27
7.25
7.25
7.23
7.23
7.21
7.19
7.19
7.17
7.16
7.15
7.14
6.97
6.95
6.93
6.88
6.85
4.77
1.15
2.71
2.87
4.15
1.15
2.00
1.82
9.5 9.0 8.5 8.0 7.5 7.0 6.5 6.0 5.5 5.0 4.5 4.0 3.5 3.0 2.5 2.0 1.5 1.0 0.5 0.0

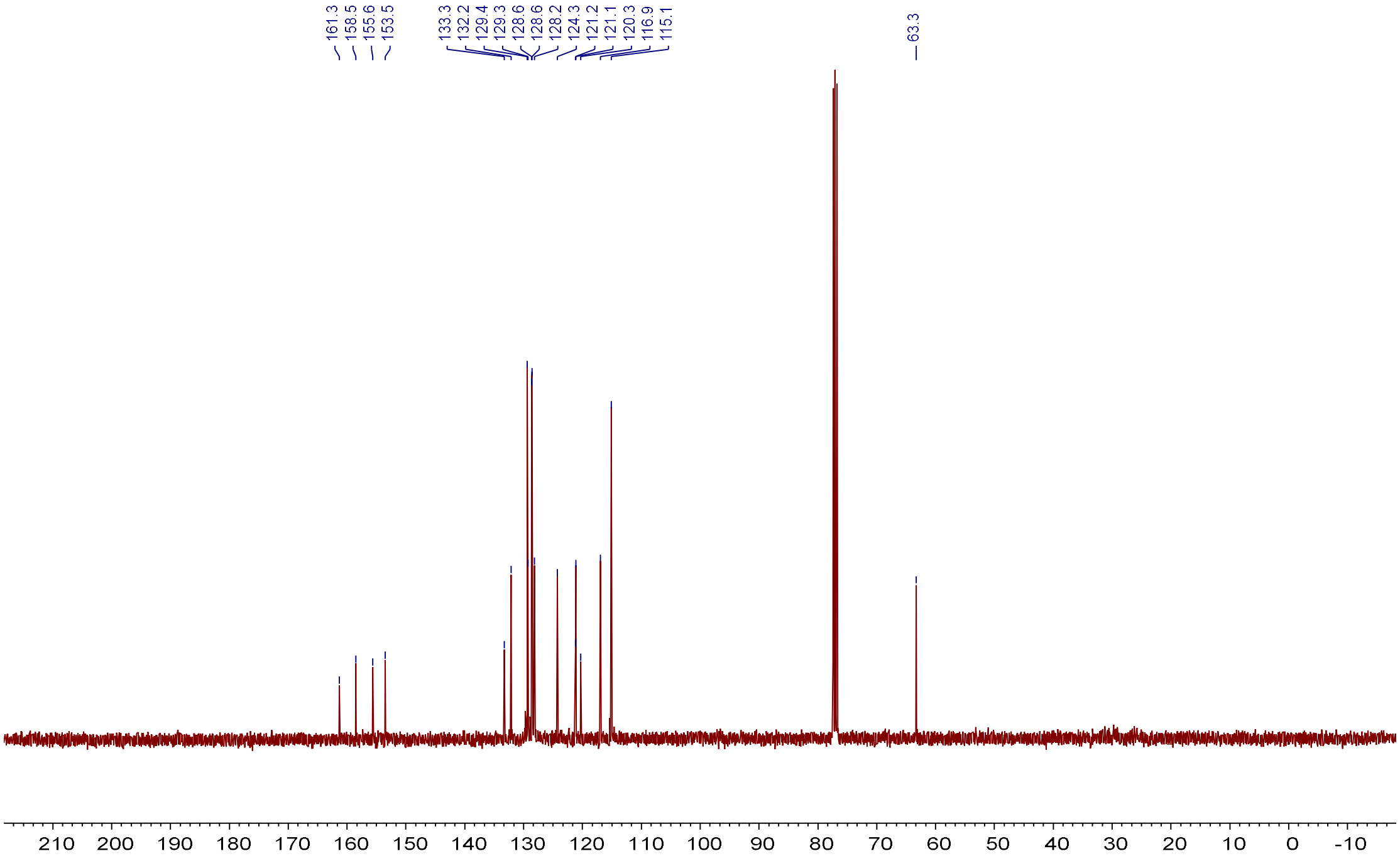
161.3
158.5
155.6
153.5
133.3
132.2
129.4
129.3
128.6
128.6
128.2
124.3
121.2
121.1
120.3
116.9
115.1
63.3
210 200 190 180 170 160 150 140 130 120 110 100 90 80 70 60 50 40 30 20 10 0 -10

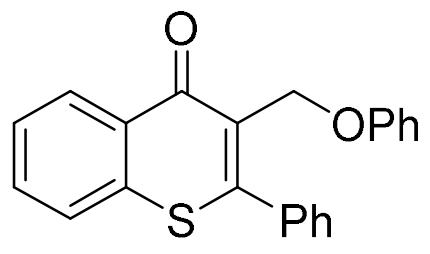
O
OPh
S
Ph

7n

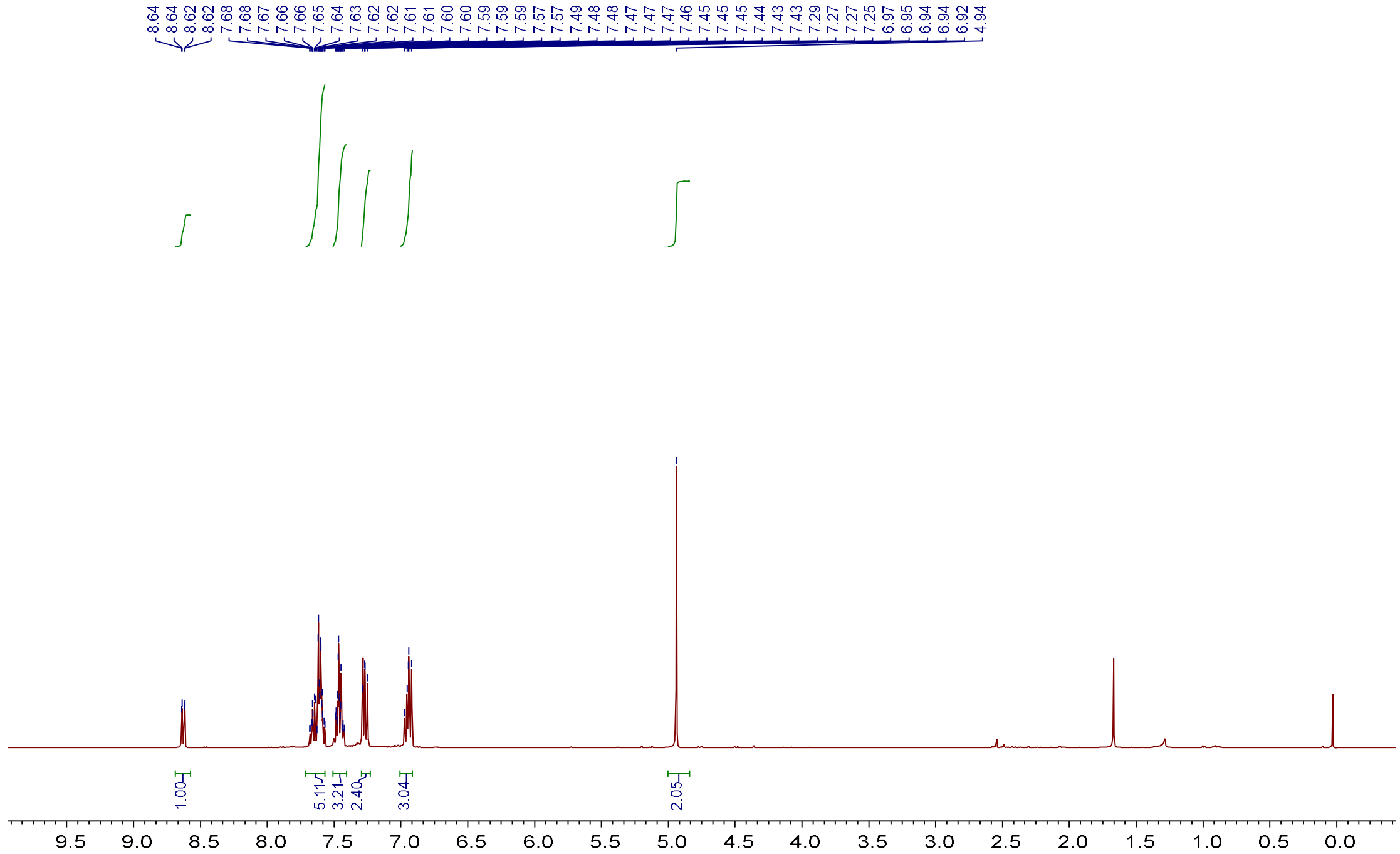
8.64 8.64 8.62 8.62 7.68 7.68 7.67 7.66 7.66 7.65 7.64 7.63 7.62 7.62 7.61 7.61 7.60 7.60 7.59 7.59 7.59 7.57 7.57 7.49 7.48 7.48 7.47 7.47 7.47 7.46 7.45 7.45 7.45 7.44 7.43 7.43 7.29 7.27 7.27 7.25 6.97 6.95 6.94 6.94 6.92 4.94
1.00
5.11
3.21
2.40
3.04
2.05
9.5 9.0 8.5 8.0 7.5 7.0 6.5 6.0 5.5 5.0 4.5 4.0 3.5 3.0 2.5 2.0 1.5 1.0 0.5 0.0

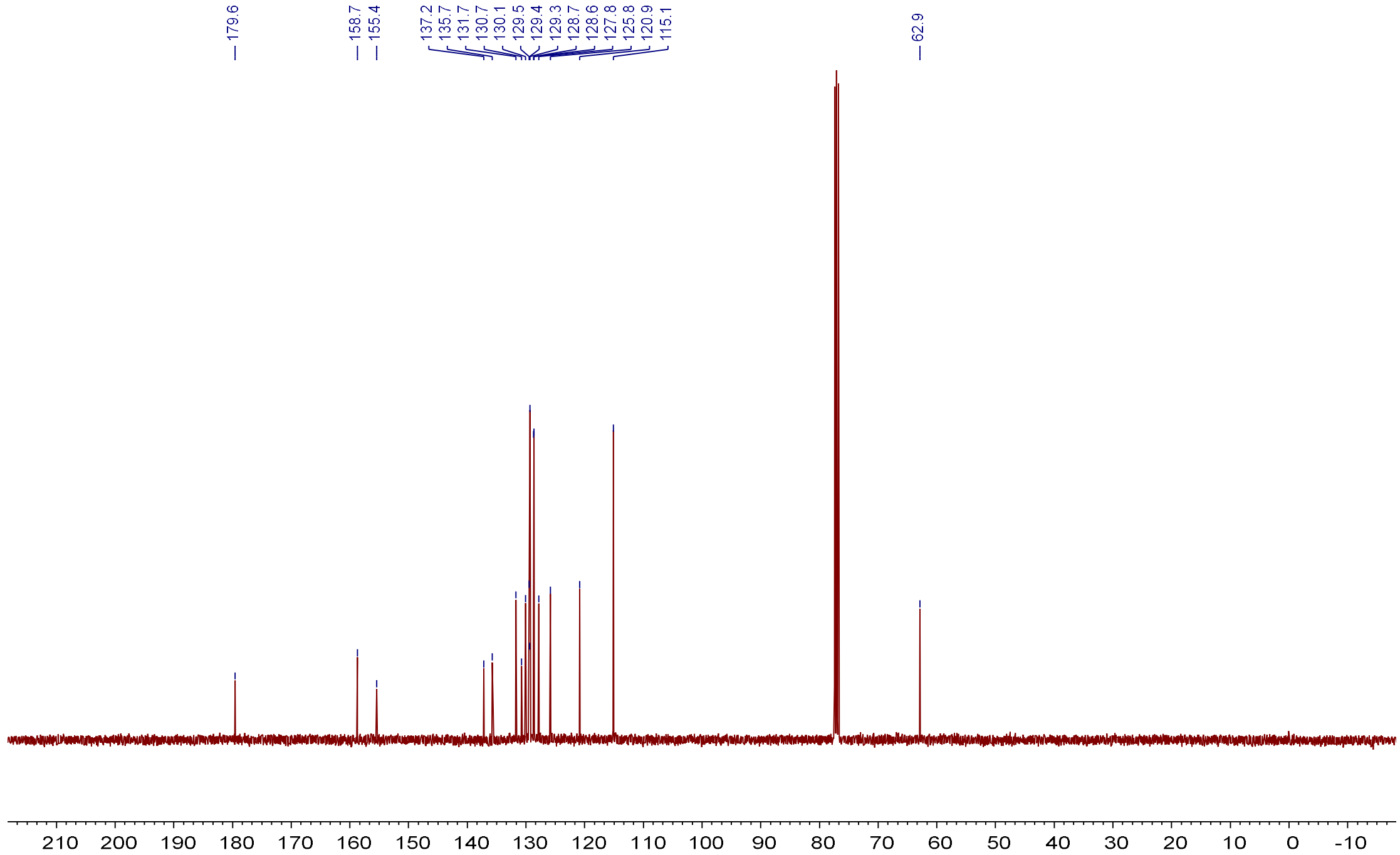
179.6
158.7
155.4
137.2 135.7 131.7 130.7 130.1 129.5 129.4 129.3 128.7 128.6 127.8 125.8 120.9 115.1
62.9
210 200 190 180 170 160 150 140 130 120 110 100 90 80 70 60 50 40 30 20 10 0 -10

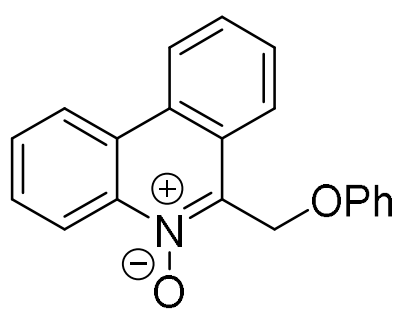

8

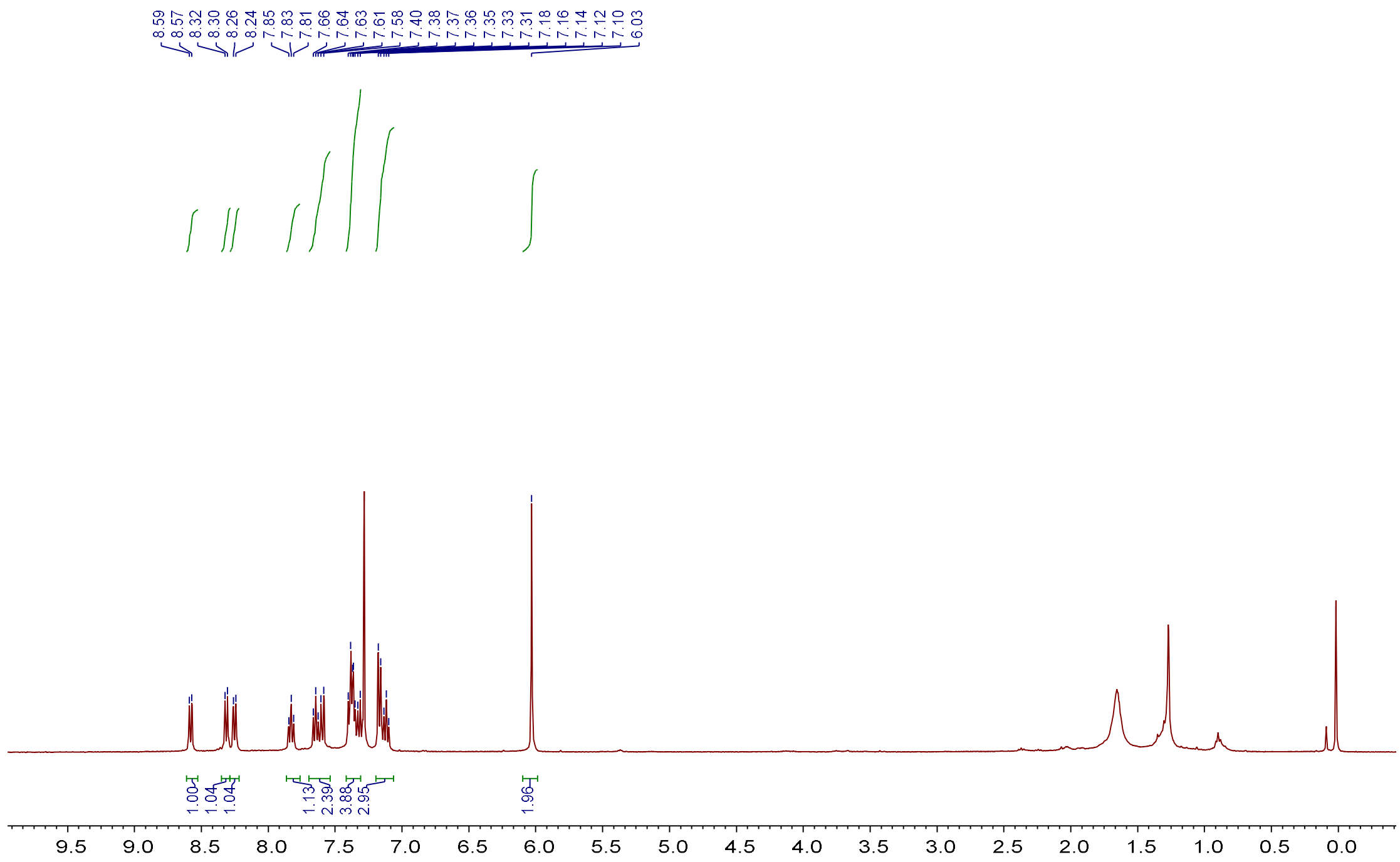

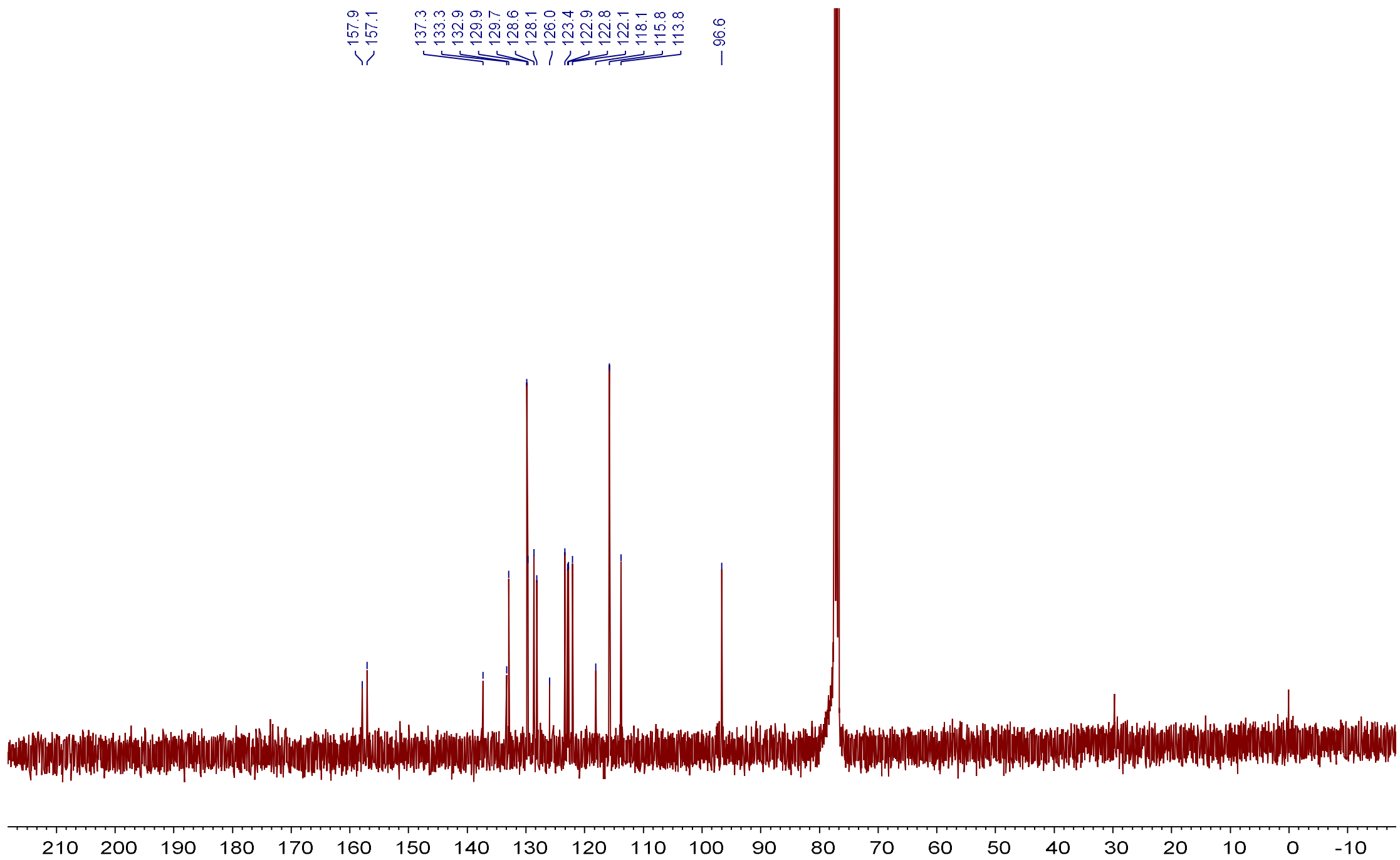

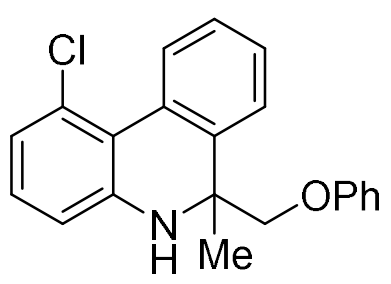

9

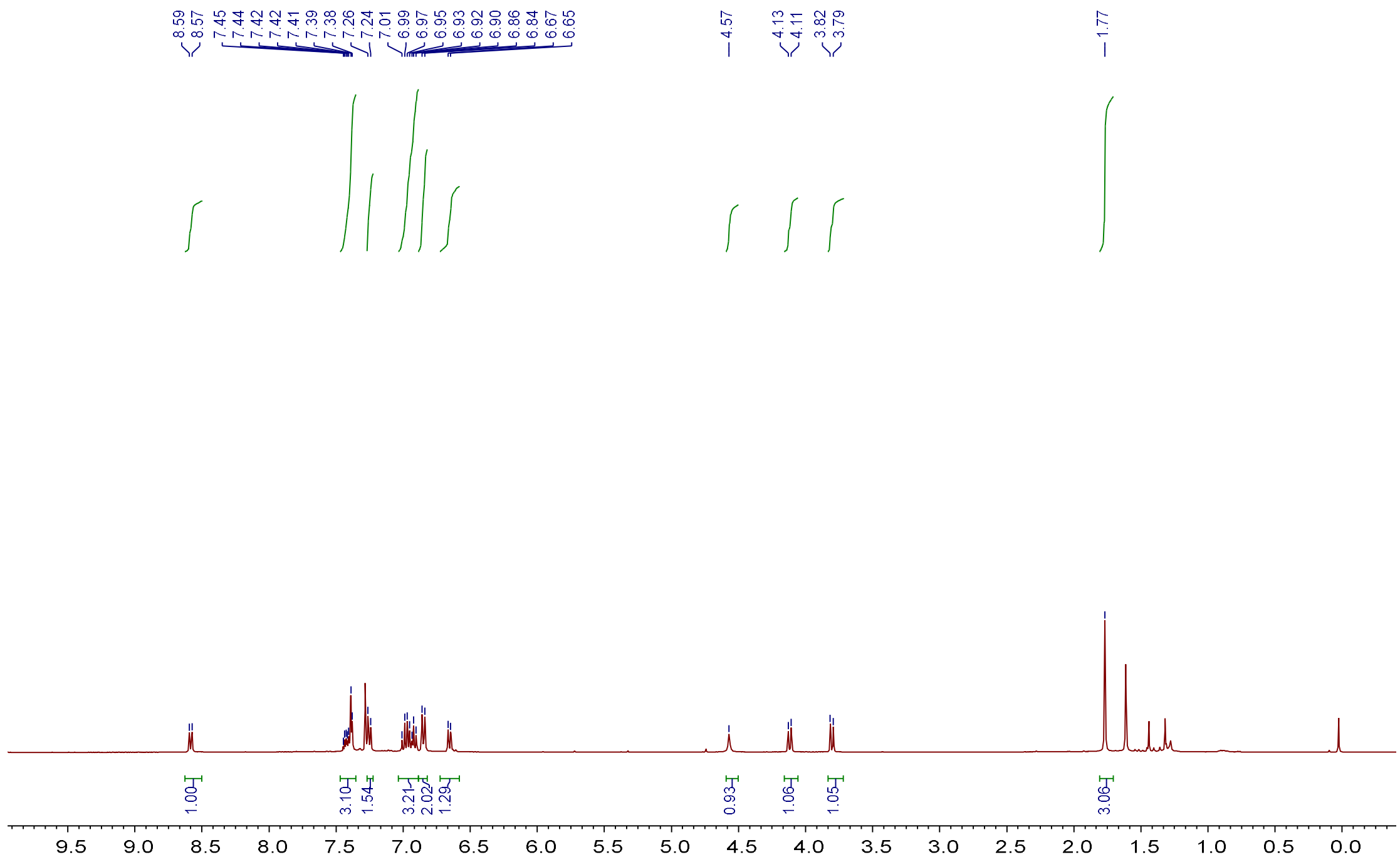

8.59
8.57
7.45
7.44
7.42
7.42
7.41
7.39
7.38
7.26
7.24
7.01
6.99
6.97
6.95
6.93
6.92
6.90
6.86
6.84
6.67
6.65
4.57
4.13
4.11
3.82
3.79
1.77
1.00
3.10
1.54
3.21
2.02
1.29
0.93
1.06
1.05
3.06
9.5 9.0 8.5 8.0 7.5 7.0 6.5 6.0 5.5 5.0 4.5 4.0 3.5 3.0 2.5 2.0 1.5 1.0 0.5 0.0

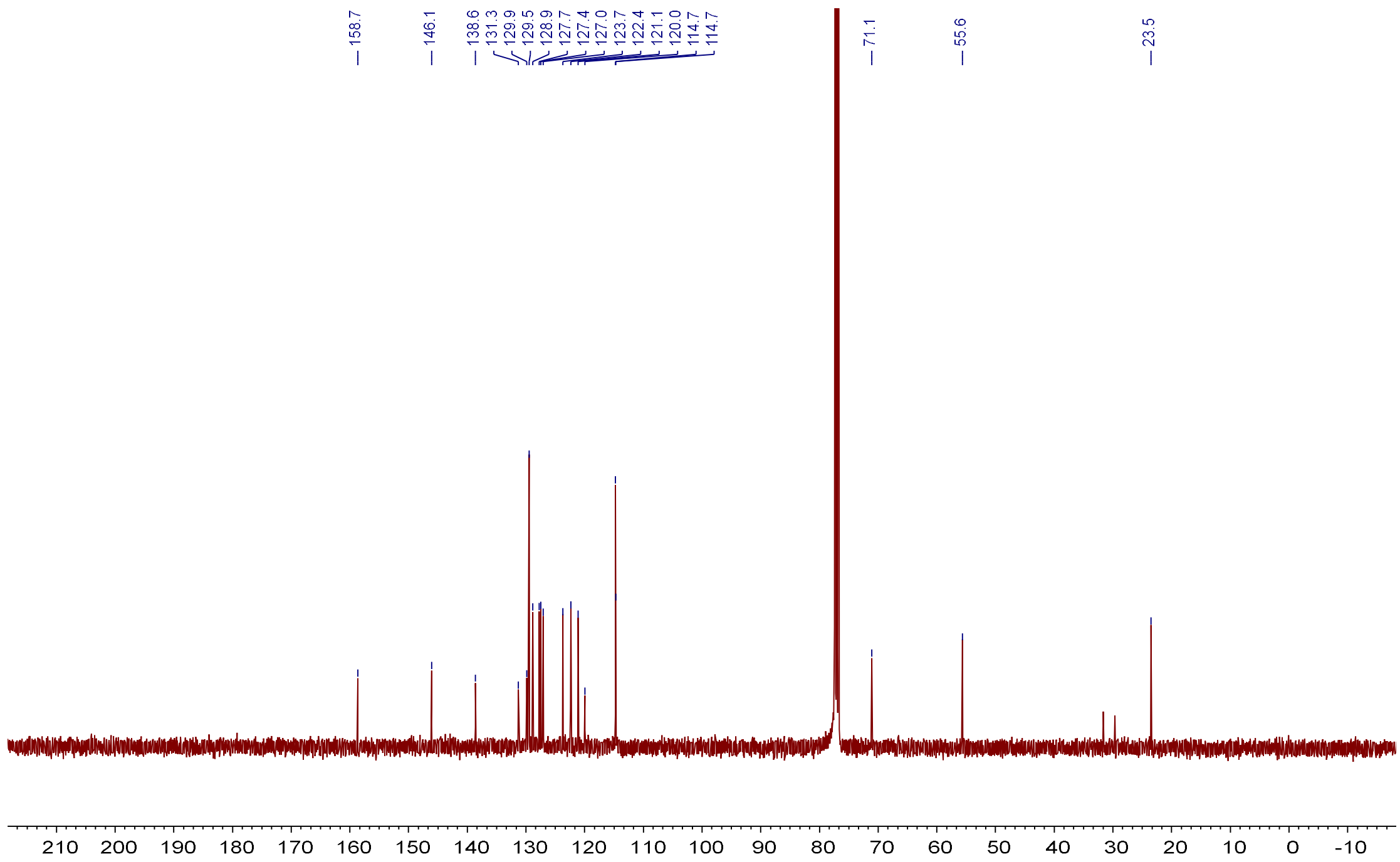

158.7
146.1
138.6
131.3
129.9
129.5
128.9
127.7
127.4
127.0
123.7
122.4
121.1
120.0
114.7
114.7
71.1
55.6
23.5
210 200 190 180 170 160 150 140 130 120 110 100 90 80 70 60 50 40 30 20 10 0 -10

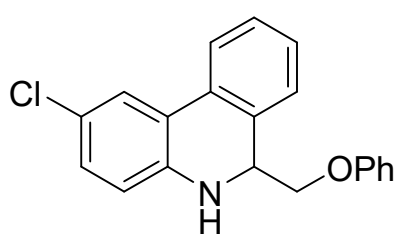
Cl
N
H
OPh

10

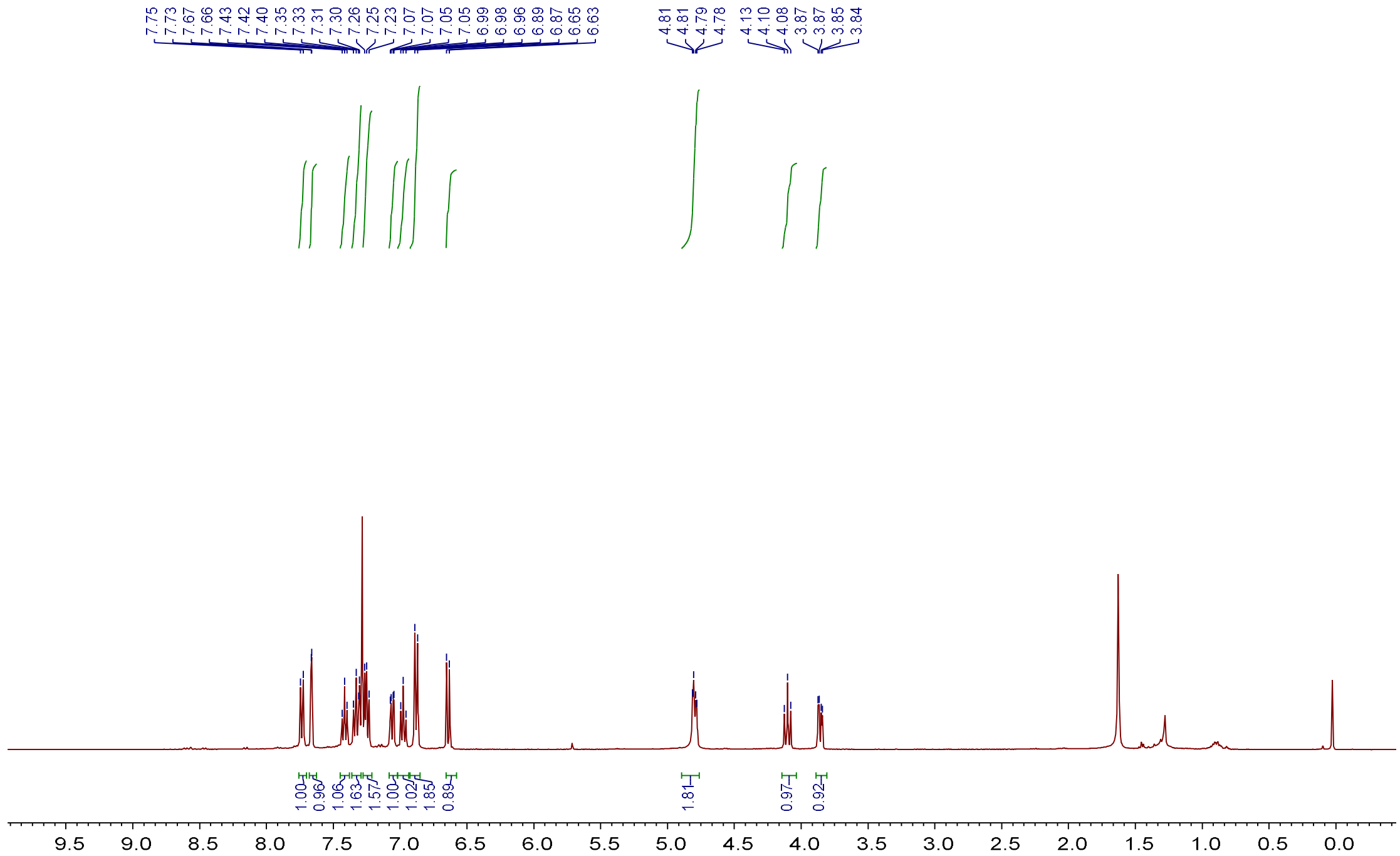
7.75 7.73 7.67 7.66 7.43 7.42 7.40 7.35 7.33 7.31 7.30 7.26 7.25 7.23 7.07 7.07 7.05 7.05 6.99 6.98 6.96 6.89 6.87 6.65 6.63
4.81 4.81 4.79 4.78
4.13 4.10 4.08 3.87 3.87 3.85 3.84
1.00 0.96 1.06 1.63 1.57 1.00 1.02 1.85 0.89
1.81
0.97 0.92
9.5 9.0 8.5 8.0 7.5 7.0 6.5 6.0 5.5 5.0 4.5 4.0 3.5 3.0 2.5 2.0 1.5 1.0 0.5 0.0

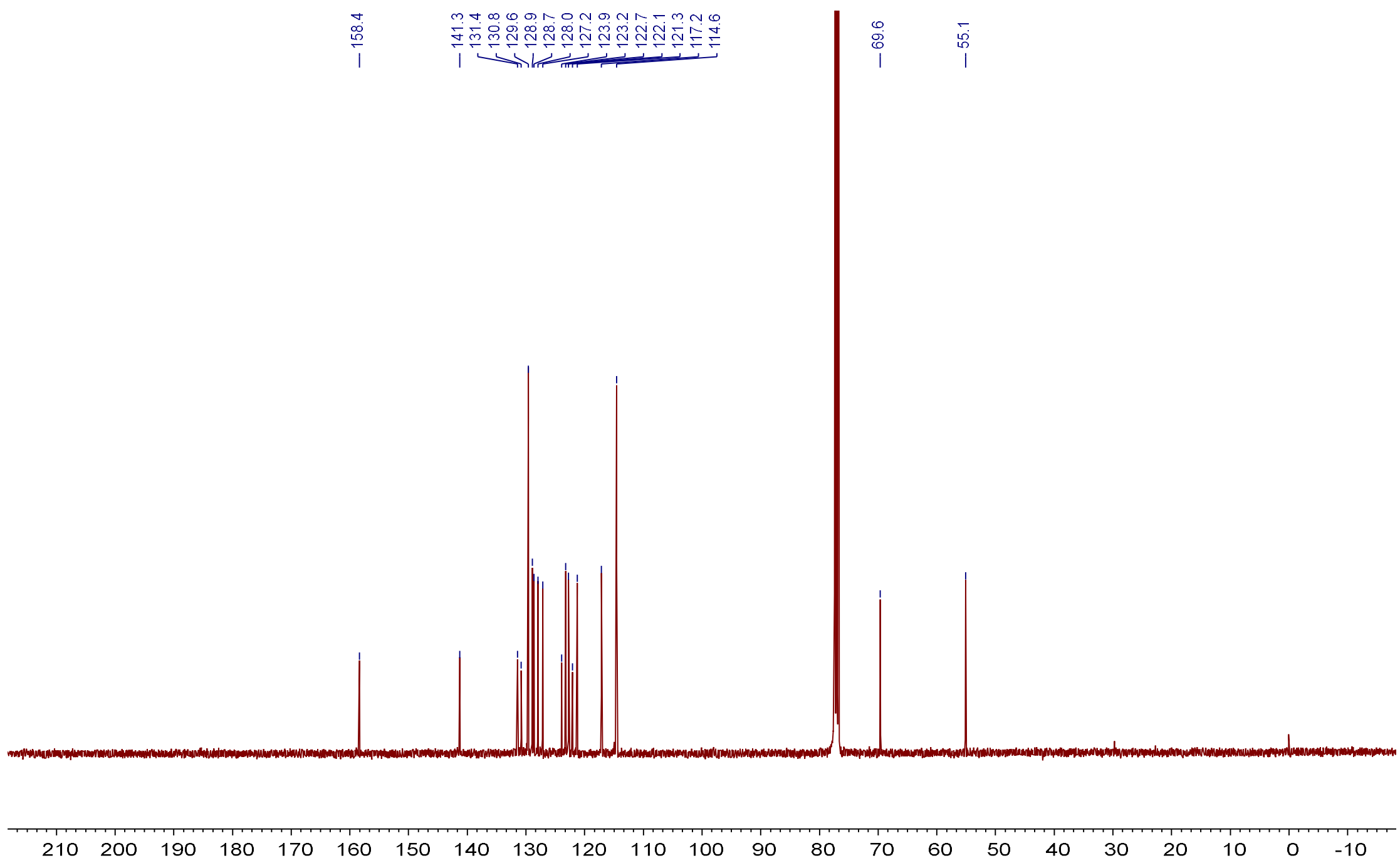
158.4
141.3 131.4 130.8 129.6 128.9 128.7 128.0 127.2 123.9 123.2 122.7 122.1 121.3 117.2 114.6
69.6
55.1
210 200 190 180 170 160 150 140 130 120 110 100 90 80 70 60 50 40 30 20 10 0 -10

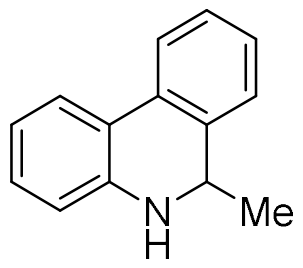
N
H
Me

11

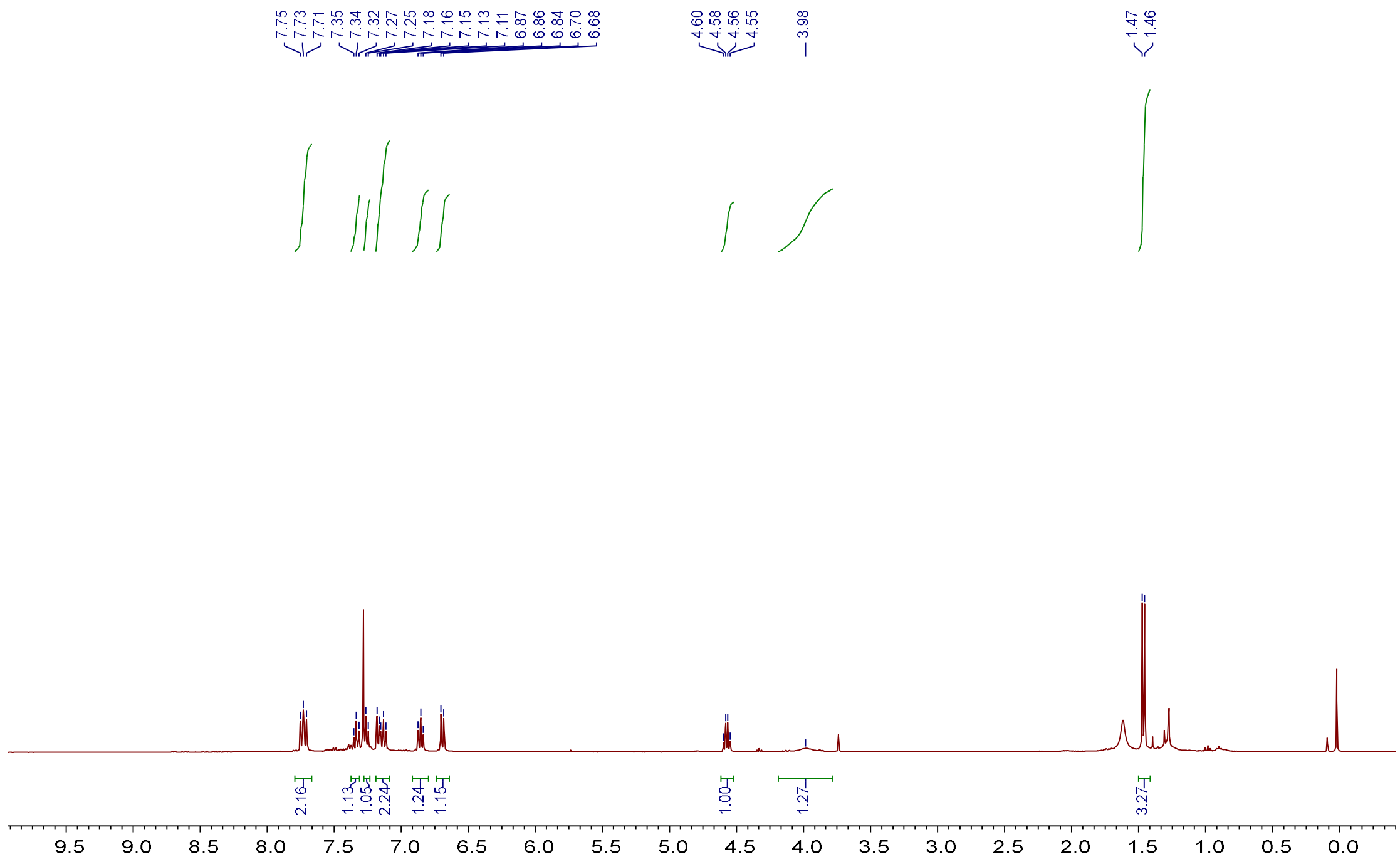
7.75
7.73
7.71
7.35
7.34
7.32
7.27
7.25
7.18
7.16
7.15
7.13
7.11
6.87
6.86
6.84
6.70
6.68
4.60
4.58
4.56
4.55
3.98
1.47
1.46
2.16
1.13
1.05
2.24
1.24
1.15
1.00
1.27
3.27
9.5 9.0 8.5 8.0 7.5 7.0 6.5 6.0 5.5 5.0 4.5 4.0 3.5 3.0 2.5 2.0 1.5 1.0 0.5 0.0

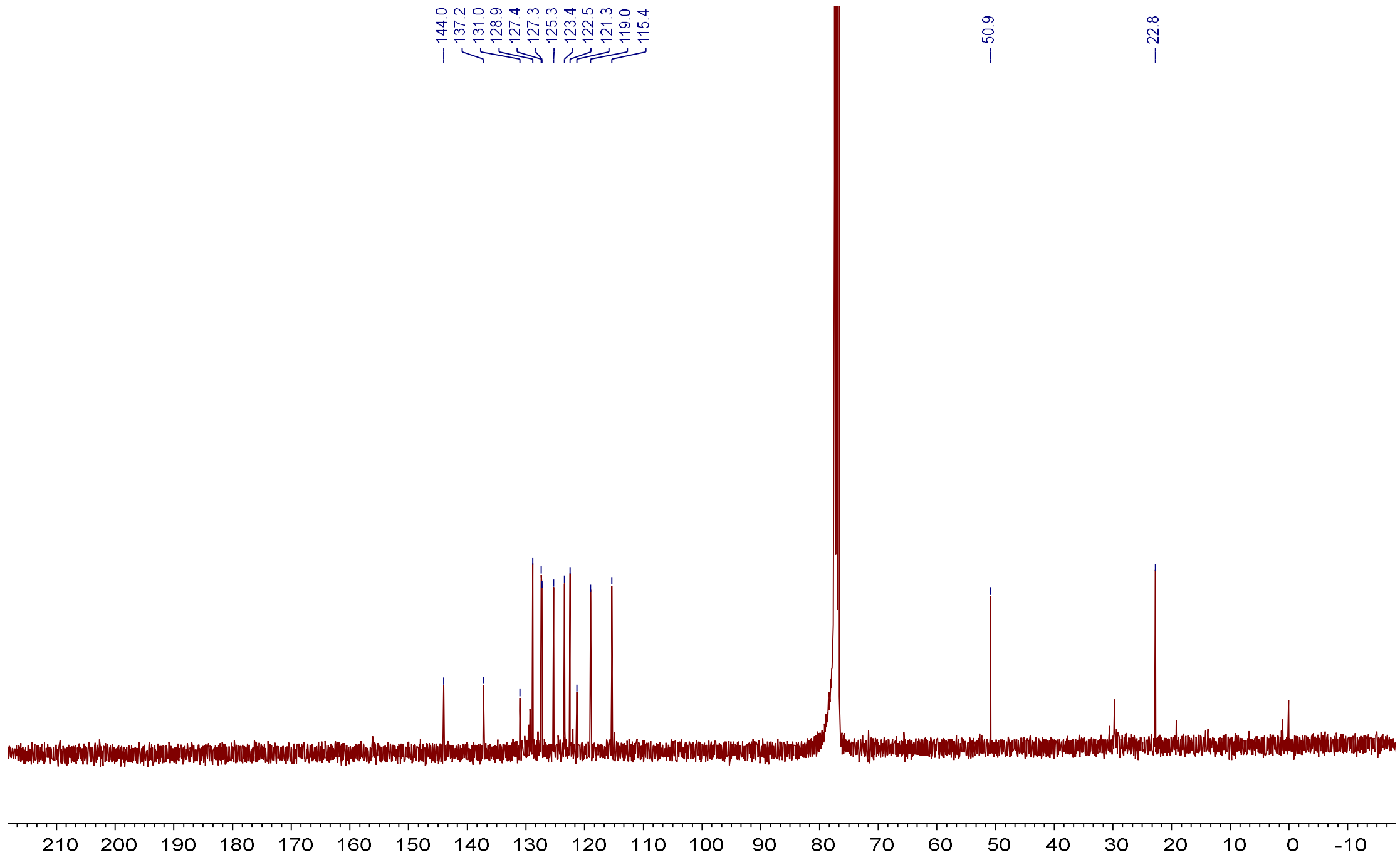
144.0
137.2
131.0
128.9
127.4
127.3
125.3
123.4
122.5
121.3
119.0
115.4
50.9
22.8
210 200 190 180 170 160 150 140 130 120 110 100 90 80 70 60 50 40 30 20 10 0 -10

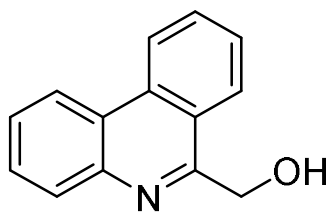

13

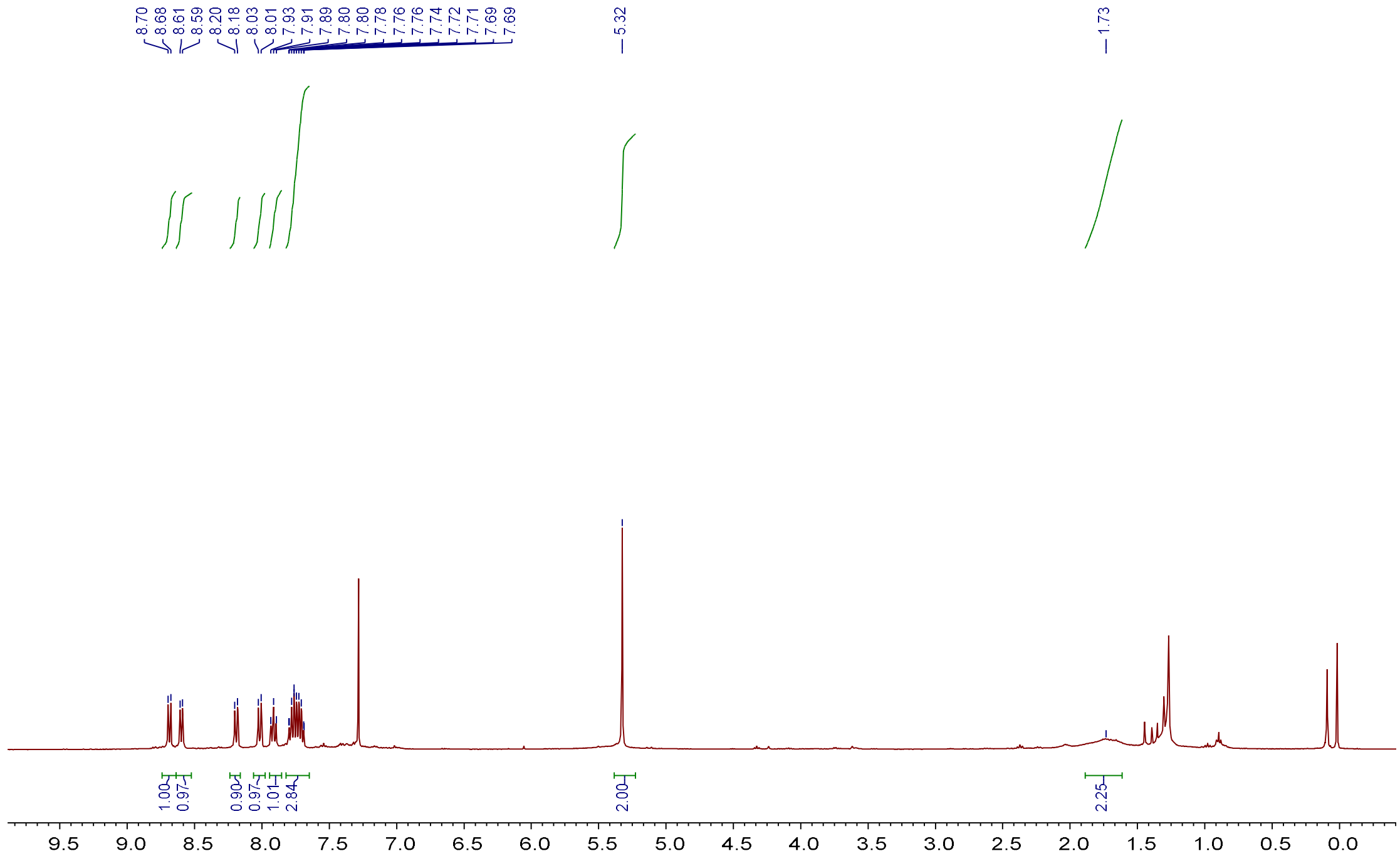

8.70
8.68
8.61
8.59
8.20
8.18
8.03
8.01
7.93
7.91
7.89
7.80
7.80
7.78
7.76
7.76
7.74
7.72
7.71
7.69
7.69
5.32
1.73
1.00
0.97
0.90
0.97
1.01
2.84
2.00
2.25
9.5
9.0
8.5
8.0
7.5
7.0
6.5
6.0
5.5
5.0
4.5
4.0
3.5
3.0
2.5
2.0
1.5
1.0
0.5
0.0

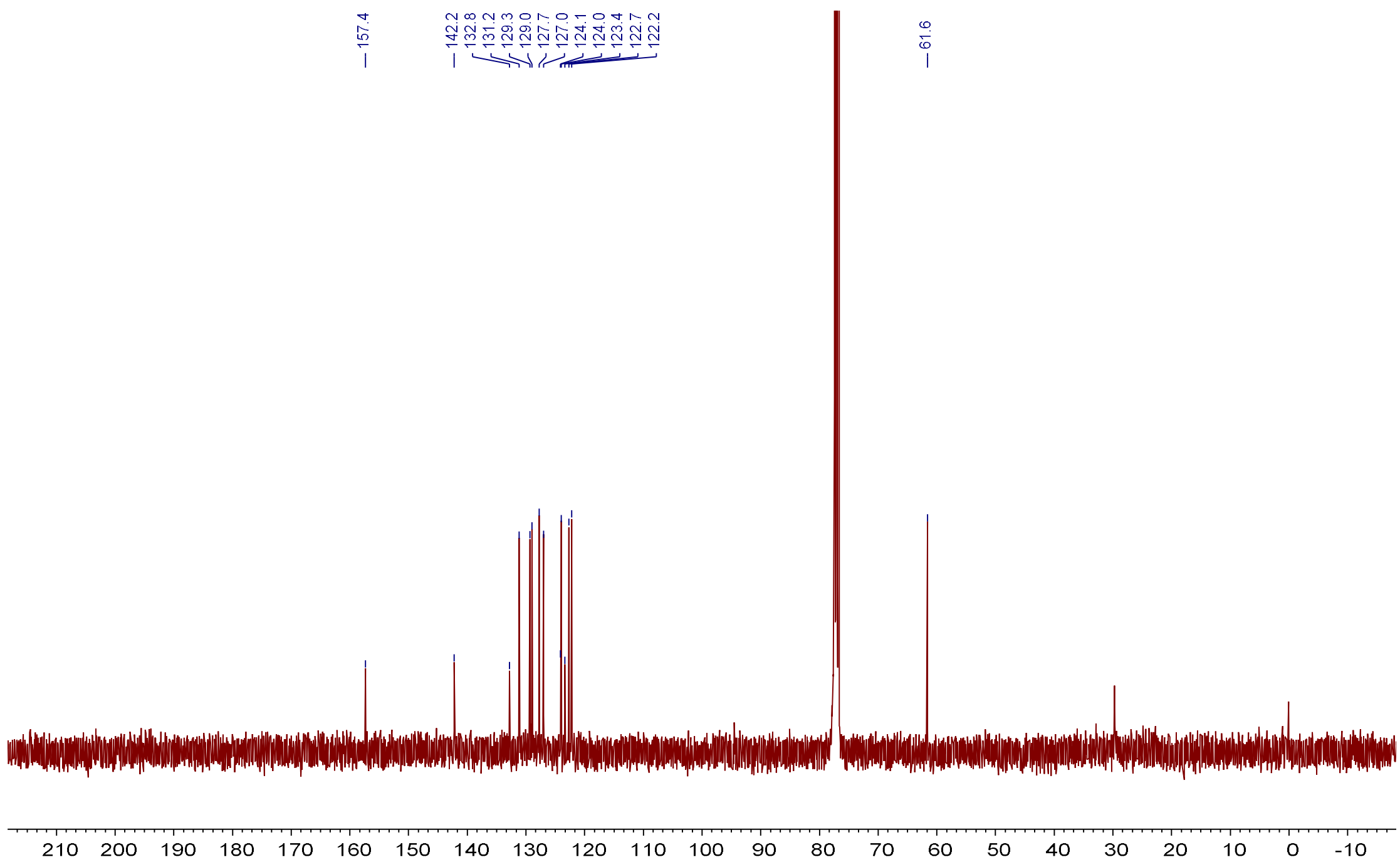

157.4
142.2
132.8
131.2
129.3
129.0
127.7
127.0
124.1
124.0
123.4
122.7
122.2
61.6
210
200
190
180
170
160
150
140
130
120
110
100
90
80
70
60
50
40
30
20
10
0
-10

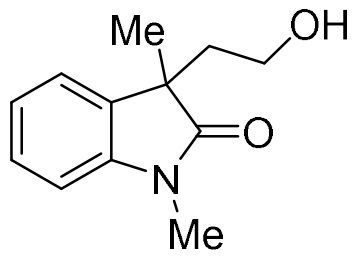
Me
OH
O
N
Me

15

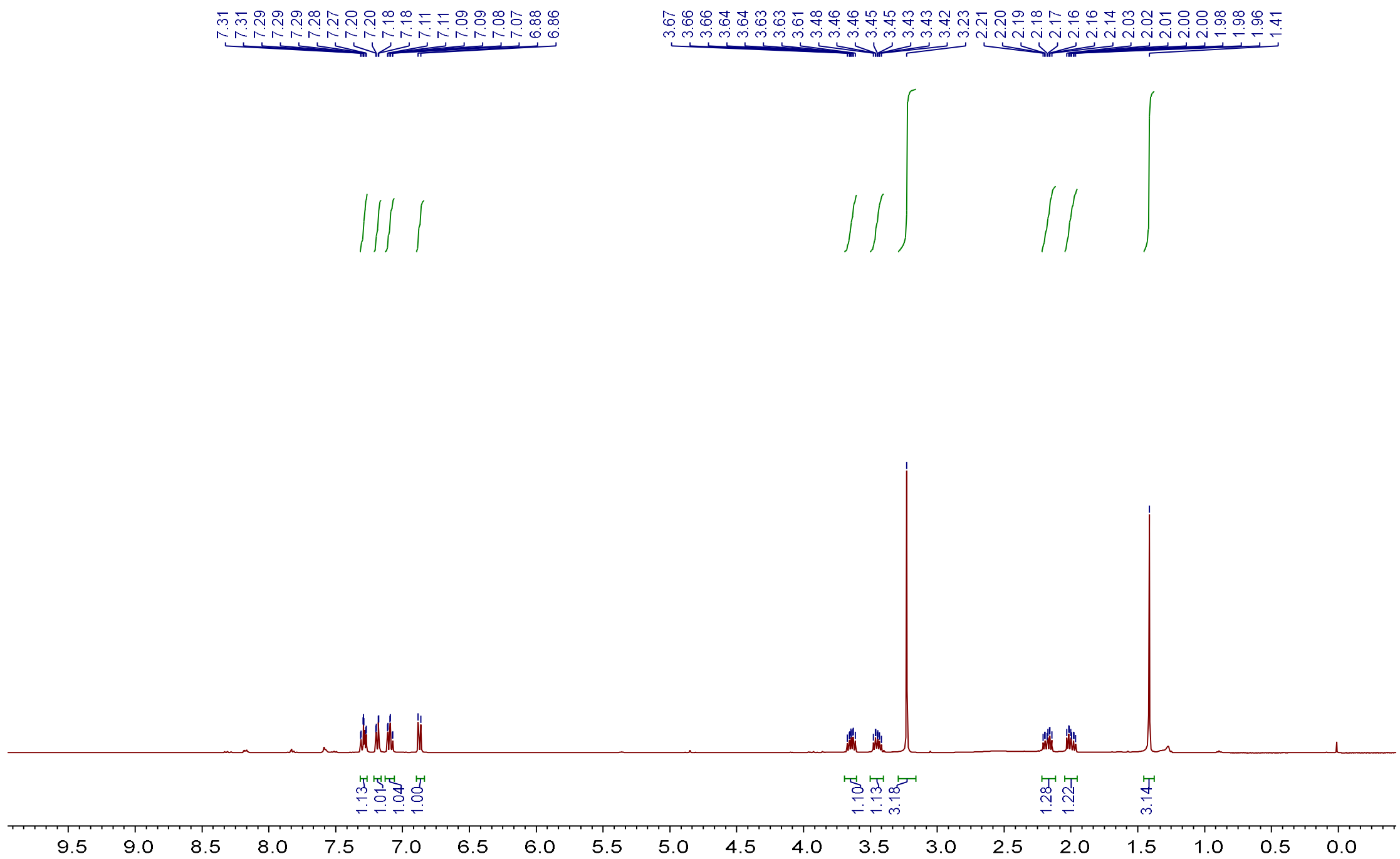
7.31 7.31 7.29 7.29 7.29 7.28 7.27 7.20 7.20 7.18 7.18 7.11 7.11 7.09 7.09 7.08 7.07 6.88 6.86
3.67 3.66 3.66 3.64 3.64 3.63 3.63 3.61 3.48 3.46 3.46 3.45 3.45 3.43 3.43 3.42 3.23
2.21 2.20 2.19 2.18 2.17 2.16 2.16 2.14 2.03 2.02 2.01 2.00 2.00 1.98 1.98 1.96 1.41
1.13 1.01 1.04 1.00
1.10 1.13 3.18
1.28 1.22
3.14
9.5 9.0 8.5 8.0 7.5 7.0 6.5 6.0 5.5 5.0 4.5 4.0 3.5 3.0 2.5 2.0 1.5 1.0 0.5 0.0

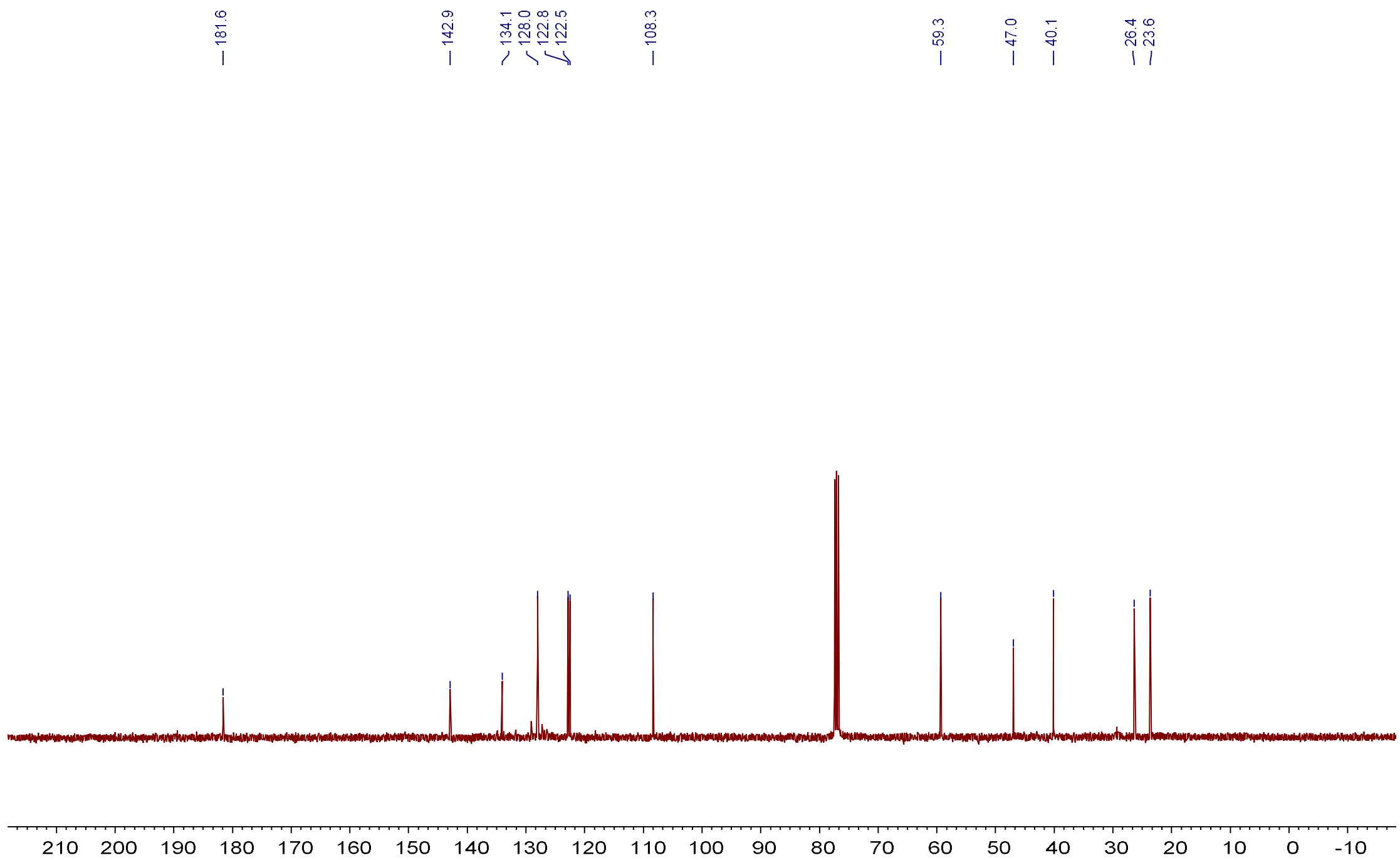
181.6
142.9
134.1 128.0 122.8 122.5
108.3
59.3
47.0
40.1
26.4 23.6
210 200 190 180 170 160 150 140 130 120 110 100 90 80 70 60 50 40 30 20 10 0 -10

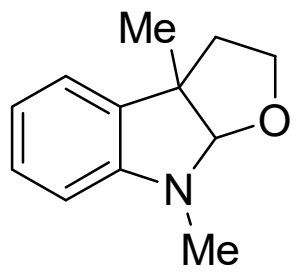
Me
O
N
Me

16

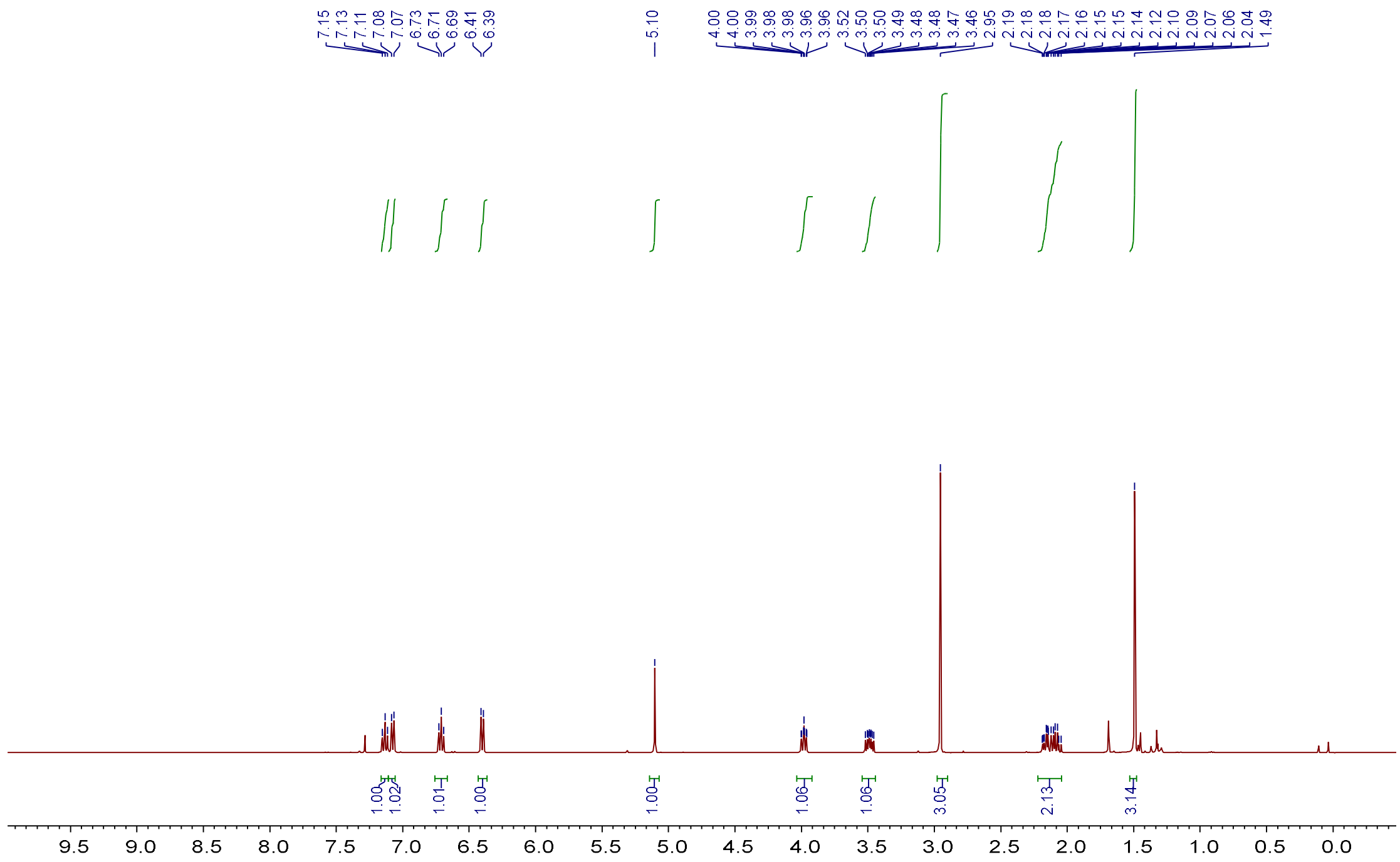
7.15
7.13
7.11
7.08
7.07
6.73
6.71
6.69
6.41
6.39
5.10
4.00
4.00
3.99
3.98
3.98
3.96
3.96
3.52
3.50
3.50
3.49
3.48
3.48
3.47
3.46
2.95
2.19
2.18
2.18
2.17
2.16
2.15
2.15
2.14
2.12
2.10
2.09
2.07
2.06
2.04
1.49
1.00
1.02
1.01
1.00
1.00
1.06
1.06
3.05
2.13
3.14
9.5 9.0 8.5 8.0 7.5 7.0 6.5 6.0 5.5 5.0 4.5 4.0 3.5 3.0 2.5 2.0 1.5 1.0 0.5 0.0

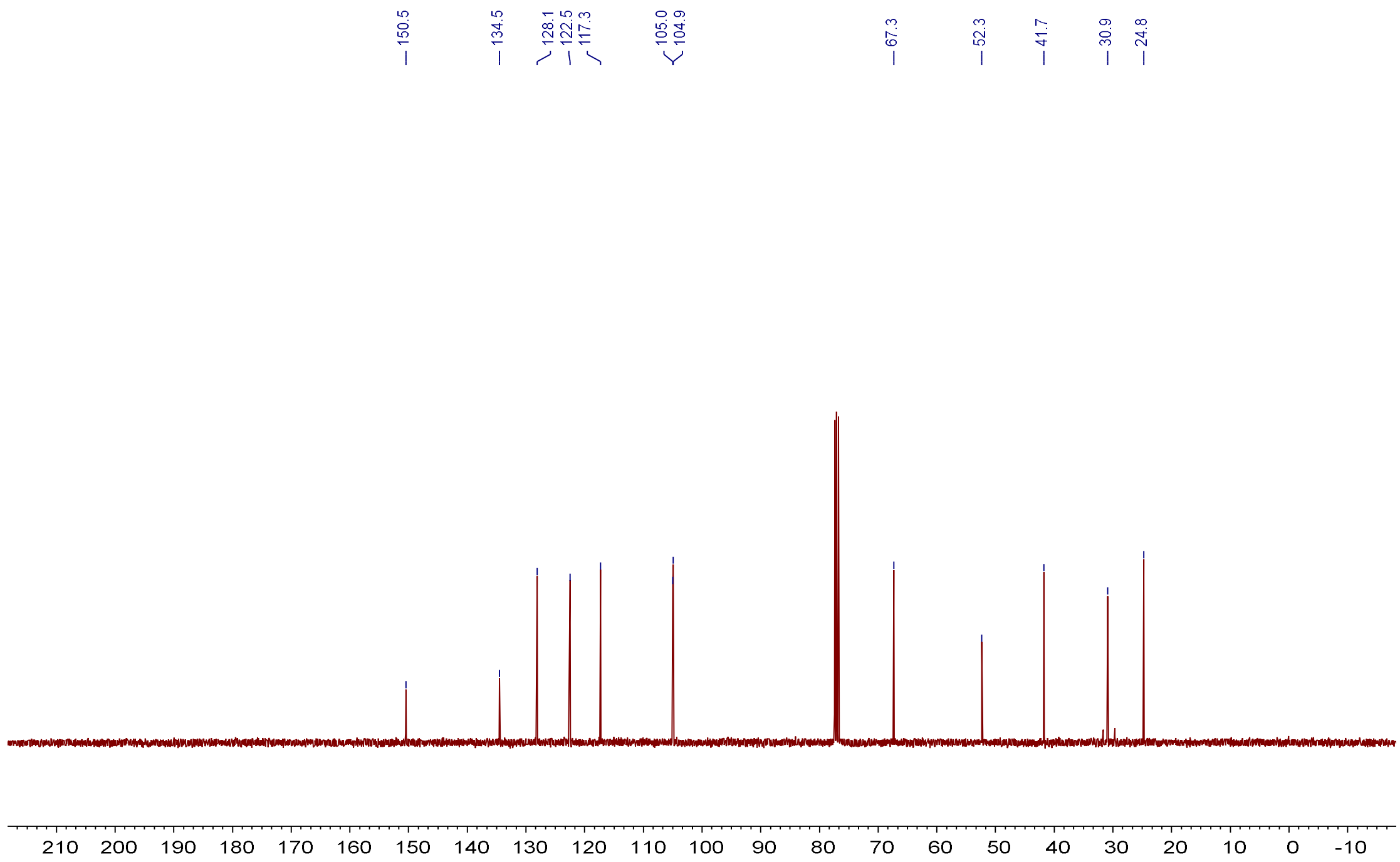
150.5
134.5
128.1
122.5
117.3
105.0
104.9
67.3
52.3
41.7
30.9
24.8
210 200 190 180 170 160 150 140 130 120 110 100 90 80 70 60 50 40 30 20 10 0 -10

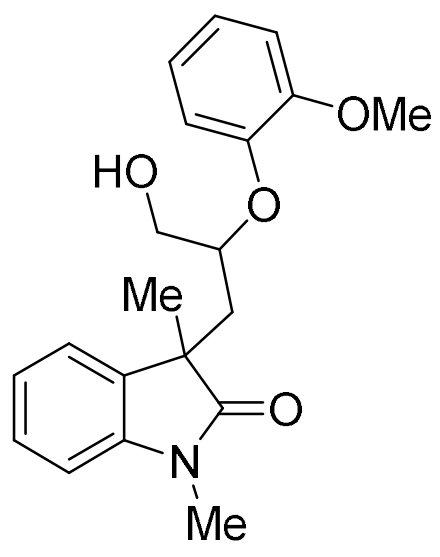
OMe
HO
O
Me
O
N
Me

17

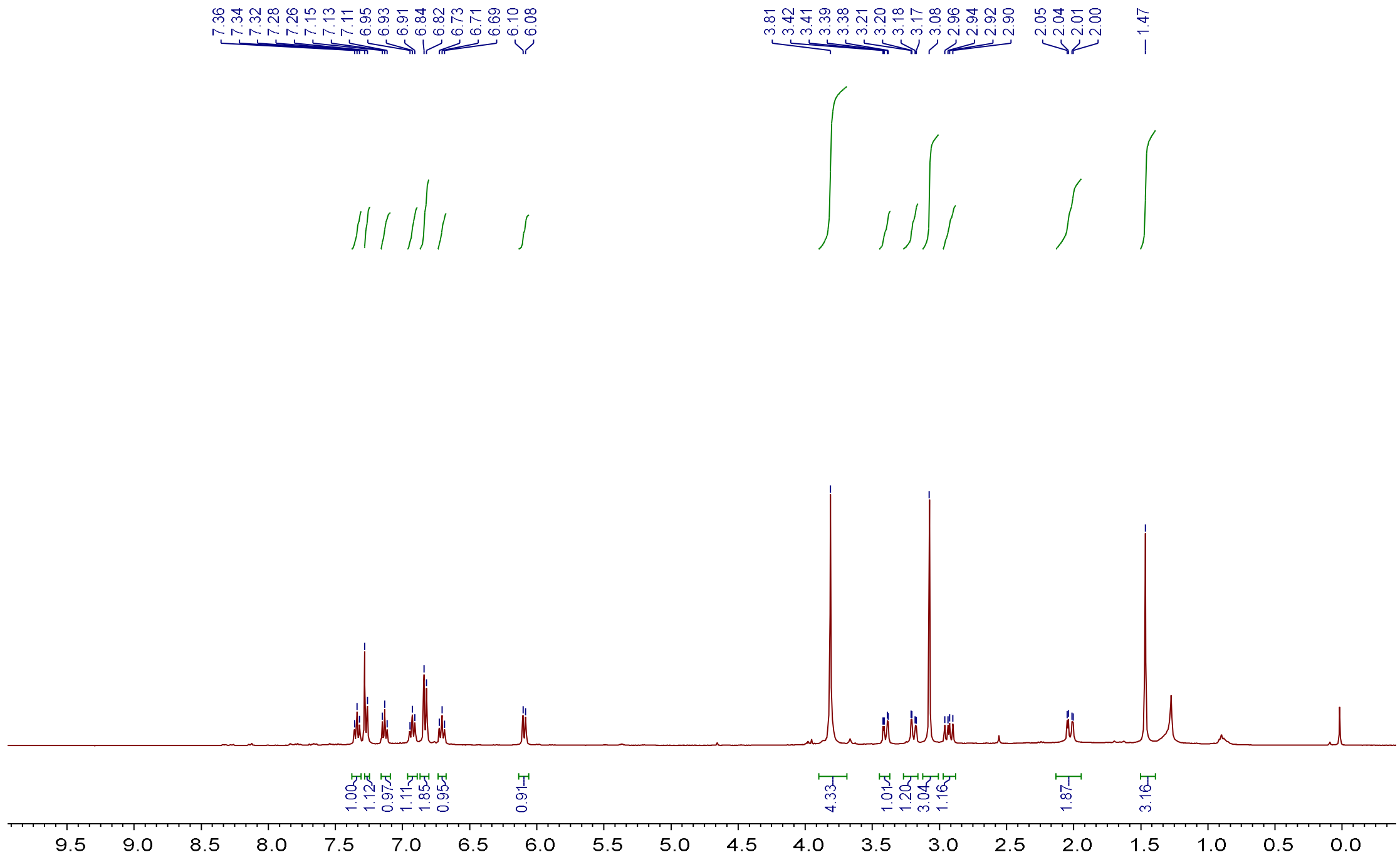
7.36 7.34 7.32 7.28 7.26 7.15 7.13 7.11 6.95 6.93 6.91 6.84 6.82 6.73 6.71 6.69 6.10 6.08
3.81 3.42 3.41 3.39 3.38 3.21 3.20 3.18 3.17 3.08 2.96 2.94 2.92 2.90 2.05 2.04 2.01 2.00 1.47
1.00 1.12 0.97 1.11 1.85 0.95 0.91 4.33 1.01 1.20 3.04 1.16 1.87 3.16
9.5 9.0 8.5 8.0 7.5 7.0 6.5 6.0 5.5 5.0 4.5 4.0 3.5 3.0 2.5 2.0 1.5 1.0 0.5 0.0

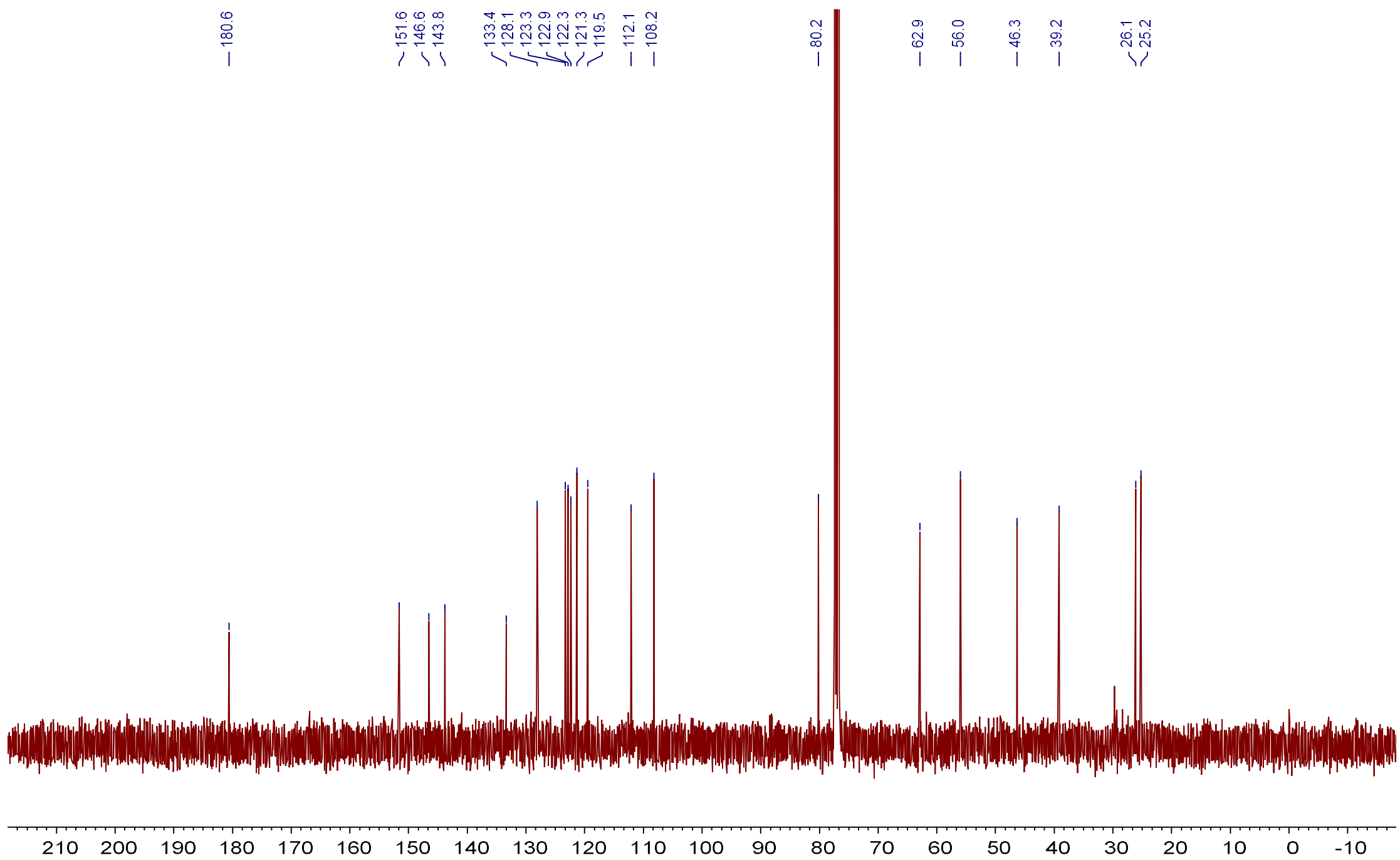
180.6
151.6 146.6 143.8
133.4 128.1 123.3 122.9 122.3 121.3 119.5
112.1 108.2
80.2
62.9 56.0 46.3 39.2 26.1 25.2
210 200 190 180 170 160 150 140 130 120 110 100 90 80 70 60 50 40 30 20 10 0 -10

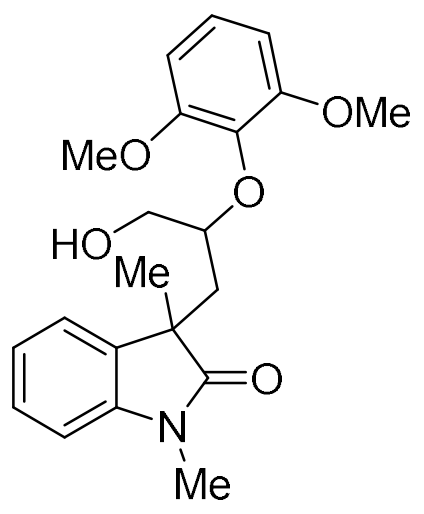

18

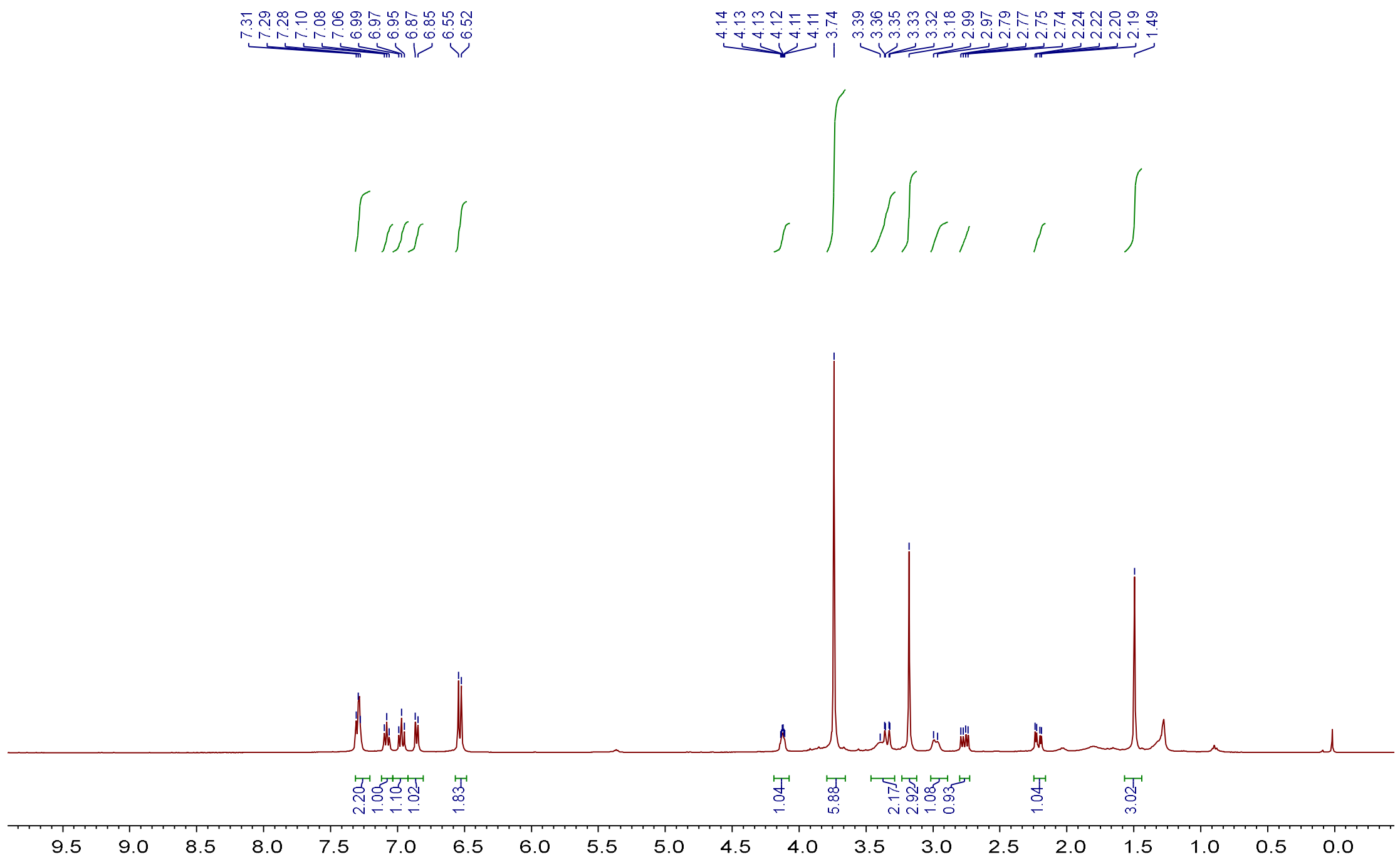

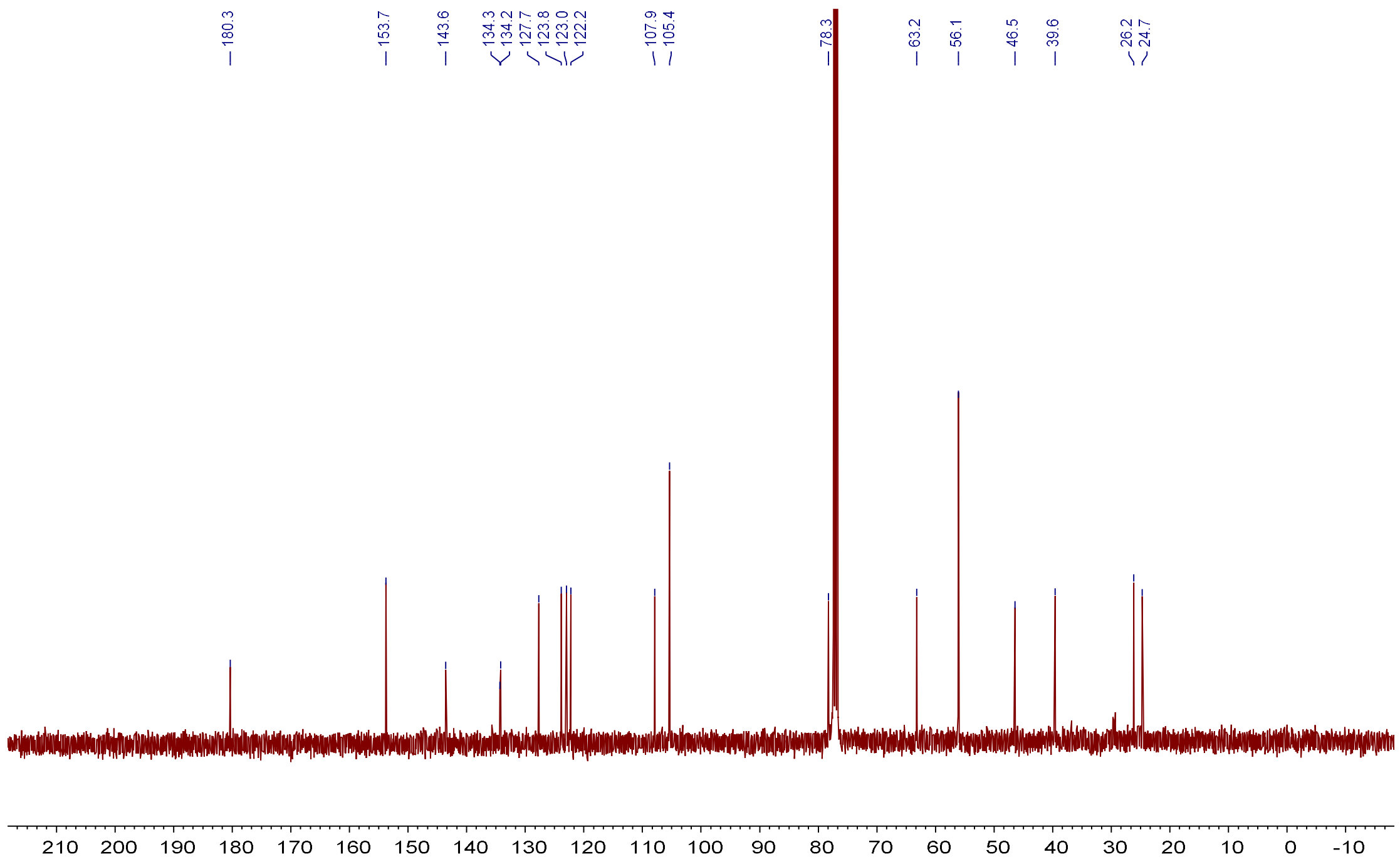

Supplementary Fig. 31. NMR spectra

File S1. Cartesian coordinates of all optimized intermediates and transition states. (upload with source data)
